
JOURNAL INFORMATION
==============================
NLM Title Abbreviation: Antimicrob Resist Infect Control
Journal ID: Antimicrob Resist Infect Control
NLM Journal ID: 101585411

Antimicrobial Resistance and Infection Control

EISSN: 2047-2994
Publisher: BMC

ARTICLE INFORMATION
==============================
PMCID: PMC5496020
DOI: 10.1186/s13756-017-0201-4
Article ID: 201
Article version: 1
Subjects: Meeting Abstracts

Meeting abstracts from International Conference on Prevention & Infection Control (ICPIC 2017)

Geneva, Switzerland. 20-23 June 2017

Electronic publication date: 2017 Jun 15
Publication date: 2017
Volume: 6
Issue: Suppl 3
Issue sponsor: Publication of this supplement was funded by ICPIC.
Electronic Location ID: 52
Copyright: © The Author(s). 2017
License: Open AccessThis article is distributed under the terms of the Creative Commons Attribution 4.0 International License (http://creativecommons.org/licenses/by/4.0/), which permits unrestricted use, distribution, and reproduction in any medium, provided you give appropriate credit to the original author(s) and the source, provide a link to the Creative Commons license, and indicate if changes were made. The Creative Commons Public Domain Dedication waiver (http://creativecommons.org/publicdomain/zero/1.0/) applies to the data made available in this article, unless otherwise stated.
License URL: https://creativecommons.org/licenses/by/4.0/

Conference name: International Conference on Prevention & Infection Control (ICPIC 2017)
Conference location: Geneva, Switzerland
Conference date: 20-23 June 2017
issue-copyright-statement: © The Author(s) 2017

==============================

### INNOVATION ACADEMY

#### Innovation Academy

##### I1

**Biological disinfection with bacteriophages: experience and perspectives**

Vasily Akimkin[1], Nikolay Shestopalov[1], Vladimir Shumilov[2], Tatiana Salmina[2], Aleksandra Dabizheva[3], Petr Kanygin[3], Izabella Khrapunova[1], Tatiana Shestopalova[1], Lyudmila Fedorova[1]
[1]Federal Budget Scientific Institution “Scientific Research Disinfectology Institute” of Federal Service for Surveillance on Consumer Rights Protection and Human Well-being; [2]"Municipal Clinical Hospital №67 named after L.A. Vorokhobov” of Moscow Healthcare Department; [3]Research and Production Association "Microgen" of the Ministry of Health of the Russian Federation, Moscow, Russian Federation
**Correspondence:** Vasily Akimkin
*Antimicrobial Resistance and Infection Control* 2017, **6(Suppl 3):**I1

**Introduction**

Application of bacteriophages for biological disinfection to decontaminate environmental objects in epidemiologically significant departments of medical organizations (intensive care units, burns units, surgical departments) represents one of the modern directions for bacteriophage usage. Indicative results of biological disinfection carried out in 2015-2016 in a large surgical hospital are reported.

**Objectives**

Epidemiological objective of conducting biological disinfection was determined by a necessity to eliminate hospital *P. aeruginosa* strains from the environment of purulent surgery department.

**Methods**

Product "Pyobacteriophag polyvalent" (lot No. 60, produced by Research and Production Association "Microgen" (Russia)) containing a mixture of sterile filtrates of phagolysates of staphylococci, streptococci, enterococci, proteus, klebsiella (pneumoniae and oxytoca), *P. aeruginosa* and *E. coli* was used for 4-time treatment of surfaces in corresponding department with determination and further checking of 140 control points. A biological disinfection of more than 700 $m^2$ was performed per a single treatment.

**Results**

The results of the biological disinfection were as follows:

1. Complete elimination of hospital strains of *P. aeruginosa*, *K. pneumoniae* in one month after treatment.
2. Significant decrease of *E. coli* bacteria group (more than 3 times) and 2-fold decrease of *S. aureus* strains.
3. Pronounced positive dynamics of "microbiological cleanliness flora" that was not present in the department before: appearance and persistent prevalence of *Bacillus cereus* and *Bacillus subtilis* in microbiological studies suggesting displacement of nosocomial HAI-inducing microorganisms from the environment.

**Conclusion**

The biological disinfection by correponding bacteriophage preparation was found to be an effective measure for the environmental disinfection. The effect of the conducted treatment was observed for 10 months, during which there were no nosocomial cases of patients diseases caused by hospital strain of *P. aeruginosa*.

**Disclosure of Interest**

None Declared.

##### I2

**Identification of anti-virulence compounds for combating staphylococcus aureus infections by High-Throughput Screening (HTS)**

Richard Y. Kao, Peng Gao
Microbiology, The University of Hong Kong, Hong Kong, Hong Kong
**Correspondence:** Richard Y. Kao
*Antimicrobial Resistance and Infection Control* 2017, **6(Suppl 3):**I2

**Introduction:** The indiscriminately use of antimicrobial drugs has led to the rapid emerging of multidrug resistant (MDR) bacteria including methicillin resistant *Staphylococcus aureus* (MRSA). Treatment by killing bacteria using antibiotics seems not to be an effective and sustainable way of controlling infections. Alternative ways for treating bacterial infections without incubating the emergence of drug resistant bacteria are highly valued.

**Objectives:** dentification of therapeutic agents that suppress the expression and production of *S. aureus* virulence factors without inhibiting bacteria growth.

**Methods:** The promoters of major virulence factors of *S. aureus* were cloned into a reporter vector using bacterial luciferase (Lux) and green fluorescent protein (GFP) as the reporter genes. Promoter activities were monitored by the measurement of luminescence and fluorescence readings. HTS of a chemical library with 50,240 compounds was carried out using *S. aureus* harboring an alpha-hemolysin gene (hla) promoter reporter plasmid and compounds that reduced the hla promoter activities considerably were selected as hits. Selected hit compounds were tested on other *S. aureus* virulence promoters for the identification of compounds that could suppress multiple virulence gene expressions. Compounds with potent suppressive effects on multiple virulence promoters were selected for further examinations using mammalian cell-based infection assays and mice *in vivo* infection models.

**Results:** *S. aureus* hla promoter together with 14 other promoters of major virulence factors or virulence associated genes were successfully constructed. HTS of 50,240 compounds using the hla promoter-based reporter system yielded 670 hits that exerted suppressive effects in hla promoter activities. The anti-virulence activities of one compound were successfully demonstrated in mammalian cell-based infection assays and a mice *in vivo* infection model.

**Conclusion:** HTS of 50,240 compounds were successfully implemented for the identification of anti-virulence compounds for *S. aureus* infections. Hit compounds with suppressive effects on multiple virulence gene promoters were identified and mammalian cell-based infection assays and a mice *in vivo* infection model showed the potential of applying anti-virulence compounds in treating *S. aureus* and other bacterial infections.
**Disclosure of Interest**
None Declared.

### I3

#### Constructional/structural infection control strategy - architectural analysis, rating and solution strategy to control infectious pathways in building systems

Jan Holzhausen, Wolfgang Sunder
IIKE, TU Braunschweig, Braunschweig, Germany
**Correspondence:** Jan Holzhausen
*Antimicrobial Resistance and Infection Control* 2017, **6(Suppl 3):**I3

**Introduction:** In order to achieve a significant improvement in the fight against infection, innovations must be generated across scientific boundaries. This development of new anti-infective strategies can only be achieved through a highly transdisciplinary approach.
**Objectives:** In the context of this "one-health-concept" [1] and the holistic approach to infection prevention [2], the study deals with the understanding of direct and indirect infection transmission in the interplay of process, actor (person, vector, etc.), space or architecture and the influence of these parameters towards optimized conditions for structural infection prevention.
**Methods:** The classical medical aspects of the epidemiology are extended by the procedural and spatial references in the sentence to the built environment (architecture/infrastructure). The work crystallizes through this procedure the neuralgic parameters in the building infrastructure, which must be considered for structural infection prevention. After analyzing the structural parameters in relation to the chain of infection and assessing the relevance of the infection disease and its localization, the work on this study records the requirements placed on the structural components and their influencing processes within the scope of the infection prevention. These requirements can be defined by criteria.
**Results:** In order to establish these criteria in a structured manner and thus to establish a holistic strategy in the highly complex field of infection prevention across all types of buildings, the present work develops a classification model for the prevention of structural infectious diseases as an entry into a prevention strategy.
**Conclusion:** The development of a solution strategy with the aid of a classification model could support the work of the planners/architects under the aspect of infection prevention and thus make the corresponding buildings more secure.

**References**
[1] American Veterinary Medical Association (Hg.) (2008): One Health: A New Professional Imperative. One Health Initiative Task Force: Final Report.
[2] Castillo-Chavez, Carlos; Curtiss, Roy; Daszak, Peter; Levin, Simon A.; Patterson-Lomba, Oscar; Perrings, Charles et al. (2015): Beyond Ebola: lessons to mitigate future pandemics. In: *The Lancet. Global health* 3 (7), e354-5.

**Disclosure of Interest**
None Declared.

### I4

#### ISEE-resistance: using *In Silico* Experimental Evolution to sensitize providers on antibiotic resistance

Guillaume Beslon[1,2], Dominique Schneider[3]
[1]Computer Science, INSA-Lyon; [2]Beagle Team, INRIA, Villeurbanne; [3]TIMC-IMAG, Université Grenoble-Alpes, Grenoble, France
**Correspondence:** Guillaume Beslon
*Antimicrobial Resistance and Infection Control* 2017, **6(Suppl 3):**I4

**Introduction**
To improve awareness and understanding of antimicrobial resistance, direct experiment with serious games offers an interesting complement to communication programs. Games help sensitizing the public by simulating the population dynamics of microbial resistance. However, modeling its evolutionary dynamics is much more difficult as it requires to model the interaction between genomic, phenotypic and population levels. Yet, such multi-scale models are mandatory to efficiently train health professionals.
**Objectives**
We propose a new approach to develop serious games by using *In Silico* Experimental Evolution (ISEE) as a game engine. ISEE is a recent research field in which simulated bugs evolve *in silico* through the joint pressure of both a mutation and a selection model. It enables to conduct large-scale simulation experiments to decipher the intertwined pressures that drive evolution.
**Methods**
Aevol (see www.aevol.fr and references therein) is an ISEE platform that models bacteria at the genomic level and includes an explicit mutational process. In Aevol, the bacterial phenotype is modeled by a mathematical function, thus allowing to simulate efficiently the evolution of large populations over thousands of generations. ISEE-Resistance uses Aevol as the engine of a serious game devoted to teach antibiotic resistance to healthcare providers. We divided the phenotypic function into a set of “core” and “resistance” traits. By submitting bacterial populations to different antibiotic dosages, one can observe the emergence of resistance traits through mutations and their spreading in the population owing to the selection pressure caused by the antibiotic treatment. One can then analyze the causes of resistance fixation and the effect of treatment strategies on the fate of the infection.
**Results**
First experiments have shown the ability of the game engine to finely follow the dynamics of antibiotic resistance emergence and spreading under *e.g.*, inappropriate dosage or discontinued drug usage.
**Conclusion**
Our aim is now to turn the ISEE-Resistance core engine into a full game by developing a user-friendly interface and by offering various scenarii mimicking real situations. We then wish to provide it as an e-learning tool in faculties of medicine and sciences.
**Disclosure of Interest**
None Declared.

### I5

#### HAITooL – using innovative design science to collaboratively implement an antibiotic stewardship decision-supporting smart system

Luis V. Lapao[1], Alexandra S. Simões[1], Mélanie R. Maia[1], João P. Gregório[1], Pedro Póvoa[2]
[1]Global Health and Tropical Medicine, Global Health And Tropical Medicine - IHMT - Universidade Nova De Lisboa; [2]Intensive Care Unit, Hospital São Francisco Xavier, Centro Hospitalar de Lisboa Ocidental, Lisbon, Portugal
**Correspondence:** Luis V. Lapao
*Antimicrobial Resistance and Infection Control* 2017, **6(Suppl 3):**I5

**Introduction**
Healthcare-associated infections (HAI) caused by antibiotic-resistant pathogens are linked with high-levels of morbidity and mortality. To prevent and control antibiotic-resistant HAI, strategies based on surveillance/monitoring systems are imperative, especially if they are well-matched with the local social-cultural background.
**Objectives**
To decrease antimicrobial-resistant HAI an antibiotic-prescription decision-supporting-system (HAITool) was co-designed to reduce antibiotic misuse and HAI.
**Methods**
Three public hospitals participate in the research, following the Design Science Research Methodology: (i) problem identification; (ii) solution definition by eliciting an Antibiotic Stewardship information system (IS); (iii) design, collaboratively with healthcare workers (aligning working processes), a toolkit that assist physicians and infection control team to manage antibiotic use and antibiotic-resistant HAI; (iv) implementation of the toolkit in the hospitals; and (v) toolkit evaluation in the control of antibiotic-resistant HAI.

**Results**

To feed the toolkit, patient, microbiology and pharmacy data are extracted, from the current hospitals IS by web services, in real-time. The information is then processed and aggregated in a unique database. A display module allows real-time visualization through innovative graphics presentation: Inform about the accuracy of antibiotic prescription, providing timely and appropriate information related with antibiotics use; monitoring the data about antibiotic use and resistant bacteria. The evaluation of the toolkit, based on a focus group questioner about the toolkit functionalities, revealed that it was considered helpful in monitoring antibiotic use, helping antibiotic prescription, and can be used to improve infection control interventions (e.g. improve communication between professionals).

**Conclusion**

This toolkit brings digital innovation to support health professionals' performance and it is an important step forward for the reduction of antibiotic misuse and in the control and prevention of antibiotic-resistant HAI, and overall patient safety.

**Disclosure of Interest**

L. Lapao Employee of: IHMT-UNL, Grant/Research support from: EEA Grants, A. Simões Employee of: IHMT-UNL, Grant/Research support from: EEA Grants, M. Maia: None Declared, J. Gregório: None Declared, P. Póvoa: None Declared.

### I6

### Welcome on board! – An edutainment movie to promote basic infection prevention measures

Aline Wolfensberger, Marie-Theres Meier, Lauren Clack, Hugo Sax
Department of Infectious Diseases and Hospital Epidemiology, University Hospital Zürich, Zürich, Switzerland
**Correspondence:** Aline Wolfensberger
*Antimicrobial Resistance and Infection Control* 2017, **6(Suppl 3):**I6

**Abstract video clip: Introduction**

Standard Precautions (SP) include a group of infection prevention and control (IPC) practices that apply to all patients and are meant to ensure safety for patients, healthcare workers (HCW), and visitors. The HCW knowledge of SP, however, often proves to be scarce. We therefore produced an educational video about SP to improve HCW knowledge at the University Hospital Zurich, Switzerland, and potentially in healthcare institutions worldwide. Since it is well known that emotions help learners to focus and facilitate uptake of information into long-term memory, we chose to use humor as a central feature in this project, making it what is called 'edutainment'.

**Methods**

As safety management in healthcare and aviation are often compared, we decided to produce a 'mash-up' between an in-flight safety video and infection prevention instructions. The audience witnesses a cabin crew/infection prevention team member giving instructions to a novice cabin crew member/healthcare worker. Six fundamental topics of SP are covered in the 5' movie: hand hygiene, use of personal protective equipment, professional appearance, respiratory hygiene, aseptic technique, environmental cleaning, and device disposal and reprocessing. The scenes were set inside an airplane (mock-up at a Swiss aviation crew training facility) with passengers appearing as patients. Fun and surprising moments chase each other throughout the script, including fast wordplay, exaggerations, and slapstick. The film was conceived and executed in a collaboration between ICP professionals and a professional film director and crew including a cast of two actors, 20 extras, a camera operator, a sound technician, a gaffer, a costume designer, a make-up artist, and two production assistants

**Disclosure of Interest**

None Declared.

### I7

### integrating patients' experiences, understandings and enactments of infection prevention and control into clinicians' everyday care: a video-reflexive-ethnographic exploratory intervention

Mary Wyer[1], Rick Iedema[2], Suyin Hor[1]
[1]Westmead Institute for Medical Research Research, Sydney, Australia; [2]Kings College, London, United Kingdom
**Correspondence:** Mary Wyer
*Antimicrobial Resistance and Infection Control* 2017, **6(Suppl 3):**I7

**Introduction**

Efforts to promote patient empowerment and involvement have become core components of global and national infection prevention and control initiatives. However, relatively little is known about how frontline healthcare professionals understand, implement or support patient involvement in general, and still less is known about patient involvement in infection control.

**Objectives**

This PhD study used video-reflexive ethnography to explore and strengthen clinicians' awareness of and commitment to patient involvement in infection prevention and control.

**Methods**

Hospital inpatients were invited to scrutinise footage of their own clinical care to look for cross-contamination risks. Group reflexive sessions were then conducted with nurses in which footage of everyday patient care interactions were presented alongside patients' observations of the same events.

**Results**

The findings show that patients were actively contributing to IPC in ways that clinicians and researchers were not fully aware of. Some of the strategies were effective and some were counterproductive. Engaging with these contributions enabled the clinicians to appreciate the importance of discussing cross-contamination risks and risk containment behaviours with patients.

**Conclusion**

The study enabled clinicians to understand how the quality of their patient-provider relationships and IPC conversations shaped patients' attention and precautions around infection risks and behaviours and motivated clinicians to develop strategies to promote greater patient involvement.

**Disclosure of Interest**

None Declared.

### I8

### The potential and risks of internet-of-things for patient safety – using indoor-location systems to improve nurses' hand hygiene performance

Luis V. Lapao[1], João P. Gregório[1], Pedro Póvoa[2]
[1]Global Health and Tropical Medicine, Instituto de Higiene e Medicina Tropical - Universidade Nova de Lisboa; [2]Intensive Care Unit, Hospital São Francisco Xavier, Centro Hospitalar de Lisboa Ocidental, Lisbon, Portugal
**Correspondence:** João P. Gregório
*Antimicrobial Resistance and Infection Control* 2017, **6(Suppl 3):**I8

**Introduction**

Hospital-acquired infections are still a major patient safety problem. Their occurrence can lead to higher morbidity and mortality rates, increased length of stay and higher costs for both hospital and patients. Performing hand hygiene (HH) is a simple and inexpensive prevention measure, but healthcare workers' compliance with it is often far from ideal.

**Objectives**

To raise awareness regarding HH compliance, individual behaviour change and performance optimization, we aimed to develop an Internet-of-Things (IoT) solution that collects data and provides real-time feedback accurately in an engaging way.

**Methods**

A Design Science Research Methodology (DSRM) was used in this research. DSRM is useful to study the link between research and professional practices by designing, implementing and evaluating systems that address a specific need. It follows a development cycle composed by six activities. Two work iterations were performed applying gamification components, each using a different indoor location technology. Preliminary experiments, simulations and field studies were performed in an Intensive Care Unit (ICU) of a Portuguese tertiary hospital. Nurses working on this ICU were engage during the research, participating in several sessions across the implementation process.

**Results**

Nurses enjoyed the concept and considered that it allows for a unique opportunity to receive feedback regarding their performance. Tests performed on the indoor location technology applied in the first iteration regarding distances estimation presented an unacceptable lack of accuracy. Using a proximity-based technique, it was possible to identify the sequence of positions but with low precision. In the second work iteration, a different indoor location technology was explored but it did not work properly, showing the limitation of present IoT technology to respond to the ward demands.

**Conclusion**

Combining automated monitoring systems with gamification seems to be an innovative and promising approach based on the already achieved results. Involving nurses in the project since the beginning allowed to align the solution with their needs. Despite strong evolution through recent years, IoT technologies are still not ready to be applied in the healthcare setting.

**Disclosure of Interest**

L. Lapao Employee of: IHMT-UNL, Grant/Research support from: FCT-Portugal, J. Gregório: None Declared, P. Póvoa: None Declared.

### I9

**Withdrawn**

### I10

#### How we talk about hand hygiene matters – an exploration of hand hygiene etymology

Claire Kilpatrick, Jules Storr
S3 Global, London, United Kingdom
**Correspondence:** Claire Kilpatrick
*Antimicrobial Resistance and Infection Control* 2017, **6(Suppl 3):**I10

**Introduction**

Numerous studies have focused on health care workers' perceptions of hand hygiene but few have addressed the etymology of hand hygiene and its influence. Words influence behaviour. The increasing use of social marketing in infection prevention is testament to the value of words in campaigning for change. However, an exploration of the use of words related to hand hygiene improvement and the World Health Organisation's (WHO) recommendations has until now received scant attention. Compliance with hand hygiene remains sub-optimal across the globe and novel approaches for behavioural impact have the potential to offer valuable adjuncts to current strategies.

**Objectives**

To describe the feelings evoked by five words commonly used in a hand hygiene context.

**Methods**

An exploratory exercise assessed the feelings evoked by five words commonly used in a hand hygiene context. A classic psychology experiment was used to evoke an instant, emotional reaction. From June 2013 to May 2014, a total of 23 face to face exercises were undertaken in seven different countries. This convenience sample totaled 2100 people consisting of nurses, doctors, senior management and a diverse range of other health workers and managers. The words tested were alcohol based handrub, compliance, monitoring, moment and system. Qualitative analysis of the findings was undertaken.

**Results**

Responses i.e. the words captured, were categorized as "warm" and "cold". During the exercises 240 words representing alcohol based handrub were collected, 510 representing compliance, 402 representing monitoring, 480 representing moment and 200 representing system. Compliance in particular evoked negative feelings, with 'cold words' being described on hearing this word. The word moment evoked the most positive reactions ('warm words').

**Conclusion**

WHO guidelines state that clear and uniform language in hand hygiene matters. Social marketing falls into the fourth component of the WHO multimodal strategy, described as "reminders in the workplace". This novel exercise has potential to stimulate the infection prevention (and academic) community to revisit the words it uses within policies/guidelines and day-to-day communications in their quest to bring about the socially desired change [hand hygiene at the right time] as a part of a multimodal approach.

**Disclosure of Interest**

C. Kilpatrick Consultant for: GOJO Industries, J. Storr Consultant for: GOJO Industries.

### I11

#### Impact of the "save lives: clean your hands 5th of May" campaign on the press: 2005-2016

Virginie Zimmerli[1], Mélissa Baudrillart[1], Daniela Pires[2], Franck Schneider[3], Tcheun-How Borzykowski[3], Carolina Fankhauser[3], Patrick-Yves Badillo[1], Didier Pittet[2,4], on behalf of Communication Language Internet Project in Health (CLIP-H)
[1]Medi@LAB-Genève, University of Geneva; [2]Infection Control Unit; [3]University of Geneva Hospitals; [4]Faculty of Medicine, Geneva, Switzerland
**Correspondence:** Virginie Zimmerli
*Antimicrobial Resistance and Infection Control* 2017, **6(Suppl 3):**I11

**Introduction**

In 2005, the World Health Organization (WHO) launched "Clean Care is Safer Care" program to promote hand hygiene (HH) worldwide. In addition, since 2009, the "Save Lives: Clean Your Hands" global campaign calls health care workers (HCWs) to actively engage with HH every 5$^{th}$ of May. This global Hand Hygiene Day is also a moment to promote awareness on the prevention of health care-associated infections (HAI) to policy makers, stakeholders, patients and the general public.

**Objectives**

We aimed to evaluate for the first time the impact of the 5$^{th}$ of May campaign on the press.

**Methods**

We used the Nexis database to identify English-language press articles containing at least one of 9 keywords related to HH. We analysed the number and the evolution of HH related articles published on the 5$^{th}$ of May every year from 2005 to 2016. In those years, we have additionally compared the number of HH articles published in 5$^{th}$ of February, 5$^{th}$ of May and 5$^{th}$ of November to ascertain the impact of the global HH Day. The keywords chosen had been previously selected and tested in the Nexis database to assess accuracy of press articles identified.

**Results**

In 2009, the first year of the global HH Day, the number of articles published on the 5$^{th}$ of May was higher than on any other year (2005:115, 2007:54, 2009:419, 2012:78, 2014:159, 2016:253). The total number of articles relating to HH on the 5$^{th}$ of May in press has more than doubled in 12 years. Additionally, after 2009, we found that there was a tendency to have more articles about the theme of HH published on the 5$^{th}$ of May than on the 5$^{th}$ of February or 5$^{th}$ November.

**Conclusion**

The highest number of HH related articles was attained in 2009 and this number has never been repeated after. Furthermore, from 2009, there has been more often HH related articles in the press on 5$^{th}$ of

May than on other days of the year. Even if it remains difficult to prove causality, it is very probable that the "Save Lives: Clean Your Hands" global campaign celebrated every 5th of May since 2009 has a significant impact on the number of articles in the press related to HH.

**Disclosure of Interest**

None Declared.

#### I12

#### Uses of twitter in health: the case of hand hygiene and infection control

Melissa Baudrillart[1], Virginie Zimmerli[1], Daniela Pires[2], Franck Schneider[2], Tcheun-How Borzykowski[2], Carolina Fankhauser[2], Patrick-Yves Badillo[1], Didier Pittet[2,3], on behalf of Communication Language Internet Project in Health (CLIP-H)
[1]Medi@LAB-Genève, University of Geneva; [2]Infection control Unit, University of Geneva Hospitals; [3]Faculty of Medicine, Geneva, Switzerland
**Correspondence:** Melissa Baudrillart
*Antimicrobial Resistance and Infection Control* 2017, **6(Suppl 3):**I12

**Introduction**

Since 2005 the World Health Organization (WHO) "Clean Care is Safer Care" program has been promoting hand hygiene (HH) worldwide. There has been an increase use of social networks, Twitter in particular, to disseminate health care messages, but little is known about its actors and networks.

**Objectives**

We aimed to identify who were the main communicators of information about HH in Twitter and explored their connections and use of Twitter.

**Methods**

Between 9th Jan and 5th Feb 2017, we used Twitter API (Application Programming Interface) to continuously collect tweets containing 17 previously tested key terms related to HH (words and hashtags). Users who tweeted more than 10 times were identified and categorized according to their sector of activity. Additionally, we analysed the network of these users based on their subscriptions on Twitter by performing follow relationships graph and social networks analysis.

**Results**

A total of 14'638 unique tweets were collected from 11'724 Twitter accounts. Of these users, 10'605 (90.5%) tweeted only once during this period and 47 (0.4%) more than 10 times. These 47 users were: healthcare workers (HCWs) (8), companies (13), alcohol-based handrub sellers (14), media (newspaper, magazine) (2) and others (10). Based on the follow relationships graph, our results showed that a user belonging to the HCWs category receives more Twitter subscriptions, is more retweeted and is more mentioned in tweets than users from other categories. Furthermore, social networks analysis indicated that these 47 accounts used more Twitter to disseminate information on HH (low information centralization in the network:5.42%) than to grow their network (low connectivity between users:5.4%).

**Conclusion**

Our results show that Twitter is actively used by several actors to disseminate HH information. We found that although HCWs are relatively few among those who use Twitter the most, they are the ones who generate more engagement, making them probably the ideal ambassadors to promote HH on Twitter. An important step to improve the use of Twitter as a tool to promote HH would be to urge the different stakeholders to connect between each other and create a community.

**Disclosure of Interest**

None Declared.

#### I13

#### Infection control on the movie screen

Borbála Szél, Kamilla Nagy
Infection Control Unit, Albert Szent-Györgyi Health Center at University of Szeged, Szeged, Hungary
**Correspondence:** Borbála Szél
*Antimicrobial Resistance and Infection Control* 2017, **6(Suppl 3):**I13

**Introduction**

Due to the unstoppable spread of the media, patients receive information about their health not only form health care facilities but also from the TV by commercials, talkshows, documentary films, even series and movies. It is well proven that these audiovisual tools have great impact on people's behaviour and opinion. Furthermore they hold the unique possibility of reaching out and educating the people not involved directly in healthcare or maybe even a whole nation without them knowing about it.

**Objectives**

The aim of this study was to reveal the infection control scenes in non-healthcare-themed movies.

**Methods**

Randomized data were gathered from a reliable internet movie database. The samples (87 movies) were non-healthcare-themed movies released from 1984 to 2016. The exclusion criteria were the absence of infection control scenes.

**Results**

13 movies (containing 19 infection control scene) met the criteria and were involved in the study. The samples could be divided into 4 main groups based on their main infection control themes: hand hygiene (9 scenes), infection control in general (4 scenes), surface disinfection and sterilization (3 scenes), hospital hygiene for laypeople (3 scenes). It is worth noting that movies released after 2005 presented more likely infection control scenes.

**Conclusion**

In conclusion, this novel study shed new light on infection control, because 15% of the samples indicated infection control as an intuitive action, a model to follow or a precaution and raising awareness with it. It seems that worldwide hand hygiene campaigns and infection prevention programs excercised influence also on the movie industry, because movie directors are presenting this topic in comedies, romantic and action movies. These movies are excellent examples for the importance of patient education at a base level while hopefully leading to more educated patients having better compliance thus more effective patient safety can be accomplished.

**Disclosure of Interest**

None Declared.

## ORAL PRESENTATIONS

### Environment, cleaning and *Clostridium difficile*

#### O1

#### Risk of nosocomial clostridium difficile infection following exposure to antimicrobial agents

Michael Rubin[1,2], Vanessa Stevens[1,2], Molly Leecaster[1,2], Jian Ying[2], Tao He[1,2], Brian Sauer[1,2]
[1]VA Salt Lake IDEAS Center, Department of Veterans Affairs; [2]Internal Medicine, University of Utah, Salt Lake City, United States
**Correspondence:** Michael Rubin
*Antimicrobial Resistance and Infection Control* 2017, **6(Suppl 3):**O1

**Introduction**

*Clostridium difficile* infection (CDI) is one of the most common nosocomial infections worldwide. While exposure to antibiotics is the most important risk factor for CDI, the magnitude of the risk from different antibiotics has not been well quantified through big data analysis of large healthcare systems.

**Objectives**

Estimate the risk of nosocomial CDI (nCDI) from exposure to different antibiotic classes using nationwide data from all US Veterans Affairs (VA) hospitals.

**Methods**

We used a historical cohort of patients admitted to acute care wards of all US VA hospitals between 1/1/08 and 12/31/13. CDI was defined according to the US Centers for Disease Control laboratory-identified (LabID) event definition. nCDI was defined as a first positive LabID event occurring >72 h after admission, occurring on the collection date of the positive test. Patient-level characteristics were collected for each hospitalization and for one year prior to admission. Patients with a history of CDI within 56d of admission were excluded. Antimicrobials were grouped according to suspected CDI risk (high, medium, or neutral) based on literature and expert opinion. Each patient day was categorized as Pre-, On, or Post-treatment according to exposure to the three groups. Days following CDI diagnosis were excluded. Survival analysis was performed using exposure category defined above as a time-varying covariate to assess the risk of CDI by antimicrobial group.

**Results**

A total of 1,138,822 first admissions covering 6,521,327 patient-days were included, with 3,760 first episodes of nCDI. Relative to days without antimicrobial exposure, the hazard ratio for developing nCDI was 2.11 while on high-risk ($p < 0.001$), 1.66 while on medium-risk ($p < 0.001$), and 0.72 while on neutral antimicrobials ($p = 0.01$). In the post-treatment period, the hazard ratios were 1.98 ($p < 0.001$), 2.63 ($p < 0.001$), and 0.89 for high-, medium-, and neutral-risk antimicrobials, respectively.

**Conclusion**

This big data analysis from a single, large healthcare system has helped to better quantify the risk of nCDI during and after receiving different categories of antimicrobials. Further work will assess the risk associated with individual antimicrobial classes.

**Disclosure of Interest**

None Declared.

### O2

#### Reduction in clostridium difficile infection associated with the introduction of a hydrogen peroxide disinfection system

Adriano Anesi[1], Vanina Rognoni[1], Sara Asticcioli[1], Matteo Gelosa[2], Rachele Accetta[3], Marco Ferrari[3]
[1]Laboratory of Microbiology; [2]Unit of Hospital Pharmacy; [3]Unit of Hospital Hygiene, ASST di Lodi, Lodi, Italy
**Correspondence:** Adriano Anesi
*Antimicrobial Resistance and Infection Control* 2017, **6(Suppl 3):**O2

**Introduction**

The efficacy of dry-mist hydrogen peroxide decontamination has been determined in various healthcare settings.

**Objectives**

The aim of this work is to evaluate the clinical impact of implementing hydrogen peroxide and silver cations micro-nebulization disinfection of rooms vacated by patients with *Clostridium difficile* infection (CDI).

**Methods**

The levels of CDI incidence in the wards of ASST of Lodi, (Italy) were monitored for three 12-month periods between 2014 and 2016. In 2015, a decontamination system based on a solution of 5-8% hydrogen peroxide and 60 ppm active silver ions (HyperDRYMist®, 99Technologies) was added as the sole additional hygiene and prophylaxis measure after room change at patient's dismissal of all CDI's affected individuals. A 'breakpoint' time series analysis model was used to detect any significant changes in the monthly CDI rate per 1000 patient-days.

**Results**

In total, 160 patient rooms previously occupied by infected/colonised patients were disinfected in 2015 and 135 patient rooms in 2016. The compliance to the procedure of disinfection was around 80% in 2015 and 95% in 2016. The CDI rate decreased from 1.73 cases per 1000 patient-days in the 12 months before HDM® usage to 1.32 compared with the first 12 months of HDM® usage and to 0.93 compared with the second 12 months of HDM® usage (60% reduction). The breakpoint model identified significant changes in the CDI rate. The first occurred in August 2014, with 95% confidence intervals around this breakpoint spanning the spring and summer months, suggesting that this first breakpoint is explained by seasonal variation. The second breakpoint occurred in March 2016, which was when HDM® disinfection procedure was fully implemented. The hypotesis that is second breakpoint is likely to be explained by the introduction of HDM® disinfection is also supported by the fact that data on hand hygiene performance and data on defined daily doses (DDD) of cephalosporins, fluoroquinolones and proton pump inhibitors didn't change during the period of observation.

**Conclusion**

Our data indicate that the hydrogen peroxide and active silver ions disinfection system, should be considered to augment the terminal disinfection of rooms vacated by patients with CDI.

**Disclosure of Interest**

None Declared.

### O3

#### Improving real world evidence around hospital cleaning – the role of a pragmatic, implementation focussed trial

Lisa Hall, on behalf of the Researching Effective Approaches to Cleaning in Hospitals (REACH) Study team
Queensland University of Technology, Brisbane, Australia
**Correspondence:** Lisa Hall
*Antimicrobial Resistance and Infection Control* 2017, **6(Suppl 3):**O3

**Introduction**

A clean hospital environment plays a vital role in reducing healthcare associated infections. However strong evidence, using well designed studies is limited. Most studies have focussed on a single intervention or product, and have failed to take into account contextual issues that may influence feasibility and sustainability in hospital settings. Data on cost-effectiveness is also lacking. A challenge for researchers in this area is how to maintain study validity and integrity, whilst allowing for flexibility in implementation.

**Objectives**

To evaluate the effectiveness and cost-effectiveness of an evidence-based environmental cleaning bundle implemented in 11 different hospitals nationally, in Australia.

**Methods**

Using a randomised stepped wedge design, and an implementation science framework we systematically examined and documented existing practices and contextual factors at each trial hospital. We used this information to identify gaps and strengths in relation to the bundle components, organisational culture and readiness for change. This then informed the development of responsive implementation plans for each site.

**Results**

Improving hospital cleaning was complex. Existing cleaning practices were diverse, as were the policy, contracts, staffing and governance arrangements. Considerable effort had to be put into a developing a bespoke strategy for each site that allowed for optimal implementation, so that hospitals could transition effectively to the 'best practice' bundle. The trial design allowed researchers to stagger the intervention, and for hospitals to act as their own controls in the effectiveness analysis. Combining this with high quality economic analysis will allow us to evaluate value for money in different scenarios.

**Conclusion**

We need better quality research in infection prevention, moving beyond effectiveness to also consider context, feasibility, sustainability and cost. Pragmatic trials combining the best of epidemiology, implementation science and economic methods are an innovative approach, providing a unique insight into what works, how it works, and how much it costs, in a variety of real world settings.

**Disclosure of Interest**

None Declared.

### O4

#### AN international survey of cleaning and disinfection practices in the healthcare environment

Nikki Kenters[1], Tom Gottlieb[2], Elisabeth Huijskens[1,3], Ermira Tartari[4], Joost Hopman[5], Marin Schweizer[6], Andreas Voss[7,8], on behalf of ISC Infection and Control Working Group
[1]Department of Infection Prevention and Control, Albert Schweitzer hospital, Dordrecht, Netherlands; [2]Department of Microbiology and Infectious Diseases, Concord Hospital, Sydney, Australia; [3]Department of Medical Microbiology, Albert Schweitzer hospital, Dordrecht, Netherlands; [4]Infection Control Programme & WHO collaborating Centre of Patient Safety, Geneva Univ. Hospitals and Faculty of Medicine, Geneva, Switzerland; [5]Department of Medical Microbiology, Radboudumc, Nijmegen, Netherlands; [6]Department of Epidemiology, College of Public Health Department of Internal Medicine, Iowa, United States; [7]Department of Medical Microbiology, Canisius Wilhelmina Hospital; [8]Department of Medical Microbiology, Radboud University Medical Centre, Nijmegen, Netherlands
**Correspondence:** Nikki Kenters
*Antimicrobial Resistance and Infection Control* 2017, **6(Suppl 3):**O4

##### Introduction
Antimicrobial resistance has become an urgent global health priority. Basic hygiene practices and cleaning and disinfection of the hospital environment are key area in preventing pathogen cross-transmission. The ISC Infection Prevention and Control Working Group, represents professionals from 50 different countries.

##### Objectives
To assess adequacy of cleaning and disinfection practices in healthcare settings globally an electronic survey was developed.

##### Methods
The survey comprised of 30 multiple-choice questions. Data was collected from July 2016 to December 2016.

##### Results
A total of 110 healthcare professionals, representing 23 countries (33% Europe, 17% Australia, 28% Asia, 18% North America, 3% South America, 1% Africa) participated in the survey. Of respondents, 96% have a written cleaning policy for clinical areas and 82% for shared clinical equipment. Training of staff occurs in 70% of the facilities at employment, 46% receive yearly training, 15% twice yearly, and 20% sporadic training. Worldwide, microfiber cloths and mops are the most common method of delivery for routine cleaning (65%), followed by the cotton cloths and mops (29%). Enhanced cleaning and/or disinfection practices while patients under contact precautions (eg. MDRO) vary; no extra cleaning (15%), extra cleaning in outbreaks (31%), more frequently cleaning (19%), disinfection added to regular cleaning (9%), extra cleaning and disinfection (26%). Halogens (82%) are the most commonly used routine disinfectants, QATs and alcohols in 33%. Most of respondents rely only on visual daily monitoring for the assessment of cleaning (47%). Further survey results will be presented.

##### Conclusion
The survey enabled assessment and recognition of widely differing global practices in approaches to environmental cleaning and disinfection. Development of guideline recommendations for cleaning and disinfection could improve practices and set minimum standards.

##### Disclosure of Interest
None Declared.

### O5

#### Transmission of pathogens from dry surface biofilms: effect of glove type

Karen Vickery[1], Shamaila Tahir[1], Durdana Chowdhury[1], Mark Legge[1], Gregory Whiteley[2], Anand Deva[1], Honghua Hu[3]
[1]Faculty of Medicine and Health Sciences, Macquarie University, Nth Ryde; [2]Whiteley Corporation, Whiteley Corporation; [3]Faculty of Medicine and Health Sciences, Macquarie University, Nth Sydney, Australia
**Correspondence:** Karen Vickery
*Antimicrobial Resistance and Infection Control* 2017, **6(Suppl 3):**O5

##### Introduction
Pathogens can survive on dry hospital environmental surfaces for extended periods especially when incorporated into dry surface biofilms. Bacteria in biofilms are protected from desiccation and have increased tolerance to removal by cleaning agents and disinfectants. We have shown that bare hands can transmit biofilm bacteria from surface to surface and hence could play a significant role in healthcare associated infections (HAI).

##### Objectives
To determine the effect of glove composition on the transfer rate of *Staphylococcus aureus* from biofilm.

##### Methods
*S. aureus* biofilm was grown *in vitro* on polycarbonate coupons in the CDC bioreactor, using our validated methods of with periodic nutrition interspersed with long periods of dehydration, over a period of 12 days. Each coupon had approximately $10^7$ bacterial cells. Transmission was tested by touching coupons, with either nitrile, latex or surgical gloved hands, and then pressing the finger and thumb onto the sterile horse blood agar (HBA) surface up to 19 consecutive times. The number of colony forming units (CFU) were recorded for each touch after incubating HBA plates at 37 °C for 48 hours. The experiment was repeated following coupon treatment with 5% neutral detergent for 5 seconds.

##### Results
Bacterial cells were readily transmitted via all three types of gloves, commonly used by healthcare workers (HCWs). Although less than 1% of the biofilm was transferred, *S. aureus* was transferred in sufficient numbers to cause infection, to 19 surfaces from touching the biofilm once. Six times more bacteria were transferred by nitrile and surgical gloves when compared to latex gloves ($P < 0.001$). Wetting the biofilm with 5% neutral detergent increased the transmission rate of bacteria by seven-fold ($P < 0.01$).

##### Conclusion
Despite bacteria being incorporated into environmental biofilm and covered by exopolymeric substances (EPS or slime), bacteria are readily transferred by HCW's gloved hands and this confirms the possibility that biofilm contributes towards patient colonization with pathogens and development of HAI.

##### Disclosure of Interest
None Declared

### O6

#### Isolation of Nontuberculous Mycobacterium (NTM) from heater cooler devices, in a tertiary care center in Lebanon

Nada K. Zahreddine[1], Joseph Tannous[1], Zeina Kanafani[2], Souha Kanj Sharara[2], Salim Jamal[3], Rihab Ahmadieh[1], George Araj[4]
[1]Infection Control and Prevention Program; [2]Department of Internal Medicine; [3]Department of General Anesthesia; [4]Department of Pathology and Laboratory Medicine, American University of Beirut Medical Center, Beirut, Lebanon
**Correspondence:** Nada K. Zahreddine
*Antimicrobial Resistance and Infection Control* 2017, **6(Suppl 3):**O6

##### Introduction
An investigation was initiated at the American University of Beirut Medical Center following the FDA safety communication report about the contamination of heater cooler devices (HCDs) associated with NTM infections. Mycobacterium chimaera and other NTM species were reported internationally.

##### Objectives
To assess the effectiveness of cleaning and disinfection methods of HCDs and to retrospectively evaluate infections in patients who underwent cardiac surgery.

##### Methods
Bacterial and mycobacterial cultures were taken on January 26, 2017, from 2 HCDs (Terumo-A and Terumo-B) manufactured by Terumo and from a newly purchased HCD (Maquet-C) made by Maquet. Cultures were obtained following regular cleaning and disinfection and included the parts of the devices that are difficult for the disinfectant to penetrate such as: water tanks, filters, tubing and air outflow surface. The water source was also cultured.

**Results**

After 6 weeks of incubation, NTM was isolated from Terumo-B only. Terumo-A results were negative; speciation of the bacterial NTM culture was conducted using 16S rRNA sequencing test that identified a *Mycobacterium simiae*. The device was immediately removed from service. The method of cleaning and disinfection was reviewed and modified using sodium hypochlorite at 10% dilution following the visit of the Engineer Clinical Specialist from Terumo. Cultures were repeated and results of Terumo-B are negative so far. However, the new Maquet-C grew NTM and the isolate was sent for speciation.

**Conclusion**

Clinicians at AUBMC were alerted of the risk of NTM infections in patients who underwent cardiac surgeries and to consider it as a potential cause of unexplained chronic infection when encountered. A new procedure for cleaning and disinfection of HCDs was introduced with an ongoing schedule for cultures. Of note, M. *simiae* is the most commonly isolated NTM species from pulmonary specimen of Lebanese patients. Investigation is underway to retrospectively evaluate NTM recovered from patients who developed chronic pulmonary infections post cardiac surgery over the last 3 years.

**Disclosure of Interest**

None Declared

### Antibiotic use, stewardship and cost of resistance

#### O7

#### Outcomes of methicillin-susceptible staphylococcus aureus bacteremia in patients with and without beta-lactam allergies

Daniel J. Livorsi[1,2], Michihiko Goto[1,2], Marin Schweizer[1,2], Bruce Alexander[1], Rajeshwari Nair[2], Brice Beck[1], Kelly K. Richardson[1], Eli Perencevich[1,2]
[1]Iowa City VA Health Care System; [2]University of Iowa Carver College of Medicine, Iowa City, United States
**Correspondence:** Daniel J. Livorsi
*Antimicrobial Resistance and Infection Control* 2017, **6(Suppl 3):**O7

**Introduction:** Definitive therapy for methicillin-susceptible Staphylococcus aureus (MSSA) bacteremia with beta-lactam antibiotics is associated with improved outcomes. However, patients with beta-lactam allergies may be treated with less efficacious antibiotics.

**Objectives:** Our objective was to define the prevalence of beta-lactam allergies in patients with MSSA bacteremia and to determine whether the documentation of a beta-lactam allergy influences clinical outcomes.

**Methods:** A retrospective cohort that included all patients with MSSA bacteremia admitted to the Veterans Health Administration during 2003-2014 was analyzed. Allergic reactions were classified as Type 1 or non-Type 1 using standardized criteria. First-line beta-lactam therapy included anti-staphylococcal penicillins and first generation cephalosporins.

**Results:** There were 17,642 unique episodes of MSSA bacteremia across 115 facilities. The median age was 64 years. An allergy to a beta-lactam was documented in 2,531 (14.4%) patients. Based on the allergy's description, 746 (29.5%) cases had a potential Type 1 reaction. Type 1 reactions were associated with penicillins and cephalosporins in 93% and 7%, respectively. Patients with any beta-lactam allergy or a Type 1 allergy were less likely than non-allergic patients to receive first-line beta-lactam therapy prior to discharge (any allergy vs. no allergy: 33.3 vs. 55.2%, $p < 0.01$ and Type 1 vs. non-Type 1: 30.7 vs. 53.0%, $p < 0.01$).

The 30-day all-cause mortality rate for MSSA bacteremia was 17.5%.On univariate analysis, neither the documentation of any beta-lactam allergy or a Type 1 reaction was associated with 30-day mortality (OR 0.99, 95% CI 0.88-1.10 and OR 0.84, 95% CI 0.69-1.03, respectively).

**Conclusion:** Beta-lactam allergies were commonly documented in patients with MSSA bacteremia, but less than a third of allergies were potential Type 1 reactions. The documentation of a beta-lactam allergy was not associated with increased mortality at 30 days even though allergic patients were less likely to receive optimal antibiotic therapy. Further analyses will adjust for potential confounders of the association between documented allergy and outcomes.

**Disclosure of Interest**

None Declared.

#### O8

#### Barriers and facilitators of responsible systemic antibiotic use from the patient's perspective: a systematic review

Benedikt Huttner[1,2], Veronica Zanichelli[1], Gianpiero Tebano[3], Inge Gyssens[4], Celine Pulcini[5], Vera Vlahović-Palčevski[6], Annelie Monnier[4], Mirjana Stanic Benic[6], Stephan Harbarth[2], Marlies Hulscher[7], on behalf of DRIVE-AB consortium
[1]Infection Control Program; [2]Division of Infectious Diseases, Geneva University Hospitals and Faculty of Medicine, Geneva, Switzerland; [3]Université de Lorraine, EA 4360 APEMAC, Nancy, France; [4]Department of Internal Medicine, Radboud University Medical Center, Nijmegen, Netherlands; [5]CHRU de Nancy, Service de Maladies Infectieuses et Tropicales, Nancy, France; [6]Department of Clinical Pharmacology, University Hospital Rijeka, Rijeka, Croatia; [7]Scientific Center for Quality of Healthcare, Radboud University Medical Center, Nijmegen, Netherlands
**Correspondence:** Benedikt Huttner
*Antimicrobial Resistance and Infection Control* 2017, **6(Suppl 3):**O8

**Introduction:** Both appropriate and inappropriate antibiotic use can be affected by patient-related factors.

**Objectives:** To perform a systematic review assessing patient-related factors potentially influencing antibiotic use.

**Methods:** Studies published in MEDLINE until 30/09/2015 were identified using combinations of terms for concepts "barriers/facilitators", "antibiotics" and "patients". Qualitative studies reporting determinants of antibiotic use from the patient's perspective and quantitative studies reporting factors associated with antibiotic use practices were included. Factors were categorized as "barriers" (B) (eg. factors associated with a higher likelihood of self-medication) or "facilitators" (F) (eg. factors associated with higher compliance to the prescribed treatment) of responsible antibiotic use.

**Results:** 87 studies met inclusion criteria (12 qualitative and 75 quantitative studies)

We identified 7 categories of patient-related factors:

- Demographic and socio/economic factors (eg. age (B/F depending on the study); B > F; meaning that in this category barriers > facilitators).
- Patient-doctor interactions (eg. counseling (receiving counseling F); F > B).
- Characteristics of the received regimen (eg. administration frequency (multiple daily doses B); B > F).
- Attitudes (eg. expecting antibiotics (demanding antibiotics B); B > F).
- Access to treatment (eg. patients' direct costs (lower costs F); B > F).
- Characteristics of the condition for which the antibiotic was prescribed (eg. duration of symptoms (longer duration B); B > F).
- Knowledge (eg. regarding antibiotic indication (greater knowledge F); B > F).

Results of this study will also be presented at ECCMID 2017.

**Conclusion:** A large variety of patient-related factors impact antibiotic use. It's noteworthy that we identified many more barriers than facilitators. Further studies should try to better understand patient's views and experiences in order to facilitate responsible antibiotic use.

**References**
DRIVE-AB is supported by IMI/EU and EFPIA.
**Disclosure of Interest**
None Declared.

### O9

#### Self medicated antibiotics in rural communities in Kano, Nigeria: a cross-sectional survey of community members

Ibrahim Yusuf[1], Yusuf D. Jobbi[2], Auwalu H. Arzai[1], Muhammad Shuaib[3]
[1]Microbiology, Bayero University, Kano; [2]Haematology; [3]Microbiology, Aminu Kano Teaching Hospital Kano, Kano, Nigeria
**Correspondence:** Ibrahim Yusuf
*Antimicrobial Resistance and Infection Control* 2017, **6(Suppl 3):**O9

**Introduction:** Abuse of antibiotics through self-treatment is of public health concern, and is mainly due to easy assess to antibiotics and lack of regulatory control of their sales and prescriptions in communities.
**Objectives:** A study was conducted to evaluate the prevalence of self medication with five broad spectrum antibiotics among non health workers living in rural communities in Kano, the second most populous state in Nigeria.
**Methods:** A cross-sectional survey of 300 randomly selected adult villagers (150 males and 150 females) at ten randomly selected locations of 5 local governments in the state from August to September, 2016 was conducted with self-administered questionnaire and interview. Questions pertaining to 5 commonly self medicated antibiotics (ampiclox, amoxillin, co-trimoxazole, metronidazole and tetracycline), their usage patterns, how to purchase them and reasons for their selection were included.
**Results:** A total of 211 (70.3%) out of the 300 respondents which are between the ages of 20-40 had experienced self-medication with at least one of the antibiotics before. The most self-medicated antibiotic is ampiclox followed by tetracycline, amoxicillin, co-trimoxazole and metronidazole. Over fifty percent of the respondents (166, 55.3%) purchased substandard antibiotics which cost between $0.1-0.5 per dose from non health care workers selling drugs in their communities. While only 29 respondents (11 males and 18 females) ever completed the dosage of the self medicated antibiotics, about 22% took the antibiotics for 2 days and 41% took only 1 dose. A total of 154 (51.3%) self used tetracycline and metronidazole for treating diarrhoea, while 30.6% and 62% used ampiclox and amoxicillin to treat undiagnosed urinary tract infections and typhoid fever respectively. Only 33% self treat themselves with antibiotics previously prescribed by health care worker but majority used them as a result of family and friends recommendation. Surprisingly, only 19 out of 300 believed that self medication is a problem, but majority (168) have contrary believe and 35 have no idea.
**Conclusion:** Antibiotic self medication is on increase in rural communities, the need to develop a viable antibiotic stewardship programs in rural areas is highly stressed.
**Disclosure of Interest**
None Declared

### O10

#### The impact of antibiotic stewardship programs in Asia: a systematic review and meta-analysis

Chun Fan Lee, Benjamin J. Cowling[1], Shuo Feng[1], Hanae Aso[1], Peng Wu[1], Keiji Fukuda[1], Wing Hong Seto[1]
School of Public Health, The University of Hong Kong, Hong Kong, Hong Kong
**Correspondence:** Chun Fan Lee
*Antimicrobial Resistance and Infection Control* 2017, **6(Suppl 3):**O10

**Introduction:** The use of antimicrobial stewardship programs (ASPs) is increasing in Asia but their effectiveness in reducing the consumption of antibiotics and their impact on clinical outcomes is not known.
**Objectives:** To review published data on the consumption of antibiotics and the impact on clinical outcomes of ASPs conducted in Asia.
**Methods:** We conducted a systematic search in the Embase and Medline (PubMed) databases for studies that compared the consumption of antibiotics or clinical outcomes of patients in an Asian hospital or clinic with an ASP (intervention group) with those in a similar setting without an ASP (control group). Meta-analyses of all-cause mortality and hospital-acquired infection (HAI) were performed using random-effects models.
**Results:** The search identified 77 studies of which 22 and 19 reported on antibiotic usage and cost, respectively. Among these, 20 (91%) studies reported reduced antibiotic usage and 19 (100%) reported cost savings in the intervention group compared to the control. Duration of antibiotic therapy was reported in 7 studies; all but one reported that duration was reduced in association with an ASP. In the meta-analyses, rates of all-cause mortality and HAI were not significantly different between the intervention and control groups, but mortality rates were significantly improved by ASPs using drug monitoring, while HAI rates were also improved by ASPs that included infection control or hand hygiene programs.
**Conclusion:** ASPs reduce the consumption of antibiotics in hospital and clinic settings and are not associated with worse clinical outcomes. The findings support the broad implementation of antimicrobial stewardship interventions conducted in hospital and clinic setting in Asia.
**Disclosure of Interest**
None Declared.

### O11

#### National prevalence study of healthcare associated infections and antibiotic use in nursing homes (France 2016)

Anne Savey[1,2], Anais Machut[2], Gaetan Gavazzi[3], Yann Lestrat[4], Anne Berger-Carbonne[4] on behalf of RAISIN working group
[1]CIRI /UCBL1; [2]CClin Sud-Est, Lyon; [3]CHU, Grenoble, [4]Sante Publique France, Paris, France
**Correspondence:** Anne Savey
*Antimicrobial Resistance and Infection Control* 2017, **6(Suppl 3):**O11

**Introduction**
The first national point prevalence study (PPS) of Healthcare-associated infections (HAI) and antibiotic use (AB) was conducted in French nursing homes (NH).
**Objectives**
1) describe & measure prevalence of HAI and AB use 2) raise awareness of HCW and prescribers 3) identify & prioritize needs for intervention, training or additional resources.
**Methods**
PPS was proposed to a sample of 719 NH selected at random among 7,387 French NH. Data were collected on a single day in May-June 2016 concerning: NH organization and resources, HAI and systemic AB among residents. We focused on urinary tract inf./UTI including germs and resistance pattern, *C. difficile* inf./CDI, pneumonia/PNE, low respiratory tract inf./LRTI, influenza/FLU, skin & soft tissue inf. /SSTI, wound & pressure sores inf./WPSI, scabies/SCA, catheter-related inf./CRI. NH were provided with standardized protocol, training, software for data input/report. National data were analyzed with STATA11; results were weighed according to sampling design.
**Results**
Data concerned 367/719 NH (51%) including 28277 residents (sex-ratio 0.36; 63.4% $\geq$85 yrs). Exposure to invasive procedures was low: 3.3% catheters (mostly subcutaneous), 1.7% urinary catheters and 0.9% surgery < 30 days. National prevalence rates were **2.9%**[$CI_{95}$ 2.57-3.29] **residents with HAI** (med 2.5, range 0-21.1) or **3.0%**[2.65-3.42] **HAI**, and **2.8%**[2.46-3.07] **residents with AB** (med 2.3, range 0-21.1). Variations were analysed according to NH and resident characteristics. Among HAI, 36.9% were UTI, 24.0% IRB, 11.0% PNE, 20.4% SSTI, 5.6% WPSI, 1.3% CRI and 0.3% SCAB, 0.1% CDI. Only 68.8% of UTI were confirmed microbiologically: *E.coli, P.mirabilis* and *K.pneumoniae* were predominant; 26.3% of *Enterobacteriaceae* strains were resistant to 3[rd] gen. cephalosporins (3GC) and 13.3% produced EBSL.
Concerning AB use, oral administration route was the most frequent (85.1%) followed by subcutaneous (8,3%). A high level of prophylactic use was observed (13.7%). Most frequent AB were 20.9% 3GC, 19.0% penic. A, 16.0% amoxicillin-clavulan., 12.3% macrolides, and 11.4% fluoroquinolones.

**Conclusion**
This PPS will provide French NH with reference data and appears effective in monitoring local and national strategies for HAI prevention and AB use.
**Disclosure of Interest**
None Declared.

### Models to better understand infection control measures

#### O12
### Recommended classification of clostridium difficile infections overestimates the proportion acquired during current hospitalisation

Angus Mclure, Archie C. A. Clements, Martyn Kirk, Kathyrn Glass
Research School of Population Health, Australian National University, Canberra, Australia
**Correspondence:** Angus Mclure
*Antimicrobial Resistance and Infection Control* 2017, **6(Suppl 3):**O12

**Introduction**
The Infectious Diseases Society of America and the Society for Healthcare Epidemiology of America recommend that *Clostridium difficile* infections (CDIs) be classified as healthcare facility associated if the onset of symptoms was more than two days after hospital admission. The incubation period for *C. difficile* is often much longer than two days, which may result in significant misclassification.
**Objectives**
We used a mathematical model to assess the current guidelines for classification of the origin of CDI and identified potential improvements to the method of classification.
**Methods**
We simulated *C. difficile* transmission in a healthcare setting from patient admission through to discharge to determine the time from admission to onset of symptoms for CDIs acquired during the current hospitalisation and CDIs acquired prior to the current hospitalisation. We conducted sensitivity analyses to compare our base scenario with a range of plausible alternative scenarios.
**Results**
In our base scenario, the recommended two-day classification had good sensitivity, but poor specificity to identify CDIs acquired in the current hospitalisation, overestimating their incidence by nearly 100%. A six-day cut-off accurately estimated the incidence of CDIs acquired during the current hospitalisation and CDIs acquired prior to the current hospitalisation. In the sensitivity analysis, a two-day cut-off overestimated the incidence of CDIs acquired in the current hospitalisation by 30-350%, with the greatest error in settings with low within-hospital transmission.
**Conclusion:** The recommended two-day cut-off for healthcare-acquired CDIs systematically overestimates the proportion of infections acquired in hospital. This may make policymakers overly optimistic about the potential benefits of interventions that only address within-hospital transmission. We recommend that infection control practitioners use a 5-day or longer cut-off to assess acquisition of CDI in healthcare settings.
**Disclosure of Interest**
None Declared.

#### O13
### Infection control of VRE in hospitals: A modeling analysis of A French outbreak

Rania Assab[1], Rose Choukroun[2], Didier Guillemot[3], Laura Temime[1], Florence Espinasse[4], Bahrami Stéphane[5], Lulla Opatowski[2]
[1]Conservatoire National des Arts et Métiers/ Institut Pasteur; [2]Institut Pasteur,Univ. Versailles St Quentin, Université Paris-Saclay,Inserm; [3]Institut Pasteur,Univ. Versailles St Quentin, Université Paris-Saclay,Inserm, Assistance Publique Hôpitaux de Paris, Paris; [4]Assistance Publique Hôpitaux de Paris, Boulogne Billancourt, [5]Univ. Versailles St Quentin, Université Paris-Saclay, Assistance Publique Hôpitaux de Paris, Versailles, Garches, France
**Correspondence:** Rania Assab
*Antimicrobial Resistance and Infection Control* 2017, **6(Suppl 3):**O13

**Introduction**
Implementation of effective control measures against nosocomial pathogens is crucial for hospitals but can lead to important disorganization and costs. Evidence is mostly focused on *Staphylococcus aureus*, while Vancomycin-Resistant *Enterococci* (VRE) received much less attention, despite a potential high burden. Assessing VRE control strategies at the hospital level is therefore of major importance.
**Objectives**
To analyze the impact of several control strategies during a VRE outbreak in a hospital.
**Methods**
We analyzed a VRE outbreak which occurred in a French hospital over 2012-2013. Detailed individual data were collected, to gather information about control measures implementation, inter-wards patients' transfers, VRE colonization and associated costs. We developed a spatially-explicit stochastic individual-based model and used statistical inference to estimate ward-specific transmission rates and simulate the impact of control measures. Different scenarios, including various screening schedules, detection techniques (PCR and cellular culture), hygiene level and cohorting strategies were simulated and compared regarding their impact on the outbreak size and associated costs.
**Results**
The outbreak affected 22 patients in 5 different wards. It was controlled after 22 weeks, following at-risk patients cohorting, interruption of admissions in affected wards and transfer to purposely set-up wards. In total, it resulted in a ~230 k€ additional cost and ~820 k€ lost revenues. Estimated transmission rates ranged 0.19-0.49 $ind^{-1}.day^{-1}$. Assuming no control measures, model simulations predicted an outbreak of 44 colonized patients [43.7,44.9]. Increasing hygiene measures reduced the global incidence in average by a factor 6.5, and lead to VRE eradication after ~7 weeks (2,13). The best scenario included optimized cohorting procedures and screening strategies (using PCR) resulting in ~10-fold reduction of global incidence.
**Conclusion**
Mathematical models are useful tools to design optimal cost effective control strategies. Optimized cohorting of at-risk patients and screening schedule are key to control VRE outbreaks.
**Disclosure of Interest**
None Declared.

#### O14
### Integrated videography and environmental microbial sampling to model hand contamination: insights from Tanzania, Vietnam, and South Africa

Timothy R. Julian[1], Heather Bischel[2], Ana Karina Pitol[1], Tamar Kohn[3], Harada Hidenori[4]
[1]Department of Environmental Microbiology, Eawag, Dübendorf, Switzerland; [2]Civil and Environmental Engineering, University of California at Davis, Davis, California, United States; [3]School of Architecture, Civil and Environmental Engineering, EPFL, Lausanne, Switzerland; [4]Graduate School of Global Environmental Studies, University of Kyoto, Kyoto, Japan
**Correspondence:** Timothy R. Julian
*Antimicrobial Resistance and Infection Control* 2017, **6(Suppl 3):**O14

**Introduction:** Hands transport microorganisms through the environment, contributing to infectious disease transmission. To understand the relative importance of hands in transmission, we typically rely on simplistic models of hand-surface interactions. For example, a constrained sequence of events (e.g., a hand touches the surface, then the hand touches the mouth). Models rarely account for the sporadic and sequential nature of multiple individual contacts between hands and surfaces in the environment.
**Objectives:** The objective of this study is to capture, quantify, and model the impact of sporadic and sequential hand-surface contacts on microbial transmission.
**Methods:** In three countries (South Africa, Tanzania, and Vietnam), we recorded people's activities using first person videography. Aided with Video Translation Software, we converted videos into time series of contact events for each persons' hands. Microbial sampling for fecal indicator bacteria on the person's hands and in their environment was integrated with MLATS to model microbial contamination of hands over time.
**Results:** Almost 50 hours of videography data were collected from more than 35 people: In South Africa, workers were recorded while collecting and processing urine for nutrient recovery. In Tanzania, women were recorded while performing daily activities. In Vietnam, farmers were recorded while collecting and applying human excreta to agricultural fields. Translation demonstrated high frequency of hands contacted surfaces (average (standard deviation) of 270 (66) / hr in South Africa, 290 (75) / hr in Tanzania, and 326 (401) / hr in Vietnam). A subset of participants exposed themselves to microorganisms from hand to mouth contacts, on average (standard deviation) 5(3)/ hr in South Africa, 3.6 (1.1) times per hour in Tanzania, and 6(6) / hr in Vietnam.
**Conclusion:** The model provides insight on the importance of rare – but high risk – contact events on hand contamination, and demonstrates stark differences in microbial transport across different settings and activities.
**Disclosure of Interest**
None Declared.

### Device-related infections

#### O15

#### Peripherally inserted central venous catheters associated bloodstream infections: a systematic review and meta-analysis

Jiancong Wang[1], Mercedes G. Gasalla[2], Walter Zingg[1]
[1]Infection Control Program and WHO Collaborating Centre on Patient Safety, University of Geneva Hospitals and Faculty of Medicine, Geneva, Switzerland; [2]Infectious diseases dept, Hospital Son Llátzer, Palma de Mallorca, Spain
**Correspondence:** Jiancong Wang
*Antimicrobial Resistance and Infection Control* 2017, **6(Suppl 3):**O15

**Introduction:** Peripherally inserted central venous catheters (PICCs) are increasingly used for therapeutic purposes in various clinical settings. However, there is a paucity of studies systematically assessing the incidence density of PICC-associated bloodstream infections (PABSI) over time.
**Objectives:** It was the aim of this systematic review to assess the PABSI-incidence in different patient populations and clinical settings.
**Methods:** We searched PubMed, EmBASE, and the Cochrane database between 1 Jan 1987 and 31 July 2016 without language restriction. Any study reporting PABSI incidence density (PABSI per 1000 PICC-days) in an inpatient or outpatient medical setting was eligible. Only baseline results and rates from control groups were included for intervention studies. Results were stratified in adults (hematology, inpatient, and outpatient), children and neonates. Weighted PICC incidence densities were calculated using a random-effects model.
**Results:** A total of 490 publications were identified, of which 104 were eligible for final analysis. PABSI incidence densities per 1000 PICC-days in the adults, children and neonates were 0.98 (0.84-1.12), 1.82 (1.23-2.41), and 6.11 (5.24-6.97), respectively. Statistical heterogeneity among adults allowed subgroup analyses in hematology/oncology, inpatient, and outpatient where PABSI incidence densities per 1000 PICC-days were 0.58 (0.39-0.77), 1.03 (0.86-1.20), and 0.94 (0.42-1.47), respectively.
**Conclusion:** This is the first systematic review addressing PABSI incidence densities in various patient populations and clinical settings. PABSI incidence density among neonates was significantly higher compared other groups. There was no significant difference between using PICC lines in inpatient and outpatient settings. Although the incidence density in adults is similar to numbers of non-tunneled central-venous lines, there is trend towards lower PABSI incidence densities over time.
**Disclosure of Interest**
None Declared.

#### O16

#### Iintervention strategy consisting of education, bundle checklist, and feedback was insufficient to reduce central line-associated blood stream infection (CLABSI) rates in Korea: 2-year experience in 26 hospitals

Pyoeng Gyun Choe[1], Myoung Jin Shin[2], Kyung Hee Lee[3], Eun Jin Kwon[4], Moon Hee Hong[5], In Young Jeon[6], Kyoung-Ho Song[1], Eu Suk Kim[1, 2], Hee-Chang Jang[7], Sun Hee Lee[8], Hong Bin Kim[1] on behalf of Korean Infectious Disease (KIND) Study Group
[1]Department of Internal Medicine, Seoul National University College of Medicine, Seoul; [2]Infection Control Team, Seoul National University Bundang Hospital, Seongnam; [3]Center of Infection Control, Dongsan Medical Center Keimyung University, Daegu; [4]Infection Control Service, Chonbuk National University Hosptial, Jeonju; [5]Infection Control Team, Yeungnam University Medical Center, Daegu; [6]Infection Control Department, Chungnam National University Hospital, Daejeon; [7]Department of Infectious Diseases, Chonnam National University Medical School, Gwangju; [8]Department of Internal medicine, Pusan National University Hospital, Pusan, Korea, Republic Of
**Correspondence:** Pyoeng Gyun Choe
*Antimicrobial Resistance and Infection Control* 2017, **6(Suppl 3):**O16

**Introduction:** There are a number of evidences based guidelines for CLABSI prevention, however implementation of these guidelines in real clinical practice is a challenge.
**Objectives:** To evaluate the impact of comprehensive approach to prevent CLABSI in adult ICUs participating in Korean National healthcare-associated Infection Surveillance System (KONIS).
**Methods:** This study was prospective multicenter quasi-experimental study, which was divided in two phases: pre-intervention observation period (Oct 2014 to Feb 2015) and intervention period (Mar 2015 to Dec 2016). In pre-intervention period, we performed surveillance for CLABSI rates and monitoring of compliances with practice guidelines. In intervention period, we had continued surveillance and performed three interventions; (1) education program for ICU staffs, (2) application of insertion checklist, (3) monthly feedback to ICU staffs on CLABSI rates and compliance with practice guidelines.
**Results:** Of 166 ICUs in 94 hospitals participating in KONIS, 58 ICUs of 26 hospitals were enrolled in this study on a voluntary basis. During the study period, 340,792 catheter-days were monitored and 742 CLABSI were occurred. After implementation of intervention, compliance with practice guidelines significantly improved; hand hygiene before insertion (93% to 95%, $P = 0.010$), use of sterile full body drape (81% to 88%, $P < 0.001$), skin preparation with > 0.5% chlorhexidine tincture (83% to 91%, $P < 0.001$). The pooled mean CLABSI rate was 2.1 per 1,000 catheter-days in pre-intervention period and 2.2 per 1,000 catheter-days during intervention (95% confidence interval, 1.8-2.5 versus 2.0-2.4, $P = 0.593$).
**Conclusion:** Comprehensive implementation strategy using education program, bundle checklist, and feedback was feasible in real clinical practice of Korean ICUs and improved performance standards, but had no further effect on CLABSI rates. To achieve zero tolerance, more aggressive intervention targeting maintenance practice is needed.
**Disclosure of Interest**
None Declared.

#### O17

#### Nurse-driven protocol for urinary catheter removal: 3 questions for easy assessment

Bispo S. Ana, Carlos Palos
Infection Control Comittee, Hospital Beatriz Ângelo, Loures, Portugal
**Correspondence:** Bispo S. Ana
*Antimicrobial Resistance and Infection Control* 2017, **6(Suppl 3):**O17

**Introduction:** Hospital Beatriz Ângelo(HBA) is a JCI-accredited, 425-bed general hospital. Urinary catheterization(UC), a major drive for Catheter-Associated Urinary Tract Infection(CAUTI), is influenced by nurse's and physician's awareness and perceptions, not only by indications. Decreasing inappropriate UC is a challenge for infection control and antibiotics committees(ICAC).

**Objectives:** Reducing inappropriate urinary catheterization with a nurse-driven protocol for urinary catheter removal

**Methods:** In order to reduce inappropriate UC, a nurse-driven protocol(NDP) for urinary catheter removal was implemented, based on *OnTheCusp:STOP HAI*(APIC, 2012). Adaptations were made in the question whether or not the catheter should be in place as well in the *Post Discontinuation Observation Algorithm* (no nurse ultrasound). Questions(Q) for NDP were as follows:Q1-Is the catheter in place for at least one of acceptable reasons(pick-list from evidence-based guidelines)?;Q2-Impossibility of urinary condom (male gender)?;Q3-Is this a patient who underwent urologic, gynecologic or general surgery procedure involving urinary tract/had difficult catheterization/had previous false passage? NDP assessment was done in the beginning of every shift. With No answer to all 3Q, nurses proceeded to UC removal. Implementation started successfully on 2014, in 1 surgical wards and 2 medical wards with significantly higher internal rates of CAUTI. In 2016, NDP was generalized to the entire hospital wards(excluding ICU, NICU and OR) and impact of such intervention in terms of device utilization ratio(DUR), mean number of catheterization days per patient (MNCD) and CAUTI incidence rate was evaluated.

**Results:** Comparing to 2015, in 2016 DUR decreased 21%(16.54% vs. 13.07%;statistically significant (ANOVA, $f = 0.79$,$alpha = 0.05$)), even if inpatient days increased by 1.42%. MNCD per patient decreased from 5,10 to 4.76.CAUTI increased from 2.93 to 3‰device-days(+2,4%).

**Conclusion:** Introduction of a NDP for UC removal was successful in decreasing both DUR and MNCD per patient. However, it didn't decreased CAUTI rate, what can be explained by an increased patient severity index, a low CAUTI starting value (2,93‰device-days) as well a worsening problem with Carbapenem-resistant *Enterobacteriacea*. Implementation of a nurse-driven protocol for urinary catheter removal bypasses lack of awareness and knowledge of nurses and physicians.

**Disclosure of Interest**

None Declared.

### O18

#### Incidental unmasking of lapses in bronchoscope re-processing by tuberculosis PCR-in a tertiary care hospital in Kolkata, India

Debkishore Gupta[1], Ajoy K. Sarkar[2]
[1]Calcutta Medical Research Institute; [2]Peerless Hospital, Kolkata, India
**Correspondence:** Debkishore Gupta
*Antimicrobial Resistance and Infection Control* 2017, **6(Suppl 3):**O18

**Introduction:** Very low concentration of Rifampicin resistant *Mycobacterium tuberculosis* was detected by nested Tuberculosis (TB) PCR (GeneXpert MTB/RIF) from bronchoalveolar lavage (BAL) samples from seven patients where there were no clinical suspicions (sent as per department protocol) except the first case who was a known drug-resistant TB patient. A single bronchoscope was involved. This molecular diagnosis was also supported by rapid TB culture (BACTEC MGIT) upon extended incubation beyond 12 weeks.

**Objectives:** To identify the source of infection, gaps in standard operating procedures (SOP), especially in the re-processing of bronchoscope.

**Methods:** A retrospective cohort study was conducted. Medical records of all the patients who underwent bronchoscopy were thoroughly checked. Also, all the steps recommended by the manufacturers for re-processing of semi-critical devices such as bronchoscopes were evaluated by infection control team. Finally, re-processing was done exactly as per the SOP and under the supervision of infection control team. Three wash samples were sent for GeneXpert MTB/RIF and rapid TB culture after the completion of re-processing.

**Results:** Only the first case among the cluster had a history of Rifampicin resistant tuberculosis and the patient was on antitubercular drugs. Other patients did not have any past or present clinical evidence suggestive of active or latent tuberculosis. Following gaps were found in the steps of disinfection process:

I) During leakage testing it was observed only for few seconds, not 30 seconds as recommended by the manufacturer.
II) Before high level disinfection by orthophthalaldehyde, internal lumens were not washed with alternate suction using water and air.
III) Before storage, the channel interiors were not dried by alcohol purging as per recommendation.

Post re-processing as per protocol, all three samples came negative.

**Conclusion:** Lapses in re-processing of bronchoscopes can be accurately identified with the help of GeneXpert MTB/RIF and corrective action can be taken in quick time. This study emphasizes the need for establishing a bronchoscope surveillance protocol especially in high TB burden countries to curb the risk of spread by bronchoscopes.

**Disclosure of Interest**

None Declared.

### O19

#### Impact of monitoring measures to prevent ventilator-associated pneumonia (VAP)

Mayra G. Menegueti, Ana E. R. Lopes, Maria Auxiliadora-Martins, Thamiris R. D. Araújo, Marcelo L. Puga, Anibal Basile-Filho, Ana M. Laus, Fernando Bellissimo-Rodrigues
Ribeirão Preto Medical School, University of São Paulo, Ribeirão Preto, Brazil
**Correspondence:** Mayra G. Menegueti
*Antimicrobial Resistance and Infection Control* 2017, **6(Suppl 3):**O19

**Introduction:** Pneumonia is one of the most common and deadly nosocomial infections among critical patients. Several preventive measures have been pointed as effective by the literature, but scarce evidence is available about how to implement it.

**Objectives:** To investigate the impact of a check list application during clinical rounds on the compliance with preventive measures against ventilator-associated pneumonia.

**Methods:** This was a quasi-experimental study performed in a general Intensive care unit (ICU) of a tertiary-care university hospital, from 2014 to 2016. In the pre-intervention period, compliance with preventive measures was assessed weekly by the ICU medical staff. The intervention consisted of having a nurse and physician from the infection control service evaluating herself the mentioned compliance along with the intensive care team, during clinical rounds. VAP diagnosis was performed based on the Centers for Disease Control and Prevention (CDC) criteria. The intervention period was initiated from January 2015 on.

**Results:** As for the preventive measures the rates after and before intervention were, respectively: 1) semi-recumbent position: 83%/98%; 2) Prevention of thromboembolic disease as indicated: 94%/97%; 3) oral hygiene with chlorhexidine 94%/95%; 4) Prophylaxis of gastrointestinal hemorrhage as indicated: 90%/ 97%. The incidence density of VAP in the years 2014, 2015 and 2016 were respectively 5.65; 3.30 and 2.32 episodes per 1,000 ventilated patients-day. The rate of use of mechanical ventilation was 71.21%; 74.27% and 76.53%.

**Conclusion:** In conclusion, our results suggest that check list application through the nurse and physician from the infection control service along with the intensive care team during clinical rounds on improve the compliance with preventive measures against VAP.

**Disclosure of Interest**

None Declared.

### O20

#### Simplified selective digestive decontaminatiom may reduce acquired gram negative bacteremia in the ICU

Yaron P. Bar-Lavie[1], Ahlam Abu Ahmad[2]
[1]Critical Care Medicine, Rambam Medical Center, Haifa, Israel; [2]Critical Care Medicine, Technion-Israel Institute of Technology, Haifa, Israel
**Correspondence:** Yaron P. Bar-Lavie
*Antimicrobial Resistance and Infection Control* 2017, **6(Suppl 3):**O20

**Introduction:** Acquired infections are a common problem in intensive care units (ICU). Most of these infections are caused by aerobic gram negative bacteria. In recent years, we saw a rise in the development of multi drug resistant organisms (MDRO). The main reservoir of these bacteria is the digestive tract, therefore Selective Digestive Decontamination (SDD) may reduce infection rate.
**Objectives:** We examined whether Simplified SDD (SSDD) given in 2011-13 to our mechanically ventilated patients lead to a reduction of bacterial infecion in comparison to the 2008-10 patients with no SSDD.
**Methods:** A retrospective-prospective interventional cohort study of two periods: 2008-2010 : 500 ICU control patients, 2011-2013: 427 SSDD protocol patients (enteral Polymyxin E and Neomycin four times daily).Data for 927 patients were included: demographic and clinical characteristics, ICU length of stay, hospital length of stay, blood and lower respiratory tract cultures, ventilator associated pneumonia (VAP), antibiotic use and development of MDRO. SPSS (Version 21) was used for the statistical analysis. A $P < 0.05$ was considered significant.
**Results:** Patients who received the SSDD protocol were older and with a higher severity of illness. Still, in those who received SSDD we found a 38.8% reduction in clinically significant bacteremia ($p < 0.0001$), a 6% decline in lower respiratory tract cultures of aerobic gram negative bacteria ($p = 0.36$), a non significant 7.27% decline in respiratory tract cultures of fungi and gram positive bacteria ($p = 0.57$), a 9.65% decline of VAP ($p = 0.43$), a small 2.9% rise in the use of antibiotics ($p = 0.16$) Patients who received the Simplified SDD protocol had a significant reduction in the incidence of clinically significant bacteremia. The mechanism for this reduction may be caused by less bacterial translocation from gut lumen to blood. A small reduction was found in VAP incidence, and in gram negative lower respiratory tract cultures. There was no rise of bacterial resistance or respiratory tract fungal /gram positive cultures.
**Conclusion:** This study was done in an ICU with a high endemic rate of antibiotic resistance. SSDD may show promise in prevention of gram negative bacteremia in mechanically ventilated patients and should further be explored in prospective randomized, controlled studies.
**Disclosure of Interest**
None Declared.

### Hand hygiene

#### O21

**"Money makes money"-effects in hand hygiene promotion: earlier adopter wards benefit stronger from tailored interventions than later adopters**

Thomas von Lengerke[1], Bettina Lutze[2], Christian Krauth[3], Karin Lange[1], Jona T. Stahmeyer[3], Iris F. Chaberny[2]
[1]Medical Psychology Unit, Hannover Medical School, Hannover; [2]Institute of Hygiene/Hospital Epidemiology, Leipzig University Hospital, Leipzig; [3]Institute for Epidemiology, Social Medicine and Health Systems Research, Hannover Medical School, Hannover, Germany
**Correspondence:** Thomas von Lengerke
*Antimicrobial Resistance and Infection Control* 2017, **6(Suppl 3):**O21

**Introduction:** The cluster-randomized controlled trial PSYGIENE has shown that psychologically tailored hand hygiene interventions in intensive care units (ICUs) at Hannover Medical School (MHH) led to more sustainable compliance rebounds than the standard German Clean Care is Safer Care-campaign (Aktion Saubere Hände, ASH) [1]. However, unexplained variations in compliance persist.
**Objectives:** To test whether the PSYGIENE-interventions worked equally well on wards with a high vs. low mean pretrial compliance (earlier vs. later adopter-wards).
**Methods:** Interventions targeted 10 ICUs and 2 hematopoietic stem cell transplantation units at MHH. Tailoring was based on the Health Action Process Approach (HAPA). Determinants were assessed among employees via questionnaire (response: physicians: 71%; nurses: 63%) and stakeholders via problem-focused interviews (100%). In the "tailoring"-study arm (6 wards), 29 behaviour change techniques were implemented in training sessions and feedback discussions, while in the control arm, usual ASH-campaign sessions were conducted (all 2013). Outcomes were 2014-15 compliance rates assessed by WHO's gold-standard. Earlier adopter-wards were defined by a mean 2008-12 compliance of ≥63%.
**Results:** Among earlier adopter-wards, in 2015 those in the "tailoring"-arm had a 12% higher compliance than the ASH-arm (75% vs. 63%, $p < 0.001$). This corresponded to a differential increase from 2013-15 ("tailoring": +15%, ASH: +6%, $p = 0.003$), and similar baseline compliance in 2013 (60% vs. 57%, $p = 0.216$). Among later adopter-wards, neither the difference between study arms in 2015 ("tailoring": 61%, ASH: 65%, $p = 0.135$) nor the difference in the increase from 2013-15 (+12% vs. +11%, $p = 0.911$) were significant.
**Conclusion:** Early adopter-wards receiving tailored interventions achieved highest levels of and increases in hand hygiene compliance. This points to "Money makes money"-effects in infection prevention, and raises issues of how to reach later adopters more successfully

**References**
von Lengerke T, Lutze B, Krauth C, Lange K, Stahmeyer JT, Chaberny IF. Promoting hand hygiene compliance: PSYGIENE—a cluster-randomized controlled trial of tailored interventions. Dtsch Arztebl Int 2017;114:29-36.
**Disclosure of Interest**
None Declared.

#### O22

**Interaction design methodology as an innovative tool to enhance hand hygiene: the intersection of art and science**

Julia Kupis[1], Greg Hallihan[1], Jaime Kaufman[1], Craig Pearce [2], Crystal Salt[3], Manas Bhatnagar[1], Haig Armen[4], Jonathan Aitken[4], John Conly[5]
[1]W21C, University of Calgary and Alberta Health Services; [2]Alberta Health Services Infection Prevention and Control; [3]Alberta Health Services, Calgary; [4]Emily Carr University of Art and Design, Vancouver; [5]Medicine, University of Calgary and Alberta Health Services, Calgary, Canada
**Correspondence:** John Conly
*Antimicrobial Resistance and Infection Control* 2017, **6(Suppl 3):**O22

**Introduction:** Strategies for improving hand hygiene compliance (HHC) are necessary in health care settings.
**Objectives:** We sought to implement and evaluate an innovative interaction design (ID) methodology developed by the Emily Carr Health Design Lab (ECHDL) to remotely monitor and enhance HHC.
**Methods:** Alcohol-based-rub (ABR) dispensers (n = 29) on a 36 bed medical teaching unit were modified to monitor their frequency of use (FoU) and transmit data to local servers over a personal area network. Real-time data visualization developed by ECHDL, presented FoU data to users on the unit as an approach to incentivize HHC. FoU data was collected simultaneously with in-person audits using iScrub Lite (V1.5.1 *U of Iowa*). Audited compliance and FoU data was modelled through linear regression and before-after comparisons were made using a Student's t-test. The FoU of ABR dispensers among HCW were analyzed before and after ID (visualizing FoU), with respect to peak and trough trends.
**Results:** FoU predicted a significant proportion of variance in compliance $R^2 = .37$, $\beta = 7.51$, $t(125) = 8.54$, $p < 0.001$. The mean frequency of dispenser use per hour with visualization was higher ($M = 5.10$, $SD = 3.04$) than with no visualization ($M = 3.55$, $SD = 2.00$), $t(22) = 1.72$, $p \leq 0.01$. The distinct FoU of ABR dispensers among HCW before and after ID revealed consistent peak and trough trends throughout the day. Peaks/troughs at specific hours revealed cyclical patterns. There were also stochastic events such as codes, visitors to a patient and intense care periods.
**Conclusion:** These data indicate ID may improve overall compliance of HH. HCW feedback indicated an increase in motivation for HHC. User feedback provided novel insights into additional uses for this technology, including remotely monitoring ABR dispenser fluid levels and spatial-temporal trends. HCW often volunteered feedback for alternative ways to visualize the data, revealing the potential for the co-design of ID interventions. Open sourcing the hardware and software components could offer a significant social innovation.
**Disclosure of Interest**
None Declared.

### O23
### A situation analysis of the world health organization multimodal hand hygiene strategy in African health care facilities

Shaheen Mehtar[1], Awa Ndir[2], Getchachew Belay[2], Andre Bulabula[3], Yomna Satate[3], Angela Dramowski[2] on behalf of Infection Control Africa Network (ICAN) hand hygiene working group
[1]ICAN; [2]Stellenbosch University, Tygerberg, South Africa; [3]Stellenbosch University, Tygerberg, Switzerland
**Correspondence:** Shaheen Mehtar
*Antimicrobial Resistance and Infection Control* 2017, **6(Suppl 3):**O23

**Introduction:** Health care associated (HAIs) infections are a major threat to patient safety worldwide, particularly in developing countries. HH compliance with recommendations remains low.
**Objectives:** To assess the effect of WHO strategy for improvement of HH by using the HH self-assessment framework (HHSAF) in 5 ICAN regions.
**Methods:** The HHSAF tool was disseminated to the five regions of ICAN via the regional ICAN representatives. Each representative perform a situation analysis from at least five hospitals in the region and return the information electronically to the coordinator during the 2 weeks of this pilot study. The HHSAF is divided into five components and 27 indicators which reflect the 5 Moments of Hand Hygiene. Based on the score achieved for the five components, the facility is assigned to one of four levels of hand hygiene promotion and practice: Inadequate, basic, intermediate, and advanced.
**Results:** Sixty-two facilities completed the survey, from 12 countries, South Africa, Botswana, Malawi, Sudan, Egypt, Cameroon, Ethiopia, Nigeria, Democratic Republic of Congo, Senegal, Guinea, Cote d'Ivoire. The majority from acute care, whilst others from long term care facilities. There were 66% (41/62) from the state sector and 24% (15/62) private hospitals. The average bed number was 542 (range 227-1384), total staff number ranged from 449- 4000. Infection prevention nurse were employed in 33.8% (21/62) facilities, and 17.7% (11/62) had infection prevention doctors. Only 13% (8/62) had registered for the Save Lives campaign, and only 22.5% (14/62) had participated in a national HH campaign. The highest scores were reported for training and education followed by system change; evaluation, feedback, reminders in the workplace and safety climate performed less well. The overall assessment reflecting the level of HH achieved for advanced and intermediate was 35% and for basic or inadequate was 62.4%. The section on the leadership was only completed by 10 respondents.
**Conclusion:** This ICAN initiative identified that HH activities are taking place in African facilities, however the HH improvement strategy requires further consolidation in terms of leadership and commitment.
**Disclosure of Interest**
None Declared.

### O24
### Determinants of hand hygiene behavior in Australian emergency departments

Andrew J. Stewardson[1,2], Rhonda L. Stuart[3], Diana Egerton-Warburton[3], Caroline Marshall[4], Michelle van den Driesen[4], Sally Havers[1,2], Jenny Bradford[1,2], Thomas Chan[1], M. Lindsay Grayson[1,2]
[1]Austin Health; [2]Hand Hygiene Australia; [3]Monash Health; [4]Melbourne Health, Melbourne, Australia
**Correspondence:** Andrew J. Stewardson
*Antimicrobial Resistance and Infection Control* 2017, **6(Suppl 3):**O24

**Introduction:** Hand hygiene compliance (HHC) is lower in Australian Emergency Departments (EDs) than acute inpatient wards.
**Objectives:** To identify modifiable determinants of HHC in the ED setting.
**Methods:** We performed enhanced hand hygiene (HH) audits in EDs at five hospitals in Melbourne, Australia. Audits were performed by direct observation by trained auditors using the 'HHA-My 5 Moments' method. In addition to standard data on healthcare worker (HCW) profession, moment, glove use, and HH action, auditors recorded information about the environment, patient, HCW and care activity. To account for the impact of workload on HHC, we extracted 'ED occupancy' for start time of each audit. We built a mixed-effects logistic regression model to assess predictors of HHC, with audit session and hospital campus as random effects.
**Results:** Twenty-four auditors recorded 1,856 HH moments involving 789 patients during 98 sessions. Overall HHC was 60.1% (95% CI, 57.8–62.3). Alcohol-based handrub was available at the point-of-care for 98% (1813/1856) of moments. Only 1.5% of moments involved a 'non-cooperative' patient (28/1856). After adjusting for traditional HH predictors (profession and indication), the regression model suggested that HHC was higher in the afternoon (adjusted odds ratio [aOR], 1.73 [1.23–2.44]; reference, morning); lower during medium and high ED occupancy (medium, 0.73 [95% 0.56-0.96]; high 0.67 [0.52-0.87]) compared with low occupancy; and lower for both hospital staff visiting the ED (0.57 [0.37–0.87]) and temporary staff (0.36 [0.17–0.75]) compared with hospital ED staff. Hand hygiene compliance was 4.9% (95% CI, 1.4–12.2) among ambulance staff. Anecdotally, patient privacy curtains represented a key barrier to good HH.
**Conclusion:** Understanding the barriers and activities that influence HHC in unique clinical settings is of fundamental importance in supporting improved HH practices. These results should inform the development of improvement strategies that are focused on conditions that currently impede HHC in EDs.
**Disclosure of Interest**
None Declared.

### O25
### Characterizing hand hygiene opportunities in the emergency department

Theresa Christine Moore[1], Emily Xu[1], Liz McCreight[2], Gillian Wilde-Friel[1], Jannice So[1], Allison McGeer[1]
[1]Mount Sinai Hospital, Toronto, Canada; [2]Infection Control, Mount Sinai Hospital, Toronto, Canada
**Correspondence:** Theresa Christine Moore
*Antimicrobial Resistance and Infection Control* 2017, **6(Suppl 3):**O25

**Introduction:** Hand hygiene is critical for prevention of healthcare associated infections. Adherence measurement by direct observation is resource intensive and biased by the Hawthorne effect. Group e-monitoring systems require knowing the expected rate of hand hygiene opportunities (HHOs) per patient care hour in each ward/department. This measurement is a challenge in emergency departments (EDs) because of wide variety of care provided.
**Objectives:** We aimed to characterize the type and frequency of HHO's in the ambulatory care (AC) zones of our ED by following patients throughout their ED visit.
**Methods:** During June and July 2016, patients who triaged to AC were enrolled in our ED waiting room. A hand hygiene observer stayed with consenting patients during their ED visit, and recorded the number of HHOs as defined by Ontario's 4 Moments for Hand Hygiene (1). Patient type, age, chief complaint and Canadian Triage and Acuity Scale score (CTAS) were recorded.
**Results:** 27 of 28 patients consented to be observed; 13 patients were at risk of deterioration (CTAS score 2/3) and 14 were less/non-urgent (CTAS 4/5). Median ED visit duration was 1.3 hr (0.3-6.1). 201 HHOs occurred in 51 hours of observation; 179 in the ED and 22 in medical imaging. Moments 1 and 4 (before/after contact with patient/environment) comprised 39% and 31% of HHOs respectively; moments 2 and 3 comprised 14% and 16%. 41% of the HHOs involved nurses, 52% physicians/nurse practitioners, 6% medical imaging technologists, and 1% others. The mean HHO/pt hour was 4.3 (95% CI 3.4-5.1); the mean HHO/visit was 6.6 (95% CI 5.2-8.1). Interactions with HHOs occurred at a relatively constant rate over each ED visit, and there was a strong correlation between visit length and number of HHOs ($R2 = 0.65$, $P < .001$).
**Conclusion:** HHO rates in AC in our ED are somewhat lower than those in major care areas (2). Physicians contribute a relatively high proportion of HHOs. These data will assist in defining expected rates of HHOs for our ED to enable e-monitoring of adherence.

**References**
1. Public Health Ontario. Best Practices for Hand Hygiene. Available from: http://www.publichealthontario.ca/en/eRepository/2010-12%20BP%20Hand%20Hygiene.pdf
2. Goodliffe L et al. Infect Control Hosp Epidemiol 2014; 35:225

**Disclosure of Interest**
None Declared.

### O26

#### Alcohol-based hand rub and incidence of healthcare associated infections in a rural regional referral and teaching hospital in Uganda

Hiroki Saito[1], Kyoko Inoue[2], James Ditai[3], Benon Wanume[4], Julian Abeso[4], Andrew Weeks[5]
[1]Japan Ministry Of Health Labour And Welfare, Tokyo; [2]Institute of Tropical Medicine, Nagasaki University, Nagasaki, Japan; [3]Sanyu Africa Research Institute; [4]Mbale Regional Referral Hospital, Mbale, Uganda; [5]University of Liverpool, Liverpool, United Kingdom
**Correspondence:** Hiroki Saito
*Antimicrobial Resistance and Infection Control* 2017, **6(Suppl 3):**O26

**Introduction:** Good hand hygiene (HH) practice is crucial to reducing healthcare associated infections (HAIs). Use of alcohol-based hand rub (ABHR) is strongly recommended but it is limited in Uganda. Data on HH and HAIs is sparse in resource-limited settings.
**Objectives:** 1. To assess the baseline HH practice among health care providers (HCPs) and the impact of ABHR and training in its use
2. To determine the incidence of HAIs and the effectiveness of ABHR on the reduction of HAIs
**Methods:** HH compliance among HCPs and the incidence of HAIs were assessed at a teaching hospital in rural Uganda. Inpatients from the obstetrics/gynecology (OB/GYN), pediatric and surgical departments were enrolled on their day of admission and followed up during their hospital stay. The baseline phase of 12-weeks was followed by a 12-week intervention phase where training for HH practice was provided and ABHR was supplied. Incidence of HAIs and or Systemic Inflammatory Response Syndrome (SIRS) was measured and compared between the two phases.
**Results:** A total of 3,335 patients were enrolled into the study. HH compliance rate significantly improved from 9.2% at baseline to 56.4% during the intervention phase ($p < 0.001$). The incidence of HAIs/SIRS was not significantly changed between the two phases (incidence rate ratio (IRR) 1.07, 95% CI: 0.79–1.44). However, subgroup analyses showed significant reduction in HAIs/SIRS on the pediatric and surgical wards (IRR 0.21 (95% CI: 0.10–0.47) and IRR 0.39 (95% CI: 0.16–0.92), respectively) while a significant increase in HAIs/SIRS was found on the OB/GYN ward (IRR 2.99 (95% CI: 1.92–4.66)). Multivariate survival analysis showed a significant reduction in HAIs with ABHR use on pediatric and surgical departments (adjusted hazard ratio 0.26 (95% CI: 0.15–0.45)).
**Conclusion:** To our knowledge, this study is one of the largest studies that address HAIs in Africa. Significant improvement in HH compliance was observed by providing training and ABHR. The intervention was associated with a significant reduction in HAIs/SIRS on the pediatric and surgical wards. Further research is warranted to identify measures to further prevent HAIs in resource limited settings.
**Disclosure of Interest**
None Declared.

### O27

#### Hand hygiene with alcohol-based solutions: a note of caution for hand wiping versus hand rubbing

Jérôme Ory, Hervé Soule, Marlieke De Kraker, Walter Zingg, Didier Pittet
Infection Control Program, University of Geneva Hospitals, Geneva, Switzerland
**Correspondence:** Jérôme Ory
*Antimicrobial Resistance and Infection Control* 2017, **6(Suppl 3):**O27

**Introduction:** According to the World Health Organization (WHO) and the Centers for Disease Control and Prevention guidelines, hand hygiene with alcohol-based handrub (ABHR) is the gold standard to prevent cross-transmission of microorganisms and subsequent infections in the hospital environment. Some companies have recently developed alcohol-based hand wipes (ABHW) and have brought them to the market. Although ABHW could serve as an alternative to conventional handrub with rinse and gel, no norm exists to evaluate their effectiveness for hand hygiene.
**Objectives:** The objective of this study was to compare the efficacy of hand rubbing and hand wiping to clean hands.
**Methods:** Efficacy tests were performed in accordance with the European Norm 1500. Primary outcome was non-inferiority of hand wiping vs. hand rubbing in reducing bacterial count on hands. Hand wiping was carried out with 2 different homemade wipes: cotton and polypropylene (PP) wipes. Dry PP wipes (DHW) were used as control. Hand rubbing was performed applying WHO technique. The isopropanol 60% (v/v) (3 ml) was used. A Generalized Linear Mixed Model (GLMM) with random intercepts for each participant, taking into account the repeated measures design, was used to assess the $log_{10}$ reduction for each hand hygiene technique, compared to hand rubbing.
**Results:** Twelve volunteers carried out all 4 tests (ABHR, 2 ABHW, DHW), and 8 volunteers carried out 3 tests (ABHR, 2 ABHWs). Hand rubbing reduced the bacterial count by a mean of 3.57 UFC/ml (95% CI 0.94-6.20), hand wiping with PP wipes by a mean of 2.39 UFC/ml (95% CI 0.95-3.82), hand wiping with cotton wipes by a mean of 2.35 UFC/ml (95% CI 0.76– 3.93), and wiping with dry PP wipes by a mean of 1.92 UFC/ml (95% CI 0.52- 3.33), respectively. The GLMM showed that hand wiping was inferior to hand rubbing, as the difference in log10 reduction was more than 0.6. In a secondary analysis, including only wiping techniques, none gave a significantly larger reduction, including a comparison between ABHW and DHW.
**Conclusion:** This study demonstrates that hand rubbing is more effective than hand wiping to reduce microbacterial burden on hands. The results suggest that a note of caution is justified when using wipes to disinfect hands. ABHWs should not be recommended for hand hygiene neither in hospitals nor in the community.
**Disclosure of Interest**
None Declared.

## Surveillance of healthcare-associated infections

### O28

#### Burden of healthcare-associated infections in outpatient care - a systematic review

Qiao Fu[1], Diana Neves[2], Walter Zingg[3]
[1]Infection Control Programme, West China Hospital, Sichuan University, Chengdu, China; [2]Infection Control Programme, Hospital Santa Maria Centro Hospitalar de Lisboa Norte, Lisbon, Portugal; [3]Infection Control Programme, University of Geneva Hospitals, Geneva, Switzerland
**Correspondence:** Qiao Fu
*Antimicrobial Resistance and Infection Control* 2017, **6(Suppl 3):**O28

**Introduction:** Healthcare delivery has shifted towards the outpatient setting over the past several years. There is little evidence about healthcare-associated infections (HAIs) in outpatient care.
**Objectives:** To assess the burden of HAI in outpatient care.
**Methods:** A systematic review was performed based on the PRISMA (Preferred Reporting Items for Systematic Reviews and Meta-analyses) guidelines. Studies referring directly or indirectly to HAI or transmission of pathogens in any patient undergoing medical care in an outpatient setting or at home were eligible for analysis. Medline, Embase, the Cochrance database and the outbreak database were searched for reports published between January 1996 to July 2016 without age restriction.
**Results:** Of a total of 7830 identified titles and abstracts, 126 reports fulfilled the inclusion criteria. Twenty reported on surgical site infections (SSIs) in various outpatient settings. The incidence varied between 0.1% and 8.6% with superficial SSIs being higher (1.7% > 8.6%) than other SS types. Bloodstream infection (BSI) was the most commonly reported outcome: 42 in total, with 17 in haemodialysis and 15 on home parenteral nutrition. Vascular access-related BSI ranged from 0.73 to 3.51 per 100 patient months, and 3.90-6.51 per 1000

catheter-days. Catheter-related BSI in home parenteral nutrition varied from 0.05 to 10.0 per 1000 catheter-days. Catheter-related urinary tract infection in homecare ranged from 1.2 to 4.5 per 1000 catheter-days. Hepatitis C seroconversion in haemodialysis varied from 0.0% to 29.4%. A total of 33 outbreaks were identified, of which six in the context of endoscopy and 16 in hemodialysis.
**Conclusion:** The incidence of BSI in haemodialysis is very high, followed by the incidence of catheter-related BSI due to the application of parenteral nutrition in homecare. The incidence of SSIs is comparable to inpatient settings but there is a high incidence of superficial incisional infections in private practices. Worrying numbers of hepatitis C transmissions have been identified for both incidence and outbreak reports.
**Disclosure of Interest**
None Declared.

### O29

#### Bacteremias surveillance in hospitals and clinics of three cantons of Switzerland: a fifteen year report (2001-2015)

Delphine Hequet[1], Christiane Petignat[1]
Unité HPCI, CHUV, Lausanne, Switzerland
**Correspondence:** Delphine Hequet
*Antimicrobial Resistance and Infection Control* 2017, **6(Suppl 3):**O29

**Introduction:** Bloodstream infections (BSI) are a leading cause of morbidity and mortality in hospitalized patients. Moreover a significant concern is antibiotic resistance among causative agents.
**Objectives:** We report a 15 year surveillance of community and nosocomial bacteremias in hospitals and clinics of three Cantons of Switzerland (VD, NE, JU).
**Methods:** Data were collected by infection control nurses and centralized in a database. We analyzed the data according to 3 distinct periods: 2001-2005, 2006-2010 and 2011-2015.
**Results:** From 2001-2015, we observed 11'788 BSI, 8858 community (75%) and 2930 nosocomial (25%). Community BSI proportion increased over the observation period (respectively 6.8, 8.39 and 10.11/1000 admissions). Mean age significantly increased over the years (from 64-69 years, $p < 0.001$). The proportion of urinary related BSI increased over time from 15.1-25.9% ($p < 0.001$). However, the proportion linked to a urinary catheter remained stable (from 44.1-47.4, $p = 0.69$). *E.coli* is the most common microorganism (36.3% of community and 22.4% of nosocomial BSI). Proportion of *Enterococci* in nosocomial BSI significantly increased over time (from 6.1-10.4%, $p = 0.03$). Moreover, the proportion of *S.aureus* with methicillin resistance (MRSA) significantly decreased over surveillance period from 5.7-2.9% ($p = 0.05$) in community BSI and from 11.6-7.9% ($p = 0.27$) in nosocomial BSI. Concomitantly, the proportion of *E.coli* with extended spectrum beta-lactamase (ESBL) significantly increased among community *E.coli* BSI, but not in nosocomial BSI (from 1.3-4.5% $p < 0.001$ and 4.8-7.0% $p = 0.32$, respectively).
**Conclusion:** Proportion of community BSI increased over time contributing to risk factors for developing nosocomial infections. Urinary related infections should be a priority target in order to prevent nosocomial BSI. Multidrug resistant bacteria distribution changed gradually, resulting in a significant MRSA decrease and an increase in ESBL *E,coli* in the community. This tendency, also not significant, is also true in nosocomial BSI and should be taken into account when treating the patient. Concomitantly, a special attention should be made to *Enterococci* seen the recent vancomycin-resistant *Enterococci* outbreaks in this part of Switzerland.
**Disclosure of Interest**
None Declared.

### O30

#### The national surveillance of healthcare-associated infections in the intensive care units of Taiwan, South Korea, and Japan

Cho-Han Chiang[1], Sung-Ching Pan[2], Tyan-Shin Yang[1], Keisuke Matsuda[3], Yee-Chun Chen[1, 2]
[1]College of Medicine, National Taiwan University; [2]Department of Internal Medicine, National Taiwan University Hospital, Taipei; [3]Faculty of Medicine, Osaka University, Osaka, Japan
**Correspondence:** Cho-Han Chiang
*Antimicrobial Resistance and Infection Control* 2017, **6(Suppl 3):**O30

**Introduction:** The demographics and epidemiology of healthcare-associated infections (HAIs) in Taiwan, South Korea, and Japan are captured by their respective nationwide surveillance system.
**Objectives:** This study aims to describe the temporal trends of HAIs in the intensive care units (ICUs) of Taiwan, South Korea, and Japan from 2008 to 2015, and the causative pathogens in each country.
**Methods:** Incidence density of urinary tract infection (UTI), bloodstream infection (BSI), hospital-acquired pneumonia (HAP), catheter-associated UTI (CAUTI), central line-associated BSI (CLABSI), and ventilator-associated pneumonia (VAP) were calculated. Temporal trends across the eight-year study period were determined using the Poisson regression. Critical causative pathogens for each country were also recorded.
**Results:** Overall, all the three countries revealed significant reduction of HAI during the 2008-2015 period (from 9.34 episodes to 5.03 episodes per 1,000 patient-days in Taiwan; 7.56 to 2.76 in South Korea; 4.41 to 2.74 in Japan; all $P < 0.001$). The most significant improvement was noted among UTI in South Korea, which experienced an 82% decrease in UTI incidence from 2008 to 2015 ($P < 0.001$). For causative pathogens of UTI, all three countries revealed similar pattern as *Candida albicans* and *Escherichia coli* were the predominant strains. In CLABSI, *Acinetobacter baumannii* (AB) was the leading pathogen in Taiwan, while *Staphylococcus aureus* was predominant in South Korea and coagulase-negative staphylococci in Japan. In VAP, *Pseudomonas aeruginosa* was the leading pathogen in Taiwan, while AB was predominant in South Korea and SA in Japan.
**Conclusion:** This study identified significant decrease of HAI rate across the three countries since 2008. Both similarity and unique features of causative pathogens were noted. Currently there is only comparable data in UTI, CLABSI, and VAP since some countries provided different measurement methods. We suggest establishing an HAI surveillance network in East Asia to better identify the HAI epidemiology and develop targeted infection control policy in this region.
**Disclosure of Interest**
None Declared.

### O31

#### Comparison of CDC/NHSN surveillance definitions and ECDC criteria in diagnosis of health care associated infections in Serbian ICU patients

Olivera Djuric[1,2], Ljiljana Markovic-Denic[1,2], Bojan Jovanovic[2,3], Milena Stopic[2], Vesna Bumbasirevic[2,3]
[1]Institute of Epidemiology, Faculty of Medicine, University of Belgrade, Belgrade, Serbia; [2]Faculty of Medicine, University of Belgrade, Belgrade, Serbia; [3]Centre for Anaesthesiology, Clinical Centre of Serbia, Belgrade, Serbia
**Correspondence:** Olivera Djuric
*Antimicrobial Resistance and Infection Control* 2017, **6(Suppl 3):**O31

**Introduction:** After three national point prevalence studies (PPS) conducted in Serbian acute care hospitals using American (CDC/NHSN) surveillance definitions, Serbia is about to switch to European (ECDC) criteria for the purpose of the fourth PPS. For the better comparability of the results, the impact of using different definitions on the HAI identification is needed.
**Objectives:** To compare American and European criteria for diagnosis of the most common types of HAI in Serbian intensive care unit (ICU).
**Methods:** Prospective surveillance was performed at two surgical-trauma ICUs of the Emergency department of Clinical center of Serbia during the period from November 2014 to April 2016. Pneumonia (PN), bloodstream infections (BSI), urinary tract infections (UTI) and surgical site infections (SSI) were prospectively diagnosed by experienced clinician and epidemiologist using both types of HAI definitions simultaneously. The level of agreement between two criteria (CDC/NHSN and ECDC) was assessed by Cohen's kappa statistic (k).
**Results:** Of 406 patients, 111 (27.3%) acquired at least one HAI (total of 134 according to American definitions and 151 HAIs when using European criteria). When considering all PN, agreement was k = 1.00. For microbiologically confirmed PN it was k = 0.99 (95% CI, 0.96-1.01) and for clinically defined k = 0.86 (95% CI, 0.58-1.13). Agreement for BSI was k = 0.79 (CI 95%, 0.70-0.89). When secondary BSI was excluded from the European classification, i.e. cases secondary to another infection site (30.9% of all BSI) concordance was k = 1.00 and when microbiologically confirmed catheter related BSI were reported separately as recommended by latest ECDC protocol update, i.e. those with same pathogen isolated from vascular catheter (20.0% of all BSI), concordance was k = 0.60 (CI 95%, 0.41-0.80). Agreement for UTIs and SSIs was perfect (k = 1.00).
**Conclusion:** Microbiological confirmation of PN should be stimulated and comparison of BSI should be done with emphasis on whether catheter related BSI is included.
**Disclosure of Interest**
None Declared.

### O32

#### Linking structure & process indicators with ICU-acquired infection surveillance

Anne Savey[1,2], Alain Lepape[3], Anais Machut[2], Jean-Francois Timsit[4], Jean-Christophe Lucet[4], Francois L'Heriteau[5], Martine Aupee[6], Caroline Bervas[7], Sandrine Boussat[8], Didier Lepelletier[9], Anne Berger-Carbonne[10] on behalf of REA-RAISIN network
[1]CIRI, UCBL1; [2]CClin Sud-Est; [3]HCL, Lyon; [4]APHP; [5]CClin Paris-Nord, Paris; [6]CClin Ouest, Rennes; [7]CClin Sud-Ouest, Bordeaux; [8]CClin Est, Nancy; [9]CHU, Nantes; [10]Sante publique France, Paris, France
**Correspondence:** Anne Savey
*Antimicrobial Resistance and Infection Control* 2017, **6(Suppl 3):**O32

**Introduction:** A pilot study lead by ECDC was set up to collect additional data on Structure & Process Indicators (SPI) linked with the healthcare-acquired infection (HAI) surveillance in intensive care unit (ICU).
**Objectives:** The aim was to integrate a limited number of SPI chosen for their strong link to prevention of HAI and antimicrobial resistance in ICU, to raise awareness and improve risk management.
**Methods:** An optional module SPI was proposed in 2015 to the 188 ICU participating in the French National surveillance network *REA RAISIN*, consisting of a self-assessment during 1 to 2 weeks. Five topics were evaluated, combining unit data, direct observation and chart review:

1. Hand hygiene: annual alcohol hand rub consumption (**HRC**)
2. ICU staffing: nurse to patient ratio (**NPR**) calculated for 7 days
3. Antimicrobial use: systematic antibiotic treatment review within 3 days after prescription (**ATR**)
4. Intubation: cuff pressure control (**CP**), oral decontamination (**OD**), patient position (**POS**)
5. CVC: dressing site conformity (**CD**).

**Results:** 27 ICU participated in SPI module. Distribution of SPI by unit was (median [IQR]):

- compliance with **ATR** 80.0%[70-91.4], **CP** 85.0%[16-95.7], **OD** 91.2%[80-100], **POS** 95.0%[81-100] and **CD** 90.0%[85.7-100]
- **HRC**: 118.3 L/1000 pat.-days [81.4-132.3]
- **NPR**: 0.43 [0.36-0.72].

A score was calculated for each ICU giving 1 point if compliance ≥ 80% for ATR, CP, POS (OD excluded), HRC ≥ 120 L/1000 pat-day and NPR ≥ 0.4. For the 23 ICU, median and mean score was at 3 points. Only one unit reached 5 and none had zero. There was no correlation between overall compliance and HAI rates.
**Conclusion:** Evaluation of SPI allows a follow-up of key prevention measures. This pilot study demonstrates the feasibility of such an embarked study in the surveillance. The level of conformity is high but heterogeneous (need to understand reasons for non compliance need especially for HRC, ATR and CP). Repeating this study annually with more ICU will allow a better follow-up, and determination of possible process/outcome correlations.
**Disclosure of Interest**
None Declared.

### O33

#### Structure, process and outcome quality of surgical site infection surveillance in Switzerland

Stefan P. Kuster[1], Marie-Christine Eisenring[2], Hugo Sax[1], Nicolas Troillet[2,3] on behalf of Swissnoso
[1]Division of Infectious Diseases and Hospital Epidemiology, University and University Hospital of Zurich, Zurich; [2]Service of Infectious Diseases, Central Institute, Valais Hospital, Sion; [3]Services of Infectious Diseases and Preventive Medicine, Centre Hospitalier Universitaire Vaudois and University of Lausanne, Lausanne, Switzerland
**Correspondence:** Stefan P. Kuster
*Antimicrobial Resistance and Infection Control* 2017, **6(Suppl 3):**O33

**Introduction:** Surgical site infection (SSI) surveillance has been performed in Switzerland since 2011, with open public reporting starting in 2014.
**Objectives:** We aimed to validate structure and process of surveillance data acquisition and the accuracy of outcome detection in participating hospitals.
**Methods:** We performed site visits with on-site structured interviews and review of a random sample of ten patient records (with or without infection) and five additional randomly selected patient records with infection per hospital between November 20, 2011, and October 20, 2015. Process and structure were rated in nine domains and a weighted overall score (maximum 50 points) was calculated. Sensitivity, specificity, positive and negative predictive value were calculated for the surveillance.
**Results:** One hundred and forty-seven hospitals or hospital units were visited, with a median (range) time of participation in the surveillance of 3.4 (0.8-15.8) years and a median (range) score of 35.5 (16.25-48.5) out of 50 points. Domains that contributed most to lower scores were the quality of chart review (weighted mean difference (SD) from maximum score: 3.97 (2.30) points) and the quality of data extraction from patient charts (weighted mean difference (SD) from maximum score: 3.22 (1.64) points). Public hospitals ($P < 0.001$), hospitals in the Italian speaking part of Switzerland ($P = 0.021$) and hospitals with longer participation in the surveillance ($P = 0.018$) had higher scores than others. Among 1110 randomly selected cases, there were 49 infections and 1061 without infection. Fifteen infections (1.4% of all cases; 30.6% of all infections) were incorrectly classified as non-infection (false negative) and one non-infection (0.09%) was classified as infection (false positive), accounting for a sensitivity (95% confidence interval (CI)) of the surveillance of 69.4% (54.6% – 81.7%), a specificity of 99.9% (99.5% – 100%), a positive predictive value of 97.1% (85.1% – 99.9%) and a negative predictive value of 98.6% (97.7% – 99.2%).

**Conclusion:** Despite a well-defined and thorough SSI surveillance methodology, we encountered a wide variation of surveillance quality. Almost 30% of cases with infections were missed. Quality of chart review and accuracy of data collection are the main areas requiring improvement.

**Disclosure of Interest**

None Declared

### O34

#### An electronic surgical site infection surveillance based on the electronic self-reporting and additional targeted audit

Choi Jongrim[1], Sun Young Cho[1,2], DooMi Kim[1], DooRyeon Chung[1,2]
[1]Center for Infection Prevention and Control, Samsung Medical Center; [2]Division of Infectious Diseases, Department of Internal Medicine, Samsung Medical Center,Sungkyunkwan University School of Medicine, Seoul, Korea, Republic Of
**Correspondence:** Choi Jongrim
*Antimicrobial Resistance and Infection Control* 2017, **6(Suppl 3):**O34

**Introduction:** Conventional Surgical Site Infection (SSI) surveillance methods dependent on comprehensive chart review are resource-intensive.

**Objectives:** We evaluated an electronic SSI surveillance method based on the electronic self-reporting and additional targeted audit.

**Methods:** All surgical procedures under SSI surveillancebetween Jan 2013 and Dec 2014 were included. Since 2013, we have performed SSI surveillance for 38 surgical categories through electronic self-reporting by surgeons and additional prospective audit through the review of the electronic medical records for all procedures by trained infection preventionists. In 2016, this comprehensive audit was changed to the targeted audit focusing on the cases extracted from the computerized program according to the algorithm satisfying any of the following criteria: 1) microbial cultures were requested; 2) antibiotics were ordered; 3) infectious diseases specialist consultation was requested. We verified the new surveillance method by determining the sensitivity of SSI detection and the number of cases requiring the review by the infection preventionists and total estimated time for the review compared to the comprehensive audit method.

**Results:** During the study period, 40516 surgical procedures were included in SSI surveillance. A total of 575 SSIs (1.42%), which were identified by the comprehensive audit method, included 205 superficial incisional, 69 deep incisional, and 301 organ/space SSIs. Switching to the targeted audit method decreased the number of cases requiring the review to 15229 cases (62.4% decrease) and total estimated time for the review from 2139.97 to 875.53 man-hours. The sensitivity of SSI detection was 97.9% (563 SSI events). Twelve cases of superficial incisional SSI were missing compared to the comprehensive audit method.

**Conclusion:** The electronic SSI surveillance method based on the electronic self-reporting and additional targeted audit could reduce workload compared to comprehensive audit method while maintaining high sensitivity of SSI detection.

**Disclosure of Interest**

None Declared.

## Behaviour and harm

### O35

#### Are contact precautions associated with physical adverse events? a systematic literature review and meta-analysis

Marin Schweizer[1], Rajeshwari Nair[1], Daniel Livorsi[1], Michihiko Goto[1], Erin Balkenende[1], Nasia Safdar[2], Daniel Morgan[3], Eli Perencevich[1]
[1]Internal Medicine, University of Iowa, Iowa City; [2]University of Wisconsin, Madison; [3]University of Maryland, Baltimore, United States
**Correspondence:** Marin Schweizer
*Antimicrobial Resistance and Infection Control* 2017, **6(Suppl 3):**O35

**Introduction:** Contact precautions (CP) are typically used to prevent transmission of multidrug-resistant organisms from infected/colonized patients to other hospitalized patients. CP are complex behavioral interventions that may adversely affect quality of care and lead to physical adverse events in patients.

**Objectives:** Systematically review studies on the association between CP and physical adverse events.

**Methods:** We conducted a systematic literature review using PubMed, CINAHL, EMBASE, and PsychInfo. Studies published from 2008-2016 and systematic reviews of studies published from 1970-2008 were included. Studies without a control group were excluded. A meta-analysis was performed by pooling risk ratios using random effects models with inverse variance weighting. Heterogeneity was assessed using the Cochran Q and $I^2$ statistics. A statistically significant p-value signifies heterogeneity between studies.

**Results:** Five thousand three hundred thirty-five titles were screened for inclusion. Overall, 9 studies evaluated falls, pressure ulcers, and/or thromboembolic events. None of the pooled analyses of these 3 adverse events were statistically significantly associated with CP. Among the 6 homogeneous studies (Cochran $p = 0.61$; $I^2 = 0\%$) that evaluated falls, there was no significant association between CP and falls (pooled risk ratio (pRR): 1.17; 95% confidence interval (CI): 0.71, 1.93). Among the 5 homogeneous studies (Cochran $p = 0.55$; $I^2 = 0\%$) that evaluated pressure ulcers, there was no significant association between CP and pressure ulcers (pRR = 1.22; 95% CI: 0.72, 2.08). Five studies evaluated thromboembolic events including pulmonary embolisms and venous thromboembolic events. When pooled, there was no significant association between CP and thromboembolic events (pRR = 1.65; 95% CI: 0.85, 3.21), however, these studies were heterogeneous (Cochran $p < 0.01$; $I^2 = 71\%$).

**Conclusion:** In this meta-analysis, CP were not associated with increased risk of falls, pressure ulcers or thromboembolic events. More high-quality studies should be done to evaluate these relationships and the relationship between CP and other preventable adverse events.

**Disclosure of Interest**

None Declared

### O36

#### Cost-effectiveness of psychologically tailored hand hygiene interventions: results of the psygiene-trial

Thomas von Lengerke[1], Christian Krauth[2], Jona T. Stahmeyer[2], Bettina Lutze[3], Karin Lange[1], Iris F. Chaberny[3]
[1]Medical Psychology Unit, Hannover Medical School, Hannover, Germany; [2]Institute for Epidemiology, Social Medicine and Health Systems Research, Hannover Medical School, Hannover; [3]Institute of Hygiene/Hospital Epidemiology, Leipzig University Hospital, Leipzig, Germany
**Correspondence:** Thomas von Lengerke
*Antimicrobial Resistance and Infection Control* 2017, **6(Suppl 3):**O36

**Introduction:** The cluster-randomized controlled trial PSYGIENE on the intensive care and hematopoietic stem cell transplantation units at Hannover Medical School, Germany, has shown that psychologically tailored interventions led to more sustainable hand hygiene compliance increases than the German Clean Care is Safer Care-campaign (ASH) [1]. However, so far it has remained unclear whether this tailoring approach was cost-effective.

**Objectives:** To test whether the tailored PSYGIENE-interventions have been cost-effective in terms of lower incidences of nosocomial infections (NI) and reduced health care costs.

**Methods:** The control group received ASH education. The tailoring-arm received educational sessions and feedback discussions psychologically tailored based on Health Action Process Approach using behaviour change techniques. Nosocomial multi-resistant gram-negative bacteria (MRGN), methicillin-resistant *Staphylococcus aureus* (MRSA) and vancomycin-resistant *Enterococcal* (VRE) infections were surveyed following German National Reference Center for the Surveillance of Nosocomial Infections (KISS) protocol. Intervention costs were calculated using standard health-economic micro-costing procedures.

**Results:** NI incidence rates in the tailoring-arm fell from 0.84 (2013) to 0.58 (2014) and 0.35 (2015; vs. 2013: p = 0.017), thus inversely relating to hand hygiene compliance (54%, 64%, 70%; 2015 vs. 2013: $p < 0.001$). Results in the ASH-arm differed in that incidences, like compliance, initially developed positively (0.69 to 0.58/55% to 68%), but then worsened again (0.67 and 64%). Controlling for patient days differences between the study arms, at least 10.1 more infections were prevented in the tailoring- vs. the ASH-arm when comparing 2015 to 2013, representing cost reductions of at least 105,318€. Since tailoring costs exceeded that of the ASH by 31,591€ (35,551€ - 3,960€), the tailored interventions were above break-even.

**Conclusion:** Psychologically tailored hand hygiene interventions can be cost-effective in preventing NI.

**Disclosure of Interest**

None Declared.

### Epidemiology and control of multi-resistant bacteria

#### O37

#### Attributable cost and length of stay of nosocomial multidrug resistant gram-negative bacteria cultures

Richard E. Nelson[1], Vanessa W. Stevens[1], Makoto Jones[1], Karim Khader[1], Matthew Samore[1], Marin L. Schweizer[2], Eli N. Perencevich[2]
[1]VA Salt Lake City, Salt Lake City; [2]Iowa City VA, Iowa City, United States
**Correspondence:** Richard E. Nelson
*Antimicrobial Resistance and Infection Control* 2017, **6(Suppl 3):**O37

**Introduction:** Few studies have estimated the excess inpatient cost due to nosocomial multi-drug resistant (MDR) Gram-negative bacteria cultures and those that do are often subject to time-dependent bias.

**Objectives:** Our objective was to generate estimates of the cost of these nosocomial cultures using a unique inpatient cost dataset from the US Department of Veterans Affairs (VA) that allowed us to reduce time-dependent bias.

**Methods:** Our study included data from inpatient admissions lasting longer than 48 hours between 10/1/2007 and 11/30/2010 were included. Nosocomial MDR Gram-negative bacteria, identified from microbiology reports in the VA electronic medical record, were defined as positive clinical cultures for *Acinetobacter, Pseudomonas,* or *Enterobacterieceae* from 48 hours after admission to discharge. Positive cultures were further classified as invasive if they were taken from a normally sterile site and otherwise were classified as non-invasive. Organisms were deemed MDR if they were resistant to 3 or more classes of antibiotics. VA inpatient cost data separate the costs incurred during patient stays by calendar month. We restricted our analysis to inpatients who were discharged in a calendar month after the month in which they were admitted. We then used multivariable generalized linear models to compare the inpatient costs and LOS in the 2$^{nd}$ calendar month between patients with and without a nosocomial MDR Gram-negative bacteria cultures on the 1$^{st}$ day of the 2$^{nd}$ calendar month.

**Results:** Of the 135,479 patients included in our analysis, 205 had a nosocomial MDR Gram-negative bacteria culture. The excess cost of invasive and non-invasive nosocomial MDR Gram-negative bacteria cultures was $43,675 ($p < 0.017$) and $34,031 (<0.0001), respectively. Costs were highest for *Acinetobacter* ($56,319, $p = 0.015$) followed by *Pseudomonas* ($37,067, $p < 0.0001$) and *Enterobacterieceae* ($32,691, $p < 0.0001$). Overall, the increased LOS was 18.0 ($p < 0.0001$) days.

**Conclusion:** We found that nosocomial MDR Gram-negative bacteria cultures significantly increased inpatient cost and LOS. This was true both for invasive cultures, which are likely to be true infections, and those that were non-invasive, which are possibly colonizations.

**Disclosure of Interest**

None Declared.

#### O38

#### Surveillance of bloodstream infections due to Extended-Spectrum Beta-Lactamase-Enterobacteriaceae (ESBLE), between 2012 and 2015 in France

Isabelle Arnaud[1], Odile Bajolet[1], Olivier Barraud[1], Anne Berger-Carbonne[2], Xavier Bertrand[1], Hervé Blanchard[1], Emmanuelle Caillat-Vallet[1], Laurent Cavalié[1], Mélanie Colomb-Cotinat[2], Lory Dugravot[1], Catherine Dumartin[1], Thierry Fosse[1], Sophie Glorion[1], Emmanuelle Reyreaud[1], Anne Savey[1], Loïc Simon[1], Elisabeth Sousa[1], David Trystram[1], Vincent Jarlier[1], Pascal Astagneau[1]
[1]CClin-Arlin, Paris; [2]Santé Publique France, Saint-Maurice, France
**Correspondence:** Isabelle Arnaud
*Antimicrobial Resistance and Infection Control* 2017, **6(Suppl 3):**O38

**Introduction:** In France as in other European Countries, for many years, ESBLE (especially *E. coli*, *K. pneumonia* (Kp) and *E. cloacae*) infections have been increasing for the last 10 years despite specific guidelines. Apart from the global incidence of ESBLE, special focus is now made on surveillance of severe infections such bloodstream infections (BSI).

**Objectives:** The aim of this study was to illustrate the temporal trends of the BSI of ESBLE, ESBL *E. coli*, ESBL Kp and ESBL *E. cloacae* from 2012 to 2015.

**Methods:** A cohort of 698 Health care facilities (HCF) was studied from 2012 to 2015 through the national monitoring network of multidrug resistant bacteria in hospital (BMR-RAISIN) implemented since 2002. HCF participated within a 3-months period per year on a voluntary basis. Strains were isolated from blood culture issued for diagnostic purposes (a single strain of the same species per patient). Incidences of ESBLE BSI were calculated per 1,000 patient-days (PD) from 2012 to 2015. An Univariate Poisson regression was used to estimate temporal trends.

**Results:** From 2012 to 2015, the incidence of EBLSE BSI increased from 0.045 to 0.063 per 1,000 PD (587 to 894 BSI), representing a 39% increase ($p < 0.001$).

The same upward trends were observed for ESBL *E. coli* (0.026 to 0.034, +33%, ESBL Kp (0.011 to 0.019, +76%) and ESBL *E. cloacae* (0.006 to 0.007, +13%).

92% of BSI were observed in acute care unit (ACU), of which 17% in intensive care unit (ICU), and 8% in long-term care facilities (R-LTCF). 57% of ACU and R-LTCF ESBLE BSI were due to *E. coli*, 27% to Kp and 11% to *E. cloacae* except for ICU where the proportion of *E. coli,* Kp and *E. cloacae* was 38%, 37% and 16% respectively.

**Conclusion:** In France, incidences of ESBLE BSI continue to increase despite control efforts, especially those due to *E. coli*, Kp and *E. cloacae*.

This result must encourage HCF to upgrade their preventive measures, in particular for contact precautions, excreta management and device-related BSI which are potentially preventable.

**Disclosure of Interest**

None Declared

#### O39

#### Prevalence of ST131 clone Among ESBL-Producing E. Coli responsible for Bloodstream Infection (BSI) at Geneva University Hospitals (HUG)

Patrice Francois[1], Carolina Fankhauser-Rodriguez[2], Damien Baud[1], Elodie von Dach[2], Jacques Schrenzel[3], Stephan Harbarth[2]
[1]Genomics Research Laboratory; [2]Infection Control Program, [3]Bacteriology Laboratory, HUG, Geneva, Switzerland
**Correspondence:** Carolina Fankhauser-Rodriguez
*Antimicrobial Resistance and Infection Control* 2017, **6(Suppl 3):**O39

**Introduction:** The routine BSI surveillance at HUG recorded an important increase in cases due to ESBL-producing *E. coli* in 2015.

**Objectives:** To assess the prevalence of clone ST131 among ESBL-producing *E. coli* BSI at HUG in 2015.
To identify potential cases of hospital transmission.
**Methods:** From Jan 1 to Dec 31, 2015, **75** patients presenting **84** episodes of BSI due to ESBL producing *E. coli* were analyzed for the presence of clone ST131. A PCR assay based on a unique combination of mutations harbored by the ST131 lineage of *E. coli* was developed. Consequently, all isolates were subjected to whole genome sequencing (WGS) and compared at the nucleotide level to evaluate strain relatedness. Clinical and epidemiological data were also retrieved.
**Results:** 44/75 (59%) of patients suffered BSI due to *E.coli* ESBL ST131. Age and gender were similar for non ST131 *vs* ST131 patients. Comparing non-ST131 vs ST131 cases, 61% (19/31) and 59% (30/51) of BSI episodes were healthcare-acquired; 64% (20/31) *vs* 59% (30/51) were secondary BSI (mainly UTI). *E. coli* ST131 and non-ST131 isolates showed similar resistance profiles except for ciprofloxacin and norfloxacin (higher resistance in ST131) and cefoxitin and furans (higher susceptibility in ST131). The rapid PCR assay and MLST results deduced from WGS yielded identical results for ST131 identification. 40% of all ST131 isolates presented the profile ST131-H30Rx. Pairwise comparisons showed 2 strains with 25 single nucleotide substitutions (SNPs); the rest showed >100 SNPs revealing isolates from different origins. Comparative genomics performed on ST131 from various countries revealed parallel evolution of ST131 clones, following different introduction events of a common ancestor in our area.
**Conclusion:** In 2015, the incidence of BSI involving *E. coli* ST131 at HUG was high.
WGS demonstrated the frequent presence of H30Rx virulent profiles in our area.
Nosocomial cross contamination was not a likely cause of this ST131 increase.
**Disclosure of Interest**
None Declared.

### O40

#### Factors influencing the acceptability of screening for carbapenemase producing enterobacteriaceae; mixed method study of the general publics' views

Kay Currie, Jacqui Reilly on behalf of AMR-BESH Study Group
Glasgow Caledonian University, Glasgow, United Kingdom
**Correspondence:** Kay Currie
*Antimicrobial Resistance and Infection Control* 2017, **6(Suppl 3):**O40

**Introduction:** The World Health Organisation has prioritised antimicrobial resistance as a global health threat. Carbapenemase Producing *Enterobacteriaceae* (CPE) is a growing challenge, with evidence of rapid spread within Europe. However, screening for CPE may involve a rectal swab, potentially considered invasive and embarrassing, and colonised patients are cared for in isolation to protect others. Whilst these measures are sound infection prevention precautions, the acceptability of CPE screening and its consequences are unknown.
**Objectives:** This mixed methods study investigated the publics' views on the acceptability of CPE screening and management.
**Methods:** Data collection and analysis was guided by Theoretical Domains Framework[1], which explores how psychological perspectives affect individual's decisions to act in specific situations. Data from three focus groups ($n = 14$) was analysed thematically; findings were used to inform subsequent survey design. Descriptive and inferential analysis of survey data ($n = 261$) was used to identify variables for inclusion in a linear regression analysis.
**Results:** Results demonstrate 'strong agreement' with the acceptability of CPE screening (median score 9, on a scale of 1-10); acceptability of rectal swabs (median score 9); and acceptability of being cared for in isolation (median score 8). Linear regression modelling identified acceptability of CPE screening was significantly associated with five predictor variables: knowledge of the problem of antimicrobial resistance (β -.108, $p = .012$), social influences (β .140, $p = .032$), acceptability of being isolated if colonised (β .221, $p = .000$), beliefs about the acceptability of rectal swabbing (β .147, $p = .003$), beliefs about the impact of careful explanation from a health professional (β .316, $p = .000$).
**Conclusion:** Findings indicate that interventions to enhance public acceptability of CPE screening should focus on shaping public knowledge of CPE by providing information about antimicrobial resistance and capitalising on social influences by harnessing ideas of collective action or the public good.

**Disclosure of Interest**
None Declared.

### O41

#### Multimodal strategy to reduce the incidence of carbapenem-resistant Klebsiella pneumoniae

Wanda Cornistein[1], Andrea Novau[1], Laura Paulovsky[1], Leonardo Fabbro[1], Guillermina Kremer[2], Maria Laura Pereyra[2], Javier Alvarez[3], Pablo Pratesi[3], Viviana Vilches[4]
[1]Prevention and Infectious Control; [2]Infectious Disease; [3]Intensive Care Unit; [4]Microbiology, AUSTRAL UNIVERSITY HOSPITAL, Buenos Aires, Argentina
**Correspondence:** Wanda Cornistein
*Antimicrobial Resistance and Infection Control* 2017, **6(Suppl 3):**O41

**Introduction:** In Argentina, the prevalence of carbapenem resistant *Klebsiella pneumoniae*(CRKp) reaches 10%.In our institution, there was an increase in cases of colonization and infection by CRKp in the last years. A multimodal strategy (MMS) can improve health personnel behaviour with better outcomes.
**Objectives:** Determine the impact of a MMS to control colonization and infection by CRKp
**Methods:** Prospective, interventional study, July-September 2016 in a general university acute care hospital in Buenos Aires (Argentina) with 145 beds (15 in the adults ICU). A multimodal program was implemented that included 1-structural changes of the unit; 2-on-spot training, 3-discontinuing universal contact precautions (modification of isolation policy); 4 - Continuous assessment of Health Care Associated Infection (HCAI) rates, Hand Hygiene (HH) compliance, ATB consumption and audit room disinfection protocol(RDP); 5-feedback of information; 6-reminders in the workplace; 7- periodic meetings with leaders with hands-on workshops to foster a culture of security. The CRKp infection and colonization rate were measured and the pre-intervention rate (PreI) January-June (1st semester 2016) was compared with postintervention (PosI) from July to December (2nd semester 2016).
**Results:** A decrease in the rate of colonization/infection by CRKp PreI 15.87/1000 patient days (pd) vs PosI 8.71/1000 pd was observed. (*p* 0.0406).
Patient unit reforms were performed to facilitate the surface cleaning. Personal staff were trained by Infection control nurses weekly. Regular Conferences with all sector nursing and staff leaders took place monthly. HH compliance was 77-80%, HAIs remained stable at 8.4/1000 pd, RDP compliance was 93%, and a 30% reduction in carbapenem consumption. (PreI 1450 DDD vs PosI 1003 DDD). Changes in unit isolation policies had a positive impact on the staff, increasing adherence to the strategy.
**Conclusion:** The implementation of a MMS can reduce the incidence of infection and colonization by CRKp.
**Disclosure of Interest**
None Declared

### O42

#### Impact of universal methicillin resistant staphylococcus aureus (mrsa) admission screening on mrsa bacteraemia incidence at Mater Dei Hospital, Malta

Michael Borg, Claire Farrugia, Elizabeth Scicluna
[1]Infection prevention and control department, Mater Dei Hospital, Msida, Malta
**Correspondence:** Claire Farrugia
*Antimicrobial Resistance and Infection Control* 2017, **6(Suppl 3):**O42

**Introduction:** Malta has historically reported one of the highest prevalence of MRSA bacteraemia (MRSA-B) in Europe. Most MRSA-B cases originate from Mater Dei Hospital (MDH), the country's only tertiary facility with 1000 beds. In 2009, median MRSA-B incidence was estimated at 1.85 cases/1000 bed days. Hand hygiene and intravenous catheter care initiatives, plus targeted screening in high risk wards (especially intensive care), reduced MRSA-B incidence to a plateau of 0.85/1000BD.
**Objectives:** To achieve further MRSA-B reduction in MDH.
**Methods:** In March 2014, universal admission screening for MRSA carriage was introduced. Dedicated trained carers, assigned to the IPC department, take nasal swabs from all patients admitted to adult medical, surgical and orthopaedic wards within 24 hours of admission (coverage >98%). Swabs are immediately plated on chromogenic medium at the bedside and taken to the microbiology laboratory. After 18 hours, colonial growth consistent with MRSA is checked; if present, it is simply reported as presumptive MRSA. Positive patients are treated with 2% nasal mupirocin and 4% chlorhexidine washes for five days. Isolation is based on risk stratification.
**Results:** Between March 2014 and December 2016, approximately 2400 patients were nasally screened monthly. 10.1% of admissions were reported as presumptively MRSA positive in 2014 (9.6% in 2015, 8% in 2016). Significant reduction in MRSA-B was achieved, reaching a median 0.16 cases/1000 bed days by 2016. It also coincided with a significant reduction in the proportion of methicillin resistance in *S. aureus* isolates from other clinical specimens. During the same period, no significant changes were observed in hand hygiene, antibiotic use and care of intravenous lines; if anything, compliance slightly regressed.
**Conclusion:** Universal MRSA admission screening (at an affordable cost of approximately €2.00 per screen) was vital to reduce MRSA-B rates in MDH, once the limit of effectiveness of other initiatives such as hand hygiene and line care had been reached. This directly contrasts with mainstream literature, which favours targeted MRSA screening, without – however – taking into consideration behavioural backgrounds, screening coverage and levels of carriage on admission.
**Disclosure of Interest**
None Declared.

### O43

#### Do probiotics reduce the duration of VRE carriage?

Li Jie[1], Izabela Kerner[2], Sik Yin Ong[2], Chiow Khuan Eu[2], Siew Yong Ng[3], Thean Yen Tan[1]
[1]Infection Prevention and Control; [2]Changi General Hospital, Singapore, Singapore; [3]Laboratory Medicine, Changi General Hospital, Singapore, Singapore
**Correspondence:** Li Jie
*Antimicrobial Resistance and Infection Control* 2017, **6(Suppl 3):**O43

**Introduction:** Vancomycin resistant enterococci (VRE) are a type of multi-drug resistant bacteria. VRE carriage may be prolonged, and there is currently no effective method for decolonisation.
**Objectives:** This study was done to understand better the duration of VRE carriage, identify the role of probiotics on shortening VRE carriage, and analyse patient risk profile for prolong VRE carriage.
**Methods:** Patients who screened positive for VRE were invited to enrol in our study. Patients were randomised into one of two groups: the control group received no additional dietary supplementation, while the trial group received daily supplementation with Lactobacillus GG (10 billion cells) for 16 weeks. All patients were tested fortnightly for the presence of VRE for a total duration of 16 weeks. Patients were classified as free from VRE carriage if they had 2 consecutively negative cultures till week 16. Data on patient's clinical comorbidities and antibiotic exposure history were collected.
**Results:** A total of 59 patients were enrolled in this study. 46 patients completed the study, with 2 death and 11 withdrawals. Of the 46 patients, 22 were from control group while the other 24 were from the trial group.
The length of VRE carriage varied from 14 to 112 days (median, 42 days), and VRE positivity was often intermittent.
31 (67%) patients were free from VRE carriage at the end of week 16, 17 (55%) were from the trial group and another 14 (45%) were from the control group. There was no significant difference in VRE clearance between the two groups.
The most common comorbidities in our study were renal failure and diabetes. There was no association between any of the documented co-comorbidities and prolonged VRE carriage.
**Conclusion:** The median duration of VRE carriage is about 6 weeks. Administration of probiotics has no significant impact on clearing of VRE.
**Disclosure of Interest**
None Declared.

## Surgical site infections

### O44

#### Structure and process indicators for the prevention of surgical site infections: results of a European pilot survey

Tommi Kärki, Carl Suetens on behalf of HAI-Net SSI pilot survey group
European Centre for Disease Prevention and Control, Stockholm, Sweden
**Correspondence:** Tommi Kärki
*Antimicrobial Resistance and Infection Control* 2017, **6(Suppl 3):**O44

**Introduction:** The European Centre for Disease Prevention and Control (ECDC) has been requested by the European Commission to complement the European surveillance of surgical site infections (SSIs) with structure and process indicators (SPIs) for the prevention of SSIs.
**Objectives:** In 2016, ECDC organised a pilot survey to assess the compliance and feasibility of collecting data on selected SPIs through EU surveillance of SSI.
**Methods:** Participants in the pilot survey reported SPI data for a freely chosen type of surgical procedure under EU surveillance. The SPIs included: 1) administration of perioperative antibiotic prophylaxis (PAP) within 60 minutes before incision, 2) discontinuation of PAP within 24 hours after incision, 3) no hair removal or use of clippers, 4) alcohol-based skin antisepsis in the operating room, 5) patient normothermia within an hour after the operation, and 6) glucose monitoring in the perioperative period. Participants were also asked about the feasibility of collecting SPI data. For each type of procedure and each hospital, we calculated the percentage of surgical procedures that complied with each SPI.
**Results:** SPI data were reported by 14 hospitals in eight countries and for 401 surgical procedures. The overall compliance with each SPI varied from 97% for no hair removal to 64% for glucose monitoring. Compliance with each SPI also varied by type of procedure. Most SPI data referred to cholecystectomies (n = 81), for which the median hospital compliance varied from 100% for alcohol-based skin antisepsis to 0% for glucose monitoring. Four countries reported that national or local guidelines affected compliance with SPIs in their hospitals.
**Conclusion:** The collection of SPI data was feasible, although not all countries were able to collect data for all SPIs. For each SPI, compliance varied by type of surgical procedure and by hospital. To further improve the prevention of SSI, national and local guidelines should be updated with the internationally recommended SSI prevention practices. The collection of SPI data is now recommended as part of the EU surveillance of SSI to facilitate the identification of outlier hospitals and improve SSI prevention practices at both national and local level.
**Disclosure of Interest**
None Declared.

### O45

#### Semiautomated surveillance of deep surgical site infections after cardiothoracic surgery

Meander E. Sips[1], Marc J. Bonten[1,2], Maaike S. van Mourik[2]
[1]Julius Center for Health Sciences and Primary Care; [2]Department of Medical Microbiology, University Medical Center Utrecht, Utrecht, Netherlands
**Correspondence:** Meander E. Sips
*Antimicrobial Resistance and Infection Control* 2017, **6(Suppl 3):**O45

**Introduction:** Reliable and timely detection of surgical site infections (SSIs) through surveillance is indispensable for targeted implementation and monitoring of preventive measures. Manual SSI surveillance is labour-intensive and may lack a standardised case-finding method. The increasing electronic availability of routine care data enables the development of surveillance algorithms to automatically and systematically select patients with the highest SSI probability for manual chart review.
**Objectives:** To develop an algorithm that relies on routine care data to retrospectively classify patients according to their probability of a deep SSI after cardiothoracic surgery.
**Methods:** All adults who underwent cardiothoracic surgery through median sternotomy from 2012–2014 at University Medical Center Utrecht were included. Routine surveillance was the reference standard, and consisted of chart review of all patients for whom at least one relevant microbiological culture was obtained. The outcome was any –sternal or harvest site– deep SSI occurring within 90 days after surgery. Ascertainment of SSI status was done by an infection preventionist using the national surveillance definition. Electronic data elements of potential interest were obtained from our clinical data warehouse for a period of 120 days after surgery. Bivariate analyses identified the most important SSI predictors, and subsequent algorithm development focused on optimizing the positive predictive value (PPV) while maintaining sensitivity and accounting for future variations in clinical practice.
**Results:** This study included 2590 procedures, of which 25 were complicated by a deep SSI (22 sternal SSIs, 3 harvest site SSIs). Relevant microbiological testing was performed after 512 procedures (19.8%). Hence, the PPV of culture-driven case-finding –as done at present– was 4.9%. Our algorithm based on microbiology, antibiotics, revision surgery and mortality classified 113 patients as having a high probability of SSI (100.0% sensitivity, 22.1% PPV). In terms of workload reduction, this means it suffices to manually assess 4.4% of all medical charts (versus 19.8% using microbiology-based screening).
**Conclusion:** Semiautomated surveillance of deep SSIs has the potential to substantially reduce workload of manual chart review without loss of sensitivity.
**Disclosure of Interest**
None Declared.

### O46

#### Conducting surgical site infection surveillance following caesarean section in a low resource setting

Anna Maruta[1], Shaheen Mehtar[2], Valerie Jean Robertson[3], Tinei Manase[4]
[1]BIOMEDICAL RESEARCH AND TRAINING INSTITUTE, Harare, Zimbabwe; [2]Unit of Infection Prevention and Control, Faculty of Medicine & Health Sciences Stellenbosch University, Stellenbosch, South Africa; [3]College of Health Sciences, University of Zimbabwe, Harare, Zimbabwe; [4]Department of Obstetrics and Gynaecology, College of Health Sciences, University of Zimbabwe, Harare, Zimbabwe
**Correspondence:** Anna Maruta
*Antimicrobial Resistance and Infection Control* 2017, **6(Suppl 3):**O46

**Introduction:** Surveillance of SSI is important for monitoring surgical outcomes. In Zimbabwe, some information on SSI occurrence is available from individual hospital infection control reports but the definitions and methods used are not consistent.
**Objectives:** The aim was to conduct SSI surveillance in a setting with limited diagnostic or financial support.
**Methods:** This was a before-and-after study with two rolling cohort periods conducted at two referral hospitals in Harare, Zimbabwe. Data regarding the various risk factors and demographic details was collected using a standardized questionnaire which was pretested before use. Women who consented to participate in the study were recruited following caesarean section and followed up by phone post discharge. Surgical site infection was defined following Centres for Disease Control National Healthcare Safety Network (CDC-NHSN) definitions based on the clinical presentation of the patient. Training and on-going feedback was given to ensure consistency in SSI definition. To simplify clinical diagnosis of SSI, a matrix was used to assign whether the patient developed SSI or not. Information collected was entered into an excel spreadsheet and Stata v13 was used for data analysis. Incidence rates during the pre and post intervention period were used to describe the differences and the relative risk ratios are reported.
**Results:** A high risk of SSI after caesarean section was identified in the pre intervention period: 29% (95% CI: 23.4-35.0) and 12.1%($n = 35$ CI 8.3 -15.8) in the post intervention period with most of these infections occurring after discharge. Follow-up of women through telephone contact for the 30-day post-operative period identified 89.8% of SSIs that would not have been identified using inpatient surveillance alone.
**Conclusion:** The detection of SSIs using clinical parameters is feasible in low resource limited settings with little or no microbiology support.
**Disclosure of Interest**
None Declared.

### O47

#### A systematic literature review of implementation approaches to reduce surgical site infections

Promise Ariyo[1], Bassem Zayed[2], Asad Latif[1], Claire Kilpatrick[2], Benedetta Allegranzi[2], Sean Berenholtz[1]
[1]Johns Hopkins University School of Medicine, Baltimore; [2]World Health Organisation, Geneva, Switzerland
**Correspondence:** Sean Berenholtz
*Antimicrobial Resistance and Infection Control* 2017, **6(Suppl 3):**O47

**Introduction:** Surgical site infections (SSI) are one of the most frequent healthcare-associated infections. Evidence-based clinical interventions can reduce SSI incidence and protect patients. However, there is limited data on the best approach to improve adherence to these interventions in clinical practice.
**Objectives:** To identify implementation strategies aimed at improving adherence to SSI reduction interventions.
**Methods:** We conducted a systematic literature review to identify implementation strategies aimed to improve adherence to evidence-based interventions and reduce SSI. We searched PubMed, Embase, CINHAL, the Cochrane Library, the WHO Regional databases, Afro-Lib and Africa-Wide for articles published between January 1,1990 through December 2015. We used structured forms to abstract data on implementation strategies and grouped them into the *Four Es* framework – Engage, Educate, Execute and Evaluate.
**Results:** Out of 9,824 hits from the initial search, 118 studies met the inclusion criteria and were analyzed. The majority used multifaceted strategies to improve adherence with clinical based interventions. Engagement strategies included multidisciplinary work, unit based teamwork and strong leadership involvement. Education strategies included the use of different teaching tools and modalities to summarize and introduce evidence based practices to clinicians and patients. Execution strategies simplified the guidelines into simple and routine tasks with redundancy in the system to facilitate uptake. Evaluation strategies allowed assessment of compliance with established standards and patient outcomes, allowing timely feedback to providers and providing opportunities for improvement.
**Conclusion:** We summarized successful implementation strategies into a framework that can facilitate adoption of evidence-based practices. We believe that these findings complement existing clinical guidelines and may accelerate efforts to reduce SSI.
**Disclosure of Interest**
None Declared.

### O48

#### A preliminary conceptual model on the relationship between two implementation approaches for surgical site infecion prevention

Claire Kilpatrick, Julie Storr, Bassem Zayed, Benedetta Allegranzi
World Health Organisation, Geneva, Switzerland
**Correspondence:** Claire Kilpatrick
*Antimicrobial Resistance and Infection Control* 2017, **6(Suppl 3):**O48

**Introduction:** The World Health Organisation (WHO) multimodal hand hygiene improvement strategy centres on five elements (system change, education, monitoring and feedback, communications and safety climate) that are adaptable to other infection prevention contexts. Scientific evidence and global experience show that addressing all five elements contributes to success. Another evidence-based approach is the *4Es* (engagement, education, execution, evaluation) developed by Johns Hopkins. WHO identified the opportunity for a mapping exercise of the two approaches to support the development of a new implementation strategy for surgical site infection (SSI) prevention.
**Objectives:** To outline a relationship between two implementation approaches for prevention of SSI.
**Methods:** To demonstrate a relationship, a thematic analysis of the *4Es* approach was undertaken to compare it to the WHO multimodal strategy. Two international SSI meetings in 2016 were also used to gain expert consensus on the success factors that support implementation. Finally a literature review (in press) outlining application of the *4Es* in SSI prevention was critiqued to assess the extent to which real-world implementation approaches resonated with the five elements of WHOs multimodal improvement strategy.
**Results:** A conceptual model representing the relationship between the elements of the WHO multimodal improvement strategy and the *4Es* was developed. Both approaches explicitly address education and evaluation. The analysis clarified the congruence and synergies between the remaining elements that are not readily evident.
**Conclusion:** The WHO Infection Prevention and Control (iPC) Global Unit aims to promote a consistent global approach to implementation of all IPC guidelines. It is clear from this specific SSI exercise that there is sufficient commonality between the two established approaches and acceptability that a summary of the steps required to achieve SSI prevention can be presented against the existing WHO multimodal strategy. The conceptual model aims to provide better understanding of the validity of the strategic approach, aid communication and provide an easily understood interpretation for the end user. Presented within the new WHO SSI implementation strategy document, the model will be further tested across WHO regions.
**Disclosure of Interest**
None Declared

#### O49

**A bundled intervention to decrease risk of complex staphylococcus aureus surgical site infections among patients undergoing clean operative procedures**

Hsiui-Yin Chiang[1], Marin L. Schweizer[1], Melissa Ward[1], Nicolas Noiseux[2], Jeremy Greenlee[2], Mohammad Bashir[2], Daniel Diekema[1], Ambar Haleem[1], Rajeshwari Nair[1], Loreen Herwaldt[1]
[1]Internal Medicine; [2]Surgery, University of Iowa, Iowa City, United States
**Correspondence:** Marin L. Schweizer
*Antimicrobial Resistance and Infection Control* 2017, **6(Suppl 3):**O49

**Introduction:** Our prior study found that a bundled intervention was associated with lower rates of complex *Staphylococcus aureus* (SA) surgical site infections (SSIs) after cardiac or orthopedic operations.
**Objectives:** Determine if this bundle is associated with decreased complex SA SSIs in patients undergoing craniotomies, spinal operations, cardiac operations, or hip or knee arthroplasties at an academic hospital.
**Methods:** This quasi-experimental study included adult surgical patients. Patients whose preoperative nares screens were SA positive were asked to apply mupirocin intranasally 2x daily and to bathe daily with chlorhexidine-gluconate (CHG) for 5 days before their operations. Methicillin-resistant SA (MRSA) carriers received vancomycin and cefazolin perioperative prophylaxis; all others received cefazolin. Non-carriers bathed with CHG the night before and morning of surgery. Monthly counts of complex (deep incisional or organ space) SA were analyzed using Poisson regression.
**Results:** 25 complex SA SSIs occurred after 3,486 operations during the 18-month pre-intervention period and 22 occurred after 7,629 operations during the 42-month intervention period (rate ratio [RR] = 0.39; 95% CI: 0.27, 0.55). The rates decreased significantly for craniotomies (RR = 0.23; 95% CI: 0.07, 0.73) and spinal operations (RR = 0.41; 95% CI: 0.39, 0.44). Rates decreased after arthroplasties (RR = 0.49; 95% CI: 0.12, 2.06) and cardiac operations (RR = 0.91; 95% CI: 0.22, 3.80) but the decreases did not reach the significance level. During the intervention, 53% of patients received all appropriate bundle elements and 39% received some of the appropriate bundle elements. Compared with the pre-intervention period, the complex SA SSI rates decreased among patients in the fully adherent group (RR = 0.23; 95% CI: 0.09, 0.57) and those in the partially adherent/non-adherent group (RR = 0.56; 95% CI: 0.39, 0.81).
**Conclusion:** Implementation of this evidence-based bundle was associated with a decrease in complex SA SSIs, especially among patients undergoing craniotomies and spinal surgery. Implementation research should evaluate ways to improve bundle adherence.
**Disclosure of Interest**
None Declared

## POSTER PRESENTATIONS

### Surgical site infection: Risk factors and methodology

#### P1

**Effect of participating in a surgical site infection surveillance (SSI) network on the time-trend of SSI rates: a systematic review**

Ermira Tartari[1], Mohamed Abbas[2], Benedetta Allegranzi[3], Didier Pittet[2], Stephan Harbarth[2]
[1]WHO Collaborating Centre for Patient Safety, Infection Prevention &Control, Geneva University Hospitals; [2]WHO Collaborating Centre for Patient Safety, Infection Prevention &Control, University Hospitals of Geneva; [3]Infection Prevention & Control Global Unit, Service Delivery and Safety, World Health Organisation, Genève, Switzerland
**Correspondence:** Ermira Tartari
*Antimicrobial Resistance and Infection Control* 2017, **6(Suppl 3):**P1

**Introduction:** The effect of surveillance on SSI prevention remains controversial.
**Objectives:** This systematic review aims to determine the effect of participating in a SSI surveillance network on the time-trend of SSI rates, using data stratified by year of surveillance.
**Methods:** We searched Medline, EMBASE, Cochrane Library and reference lists, websites of networks and Google® for multicentre studies published between 1980 and June 2016. Studies reporting participation in a surveillance network for ≥ 3 years and presenting annual SSI rates stratified by hospitals' year of participation in the network were included. Randomized controlled trials, those concerned with ambulatory surgery only or procedures for which there is no National Healthcare Safety Network recommendation for surveillance were excluded. Results were summarized by pooling numerator (SSIs) and denominator data (no of procedures), and by calculating annual rate ratios (RR) with 95% confidence intervals (CI), using year 1 as reference.
**Results:** Of the 1079 hits, 6 studies were included representing 4 networks from Germany, the Netherlands, Switzerland, and the US. These networks reported data on 3,085,448 surgical procedures and 115,604 SSIs, with an overall pooled cumulative SSI rate of 3.75% (CI 3.73-3.77). Pooled data showed significant decreases in the RR for SSI for year 2 (RR 0.80, CI 0.79-0.82), year 3 (RR 0.92, CI 0.90-0.94), year 4 (RR 0.98, CI 0.96-1.00), and year 5 (RR 0.95, CI 0.93-0.97).
**Conclusion:** Pooled data from the currently available literature suggest that there is a decrease of SSI rates during the first 5 years of participation in a surveillance network. However, there was limited data available for synthesis.
**Disclosure of Interest**
None Declared

### P2

#### Improvement of predictive performance of the new nhsn surgical site infection risk adjustment model for colon surgery by adding variable emergency operation to the model

Pattharapa Watcharasin, on behalf of MedPSU, Supree Vikan, Pavitchon Roong-rajatavej, Tharntip Sangsuwan, Silom Jamulitrat, on behalf of MedPSU
Community Medicine, Prince of Songkla University, Hat Yai, Thailand
**Correspondence:** Silom Jamulitrat
*Antimicrobial Resistance and Infection Control* 2017, **6(Suppl 3):**P2

**Introduction:** In 2011, the National Health Safety Network (NHSN) had introduced the novel surgical site infection (SSI) prediction model which adding variables age, mode of anesthesia, endoscopic surgery, medical affiliation, hospital bed size to the previous NNIS risk model. There are no published report of the validity of the new model
**Objectives:** To evaluate the predictive performance of the new SSI risk adjustment model for postcolectomy SSI
**Methods:** Surveillance of data for postoperative SSI were retrieved from Infection Control Unit of Songklanagarind Hospital. Which surveillance system use the former NNIS risk index for SSI risk adjustment. The data included 950 colon surgeries in 931 patients admitted to the hospital during January 2005 to September 2016. Medical records were reviewed for additional information including body mass index, diabetes mellitus, colon cancer, emergency operation, American Society of Anesthesia score, anatomic site of colectomy, emergency operation, and operated under endoscope. The predictive performance of the former NNIS risk index, new NHSN model, and our proposed model was then compared by mean of area under receiver operating curve (AUC)
**Results:** The study identified 45 operations with SSI. AUC of the former and the new model were 61.2% (95% C.I = 54.2 – 69.9) and 62.5% (C.I = 54.2 – 70.8) respectively. Multiple logistic analysis revealed significant (P-value = 0.002) association between emergency colon operation and SSI with coefficient 1.01 (95% C.I = 0.38 – 1.65). We included this variable with its coefficient to the new model and calculated AUC. The resulted AUC of our model was 68.3% (95% C.I = 59.7 – 77.0). The difference of the three models was not significantly different
**Conclusion:** Predictive performance of the new CDC NHSN model is not better than the former one and can be improved by including variable emergency operation to the model
**Disclosure of Interest**
None Declared.

### P3

#### Performance of the newly introduced nhsn logistic risk model for classification postcholecystectomy surgical site infection

Tharntip Sangsuwan, Jirawat Prueksasri, Jirayu Sae-Chan, Kwanruedee Chotpitchayanukul, Silom Jamulitrat
Community Medicine, Prince of Songkla University, Hat Yai, Thailand
**Correspondence:** Tharntip Sangsuwan
*Antimicrobial Resistance and Infection Control* 2017, **6(Suppl 3):**P3

**Introduction:** NHSN has replaced the NNIS risk index model with the new logistic model. There is no report comparing the predictive performance between the two models
**Objectives:** To compare the predictive performance of the new CDC NHSN logistic risk model and the former NNIS risk index for postcholecystectomy surgical site infection (SSI) classification
**Methods:** Surveillance of data for postcolecystectomy SSIs were retrieved from Infection Control Unit of Songklanagarind Hospital. Which surveillance system use the former NNIS risk index for SSI risk adjustment. The study included 2422 patients underwent gall bladder surgery in the hospital from January 2005 to September 2016. Medical records were reviewed for additional information including emergency operation, and gastrointestinal cancer. The predictive performance of the former NNIS risk index, new NHSN model was then compared by mean of area under receiver operating curve (AUC)
**Results:** The study identified 71 operations with postcolecystectomy SSI while the former and new model predicted 21.6 and 14.0 SSIs respectively. AUC of the new model (65.7%; 95% C.I = 59.1 - 72.3) was not significantly (*P*-value = 0.5) different from the former one (64.5%; 95% C.I = 58.0 - 71.0)
**Conclusion:** The predictive performance of new CDC NHSN and the former NNIS risk index model were equivalently fairly performed for classification risk of postoperative cholecystectomy SSI
**Disclosure of Interest**
None Declared.

### P5

#### Predictors of surgical site infection in liver transplant recipients: historical cohort

Ramon A. Oliveira, Vanessa B. Poveda
Adult Health Nursing Graduate Program, Nursing School of University of Sao Paulo, Sao Paulo, Brazil
**Correspondence:** Ramon A. Oliveira
*Antimicrobial Resistance and Infection Control* 2017, **6(Suppl 3):**P5

**Introduction:** One of the major complications in liver transplant recipients (LT) is the surgical site infection (SSI).
**Objectives:** We aimed to analyse the incidence rate and the SSI predictors in LT recipients.
**Methods:** We performed an historical cohort study in a philanthropic hospital in a Brazilian city. An ethics committee has approved the research before the data collection. Inclusion criteria were: recipients older than 18 years old that did not undergo other surgical procedures 30 days before the LT, and allografts provenient from deceased donors. LT recipients that did not survive the first 72 hours, and the ones who underwent liver retransplantation within 30 days after the LT were excluded. The population was compounded by 156 LT recipients. We adopted the Centers for Disease Control and Prevention SSI diagnostic criteria. Clinical and surgical data were collected from the operation day until patient discharge or 30 days after the LT. Data was analysed by central tendency and variability measures, Pearson $X^2$ test, Fisher exact test, Mann Whitney test and Wilcoxon-Man Whitney test. After the bivariate analyses, and the variables were included in the Classification and Regression Tree model.
**Results:** The SSI incidence rate was 26.9%. The main microorganisms isolated from surgical wound were: meticilline-resistant Staphylococcus sp.; vancomycin-susceptible Klebsiella sp.; carbapenem-resistant P. aeruginosa; carbapenem-resistant A. baumanii; vacomycin-susceptible E. faecalis. We compared the bio data from the groups with and without SSI. Respectively, mean age were 54.9 years old (SD ± 9.7 years old) and 54.6 years old (SD ± 10.6 years old), 35 (83.3%) and 88 (77.2%) patients were male, body mass index (BMI) mean were 28.2 kg/m$^2$ (SD ± 5.3 kg/m$^2$) and 26.9 kg/m$^2$ (SD ± 4.4 kg/m$^2$). Prolonged operative duration (≥487 minutes) associated with BMI differences between donor/recipient (≥1.3 kg/m$^2$) increased the chance of SSI in approximately 5.5 times (OR 5,5; CI95% 2,5-1,8). In the first 96 postoperative hours capillary glycaemia (≥175 mg/dl) increased the chance of SSI in approximately three times (OR 2.97; CI95% 1.43–6.17).
**Conclusion:** There is a high incidence of SSI among the studied population suggesting that beyond the classic risk factors indicated by the literature distinct ones for this patients' category must be considered.
**Disclosure of Interest**
None Declared

### P6

#### The value of calling-back patients to detect surgical site infections (SSIS) following orthopedic and neurological surgeries in a tertiary care center in Lebanon

Nada K. Zahreddine[1], Joseph Tannous [2], Rihab Ahmadieh [1], Tala Kardas [1], Zeina Kanafani[3], Souha Kanj Sharara[3]
[1]Infection Control and Prevention Program; [2]American University of Beirut Medical Center, Beirut, Lebanon; [3]Department of Internal Medicine, American University of Beirut Medical Center, Beirut, Lebanon
**Correspondence:** Nada K. Zahreddine
*Antimicrobial Resistance and Infection Control* 2017, **6(Suppl 3):**P6

**Introduction:** SSIs are a major source of morbidity and mortality among patients undergoing surgeries. SSIs may be detected during hospitalization following surgery, upon readmission, through Emergency Department or clinic visits.
**Objectives:** To assess the role of telephone calls in detecting post discharge SSIs in the orthopedic and neurological specialties, in patients who might seek medical care in different centers. To evaluate the real increase in the SSI rates for particular surgeons in the same specialties.
**Methods:** An active, patient-based, prospective surveillance for SSI following orthopedic and neurological procedures was conducted between July and September 2016 at the American University of Beirut Medical Center (AUBMC). Surveillance was based on the CDC/NHSN (Centers Disease control and Prevention/National Health Care Safety Network) definition of SSI. Calling-back patients and assessing post discharge signs and symptoms of SSIs at 30 or 90 days was conducted using a standardized checklist. Rates were analyzed and benchmarked with NHSN and the International *Nosocomial* Infection Control Consortium (INICC) rates.
**Results:** No SSIs were identified through the phone calls among the 178 patients who were assessed throughout the surveillance period. Whereas, 2 SSIs were identified through the routine surveillance of hospital re-admissions and one SSI was identified from the review of the outpatient clinic records. SSI rates remained unchanged compared to the adopted surveillance methodology and were 3.7% following neurological surgeries and Zero following orthopedic surgeries at the time of the active surveillance.
**Conclusion:** Call-back programs may be beneficial to obtain additional post-discharge surveillance information. However, patients may have a difficult time assessing their status and the possibility of developing an SSI. Moreover, this process was found to be time consuming, and was not successful in identifying additional SSIs. Re-assessment of this method is essential to examine the role of calling-back patients in detecting SSIs.
**Disclosure of Interest**
None Declared.

### P7
### Risk factors for developing surgical site infection after pediatric cardiac surgery
Seila I. Do Prado, Mayra G. Menegueti, Ana E. R. Lopes, Fabiana M. R. Molina, Gilberto G. Gaspar, Fábio Carmona, Ana Paula C. Carlotti, Fernando Bellissimo-Rodrigues
Ribeirão Preto Medical School, Ribeirão Preto, Brazil
**Correspondence:** Seila I. Do Prado
*Antimicrobial Resistance and Infection Control* 2017, **6(Suppl 3):**P7

**Introduction:** Surgical Site Infection (SSI) is a potentially life-threating complication for patients undergoing cardiac surgery. Most of the risk factors for SSI were identified through studies based on adult populations, being scarce the evidence gathered from pediatric populations.
**Objectives:** To identify risk factors for developing SSI among pediatric patients undergoing cardiac surgery.
**Methods:** This was a prospective cohort study performed in a tertiary-care university hospital from January to December 2016. All children under 16 years old submitted to any cardiac surgery during the study period were included, and SSI diagnosis was made based on the Centers for Disease Control and Prevention (CDC) criteria, including post-discharge surveillance. Selected clinical and demographic characteristics were evaluated on the patient's medical records as potential risk factors for SSI. We used Two-tailed Fisher's exact and Mann-Whitney tests for statistical analysis.
**Results:** Among 85 patients operated in the study period, 16 (18.8%) developed a SSI, being 7 (43.8%) of them mediastinitis. The following variables were associated with those who experienced SSI versus those who did not, respectively: median age (49 vs. 518 days, $p < 0.001$), length of stay before surgery (5 vs. 1 day, p = 0.001); surgical duration (339 vs. 228 minutes, $p = 0.001$), time of extracorporeal circulation (137 vs. 90 minutes, $p = 0.003$); antimicrobial prophylaxis with vancomycin (56.2% vs. 21.7%, $p = 0.011$), Risk Adjustment for Congenital Heart Surgery I (RACHS-I) escore ≥ 4 (37.5%. vs. 4.3%, $p < 0.001$), and delayed sternal closure (37.5% vs. 8.7%, $p = 0.008$).
**Conclusion:** In the present study**,** several risk factors for SSI after pediatric cardiac surgery have been identified, such as: age, length of stay before surgery, surgical duration, time of extracorporeal circulation, use of vancomycin for antimicrobial prophylaxis, delayed sternal closure and score RACHS-I ≥ 4.
**Disclosure of Interest**
None Declared.

### P8
### Micro-dtteect system on the identification of pathogens responsible for surgical site infections (SSI) in ortophaedics
Antonio Piscitelli, Paola Navone
Orthopaedic Institute ASST Gaetano Pini/CTO, Milan, Italy
**Correspondence:** Paola Navone
*Antimicrobial Resistance and Infection Control* 2017, **6(Suppl 3):**P8

**Introduction:**
Surgical site infections (SSI) after arthroplasty surgeries are severe complications for the patient and are associated with significant additional costs resting on the Health Care System (Wijeratna MD, 2015). On prosthetic and fixation devices there might be a formation of biofilm, a collection of irreversibly adhering bacterial cells, which prolongs the survival of microorganism and hinders their identification through intraoperative swabs and other traditional isolation methods. The Micro-DTT system contains a dithiothreitol solution capable of dissolving the biofilm.
**Objectives:**
The aim of the study, conducted at the Orthopaedic Institute ASST Pini/CTO, is to compare its isolation sensitivity with the traditional methods, and correlate the PCR and IL-6 values in the serum of patients with the outcome data.
**Methods:**
The study population, recruited through prospective inclusion, consists of all patients with infected prosthesis or fixation devices or diagnosed with osteomyelitis who underwent surgery at the COR (Restorative Orthopaedic Surgery) Department.
The following are collected for each operation:

- intraoperative swabs for aerobes and anaerobes
- samples of prosthesis and/or tissue to be analysed through the Micro-DTT system
- serum for dosing the IL-6 and PCR levels, prior to the operation, and 2 and 7 days after it.

Data relating to the admission, the patient, the operation, the post-operative care and the antibiotic prophylaxis are collected.
**Results:**
To date, 107 patients, 64 males and 43 females, have been enrolled from November 2015 to December 2016. 80.3% of the operations were carried out for non-traumatic injuries. The average age of patients is 55 years.
**Conclusion:**
Preliminary results indicate that the micro-DTT systems enable the identification of a larger number of microbes compared to swabs, along with a reduction in costs, processing times and the risk of contamination of samples.

**References**
Calori G.M*., Navone P.*, Colombo M.*, Toscano M.°, Nobile M.*, Piscitelli A**., Drago L.°, Mazzola S.*
** Orthopaedic Institute, ASST Pini/CTO, Milan, Italy*
***Specialization School, Hygiene and Preventive Medicine, University of Milan, Italy*
*° IRCCS Galeazzi Institute, University of Milan, Italy*
**Disclosure of Interest**
None Declared.

### P9

#### Assessment of the risk factors of surgical site infection in a Tunisian University Hospital

Héla Ghali, Sihem Ben Fredj, Salwa Khefacha, Mohamed Ben Rejeb, Chatha Chahed, Houyem Said Latiri
Department of Prevention and Security of Care, Sahloul-Sousse, Tunisia
**Correspondence:** Héla Ghali
*Antimicrobial Resistance and Infection Control* 2017, **6(Suppl 3):**P9

**Introduction:** Surgical site infection (SSI) is still the most common problem in surgical patients.It is associated with increased morbidity and mortality, length of hospital stay, and healthcare costs and is recognized as an important indicator of the quality of care. Since around one-third of SSI is reported to be preventable, a risk factor evaluation is needed.
**Objectives:** Our study aimed to determine independent risk factors of SSI in a cohort of general surgical procedures.
**Methods:** A prospective observational study was carried out from January 2015 to May 2015 in General Surgical department of university hospital Sahloul, Sousse, Tunisia. All patients who underwent general surgical procedures and matching the inclusion criteria were included. Data were collected using a form built on the French national protocol for the SSI surveillance of *ISO-RAISAN*. The diagnosis of SSI was established according to *CDC* criteria. The studied variables were the possible risk factors related to the patient, demographic characteristics and the surgical procedure.
**Results:** 365 patients were evaluated with an overall incidence of SSI of 8.6%. Univariate analysis demonstrated the significance of an American Society of Anesthesiologists (*ASA*) score between three and six ($p < 10^{-4}$), prolonged preoperative hospital stay ($p < 10^{-3}$), contaminated and dirty wound class ($p = 0.002$), carcinological Surgery ($p = 0.04$), conventional surgery ($p = 0.009$), surgical drain ($p = 0.003$), prolonged operative duration over than 75$^{th}$ percentile ($p < 10^{-4}$), *NNIS* risk Index ($p < 10^{-4}$) on the incidence of SSI. *NNIS* risk Index and prolonged operative duration over than 75$^{th}$ percentile were found to be independent risk factors with odds ratios of 46.07 (95%, CI: 14.64 to 145) and 1.2 (95%, CI: 1.06 to 1.37), respectively.
**Conclusion:** The high SSI rate reported in our study suggests the need to implement preventive infection programs. Therefore, a combination of approaches should be taken in order to bring down incidence of SSIs such as clear guidelines for environmental hygiene, sterilization procedures, and management of sterile operative clothing. Given SSI costs, clinical morbidity and mortality, this should be a priority for our health care system.
**Disclosure of Interest**
None Declared.

### P10

#### Postdischarge surveillance: an integrative review

Viviane Harumi A. Binotto, Gabrielle M. G. B. D. S. Guatura, Vanessa D. B. Poveda
Medical and surgical nursing department, University of São Paulo, São Paulo, Brazil
**Correspondence:** Vanessa D. B. Poveda
*Antimicrobial Resistance and Infection Control* 2017, **6(Suppl 3):**P10

**Introduction:** Due to the short period of surgical hospitalization currently practiced, it is recognized that some cases of surgical site infetion (SSI) is manifested after hospital discharge.
**Objectives:** To analyze the available evidence in the scientific literature about the capacity to detect cases of surgical site infection (SSI), after hospital discharge through telephone surveillance.
**Methods:** This is an integrative review of the literature. The databases consulted were CINAHL; PUBMED; LILACS; EMBASE and Scopus. The keywords were post-discharge surveillance, telephone follow-up, telephone surveillance, postdischarge questionnaire, mhealth, telehealth, telemedicine, surgical wound infection, surgical site infection and surgical infection. We included complete articles about post-discharge surveillance by telephone, analyzing patients over 17 years old, published in English, Spanish and Portuguese, between 2000 and 2017. Studies involving animals and post-discharge surveillance methods for other types of infectious topographies or non-hospital specialties were excluded; and research designs such as case reports, case studies, qualitative approaches and narrative reviews.
**Results:** One thousand eight hundred fifty-two articles were found, of which 12 met the inclusion criteria. Of these, the majority approached several specialties in the same research (50%), followed by the specialty of gynecology and obstetrics (41.6%) and orthopedics (33.3%). Most adopted design were the observational (91.6%), with emphasis on prospective cohort studies. The number of patients analyzed in these studies ranged from 109 to 4,665. The criteria adopted during postdischarge surveillance followed those recommended by international guidelines. Only 25% studies described the sensitivity of the tested method, with results of 100%, 73.3% and 69.6%. The specificity was described in 16.6% studies, with conflicting values, range between 100% and 7.4%. The majority (66.6%) detected, through the postdischarge surveillance telephone method, between 2% and 10% cases of infection.
**Conclusion:** It is concluded that the telephone method has been used with good percentage detection capacity and sensitivity for SSI cases.
**Disclosure of Interest**
None Declared

### P11

#### Is the surgical site infections monitoring after caesarean sections possible without post-discharge surveillance? Results from multicentre study in Polish hospitals

Anna Różańska[1], Jadwiga Wójkowska-Mach[1], Andrzej Jarynowski[2], Małgorzata Bulanda[1], Katarzyna Kopeć-Godlewska[3]
[1]Chair of Microbiology, JAGIELLONIAN UNIVERSITY MEDICAL COLLEGE; [2]Jagiellonian University; [3]JAGIELLONIAN UNIVERSITY MEDICAL COLLEGE, Kraków, Poland
**Correspondence:** Anna Różańska
*Antimicrobial Resistance and Infection Control* 2017, **6(Suppl 3):**P11

**Introduction:** Medical complications after ceasarean sections are more often than after natural deliveries. Among these complications infections are very common. Monitoring of the infections rates is a valuable way leading to help identyfy areas of healhcare demanding improving or modification.
**Objectives:** The aim of this study was the analysis of incidence and SSI microbiology in patients from Polish hospitals who underwent CC and risk factors of such infections.
**Methods:** The study was conducted using active infection surveillance in 5 Polish hospitals in the years 2013-2015 according to HAI-Net ECDC. For each procedure the following data were registered: age, date of admission to the hospital ward and date of surgery, ICD-9 code, microbiological level of contamination of the surgical field, ASA system scale, procedure time, elective/emergency procedure, use perioperative antibiotic prophylaxis or lack thereof, etiological factor of infection and the treatment used.
**Results:** SSI incidence was 0.5% and significant differences were noted among hospitals (from 0.1% to 1.8%), for different ASA scales (from 0.2% to 4.8%) and different values of standardized SSI risk index (from 0.0% to 0.8%). In 5.6% of procedures, with no antibiotic prophylaxis, or no information about it, SSI risk was significantly higher. Deep infections dominated: 61.5%, with superficial infections in only approx. 30% cases. Only 2.6% infections were detected post-discharge, without readmissions.
**Conclusion:** Results show SSI surveillance to be ineffective, considering the character of outpatient obstetric care. Without sensitive postdischarge surveillance, it's not possible to recognize the epidemiological situation, and further, to set priorities and needs when it comes to infection prophylaxis. Especially since such low incidence may indicate no need for improvement whatsoever to the infection control teams.
**Disclosure of Interest**
None Declared.

### P12

#### Forced air warming system: colonization of internal system

Letícia L. da Silva [1], Alda Graciele C. D. S. Almeida[1], João Francisco Possari[2], Vanessa B. Poveda[1]
[1]Medical and surgical nursing department, University of São Paulo; [2]Surgical Theatre, São Paulo Institute of Cancer Octavio Frias de Oliveira, São Paulo, Brazil
**Correspondence:** Vanessa B. Poveda
*Antimicrobial Resistance and Infection Control* 2017, **6(Suppl 3):**P12

**Introduction:** Perioperative hypothermia is common among surgical patients and is associated with several damages to the patient. For its prevention, several current clinical guidelines recommend the use of the forced air warming system. However, it is currently questioned the possible role forced air warming system as a risk factor for the occurrence of surgical site infection, since the airflow from these devices is often ventilated near the surgical site.
**Objectives:** To evaluate the maintenance of equipment in relation to microbiological safety and colonization.
**Methods:** This is a laboratory study, performed in a large hospital, with one equipment of forced air warming system by operating room and air conditioning system with HEPA filter. In the first stage of investigation a survey of the control of the maintenance and exchange of filters of forced air warming system was carried out. Subsequently, the evaluation of possible internal contamination of the equipment was performed by collecting the airflow emitted by the devices, placing sterile plates containing Trypticase Soy Agar (TSA) 1.5 cm from the air outlet for 1 minute. Data collection was done in triplicate. The sample of 13 (50%) equipment in use was randomly drawn by lot. After collection, the plates were incubated for 48 hours at a temperature of 36 °C ± 0.1 °C. Plaque readings occurred every 24 hours after the experiment.
**Results:** All the equipment analyzed had preventive maintenance, with filters changed in February 2016. Samples of 13 forced air warming system were collected in triplicate, totaling 39 analyzed samples. After 24 hours of the experiment, only nine (23%) samples presented growth of one or two colonies, and among the samples with microbial growth, six cases (66.6%) were present in the first sample collected.
**Conclusion:** The results evidenced the absence of significant contamination, demonstrating that the periodic maintenance, every six months, with equipment filter changes, guarantees the efficiency of the filter in relation to the microbiological safety of the equipment.
**Disclosure of Interest**
None Declared.

### P13

#### Withdrawn

### P14

#### Evaluation of a program to improve the antimicrobial prophylaxis in surgery (APS) between 2011 and 2015

Ginger G. Cabrera Tejada, Juan G. Mora Muriel, Patricia García Shimizu, Cesar O. Villanueva Ruiz, Victor M. Soler Molina, Ana Sofia L. Azevedo, Luis F. Pinto Riera, Jose Sanchez Payá
Unidad de Epidemiología, Servicio de Medicina Preventiva, Hospital General Universitario de Alicante, Instituto de Investigación Sanitaria y Biomédica de Alicante (ISABIAL - Fundación FISABIO), Alicante, Spain
**Correspondence:** Ginger G. Cabrera Tejada
*Antimicrobial Resistance and Infection Control* 2017, **6(Suppl 3):**P14

**Introduction:** Surgical site infections are one of the most common and costliest healthcare-associated infections.
**Objectives:** The aim of this study is to evaluate the effectiveness of a program to improve the adequacy of the antimicrobial prophylaxis in surgery.
**Methods:** An improvement program with elaboration-distribution of global and individual reports by services and clinical sessions were developed. Surgical site prophylaxis (SP) adequacy assessment: seven observational cohort studies, 2011 (1 study), 2012 (2), 2013 (2), 2014 (1) and 2015 (1). Patients undergoing surgery hospitalized > 24 hours ($n$ = 3,407 procedures) were included. Two indicators were calculated: overall adequacy (not performed not indicated or when performed if indicated appropriate by choice of antibiotic, start and duration); and adequacy of indicated surgical site prophylaxis (by choice, start and duration). To quantify both indicators, the adequacy ratio for 2011 to 2015 and their 95% confidence intervals were calculated. To analyze the evolution of adequacy, we used the Chi square test for trends.
**Results:** The characteristics of the surgical procedures of 2011, 2012, 2013, 2014 and 2015 were homogeneous (age, immunosuppression, clean surgery and implant surgery). The overall adequacy of SP use was 57.6% (52.9% -62.2%) in 2011, 62.5% (59.4% -65.5%) in 2012, 60.5% (57.2% -63.7%) in 2013, 74.8% (70.4% -79.1%) in 2014 and 72.5% (69.0% -75.8%) in 2015; $p = <0.001$. The adequacy of SP performed when indicated was 61.4% (55.3% -67.2%) in 2011, 61.0% (56.9% -65.1%) in 2012, 61.1% (57.1% -65.1%) in 2013, 76.1% (70.8% -81.3%) in 2014 and 74.5% (70.3% -78.7%) in 2015; $p = <0.001$.
**Conclusion:** There has been an improvement of the overall adequacy and adequacy of indicated SP in 2014 compared to previous years. Despite a decrease in 2015 compared to 2014, the observed changes are not significant. It is necessary to continue developing new interventions to improve the level of adequacy of antibiotic prophylaxis in surgery.
**Disclosure of Interest**
None Declared.

## Device-associated infections: ventilator-associated pneumonia (VAP)

### P15

#### Ventilator associated events after cardiac surgery

Siyi He, Jinbao Zhang, Hui Ouyang
Department of Cardiovascular Surgery, Chengdu Military General Hospital, Chengdu, China
**Correspondence:** Siyi He
*Antimicrobial Resistance and Infection Control* 2017, **6(Suppl 3):**P15

**Introduction:** Ventilator associated event (VAE) is a new surveillance for nosocomial infections in mechanically ventilated patients. To date, little is known about VAEs after cardiac surgeries.
**Objectives:** The present study firstly focused on patients who have undergone heart operations, intending to draw a comprehensive description of VAEs.
**Methods:** Postoperative patients from September 2012 to December 2015 were monitored for VAEs. By reviewing electronic medical records and preserved files retrospectively, clinical data were further analyzed.
**Results:** A total of 1709 adult patients were enrolled, of which 166 episodes met the criteria for VAE. The mean incidence rate reached up to 9.7% and 49.9 per 1000 mechanical ventilation days. By using both univariate analysis and multiple logistic regression analysis, chronic bronchitis, ejection fraction < 30%, cardiopulmonary bypass time, aortic clamping time, mechanical ventilation time, reintubation, dosage of blood products and peritoneal dialysis were found to be risk factors for VAEs. Compared with non-VAE group, VAEs were closely related to higher mortality, longer intensive care unit stay time and hospitalization time. In addition, 91 strains of pathogens were isolated from endotracheal aspirates of 81 patients with VAE, of which Pseudomonas aeruginosa was the most common pathogenic microorganism (30 isolates, 37.0%), followed by Acinetobacter baumannii (27 isolates, 33.3%) and other five types.
**Conclusion:** VAE algorithm is a valid and reliable surveillance for possible infections caused by mechanical ventilation, which could easily occur in patients after cardiac surgery and is associated with poor prognosis. The risks and pathogens that we have investigated will provide potential preventive strategies.

**Disclosure of Interest**

None Declared.

### P16

#### Healthcare workers' knowledge of evidence-based guidelines for the prevention of ventilator-associated pneumonia: a before/after study

Audrey DE GEA-HOMINAL[1], Caroline Landelle[1], Valérie NOCQUET[2], Emilie GENEVOIS[3], Nadia Colaizzi[1], François EGGIMANN[1], Didier PITTET[1], Stephan HARBARTH[1], Jérôme PUGIN[2]
[1]Infection Control Program; [2]Intensive Care Unit; [3]Infection control unit, University of Geneva Hospitals, Geneva, Switzerland
**Correspondence:** Audrey DE GEA-HOMINAL
*Antimicrobial Resistance and Infection Control* 2017, **6(Suppl 3):**P16

**Introduction:** Although well-documented evidence-based ventilator-associated pneumonia (VAP) prevention guidelines exist, they are rarely implemented in most intensive care units (ICU).
**Objectives:** To evaluate healthcare workers' (HCW) knowledge of these guidelines before and after a targeted educational program.
**Methods:** Voluntary HCWs of a 34-bed ICU were included. A pre-validated multiple choice self-administered questionnaire of 13 items for physicians, nurses, and physiotherapists and 7 items for nursing assistants was developed. The 1st questionnaire was completed in August 2014; a targeted educational program of 1 hour was designed for nurses, nursing assistants and physiotherapists and implemented between September 2014 and March 2015; the 2nd questionnaire was completed in December 2015.
**Results:** Among 350 HCWs, 216 (62%) completed the education session. 174 (50%) completed the 1st questionnaire (58% of nurses, 17% of nursing assistants, 19% of physicians and 6% of physiotherapists). 139 (40%) completed the 2nd questionnaire (70% of nurses, 16% of nursing assistants, 12% of physicians and 2% of physiotherapists); among them, 66% have received the educational program. The proportion of correct answers at baseline was 61 ± 19% (mean ± standard deviation); after educational session, it had increased to 65 ± 16%. A non-significant increase was observed among physicians (52 ± 19% *vs* 55 ± 13%), physiotherapists (61 ± 16% *vs* 81 ± 16%), and nurses (62.5 ± 18% *vs* 66 ± 13%) for the 1st and the 2nd questionnaire, respectively. Level of knowledge for nursing assistants remained unchanged (67 ± 21% *vs* 66 ± 27%). Multivariate analysis revealed that being a physician was the only factor (OR 2.5, IC95%: 1.2-5.4) associated with lower knowledge level (<63%) adjusted on level of experience and training by the educational program.
**Conclusion:** Level of evidence-based VAP prevention measures' knowledge is high in our institution. Our educational program doesn't increase significantly the level of knowledge. Interns were identified as the target population that could have the highest benefit of educational strategies to improve their knowledge.

**Disclosure of Interest**

None Declared

### P17

#### Effect of the ventilator-associated pneumonia bundle in a medical intensive care unit

Hye-Ran Choi[1], Song Yi Yoo[2], Jae Sim Jeong[3]
[1]Department of Clinical Nursing, University of Ulsan College of Medicine; [2]Department of Clinical Nursing, University of Ulsan Graduate School of Industrial Technology; [3]Department of Nursing, University of Ulsan College of Medicine, Seoul, Korea, Republic Of
**Correspondence:** Hye-Ran Choi
*Antimicrobial Resistance and Infection Control* 2017, **6(Suppl 3):**P17

**Introduction:** Ventilator-associated pneumonia(VAP) in the intensive care unit affects the patient mortality and the bundle is used as an intervention to reduce it.
**Objectives:** This study was to investigate the compliance of the VAP bundle and to determine the effect of the bundle on the decrease of VAP rate.
**Methods:** A pretest-posttest experimental study was done. Patients in the medical intensive care unit with a 48-hour ventilator were included. The incidence of VAP was compared between January 2014 and June 2014 before implementation of the bundle and between October 2014 and March 2015 after implementation. There were 112 subjects before application of bundle and 107 subjects after application. VAP bundles included sedative interruption and assessment of readiness to extubate, elevation of the head of the bed 30°, peptic ulcer disease prophylaxis, deep vein thrombosis prophylaxis, daily oral care with 0.12% chlorhexidine, intra-cuff pressure control, and aspiration of subglottic secretions.
**Results:** After the application of bundles, the compliance of nurses increased from 27.6%(8/29) to 65.5%(19/29) and VAP cases decreased from 3 to 1. The ventilator days were 2,143 days before the intervention and 2,232 days after the intervention. The VAP rate per 1,000 ventilator insertion days was 1.40 before the intervention and decreased to 0.45 after the intervention, but there was no significant difference.
**Conclusion:** There was no statistically significant decrease in VAP incidence, but bundle compliance was increased. However, it is meaningful that we conducted a study on bundle compliance in Korea. It is recommended that a bundle suitable for the Korean situation be developed.

**Disclosure of Interest**

None Declared.

### P18

#### Is it possible to decrease the rate of ventilator-associated pneumonia with infection control prevention bundle

Mehtap Aydin[1], Funda Timurkaynak[1], Hande Arslan[2]
[1]Infectious Disease and Clinical Microbiology, Başkent University, Istanbul; [2]Infectious Disease and Clinical Microbiology, Başkent University, Ankara, Turkey
**Correspondence:** Mehtap Aydin
*Antimicrobial Resistance and Infection Control* 2017, **6(Suppl 3):**P18

**Introduction:** Ventilator-associatedpneumonia (VAP) is a commonhealthcareassociatedinfection in theintensivecareunit (ICU) withhighmortalityandmorbidity.
**Objectives:** The aim of this study was to investigate the effectiveness of the infection control bundle on VAP) rate in ICU.
**Methods:** A VAP preventionbundleincluding hand hygiene, oral chlorhexidine use, 30-45 degree head positioning, compliance with aseptic aspiration techniques, daily evaluation of sedation score was prepared and implemented in ICU on January 2014. Training sessions were held with intensive care workers before the practice. The information about the importance of scientific evidence, content and how to apply the precautionary bundle in reducing the VAP rate was given. During the first three-month period the infection control nurse trained the intensive care workers beside the patient for correct application of thebundle steps. Observedinconsistencies were recorded. Feedback meetings were held monthly with staff about the compliance rate of the bundle,with the results of the observation.VAP rate was monitored by active surveillance. The obtaineddata were compared with the year 2013, when the prevention bundle was not implemented. All data were evaluated using software program (version 15.0) from SPSS (SPSS Inc., Chicago, IL, USA).
**Results:** The VAP rate was13.05 / 1000 ventilator days before the prevention bundle was implemented, it decreased to 2.68/1000 and 1.94/1000 ventilator days in 2014 and 2015 consecutively. We observed that althoughthere were more ICUpatient daysin 2014 and 2015 when compared with 2013; the rate of VAP decreasedafter implementationof the VAP bundle ($r = 0.96$; $R^2 = 0.92$) ($p < 0.05$).A

strong linear positive correlation was found between the ventilator utilization ratio and the VAP rate. Both a decrease in ventilator utilization days and VAP incidence was observed after the implementation of the bundle ($r = 0.99$; $R^2 = 0.98$) ($p < 0.05$).

**Conclusion:** It is possible to achieve significant reductions in VAP rates despite increases in patient days by implementing an infection control prevention bundle.

**Disclosure of Interest**

None Declared.

### P20

#### Ventilator associated pneumonia in intensive care unit and their antibiotics sensitivity pattern (2012-16) At Al Qassim region of Saudi Arabia

Sanjay K. Gupta, Ibrahim S. Al Harby, Omar H. Romaih, Sinimol Jabar, Sunitha L. Mathias
Infection control, AR RASS HOSPITAL, Ar Rass, Saudi Arabia
**Correspondence:** Sanjay K. Gupta
*Antimicrobial Resistance and Infection Control* 2017, **6(Suppl 3):**P20

**Abstract video clip: Introduction:** Ventilator-associated pneumonia (VAP) is defined as pneumonia that occurs 48-72 hours or thereafter following endotracheal intubation, characterized by the presence of a new or progressive infiltrate, signs of systemic infection like (fever, altered white blood cell count), changes in sputum characteristics, and detection of a causative agent [4]. **Objective:** To find out the hospital wide prevalence of the ventilator associated pneumonia and their antibiotics sensitivity patterns. **Methodology:** The retrospective data were analyzed from ICU, collected by infection control department about VAP and confirmed from laboratory. **Observations:** In the last five years 20.24% cases of VAP were reported out of total health care associated infection. The male cases were nearly four times 54 (78%) higher than females 15 (22%) and difference is statistically significant ($X^2 = 15.2$, $P < 0.05$), according to age common among above 60 years 24 (35%) and least common in below 15 years of age 04 (6%), the difference between two groups is significant ($X^2 = 12.1$, $P < 0.05$). If we see the year wise pattern of distribution of VAP cases we were found increasing trend from 2012-2014, 18%, 21% and 25% respectively. Mean hospital wide infection rate in 2015 was higher 4.34 (SD = 3.33) than 2016 2.14 (SD = 2.25). The most common bacteria reported in VAP cases was Acenatobacter B 72% followed by pseudomonas aeruginosa 20%. The sensitivity pattern of the most common organism responsible for VAP was Acenatobacter B 72%, in 2015-16 20% sensitive to amikacin, ceftazidime, ciprofloxacin, Pipaeracillin and imipenem and resistance to gentamicin, cefotaxima then slowly reported 80% of isolates resistance to most of the drugs only sensitive to imipenem now.Second common organism discover in VAP cases was Pseudomonas A, but this also slowly develop resistance to most of the commonly use antimicrobial drugs and 90% of isolates now only sensitive to imipenem. **Conclusion:** The VAP was common among male and above 60 years of age, most common causative organism during the study period was Acenatobacter B. The mean hospital wide VAP infection rate was 4.34 and most of the isolates resistant to most of the antibiotics and sensitive to only imipenem.

**Disclosure of Interest**

None Declared.

## *Clostridium difficile*

### P21

#### Epidemiological and microbiological aspects of clostridium difficile infections in Hungary: results from the newly launched european surveillance

Ágnes Hajdu[1], Andrea Kurcz[1], Éva Popovics[2], Virág Lesinszki[3], Judit Pászti[3]
[1]Dept. of Hospital Epidemiology and Hygiene; [2]Dept. of Bacteriology; [3]Dept. of Phage-Typing and Molecular Epidemiology, National Center for Epidemiology, Budapest, Hungary
**Correspondence:** Ágnes Hajdu
*Antimicrobial Resistance and Infection Control* 2017, **6(Suppl 3):**P21

**Introduction:** Since 2012, mandatory notification of healthcare-associated Clostridium difficile infections (HA-CDI) has been in place in Hungary, following a national protocol used in acute and chronic care hospitals.

**Objectives:** To further improve the national surveillance, a start-up data collection based on the CDI surveillance protocol v2.2 of the European Centre for Disease Prevention and Control was organised.

**Methods:** We invited all hospitals in Hungary to complement the mandatory HA-CDI surveillance in February 2016 with additional variables of the European protocol: hospital type, testing frequency, positivity rate, diagnostic algorithm used; case-based data on community-acquired CDI (CA-CDI), CDI of unknown origin (UO-CDI) and recurrent cases. Twenty hospitals were asked to send five C. difficile isolates per hospital for PCR-ribotyping to the National Center for Epidemiology, Budapest.

**Results:** Overall 45 Hungarian hospitals participated in the European CDI start-up data collection, 42 reporting at least one case (59% of the hospitals reporting into the mandatory surveillance in February 2016). In total, 441 CDI were reported, of these 58% primary and 6% recurrent cases (36% unknown). The proportion of HA-CDI, CA-CDI and UO-CDI was 90%, 5% and 5%, respectively. The median incidence of HA-CDI was the lowest in primary and the highest in tertiary care hospitals (1.7 and 4.3 cases/10000 patient-days, respectively). Combined diagnostic tests (GDH + toxin A/B) were used predominantly. Testing frequency has notably differed across hospital types and showed lineral correlation with CDI incidence ($R2 = 0.4$). Eighty-five isolates were ribotyped: 58 (68%) were RT027, 23 (27%) belonged to other types (e.g. RT012, RT035, RT036), and 4 were non-typeable.

**Conclusion:** We conclude that participation in the European surveillance is valuable and can offer a better understanding of the diagnostic practices, total CDI burden and circulating C. difficile ribotypes in Hungarian hospitals, also through future inter-country comparisons.

**Disclosure of Interest**

None Declared.

### P22

#### Reduction in c. difficile hospital spread through cleaning control and hand hygiene improvement

Pawel Grzesiowski[1], Danuta Pawlik[2], Magdalena Sekuna[3]
[1]Institute for Infection Prevention, Warsaw; [2]Microbiology; [3]Surgery, County Hospital, Makow Maz, Poland
**Correspondence:** Pawel Grzesiowski
*Antimicrobial Resistance and Infection Control* 2017, **6(Suppl 3):**P22

**Introduction:** Health-care associated Clostridium difficile infection (HAI-CDI) is currently the most prevalent cause of hospital outbreaks in Poland. From 350 hospital outbreaks reported to the national surveillance system in 2015, almost 35% have been caused by CDI. In primary care hospitals, the highest risk of HAI-CDI is connected to prolonged hospitalization in non-invasive medicine wards and intensive antibiotic use.

**Objectives:** This prospective single hospital study has been developed to assess the effectiveness of bi-modal strategy to reduce HAI-CDI in non-invasive wards. The strategy included fluorescent markers introduction to improve and control cleaning procedures and hand hygiene improvement according to WHO Guidelines "Clean Care is Safer Care".

**Methods:** Between 2012 and 2016 the bi-modal strategy to reduce HAI-CDI has been developed by the Infection Control Team in the primary care 300-bed hospital in central region of Poland. The intervention consisted of two phases. The first phase has been based on cleaning personel training program and introduction of fluorescent markers according to En-Compas (Ecolab) system. The second phase consisted of introduction of the WHO Multimodal Hand Hygiene Improvement Strategy.

**Results:** Before the bi-modal strategy has been established, accuracy of the cleaning procedure according to fluorescent markers testing was 30%. After the first phase of the project, cleaning accuracy increased significantly to 92% ($p < 0,01$). During the second phase, the substantial improvement in hand hygiene of medical personel has been observed, as measured by direct observation (mean rate 35% vs 75%) and volume of hand disinfectants used (12 vs 19 liters per 1000 patient-days respectively). During 4 years of the observation, the incidence of HAI-CDI significantly decreased from 54 cases to 5 cases per year (6,75 vs 0,5 per 1000 hospitalizations, respectively) ($p < 0{,}01$).

**Conclusion:** According to our results, the bi-modal strategy based on cleaning improvement and implementing WHO Multimodal Hand Hygiene Improvement Strategy, is highly effective way to deal with HAI-CDI in primary care hospital. The usage of fluorescent markers for routine control of cleanig procedures is simple and well accepted by the trained personel.

**Disclosure of Interest**

None Declared.

### P23

#### Hospital wide reduction of clostridium difficile after a cleaning intervention in three wards

Birgitta Lytsy

Department of Clinical Microbiology and Infection Control, Uppsala University Hospital, Uppsala, Sweden

*Antimicrobial Resistance and Infection Control* 2017, **6(Suppl 3):**P23

**Introduction:** Uppsala University Hospital (UUH) in Sweden is a 1000 bed, highly specialized public hospital with 80 wards each housing 20-25 patients, in 1-4 bed rooms, with five intensive care units providing a region of 3 million inhabitants of tertiary care. About 10% of the patient beds are located in single rooms with private en-suite bathrooms. According to the Swedish national surveillance system UUH has over the last 10 years had the highest incidence of *Clostridium difficile* compared to other Swedish university hospitals. Terminal cleaning with natirumhypochlorite has been shown to be important in order to limit the sprad of C. difficile spores in the hospital environment (1).

**Objectives:** To show how a hygienic intervention consisting of terminal cleaning of rooms post discharge with natriumhypochlorite in three wards reduced the overall incidence of *Clostridium difficile* at UUH between 2013 and 2016.

**Methods:** Three wards; one geriatric, one internal medicine and one infectious disease ward which had the highest incidence of *Clostridium difficile* at UUH were included in the intervention starting in May 2015 and is still ongoing. The intervention consisted of terminal cleaning of rooms post-discharge with natriumhypochlorite when the room had been previously occupied by a patient colonized or infected by *Clostridium difficile.* No other IPC measures and other interventions such as antibiotic stewardship and other had been implemented in the three wards during this intervention.

**Results:** The number of new cases with *Clostridium difficile* at UUH decreased from 302 in 2014 to 189 in 2016 according to the Swedish national surveillance system health-care associated infections. The overall *Clostridium difficile* incidence rates at UUH decreased at UUH from 0.53 cases in 2014 to 0,34 cases per 1000 patient-days in 2016.

**Conclusion:** Terminal cleaning of rooms post-discharge with natriumhypochlorite reduced the incidence of *Clostridium difficile* in three wards with many cases which reflected on a reduction of hospital wide figures of incidence.

**Disclosure of Interest**

None Declared.

### P24

#### Effectiveness of various cleaning and disinfectant products on clostridium difficile spores of pcr ribotypes 010, 014 and 027

Nikki Kenters[1], Elisabeth Huijskens[1,2], Sophie De Wit[1], Ingrid Sanders[3], Joost Van Rosmalen[4], Ed Kuijpers[3], Andreas Voss[5,6]

[1]Department of Infection Prevention and Control; [2]Department of Medical Microbiology, Albert Schweitzer hospital, Dordrecht; [3]Section Experimental Bacteriology, Department Medical Microbiology, Leiden University Medical Centre, Leiden; [4]Department of Biostatistics, ErasmusMC, Rotterdam; [5]Department of Medical Microbiology, Radboud University Medical Centre; [6]Department of Medical Microbiology, Canisius Wilhelmina Hospital, Nijmegen, Netherlands

**Correspondence:** Nikki Kenters

*Antimicrobial Resistance and Infection Control* 2017, **6(Suppl 3):**P24

**Introduction:** In healthcare facilities, *Clostridium difficile* infections spread by transmission of bacterial spores. Appropriate sporicidal disinfectants are needed to prevent development of clusters and outbreaks.

**Objectives:** In this study different cleaning/disinfecting wipes and sprays were tested for their efficacy against spores of distinctive *C. difficile* PCR ribotypes.

**Methods:** Four different products were tested; 1) hydrogen peroxide 1.5%; 2) glucoprotamin 1.5%; 3) a mixture of ethanol, propane and N-alkyl amino propyl glycine; and 4) a mixture of didecyldimonium chloride, benzalkonium chloride, polyaminopropyl, biguanide and dimenthicone as active ingredients. Tiles were contaminated with a test solution containing a concentration of $5x10^6$CFU/ml spores of *C. difficile* strains belonging to PCR ribotypes 010, 014 or 027. The tiles were left to dry for an hour and then wiped or sprayed with one of the sprays or wipes as intended by the manufacturers. When products neutralized after 5 minutes microbiological cultures and ATP measures were performed.

**Results:** Irrespective of the disinfecting method, the microbial count $log_{10}$ reduction of *C. difficile* PCR ribotype 010 was highest, followed by the reduction of *C. difficile* 014 and *C. difficile* 027. Overall, the wipes performed better than the sprays with the same active ingredient. On average, although not significantly, a difference in relative light units (RLU) reduction between the wipes and sprays was found. The wipes had a higher RLU $log_{10}$ reduction, but no significant difference for RLU reduction was observed between the different *C. difficile* strains ($p = 0.16$).

**Conclusion:** *C. difficile* spores of PCR ribotypes 014 and 027 strains are more difficult to eradicate than non-toxigenic PCR ribotype 010. In general, impregnated cleaning/disinfection wipes performed better than ready-to-use sprays. Wipes with hydrogen peroxide (1.5%) showed the highest bactericidal activity.

**Disclosure of Interest**

None Declared.

### P25

#### Effects of rhodomyrtone on hypervirulent clostridium difficile strain

Sutthirat Srisuwan[1], Kate E. Mackin[2], Dianna Hocking[3], Dena Lyras[2], Roy M. Robins-Browne[3], Supayang P. Voravuthikunchai[4]

[1]Microbiology and Excellence Research Laboratory on Natural Products, Faculty of Science and Natural Products Research Center of Excellence, Prince of Songkla University, hat yai, Thailand; [2]Microbiology, Monash University; [3]Microbiology and Immunology, the University of Melbourne, Victoria, Australia; [4]Microbiology and Excellence Research Laboratory on Natural Products, Faculty of Science and Natural Products Research Center of Excellence, Prince of Songkla University, Songkhla, Thailand

**Correspondence:** Sutthirat Srisuwan

*Antimicrobial Resistance and Infection Control* 2017, **6(Suppl 3):**P25

**Introduction:** *Clostridium difficile* infection (CDI) is a significant increase in hospital-acquired antibiotic-associated diarrhea resulting in high mortality and healthcare cost. The incidence of CDI requires an urgent need for alternative treatment options since the emergence of epidemic hypervirulent strain BI/NAP1/027 is the key contribution to the worldwide. Rhodomyrtone, a bioactive compound from *Rhodomyrtus tomentosa* leaf extract, has potent antibacterial activity against several Gram-positive bacteria.
**Objectives:** The aim of this study was to examine an antibacterial activity of rhodomyrtone against *C. difficile* cells and their spores.
**Methods:** *C. difficile* and their spores were treated with rhodomyrtone or vanomycin to determine the susceptibility using broth microdilution method and time kill assay. In addition, transmission electron microscopy was used to confirm anti-difficile activity.
**Results:** Rhodomyrtone demonstrated an anti-difficile activity at 0.625-2.5 mg/L, which was close to that of vancomycin. Bactericidal activity of rhodomyrtone ranged from 1.25-5 mg/L against 10 strains including hypervirulent strain BI/NAP1/027 and multidrug-resistant strain 630, whereas MBCs of vancomycin were much higher in most strains (20 to ˃40 mg/L). Time kill study showed rapidly bactericidal effects of rhodomyrtone against the hypervirulent strain with ≥ 99 killing within 4 h and more bactericidal than vancomycin (16 h). Rhodomyrtone at 8× MIC destroyed the treated cells as observed by TEM. Moreover, rhodomyrtone was 4-fold more efficient than in vancomycin in inhibiting spore outgrowth.
**Conclusion:** It can be concluded that rhodomyrtone was effective in combating with *C. difficile* by possessing germicidal activity against the vegetative cells. These data support further development of rhodomyrtone as a candidate agent for treatment of CDI.
**Disclosure of Interest**
None Declared.

### P26
### Severe cases of clostridium difficile infections (SCDI) in North Rhine-Westphalia (NRW), 2008-2016

Annette Jurke[1], Mechthild Lunemann[1], Inka Daniels-Haardt[2]
[1]Infectiology and Hygiene; [2]Health Protection, Health Promotion, NRW Centre for Health, Münster, Germany
**Correspondence:** Annette Jurke
*Antimicrobial Resistance and Infection Control* 2017, **6(Suppl 3):**P26

**Introduction:** In Germany in November 2007 sCDI has been classified as threatening disease with evidence of serious danger to the community. These cases are notifiable according to Infection Protection Act (IfSG).
**Objectives:** We characterize the sCDI epidemiology state-wide from 2008 to 2016 .
**Methods:** We used the sCDI-notifications of the 53 local health departments submitted to NRW Centre for Health. The CDAD Reference data 2015 of German Reference Centre for Surveillance of nosocomial Infections were used to estimate possible underreporting. The number of hospitals and inpatients in NRW in 2015 were taken from NRW hospital statistics.
**Results:** In NRW the number of notified sCDI cases was 61 in 2008 and 2009; 73 in 2010; 145 in 2011; 360 in 2012; 469 in 2013; 710 in 2014; 1,094 in 2015 and 815 in 2016. Extrapolating from data of 415 hospitals, which took part in the German CDI surveillance in 2015, we estimate for the 352 hospitals in NRW covering 4,546,310 inpatients in 2015 about 1,359 sCDI-cases. The 1,094 sCDI cases, submitted to the NRW Centre for Health in 2015, may indicate underreporting. Out of the 1,094 sCDI cases 283 (25.9%) were notified because of detection of ribotype O27 only. From 2008 to 2015 in NRW in 31.8% of sCDI cases a detection of ribotype O27 is specified; in 24.8% CDI-death; in 23.8% a CDI-relapse; in 15.6% a transfer to ICU and in 3.9% surgery. According to the new case definition from May 2016 to December 2016 in NRW in 26.3% of sCDI cases a CDI-death is specified; in 30.6% a transfer to ICU, in 7.4% surgery and in 35.7% in-hospital treatment of community acquired sCDI.
**Conclusion:** The complete registration of sCDI is still a challenge. National surveillance data can be used to estimate regional underreporting. From 2008 to 2015 the increasing trend of sCDI notifications may indicate a decrease in underreporting. The high percentage of notified sCDI cases with detection of ribotype O27 only may indicate that O27 CDI not always imply a severe course, justifying the elimination of detection of ribotyp O27 only in the sCDI case definition. Replacing the frequent notification criteria CDI-relapse by data on in-hospital-treatment of community acquired CDI the notification data allows for describing the sCDI epidemiology in outpatients and consequently more complete.
**Disclosure of Interest**
None Declared.

### P27
### Correlation of antibiotic usage and clostridium difficile infections in a German University Hospital

Anna Rohde[1], Janine Zweigner[1, 2], Miriam Wiese-Posselt[1], Michael Behnke[1], Frank Schwab[1], Petra Gastmeier[1]
[1]Institute for Hygiene and Environmental Medicine, Charité - Berlin, Berlin; [2]Department of Hospital Hygiene and Infection Con, University Hospital Cologne, Cologne, Germany
**Correspondence:** Anna Rohde
*Antimicrobial Resistance and Infection Control* 2017, **6(Suppl 3):**P27

**Introduction:** Systemic antibiotic usage plays an important role in the emergence of *Clostridium difficile* infections (CDI).
**Objectives:** The aim of this study was to assess a possible correlation of antibiotic usage and CDI in Germany's largest university hospital.
**Methods:** In 2014, CDI occurrence, number of patients and patient days and antibiotic usage were monitored monthly in 61 wards. Paediatric, psychiatric, dermatological, otorhinolaryngological and ophthalmological wards were excluded. CDI cases were defined as patients presenting with loose stools and microbiological finding of toxin-producing *C. difficile*. CDI with onset in the first 3 days of hospital stay and without hospital admission in the previous 4 weeks was classified as community-acquired (CA-CDI). If previous hospital admission was reported and cases with onset later than day 3 were classified as hospital-acquired (HA-CDI). Spearman correlation of antibiotic usage and HA-CDI incidence density and regression analysis were performed to examine the effect of length of stay (LOS), bed occupancy, incidence of CA-CDI, department type and antibiotic usage on HA-CDI.
**Results:** The surveillance period included over 101 000 patient admissions and almost 471 000 patient days in the 14 ICUs, 9 haematological, 20 surgical and 18 medical wards (the latter include internal medicine, neurology and obstetrics). We observed 95 CA- and 172 HA-CDI cases. The median usage of systemic antibiotics was 74.6 DDD per 100 patient days (IQR 61.5-138.2). Multivariable analysis identified the department type as risk factor for HA-CDI: ICUs had a 16.9 times higher (IRR, 95% CI 4.5-63.1), haematological wards a 15.3 times higher (IRR, 95% CI 4.5-51.3) and medical wards a 2.8 (IRR, 95% CI 0.5-16.0) times higher HA-CDI incidence density than surgical wards. Only ciprofloxacin (correlation coefficient of 0.369) was identified as independent risk factor for HA-CDI in multivariable analysis. Irrespective of department type, an increase of 1 DDD ciprofloxacin/100 patient days increased the ward's HA-CDI incidence density by 3%.
**Conclusion:** Our study showed a prominent role of the ward's department type and could confirm ciprofloxacin (but not clindamycin or cephalosporins) as independent risk factor for HA-CDI.
**Disclosure of Interest**
None Declared.

### P28
### Surveillance of clostridium difficile infections in a Northern Italian Hospital, 2005-2015: trends and control measures

Loss Rosanna[1], Bombonato Mirella[1], Lopez Maria[1], Santa Peter[1], Pagani Elisabetta[2], Aschbacher Richard[2]
[1]Medical Direction Central Hospital of Bolzano; [2]Laboratory of Microbiology and Virology Central Hospital of Bolzano, Bozen, Italy
**Correspondence:** Loss Rosanna
*Antimicrobial Resistance and Infection Control* 2017, **6(Suppl 3):**P28

**Introduction:** Intensive surveillance and prevention of *Clostridium difficile* (CD) in the Central Hospital of Bolzano, Italy, started after detection of an epidemic cluster in March 2005.
**Objectives:** To evaluate the trends of *C. difficile* infection (CDI) over following ten-year period 2005-2015, in relation to control measures and education of hospital ward staff.
**Methods:** Stool samples were screened for CD toxins A/B by immunoassay (Vidas® *C. difficile* A&B, bioMérieux) and notified to the Hospital Hygiene Service (HHS). Annual incidence rates were calculated as number of patients with at least one toxin A/B positive stool sample per 10.000 patient bed-days.
**Results:** A total of 1160 CD toxin A/B positive patients were identified, with an incidence decreasing from 10,0 (2005) to 3,1 (2015) per 10.000 patient bed-days. Since the beginning following control interventions were adopted:1) Contact precautions: An …ad hoc" protocol was developed and implemented for all involved categories (health professionals, cleaning staff, patients, visitors). 2) Surveillance of new cases: Regular surveillance and epidemiological reports were provided to clinicians by using the software Virtuoso Plus (Dedalus Healthcare Systems Group). 3) Introduction of new assays for routine laboratory diagnosis and molecular characterization of CD isolates (CD culture, GDH-testing combined with toxin A/B testing, direct molecular toxin B gene testing and presumptive identification of ribotype 027). 4) Control and restriction of antibiotics use by the Hospital Pharmacy, in collaboration with the Infectious Diseases Ward. 5) Teaching of CDI implications and correct hand hygiene by the HHS was offered to hospital wards. From 2005 to 2015 education sessions for physicians and nurses (Median 4 hours per year) and Conferences were conducted in the most involved wards. 6) An information leaflet for CDI was provided to patients and carers.
**Conclusion:** A marked decrease in the incidence of CDI in the Central Hospital of Bolzano during the 10-year study period and the prevention of its further spread was probably the result of educational efforts and intense collaboration between different professionals, leading to the implementation of various control measures.
**Disclosure of Interest**
None Declared

### P29

#### Burden of clostridium difficile infection among hospitalized patients in Slovakia

Maria K. Garabasova[1], Maria Stefkovicova[1, 2], Jaroslava Brnova[3], Elena Novakova[4], Slavka Litvova[1], Iveta Matišáková[2]
[1]Department of Epidemiology, Regional Public Health Authority; [2]Department of Laboratory Medicine and Public Health, Faculty of Health Care, Alexander Dubček University, Trenčín; [3]Department of Laboratory Medicine, School of Health Sciences and Social Work, Trnava University, Trnava; [4]Department of Microbiology and Immunology, Jessenius Faculty of Medicine, Martin, Slovakia
**Correspondence:** Jaroslava Brnova
*Antimicrobial Resistance and Infection Control* 2017, **6(Suppl 3):**P29

**Introduction:** Clostridium difficile infections (CDI) are currently the leading cause of healthcare-associated infections, but in Slovakia are the data underestimated and lack of standardized surveillance.
**Objectives:** The objectives for this study were to estimate the incidence of CDI in Slovak acute care hospitals and assess the burden of CDI.
**Methods:** This hospital - based multicenter study was conducted using all patients admitted to 30 acute care hospitals from October 1, 2016 through December 31, 2016. The diagnosis of CDI was determined based on anamnestic data, epidemiological data, clinical picture (basic clinical symptoms and signs), microbiological tests (positive laboratory assay for C. difficile toxin A and/or B in stools) according to the protocol of ECDC. Descriptive statistical parameters have been used for the determination of baseline characteristics.
**Results:** During a 3-month hospital – based study we identified 325 cases of *CDI*. Of these cases, 84.3% were health care–associated (HA-CDI) and 15.7% were community-associated infection (CA-CDI). The mean incidence rate of HA - CDI was 2.7 per 10,000 patient days and rates of hospitalization were 17.0 per 10,000 admissions. Incidence rate of CA-CDI was 0.6 per 10,000 patient days and rates of hospitalization were 3,3 per 10,000 admissions. Most patients had a previously identified risk profile of old age (72,3% aged ≥65 years), comorbidity and recent use of antibiotics. McCabe score categories rapidly fatal were registred in 10.8% patients and ultimately fatal disease in 18.2% patients. Complicated CDI were registered in 13.2% patients and recurrent infections in 3.4%.
**Conclusion:** Increasing incidence of CDI and changing epidemiology highlight need for standardized definitions and surveillance methods. Our data suggest that CDI is largely present in our setting which represents a serious problem and points to the importance of continuous surveillance, detection and control of CDI.
**Disclosure of Interest**
None Declared

### P30

#### Enhanced surveillance of clostridium difficile infection: a reassessment of 2015-2016 reporting in greater Glasgow & Clyde, Scotland

Laura Cottom[1,2], Teresa Inkster[2]
[1]Department of Medical Microbiology, Glasgow Royal Infirmary; [2]Department of Medical Microbiology, Queen Elizabeth University Hospital, Glasgow, United Kingdom
**Correspondence:** Laura Cottom
*Antimicrobial Resistance and Infection Control* 2017, **6(Suppl 3):**P30

**Introduction:** *Clostridium difficile* infection (CDI) remains an important cause of diarrhoea and colitis, associated with significant morbidity and mortality. Since its first description in 1978, CDI has principally been considered a nosocomial disease. Primarily associated with increasing age, hospitalisation and antibiotic exposure; surveillance programs focused on healthcare associated infection (HAI). The epidemiology has changed over the last decade, however limited information exists for community-associated infection. Case ascertainment and definitions used in mandatory reporting also vary across Europe. Our surveillance programme previously categorised cases into hospital or broadly into non–hospital/community acquired.
**Objectives:** Following an increase in 'non-HAI' CDI cases during the 3rd quarter of 2016 we implemented enhanced surveillance and retrospectively reclassified cases.
**Methods:** Analysis was performed over 12-months (Oct.15-Sept.16). Data was provided by the Surveillance IPC team. Patients were identified using electronic clinical records and our laboratory information management system. Cases were reclassified as either; Hospital acquired, Healthcare associated, Indeterminate or Community associated. A comparison between the two classification systems was made.
**Results:** 137 cases over the first 3-months were identified. 39%(54/137) were defined as HAI and 61% as non-HAI. Using the enhanced surveillance definitions, the same 39% were reclassified as Hospital acquired. Of the 61% previous non-HAI cases; 25% were reclassified as Healthcare associated, 21% were classified as community-associated CDI and 15% were classified as Indeterminate. Of interest no further information would have been reported for the 83 non-HAI cases according to the previous surveillance programme.
**Conclusion:** CDI continues to pose a significant burden on patients, clinicians and the National Heath Service. This study provides a crucial insight into the incidence of true community-associated versus CDI associated with hospital admission. Enhanced surveillance better informs public health guidance, allowing a more detailed epidemiological assessment. Importantly improving our understanding of CDI also allows risk stratification and targeted prevention strategies.
**Disclosure of Interest**
None Declared

### P31

#### Healthcare resource use and direct costs of recurrent clostridium difficile infection in hospitalised patients

Simon Goldenberg, Rahul Batra
Guy's and St Thomas' NHS Foundation Trust, London, United Kingdom
**Correspondence:** Simon Goldenberg
*Antimicrobial Resistance and Infection Control* 2017, **6(Suppl 3):**P31

**Introduction:** *Clostridium difficile* Infection (CDI) is associated with significant economic burden. There is limited detailed data examining resource use and financial cost of hospitalised patients with CDI.
**Objectives:** To measure financial costs associated with recurrent CDI.
**Methods:** A retrospective case-control study in an NHS hospital including 32 patients with rCDI and 32 matched controls (index case of CDI but no recurrence) between 2013 and 2015. Patients were matched by date of CDI diagnosis (±3 months), age and sex.
Hospital finance systems were used to compare detailed financial costs for a range of categories including the following: drugs; blood and blood products; dialysis; radiotherapy; physiotherapy and occupational therapy; endoscopy; imaging; pathology; staffing costs comprising medical (training and non-training), nursing, administrative and clerical, professional and technical; ward costs; High Dependency Unit costs; Critical Care Unit costs; operating theatres costs; linen; sterile services; medical physics; catering; patient transport; portering; security; clinical coding; clinical audit; insurance; overheads.
**Results:** Mean length of stay was longer in those with rCDI (26.4 days) compared with those with index CDI (18.4) although this was not significant. There were significant differences in mean costs for the following (rCDI, index only, p value):
Staffing costs: medical non-training grades £1019, £387.40, <0.01
Pathology £776.70, £335, <0.01
Sterile services £31.61, £8.41, <0.01
Surgical appliances £8.41, £0.38, <0.04
Departmental support £9.99, £3.99, <0.01 Clinical coding £40.29, £28.65, <0.03
Clinical audit £5.45, £2.45, <0.03
Security £4.33, £2.19, 0.02
Training £4.24, £0.17, <0.01
Overheads £3950, £2155, <0.03
Total costs £26,568, £15,796, <0.02
Costs for other categories were not significantly different.
**Conclusion:** rCDI is associated with significant costs which do not appear to be significantly associated with extended hospital stay. The majority of the excess costs comprised additional staffing, pathology and other 'hidden' costs that have been previously unrecognised e.g. clinical coding and audit, security, training and overheads. Higher costs for prevention strategies and treatment modalities that can reduce risk of CDI recurrence may be justified when this excess financial cost is taken into consideration.
**Disclosure of Interest**
S. Goldenberg Grant/Research support from: Astellas, Speaker's bureau of: Astellas, MSD, Consultant for: Astellas, MSD, R. Batra: None Declared.

### P32

#### Clinical characteristics of community-onset clostridium difficile infection and the factors associated with complication

Eunyoung Lee[1,2], Kyoung-Ho Song[1,2], Ji Yun Bae[2], Doran Yoon[2], Joo-Hee Hwang[2], Pyoeng Gyun Choe[2], Wan Beom Park[2], Ji Hwan Bang[2], Eu Suk Kim[1,2], Sang Won Park[2], Nam Joong Kim[2], Myoung-don Oh[2], Hong-Bin Kim[1, 2]
[1]Department of Internal Medicine, Seoul National University Bundang Hospital, Seongnam-si, Gyeonggi-do; [2]Department of Internal Medicine, Seoul National University College of Medicine, Seoul, Korea, Republic Of
**Correspondence:** Eunyoung Lee
*Antimicrobial Resistance and Infection Control* 2017, **6(Suppl 3):**P32

**Introduction:** Recent studies reported that a substantial portion of *Clostridium difficile* infection (CDI) occurred in the community and the community-onset CDI (CO-CDI) can lead to various complications including mortality.
**Objectives:** We evaluated the clinical characteristics of CO-CDI and elucidated risk factors for complication.
**Methods:** We performed a retrospective review of all inpatients with CDI of a 1,300 bed tertiary-care hospital in Korea from 2008 through 2015. CDI was defined as positive or equivalent result of the fecal toxin assay in patients with diarrhea. CO-CDI was defined as CDI occurring within 48 hours of admission. Complicated CO-CDI was defined if at least one of following event was occurred: 1) all-cause 30-day or in-hospital mortality, or 2) surgery due to CDI.
**Results:** Of total 1256 CDI during 8 years, 153 cases (12.2%) were classified as CO-CDI and 23 (15.0%) had complications, including 22 cases (14.4%) with mortality. 110 cases (71.9%) received antibiotic treatment within 30 days of the diagnosis of CO-CDI. 38 (24.8%) were long-term care residents, and 23 (15.0%) had previous CDI history. Mean age of patients with complicated infection was higher than non-complicated patients (75.8 *vs.* 69.6, $p = 0.03$) Proportion of men and prior proton pump inhibitor (PPI) use were also significantly higher in complicated group (65.2% *vs.* 41.5%, $p = 0.04$, 39.3% *vs.* 17.5%, $p = 0.02$, respectively). According to antibiotic classification, penicillin-derivatives were more frequently used in complicated group (40.0% *vs.* 16.3%, $p = 0.04$). Multivariate regression model showed leukocytosis (>20,000/μL) was significantly associated with complications (adjusted odds ratio [aOR], 4.05; 95% confidence interval [CI95], 1.20-13.72). Prior use of PPI was marginally associated with complications (aOR, 3.54; CI95, 1.00-12.48).
**Conclusion:** A considerable proportion (12.2%) of CDI was CO-CDI and 15.0% of them had poor outcome. Clinicians should be aware of the possibility of CDI when an elderly patient with diarrhea has prior antibiotic agent use within 1 month, despite suspicion of community-onset infection.
**Disclosure of Interest**
None Declared.

### P33

#### Factors associated with increased risk of adverse outcomes in patients with toxigenic clostridium difficile infection: preliminary data from the Hungarian national nosocomial surveillance system

Agnes Hajdu, Judit Pászti, Zsuzsanna Barna, Éva Popovics, Virág Lesinszki, Andrea Kurcz
National Center for Epidemiology, Budapest, Hungary
**Correspondence:** Agnes Hajdu
*Antimicrobial Resistance and Infection Control* 2017, **6(Suppl 3):**P33

**Introduction:** *Clostridium difficile* infection (CDI) is a notable clinical and patient safety concern for hospitals in Hungary.
**Objectives:** We aimed to identify epidemiological and microbiological factors associated with increased risk of adverse outcomes in CDI patients in hospitals to inform clinical and infection control practice.
**Methods:** We analysed 4-month case-based mandatory CDI surveillance data from the Hungarian National Nosocomial Surveillance System reported by 53 hospitals that also participated in an enhanced microbiological surveillance in February and October-December 2016. Variables included detailed demographic and clinical data. Toxigenic *C. difficile* isolates were sent for typing to the national reference laboratory. Ribotypes (RT) were identified by conventional PCR ribotyping. Presence of binary toxin genes was detected by PCR. To explore associations between various factors and adverse outcomes (in-hospital case fatality; severe case – surgery, intensive therapy or death due to CDI), we performed univariate analyses and multiple logistic regressions in STATA.
**Results:** Overall 291 CDI cases were analysed. The following factors were found to be associated with an increased risk of in-hospital case fatality in univariate analysis: male sex, rapidly (<1 year) and ultimately (<5 year) fatal underlying illness (McCabe scores), healthcare-associated origin, outbreak case, chronic kidney disease (CKD), binary toxin gene positivity. Among these, the unfavourable McCabe scores were independent predictors of death. The following factors were found to be associated with an increased risk of being a severe case in univariate analysis: rapidly fatal underlying illness, immunosuppressed status, CKD, malnutrition, RT027, binary toxin gene positivity. Among these, CKD and malnutrition were independent predictors of severity.
**Conclusion:** Distinguished therapeutical and infection control approach should be devoted to the most vulnerable patients suffering from CDI to prevent adverse outcomes.
**Disclosure of Interest**
None Declared

### P34

#### Prevalence of clostridium difficile infection in IBD patients

Chetana Vaishnavi, Rakesh Kochhar
Gastroenterology, Post Graduate Institute of Medical Education and Research, Chandigarh, India
**Correspondence:** Chetana Vaishnavi
*Antimicrobial Resistance and Infection Control* 2017, **6(Suppl 3):**P34

**Introduction:** The prevalence of *Clostridium difficile* infection (CDI) in inflammatory bowel disease (IBD) has become a focus of increased attention. As the *C. difficile* epidemic continues to grow, overlapping clinical presentations of CDI and IBD pose barriers to diagnosis and treatment.
**Objectives:** We retrospectively investigated the prevalence of CDI in IBD patients and analysed their demographic and clinical profile.
**Methods:** During analysis, the IBD patients (n = 721; M:F = 1.10:1) comprised of test group and an equal number of gender-matched patients with no indication of IBD was included as non-IBD controls. The demographic and clinical data as well as fecal *C. difficile* toxin status of all the patients were retrieved from laboratory records.
**Results:** The number of *C. difficile* positivity was more in the non-IBD group (20%) compared to IBD patients (16%) but the difference was not significant ($p < 0.064$). The patients in non-IBD group were relatively older ($p < 0.001$) compared to those in IBD group. The duration of diarrhea in the IBD group was significantly longer ($p < 0.001$) compared to the non-IBD group, but there was no significant difference ($p > 0.063$) in the frequency of diarrhea between the two groups. Blood in stool and abdominal pain symptoms were found to be significantly higher ($p < 0.001$) among the IBD group whereas watery diarrhea and fever were significantly associated ($p < 0.001$) with non-IBD group. There was significant improvement in most of the parameters during the time of follow up.
**Conclusion:** Though the prevalence of CDI in both IBD and non-IBD groups were almost similar, clinical symptom and age of presentation varied in them.
**Disclosure of Interest**
None Declared.

### P35

#### Control of a rotavirus gastroenteritis outbreak in three psychogeriatric units

Myriam Gyger Wanzenried[1], Christiane Petignat[2], Immaculée Nahimana Tessemo[2]
[1]Département de psychiatrie, CENTRE HOSPITALIER UNIVERSITAIRE VAUDOIS, PRILLY; [2]Unité Cantonale HPCI, CENTRE HOSPITALIER UNIVERSITAIRE VAUDOIS, Lausanne, Switzerland
**Correspondence:** Myriam Gyger Wanzenried
*Antimicrobial Resistance and Infection Control* 2017, **6(Suppl 3):**P35

**Introduction:** Rotavirus gastroenteritis, common in paediatric population is underestimated in adult. Even if the majority of non bacterial outbreak gastroenteritis is due to *norovirus*, the part of rotavirus is probably undervalued. Control of gastroenteritis is more complicated when the characteristics of patients make the implementation of recommendations in infection control difficult. We describe the rotavirus impact on the ward organization during an outbreak of gastroenteritis in the department of psychiatry of the Centre hospitalier universitaire vaudois of Lausanne.
**Objectives:** To manage a gastroenteritis outbreak in a psychiatric unit and to evaluate the impact of the ward organization.
**Methods:** We collected data from patients and staff data members in epidemic period of gastroenteritis. Stools of symptomatic patients were sending to the laboratory for screening of *norovirus* and *rotavirus*. Additional measures to Standard Precautions and specials measures (eg : closing units to news admissions; postponing transfers of patients; management of staff members) were implemented.
**Results:** From April 9$^{th}$ to 6$^{th}$ of May 2016, 17/31 patients and 10/78 staff members were affected with respectively an attack rate of 55% and 13%. Among patients, 88% (15/17) presented diarrhea first and that were associated with vomiting in 41% (7/17). The median duration of the disease in patients was 4 days (range; 1 to 12 days). 8/9 samples of stool screened were positives to *rotavirus* and negatives to *norovirus*. Three units were closed eleven days additionally to Measures of infection control.
**Conclusion:** Control of rotavirus gastroenteritis remains a challenge in psychiatric facilities. The prevention involves routine and terminal cleaning of the patient environment and a particular attention in communication and coordination of different teams.
**Disclosure of Interest**
None Declared.

## Influenza

### P36

#### Prevention of Nosocomial Influenza (NOSO-INF): visitors as key partners?

Anne Iten[1], Chantal Bonfillon[2], Claire-Anne Siegrist[3], Laurent Kaiser[4], Didier Pittet[1]
[1]Infection Control Program; [2]Hospital Health Service; [3]Department of Child and Adolescent Medicine; [4]Laboratory of Virology, HUG, Genève, Switzerland
**Correspondence:** Anne Iten
*Antimicrobial Resistance and Infection Control* 2017, **6(Suppl 3):**P36

**Introduction:** Vaccination is the cornerstone of the prevention against seasonal influenza (SI). In countries where vaccination cannot be made mandatory among healthcare workers (HCWs), the alternative strategy for hospitals to prevent NOSO-INF during SI epidemics combine the obligation for healthcare workers (HCW) to be vaccinated or to wear a mask, as well as recommendations for visitors to wear a mask, in addition to the promotion of hand hygiene. This is the strategy (called "zoning") adopted by HUG since 2009.
**Objectives:** We describe SI epidemics (inclusive NOSO-INF) and compliance with recommendations at HUG from winter 2011/12 to winter 2015/2016.
**Methods:** Suspected cases of SI (respiratory symptoms, fever with chills, muscular pain, or prostration) were screened using nasopharyngeal samples and confirmed by RT-PCR. Cases were defined as NOSO-INF when symptoms occurred >72 h after admission. Regular audits were performed to assess HCWs and visitors' compliance with recommendations. HCWs were regularly informed about the evolution of SI epidemics and results of the audits.
**Results:** Between 2011/12 and 2015/16, a total of 1432 patients (min = 148 in 2011/12; max = 471 in 2011/12) tested positive for influenza. Droplet precautions with single room isolation whenever possible were implemented for 1245 patients (86.9%); in 2011/12, droplet precautions were installed for only 73/148 patients (49.3%). The vaccination rate of hospital staff increased 12% (2011/12 29%; 2015/16 37%) and HCWs' compliance with recommendations (vaccine or wearing of a mask during SI epidemics) increased 24% (2011/12 55%; 20015/16 79%). During 2015/16, only 11% of the visitors wore a mask while in wards. While the overall incidence of NOSO-INF declined by 31% over the study period, it was still 24% in 2015/16.

**Conclusion:** The combination of HCW vaccination and infection control measures (hand hygiene, droplet precautions and single room when available) for patients and HCWs is not sufficient to prevent nosocomial transmission of the influenza virus. Mandatory masking for visitors during SI should be considered as a necessary additional measure.

**Disclosure of Interest**

None Declared.

### P37

#### Healthcare workers' perception towards the systematic use of mask during a seasonal influenza outbreak in a French University Hospital: a descriptive study

Matthieu Beuvelet[1], Claire Masson[2], Deborah Gantz[2], Sylvie Levet[2], Benoit Allenet[1], Marie Reine Mallaret[2, 3], Caroline Landelle[2,3]
[1]Pharmacy department; [2]Infection control unit, CHU Grenoble Alpes; [3]ThEMAS TIM-C UMR 5525, University Grenoble Alpes/CNRS, Grenoble, France
**Correspondence:** Caroline Landelle
*Antimicrobial Resistance and Infection Control* 2017, **6(Suppl 3):**P37

**Introduction:** Systematic use of mask was recommended for healthcare workers (HCWs) of our institution during the 2016/2017 influenza outbreak to prevent respiratory virus transmission and nosocomial influenza.

**Objectives:** To evaluate HCWs' perception towards the systematic use of mask in a French University hospital.

**Methods:** Voluntary HCWs (physician, nurse, nursing assistant, physiotherapist and others) of 16 emergency, internal medicine, infectious diseases, geriatric, pediatric and gynecology units were included in the perception survey; one investigator collected data by using a pre-tested questionnaire of 11 items. An infection control nurse observed compliance with use of mask for all HCWs of the same units.

**Results:** Between January and February 2017, 177 HCWs were included (mean of 11 HCWs/units); 102 (58%) were not used to wear a mask more than once per month. Overall, 169 (96%) reported to comply with use of mask during the influenza outbreak. Between December and March 2017, observed compliance with use of mask was 82% (2008/2461). Regarding daily care of patients, 43 (25%) and 26 (14%) declared that use of mask changed their practices and decreased quality, respectively. Comfort was decreased for 142 (80%) and 123 (70%) had trouble communicating with patients. Wearing a mask was an effective mean to protect patients for 158 (89%), themselves for 142 (80%), their coworkers for 133 (75%) and their family for 107 (61%). A total of 33 (19%) considered not to be enough informed about the interest of wearing a mask and 155 (88%) were convinced by the usefulness of this measure.

**Conclusion:** A majority of HCWs is convinced by the interest of wearing a mask during influenza outbreak to prevent influenza transmission and follows this practice. However, wearing a mask is often uncomfortable and modifies daily care of patients for one-quarter of HCWs. Following this survey, we plan several sessions of information for the next influenza outbreak.

**Disclosure of Interest**

None Declared

### P38

#### Notification of healthcare-associated influenza in a French region: what is the trend since 2012

Florence STORDEUR, Léa HOISNARD, Elise SERINGE, Pascal ASTAGNEAU
CPias Ile-de-France, Paris, France
**Correspondence:** Florence STORDEUR
*Antimicrobial Resistance and Infection Control* 2017, **6(Suppl 3):**P38

**Introduction:** Several networks in France are in charge of the surveillance of community acquired influenza. Nevertheless, hospital's data exist. As healthcare-associated infections (HCAI), cases contracted in hospital have to be notified since 2001.

**Objectives:** The aim of the study was to analyze and compare to community data HCAI notifications during the five flu-seasons from 2012 to 2017 in a French region.

**Methods:** Data were collected from the electronic tool used since 2012 to notify HCAI. Notifications were included if the first case of HCAI had occurred between the 1st october and the 31st march. As the involved microorganism was not systematically known, selection criteria were multiple: the anatomical site ("top"- "bottom"- "unknown"- "others" respiratory infection), the microorganism ("influenza" and "others" virus), the linked nature ("influenza outbreak" and "influenza-like illness"), and "influenza" as free text. In addition, community data collected from a general physician network (French Sentinelles network) were analyzed.

**Results:** A total of 97 notifications were analyzed, respectively: 10, 14, 32, 10 and 31 from 2012 to 2017, representing a total of 1106 cases (respectively 135, 169, 315, 135 and 352). The healthcare outbreak peak occurred simultaneously with influenza community transmission for the same region, except for the season 2016-2017 for which hospital was concerned 3 weeks before community. About 90% of the notifications were outbreak episodes. The six sporadic cases concerned severe flu. Case-fatality rate reported in the notifications was respectively 0.3%, 0.6%, 0.6%, 1.5% and 2.2%. For the whole seasons, cases were mainly reported in long-term dependency care (29.5%), rehabilitation (27.0%), medicine (21.0%) and long-term care (20.3%) wards. Notifications were done around 25 days after the identification of the first case. Episodes included at least one infected health care professional in 50.0%, 57.1%, 53.1%, 60.0% and 22.6% respectively.

**Conclusion:** The outbreak of healthcare-associated influenza is strongly linked to the community transmission. The strenghtening of standard precautions and the vaccination of healthcare professionals and high-risk patients are particularly essential during the epidemic period.

**Disclosure of Interest**

None Declared.

### P39

#### Leadership commitment – a core strategy to improve influenza vaccination coverage among healthcare workers

Merete E. Gjerde, Kristin S. Kilhus
Department of Research and Development, Division of Patient safety and Infection Control, Haukeland University Hospital, Bergen, Norway
**Correspondence:** Merete E. Gjerde
*Antimicrobial Resistance and Infection Control* 2017, **6(Suppl 3):**P39

**Introduction:** Annual influenza vaccination of healthcare workers (HCWs) is considered an important intervention to prevent transmission of influenza in health care settings. We describe a strategy where leadership involvement improved flu vaccination among HCWs in a Norwegian University Hospital with a total of 12 000 employees.

**Objectives:** Increased influenza vaccination coverage among HCWs.
**Methods:** For 18 years seasonal influenza vaccine has been offered to employees free of charge and measures to improve vaccination have been targeting clinical staff. In 2015 the hospital senior management and infection control team introduced a new infection control strategy where the main objective was establishing a more systematic approach to infection control measures. The aim was to empower local leaders to take responsibility and put infection control on the daily agenda. The infection control team offered to facilitate departments in their work, and created a toolkit making it easier for local managers to set achievable goals. Improving influenza vaccination coverage among HCWs was one of five hospital-wide topics of priority. Measures to improve vaccination coverage are management commitment, education, vaccination availability, communication strategies and comparing results within the hospital. The Pharmacy provides statistics of purchased vaccines for each department. An overview is published on the hospital intranet and department managers are responsible to register administered vaccines.
**Results:** The average number of purchased vaccines was 1780 from 2006-2012. From 2012-2014 the average was 3033, most likely due to a local campaign. After introducing the new infection control strategy a total of 4469 vaccines were purchased in 2016, a 60% increase compared to earlier seasons.
**Conclusion:** Introducing an infection control strategy ensuring management commitment seems to have had effect. We are satisfied with the preliminary improvement in influenza vaccination coverage among HCWs in our hospital. We now focus on the sustainability of the improvement, and are ambitious to achieve even better results.
**Disclosure of Interest**
None Declared

### P40

#### Comparison between routine and modified scales in predicting influenza a (H1N1) pneumonia's mortality

Ali Alrstom
INTERNAL MEDICINE, DAMASCUS UNIVERSITY, DAMASCUS, Syrian Arab Republic
*Antimicrobial Resistance and Infection Control* 2017, **6(Suppl 3):**P40

**Introduction:** Current pneumonia prediction scales may underestimate the severity of influenza pneumonia.
**Objectives:** This research study aimed to analyze routine and modified scales' usefulness in predicting 28-day mortality.
**Methods:** Prospective cohort study included 145 patients with community acquired pneumonia (CAP); 48 of patients were with flu-like illness, 25 of them were (H1N1) RT-PCR positive. Routine CAP severity scores Pneumonia Severity Index (PSI) and CRB-65 (confusion, respiratory rate, blood pressure, age $\geq$ 65 years) were calculated; in addition, a modified and stratified scale was calculated by adding underlying disease 'D' defined according to (PSI) rule, peripheral oxygen saturation 'S' with stratification compatible with severity of hypoxemia, modifying the cutoff values of systolic blood pressure and respiratory rate according to quick Sequential Organ Failure Assessment (q-sofa) score and stratifying the age. These modifications led to DS-CRB-Age scale: underlying disease, peripheral oxygen saturation (One point if SaO2 81% - 89%, two points if SaO2 $\leq$ 80%), confusion, respiratory rate $\geq$ 22, systolic blood pressure $\leq$ 100 mm-Hg or diastolic blood pressure $\leq$ 60 mm-Hg, Age (one point if $\geq$ 65 years, two points if $\geq$ 75 years). The area under the receiver operating characteristic curves (AUC) was used to compare the discriminative power of the severity scales. Serum creatinine phosphokinase (CPK) level was measured for each patient.
**Results:** The patients with flu-like illness were younger than patients with CAP other than influenza. The low-risk influenza pneumonia actual mortality was (PSI, CRB-65, DS-CRB-Age: 44.44%, 46.2%, 38% respectively) which was significantly higher than what reference predicted by PSI, CRB-65 (0.9%, 1.2% respectively). Of note, the AUC of DS-CRB-Age scale for patients with CAP other than influenza pneumonia was 0.78 (95% CI: 0.675-0.859), similar to that of PSI 0.77 (95% CI: 0.662-0.846). The positive likelihood ratio of CPK elevation above 1000 U/L for positively (H1N1) RT-PCR was 11.
**Conclusion:** PSI, CRB-65 and DS-CRB-Age scales are all weak prognostic scoring systems in predicting 28-day mortality in influenza pneumonia. Further studies are needed in patients with influenza pneumonia to find a better prognostic system.
**Disclosure of Interest**
None Declared.

### P41

#### Characterisation of nosocomial and community-acquired influenza in a French University Hospital during influenza season 2015/2016: a prospective study

Alexis Bocquet[1], Claire Wintenberger[1], Julien Lupo[2], Christine Morel-Baccard[2], Patrice Morand[2], Patricia Pavese[3], Laurence Bouillet[1], Jean Luc Bosson[4], Marie Reine Mallaret[4,5], Caroline Landelle[4,5]
[1]Internal Medicine Department; [2]Virology Laboratory; [3]Infectious diseases unit, CHU Grenoble Alpes; [4]ThEMAS TIM-C UMR 5525, University Grenoble Alpes/CNRS; [5]Infection control unit, CHU Grenoble Alpes, Grenoble, France
**Correspondence:** Caroline Landelle
*Antimicrobial Resistance and Infection Control* 2017, **6(Suppl 3):**P41

**Introduction:** Influenza is increasingly recognized as an important public health threat causing considerable morbidity and mortality each year.
**Objectives:** To describe the influenza outbreak in our hospital during influenza season 2015/2016.
**Methods:** A prospective observational study was conducted in a 2200 bed University Hospital. All hospitalized patients with influenza-like illness associated with laboratory-confirmation by RT-PCR were included. Nosocomial influenza was defined by a time interval between admission and symptoms' onset >72 hours. Severe influenza was defined by admission into an intensive care unit.
**Results:** A total of 233 cases of influenza occurred, 178 adults and 55 children. Among 178 adults, 138 (78%) had at least one risk factor of complication and 61 (44%) were not vaccinated. Influenza A and Influenza B were diagnosed for 86 (48%) and 92 (52%) adults, respectively. A total of 22 (12%) adults developed severe influenza; 15 (68%) had Influenza A and 7 (32%) Influenza B. Among 55 children, Influenza A and Influenza B were diagnosed for 19 (35%) and 35 (64%) children, respectively. A total of 20 (36%) children developed severe influenza; 8 (40%) had Influenza A and 11 (55%) Influenza B. A total of 44 (19%) nosocomial-acquired influenza were identified; 38 (21%) adults and 6 (11%) children. Patients with nosocomial-acquired influenza were significantly older (median age 77y *vs* 67y), had more risk factors of complication (90% *vs* 74%), were hospitalized more frequently in double or multi-occupancy room (77% *vs* 54%) and droplet precautions were less applied (62% *vs* 82%) than the community-acquired influenza group.
**Conclusion:** These results highlight the necessity of a better education about droplet precaution and patient isolation. Moreover, a public policy vaccination for healthcare workers and patients should be encouraged to prevent nosocomial influenza infection.
**Disclosure of Interest**
None Declared

### P42

#### Influenza preventive measures: campaigns to promote vaccination and surgical mask among health-care workers

Léa HOISNARD, Elise SERINGE, Karin LEBASCLE, Pascal ASTAGNEAU
CPias Ile-de-France, Paris, France
**Correspondence:** Léa HOISNARD
*Antimicrobial Resistance and Infection Control* 2017, **6(Suppl 3):**P42

**Introduction:** The prevention of health-care associated infections is an important goal in all health care facilities (HCF). Influenza well-known preventive measures are vaccination and the use of surgical masks.

**Objectives:** This study estimates vaccination coverage among health care workers and assesses campaigns organized to promote these methods in 2017. This study has been conducted every flu season for 3 years.
**Methods:** A descriptive survey was conducted during the 2016-2017 flu epidemic. Data from HCF were collected thanks to an online platform. Items about vaccination rate and campaigns promoting mask use and vaccination were filled by Infection Control Team (ICT) in 2 French regions. Evolution of vaccination coverage was assessed for HCF that participated during the last 3 years.
**Results:** A total of 191 HCF (25%) participated. The overall influenza vaccination coverage ($n = 177$) was 20.4% [18.1 - 22.8] including 38.0% [33.8 - 42.2] ($n = 158$) for doctors and midwives, and 19.6% [16.8 – 22.4] ($n = 167$) for nurses and nursing assistants. The overall coverage increased in 36% of HCF between 2016 and 2017. Most of the HCF (93%) organized an information campaign and carried out a vaccination campaign. The organization was shared between Occupational health team (OHT) (21%), ICT (26%), and the hospital pharmacy (20%). Most healthcare workers (HCW) were vaccinated by their colleagues and OHT. Posters advising wearing a mask were present in 86% of HCF, mainly at the hospital entrance. Masks were available in the emergency, maternity and pediatric wards. Among participants, 44 HCF (23%) reported a flu outbreak, of which 45% involved long term care wards, and 73% used prophylaxis treatment for contacts (73%). This season, 33 HCF (17%) reported flu cases among HCW, either during cluster of case-patients or sporadic cases. A subset of 74 (39%) HCF has been answering for 3 seasons. No difference was found between season for overall vaccination coverage nor in each HCW category.
**Conclusion:** Despite information and vaccination campaigns, assessed during three years, the overall vaccination among HCW remains below expectation, about 20%. More effective measures such as behavioral methods are needed to reinforce prevention campaigns and enhance information level and awareness of patients and HCW.
**Disclosure of Interest**
None Declared.

### P43
### Frequency, evolution, clinical and epidemiological characteristics of severe nosocomial influenza cases in the Valencian Community. 2010/2011 -2014/2015

Juan G. Mora Muriel, Ginger G. Cabrera Tejada, Patricia Garcia Shimizu, Cesar O. Villanueva Ruiz, Marina Fuster Perez, Marina D. C. Lopez-Bajoz Mesa, Carmen M. Benito Miralles, Jose Sanchez Payá
Unidad de Epidemiología, Servicio de Medicina Preventiva, Hospital General Universitario de Alicante, Instituto de Investigación Sanitaria y Biomédica de Alicante (ISABIAL - Fundación FISABIO), Alicante, Spain
**Correspondence:** Juan G. Mora Muriel
*Antimicrobial Resistance and Infection Control* 2017, **6(Suppl 3):**P43

**Introduction:** A major public health challenge, influenza represents a special hazard inside health facilities.
**Objectives:** The purpouse of this study is to know the frequency, evolution and clinical-epidemiological characteristics of severe nosocomial influenza cases (SNIC) in the Valencian Community from 2010/2011 to 2014/2015.
**Methods:** Observational study of SNIC during 2010/11 to 2014/15 influenza seasons based on the data obtained from the epidemiological surveillance network (AVE) of hospitals in the Valencian Community. The severe influenza cases (SIC) definition established in the Epidemiological Surveillance System was used. As explanatory variables were used those collected in the epidemiological survey of SIC. A SIC was considered from nosocomial origin (SNIC) when three or more days elapsed from the day of admission to the center and the day of onset of the symptomatology. The percentage of SNIC with its 95% confidence intervals was calculated, the evolution in time was studied using the Chi square test for trends; for the magnitude association study between the different characteristics of the patients and the SNIC the Odds Ratio (OR) was calculated with their 95% confidence intervals (95% CI).
**Results:** A total of 1202 patients were included in the study; 2.7% were SNIC. As of 2010, there was a slight decrease of the SNIC with a minimum value in the 2012/13 season of 1.7% compared to a 5.7% reached in the 2010/11 season. The observed changes in the SNIC were not statistically significant. In the analysis of factors associated with SNIC, when adjusting the multivariate model only the antecedent of cancer ORa = 3,4 (1,2-9,2), the presence of anemia ORa = 3.0 (1-9.14) and the presence of myalgia ORa = 0.25 (0.07-0.91) were independently associated.
**Conclusion:** The frecuency of severe nosocomial influenza cases in the Valencian Community is low and there has been no major changes in the evolution during the seasons 2010/11 to 2014/15. The characteristics of the patients that are independently associated to being SNIC are: antecedent of cancer, the presence of anemia and the presence of myalgia.
**Disclosure of Interest**
None Declared.

### P44
### Withdrawn

### P45
### Potential genetic polymorphism of PB2-701 and 702: implication in virus-host interaction of influenza a virus

Alex Wing Hong Chin, Nathaniel Keng Chon Leong, Leo Lit Man Poon
School of Public Health, THE UNIVERSITY OF HONG KONG, Pokfulam, Hong Kong
**Correspondence:** Alex Wing Hong Chin
*Antimicrobial Resistance and Infection Control* 2017, **6(Suppl 3):**P45

**Introduction:** PB2-701 of influenza A virus is a genetic marker for host adaptation. A D701N mutation of an avian virus can enhance the polymerase activity and viral replication in mammalian cells. PB2-702 shows host specificity, with most human viruses carry an arginine and most avian viruses carry a lysine. However, limited polymorphisms at these two residues are found in the natural isolates, limiting the study of their role in the polymerase.
**Objectives:** We aim to investigate the potential genetic polymorphism of the PB2-701 and 702 residues by site-directed random mutagenesis of the PB2 gene of the virus.
**Methods:** To elucidate the role of PB2-701/702 in viral fitness, site-directed random mutagenesis of the PB2 gene was performed to generate recombinant viruses with random mutations at PB2-701/702 in mammalian and avian cells. The polymerase activity, viral replication and pathogenicity of the mutant viruses generated were characterized.
**Results:** A wide range of viruses with different PB2-701/702 mutations were isolated. Several mutants demonstrated enhanced polymerase activity in mammalian cells and comparable viral replication and pathogenicity in mice when compared to the wild-type virus. Surface electrostatic charge prediction on the PB2 structural model revealed that the polymerase activity in mammalian cells generally increases as the surface of the PB2-700–703 region becomes more positively charged. On the other hand, some mutants had reduced polymerase activity and viral replication in mammalian cells. One of them (701A/702E) also had lower pathogenicity in mice. Distinct tissue tropism of the PB2-701/702 mutants was observed in chicken embryos. It was found that importin-α4 has a role in the reduction of the polymerase activity in some mutants. Knocking-down the importin-α4 of the mammalian cells enhanced the polymerase activity of the mutants with low polymerase activity, while the importin-α4 inhibitory effect is not significant in other mutants with high polymerase activity.
**Conclusion:** Overall, this study demonstrated the potential genetic polymorphisms at PB2-701/702 and revealed the potential role of importin-α4 in modulating the polymerase activity. The findings in this study may lead to the further study of the role of PB2-701/702 in virus-host interaction and contribute to the control of influenza.

**Disclosure of Interest**
None Declared

### P46

#### A retrospective analysis of spreading 2014/2015 influenza and 2015/2016 influenza infections in our facility

Hiroko Shigemi[1], Kazuhiro Itoh[1], Yoko Muroi[2], Katsunori Tai[3], Yumiko Fujitomo[4], Naohisa Fujita[4], Takanori Ueda[1], Takahiro Yamauchi[1], Hiromichi Iwasaki[3]
[1]Hematology and Oncology; [2]Control and Prevention of Infectious Diseases; [3]Control and Prevention of Infectious Diseases, University of Fukui, Faculty of Medical Science, Fukui; [4]Control and Prevention of Infectious Diseases, University of Kyoto Prefectural of Medicine, Faculty of Medical Science, Kyoto, Japan
**Correspondence:** Hiroko Shigemi
*Antimicrobial Resistance and Infection Control* 2017, **6(Suppl 3):**P46

**Introduction:** Influenza presents a life-threatening infection for hospitalized patients, who might be nosocomially infected via healthcare workers (HCWs).
Especially the patients with a weakened immune system should take themselves intensively. It is necessary to look through the intermediary kind of occupation in hospital. Our facility is located in the suburbs and more than 20 km-distance from the center of the city.
**Objectives:** To certificate whether the incidences of influenza spreading in our hospital are related with the occurrence in the city, it is necessary to compare the mortality rate of influenza in our hospital with that of in the city retrospectively.
**Methods:** 1) Examinations to detect infection were performed in all cases and analyzed the periods, the type of occupation and the route of infection in 2015/2016 influenza.
2) Influenza peaks were examined in the report by Official Infectious Disease Surveillance Center in both 2014/2015 and 2015/2016 influenza. Incidences for patients and HCWs were calculated and possible epidemiological links were analyzed.
**Results:** Thirty patients and 98 HCWs were affected, including infected 11 HCWs in operation regions within 1 week. In HCWs, the rate of nurses was 44%, that of doctors 18% and that of clerks 9%. Influenza peak was shown at Jan.24 in 2014/2015 and bimodal peaks at Feb.3$^{rd}$ and Feb 23$^{rd}$ in 2015/2016. In 2014/2015 season, the high incident rate decreased until the first of Feb. Compared to Surveillance Data in Official Records, the peaks of influenza in patients and HCWs were the same in both years. Transmission from HCWs to patients was not possibly supposed in our facility.
**Conclusion:** Epidemiological official records have indicated the same results as prevalence in our hospital. Besides a higher vaccination rate in our facility and standard precaution, recognizing the information from the Surveillance Center is very useful to prevent outbreaks. We have no COI to declare.
**Disclosure of Interest**
None Declared

## Carbapenemase Producing *Enterobactericeae* (CPE)

### P47

#### Computer application for early identification and control of first cases of carbapenemase - producing Enterobacteriaceae in Seville - Andalusia

Encarnacion Roman, Carmen Escassi, Raquel Valencia, Jose María Pérez, Salvador Vergara, Carmen Castro, Jose Antonio Mira, Maria José Pérez
Servicio Andaluz de Salud, Seville, Spain
**Correspondence:** Maria José Pérez
*Antimicrobial Resistance and Infection Control* 2017, **6(Suppl 3):**P47

**Introduction:** The transmission of multi-drug-resistant microorganisms (MDRM) is a public health problem, to limit antimicrobial treatment options and its ability to spread. This is especially worrisome for carbapenemase-producing Enterobacteriaceae (CPE) for their difficult therapeutic management and high mortality rates. Early detection is essential to break transmission and prevent its spread.
At the end of 2015, an automatic alert system for the early identification of patients affected by these MDRMs was designed in our hospital. In March 2016, one case of CPE infection was identified in Seville. Until then, no cases with this genotypic profile had been recorded. This case was later transferred to another hospital in Seville.
**Objectives:** To describe the management of the first cases of CPE in Seville
**Methods:** Strict adherence to infection control measures and epidemiological investigation of cases, including identification of contacts, search of environmental reservoirs and review of cleaning and disinfection of medical devices were monitored. The strains of the affected patients were sent to the reference microbiological laboratory in Andalusia. "Alerts" were recorded on digital medical records of cases and contacts to detect early readmissions.
**Results:** There were 5 cases (3 infected and 2 colonized), involving three hospitals in Seville for transfer of patients. The identified strains of all patients were identical (KPC-3 producing clone ST512). 128 contacts were identified and "alert" in their history record. 21 contacts were readmissioned and all colonization studies were negative. Environmental and medical devices samples were also negative.
**Conclusion:** Cross-transmission, through the hands of healthcare professionals, has been the most probably cause of secondary cases in the province. Strict control measures proved effective in controlling the situation. The automatic alert system has enabled the contacts to be identified early to prevent transmission of the MDRM.
**Disclosure of Interest**
None Declared.

### P48

#### Intensified infection control measures were effective to reduce the circulation of carbapenem-resistant klebsiella pneumoniae in intensive care units at a large Italian Hospital

Cristiano Alicino[1,2], Daniele R. Giacobbe[1,3], Cecilia Trucchi[1], Federico Grammatico[1], Matteo Astengo[1,2], Angela Battistini[2], Anna Maria Di Bella[2], Barbara Guglielmi[2], Valerio Del Bono[3], Angelo Gratarola[4], Paolo P. Pelosi[4], Filippo Ansaldi[1], Andrea Orsi[1,2]
[1]Department of Health Sciences, University of Genoa; [2]Hygiene Unit; [3]Infectious Diseases Unit; [4]Intensive Care Unit, I.R.C.C.S. University Hospital San Martino - IST National Institute for Cancer Research, Genoa, Italy
**Correspondence:** Cristiano Alicino
*Antimicrobial Resistance and Infection Control* 2017, **6(Suppl 3):**P48

**Introduction:** In recent years, carbapenem-resistant *Klebsiella pneumoniae* (CR-Kp) has become endemic in Italy.
**Objectives:** A quasi-experimental study was conducted to assess the effectiveness of intensified infection control measures, including active surveillance of colonized patients through rectal swab, on reducing the circulation of CR-Kp in the intensive care units (ICUs, nearly 40 beds) of the tertiary 1,300 acute-care beds regional referral center university hospital, I.R.C.C.S. AOU San Martino-IST of Genoa, Northern Italy.
**Methods:** The intervention effect was analyzed with interrupted time series regression analysis. The study included a pre-intervention period (January 2009 - December 2011) and an intervention period (January 2013 - December 2016). During 2012, routine rectal screening was implemented and reached high level of adherence. Monthly incidence of first positive culture result for CR-Kp, either including or excluding rectal swab, and incidence of first isolation from blood, urine, respiratory specimens and samples from surgical site were compared.
**Results:** After the implementation of the intervention, the incidence of first positive culture results for CR-Kp significantly decreased, both including (slope: -0.15; $p$-value: 0.03) and excluding rectal swabs (slope: -0.13; $p$-value: 0.02). A significant reduction in the incidence of CR-Kp has been demonstrated also for samples from surgical site (slope: – 0.06, $p$-value < 0.001) and lower respiratory tract (slope: -0.09, $p$-value = 0.05). A decreasing trend, even though not statistically significant, was observed for the incidence of isolation of CR-Kp from blood (slope: -0.06; *p*-value: 0.1) and urine (slope: -0.03; *p*-value: 0.16).

**Conclusion:** Intensified infection control measures, including routine rectal surveillance swabs, resulted effective in reducing the circulation of CR-Kp in the ICUs. However, more efforts are needed to prevent bloodstream infections sustained by this microorganism.

**Disclosure of Interest**

None Declared.

### P49

#### Control of KPC-producing Klebsilla pneumonia using multi-prong approach in surgical discipline ward

Li Xin Gillian Lee, Moi Lin Ling, Kwee Yuen Tan, Jia Hua Seah, Bushra Binte Shaik Ismail

Singapore General Hospital, Singapore, Singapore

**Correspondence:** Li Xin Gillian Lee

*Antimicrobial Resistance and Infection Control* 2017, **6(Suppl 3):**P49

**Introduction:** Klebsiella pneumoniae carbapenemase (KPC)-producing bacteria are a group of highly drug-resistant Gram-negative bacilli causing infections associated with significant morbidity and mortality. In recent years, Klebsiella have become a common pathogen causing nosocomial infections. KPC can spread rapidly within healthcare facilities, especially in long-term acute care hospitalshospitals

**Objectives:** To break the chain of transmission of CP-CRE in a surgical ward in a 1750-bedded tertiary hospital

**Methods:** CP-CRE patients were promptly isolated upon receiving positive results from the lab. Daily audit on personal protective equipment (PPE) and hand hygiene was conducted. Immediate feedback was given to healthcare workers and senior management was informed on non-compliance on a daily basis. Environmental cleaning was increased to twice a day with Sodium hypochlorite 1000 ppm. During the outbreak, environmental samplings were conducted. Sinks with positive cultures were scrubbed using sodium hypochlorite 1000 ppm. All affected rooms were disinfected with Hydrogen Peroxide Vaporization (HPV).

**Results:** Daily hand hygiene and PPE audit compliance improved from 70% to 100%. Environmental compliance improved from 14.3% to 100%. The majority of environmental samplings from sinks conducted post hydrogen peroxide vaporization were negative. Sinks with positive results were further scrubbed with Sodium hypochlorite. There are no reported cases of CP-CRE since the outbreak closure.

**Conclusion:** Sink scrubbing and hydrogen peroxide vaporization of environment together with good infection control practices helped to break the chain of transmission of CP-CRE in the surgical wards.

**Disclosure of Interest**

None Declared.

### P50

#### Klebsiella Pneumoniae Carbapenemase (KPC)-3 outbreak: effecting change in infection prevention and control in a limited resource setting in Barbados

Corey A. Forde, Corey Forde, Bryan Stierman, Nalini Singh, Yvonne Martindale, Keisha Gustave,Marsha Burton

Infection Prevention and Control, Queen Elizabeth Hospital, Barbados, Bridgetown, Barbados

**Correspondence:** Corey A. Forde

*Antimicrobial Resistance and Infection Control* 2017, **6(Suppl 3):**P50

**Introduction:** CRKP outbreaks have occurred all over the world and carries a high morbidity and mortality. There is limited information in the english speaking Caribbean where counties have newly developing or no AMR programs.

**Objectives:** To define the burden of colonisation with CRKP in the major hospital and to demonstrate how the use of this information can drive change and foster development of an IPC program in low resources setting.

**Methods:** *Setting: The 600 bed* Queen Elizabeth Hospital (QEH) is the only public hospital and serves as the referral hospital for a population of 270,000.

*Study Design:*point-prevalence study of 26 wards, containing a total of 311 patients using rectal swabs were collected to determine colonization status in the hospital. This was followed by an in IPC interventional program.

*Statistics:*Data analyses were carried out with EpiInfo7.1.5 to determine associations between CRKP colonized patients and predictors.

**Results:** 18% of patients (53/299) were colonized/infected with CRKP,

Infected/colonized patients were older (64.7 vs. 48.7 years, $p < 0.0001$),

Infected/colonized patients had a longer length of stay (42.5 vs. 27 days, $p = 0.0042$,

Infected/colonized patients were more likely to have invasive devices (66% vs. 32%, $p < 0.0001$) and urinary catheters (55% vs. 24%, $p < 0.0001$)

*Intervention Strategies:*Hygiene audits were carried out prior to the estimation of burden of colonisation showing 20% compliance.A hand hygiene education program (STOP, THINK, GO) was set up with bimonthly audits & by 2016 compliance was 60%.Polices on urinary catheters with staff education, an antimicrobial stewardship program (ASP), screening (CRE/MRSA) of long term stay commenced.

**Conclusion:** Determining the burden of CRKP in the hospital has helped drive change in establishment of a robust IPC program with a new leadership structure.Strategies used in CRKP control from our experience can be used in other resource limited setting across the world.

Barbados lead in the wider regional in education & mentorship programs to improve IPC across the eastern Caribbean in conjunction with WHO/PAHO - IPC (ECC & WDC). The importance of international cooperation with agencies such as PAHO/WHO cannot be understated in a resource limited setting.

**Disclosure of Interest**

None Declared.

### P51

#### "Screen, isolate and report" strategy to control carbapenem resistant Enterobacteriaceae Hospital transmission at regional level in Poland

Pawel Grzesiowski[1], Maria Pawlak[2], Anna Tymoczko[3]

[1]Institute for Infection Prevention; [2]Chief Mazovian County Sanitary Inspectorate; [3]Epidemiology, Mazovian County Sanitary Inspectorate, Warsaw, Poland

**Correspondence:** Pawel Grzesiowski

*Antimicrobial Resistance and Infection Control* 2017, **6(Suppl 3):**P51

**Introduction:** In 2012 first isolates of NDM producing Klebsiella pneumoniae (KP-NDM) have been reported in Poland. After increase of KP-NDM frequency in 2015 in Warsaw and Masovian County, the expert group at the Masovian Sanitary Inspectorate has been asked to develop policy to control transmission of Carbapenem-resistant Enterobacteriaceae (CPE) in hospitals.

**Objectives:** The prospective study at the regional level has been undertaken to assess the effectiveness of the policy to control of KP-NDM spread in hospitals.

**Methods:** The policy "Screen, Isolate and Report" (SIR) has been developed based on ECDC and national guidelines on CPE prevention and control. The main interventions: 1)screening of the risk patients at admission time; 2) Carba-NP testing for rapid detection of CPE; 3) contact isolation precausions of colonized and infected patients; 4) weekly screening of contacts in hospitals; 5) monthly reporting to co-ordinating centre; 6) feedback from the coordinating centre to hospitals with KP-NDM transmission or outbreak.

**Results:** The SIR policy has been published on the web site and distribuited to all 171 hospitals through sanitary inspection network with 63% implementation rate during 2016. Before the policy introduction, in 2015, 410 cases of KP-NDM have been reported from 31 hospitals in comparison to 1360 cases reported from hospitals in 2016. Almost 50% of KP-NDM positive patients has been diagnosed in patients screened at the admission time with the overall incidence rate 1 per 1000 admissions. From 29.500 patients screened during hospitalization, 5% patients were positive for KP NDM, incuding 35% with symptomatic infection, most frequently, urinary tract infection. In 24 hospitals there were 50 episodes of inter-ward KP-NDM transmission, including 19 outbreaks of symptomatic infection. No increase in KP-NDM incidence has been observed during 2016.

**Conclusion:** Almost 75% of KP-NDM positive patients have been isolated according the policy, including 30% in single rooms with toilets. The hand hygiene in hospitals has improved, from 27 to 45 liters per 1000 patient-days. The SIR policy established at the regional level has been accepted in majority of hospitals with substantial effectiveness.

**Disclosure of Interest**

None Declared.

### P52

#### Evaluation of in vitro susceptibility of minocycline for carbapenem resistant Enterobacteriaceae (CRE)

Anup R. Warrier, Rachana Babu
Aster Medcity, Kochi, India
**Correspondence:** Anup R. Warrier
*Antimicrobial Resistance and Infection Control* 2017, **6(Suppl 3):**P52

**Introduction:** We are in a position where Multidrug resistant bugs are over ruling the healthcare settings with only limited availability of drugs effective against them. Considering the cost factor, side effects and prolonged hospital stay for the treatment of these bugs we are desperately in need of a drug that would help reduce the hospital stay and would be effective orally.

**Objectives:** 1. To evaluate the in vitro susceptibility of Minocycline in CRE

2. To compare the in vitro susceptibility of Minocycline against Tigecycline for the same isolates

**Methods:** Non-repetitive, consecutive 57 isolates of MDR Klebsiella pneumoniae species from all clinical samples were tested for susceptibility against Minocycline using E-test (supplied by BIOMERIOUX) that detects MIC range from 0.016 to 256 μg/ml. The MIC cut-off range for Minocycline is based on CLSI 2016 and the interpretation is as follows - Sensitive:<=4, Intermediate:8, Resistant:> = 16.

The Tigecycline MIC will be interpreted as per the EUCAST guidelines 2016. MIC range is as follows: sensitive (1), Resistant (4). The MIC for the same will be looked into as per the susceptibility pattern provided by the automated system BD PHOENIX/VITEK.

**Results:** The in vitro susceptibility of these isolates to Minocycline were as follows:

Klebsiella pneumoniae- 38.5% sensitive(22/57); 21.05% Intermediate(12/57); 40.3% Resistant(23/57).

The in-vitro susceptibility of Klebsiella pneumoniae to Tigecycline was as follows –8.7% sensitive (5/57); 24.5% Intermediate (14/57); 66.6% Resistant (38/57).

**Conclusion:** 1. Minocycline is a reasonble option for CRE but NOT as empiric therapy because of less than 50% susceptibility among tested isolates.

2. Minocycline has better susceptibility compared to Tigecycline and should be tested separately to be useful as a possible choice for CRE

**Disclosure of Interest**

None Declared

### P53

#### Identification & sensitivity pattern of bacterial isolates from low resource setting ICU, Rajshahi Medical College Hospital, Bangladesh

Abu Hena M. Kamal
ICU, ICU, RAJSHAHI MEDICAL COLLEGE HOSPITAL, RAJSHAHI, Bangladesh
*Antimicrobial Resistance and Infection Control* 2017, **6(Suppl 3):**P53

**Introduction:** Worldwide, ICUs are faced with increasingly rapid emergence and spread of AM-resistant bacteria because of frequent use of broadspectrum AMs, shortage of nursing and other supporting staff due to economic pressures (which increases the likelihood of person-to-person transmission of microorganisms).

**Objectives:** The present study was, designed to the AM sensitivity pattern of microbial isolates from patients in intensive care units (ICUs) of a low resource setting tertiary care hospital, Bangladesh.

**Methods:** Prospective observational study (Descriptive longitudinal) done in ICU of Rajshahi Medical College Hospital, Bangladesh from 2014 to 2015. All demographic, clinical & laboratory data were recorded in a pre designed data collection sheet. The study protocol was approved by the institutional review board. All data were analyzed by using computer based SPSS (statistical program for social science)

**Results:** Over a period of 24 months, 1367 patients were admitted in the ICU with 129 cultures from 305 patients for laboratory tests. Out of these,organisms were isolated from 129 cultures taken from 305 patients, giving an infection rate of 42%.

Of the 129 cultures, 49 were Gram-positive and 75 Gram-negativ.

The most frequently isolated organisms were Coagulase-negative staphylococci (23.4%) and K. pneumoniae (22.5%).

Gram-negative isolates were sensitive to Meropenem (75.19%). The 3rd generation cephalosporins, Ceftriaxone were widely resistant (73.5%).

**DISCUSSION**

Infection with resistant organisms can be associated with poor prognosis if the initial antibiotic used does not provide adequate coverage.

AMs should be altered based on sensitivity results or stopped altogether if no organism has been isolated and the clinical picture of patients permit it.

**Conclusion:** Coagulase-negative staphylococci & K. pneumoniae are the predominant isolated organism.

Since the 3rd generation cephalosporins, Ceftriaxone are very ineffective due, possibly, to their frequent use in the ICUs studied, their use should be restricted.

**Disclosure of Interest**

D. A. H. Kamal Other conflict with: No conflicts of interest.

### P54

#### Oxa-48 Producing Klebsiella pneumoniae isolated from clinical samples in Iran

Leila Azimi, Shahnaz Armin, Fatemeh Fallah, Abdollah Karimi, Saeed Maham, Kiarash Ghazvini, Sepide Hasanzadeh
PEDIATRIC INFECTIONS RESEARCH CENTER, MOFID CHILDREN HOSPITAL, SHAHIDBEHESHTI UNIVERSITY OF MEDICAL SCIENCES, TEHRAN, IRAN, Tehran, Iran, Islamic Republic Of
**Correspondence:** Leila Azimi
*Antimicrobial Resistance and Infection Control* 2017, **6(Suppl 3):**P54

**Introduction:** Carbepenem resistant is one of the increasing global problems in health care systems. Carbapenem is one of the most effective antibiotic for treatment infections that caused by multi-drug resistance bacteria. So, emergence of resistance to carbapenem can lead to considerable problem.

**Objectives:** The aim of this study was detection of the OXA-48 as an important metallo-beta-lactamase in the isolated *Klebsiella pneumoniae* cause of serious nosocomial infection.

**Methods:** In this cross sectional study 32 carbapenem resistant *Klebsiella pneumoniae* isolated from clinical specimens in teaching hospitals in Mashhad and Hamedan were examined. After identification and determination of resistance to carbapenem, antibiotic susceptibility testing has been done according to CLSI guide line. OXA-48 gene has been detected by PCR assay and confirmed by sequencing.
**Results:** All of 32 isolated *K. pneumoniae* confirmed as carbapenem resistant strains were detected according to conventional biochemical and microbiological tests in this study. All of tested specimens resistant to imipenem or meropenem in collected strains and the most effective antibiotic was colistin. OXA-48 observed in 22 (69%) of carbapenem resistant *K. pneumoniae*.
**Conclusion:** OXA-48 is one of the important metallo-beta-lactamase that can cause of resistant to carbapenem as a broad spectrum beta-lactam antibiotic. OXA-48 gene is located on plasmid and can spread rapidly in bacteria. So spread of that is considerable problem in hospitals and maybe alarming for health care systems.
**Disclosure of Interest**
None Declared

### P55
### Plasmid control of carbapenem resistance in pandrug resistant Klebsiella pneumoniae strains in intensive care units of Mansoura University Hospitals

Ahmed M. Mostafa, Waleed M. Eldars, Mohamed A. Abou Elela
Medical Microbiology and Immunology department, Faculty of Medicine, Mansoura University, Mansoura, Egypt
**Correspondence:** Ahmed M. Mostafa
*Antimicrobial Resistance and Infection Control* 2017, **6(Suppl 3):**P55

**Introduction:** *Klebsiella pneumoniae*, although a part of the normal flora of intestine of humans, has received an increasing attention due to its increasing ability to resist many antimicrobials particularly carbapenem. *CRKP* has become an important nosocomial pathogen because of its rapid spread, limited treatment options, and ability to transfer resistance genes to other prevalent pathogens as *E.coli*.
**Objectives:** Determination of *K.pneumoniae* antimicrobial resistance pattern, detection of plasmid control in carbapenem-resistant strain and proving horizontal gene transfer.
**Methods:** Samples from patients are sent to Microbiological Diagnostics and Infection Control Unit (MDICU). After *K.pneumoniae* isolates are confirmed, the overall prevalence of *CRKP* among all isolates is detected and classified into MDR, XDR and PDR to all antibiotics using disk diffusion, MIC with resazurin dye and E-test. After plasmid extraction, isolates containing plasmids were subjected to PCR for KPC gene. Plasmid curing, transformation and conjugation experiments are done using Escherichia coli (ATCC® 25922™) kindly given by NAMRU3.
**Results:** From July 2014 till July 2015, including 962 patients with clinically suspected HAIs, from ICUs. Out of the 1289 clinical samples, 507 pathogenic organisms were detected. After confirmatory tests, 119 isolates were confirmed *K.pneumoniae and* was the only microorganism isolated from 102 samples (85.7%), while the other 17 samples (14.3%) were mixed growth. The overall prevalence of *CRKP* among all isolates causing nosocomial infections was 8.3%. Out of 42 CRKP isolates, 27 isolates were MDR to three antibiotics or more, 13 isolates were XDR to all antibiotics except one or two and only 2 isolates were PDR to all antibiotics. After plasmid extraction, 34 isolates were containing plasmids from them 25 isolates harbored KPC gene.Plasmid curing was successfully done. Transformation and conjugation experiment were successfully done using Escherichia coli (ATCC® 25922™).
**Conclusion:** Most effective drugs against ***CRKP*** infections were Tigecycline, Colistin and dual carbapenem therapy whereas the least effective were Ampicillin and Augmentin.
**Disclosure of Interest**
None Declared

### P56
### Carbapenem non-susceptibility of klebsiella pneumoniae isolates in hospitals from 2009 to 2016, data from the German Antimicrobial Resistance Surveillance (ARS)

Uwe Koppe, Anja Wieland, Ines Noll, Marcel Feig, Muna Abu Sin, Tim Eckmanns
Infectious Disease Epidemiology, Robert Koch Institute Berlin, Berlin, Germany
**Correspondence:** Uwe Koppe
*Antimicrobial Resistance and Infection Control* 2017, **6(Suppl 3):**P56

**Introduction:** Carbapenem resistance in *Klebsiella pneumoniae* is of significant public health concern and recently increased in several European countries.
**Objectives:** We analysed data from the German Antimicrobial Resistance Surveillance (ARS) System to investigate non-susceptibility of *K. pneumoniae* isolates from hospital patients to carbapenems.
**Methods:** ARS contains routine data of antimicrobial susceptibility testing from voluntarily participating laboratories. The first clinical *K. pneumoniae* isolate per quarter per hospital patient from 2009 to 2016 was included. Data from 2016 are preliminary and might be subject to changes. Isolates tested resistant or intermediate to at least 1 carbapenem were classified as non-susceptible.
**Results:** 177,936 isolates from 619 hospitals were included. The median patient age was 74 years (IQR: 61-82). 44.0% of the isolates were from women, 35.9% from men, and 20.1% were unspecified. Most isolates were from urine (57.3%), swabs (14.7%), respiratory tract (11.0%), wounds (8.8%), or blood (4.3%).
97.0% of the isolates were tested against ≥1 carbapenem (meropenem: 92.7%, imipenem: 94.9%, ertapenem: 33.8%) and predominantly evaluated according to EUCAST guidelines (68.9%).
943 of 172,624 isolates (0.55%, 95% CI 0.51; 0.58) were tested non-susceptible to ≥1 carbapenem. Non-susceptibility increased from 0.20% (95% CI 0.12; 0.32) in 2009 to 0.68% (95% CI 0.60; 0.76) in 2015 and 0.72% (95% CI 0.63; 0.82) in 2016. An analysis in continuously participating hospitals indicated an increasing linear trend ($p < 0.001$). Non-susceptibility was higher in men (0.82%; 95% CI 0.75; 0.89) than women (0.39%, 95% CI 0.35; 0.44) and highest in patients aged 1-19 years (1.37%, 95% CI 1.03; 1.82). Non-susceptibility differed by source: urine: 0.34% (95% CI 0.31; 0.38), swabs: 0.82% (95% CI 0.72; 0.94), respiratory tract: 0.83% (95% CI 0.71; 0.97), wounds: 0.62% (95% CI 0.50; 0.75), blood: 0.58% (95% CI 0.43; 0.78).
**Conclusion:** Non-susceptibility of *K. pneumoniae* isolates against carbapenems in Germany is still uncommon but steadily increasing. These results emphasize the need for public health measures to control further spread of carbapenem-resistance and preserve treatment options for patients.
**Disclosure of Interest**
None Declared.

### P57
### Epidemiological aspects of nosocomial infections caused by carbapenem-resistant gram-negative bacteria in a Brazilian Hospital

Ana Elisa Ricci Lopes[1], Mayra G. Menegueti[1], Cinara S. Feliciano[1], Gilberto G. Gaspar[1], Lecio R. Ferreira[1], Magda Fabbri Isaac Silva[1], Silvia Rita Marin da Silva Canini[2], Fernando Bellissimo-Rodrigues[3]
[1]Hospital das Clinicas FMRP/USP; [2]Escola de Enfermagem EERP/USP; [3]Faculdade de Medicina FMRP/USP, Ribeirão Preto, Brazil
**Correspondence:** Ana Elisa Ricci Lopes
*Antimicrobial Resistance and Infection Control* 2017, **6(Suppl 3):**P57

**Introduction:** The incidence of hospital-acquired infections caused by carbapenem-resistant Gram-negative bacteria has increased worldwide. These infections significantly impact in-hospital mortality rates and healthcare costs

**Objectives:** To evaluate epidemiological aspects of nosocomial infections caused by carbapenem-resistant Gram-negative bacteria in a Brazilian tertiary-care public-affiliated hospital.
**Methods:** This is a descriptive study, based on data from the surveillance system of the study hospital infection control service and on the patients' medical records admitted from 2014 January to 2015 December.
**Results:** Of the 15,991 patients admitted in these two years, 3.39% (543/15,991) developed any nosocomial infection and, among these, 14.18% (77/543) were infected by carbapenem-resistant Gram-negative bacteria. Most common isolates were *Klebsiella pneumoniae* (31), *Acinetobacter baumannii* (31), *Pseudomonas aeruginosa* (12), *Enterobacter cloacae* (2), and *Escherichia coli* (1). The mean age of the infected patients was 53 years old, ranging from 6 months to 89 years old. Patients have been hospitalized for an average period of 63 days, ranging from 8 to 443 days. The mean time between admission and onset of the nosocomial infection episode was 31 days, ranging from 6 to 221 days. Among these 77 patients, 40 (51.28%) were submitted to surgery and 42 (54.5%) were admitted to an intensive care unit. Regarding the topography of infection, 32.47% were bloodstream infections, 23.38% were pneumonia, 22.09% were surgical site infection infections, 14.29% were urinary tract infections, 6.49% were infections of a catheter insertion site, and 1.28% were tracheobronchitis. Most of the infected patients (64.9%) died in the hospital.
**Conclusion:** Our data highlight the emergence of carbapenem-resistant Gram-negative bacteria as cause of nosocomial infections and its high impact on in-hospital mortality rates, reinforcing the need of new preventive and therapeutic strategies to control the spread of these infections.
**Disclosure of Interest**
None Declared.

### P58

#### Clinical outcome of pan-drug resistant Klebsiella Pneumoniae (PDRKP) bloodstream infections- a single center study in India

Amarjeet Kaur[1], sumanth gandra[2], sharmila sengupta[1], yatin mehta[1]
[1]Medanta, The Medicity, Gurgaon; [2]Center for Disease Dynamics, Economics & Policy, delhi, India
**Correspondence:** Amarjeet Kaur
*Antimicrobial Resistance and Infection Control* 2017, **6(Suppl 3):**P58

**Introduction:** Carbapenem resistant *Klebsiella pneumoniae* bloodstream infections (BSI) have become endemic in Indian tertiary care hospitals. This led to increased use of colistin resulting in emergence of colistin resistant & pan-drug resistant strains.
**Objectives:** Objective of the study was to evaluate clinical outcomes of 50 patients with PDRKP BSI over a five year period in a tertiary care hospital
**Methods:** Retrospective chart review of 50 patients with PDRKP BSI was done. Isolates were identified as PDRKP if they were resistant to all available antibiotics including carbapenems and colistin. Pathogen identification and antibiotic susceptibility testing was done with VITEK 2 compact (bioMérieux, France). To identify independent risk factors associated with in-hospital mortality, risk factors with a *p*-value of <0.1 in univariate analysis were included in a stepwise multivariable logistic regression model. Statistical analysis was done using the STATA 12.1 (StataCorp, USA).
**Results:** 66 cases of PDRKP BSI were identified in the 5 year period (January 2011- December 2015). Among the 66 patients, 16 patients were excluded as they left the hospital against medical advice. Overall in-hospital mortality rate was 74% (37/50). In univariate analysis, risk factors significantly associated with mortality were- ICU stay (61.5% vs. 83.8%, $P = 0.07$), length of stay prior to onset of BSI (mean days (SD)- 9.2 (10.2) vs. 18.2 (20.2), $P = 0.06$), Pitts bacteremia score of ≥4 (23.1% vs. 86.5%, $P < 0.001$), pneumonia as source of BSI (0% vs. 27.2%, $P = 0.03$), and mechanical ventilation (30.7% vs. 89.1%, $P < 0.001$). In stepwise multivariate analysis, only Pitts bacteremia score ≥4 (OR,21.3 [95% CI, 4.3-105.4]; $P < 0.001$) was significantly associated with mortality. Among patients who died, 18 died within 48 hours of culture positivity.
**Conclusion:** PDRKP BSI is associated with a high mortality. Pan-drug resistant invasive infections are on the rise in Indian hospitals and there is an urgent need for newer antibiotics. Implementation of antimicrobial stewardship programs and stringent infection control measures is a priority.
**Disclosure of Interest**
None Declared

### P59

#### Predicting carbapenem resistance using whole genome sequencing

Isabella A. Tickler, Scott B. Dewell, JoAnn Kop, Fred C. Tenover
CEPHEID, Sunnyvale, CA, United States
**Correspondence:** Isabella A. Tickler
*Antimicrobial Resistance and Infection Control* 2017, **6(Suppl 3):**P59

**Introduction:** Gram-negative carbapenemase-producing organisms (CPO), especially Enterobacteriaceae, have become a global concern in healthcare settings due their rapid spread and resistance to most antimicrobial options. Early and accurate detection of CPOs is crucial for optimizing both infection control and therapy. However, phenotypic and genotypic results do not always agree, due to expression of β-lactam resistance, efflux mechanisms, and difficult to detect porin changes. Whole-genome sequencing (WGS) provides comprehensive predictions of antimicrobial resistance (AR), but generates substantial amounts of data.
**Objectives:** The goal of this study was to evaluate two popular web-based resources that identify AR genes from WGS sequences for their accuracy and ease of use.
**Methods:** We analyzed WGS data from 27 CPOs (previously identified by two commercial assays) by querying gene databases available from the Center for Genomic Epidemiology (ResFinder) and the Comprehensive Antibiotic Resistance Database (Resistance Gene Finder -RGI). The genotype predicted by WGS was correlated with standard antimicrobial susceptibility testing (AST) results for β-lactam, aminoglycoside, and tetracycline resistance.
**Results:** 258 unique resistance determinants were identified by RGI and 152 by ResFinder. Average correlation between AR genotypes and phenotypes ranged between 85-100% for most classes of antibiotics with the exception of tetracycline, where sensitivity relative to AST was only 58%. Both ResFinder 2.1 and RGI detected the presence of aminoglycoside resistance genes in 89% of phenotypically resistant strains. Correlation between resistance genotypes and resistance to cephem antibiotics was high (95% with ResFinder and 96% using RGI). ResFinder, however, provided a higher concordance between phenotype and genotype for carbapenem resistance than RGI (97% versus 85%, respectively; $p = 0.343$).
**Conclusion:** Both ResFinder and RGI predicted phenotypic resistance from WGS data with a high degree of accuracy. RGI was quick and easy to use, but missed three carbapenem resistance genes. ResFinder provides more options for analysis, and performed slightly better, but is limited to acquired resistance genes. Both are useful epidemiologic tools but are not yet ready to replace standard AST results.
**Disclosure of Interest**
I. Tickler Employee of: Cepheid, S. Dewell Employee of: Cepheid, J. Kop Employee of: Cepheid, F. Tenover Employee of: Cepheid.

### P60

#### Biofilm in hospital effluent: a carbapenemase hotspot between hospital and environment?

Jérôme Ory[1], Geneviève Bricheux[2], Christiane Forestier[2], Ousmane Traore[1]
[1]Service d'hygiène hospitalière, CHU Clermont-Ferrand; [2]Laboratoire Microorganismes Génome et Environnement, Clermont-Ferrand, France
**Correspondence:** Jérôme Ory
*Antimicrobial Resistance and Infection Control* 2017, **6(Suppl 3):**P60

**Introduction:** The emergence of carbapenemases represents a major problem due to their successful dissemination capacity. Biofilms formed in hospital effluents represent crossroads of human feces (bacteria and antibiotics) and environmental microorganisms.

**Objectives:** In the present work, we assessed the presence of antibiotics and carbapenem-resistant bacteria in hospital effluent and we pointed out the presence of mobile genetic elements with diffusion capacities.

**Methods:** Biofilms (15 days-old) formed in the effluents of the teaching hospital Gabriel Montpied, (Clermont-Ferrand, France) were disassembled and spread onto 8 μg/mL Imipenem-R2A/agar plates. Resulting colonies were identified using mass spectrometer analysis (MALDI-TOF technology BioMérieux) or 16S DNA sequencing. Antibiotic susceptibility patterns were determined using Vitek2 (Biomérieux). Genes conferring resistance to carbapenem were detected by multiplex PCR. The amount of imipenem potentially released in the hospital effluent was calculated using pharmacokinetics parameters and its Predicted Environmental Concentration (PEC) in the wastewater was calculated.

**Results:** Imipenem-resistant isolates were identified as *Aeromonas* (63%), *Pseudomonas* (23.6%), *Stenotrophomonas* (8%) and *Acinetobacter* (2.5%). Most of them harbored associated resistances to aminoglycosides, fluoroquinolon, tetracyclin and/or colistin. The majority of isolates harbored at least one integron and one carbapenemase-encoding gene: $bla_{GES}$, $bla_{VIM}$, $bla_{OXA\text{-}48}$, $bla_{NDM}$ and $bla_{KPC}$. The imipenem PEC was equal to 3.16 μg/L, indicating that all bacteria were potentially subjected to sub-MICs concentrations of imipenem within the effluent.

**Conclusion:** The presence of acquired resistance mechanisms in nearly all the carbapenem resistant isolates, together with the presence of low levels of antibiotics suggests that hospital wastewater represents a privileged place for antibiotic resistance dissemination. Specific monitoring of such environment should therefore be performed actively on a regular basis.

**Disclosure of Interest**

None Declared

### Surveillance of multidrug-resistant Gram-negative bacteria

#### P61

#### The economic burden of key gram negative bacteria in Australian Hospitals

Teresa M. Wozniak, Nicholas Graves, Kate Halton
Queensland University of Technology, Brisbane, Australia
**Correspondence:** Teresa M. Wozniak
*Antimicrobial Resistance and Infection Control* 2017, **6(Suppl 3):**P61

**Introduction:** Antimicrobial resistance of gram-negative bacilli (GNB) is a global threat but little is known of the health and economic impact at the country-level. This information is necessary to ensure relevant and local data are used to make clinical decisions and to drive health policy changes.

**Objectives:** To determine the impact of antimicrobial resistance on bloodstream, urinary and respiratory tract infections caused by *Escherichia coli* (*E. coli*), *Klebsiella pneumoniae* (*K. pneumoniae)* and *Pseudomonas aeruginosa* (*P. aeruginosa*) in Australian hospitals.

**Methods:** We developed a model to measure the healthcare costs of third-generation cephalosporin (3GC)-resistant *E.coli,* 3GC-resistant *K. pneumonia* and ceftazidime-resistant *P. aeruginosa* in Australia. The model utilizes Australian estimates of incidence density and the proportion of these that are resistant. These data are supplemented with estimates from high-quality studies of attributable morbidity and mortality. We performed a Monte Carlo simulation to estimate the healthcare costs of additional bed-days in 2014 for each resistant infection.

**Results:** In 2014, we estimate a total of 7,593 3GC-resistant *E.coli* infections, 1,091 3GC-resistant *K. pneumoniae* infections and 3,374 ceftazidime-resistant *P. aeruginosa* infections, which includes bloodstream, urinary and respiratory tract infections. In bloodstream infections (BSI), 3GC-resistant *E. coli* resulted in largest healthcare burden, with an additional 3,153 bed-days. Ceftazidime-resistant *P. aeruginosa* isolated from respiratory tract infections resulted in an additional 692 bed-days which amounted to an additional AUD1,862,779 (95% CI 1.7-2.0 million) per year. Both 3GC-resistant *E. coli* and *K. pneumoniae* BSIs resulted in an additional cost of AUD 5,798,512 (95% CI 1.5-12.9 million) and AUD1,311,030 (95% CI 325,785-3.1million), respectively and a total cost of AUD 8,972,321 for the three GNB resistant infections.

**Conclusion:** Understanding the country-specific epidemiology and consequent costs of resistant infections are increasingly important given the global spread of antimicrobial resistance and in particular for Australia, given the close proximity with highly endemic countries. These data provide the baseline estimates of the economic burden of resistance of key GNB within Australia.

**Disclosure of Interest**

None Declared

#### P63

#### Molecular characterization of resistance to beta-lactam antibiotics, aminoglycosides and colistin in carbapenem- and aminoglycoside-resistant gram-negative bacteria in Turkey

Tulay Kandemir[1], Togrul Nagiyev[1], Tulin Guven Gokmen[2], Farzad Heydari[1], Fatih Koksal[1]
[1]Medical Microbiology, Medical Faculty; [2]Medical Microbiology, Ceyhan Veterinary Faculty, Cukurova University, Adana, Turkey
**Correspondence:** Togrul Nagiyev
*Antimicrobial Resistance and Infection Control* 2017, **6(Suppl 3):**P63

**Introduction:** Resistance to colistin, the major drug of last-resort for carbapenem- and aminoglycoside-resistant Gram-negative bacteria, also is associated with plasmid-mediated *mcr-1* gene. Plasmids can harbour a lot of resistance genes together, and these genes can be transferred horizontally both within and between species.

**Objectives:** We aimed to investigate molecular mechanisms of resistance to β-lactam antibiotics, aminoglycosides and colistin in carbapenem- and aminoglycoside-resistant Gram-negative bacteria causing healthcare-associated infections in a major regional hospital in Turkey.

**Methods:** A total of 92 isolates, 70 *Acinetobacter baumannii,* 14 *Pseudomonas aeruginosa* and 8 *Klebsiella pneumoniae* were enrolled in the study. Only 4 (% 4.3) of them were colistin-resistant. Extended-spectrum β-lactamase (*ESBL*) genes, plasmid-mediated aminoglycoside resistance genes and *mcr-1* gene were investigated by using PCR method.

**Results:** Aminoglycoside resistance genes were detected in 14 (%15.2) isolates, including 7/14 (%50) *rmtC*-positive *P.aeruginosa*, 4/70 (%5.7) *rmtH*-positive *A.baumannii* and 3/8 (%37.5) *rmtH*-positive *K.pneumoniae*. The single colistin-resistant *A.baumannii* isolate carried only *bla*OXA-23-like gene. All of 3 colistin-resistant *K.pneumoniae* isolates were *bla*CTX-M-positive, 2 of them were *rmtH*- and *bla*OXA-24-like-positive, while other was *bla*OXA-48-like-positive and did not carry aminoglycoside resistance genes. Other *rmtH*-positive *K.pneumoniae* isolate was colistin-susceptible and also harbored *bla*OXA-48-like and *bla*CTX-M genes. All of 4 *rmtH*-positive *A.baumannii* isolates also harbored *bla*CTX-M genes, 2 of them further harbored *bla*OXA-23-like and *bla*OXA-24-like genes. Four of 7 *rmtC*-positive *P.aeruginosa* isolates also harbored *bla*OXA-24-like gene. The *mcr-1* gene was not detected.

**Conclusion:** The high coexistence rate of aminoglycoside resistance genes with ESBL genes may be a strong sign for upcoming threat. We can conclude that prospective studies investigating these resistance genes in larger populations, especially during the healthcare-associated infection outbreaks, is extremely critical for surveillance. Thus, rational use of antibiotics can decrease mortality.

**Disclosure of Interest**

None Declared.

### P64

#### Study of hospital microbial strains resistance to disinfecting agents as a factor for combating healthcare-associated infections

Nikolay Shestopalov, Vasily Akimkin, Lyudmila Fedorova, Aleksey Serov
Federal Budget Scientific Institution "Scientific Research Disinfectology Institute" of Federal Service for Surveillance on Consumer Rights Protection and Human Well-being, Moscow, Russian Federation
**Correspondence:** Vasily Akimkin
*Antimicrobial Resistance and Infection Control* 2017, **6(Suppl 3):**P64

**Introduction:** Prevention and control of healthcare-associated infections (HAI) are of a high priority in modern healthcare. Monitoring and investigation of the resistance of microorganisms isolated from patients and from hospital facilities to disinfecting agents (DA) allow to substantiate DA selection and correct timely rotation principles to reduce a possibility of spreading of resistant strains of HAI pathogens.

**Objectives:** A research on resistance to DA was carried out for two strains of microorganisms (*Acinetobacter baumannii* and *Elizabethkingia meningoseptica*), which caused severe septic diseases with several fatal cases in a perinatal center. *A. baumannii* strain was resistant to β-lactam antibiotics, II generation of aminoglycosides, fluoroquinolones, nitrofurans, levomycetins, but was susceptible to tetracyclines, glycylcyclines, and particularly to taigecycline. *E. meningoseptica* strain was resistant to β-lactam antibiotics, II and III generations of aminoglycosides, tetracyclines, glycylcyclines, nitrofurans, levomycetins, but was susceptible to fluoroquinolones. The main objective of the research was to evaluate efficacy of DA, which were used in the perinatal center, towards corresponding pathogens and to suggest possible alternatives.

**Methods:** The study was carried out according to the methodology developed at the Institute and harmonized with European norms for 5 DAs (3 agents based on quaternary ammonium compounds (QACs), 1 agent based on hydrogen peroxide, 1 agent based on dichloroisocyanuric acid sodium salt) that were used in the perinatal center.

**Results:** Study of strains susceptibility revealed resistance of *A. baumannii* to QACs based disinfectants and resistance of *E. meningoseptica* to all five disinfectants. After further study both strains were found to be susceptible to agents based on aldehydes, calcium hypochlorite, chloramine, peracetic acid and sodium percarbonate.

**Conclusion:** Conducted studies show an importance of monitoring and assessing of hospital microbial strains resistance not only to antibiotics, but also to disinfectants. This is required for conduction of timely scientifically based rotation of disinfectants, and allows to evaluate a possibility and necessity of biological disinfection for effective prevention of HAI.

**Disclosure of Interest**

None Declared.

### P65

#### Transferable resistance in gram-negative bacteria isolated from hospital environment in Slovakia

Lenka Michalikova[1], Jaroslava Brnova[1], Sona Hnilicova[1], Anna Streharova[2], Anna Liskova[3], Vladimir Krcmery[4]
[1]Department of Laboratory Medicine; [2]Department of Pubic Health, School of Health Sciences and Social Work, Trnava University, Trnava; [3]Laboratory of Molecular Microbiology, St. Elisabeth University, Bratislava, Slovakia, [4]Laboratory of Molecular Microbiology, St. Elisabeth University, Bratislava, Slovakia
**Correspondence:** Jaroslava Brnova
*Antimicrobial Resistance and Infection Control* 2017, **6(Suppl 3):**P65

**Introduction:** Antimicrobial resistance can be transferred between bacteria, and their plasmid-encoded resistant genes can be next transferred to other pathogens.

**Objectives:** We aimed to detect occurrence of transferable resistance among Gram-negative bacteria isolated from hospital environment in Slovakia between January - December 2015.

**Methods:** In this study, we tested 137 isolates of Gram-negative bacteria from hospital environment for transferable resistance. Transferability of resistance–determinants was assessed by phenotypic methods with conjugational experiments. Strains of rifampin-resistant Escherichia coli 3110, rifampin-resistant Proteus mirabilis P38, rifampin resistant Pseudomonas aeruginosa 1008, and rifampin resistant Pseudomonas aeruginosa 1670 were used as recipient strains. Escherichia coli strain ATCC 25922 was used as a control strain.

**Results:** Analysis of transferable resistance was performed with 158 environmental isolates, excluding 21 (13,3%) isolates for selectable resistance of rifampicin and nalidixic acid. Transferable resistance was confirmed in 54 isolates (39,4%), of which 29 (53,7%) were Pseudomonas spp., 12 (22,2%) Enterobacter spp., 10 (18,5%) Klebsiella spp. and 3 (5,6%) other species. Colistin (36; 66,7%), cefotaxime (21; 38,9%), ceftazidime (16; 29,6%) and aztreonam (16; 29,6%) resistances were the most frequently transferred resistotypes. Transfer only the one determinant of resistance was observed in 36 isolates (66,7%) and the multiple transfer in 18 isolates (33,3%). Interspecies transfer among the multiresistant Gram-negative bacteria was observed in 29 isolates (53,7%). The most frequent recipient of antibiotic resistance determinants was strain Escherichia coli 3110 with 48 transfers (88,9%), followed Pseudomonas aeruginosa 1670 (22 transfers; 40,7%), Pseudomonas aeruginosa 1008 (19 transfers; 35,2%) and Proteus mirabilis P38 (5 transfers; 9,3%).

**Conclusion:** In this study, we described high proportion of transferable resistance among environmental isolates of Gram-negative bacteria from health-care facilities in Slovakia. It was shown that multiple resistance determinants can be transferred not only among same species, but interspecies, too.

**Disclosure of Interest**

None Declared.

### P66

#### Withdrawn

### P67

#### Antimicrobial resistant bacteria in an intensive care unit, incidence and risk factors

Asma Ammar[1], Nabiha Bouafia[1], Asma Ben Cheikh[1], Olfa Ezzi[1], Mohamd Mahjoub[1], Hela Ghali[1], Imed Chouchène[2], Nesrine Sma[2], Hammadi Bousarsar[2], Manel Marzouk[3], Jalel Boukadida[3], Mansour Njah[1]
[1]Hospital Hygien Department; [2]Medical intensive care unit; [3]Laboratory of microbiology and immunology, University Hospital. Farhat Hached-Sousse, Tunisia, Sousse, Tunisia
**Correspondence:** Asma Ammar
*Antimicrobial Resistance and Infection Control* 2017, **6(Suppl 3):**P67

**Introduction:** In intensive care units (ICUs), health-care associated infections (HAI) caused by antimicrobial-resistant bacteria (AMRB) constitute a difficult task for physicians.

**Objectives:** - To determine the incidence of HAI caused by AMRB in a medical ICU of a university hospital and to describe the antibiotic resistance profiles of infecting pathogens

- To identify independent risk factors for acquiring AMRB in ICUs

**Methods:** We conducted a 15 months prospective study (September 15$^{th}$ 2015 to December 15$^{th}$ 2016) in the adult medical ICU of University Hospital-Farhat Hached (Sousse-Tunisia). Patients admitted in the ICU for more than 48H were included. The resistance patterns of all isolates were collected. Univariate and multivariate analysis were performed to identify independent risk factors for acquiring AMRB. A *p* value of 0.05 was considered significant.

**Results:** During the study period, 221 patients were eligible, 40 of them developed 45 AMRB acquired infections with an incidence of 16.08/1000 patient-days. AMRB was mostly incriminated in ventilator associated pneumonia (56.5%).The most frequent organisms were: AMR *Acinetobacter Baumanii sensitive only to colimycin and Rifampicin*

(73.3%), Extended-spectrum beta-lactamase producers (15.5%) represented essentially by *Klebsiella pneumoniae.* Finally Methicillin Resistant *Staphylocoque Aureus* in 11.1% of cases. Independent risk factors of AMRB acquired infections are duration of sedation OR = 1.19, CI $_{95\%}$ [1.08; 1.38], $p < 10^{-3}$, Immunodeficiency; OR = 5.33, CI $_{95\%}$ [1.58; 17.94], $p = 0.007$ and duration of central venous catheter OR = 1.06, CI $_{95\%}$ [1.01; 1.11], $p = 0.019$.

**Conclusion:** The study demonstrated the emerging trend of AMRB in our ICU. Therefore, judicial use of antibiotics, the strengthening of infection control measures and resistance surveillance of pathogens should be emphasized in order to prevent the spread of AMRB.

**Disclosure of Interest**

None Declared.

### P68

#### Epidemiology of multidrug-resistant organism infection in patients with traumatic injury

Hyo-Jin Lee[1], Yang Ree Kim[1], Hang Joo Cho[2], Kyoung Ho Choi[3]
[1]Department of Internal Medicine; [2]Department of Trauma Surgery; [3]Department of Emergency Medicine, College of Medicine, The Catholic University of Korea, Seoul, Korea, Republic Of
**Correspondence:** Hyo-Jin Lee
*Antimicrobial Resistance and Infection Control* 2017, **6(Suppl 3):**P68

**Introduction:** Infection remains a major cause of mortality and morbidity after trauma. The emergence of multidrug-resistant organisms (MDRO) is responsible for limited therapeutic options.

**Objectives:** The objective of this study was to identify the prevalence and risk factors of MDRO infections in multiple trauma patients.

**Methods:** We conducted a single-center, retrospective study of 820 adult patients admitted to a trauma center of Uijeongbu St. Mary's Hospital in Korea from 2014 to 2015. Only first positive culture data per organism from each specimen were analyzed. Organism considered a contaminant was excluded.

**Results:** Total 515 positive cultures from 125 patients were obtained. Carbapenem-resistant *Acinetobacter baummanii* (CRAB, 22.1%), methicillin-resistant *Staphylococcus aureus* (MRSA, 14.2%), carbapenem-resistant *Pseudomonas aeruginosa* (2.1%), vancomycin-resistant *Enterococci* (1.7%), and carbapenem-resistant *Enterobacteriaceae* (0.6%) were isolated in multiple trauma patients. Multivariate logistic regression analysis found that, abdomen Abbreviated Injury Scale ≥3 (odds ratio [OR] = 2.83; 95% confidence interval [CI] = 1.07-7.89; $P = 0.039$), intensive care treatment (OR = 5.07; 95% CI = 1.67-17.89; $P = 0.006$), number of operative procedures (OR = 3.49; 95% CI = 1.33-9.96; $P = 0.014$), and pneumonia (OR = 5.51; 95% CI = 1.97-16.96; $P = 0.002$) were risk factors for MDRO infections. Age, sex, preexisting medical condition, Glasgow Coma Scale, type of injury, and invasive device usage were not statistically related to MDRO infections. By Kaplan-Meier analysis, the in-hospital mortality was higher in the MDRO group (18.8% vs. 5.41%; $P = 0.053$).

**Conclusion:** Multiple trauma patients had high rates of CRAB and MRSA. This research suggests that trauma patients with severe abdominal injury, intensive care treatment, multiple operative procedures and pneumonia are at increased risk for MDRO infections. Recognition of the identified risk factors may help to guide the appropriate use of empirical antibiotics.

**Disclosure of Interest**

None Declared.

### P69

#### Collaboration between clinicians, microbiologists and epidemiologists to achieve robust AMR surveillance, AMR stewardship and infection prevention and control

Romella Abovyan[1], Kristina Gyurjyan[2], Tjalling Leenstra[3], Nandini Shetty[4], Gayane Ghukasyan[5], Nienke BRUINSMA[6], Ana Paula Coutinho Rehse[7]
[1]National Center of Disease Control and Prevention; [2]Public Health Unit, Ministry of Health, Armenia, Yerevan, Armenia; [3]Centre for Infectious Disease Contro - WHO Collaborating Centre for Antimicrobial Resistance Epidemiology and Surveillance, National Institute for Public Health and the Environment, Bilthoven, Netherlands; [4]Reference Microbiology Services - WHO Collaborating Centre for Reference & Research on Antimicrobial Resistance and Healthcare Associated Infections, Public Health England, London, United Kingdom; [5]WHO Country Office Armenia, WHO REGIONAL OFFICE FOR EUROPE, Yerevan, Armenia; [6]Control of Antimicrobial Resistance Programme - Division of Communicable Diseases and Health Security; [7]Health Emergencies Programme - Division of Health Emergencies and Communicable Diseases, WHO REGIONAL OFFICE FOR EUROPE, Copenhagen, Denmark
**Correspondence:** Romella Abovyan
*Antimicrobial Resistance and Infection Control* 2017, **6(Suppl 3):**P69

**Introduction:** Armenia has not yet established a formal antibiotic resistance (AMR) surveillance system program. An important limiting factor to conducting routine AMR surveillance is the underutilization of bacteriological diagnostics in routine clinical practice.

**Objectives:** To demonstrate that routine bacteriological diagnostics and AMR surveillance can provide clinicians with the information needed to guide better antimicrobial treatment decisions and inform infection prevention and control policies in clinical settings.

**Methods:** Descriptive observational study. Clinicians will be requested to send blood cultures for all cases meeting a standardized case definition of sepsis. Local diagnostic laboratories underwent capacity building workshops to standardize detection of AMR according to EUCAST methodology. A national reference laboratory will be supported to provide confirmation of AMR and to collate the data. Data will be analyzed by epidemiologists and reported as part of surveillance program, to inform stewardship and IPC.

**Results:** The European strategic action plan on AMR was adopted in September 2011. The government of Armenia developed a national AMR action plan in 2015. Since then, Armenia is working towards achieving a robust AMR surveillance program that will contribute to the CAESAR and eventually to WHO GLASS. The study is in the implementation phase and data collection has not yet commenced. In addition to technical guidance, material support will be provided to clinicians and hospital laboratories. Samples collected as part of this study will be processed in parallel at the hospital laboratory and at the national reference laboratory for AMR.

**Conclusion:** As a result of this study, baseline data on main pathogens causing BSIs and their antimicrobial susceptibility patterns will become available. Capacity for diagnostic bacteriologic testing at the local hospital laboratory and at the national reference laboratory level will be strengthened

**Disclosure of Interest**

None Declared.

### P70

#### Emergence of Elizabethkingia meningoseptica as a nosocomial pathogen causing outbreaks and lessons learnt in containing the spread in the hospital

Teena Wadhwa, Sharmila Sengupta, Smita Sarma, Amarjeet Kaur, Navin Kumar, Usha Baveja
Department of Microbiology and Infection control, Medanta,The Medicity, Gurugram, India
**Correspondence:** Teena Wadhwa
*Antimicrobial Resistance and Infection Control* 2017, **6(Suppl 3):**P70

**Introduction:** An outbreak of *Elizabethkingia meningoseptica* occurred between October and November 2014, in the liver transplant unit of tertiary health care center in northern India. A steady rise of incidence of this nosocomial pathogen had been observed prior to the outbreak.
**Objectives:** This study reports lessons learnt from the outbreak, early recognition of the pathogen, role of environmental surveillance and infection control measures undertaken to contain spread or outbreaks in the hospital.
**Methods:** Identification and antimicrobial sensitivity of *E meningoseptica* isolated from clinical and environmental samples were done by Vitek 2 Compact (Biomerieux). Identification was confirmed by Polymerase chain reaction (PCR) for isolates identified during the outbreak. Environmental samples were collected from water, sink surfaces, furniture and equipments that were in immediate contact of patients who were culture positive for *E meningosepticum*. Rigorous cleaning and decontamination measures were undertaken increasing the number to three times daily (initially done twice) and cohorting of the patients was done. Traffic in those wards and rooms were controlled. Stringent hand hygiene practices were assured for the staff and visitors.
**Results:** A total of 473 isolates were identified between 2010 and 2016, it showed a steady rise from 19 in 2010 to 145 in 2014, with an outbreak in the liver transplant unit and then decreasing to 126 in 2015 and 42 in 2016. It was most commonly seen in patients of liver transplant (32.13%) followed by Gastroenterology (21.35%) and were mostly isolated from blood (38.68%) followed by endotracheal secretions (23.89%).Out of 117 environmental samples, 4(3.4%) surface swabs grew *E meningoseptica*.
**Conclusion:** *E meningoseptica* is an emerging multiresistant nosocomial pathogen with potential to cause outbreaks. Its early recognition and implementation of stringent infection control measures helped in reducing number of cases in subsequent years. Vigilant environmental surveillance and infection control practices are the mainstay in preventing such environmental organisms establishing as pathogens in resource limited settings
**Disclosure of Interest**
None Declared.

### Outpatient antibiotic use and antibiotic stewardship

#### P71
**Public engagement in promoting antibiotic awareness in Hong Kong**
Suk Yee J. Leung, Ada W. Wong, Andrew T. Wong
[1]DEPARTMENT OF HEALTH, INFECTION CONTROL BRANCH, HONG KONG, Hong Kong
**Correspondence:** Suk Yee J. Leung
*Antimicrobial Resistance and Infection Control* 2017, **6(Suppl 3):**P71

**Introduction:** Multi-drug resistant organisms (MDROs) are one of the most pressing issues in public health nowadays. The overuse and misuse of antibiotics has accelerated the spreading of MDROs. Infection Control Branch (ICB) launched out a "Use Antibiotic Wisely. Prevent Antimicrobial Resistance" Video Competition in 2015-2016. The competition aims to raise public's awareness on using antibiotic wisely so as to prevent antimicrobial resistance.
**Objectives:** To encourage general public especially the younger generation to support safe use of antibiotics and realize the antimicrobial resistance is a major threat to public health in Hong Kong.
**Methods:** A designated mini-web was developed to promote smart use of antibiotics and call for participation in video competition was launched out during Antibiotic Awareness Week (AAW) 2015. In addition, invitations to secondary school students to participate in the competition were disseminated by Education Bureau. Publicity in social media and briefing sessions on antibiotic awareness was arranged and conducted.
All submissions were assessed by professionals in infection control (IC) and film production. The champion and winners were selected by microbiologists. Final entries were uploaded to the website for public voting. The entry with highest number of votes of each category was awarded the Most Liked Award.
**Results:** There were 2 categories targeting general public and secondary school. A total of 32 video submissions (12 in secondary school categories and 20 in open category) were received. The 6 finalist entries were uploaded to social media (Facebook) for public voting. A total of 2,356 voters had selected their preferences.
**Conclusion:** The video competition is an innovative idea to promote antibiotic awareness programme which was successfully implemented in Hong Kong. Delivering the antibiotic awareness education through video competition among general public is the first of the kind in Hong Kong so far. In this program, we have also taken the opportunity to deliver health education to the participants through a briefing session and public health talk in the award ceremony. The submitted videos are full of creativity and affective impact that has ability to engage viewers on the importance of "Use Antibiotic Wisely. Prevent Antimicrobial Resistance".
**Disclosure of Interest**
None Declared

#### P72
**Using a theoretical model to explore nurse independent prescribers' antibiotic prescribing behaviour**
Valerie Ness, Lesley Price, Kay Currie, Jacqui Reilly
Nursing and Community Health, Glasgow Caledonian University, Glasgow, United Kingdom
**Correspondence:** Jacqui Reilly
*Antimicrobial Resistance and Infection Control* 2017, **6(Suppl 3):**P72

**Introduction:** Antimicrobial Resistance (AMR) is a major public health concern and inappropriate prescribing is a contributing factor, especially when managing Upper Respiratory Tract Infections (URTI). More nurses than ever are now prescribing; however, little is known about their antibiotic prescribing behaviour.
**Objectives:** This mixed method study used the Reasoned Action Approach (RAA) (Fishbein & Ajzen 2010) to explore and measure nurses' intention to manage patients, presenting with an URTI, without prescribing an antibiotic. The RAA suggests behaviour is influenced by attitudes, perceived norms and perceived behavioural control (PBC).
**Methods:** In stage one, one-to-one telephone interviews took place with 27 Nurse Independent Prescribers (NIP) from across Scotland, to establish their most accessible beliefs. A RAA guided data collection tool was used and open-ended questions were asked to elicit the constructs. Inductive content analysis took place. Interviews were coded, categorised and then ranked in order of most frequently occurring categories.
**Results:** Attitude: Most frequently mentioned advantage for managing patients without prescribing an antibiotic was reducing AMR ($n = 21$;78%); disadvantage was the stress of patient expectation ($n = 11$;41%).
Perceived Norm: Most frequently mentioned groups, most likely to manage patients without prescribing were other NIPs ($n = 11$;41%); least were General Practitioners ($n = 16$;59%).
PBC: Most frequently mentioned barrier to managing patients without prescribing was patient pressure ($n = 21$;78%); most frequent enabler was time to manage patient expectations by giving advice ($n = 27$;100%).
**Conclusion:** This study has explored NIP antibiotic prescribing behaviour in patients with URTI. Implications for clinical practice are consideration of the effect of patient expectations and the challenges this creates when trying to manage patients without prescribing an antibiotic. Using a behavioural model allows researchers to understand the uniqueness of a behaviour and the factors that determine that behaviour. Findings from this study have informed the design of a national, cross-sectional survey, as a precursor to designing targeted interventions for NIP behaviour change.

**References**
Fishbein, M., Azjen, I. 2010, Predicting and Changing Behaviour: The Reasoned Action Approach. Psychology Press, New York.
**Disclosure of Interest**
None Declared.

### P73

#### Knowledge, attitude and practice of appropriate use of antibiotics among primary health care workers in Zaria

Onoja-Alexander O. Mary[1], Olorukooba A. AbdulHakeem[2], Igboanusi C. John-Camillus[3], Onoja D. Alexander[4], Bulus B. Musa[5]
[1]Department of Community Medicine, Ahmadu Bello University Teaching Hospital; [2]Department of Community Medicine; [3]Department of Community Medicine, Ahmadu Bello University Teaching Hospital Zaria, Zaria; [4]Wellcare Initiative Kano, Kano; [5]Department of Medicine, Gombe State University Teaching Hospital, Gombe, Nigeria
**Correspondence:** Onoja-Alexander O. Mary
*Antimicrobial Resistance and Infection Control* 2017, **6(Suppl 3):**P73

**Introduction:** Antibiotic prescribing by primary health care (PHC) physicians and other health workers in the absence of bacterial infections is a problem in many countries especially developing countries like Nigeria
**Objectives:** The aim of the study was to assess the knowledge, attitude and practice of appropriate use of antibiotics among PHC workers in Zaria.
**Methods:** A cross sectional descriptive study among 119 PHC workers who were selected using multi stage sampling technique.Data was collected using pre-tested semi-structured self-administered questionnaire. Data was analysed using SPSS version 21, Chi-square test was used to test for association and results presented in tables and charts.
**Results:** Majority (99.2%) of the respondents have heard of appropriate antibiotic use and correctly defined it (76.5%). Majority (91.6%) of them were aware of antibiotic resistance. Only 22.7% of respondents correctly knew that antibiotic is not the first line therapy in acute diarrhoeal disease. Only 19.3% had good knowledge Two-fifth (40.3%) of the respondents strongly agreed on the importance of knowing the resistance rate of the bacteria in the local setting while prescribing. Only 31.1% of the respondents had positive attitude. Majority (63.9%) of the respondents prescribed antibiotics greater than once a day. Only 35.3% used generic name alone in their prescription and 35.2% of them usually request for Microscopy Culture and Sensitivity test before antibiotic prescription. About 17.1% had good practice of appropiate use of antibiotics.The three most common antibiotics being prescribed were Amoyxl, Ampiclox and Co-trimoxazole.
**Conclusion:** Majority of the PHC workers in Zaria prescribed antibiotics more than once a day, and their knowledge, attitude and practice towards appropriate use of these drugs were poor. Hence there is the urgent need to train these PHC workers by the local health authorities on appropriate use of Antibiotics.
**Disclosure of Interest**
None Declared.

### P74

#### Antibiotic stewardship in primary care: what is the best strategy?

Isabel Neves[1,2,3], Gabriela Poças[3,4], Ricardo C. Abreu[1,2], Sofia Jordão[1,2], Mario Guimarães[1,2], Maria J. Soares[1,5], Ilda Devesa[3], Valquíria Alves[1,3,5]
[1]Antibiotic Stewardship Group; [2]Infectious Diseases Unit; [3]Infection Control and Antimicrobial Resistance Unit; [4]Primary Care; [5]Microbiology Laboratory, Matosinhos Local Health Unit, Matosinhos, Portugal
**Correspondence:** Isabel Neves
*Antimicrobial Resistance and Infection Control* 2017, **6(Suppl 3):**P74

**Introduction:** Portugal has a high rate of associated healthcare infection, antibiotic prescription cares judicious use and antimicrobial resistance is worrying. Primary care (PC) prescribe 80% of all antibiotics. In recognition of the urgent need to improve antibiotic use in this setting and reduce resistance, we apply an Antibiotic Stewardship Program (ASP).
**Objectives:** To infer the benefits of ASP in PC in monitoring quinolones prescription and antimicrobial resistance.
**Methods:** Our PC includes 20 care units and a proximity emergency unit and serves a population of 174931 inhabitants. Multimodal strategy: 1. Training in antibiotic therapy was provided to family doctors, promoting its correct use. 2. Strategy *front end*: prescription according to national guidelines and community microbiological chart. 3 Selective report of antibiotic susceptibility test results as microbiology-based antibiotic stewardship intervention. 4. ASP Group available for proactive consulting and case talk. 5. Monitoring results discussion with medical coordinators/feedback to prescribers. Quinolone consumption data were taken from the PC prescription data base and the ATC and DDD system were used in the analysis of the data. Community microbiological chart are given annually by centralized microbiology laboratory and posted on the internet.
**Results:** In five years (2011 to 2016) quinolones consumption was audited, and there was a reduction from 0,75 to 0.40 DHD (2011 to 2016, respectively) and improved susceptibility of *E. coli* to quinolones from 79 to 86% and *S. aureus* to methicillin from 52 to 80% (2011 and 2016, respectively).
**Conclusion:** This strategy allowed to reduce direct quinolone antibiotic consumption and this was reflected in the decrease in antimicrobial resistance. Training, standard prescription, availability for proactive consulting and feedback to prescribers are tools that help judiciously use antibiotics in PC.
**Disclosure of Interest**
None Declared

### P75

#### Knowledge of antibiotic resistance and proper antibiotic use among patients attending a private hospital in Zaria, North-Western Nigeria

Lawal Amadu[1], Olorukooba A. Abdulhakim[1], Aisha A. Abubakar[1], Muawiyyah B. Sufiyan[1], Bilyaminu B. Lawal[1], Shamsuddeen S. Yahaya[2], Muhammad S. Balogun[3], Stephen Z. Bayero[1], Bashir P. Zakariyya[1]
[1]Community Medicine Department, Ahmadu Bello University Zaria, Kaduna state, Nigeria, Zaria; [2]Katsina state primary Health Care Development Agency, Katsina state ministry of Health, Katsina, [3]African Field Epidemiology Network, AFENET, Abuja, Nigeria
**Correspondence:** Lawal Amadu
*Antimicrobial Resistance and Infection Control* 2017, **6(Suppl 3):**P75

**Introduction:** Antibiotics resistance is a major public health problem globally. The main contributor to this problem is human behaviours at various levels. Understanding the knowledge and attitude of patients and the general public towards antibiotics resistance is key to controlling this public health problem.
**Objectives:** We aimed at determining the knowledge of antibiotic resistance and proper antibiotic use among patients attending a private hospital in Zaria, North-Western Nigeria.
**Methods:** A cross sectional descriptive study was carried out among 340 respondents selected using a systematic random sampling technique. A pretested self-administered questionnaire was used to elicit information from the respondents. Data was analysed using IBM SPSS version 21.0. Bivariate analysis was done using chi-square test and a *p*-value ≤0.05 was considered statistically significant.
**Results:** Only 9.71% of the respondents had good knowledge of antibiotics resistance while 16.47% appropriately used antibiotics. The average monthly income of the respondents was associated with knowledge of antibiotic resistance ($p < 0.001$) and proper antibiotic use ($p < 0.001$). Occupation of respondents, level of education and knowledge of antibiotic resistance were associated with proper antibiotic use ($p < 0.001$, $p < 0.001$ and $p < 0.001$ respectively).
**Conclusion:** There was poor knowledge of antibiotic resistance and low prevalence of appropriate antibiotic use among the respondents. Therefore, educational interventions are needed to promote appropriate use of antibiotics among the public.
**Disclosure of Interest**
None Declared.

### P76

#### Ampiclox: antibiotic or analgesic? Self-reported use of ampiclox among farmers and labourers in rural communities in the Nigerian State of Kano

Ibrahim Yusuf[1], Abdulhakeem Gbadamosi[2], Musa Haruna[3], Sani Yahaya[1]
[1]Microbiology, Bayero University, Kano, Kano; [2]Nephrology, Federal Medical Center, Katsina, Katsina; [3]Biology, Kano University of Science and Technology, Wudil, Kano, Nigeria
**Correspondence:** Ibrahim Yusuf
*Antimicrobial Resistance and Infection Control* 2017, **6(Suppl 3):**P76

**Introduction:** The proliferation of substandard Ampicillin and cloxacillin antibiotics (popularly known as Ampiclox) and their abuse, especially in the rural communities in Nigeria is highly worrisome. Three separate encounters with some farmers and labourers purchasing and using Ampiclox as a pain reliever at some different unregistered drug stores operated by non-educated personnel in rural areas necessitated the conduct of this study.

**Objectives:** The main objective of the study is to assess the knowledge and practice of randomly selected farmers and labourers (males and females) in rural areas toward use of Ampiclox.

**Methods:** A survey of 200 farmers and labourers (100 each) recruited from 3 selected local government areas of Kano, North-West Nigeria was conducted by means of a self-report drug-use questionnaire and interview in October 2016.

**Results:** The majority of respondents (87%) lack formal education and were from a low socio-economic class that lived below $2 daily. About 75% of them had self-used Ampiclox capsules before the study, and 26% (52/200) had used it for at least a time as a pain reliever. Further, 23% of farmers and 15% of labourers had used a dose of Ampiclox for at least a time to relieve pain after hectic work prior to the study. However, 76 respondents (38%) have the knowledge that Ampiclox is used to treat boils and rashes. Another 48% believed that Ampiclox is very good in treating fresh wounds only. Of the total respondents (151) who had self-used Ampiclox before, 35.0%, 31.7%, 23.8%, 9.2% had used only two capsules of Ampiclox to treat boils, rashes, wound infections and body pain respectively. Only 9% had used the complete dosage of Ampiclox for the treatment of either wound infections, rashes or boils. Low use of Ampiclox as a pain reliever was recorded among labourers when compared with farmers. Males used Ampiclox for painkiller significantly more than females (34:18). The majority of respondent's knowledge of using Ampiclox as a painkiller came from either families, friends and/or medical store attendants.

**Conclusion:** The authors stressed the need to develop a viable antibiotic-abuse preventive programme in the rural area to educate members of the communities on prudence use of antibiotics.

**Disclosure of Interest**

None Declared.

### P77

#### Knowledge of antibiotic resistance and appropriate use of antibiotics in children among caregivers in semi-urban community of Kaduna State Northwest Nigeria

Muawiyyah B. Sufiyan[1], Daniel A. John[1], Shafi'u Mohammed[2]
[1]Community Medicine; [2]Clinical Pharmacy, Ahmadu Bello University, Zaria, Kaduna, Nigeria
**Correspondence:** Muawiyyah B. Sufiyan
*Antimicrobial Resistance and Infection Control* 2017, **6(Suppl 3):**P77

**Introduction:** Globally, antibiotic resistance has led to higher medical costs, prolonged hospital stays and increased mortality. Irrational use of antibiotics has been shown to be the major factor driving the spread of resistance.

**Objectives:** The aim of this study is to assess the knowledge of caregivers in Basawa community on antibiotic resistance and appropriate use of antibiotics in their children.

**Methods:** We conducted a community based cross sectional descriptive study among 242 caregivers of children below the age of five years that were selected via cluster sampling technique. A pretested semi structured interviewer administered questionnaire was used for data collection. The collected data was then entered into a computer, validated and analyzed using statistical package for social sciences software (IBM SPSS) version 21.0 and results were presented in tables and charts.

**Results:** Majority of the respondents (79.0%) were within the age group of 15-34 years with a mean age of 28.24 (+8.43 years). Female respondents constitute 67.4% and 48.3% of all respondents have attained secondary level of education. Most of the respondents have never heard about antibiotic resistance (73.4%). At least three-quarter (77.5%) of them had poor knowledge on antibiotic resistance. More than half (63.3%) of the respondents had negative perception on antibiotic resistance while 80.9% of them used antibiotics inappropriately. Level of education was a strong predictor of perception ($p = 0.000$) and appropriate antibiotic use ($p = 0.032$).

**Conclusion:** Poor knowledge and negative perception on antibiotic resistance was prevalent among caregivers in studied community. They also use antibiotics on their children inappropriately, thereby contributing towards the prevailing problem of antibiotic resistance. More time and resources therefore need to be invested in awareness creation and educating the populace, especially caregivers of children on antibiotic resistance.

**Disclosure of Interest**

None Declared

### P78

#### Evaluating the utility of icd-10 codes for outpatient antimicrobial stewardship

Daniel J. Livorsi[1, 2], Carrie Linn[2], Bruce Alexander[1], Traviss Tubbs[1], Eli Perencevich[1, 2]
[1]Iowa City VA Health Care System; [2]University of Iowa Carver College of Medicine, Iowa City, United States
**Correspondence:** Daniel J. Livorsi
*Antimicrobial Resistance and Infection Control* 2017, **6(Suppl 3):**P78

**Introduction:** Audit-and-feedback can promote outpatient antimicrobial stewardship, but it is unclear if international classification of disease (ICD) codes can reliably identify antimicrobial-prescribing opportunities.

**Objectives:** We sought to define the frequency at which ICD-10 codes are entered for antimicrobial-prescribing visits and whether these codes have diagnostic validity.

**Methods:** Antimicrobial prescriptions from our medical center were retrospectively reviewed if the prescription originated from primary care or the emergency department (ED) during 2016. For each antimicrobial-prescribing visit, we sought a relevant ICD-10 code entered for that encounter that identified the indication for antimicrobials. If no relevant code was entered, chart review was performed to identify the provider's diagnosis. For all cases of cystitis and pneumonia, the diagnosis was validated using standardized criteria.

**Results:** There were 2,353 antimicrobial prescriptions from 52 providers. The most common reasons for prescribing antimicrobials were acute respiratory tract infections (ARTIs, 35.0%), skin-soft tissue infections (17.0%), and cystitis (14.4%). The most commonly prescribed agents were macrolides (17.6%), amoxicillin-clavulanate (17.0%), and fluoroquinolones (15.3%). All prescriptions included at least one refill. A relevant ICD-10 code was entered for 1153 (49.8%) antimicrobial-prescribing visits, including 529 (64.2%) ARTI-related visits. Among 17 providers who wrote ≥ 20 antimicrobial prescriptions, the median frequency of entering a relevant ICD-10 code was 60.2% (IGQ 44.7-75.0%). There was significant variability across providers in the frequency of using relevant ICD-10 codes for antimicrobial-prescribing visits ($p < 0.01$).

ICD-10 codes performed poorly in identifying cases of true cystitis ($n = 284$) and pneumonia ($n = 94$). The sensitivity, specificity, positive predictive value and negative predictive value were 66.3%, 59.8%, 76.1%, 59.8% for cystitis and 58.9%, 31.6%, 55.9%, 34.3% for pneumonia.

**Conclusion:** Performing antimicrobial audits based on ICD-10 codes would overlook a large proportion of outpatient antimicrobial-prescribing visits at our medical center. ICD-10 codes were neither sensitive nor specific in identifying true cases of pneumonia or cystitis.

**Disclosure of Interest**

None Declared

### P79

#### Outpatient antibiotic consumption in Switzerland: trends over the years 2013 – 2015 and comparison with Europe

Catherine Plüss-Suard[1], Andreas Kronenberg[2], Giorgio Zanetti[1] on behalf of the Swiss Centre for Antibiotic Resistance (anresis.ch)
[1]CHUV, Lausanne; [2]University of Bern, Bern, Switzerland
**Correspondence:** Catherine Plüss-Suard
*Antimicrobial Resistance and Infection Control* 2017, **6(Suppl 3):**P79

**Introduction:** Surveillance of antibiotic (AB) consumption is a key component of AB stewardship activities (1).

**Objectives:** Our objectives were to describe AB consumption in the outpatient setting in Switzerland in the frame of the programme anresis.ch and to compare it to European antibiotic use level.

**Methods:** AB for systemic use (class J01 of WHO Anatomical Therapeutic Chemical system, 2017) were collected over the years 2013-2015 through the billing office of the Swiss Pharmacist's Association. Aggregated data were converted into defined daily doses (DDD) and AB use expressed in DDD per 1000 inhabitants and per day (DID).

**Results:** Global AB consumption (5.7 DID in 2013, and 5.5 in 2015, -3%) was relatively low compared with European countries participating to ESAC-Net (20.1 in 2013, and 20.7 in 2015, +3%) (2). It was higher in the French- (12.0 and 11.2 DID resp., - 7%) and in the Italian-part of Switzerland (11.9 and 11.2 DID resp., - 6%). Geneva was the canton with the highest AB consumption (14.3 DID) followed by Fribourg (12.3) and Tessin (11.2) in 2015. Combination of penicillins was the most used class in all cantons (30% ± 7%, mean of global use ± standard deviation; ESAC-Net median 24%). Fluoroquinolone use was the second most used class (15% ± 3%; ESAC-Net median 8%). Global AB consumption was higher in patients aged 18-64 (3.7 DID, 61% of the Swiss outpatient AB use) and > 65 (1.8 DID, 29%) than in children. Global AB use in the quarters 1 and 4 was 20% higher than in the quarters 2 and 3 (ESAC-Net median 33%).

**Conclusion:** Compared with European countries, Switzerland has a relatively low global AB consumption. French- and Italian-speaking part of Switzerland remained higher AB consumers as observed 10 years earlier (3). However, global AB consumption is underestimated as a recent survey suggested that half of AB consumers got the drugs directly from their attending physicians (or hospitals) (4). Intervention to optimize AB prescribing practices should take regional differences into account.

**Disclosure of Interest**

None Declared.

### P80

#### Views and experiences of currently or recently hospitalized patients in four European countries with regard to barriers and facilitators to responsible antibiotic use: a qualitative descriptive study

Benedikt Huttner[1, 2], Veronica Zanichelli[1], Annelie Monnier[3], Gianpiero Tebano[4], Mirjana Stanic Benic[5], Inge Gyssens[3], Celine Pulcini[6], Vera Vlahović-Palčevski [5], Stephan Harbarth[2], Marlies Hulscher[7] on behalf of DRIVE-AB consortium
[1]Infection Control Program; [2]Division of Infectious Diseases, Geneva University Hospitals and Faculty of Medicine, Geneva, Switzerland; [3]Department of Internal Medicine, Radboud University Medical Center, Nijmegen, Netherlands; [4]Université de Lorraine, EA 4360 APEMAC, Nancy, France; [5]Department of Clinical Pharmacology, University Hospital Rijeka, Rijeka, Croatia; [6]CHRU de Nancy, Service de Maladies Infectieuses et Tropicales, Nancy, France; [7]Scientific Center for Quality of Healthcare, Radboud University Medical Center, Nijmegen, Netherlands
**Correspondence:** Benedikt Huttner
*Antimicrobial Resistance and Infection Control* 2017, **6(Suppl 3):**P80

**Introduction:** Patient's experiences and perceptions with regard to antibiotics have been extensively studied in the outpatient setting but little is known about the inpatient setting.

**Objectives:** To assess patients' experiences and views (not knowledge) with regard to barriers and facilitators concerning several aspects of the received antibiotic treatment (indication, regimen, diagnostics, side effects and resistance) in 4 European hospitals (CH, FR, HR, NL)

**Methods:** A purposeful sample of patients aware of having received antibiotics during their recent hospitalization was asked to participate in interviews and focus groups.

**Results:** Three focus groups (11 participants) and 26 individual interviews were performed and analyzed. Five main themes regarding barriers and facilitators of antibiotic use in hospitalized patients emerged: 1) Patients often don't receive information regarding different antibiotic-related aspects unless they ask. 2) Patients often feel unprepared to fully understand different aspects of their treatment but they want to be informed in an understandable way. 3) Patients trust hospital staff and often "surrender" to decisions taken by doctors 4) Patients feel that doctors don't prioritize communication due to time constraints. 5) Patients frequently rely on sources of information regarding antibiotics outside the hospital.

Results of this study will also be presented at ECCMID 2017.

**Conclusion:** In this study we found that during hospitalization patients often receive little information concerning antibiotics unless they specifically ask for it. However, patients often doubt their own ability to understand medical information and trust their physicians to take the best decisions for them. Patient-centered care strategies may promote patient involvement and improve communication between patients and healthcare providers.

DRIVE-AB is supported by IMI/EU and EFPIA.

**Disclosure of Interest**

None Declared.

## Hand hygiene around the globe

### P81

#### Impact of social media campaign: 5th may global hand hygiene day

Tcheun-How Borzykowski, Ermira Tartari, Daniela Pires, Didier Pittet
WHO collaborating centre for Patient Safety, Infection Prevention &Control, University Hospitals of Geneva, Genève, Switzerland
**Correspondence:** Tcheun-How Borzykowski
*Antimicrobial Resistance and Infection Control* 2017, **6(Suppl 3):**P81

**Introduction:** Social media has emerged as a valuable tool engaging health care workers (HCWs) worldwide to celebrate the World Health Organization (WHO) SAVE LIVES: Clean Your Hands global annual campaign.
**Objectives:** To describe the impact of social media (especially Twitter) interventions in engaging HCWs to build a momentum on the annual WHO call for action to sustain hand hygiene (HH) improvements.
**Methods:** Each year the WHO SAVE LIVES: Clean Your Hands campaign launches a call for action with a different theme in response to the global burden of health care associated infections and antibiotic resistance. The campaign aims to raise awareness and bring HCWs together in support of HH improvement globally. A campaign toolkit is made available on the WHO IPC webpages with resources to be used from health care facilities and countries worldwide. Hashtags such as #safesurgicalhands, #safeHANDS are made available for use in social media during the campaign to encourage commitment and a global reach. Symplur.com was used to generate data from the Twitter platform and a marketing company provided a landing page to collect photos shared with the hashtag promoting 5th May campaign.
**Results:** The impact of social media has been unique and unprecedented action has been reported. The safe hands campaign in 2015, using the hashtag #safeHANDS was a great success with a global reach of 55 million. The volume of conversations on the social networks (especially Twitter) started growing from April 20 with a peak of 12'000 messages using the hashtag #safeHANDS on the 5$^{th}$ of May. More than 6,200 photos have been published on the photo wall and shared through Twitter and Instagram with the hashtag #safeHANDS. The impact of the campaign has further increased, with an estimated reach of 98 million worldwide in 2016, from use of the #safesurgicalhands hashtag between April - July 2016. 15,000 photos have been shared. The campaign had a global impact with participants across the world. 19,217 health care facilities from 177 countries have registered to support the 5th of May campaign.
**Conclusion:** The use of social media holds promise to maintain a global momentum around the annual HH campaign. This proved to be a unique platform allowing HCWs to connect and engage in real time on a global scale.
**Disclosure of Interest**
None Declared.

### P82

#### Evaluation of the degree of compliance of hand hygiene recommendations in pediatric care areas of a third level hospital

Juan G. Mora Muriel, Ginger G. Cabrera Tejada, Carmen M. Benito Miralles, Marina D. C. Lopez-Bajoz Mesa, Victor M. Soler Molina, Patricia García Shimizu, Concepción García González, Jose Sanchez Payá
Unidad de Epidemiología, Servicio de Medicina Preventiva, Hospital General Universitario de Alicante, Instituto de Investigación Sanitaria y Biomédica de Alicante (ISABIAL - Fundación FISABIO), Alicante, Spain
**Correspondence:** Ginger G. Cabrera Tejada
*Antimicrobial Resistance and Infection Control* 2017, **6(Suppl 3):**P82

**Introduction:** In our center, the Pediatric Care Areas (PCA) are among those with a higher Degree of Compliance of Hand Hygiene Recommendations (DCHHR).
**Objectives:** The objective of the present study is to evaluate the DCHHR, and the factors associated with it, in the PCA of a third level hospital.
**Methods:** Observational cross-sectional study of a surveillance program by direct observation of the DCHHR during the daily activity of the health workers during the years 2005-2016. The outcome variable is DCHHR, and as explanatory variables: Area of Pediatric Care, years, age, sex, estate and use of pocket size alcohol-based hand rub solution (PAS). For the association study, the Chi-square test was used, and to quantify the magnitude of the association, the Odds Ratio (OR) was calculated with its 95% confidence intervals (95% CI). The adjusted OR was calculated using a logistic regression model. The level of statistical significance was $p <0.05$.
**Results:** We analyzed a total of 8704 observations in which HH was recommended. The DCHHR was: 52.0% in Surgery, 73.8% in Oncology, 61.4% in Infants, 61.9% in Schoolchildren, 73.2% in Neonatology, 70.2% in ICU-Neonatal, 61.7% in ICU-Pediatric, 51.8% in ER, 63.0% in Hospital Day Oncology. The magnitude of the association with the DCHHR was: Oncology,ORa = 2.7(2.2-3.5); Infants,ORa = 1.5(1.2-1.8); Schoolchildren,ORa = 1.5(1.3-1.9); Neonatology,ORa = 2.9(2.4-3.4); UCI-Neonatal,ORa = 2.5(2.0-3.0);UCI-Pediatric,ORa = 1.5(1.2-1.8); Urgencies,ORa = 1.0 (0.8-1.3);Oncology-Day Hospital,ORa = 1.6(1.3-1.9); Years(2009-2012),ORa = 1.2(1.0-1.3); Years(2013-2016),ORa = 1.1(1.0-1.3);Age(<35 years),ORa = 0.8(0.8-0.9); Gender(Male),ORa = 0.7(0.6-0.9); State(Nursing),ORa = 0.9(0.8-1.0); State(Nursing Assistant), ORa = 0.8(0.7-0.9); (Other),ORa = 0.3(0.2-0.5); use of PAS (Yes),-ORa = 2.2(1.9-2.4).
**Conclusion:** The DCHHR in PCA are among the highest of the center, being approximately 65%. The area of Oncology, Neonatology, ICU-Neonatal and the use of the pocket size alcohol solution for the accomplishment of the HH are the variables that, once adjusted by the rest of variables, greater magnitude of association have with the Degree of Compliance of Hand Hygiene Recommendations.
**Disclosure of Interest**
None Declared.

### P83

#### Promotion of hand hygiene: the experience of asst Gaetano Pini-CTO

Antonio Piscitelli, Paola Navone
Orthopaedic Institute ASST Gaetano Pini/CTO, Milan, Italy
**Correspondence:** Paola Navone
*Antimicrobial Resistance and Infection Control* 2017, **6(Suppl 3):**P83

**Introduction:**
Hand hygiene is the primary preventive measure in terms of cost-effectiveness of care-associated infections (CAI). In 2005, the WHO proposed the "Global Patient Safety Challenge" campaign, with the aim of reducing the overall incidence of CAI, through multimodal programs and strategies, interactive training tools and standardised methods for monitoring compliance.
**Objectives:**
On the basis of project Rimani, sponsored by the Health and Social Care Authority of Emilia-Romagna, the Orthopaedic Institute ASST Pini/CTO has implemented a project which seeks to increase compliance with hand hygiene by healthcare staff.
**Methods:**
A multidisciplinary work group was created to prepare the project documents. Starting from 5th April 2015, twenty operational units were asked to produce information and training material. On 5th May, during a Conference, a committee selected the three best works. The material was distributed on a monthly basis to the various departments. In addition to the training, the specifically trained operators of the Medical Monitoring Department carried out direct hand washing tests, filling out appropriate checklists. Non-compliance relating to the technique recorded in 2014 and 2015 was compared and a statistical analysis was conducted using Student's t-test for paired data.
**Results:**
Three tests were carried out in 2014. 99 cases were monitored and 5 non-compliance cases reported concerning the execution of the procedural steps, the running time and closing the tap with one's hands at the end of the wash.
In 2015 two tests, involving 30 operational units, were carried out. 60 observations were performed, and no non-conformities emerged concerning the technique. Statistical analysis showed a significant reduction between the 2014 and 2015 non-compliances ($P = 0.000$).
**Conclusion:**
The results obtained agree with the main findings in literature. The comparison between the 2014 and 2015 tests shows a trend improvement in the wash technique. This confirms the effectiveness of a multidisciplinary approach and recognises the active and participatory involvement suggested by the WHO.

References
*Nobile M.*, Conti C.**, Bastianelli A.*, Piscitelli A.°, Calori G.M.*, Navone P.**
** Orthopaedic Institute, ASST Pini/CTO, Milan, Italy*
*** Medicine and surgery, University of Milan, Italy*
*° Specialization School, Hygiene and Preventive Medicine, University of Milan, Italy*

**Disclosure of Interest**
None Declared.

### P84
### Adherence and knowledge of hand hygiene in six acute care hospitals

Milena Sorrentino[1], Gaetano Liotta[2], ANNA RITA MARUCCI[1], CARLA FERRARA[1], GIOVANNI GALEOTO[1], SALVATORE MORETTA[2], JULITA SANSONI[1]
[1]SAPIENZA UNIVERSITA' DI ROMA, ROMA; [2]ASL CASERTA, CASERTA, Italy
**Correspondence:** Milena Sorrentino
*Antimicrobial Resistance and Infection Control* 2017, **6(Suppl 3):**P84

**Introduction:** Safety care is crucial in health care during economic constrain and rationalization of resources. Interventions to identify gaps between evidence based nursing/medicine and clinical practice are necessary. Multidrug resistant microorganisms, requires strategies for infection control (IC). Hand hygiene (HH) is one of the best practice to control germs or bacteria spread.
**Objectives:** The aim of this study is to evaluate adherence and knowledge of healthcare professionals (HCPs) in an acute care hospital in order to correct bad practices.
**Methods:** The sample of the study is represented by HCPs of six acute care hospitals in southern Italy. The framework is supported by the social cognitive theory. A validated questionnaire was used to investigate HH beliefs, practices and knowledge. Ethical issues were considered for the study. Data were processed in SPSS.
**Results:** Out of the 716 HCPs respondents to the questionnaire, 86% considered HH an important measure for hospital acquired infection control. Some critical issues emerged: only 39% ($P <$ 0,0001) of HCWs consider themselves at risk of contracting an infection, 55% they claim to wash their hands before preparing infusion therapy and only 42% always wash their hands before contact with the patient.
Our sample has demonstrated a poor knowledge how correctly use alcohol based solutions, only 20% HCPs it does not use the alcohol solution if your hands are visibly dirty. Low adherence emerged mainly linked to overcrowding and excessive workloads.
**Conclusion:** The results of this study show a lack of knowledge regarding the use of alcoholic solution by HCPs making it essential in the short term the implementation training events. Current and future implications should concern changing practice environments that will take into consideration not only multimodal interventions for HCPs but also organization and structure in order to increase adherence and impact on patient safety. Limitations of this study are self-assessment with a possible overestimation of the data and sample isolation since the case of hospitals owned by a single health institution.
**Disclosure of Interest**
None Declared

### P85
### Evaluation of compliance with 'bare below the elbows' policy on the correctness of hand hygiene in medical staff of polish healthcare facilities

Emilia Szumska[1], Dorota Rozkiewicz[2], Elzbieta Oldak[2], Michal Zablocki[1]
[1]Medilab Sp. z o.o.; [2]Medical University, Bialystok, Poland
**Correspondence:** Emilia Szumska
*Antimicrobial Resistance and Infection Control* 2017, **6(Suppl 3):**P85

**Introduction:** In the last decade it is important to pay attention to 'bare below the elbows' (BBE) dress code in medical staff exercising direct patient's care.
**Objectives:** The aim of this study was to determine whether the medical staff was following the rules of BBE policy and how non-compliance with BBC rules affects hand disinfection correctness.
**Methods:** Directly after hand hygiene training session, we asked participants to disinfect their hands with alcohol-based preparation which is fluorescent under UV light. External observer noted if every member fulfilled the rules of BBE: short sleeves, no watch, no jewelry, unpainted nails, no artificial nails. Otherwise, received the information about following aspects: gender, job seniority, occupation, place of work. This study comprised 4202 people; 72,1% > nurses, 27,9% physicians. Among of them 3762 were working in hospital, 343 in clinic and 97 in long term care facilities.
**Results:** BBE compliance was observed in 2227/4202 (53%) of employees, statistically significant more often in nurses (1676/3031;55,3%) in comparison to doctors (551/1171; 47,1%) ($p = 0.0000$). The same correlation was showed in all of the analyzed medical facilities.
Among the doctors, the job seniority did not influence on frequency of BBE compliance (<10 years-51,7%, >10 years- 48,3%; $p = 0.2523$), whereas nurses working >10 years presented BBE compliance determined at level 77,4%. Only in 24,5% (135/551) physicians with BBE and 26,9% (451/1676) nurses with BBE incorrect hand disinfection procedure was showed. The group of No BBE was represented by 47% (1975/4202) of all employees. Artificial nails and rings were observed statistically significant more often in group of nurses in comparison to physicians (45,2% and 32,1% vs3 3,7% and 26,5%; respectively). Doctors statistically more often wore watches (44,0% vs 21%) and sheets with long sleeves (48,7% vs 16,8%) ($p < 0.05$) than nurses. In group of No BBE, 43,3% represents improper procedure of hand hygiene.
**Conclusion:** It was found that compliance with BBE policy has a close relationship to proper hang hygiene performance.
**Disclosure of Interest**
None Declared

### P86
### Effect of the world health organization multimodal hand hygiene improvement strategies on healthcare associated infections prevalence in 14 tertiary hospitals in Malaysia

Suraya A. Husin [1], Yew Fong Lee[1, 2], Noor Amelia Abd Rashid [1], Affaf Azizan[1], Puteri Fajariah[1], Nor Farah Bakhtiar[1], Suhaily Othman[1], Rohana Omar[1]
[1]Ministry of Health, Kuala Lumpur, Malaysia; [2]Institute of Global Health and Infection Control Program, University of Geneva Hospitals and Faculty of Medicine, Geneva, Switzerland
**Correspondence:** Yew Fong Lee
*Antimicrobial Resistance and Infection Control* 2017, **6(Suppl 3):**P86

**Introduction:** In 2005 the World Health Organization (WHO) launched the First Global Patient Safety Challenge, "Clean Care Is Safer Care", with the objective to reduce Healthcare Associated Infections (HCAI) and cross transmission of antimicrobial resistant (AMR) pathogens. On 15$^{th}$ May 2006, Malaysian Minister of Health pledged and signed a statement of commitment towards this First Global Patient Safety Challenge and has since adopted the WHO's multimodal HH improvement strategies.
**Objectives:** To assess the trend of HH compliance rate after the implementation of WHO's multimodal HH improvement strategies and to associate the trend of HH compliance rate with the trend of HCAI prevalence rate in Malaysia.
**Methods:** 14 public state hospitals (tertiary hospitals) throughout Malaysia were involved in the WHO multimodal HH improvement strategies implementation. The strategies were based on WHO's recommendation that included 'adoption of alcohol based hand rub', 'education', 'surveillance', 'facility improvement', 'reminders at workplace', 'commitment from stakeholders' and 'leadership by example'. The trend of HH compliance and HCAI rates were obtained from 2008 to 2015. The HH compliance rates were measured quarterly using the WHO's "Your 5 Moments for Hand Hygiene" audit tool. The HH opportunities observed for these hospitals were based on the number of patient beds (range: 400 to 750 HH opportunities) for every quarter. The total HCAI rate and types of common infections were also observed six monthly throughout this 8-year period via a hospital-wide point prevalence survey.

**Results:** Overall HH compliance rate has shown gradual increment from 56.6% in 2008 to 79.8% in 2015. The HCAI prevalence rate over these years has progressively reduced from the baseline of 3.2 to 1.6 per 100 patients surveyed in 2008 and 2015 respectively. The 41% increase in HH compliance rate corresponded to the 50% reduction of HCAI prevalence rate.

**Conclusion:** The implementation of the WHO's multimodal HH improvement strategies has successfully increased the HH compliance. The increased HH compliance was associated with the reduction of HCAI prevalence in Malaysia.

**Disclosure of Interest**

None Declared

### P87

#### Hand Hygiene Self-Assessment Framework (HHSAF) survey of 23 hospitals in Sarawak, Malaysia

Yew Fong Lee[1,2], Suraya A. Husin [1], Walter Zingg[2], Hock Hin Chua [3], Mary W. Ago [3], Fatin A. Ibrahim [3], Loke Meng Ong [1], See Yin Wong [3], MaryLouise McLaws[4], Didier Pittet [2]

[1]Ministry of Health, Kuala Lumpur, Malaysia; [2]Institute of Global Health and Infection Control Program, University of Geneva Hospitals and Faculty of Medicine, Geneva, Switzerland; [3]Sarawak General Hospital, Kuching, Malaysia; [4]University of New South Wales, Sydney, Australia

**Correspondence:** Yew Fong Lee

*Antimicrobial Resistance and Infection Control* 2017, **6(Suppl 3):**P87

**Introduction:** Located in South-East Asia, Sarawak is one of a Malaysian state of Borneo with 2.6 million population whose income is middle to high. In March 2017, 23 public healthcare facilities in Sarawak participated in a survey to monitor the World Health Organization (WHO) Hand Hygiene Self-Assessment Framework (HHSAF).

**Objectives:** To obtain a situation analysis of HH promotion and practices within an individual health-care facility; to reflect the existing resources and achievements in HH.

**Methods:** The state of Sarawak offers a total of 3978 patient beds with one ID physician for the state and 38 IPC nurses (1 per 105 beds in average). Infection control professionals from 23 public health care facilities were invited to email their HHSAF survey results to the state IC unit. Based on the facility's score, it is allocated to one of the four levels of progress within the HH improvement continuum.

**Results:** Of the 23 surveyed public healthcare facilities, four were major specialist hospitals, five minor specialist hospitals, 11 non-specialist hospitals and three special care institutes (psychiatric, leprosy and heart). The overall mean score indicated an advanced level of progress (score 434) as defined by the WHO HHSAF. Most facilities were at intermediate (26%) or advanced levels (74%) of progress. The average score for leadership criteria in 17 advanced healthcare facilities was 17 (range: 9-20). 18% of the advanced level facilities did not qualify for the leadership level due to the lack of 'Reminders in the workplace'. The lowest mean score (score 333) was recorded in a non-specialist hospital while the highest was recorded in a major specialist hospital (score 498). The lowest scores were identified for the 'Institutional safety climate for hand hygiene' and the 'Reminders at workplace'.

**Conclusion:** The survey helps to ensure that the necessary infrastructure is in place to allow health-care workers to practice HH. An innovative state-wide hand hygiene poster competition will be held during in May 2017, in parallel to the WHO Hand Hygiene Day in healthcare (5$^{th}$ May 2017); posters will be used as reminders in these facilities.

**Disclosure of Interest**

None Declared.

### P88

#### Hand hygiene levels of Philippine Hospitals: an application of the World Health Organization (WHO) Hand Hygiene Self-Assessment Framework

Nicolo Andrei A. Añonuevo

INFECTION PREVENTION AND CONTROL, ASIAN HOSPITAL AND MEDICAL CENTER, Alabang, Philippines

*Antimicrobial Resistance and Infection Control* 2017, **6(Suppl 3):**P88

**Introduction:** The caring hands of the healthcare workers play a significant role for the transfer of the opportunistic microorganisms, thus the development of HAIs. With this, the patient is at risk at the presence of a healthcare worker who should provide the best care.

**Objectives:** This study is a pilot attempt in the Philippines to determine the level of compliance on hand hygiene based on the Hand Hygiene Self-Assessment Framework of the World Health Organization.

**Methods:** This study utilized descriptive-evaluative, descriptive comparative, and descriptive-correlational research designs and looked into the level of compliance to hand hygiene among the respondents using purposive sampling in NCR Philippines. Standard Hand Hygiene Self-Assessment Framework adapted from WHO was utilized on this study.

**Results:** Majority of the respondents were registered nurses with the Degree of Bachelor of Science in Nursing with 38 percent or 40 out of 106. In addition, based on category, majority of the respondents are full time Infection Prevention and Control Nurses with 71 percent or 75 out of 106 with length of hospital experience ranging from 1-5 years. Based on location, majority of the hospitals are located in Quezon City with 23 percent or 24 out of 106 and these hospitals are Level 3, General Hospitals with 30 percent or 32 out of 106. Majority of these hospitals are Philhealth accredited and privately owned with 69 percent or 71 out of 106.

The level of compliance to HHSAF in terms of System Change, Education and Training, Evaluation and Feedback, Reminders in Workplace, and Institutional Safety Climate is Intermediate Hand Hygiene Level which means that appropriate hand hygiene promotion and strategy is in place and hand hygiene practices have improved in most of the hospitals who participated on this study.

**Conclusion:** 1. Majority of the hospitals have designated full time infection prevention and control nurse.

2. ALcohol based hand rubs are not available at the foot part of the bed.

3. Majority of the hospitals do not have single use paper towelss

4. Majority of the hospitals hand hygiene compliance are irregularly monitored.

75. Majority of the hospitals do not have a system to check hand hygiene champions.

**Disclosure of Interest**

None Declared

### P89

#### The development and effect of the education program on hand hygiene and use of personal protective equipment

Ihnsook Jeong, Eun-gyeong Kim

Pusan National University, Yangsan, Korea, Republic Of

**Correspondence:** Ihnsook Jeong

*Antimicrobial Resistance and Infection Control* 2017, **6(Suppl 3):**P89

**Introduction:** Nepal is a developing country in which HAIs pose a major problem in terms of patient safety. In such an environment with a high necessity of HAI control, there is almost no training or study on HAI control for nurses

**Objectives:** The purpose of this study was to develop a education program for nurses in Nepal on hand hygiene and use of personal protective equipment based on ADDIE (analysis, design, development, and implementation) model and ARCS (attention, relevance, confidence, and satisfaction) model and verify its effects.

**Methods:** The education program was developed in the stages of ADDIE's analysis, design, development, and implementation applying four major subdomains of ARCS; attention, relevance, confidence, and satisfaction. For effectiveness evaluation a time series, randomized control group pretest-posttest study was used. The subjects of this study were nurses working at L university hospital in western development region, Nepal, and they were randomly assigned to an experimental group (34 subjects) or a control group (34 subjects). Data were analyzed using descriptive statistics, $\chi 2$-test, t-test and repeated measures ANOVA with the SPSS 23.0 program.

**Results:** The experimental group showed significant improvements after intervention on all variables such as accuracy of hand hygiene ($p < .001$) and accuracy of wearing ($p < .001$) and removal of personal protective equipment ($p < .001$). Learning motivation of the experimental group was as high as $4.19 \pm 0.27$ points.

**Conclusion:** The training program on hand hygiene and personal protective equipment use had a positive effect on the accuracy of hand hygiene performance and personal protective equipment use by the nurses in Nepal. Therefore, this study suggests utilizing the education program for nurses working at medical institutions.

**Disclosure of Interest**

None Declared

### P90

#### Rising hand hygiene compliance in a public hospital through the implementation of the World Health Organization multimodal strategy

Alejandra Ramirez, Veronica Villalobos
Infection control and quality, Hospital de la Serena, La Serena, Chile
**Correspondence:** Alejandra Ramirez
*Antimicrobial Resistance and Infection Control* 2017, **6(Suppl 3):**P90

**Introduction:** Despite of its importance, infection control nurses usually struggling to maintain health care workers motivated and sensitized about hand hygiene performance**.**

**Objectives:** Our aim was to establish the impact in hand hygiene compliance with the implementation of the Multimodal Strategy.

**Methods:** The Multimodal Strategy in hand hygiene has been implementing in our hospital since 2013. 1250 health care personnel work in this 284 bed-hospital.Self-assessment questionnaire was applied. The level of hand hygiene achieved was BASIC. An action plan was developed and implemented. **SYSTEM CHANGE**: High quality alcohol-based hand rub (ABHR) was acquired. Dispensers were installed at the point of care. Hand lotion was also available. **EDUCATION AND TRAINING:** hand hygiene training was performed. Technique was assessed based on ATP Bioluminescence and black -box. Hand hygiene videos were developed by different units. Full commitment and creativity were awarded. **MONITOR AND FEEDBACK**: Hand hygiene compliance were monitored. The results were communicated and improvements implemented. **REMINDERS IN THE WORKPLACE:** local creativity was deployed in the new hospital- made posters. The activities were promote inside and outside the hospital by a local newspaper's support. **INSTITUTIONAL SAFETY CLIMATE:** Hospital Board committed with this initiative.

**Results:** In 2013, the level of hand hygiene of this facility was BASIC. Hand hygiene compliance was unknown. In 2016 the hand hygiene level was INTERMEDIATE and the hand hygiene compliance was 76%.

**Conclusion:** The hand hygiene self-assessment tool was a useful tool to determine objectively the improvement opportunities of our hospital. The hand hygiene multimodal strategy has been helping us to provide sustainability of hand hygiene program in a hospital characterized by budget constraints, over demand of attention and inappropriate infrastructure.The 2017 challenge is to incorporate patients and its families.

**Disclosure of Interest**

None Declared.

### P91

#### Utility of the multimodal strategy in hand hygiene: the experience of a public hospital in Chile

Karen Ulloa[1], Ana Seguel[1], Claudio Vargas[1], Viviana Muñoz[2], Naldy Febré[3]
[1]Programa de prevención y control de IAAS, Hospital de Urgencia Asistencia Pública; [2]Academia Aesculap; [3]Programa de Doctorado en Enfermería., Universidad Andrés Bello, Facultad de Enfermería, Santiago, Chile
**Correspondence:** Karen Ulloa
*Antimicrobial Resistance and Infection Control* 2017, **6(Suppl 3):**P91

**Introduction:** Hand hygiene is well-know as the most important measure to prevent health care associated infections.

**Objectives:** To establish the impact in hand hygiene compliance with the implementation of the Multimodal Strategy.

**Methods:** Prospective study of seven months duration. The services analyzed were: intensive and intermediate units, with a total of 70 staff in rotating shifts of 12 hours. Implementation was divided in three stages. Stage 1: the WHO observation form and self-assessment questionnaire were applied in order to know basal hand hygiene compliance and level of hand hygiene in this facilities. Stage 2: Data was analyzed and communicated. Diverse interventions were implemented. Stage 3: a post-intervention evaluation was accomplished conducive to determine impact in hand hygiene compliance. The ethical standards internationally required were followed.

**Results:** During the intervention period, 100% of planned activities were performed, such as: 1- Baseline compliance in hand hygiene was 53%. 2- The level of hand hygiene assigned was INSUFFICIENT. System change: a new formulation of alcohol-based hand rub was provided, dispensers were installed at the point of care, cream was available, education and training in hand hygiene. Compliance monitored and feedback of the results divided by professional category and moment were performed. Reminders at the workplace: new posters were designed. Institutional safety climate: project supported by the hospital board and medical and nursing chiefs. Multimodal Strategy managed to increase hand hygiene compliance by 34.8 percentage points when comparing the pre-intervention period (53%) versus the post-intervention period 87.8 evidenced by increased consumption of alcohol-gel.

**Conclusion:** Multimodal Strategy is a useful tool to promote compliance in hand hygiene, increasing the adhesion by the health personnel.

**Disclosure of Interest**

None Declared

### P92

#### Effectiveness of regular feedback in improving hand hygiene compliance among doctors and nurses in a tertiary care hospital in India

Kanchan Raghuwanshi, Smita Sarma, Sharmila Sengupta, Yatin Mehta, Pankaj Sahni
Microbiology and Infection Control, Medanta- The Medicity, GURGAON, India
**Correspondence:** Kanchan Raghuwanshi
*Antimicrobial Resistance and Infection Control* 2017, **6(Suppl 3):**P92

**Introduction:** Adherence to hand hygiene practices especially among doctors and nurses always pose a challenge to quality care and patient safety. Low compliance to hand hygiene among healthcare professionals is contributed by behavioural, attitude and knowledge components. Also availability and appropriateness of resources contribute to low compliance for hand hygiene practices.

**Objectives:** This study aims at exploring the impact of regular feedback mechanism on improving hand hygiene compliance among doctors and nurses

**Methods:** This study was conducted to observe the hand hygiene compliance of doctors and nurses using the WHO hand hygiene tool for duration of 3 months. Resources were made available in each nursing unit, bedside and outside the patient cubicle before the start of study.

Shadow audits were conducted in 17 inpatient ward and 11 Intensive Care Units.
A feedback format was designed including the following information:
1. Cumulative compliance of audited units
2. Compliance of staff according to specialty (medical/ surgical)
3. List of compliant doctors and nurses
4. List of non compliant doctors and nurses
5. Most missed/ compliant moment of hand hygiene
The feedback was shared via mail and hard copy to specialty heads and the hospital chief medical director (CMD). The speciality heads later shared the data with their team members.
**Results:** By the end of the first month the doctors showed a cumulative increase in hand hygiene compliance by 11% whereas the nurses showed an increase in compliance by 13%. By the end of third month doctors showed further increase in compliance by 9% and nurses by 11% thus improving the overall compliance to 72% in doctors and 75% amongst nurses.
**Conclusion:** The feedback mechanism showed success in terms of self awareness and consciousness to perform hand hygiene among doctors and nurses. Doctors and nurses both gave encouraging response to the feedback mechanism This study emphasise the importance of regular feedback mechanism (Top to Bottom approach) in creating a healthy competition, self corrective attitude and positive peer pressure without creating embarrassment or negative attitude towards the process. In a developing country with minimal resources, this simple method proved effective and sustainable.
**Disclosure of Interest**
None Declared

### P93

#### Increased hand hygiene compliance following hospital hygiene measures implemented after an epidemic of Lassa haemorrhagic fever in a network of hospitals republic of Benin

Charlemagne Degan
ADEQSS, COTONOU, Benin
*Antimicrobial Resistance and Infection Control* 2017, **6(Suppl 3):**P93

**Introduction:** The safety care in african hospitals is not the priority of the health system. This situation expose users to infectious risk and lead to high rate of morbidity and mortality within the hospitals. The Lassa haemorrhagic fever epidemic in two hospitals in northern of BENIN had been an opportunity to introduce control measures and prevention infection, particulary in hand hygiene into a network of hospitals. This network about eigth hospitals wich have the same geographic characteristics are concerned.
**Objectives:** Increase the compliance rate for hand hygiene from 15% to 40%.
Reduce the risk of hand infection transmission during healthcare.
**Methods:** The strategy implemented to improve hand hygiene was based on five actions
- Clinical audit on hand hygiene on the input's existence; Mastery of hand hygiene technics; and opportunities. Direct observations and questionaries are the tools used for this study.
- Generalization of use the hydroalcoholic solution in all health workers
- Organization of awareness-raising and training campaigns
- Reporting audits results to hospital's teams
- Continued learning and performance monitoring.

**Results:** Equipment of 100% of the care services in mobile hand washing station and then increase the quantity of the hydro alcoholic solution.
- 1248 moment for hand hygiene were observed.
- 350 sessions of observation followed by formative restitution
- Increase in the overall compliance rate from 15% to 45% from 2014 to 2016
- The proportion of correct alcoholic hand rubbing was 61% of the cases.
- A national programme had been put on to manage infection prevention in all the hospitals in the country.

**Conclusion:** This epidemic revealed the weakness of the health system in our country. The situation has been an opportunity to set up a national program to fight
against infections associated to healthcare. But it must be a long fight to have best results in those hospitals.

**References**
1-world
Health Organization (W.H.O) Guideline on hand hygiene in health care GENEVA 2009
2-PITTET
D. Compliance with hand desinfection and its impact on hospitals acquired infections.
Disclosure of Interest
None Declared

### P94

#### Hand hygiene in hospitals in Benin three years after the institutionalization of patient safety

Alain K. Aissi, Ahoyo A. Théodora, Louis Affovehounde, Denis Sossa, Eléonore A. Gandjeto, Chabi Bagou Orou Yorou
Direction Nationale la Santé Publique, MINISTÈRE DE LA SANTÉ, Cotonou, Benin
**Correspondence:** Ahoyo A. Théodora
*Antimicrobial Resistance and Infection Control* 2017, **6(Suppl 3):**P94

**Introduction:** A unit was created in 2013 at the National Directorate of Public Heath to operationalize the commitments made by Benin after the 1st International Conference of Health Ministers of Africa on Patient Safety.
**Objectives:** Assess the evolution of hand hygiene promotion in Benin's health system
**Methods:** Interview with hospital managers, members of committees of fighting against Nosocomial infections and supervisors in three technical directorate from the Ministry of Health. Observation in 24 public and private hospitals between October 2016 and March 2017. Hand Hygiene Self-Assessment Questionnaire according to the WHO Multimodal Strategy. Data processing by content analysis and Excel based on the system of assignment efficiency scores in percentage from 0 to 100%.
**Results:** The number of hospitals enrolled in Save Yours Live Clean hands (SLCYH) increased from 01 in 2013, 28 in 2015 and 34 in 2016. This number (34) represents 68% of Benin hospitals. Staff awareness of hand hygiene is done at least once a year in each hospital, apart from actions related to the World Day of 05 May, taken into account in the budget of the Ministry of Health. The average rate of adherence to hand hygiene remains low in 90% of hospitals with the following major reasons: poor availability of washbasins, irregularity of hydroalcoholic products (PHA) in several services; High cost of imported PHA causing frequent supply disruptions by hospital managers.
**Conclusion:** Despite its fourth position among countries in the WHO Afro region with the largest number of hospitals enrolled in SLCYH, the level of hand hygiene practice in Benin varies from inadequate to basic. The Ministry of Health must be supported to implement its IPC National plan which was adopted in 2016 and which places a high priority on promoting hand hygiene compliance
**Disclosure of Interest**
None Declared

### P95

#### Hospital hand hygiene in the DR Congo: a survey of healthcare workers' knowledge, attitudes and practice

Elie K. Kabululu[1], John K. Mutendela[2], Gillian Ray-Barruel[1], Naomi Kyakimwa Mukono, Zawadi Vulwage, Paluku Muvunga, Jeanne-Marrie Kyavalanga, Silewa Zombo, Lumunya Essombo, Baraka Mandro, Bahenduka Dubatso
[1]CENTRE MEDICALE EVANGELIQUE DE NYANKUNDE / EQUIPE MEDICALE DES VOLONTAIRES, BENI, Congo, The Democratic Republic of the; [2]CENTRE MEDICALE EVANGELIQUE DE NYANKUNDE / EQUIPE MEDICALE DES VOLONTAIRES, BENI, France
**Correspondence:** Elie K. Kabululu
*Antimicrobial Resistance and Infection Control* 2017, **6(Suppl 3):**P95

**Abstract video clip: Introduction**

Hospitals in the DR Congo care for patients with extensive traumatic wounds, advanced cancer, and obstetric emergencies, among others. With minimal healthcare resources, healthcare associated infection is often deadly. We carried out a cross-sectional survey to evaluate health professionals' knowledge of handwashing indications (in relation to the 5 WHO standard indications) and to analyze the factors that may influence the practice of hand hygiene.

**Methods**

The researchers developed a short, anonymous survey to examine healthcare workers' knowledge, attitudes and practices of hand hygiene in one hospital in the DR Congo in order to develop a targeted educational and interventional strategy for improving infection prevention and control.

**Results**

Thirty-one respondents completed the survey. Healthcare workers' knowledge of the indications for handwashing were closely (90%) aligned with the WHO standards. More than 90% consider that hand transmission of nosocomial infections and hand hygiene is a major concern in the care setting. Between 60%–80% reported that they wash their hands regularly, have enough time to wash their hands, and believe their colleagues demonstrate good handwashing practice. However, 60% believe that the equipment available to guarantee handwashing is insufficient at their place of work. Alarmingly, almost one-third of respondents consider that by regularly washing their hands they risk developing skin lesions.

**Conclusions**

The majority of healthcare workers in this hospital are aware of the indications for handwashing and report compliance with the WHO standards. However, factors such as inadequate resources and the fear of developing skin lesions from frequent handwashing remain a concern. Education training sessions and practical strategies are being implemented in this hospital to address the prevention of healthcare associated infection. Sessions have been very well attended, and ongoing audits continue to measure patient outcomes.

**Disclosure of Interest**

None Declared

### P96

#### Hand hygiene compliance during labour and delivery in maternity units in Zanzibar

Giorgia Gon[1], Said M. Ali[2], Marijn de Bruin[3], Oona Campbell[4], Mícheál de Batta[3], Loveday Penn-Kekana[4], Sandra Virgo[4], Susannah Woodd[4], Wendy J. Graham[4] on behalf of HANDS study group
[1]Infectious Disease Epidemiology, London School of Hygiene and Tropical Medicine, London, United Kingdom; [2]Pemba Health Laboratory – Ivo de Carneri Foundation, Chake Chake, Tanzania, United Republic of; [3]University of Aberdeen, Aberdeen; [4]London School of Hygiene and Tropical Medicine, London, United Kingdom
**Correspondence:** Giorgia Gon
*Antimicrobial Resistance and Infection Control* 2017, **6(Suppl 3):**P96

**Introduction:** Hand hygiene is necessary during birth to prevent newborn and maternal infection.

**Objectives:** This study aimed to assess hand hygiene compliance and its determinants among birth attendants in the 10 high-volume maternity units in Zanzibar

**Methods:** Data were collected on hand hygiene and its determinants between March and December 2016. In four facilities, we conducted unstructured observation and 54 qualitative interviews with health professionals. The quantitative phase included longitudinal observation of all birth attendants *in situ* in each of the 10 facilities and a questionnaire with each of them (N = 107). We calculated the mean hand hygiene compliance using different definitions. We used thematic analysis to analyse the qualitative data, and multi-level logistic regression models to analyse the quantitative data

**Results:** Using the World Health Organisation definition of hand hygiene which includes avoiding unclean touches between handwashing/rubbing and a clinical procedure, the mean compliance before delivery was 11.6%; when allowing for unclean touches this rose to 30.1%. The relative importance of psychological and context variables in explaining compliance are different for the two hand hygiene definitions. Descriptive norms seem associated with the second definition (OR = 1.12,CI:0.99-1.28,p-value = 0.069), whilst not with the first (p-value = 0.305). From the qualitative results, an efficient physical layout of the ward seems to explain poor compliance to the first but not the second definition.

**Conclusion:** Compliance to hand hygiene during birth was low in this context. This study suggests that handwashing/rubbing and avoiding unclean touches are two distinct behaviours, and that both individual and environmental factors influence these behaviours.

**Disclosure of Interest**

None Declared

### P97

#### Hand hygiene compliance among healthcare workers in Liberia: hand hygiene audit result

Theodosia S. Kolee
Infection Prevention and Control, WORLD HEALTH ORGANIZATION-LIBERIA, Monrovia, Liberia
*Antimicrobial Resistance and Infection Control* 2017, **6(Suppl 3):**P97

**Introduction:** With Liberia being declared Ebola Virus Disease free in June 2016, the focus of Infection Prevention and Control (IPC) shifted from additional precautions to ensuring adherence of standard precautions; of which hand hygiene is a key component. This paper reports on the first ever national hand hygiene compliance status among hospital healthcare workers, conducted from August 2016-February 2017. Liberia has 15 counties and each county has a public referral hospital which is where the audits were undertaken.

**Objectives:** The objective of the audit is to assess and evaluate Healthcare workers' (HCWs) hand hygiene compliance, as it has been reported that 80% compliance significantly reduces Healthcare Associated Infection (HCAI).

**Methods:** Collection of data was done using the Hand Hygiene standardized observational form through direct observation of healthcare workers; number of times hand hygiene was done, and the timing and technique of hand hygiene were undertaken. It took 20 minutes' duration per HCW, on different wards, and at different times of the day (morning, afternoon, night shifts). The assessment team comprises of; hospital IPC Focal person, county IPC Focal person(s), Academic Continnum Combating Ebola in Liberia (ACCEL) team and WHO IPC Focal person.

**Results:** Total number of Healthcare Facilities (HCFs) audited: 29 out of 41 (71%) hospitals in Liberia. Result from the audit shows that the national hand hygiene compliance is 45% (this is based on the results of the 15 counties). The best performing county is Grand Cape Mount County which has a compliance rate of 86% while the lowest performing County is RiverCess at 20%, hence it is noted that there is a wide spread in compliance, although the reasons for this are unknown. Hand Hygiene compliance comparing private to public hospital was 40% to 49% respectively. When comparing hand hygiene before and after patient contact; 11% before and 34% after. Some of the observations made during the audit were; hand wash (HW)/hand rub (HR) technique and the appropriate duration of HR/HW were not followed.
**Conclusion:** The national hand hygiene audit result of 45%, gives the first insight into hand hygiene compliance based on actual data. These results are now being used to guide healthcare facilities and policy makers in developing strategies to improve hand hygiene practices among healthcare workers in Liberia.
**Disclosure of Interest**
None Declared

### P98

#### Hand hygiene self-assessment framework: baseline assessments to determine hand hygiene practice level and facilitate planning for improvement in healthcare facilities in Liberia

Garrison Kerwillain
Quality Management Unit, Ministry of Health Liberia, Monrovia, Liberia
*Antimicrobial Resistance and Infection Control* 2017, **6(Suppl 3):**P98

**Introduction:** Liberia is divided into 15 political divisions known as counties with a total estimated population 4.2 million (2016 SARA report). There are approximately 770 health facilities (subcategorized into hospitals, health centers, and clinics); of which 41 are hospitals. There are 80% (616/770) public facilities and 20% (154/770) private/faith based facilities. The Ebola outbreak highlighted the need for universal standard precautions, specifically the importance of hand hygiene compliance in reducing Health Care Associated Infections (HCAI).
**Objectives:** To identify hand hygiene framework gaps among healthcare workers, and help in developing an action plan.
**Methods:** The standardized WHO HHSAF was adopted and adapted to the Liberian context, however without affecting the scoring system. The methodology consisted of identifying the county referral hospitals to assess, informing senior facility managers prior to visit and sharing the template with them. The assessment team included the Ministry of Health (national and county level), hospital senior managers, and WHO county IPC focal person. Once at the facility a Conference and open discussion were held with these key stakeholders and the HHSAF was completed with consensus.
**Results:** Eight HHSAF assessments were undertaken in eight hospitals in four counties. The HHSAF average for the eight facilities was 52% (2070/4000) which correlates to intermediate hand hygiene level; however, there was a wide range between the best performing facility (70%) compared to the worst performing (35%). When comparing the five HHSAF components, institutional safety climate was the best performing with 60%, and evaluation and feedback was the worst performing area with 42%. In relation to ownership, the four public facilities performed better with an average of 54% compared to the four-private facilities average of 41%.
**Conclusion:** This HHSAF assessment is conducted for the first time in Liberia; showing an average intermediate hand hygiene level for the facilities assessed. These results are being used to develop facility based action plans to address identified gaps. Reassessments are planned for within six months.
**Disclosure of Interest**
None Declared

### P99

#### Evaluating the effectiveness of an intervention program to improve hand hygiene compliance in a Tunisian University Hospital

Sihem Ben Fredj, Hela Ghali, Mohamed Ben Rejeb, Salwa Khefacha, Houyem Said Latiri
Prevention and Security of Care, University Hospital Of Sahloul, Faculty of Medecine of Sousse, Sousse, Tunisia
**Correspondence:** Sihem Ben Fredj
*Antimicrobial Resistance and Infection Control* 2017, **6(Suppl 3):**P99

**Introduction:** The transmission of healthcare-associated pathogens from patient to another and within the healthcare environment is mainly via the hands of healthcare workers. The Hand Hygiene (HH) is considered the most important measure, because of its proven efficiency, its effectiveness, and its low cost, but compliance with recommendations is usually low and effective improvement strategies are needed.
**Objectives:** to assess the effectiveness of a 1-year health-setting intervention targeting hand hygiene promotion.
**Methods:** We conducted a prospective before-and-after study design in university hospital Sahloul, Sousse, Tunisia, from January 2015 to December 2016. The intervention program consisted of training sessions and distribution posters of hand hygiene guidelines. To assess the knowledge evolution at pre and post intervention, the same questionnaire was distributed and collected at HCWs' workplace. Another questionnaire was used to evaluate the HH observance.
**Results:** Globally, our results showed tendencies toward improvement, but only two items were significant: the HW is the appropriate technique after injury (73.5% vs 86.8%; $p = 0.006$) and the hands of HCWs are the lead vehicle of HAIs (65.1% vs 83.6%; $p = 0.01$). Judging by the HCWs' responses, the probably most effective intervention to enhance hand hygiene compliance was that an alcohol-based hand-rub is available at each point of patient care. On the other hand, of the 1201 and 1057 opportunities for hand hygiene observed among all categories of HCWs, overall compliance enhanced significantly from 31.6% to 40.4% ($p < 0.001$) respectively at pre and post intervention.
**Conclusion:** This study showed the feasibility and effectiveness of a health-setting-based intervention to enhance hand hygiene observance in the context of a developing country.
**Disclosure of Interest**
None Declared

### P100

#### On the spot hand hygiene and injection safety assessment in a tertiary hospital in Nigeria

Kenneth I. Onyedibe[1], Nathan Shehu[2], Rachel Odesanya[3], Mark Okolo[1], Simji Gomerep[2], Yadang Maktep[1], Peter Pama[1], Peter Mbigurum[3], Gabriel Shut[2], Samson Isa[2], Daniel Egah[1], Daniela Pires[4], Didier Pittet[4]
[1]Medical Microbiology; [2]Medicine; [3]Pharmacy, Jos University Teaching Hospital, Jos, Nigeria; [4]Infection Control Unit, University of Geneva Hospitals, Geneva, Switzerland
**Correspondence:** Kenneth I. Onyedibe
*Antimicrobial Resistance and Infection Control* 2017, **6(Suppl 3):**P100

**Introduction:** Health-care-associated infections (HAIs) are important cause of morbidity and mortality. HAIs in Africa are two to twenty times higher than in developed countries. In Nigeria, prevalence of HAIs range between 2.6 and 30.9%. Poor Infection Prevention and Control (IPC) facilities largely contribute to this problem and it is therefore needed to assess its availability and functionality in African countries
**Objectives:** We aimed to perform a survey on hand hygiene and injection safety facilities available at a tertiary Nigerian hospital
**Methods:** This cross-sectional study was carried out during a seven day period from 19th to 25th November 2013 at a 600 bed tertiary health facility in North central Nigeria. Assesors were trained on how to assess hospital infection facilities and collect data using a modified CDC facility assessment tool.
**Results:** Thirty six units were assessed out of the fifty units of the Hospital (72%); these units comprised wards (19), out-patient clinics(5), canteens (3), Labs (2), operating theatres(1), delivery suites(1), mortuary (1), dialysis (1), laundry (1), Dietetics (1) and physiotherapy units (1). Fifty two percent of the units had no poster or written policy on hand hygiene and 94% didn't have alcohol based hand rubs available. About 89% of the units had at least one hand washing station. Of these, 22% had running tap water all day and 78% utilized cup and bucket. 75% used bar soap, but this was always availlable only in 63% of the units. 43% of the units had no hand drying facilities. The main water source was municipal water (69%), but also municipal water augmented with tanker (11%) and surface or well water (9%). Only 11% had written policy and posters on injection safety and 22% had self-retracting needles. 79% of the units had appropriate containers for disposal of sharps and 45% of the containers are disposed when they are three quarters full.

**Conclusion:** Facilities for hand hygiene and injection safety are sub-optimal in the hospital. Measures to improve the availlability of these facilities are greatly needed to improve IPC practices.

**Disclosure of Interest**

None Declared

### P101

#### Assessment of hand hygiene compliance among health care workers in a Nigerian Tertiary Hospital

Nathan Y. Shehu[1], Kenneth Onyedibe[2], Mark Okolo[2], Simji Gomerep[1], Samson E. Isa[1], Comfort Ibrahim[3], Sunday Igboanugo[4], Adebola Olayinka[5], Daniel Egah[2], Daniela Pires[6], Didier Pittet[6]

[1]Internal Medicine; [2]Medical Microbiology; [3]Nursing Department; [4]Pharmacy, Jos University Teaching Hospital, Jos; [5]Medical Microbiology, Ahmadu Bello University Teaching Hospital, Zaria, Nigeria; [6]Infection Control Programme & WHO Collaborating Centre on Patient Safety, The University of Geneva Hospitals and Faculty of Medicine, Geneva, Switzerland

**Correspondence:** Nathan Y. Shehu

*Antimicrobial Resistance and Infection Control* 2017, **6(Suppl 3):**P101

**Introduction:** The burden of healthcare-associated infection (HCAI) is 2 to 18 times higher in developing than in developed countries. Good hand hygiene practices reduce healthcare associated infections. However, very few data is available regarding factors influencing compliance to hand hygiene in developing countries.

**Objectives:** To evaluate hand hygiene compliance amongst healthcare workers (HCW) in a tertiary hospital in Jos, Nigeria.

**Methods:** Hand hygiene compliance assessment was conducted using the World Health Organization (WHO) direct observation method. The observed compliance rates in the different hospital units and amongst different categories of HCWs was calculated and analyzed using Epi-Info version 3.5.1. The correlates of compliance were grouped and listed. A P value of <0.05 was considered significant.

**Results:** One hundred and seventy five (175) HCWs were observed. Fifty-four (30.9%) were ward attendants, 13 (7.4%) medical students, 42 (24%) nurses, 21 (12%) nursing students, 18 (10.3%) pharmacists and 27 (15.4%) doctors. Four hundred and six (406) hand hygiene opportunities were observed and the overall compliance rate was 31%. The compliance rates varied from a maximum of 82% amongst medical students to a minimum of 18% amongst ward attendants. Based on indication, compliance rates were: 21% before patient contact, 41% after patient contact, 23% before aseptic procedure, 40% after contact with patients' surrounding and 63% after body fluid contact. Being a medical student was the only factor independently associated with good hand hygiene compliance.

**Conclusion:** Hand hygiene compliance in the setting of a large tertiary hospital in Nigeria is low. Compliance before patient contact was lower than after patient contact or contact with patient surroundings and before body fluid exposure risk. Our findings confirm that HCWs seem more sensibilized to their risk of exposure to potential pathogens than to the prevention of transmission of HCAI. Specific measures like hand hygiene training, monitoring and patient driven prompting are needed.

**Disclosure of Interest**

None Declared

### P102

#### Infection prevention and control in non-healthcare settings: the role of hand hygiene-Oyo state, South West Nigeria 2017

Olatunji T. Badmus[1], Kehinde Omisile[2], Bunmi Bamgbade[3], Biodun Ogunniyi[4], Olukemi Olugbade[5]

[1]Enterprise Business Unit, Airtel Networks Limited, Lagos; [2]Epidemiology and Biostatistics; [3]Health education and Promotion, University of Ibadan, Ibadan; [4]Nigeria Centre for Disease Control (NCDC); [5]Nigeria Field Epidemiology and Laboratory Training Programme (NFELTP), Abuja, Nigeria

**Correspondence:** Olatunji T. Badmus

*Antimicrobial Resistance and Infection Control* 2017, **6(Suppl 3):**P102

**Introduction:** Recent studies have shown that several infections can be transmitted in the workplace. Many workers hardly wash hands when ill or not. Hands are responsible for the spread of 80% of common infectious diseases. Effective hand hygiene continues to be recognized universally as the safest, smartest, and most cost effective means of infection control. There is paucity of information on hand hygeine practices in non health care settings, especially in developing countries

**Objectives:** The objective of our study was to assess the knowledge, attitude and infection control practices among workers and identify barriers to hand hygiene in a non-healthcare setting in Nigeria

**Methods:** We carried out a descriptive cross sectional survey in a telecommunications company. Respondents were recruited using a systematic random sampling method. We used an adapted (electronic Android-based) interviewer administered questionnaire to collect information on socio-demographic characteristics, and a checklist was used to score on knowledge, attitude and practice of hand hygiene. Barriers to hand hygiene and infection control practices in the workplace were also identified

**Results:** We interviewed a total 34workers. Mean age of respondents was 34 ± 8.5 years, with 21 (62%) male and 13(38.2%) being senior cadre staff, mean years of working with the organization was 5.3 ± 4.4 years. A total 32(94%) had good knowledge of hand hygiene with mean score of 8.1 ± 1.4, 28 (88%) had good attitude to handwashing and hand rubbing and 10 (29%) had poor hand hygiene practices. There was no significant difference between cadre, persons who had organizational training on hand hygiene and those who had spent more than 5 years in the organization as regards hand hygiene practices ($p > 0.05$).The main barrier to hand hygiene in this setting was the frequency of hand hygiene required daily

**Conclusion:** Good knowledge of hand hygiene does not always translate to good practice. Hand hygiene promotion should be considered and implemented in non-healthcare settings where workers and staff need to observe good hand hygiene practices frequently.These will improve workplace infection control and limit transmission of work-related infectious diseases

**Disclosure of Interest**

None Declared

### P103

#### The hand hygiene journey of a small pediatric hospital to prevent hospital acquired infections in Palestine

Regeen Handal[1], Sister Lucia Corradin[1], Hiyam Marzouqa[2], Musa Hindiyeh[3]

[1]Patient Safety Office; [2]Medicine; [3]Laboratory, Caritas Baby Hospital, Bethlehem, Palestinian Territory, Occupied

**Correspondence:** Regeen Handal

*Antimicrobial Resistance and Infection Control* 2017, **6(Suppl 3):**P103

**Introduction:** Hand hygiene is a principal measure for preventing healthcare-associated infections (HAI's).

**Objectives:** In this study, we report the measures taken to improve health care providers hand hygiene compliance rates and the status of HAI's due to respiratory virus or bacterial blood stream at Caritas Baby Hospital (CBH) in Palestine.
**Methods:** CBH, a 90 bed hospital, has been monitoring HAI's and promoting a hand hygiene culture since 2008. Educative lectures of the 5-moments of hand hygiene were given to all healthcare providers and patients/families; posters of hand washing and hand rubbing were visible throughout the hospital. An active surveillance screening system for the presence of multi-drug resistant bacteria was implemented. Not only normal soap and antiseptic soap were accessible, but also alcohol-based hand rubs were available. Moreover, compliance with hand hygiene, was monitored through 20 spot-checks per month in the hospital's key departments. With the adaptation of an electronic Health Information System (HIS), the hospital's infection control team was able to collect precise data on both viral respiratory and bacterial blood stream HAI's.
**Results:** The overall hand hygiene compliance among healthcare providers was 92%. The overall respiratory viral HAI's (RSV, Rotavirus, Influenza, adenovirus and hMPV) during the same time period was 4.0/1000 hospitalization days. Stratifying the HAI's by month showed that during the winter respiratory season the rate ranged between 5.5-7.5/1000 HD's. The viral respiratory HAI's were mainly seen in the pediatric units and were less prevalent in the neonatal-ICU. On the other hand, the healthcare associated blood stream infections (BSI's) rate was 0.4/1000 hospitalization days, mainly seen in ICU patients and caused by multidrug resistant bacteria.
**Conclusion:** There is a remarkable steady progress and commitment by the staff to follow a hand hygiene culture. This in part is attributed to the administrative support and staff commitment to provide the safest patient care because "Clean Hands Count". Our future goal is to reduce the overall percent of respiratory viral HAI's to lower levels.
**Disclosure of Interest**
None Declared

### P104

#### Hand hygiene pilot test at the Mexican social security institute

Carmen Fernandez
Finance, Instituto Mexicano del Seguro Social, Mexico city, Mexico
*Antimicrobial Resistance and Infection Control* 2017, **6(Suppl 3):**P104

**Introduction:** This paper presents from the financial and operational point of view, the results of pilot test of a hand hygiene project at the Mexican Social Security Institute (IMSS).
**Objectives:** To explain the obstacles and the possible solutions to increase this practice, a pilot project was developed at four hospitals. The project's goals are to prove the following statements:
- That Nosocomial Infections (NI) I can be reduced with proper hand hygiene.
- That the obstacles for the staff engagement to the hand hygiene strategy can be removed.
- That having an excellent service and state of the art products is cost effective, regardless of the highest price in comparison with the low cost services and products.

**Methods:** With participation of professionals from different departments and backgrounds, the project's main task was to ensure that the hospitals would have full and continuous supply of alcohol based solution (ABS), chlorhexidine, liquid soap and paper towels. External providers were hired for the first two items, and the latter were managed internally.
In order to compare providers, three different companies participated. Their contracts included training and supervision of the hand hygiene technique, in addition to the continuous supply of ABS and chlorhexidine. The test began on November 2015 and will end on March 2017.
**Results:** The figures as of December 2016 show that the success of the project has been undeniable. For each peso IMSS invested, the return was 8 pesos. Apart from the financial gains, there are other positive outcomes:
- Infections were reduced by half.
- The consumption of ABS and chlorhexidine doubled.
- All hospital staff, medical and administrative, was trained and was engaged in the project.

**Conclusion:** IMSS is planning to continue the project, and extend it to other 6 additional hospitals during 2017 and 2018.
**Disclosure of Interest**
None Declared

### P105

#### Place of hydro-alcoholic hand-rub products in city care

Narbey David, Bouget Stéphanie, Chassy Sylvie, Savey Anne, Fascia Pascal
ARLIN RHONE-ALPES, [2]CCLIN SUD EST, SAINT-GENIS-LAVAL, France
**Correspondence:** Narbey David
*Antimicrobial Resistance and Infection Control* 2017, **6(Suppl 3):**P105

**Introduction:** In 2015, the French hospital hygiene society issued recommendations on good basic hygiene practices for the use of city health professionals. Infection Prevention Program also focuses on the prevention of infectious risk in the city sector.
**Objectives:** To assess the impact of access to training and professional practice in a group practice on the use of Hydro Alcohol Products (HAP) in the liberal sector.
**Methods:** It is a study by online questionnaire from 01 to 30/04/2016, with the general practitioners and nurses liberal volunteers in a specific region. The analysis consisted of a descriptive study and then identified the factors favoring the use of HAP by logistic regression. The evaluation of the model was tested by the Hosmer-Lemeshow test, the discriminatory capacity by the ROC curve.
**Results:** 677 completed questionnaires. The cohort consisted mainly of nurses (88%) and women (82%). Almost 80% of professionals had graduated for more than 10 years, 61% were in private practice $\geq 3$ people. 84% reported using HPA at home, 63% in the practice, and 5% were aware of HPA indications and contraindications. In the office, only sex is a risk factor for HPA under-utilization ($p = 0.002$): women using HPA 2 times less than men. At home, only the seniority of the diploma is a factor of risk of under-utilization of the HPA ($p = 0.01$): professionals graduates between 5 and 10 years use 3 times more HPAs than graduates > 10 years (no difference between graduates < 5 years and graduates > 10 years).The barriers to the use of HPAs were numerous, however, 61% of professionals would be interested in training.
**Conclusion:** The lesser participation of doctors is due to a less relayed diffusion than to the nurses. A significant difference in practice between practice and home is explained by the use of HPAs instead of hand washing when a water source is not readily available. However, these professionals use HPAs in an insufficient manner and group work does not affect their use. The only contribution of knowledge in training does not make it possible to improve these practices. It would be interesting to work on the representations of the professionals concerning the PHA in collaboration with a psychologist, a communicator or a sociologist.
**Disclosure of Interest**
None Declared

### P106

#### Standardized handwashing education in kindergarten - this single step on the way to better infection control could be significant in Switzerland

Pirmin Pfister
GP Practice, Regensdorf, Switzerland
*Antimicrobial Resistance and Infection Control* 2017, **6(Suppl 3):**P106

**Introduction:** Patients' hand hygiene behavior has an influence on the control of healthcare associated infections. Moreover, healthcare workers learn professional hand hygiene methods, which reduce healthcare associated infections. In both situations the hand hygiene education, which took place in childhood, is their basis.
**Objectives:** To understand how handwashing education is intended to take place in Swiss kindergartens.

**Methods:** Analyse of Swiss kindergarten curricula concerning handwashing education. In addition, 12 Swiss teacher training colleges were asked per e-mail about how they train future teachers in the instruction of handwashing to kindergarten children.
**Results:** The cantonal curricula differ. In some the handwashing is a specific subject (e.g. Canton Zurich). In others handwashing is not mentioned specifically (e.g. Lehrplan 21, plan d'études romand, piano di studio), rather the handwashing subject is thought to be integrated in a more general health competence. Of the 12 teacher training colleges contacted 9 (75%) gave an answer. These colleges do not use standardized teaching aids concerning handwashing.
**Conclusion:** In Switzerland handwashing education at kindergarten is not uniform. Standardized teaching aids, which are of the latest scientific findings, would help the kindergarten teachers to focus on the subject of effective handwashing and to teach it in an optimal way. A good fundament of hand hygiene behavior in childhood may later help patients and healthcare workers to attain better, habitual supported hand hygiene performance.
**Disclosure of Interest**
None Declared

### P107
### Surgical hand preparation in surgical services: results of an audit conducted at the Chu of Beni Messous Algiers in 2017

R. Belkaid, G. Brahimi, A. L. Rebouh, S. Ait Seddik, A. Larinouna, N. Cheboub, H. Khellaf, W. Djouimaa, A. Chetitah, A. EL Kechai, D. Chenini, F. Boudebouz, S. Amrit
CHU de Béni Messous, ALGER, Algeria
**Correspondence:** R. Belkaid
*Antimicrobial Resistance and Infection Control* 2017, **6(Suppl 3):**P107

**Introduction:** Hand hygiene (HH) is key to prevent healthcare-associated infections. Surgical hand preparation (SHP) is part of the validated measures for the prevention of surgical site infections in operating rooms.
**Objectives:** To evaluate the adherence and quality of SHP by washing or alcohol-based hand scrubbing.
**Methods:** A prospective internal audit on SHP practices and quality in child surgical services (CSS), general surgery (GS) and ophthalmology (OPHT) by the observation of the operating room staff. The audit took place from 22/01 to 05/02/2017. The data were entered and analyzed using EPIDATA software.
**Results:** The audit involved 140 SHP opportunities; surgeons represented 42.7%, anesthetists 25.7% and paramedical staff 31.4%. Adherence to HH was 52.1% (CSS 56.1%, GS 54.5%, OPHT 45.4%). 95.9% carried out a SHP by washing and 4.1% by alcohol-based scrubbing. Adherence to SHP was better for surgeons (90% versus 20% for the rest of the operating room staff ; $p < 0.001$). Professional attire was compliant in 76.4% (absence of jewelery 85%, short nails 90%, short sleeves 91.4%), with significant difference for surgeons (surgeons 90%, anesthetists 55.6%, paramedicals 75%, $p < 0.001$). For the SHP steps, the first wash was performed in 53.7% for CSS, 52.7% in GS and 38.6% OPHT. The misuse of the Betadine brush was observed in 39.3% (nail, hand and forearm brushing), surgeons in 72.7% of cases, anesthetists in 18.2% and para-medical staff in 9%. The average duration of the SHP was 1.40 minutes ± 1.02, the overall time was met in only 2.73%. Tamponing drying was not respected.
**Conclusion:** Adherence to SHP remains low among anesthesiologists and paramedics. The SHP steps weren't met by the majority of the staff. The SHP by alcohol-based handrub scrubbing remains to be promoted.
**Disclosure of Interest**
None Declared

### P108
### Compliance of surgical hand prepartion before and after training of obstetric-gynecologists, Algiers 2014-2015

G. Brahimi, S. AMRIT, S. AIT SEDDIK, H OULD BABA ALI, A. L. REBOUH, Y. TAYEBI, R. BELKAID
CHU de Béni Messous, ALGER, Algeria
**Correspondence:** G. Brahimi
*Antimicrobial Resistance and Infection Control* 2017, **6(Suppl 3):**P108

**Introduction:** Healthcare workers hands are described as the most frequent source of microorgamisms cross-contamination during patient care. Surgical hand preparation (SHP) is an unavoidable step that no one questions in the prevention of the risk of surgical site infection (SSI).
**Objectives:** To evaluate the impact of hand hygiene training (HH) and the introduction of alcohol-based handrub (ABHR) solutions on SHP compliance.
**Methods:** It is a before and after training on HH audit. It concerned the resources, the practices of the SHD by washing or ABHR friction realized among the operating room staff. A clear definition of non-compliance was established and used by observers to achieve high inter-observer reliability. The first audit took place in November 2014 and the second in June 2015. Data were entered and analyzed using EPI INFO software.
**Results:** A total of 111 individuals were audited, 52 before training and 59 after.The adherence rate increased by 16.3 points from 76.9% to 93.2% after training (p
**Conclusion:** By focusing on material resources, complemented by training and awareness of caregivers, we improved the compliance rate of SHP.
**Disclosure of Interest**
None Declared

### P109
### The 250-year-old history of hand hygiene in health care

Rita Szabó[1], Andrea Kurcz[1], Arnold Bosman[2]
[1]Department of Hospital Epidemiology and Hygiene, National Center for Epidemiology, Budapest, Hungary; [2]Transmissible, Houten, -
**Correspondence:** Rita Szabó
*Antimicrobial Resistance and Infection Control* 2017, **6(Suppl 3):**P109

**Introduction:** Healthcare-associated infections affect millions of patients each year, generating a major threat to patient safety all over the world. Many investigations conducted over the past centuries have confirmed the important role of contaminated hands of healthcare workers' in the transmission of pathogens.
**Objectives:** This review is aimed to serve the long-lasting tribute of these great pioneers in the history of medicine, although their theories were often dismissed by doctors, midwives, nurses, and even the public.
**Methods:** Historical review.
**Results:** The concept of hand hygiene as a method for infection control dates back to Alexander Gordon in the 18$^{th}$ century. He documented the first systematic observation regarding hand hygiene more than 200 years ago in Aberdeen, Scotland. He recognized the contagiousness of the puerperal fever and the role of caretakers' hands in cross-transmission leading to outbreaks and high fatality. Over the centuries, several pioneers suggested the hand hygiene as a preventive measure, with various degrees of professional recognition. Work of Ignaz Phillip Semmelweis, Hungarian obstetrician who demonstrated experimentally that appropriate hand hygiene significantly reduced risk of puerperal infections and maternal deaths, was rejected by peers. The germ-theory was established only 20 years later, through discoveries of Pasteur, Koch, and Lister. Even in 21$^{st}$

century, compliance to hand hygiene remains low. Current work by Didier Pittet highlights the importance of a multimodal approach.
**Conclusion:** Strategies to improve hand hygiene compliance must include system change, staff education and motivation, the use of performance indicators, and hospital management support to save our patients.
**Disclosure of Interest**
None Declared

### P109b
### State action plan for the implementation of the multimodal hygiene promotion strategy in Jalisco, Mexico
Hilda Marquez
Health Quality Division, The Jalisco Health Secretariat, Mexico, Mexico
*Antimicrobial Resistance and Infection Control* 2017, **6(Suppl 3):**P109b

**Introduction:** Reducing patient harm is the main objective of quality in health services in Jalisco. Considering patient safety as a priority, the Jalisco State Quality Strategy coordinated the implementation of the WHO multimodal hand hygiene (HH) improvement strategy, including the monitoring of the WHO Hand Hygiene Self-Assessment Framework (HHSAF).
**Methods:** The HHSAF was applied by the quality manager and the epidemiologist of each hospital. According to the HHSAF, the level of HH of each hospital was classified as "inappropriate" when the score ranged from 0 to 125; "basic", from 126 to 250; "intermediate", from 251 to 275; or "advanced", from 376-500.
**Results:** In total, there were 36 public hospitals from the State Health sector, 32 from the Ministry of Health (SSJ), 3 from Municipal Medical Services (SMM) and 1 from the Institute of Social Security and Social Services of State Workers (ISSSTE). Results identified 1 hospital in an inadequate level, representing 3%; 13 hospitals in a basic level (36%); 15 hospitals at intermediate level (42%); and 7 hospitals at the advanced level (19%). Specific improvement areas have been identified according to each component, to favor the impact of actions to promote HH in health services. The need to improve the availability of alcohol-based hand sanitizers was identified, as well as needs to invest in infrastructure at patient care points and in critical areas, Hospitals have received a specific budget for the continuous acquisition of HH products and training. Likewise, the commitment of the medical, nursing and administrative managers to promote HH activities was identified, as well as the need to strengthen the continuous training of all personnel, and all new staff.
**Conclusion:** The results showed that it is necessary to strengthen dissemination, promotion and continuous improvement actions for HH in Jalisco, as well as to implement or strengthen inter-institutional exchanges for innovation related to HH, recognition systems for staff, and to establish institutional goals related to HH. This action is the first part of the implementation of the multimodal HH strategy kn Jalisco, with the objective to progressively improve HH statewide and reduce healthcare-accociated infections in each hospital.
**Disclosure of Interest**
None Declared

### P109c
### Catheter-related bloodstream infection in critical care units of a Costa Rican third level pediatric hospital: incidence and risk factors
Tatiana Barrantes Solís, Milena Arias Jiménez, Marlen Villegas Sánchez, Jorge Delgado Arguedas, Marcela Hernández de Mezerville
Hospital Nacional de Niños, Dr. Carlos Sáenz Herrera (HNN), San Jose, Costa Rica
**Correspondence:** Tatiana Barrantes Solís
*Antimicrobial Resistance and Infection Control* 2017, **6(Suppl 3):**P109c

**Introduction:** Catheter-related bloodstream infection (CRBSI) is one of the main complications among critical care patients, associate high morbidity and mortality in children. We use CDC case definition to identify and confirm bloodstream infections.
**Objectives:** To describe the rate and risk factors associated with central line infections in the intensive care units of the "Hospital Nacional de Niños, Dr. Carlos Sáenz Herrera" (HNN), the only pediatric referral center of Costa Rica.
**Methods:** Descriptive study of all patients under 13 years old, hospitalized at the Neonatal Intensive Care Unit and the Pediatric Intensive Care Unit at HNN, who had a central line and developed a bloodstream infection between January 1$^{st}$, 2013 and December 31$^{st}$, 2015.
**Results:** We identified 161 CRBSIs, for a global rate of 4.2 for 1000/catheter-days. Average age was 2.8 months and there was no difference among gender. The most common previous illness was non-cardiac congenital malformation. The median duration of hospitalization was 112 days. Only 12 children had more than one CRBSI episode during hospital stay. The median time between central line insertion and positive blood culture was 20.3 days. Main risk factors were guidewire exchange (OR 6,7; CI95% 0.8-55), congenital heart disease (OR 3.7; CI95% 1.1-12.6) and other congenital malformations (OR 3.5; CI95% 1.3-9.3).
**Conclusion:** The overall rate of CRBSI at the Hospital Nacional de Niños is similar to those reported from other third level pediatric hospitals in developing countries. The main affected children were infants with previous illnesses and longer hospital stay. Time elapsed between central line insertion and bloodstream infection suggests manipulations of the lines as possible source of catheter colonization; we also recommend the practice of guidewire exchange to be discouraged.
**Disclosure of Interest**
None Declared

## Session title: Behavioural science

### P110
### Multidrug resistant organism surveillance project: assessing patients and healthcare providers attitudes; knowledge and beliefs regarding multidrug resistant organism infection, surveillance and infections control practices at Chu Kigali
Olivier M. Manzi
University of Rwanda/ CHUK, Kigali, Rwanda
*Antimicrobial Resistance and Infection Control* 2017, **6(Suppl 3):**P110

**Introduction:** Nosocomial infections caused by multi-drug resistant organisms (MDRO) contribute significantly to mortality and prolonged length of stay among hospitalized patients, A significant proportion of MDRO infections are transmitted between patients via healthcare workers hands, methicillin resistant staph aureus (MRSA) and extended-spectrum beta-lactamase producing (ESBL) organisms are recognized as principal pathogens that are easily transmitted in healthcare settings.
**Objectives:** This study aims to assess patients and healthcare providers attitudes, knowledge or beliefs around multi-drug resistant surveillance and infection control practices at CHU Kigali.
**Methods:** Questionnaires were administered to both healthcare providers as well as patients hospitalized on various department of the hospital. Healthcare providers self-administered questionnaires while patient respondents were guided on how to fill them by study investigators. Afterwards, data was collated for final analysis. Simple descriptive statistics were used to report study findings.
**Results:** Overall, 250 healthcare providers and 245 patients completed the survey. The study found that while 128/250 (51,2%) of HCWs had limited knowledge about MDROs, there was a significantly lower knowledge about them among surveyed patients ($P < 0.05$). While 236 HCWs (94.4%) agree with the of soap and water as being appropriate for hand hygiene, only 174 (69.6%) HCWs reported that they routinely wash their hands after handling patients. A majority of patients (64,9%) were not satisfied with HCWs hand hygiene practices. Regarding surveillance procedures, 71 patients (29%) reported discomfort with accepting rectal swab for screening of colonization with ESBL producing organisms and 74% of HCWS still believe that beta-lactam antibiotics can be used in MDRO infections.
**Conclusion:** This study showed the low level of knowledge by patients and limited knowledge by healthcare providers regarding nosocomial infections caused by MRSA and ESBL producing organisms, these knowledge gaps about MDRO and their surveillance is concerning and should inform future efforts to educate patients and their providers about this public health problem.

**Disclosure of Interest**

None Declared

### P111

#### An observational study on the knowledge and adherence of personal protective equipment removal guidelines in intensive care unit nurses

Hyun Ju Chun[1, 2], Jae Sim Jeong[3], Hye-Ran Choi[1], Ihn Sook Jeong[4]
[1]Department of Clinical Nursing, University of Ulsan, Ulsan; [2]Department of Nursing, Asan Medical Center, Seoul; [3]Department of Clinical Nursing, College of Medicine, University of Ulsan, Ulsan; [4]College of Nursing, Pusan National Univeristy, Pusan, Korea, Republic Of
**Correspondence:** Jae Sim Jeong
*Antimicrobial Resistance and Infection Control* 2017, **6(Suppl 3):**P111

**Introduction:** Proper and precise removal, as well as wearing personal protective equipment (PPE) after contact with an infectious disease is essential to prevent infection transmission. However, there are no studies reported in Korea and the medical environment is different from that of foreign countries.

**Objectives:** This study is to investigate the nurses' knowledge and adherence of PPE removal guidelines for patients on the contact precautions and to explore ways to improve the adherence of the PPE removal guidelines by identifying the factors that affect them.

**Methods:** The 43 nurses working in two medical intensive care units and one surgical intensive care unit of Asan Medical Center (Seoul, Korea) were observed at least twice about the adherence of PPE removal guidelines in October 2016. The general characteristics and knowledge on the adherence of the PPE removal guidelines were identified with self-reported questionnaires.

**Results:** The general characteristics of 43 subjects were 86.0% for female and 86.0% for 4-year bachelor graduates. 97.7% were staff nurses and the total hospital career was 3.56 ± 3.71 years. The average daily need for PPE was 21.44 ± 14.11 per patient. The knowledge of PPE removal guidelines was 82.36 ± 10.32 points on a scale of 100 points. The observed adherence of the PPE removal guideline was 83.54 ± 9.95 points on a scale of 100 points. But 9.8% of the nurses correctly performed the entire removal instructions. The knowledge of the PPE removal guidelines was significantly higher in nurses with above the master's degree ($p = .001$) and nurses who was confident on the accuracy of the removal guideline ($p < .001$). There was a difference in the observed adherence of the PPE removal guidelines depending on the type of gloves ($p = .001$). The reason of not correctly removing PPE was that they 'did not know the proper method' (48.1%).

**Conclusion:** Knowledge and adherence scores for PPE removal guidelines were high for each item, but few nurses accurately performed the entire removal instructions, and confidence in correct removal instructions was low. The factors that influence the adherence on the PPE removal guidelines are not clearly identified and need further clarification.

**Disclosure of Interest**

None Declared

### P112

#### Improvement of behaviors and practices for infection control in West Africa: observation of early trends on the success factors

Babacar Ndoye
ICAN, Dakar, Senegal
*Antimicrobial Resistance and Infection Control* 2017, **6(Suppl 3):**P112

**Introduction:** The gradual change in the behaviors and practices of health workers in infection prevention and control (IPC) is currently one of the main challenges faced by the first structured programs being implemented in the African region. The author presents his experience, based on the results of some projects to improve hand hygiene and health care waste management in some West African hospitals.

**Objectives:** The aim is to identify some key success factors for easier implementation of the numerous current projects on IPC in the region, with a situation of huge and diversified gaps.

**Methods:** Observational study on the results of improvement of performances obtained on two projects, carried out in three different countries:

- A project to improve hand hygiene performance in 21 Senegalese hospitals based on the multimodal strategy and the tools proposed by the WHO (using the hand hygiene self-assessment framework tool, and then the five components of the strategy)
- A project to improve the healthcare waste management performance in 10 Sierra Leonean hospitals and 8 Liberian hospitals, based on a similar strategy: situational analysis (using a simplified rapid assessment tool), training and raising awareness of technical staffs and administrators, and providing equipment (autoclave for Treatment of infectious waste), followed by implementation and monitoring and evaluation with a supervisory grid to quantify the evolution of performances.

**Results:** - Senegal: the National Armed Forces Training Hospital recorded the best performances

- Sierra Leone: the two hospitals managed by the Italian and Dutch cooperation performed best and were the only ones to regularly use the autoclaves supplied to treat infectious waste
- Liberia: the hospital managed with the Chinese cooperation support, achieved the second best overall performance and was the only hospital to have regularly used the autoclave provided for the treatment of infectious waste

**Conclusion:** Hospitals not administered directly by health ministries but, either by another national institution such as the military or through international cooperation support, have consistently recorded the greatest progress in changing behavior and practices. Resources and leadership will undoubtedly be determining success factors for the implementation of ICP projects in Africa.

**Disclosure of Interest**

None Declared

### P113

#### Qualitative analysis of perceptions, attitude and behaviors of healthcare workers and researchers regarding an automated hand hygiene monitoring technology

Clément Tarantini
Social Sciences, Aix-Marseille Université-Inserm, MARSEILLE, France
*Antimicrobial Resistance and Infection Control* 2017, **6(Suppl 3):**P113

**Introduction:** The infectious disease unit of North Hospital, Marseille, has set up automated monitoring of the hand hygiene of healthcare workers (HCWs) (1) in order to provide a better understanding of their behaviors.

**Objectives:** To assess HCWs perceptions and attitudes toward research and development of technological devices to improve hand hygiene practices in hospital ward.

**Methods:** In-depth interviews and ethnographic fieldwork.

**Results:** To the HCWs these technologies can introduce health risks and social risks due respectively to electromagnetic waves and principle of traceability on which these devices are based (2). They expressed that these devices strongly decontextualizes their practices from the realities of their daily work. HCWs also criticize scientific researches. They felt tensions and contradictions with the goals, the priorities and the values of team researchers (3). To conclude this study showed that the implementation of scientific researches in this ward emphasizes the hierarchical order and the power struggles within the department and disrupt the negotiated order in the ward (4).

**Conclusion:** Innovative technologies in the field of scientific research should be adapted to the routine practices of HCWs in the complex hospital environment and specific care constraint. Researchers have to take into account the social and the professional issues of HCWs.

(1) Boudjema S et al.. MediHandTrace (R): a tool for measuring and understanding hand hygiene adherence. Clin Microbiol Infect 2014 Jan;20(1):22-8
(2) Fisher, J & Monahan, T. Tracking the social dimensions of RFID systems in hospitals. Intern J of Med Inf 2008, (77): 176-183.
(3) Sutton E et al. Ethnographic process evaluation of a quality improvement project to improve transitions of care for older people. BMJ Open 2016; 6:e010988.
(4) Strauss, A et al. Social organization of medical work. 1985, Chicago and London: The University Chicago Press.

Disclosure of Interest
None Declared

### P114

#### The behaviour of health care workers on infection prevention and control practices and its impact on nosomial infections

Angeline Chiotcha
Quality Management, MINISTRY OF HEALTH, LILONGWE 3, Malawi
*Antimicrobial Resistance and Infection Control* 2017, **6(Suppl 3):**P114

**Introduction:** One of the fundamental principles of patient safety in hospital settings is the reduction of cases of nosocomial infections. Infections acquired during hospitalization have been reported in literature, what has not been given attention is how social and psychosocial behaviour of health workers contribute to these infections

**Objectives:**

· To determine how social bonding can lead to negative behaviour and its consequence on IPC practices

- To understand if effective infection prevention and control practices is behaviour-driven rather than mere lack of knowledge and awareness

**Methods:** Descriptive qualitative research design was deployed to uncover the health care workers behaviour in infection control and prevention phenomena using an in-depth interview guide. A purposive sample of 12 Health Workers, with different levels of experience from Operating Theatres was interviewed. Interviews were recorded and transcribed verbatim. Thematic analysis was done to identify dominant themes that described behaviours in infection control practices.

**Results:** Respondents stated that they changed to inappropriate practices in order to see themselves as belonging to a group and they would bring behaviour to align with the group norms. Inconsistencies in behaviour and attitudes were identified where some health workers influence others within their space to behave in a particular way. Even if some of the respondents have positive attitude towards Infection Control by doing what is right, their behaviour will still be weakened when others in the practice setting do not conform to standards of hygiene.

It was also discovered that individual behaviour depends on the environment, punishment and rewards associated with engaging or not engaging in the desired IPC guidelines.

**Conclusion:** Results suggest how psychosocial factors influence HCWs behaviour thus compounding concerns about the risk of HCAI. Addressing psychosocial and motivation is an important step in effective infection prevention and prevention of HCAI.

Although the study took place in a small developing country, it is possible that the issues raised cuts across boundaries.

**Disclosure of Interest**

None Declared

### P115

#### Knowledge, attitude & practices vis-a-vis needle stick injuries amongst nurses: a pilot study in a tertiary care hospital in India

Vipin Koushal[1], NAVIN PANDEY[1], Pranay Mahajan[1], Yashpal Sharma[2]
[1]Hospital Administration, Post Graduate Institute of Medical Education & Research, Chandigarh; [2]Deen Bandhu Chotu Ram Institute, Karnal, India
**Correspondence:** Vipin Koushal
*Antimicrobial Resistance and Infection Control* 2017, **6(Suppl 3):**P115

**Introduction:** Needle stick injuries can be a major source for blood-borne diseases among the health care workers.

According to Centre for disease control, 600,000 to one million NSIs occur each year. More than 50% of these NSIs go unreported.

Lack of SOPs by healthcare organizations further compound this problem.

**Objectives:** To assess incidence, awareness and perception regarding needle stick injuries amongst nurses of PGIMER.

**Methods:** Cross sectional KAP study on a sample of 164 Nurses working at PGIMER

Using Semi structured, self administered questionnaire.

**Results:** 65% of nurses had correct knowledge of diseases spread by NSIs.

80% nurses were aware of procedures to be followed post NSI.

34.1% nurses practiced re-capping of needles. Only 50.6% nurses admitted to wearing gloves.

42.07% of nurses encountered NSI during their career.

84.05% nurses of those suffering NSIs enquired about the patient's disease history.

39.1% nurses had NSIs during administrating injection, 8.6% nurses during manipulation of I.V. line, 20.2% nurses during recapping, 26% nurses during discarding needle, & 10.1% nurses during clean-up.

11.5% nurses suffered NSI in the palm, 92.7% nurses in fingers & 2.8% nurses in the arm. 58.18% nurses did not report Needle Stick Injuries.

14.4% of nurses were unaware of reporting procedure post NSI.

4.3% of nurses felt they might get into administrative trouble on reporting a Needle Stick Injury. 4.3% nurses felt it was not important to report a NSI.10.1% of nurses did not report considering the prick as minor.

Only 10% of those suffering NSI had taken Post Exposure Prophylaxis.

Only 7% of nurses suffering NSI completed the recommended PEP course.

59.4% nurses felt that they did not get adequate psychological support from the hospital.

**Conclusion:** Incidence of NSIs in this study was 42%.

Only 72% nurses washed their hands with water & soap after NSI.

PEP was not taken by 88.2% nurses.

All this indicate a lack of adequate awareness which can be achieved by rigorous Continuous Medical Education.

Hospital management has to devote resources to address this problem.

Training, training and training is the mantra.

**Disclosure of Interest**

None Declared

### P116

#### Knowledge, attitudes and practices of the hospital staff towards the respect of the standard precautions of hygiene

Héla Ghali, Nabiha Bouafia, Asma Ammar, Sabrine Ben Abdallah, Mohamed Mahjoub, Asma Ben Cheikh, Olfa Ezzi, Radhia Helali, Wadia Bannour, Mansour Njah
Hospital Hygiene Department, Sousse, Tunisia
**Correspondence:** Mohamed Mahjoub
*Antimicrobial Resistance and Infection Control* 2017, **6(Suppl 3):**P116

**Introduction:** Patient safety is receiving attention worldwide growing and is now being considered as a human rights issue. One important indicator of patient safety is the rate of nosocomial infections (NI) among hospital patients. However the prevention of NI needs a better knowledge of the risk factors, such as the application of standard precautions defined by the "Centers for Disease Control" (CDC).

**Objectives:** Our study aimed to evaluate the knowledge, attitudes and practices of nurses towards the respect for Standard Precautions of Hygiene (SPH) in a Tunisian teaching hospital.

**Methods:** It was a cross-sectional study conducted during two months (February and March 2016), in a hospital of the center-east of Tunisia, among nurses working in 13 services deemed to have a high risk of NI because of the workload and profile of patients hospitalized in these units. We used for the collection of data, a self-administered questionnaire inspired of the Protocol of "The audit Standard Precautions" adopted by the Coordination Centers and the fight against the Nosocomial Infections of France. The statistical analysis was performed using the SPSS software 18.

**Results:** The response rate was 88.6%. 61.3% said that they have already received training on SPH. 32.3% of nurses thought that they are well aware of the components of the SPH. The majority of respondents knew that these precautions are intended to protect patients and caregivers. 78.1% of the paramedical personnel responded that the hygiene of the hands is the most effective measure to limit the transmission of infectious agent in a care institution and 93.5% felt that it is necessary to disinfect hands before and after contact with a patient. However, less than half believed that the hygiene of hands must be respected between two cares given for the same patient. 68.4% made a flush of physiological serum in case of projection of blood or biological fluid on the mucous membranes. 69% of respondents washed their hands or used an antiseptic in case of an accident of exposure to blood.

**Conclusion:** Our study has shown that there are many shortcomings which require the implementation of certain measures such as awareness and training sessions.

**Disclosure of Interest**

None Declared

### P117

#### Difficulties in using personal protective equipment: training experiences with the 2015 outbreak of Middle East respiratory syndrome in Korea

Jahyun Kang[1], Eun Jin Kim[2], Jeong Hwa Choi[3], Hae Kyung Hong[4], Si-Hyeon Han[5], In Soon Choi[6], Jae Geum Ryu[1], Jinwha Kim[7], Jae Yeun Kim[8], Eun Suk Park[9]

[1]Seoul National University, College of Nursing; [2]Seoul National University Hospital; [3]Konkuk University Medical Center, Seoul; [4]Soonchunhyang University Bucheon Hospital, Bucheon-si; [5]Dankook University Hospital, Cheonan-si; [6]Chung-Ang University Hospital; [7]Soonchunhyang University Seoul Hospital, Seoul; [8]Konyang University Hospital, Daejeon-si; [9]Yonsei University Severance Hospital, Seoul, Korea, Republic Of

**Correspondence:** Jahyun Kang

*Antimicrobial Resistance and Infection Control* 2017, **6(Suppl 3):**P117

**Introduction:** The inter- and intra-hospital outbreak of Middle East respiratory syndrome (MERS) in South Korea resulted in 38 deaths, 186 confirmed cases, and 16,693 quarantines from May to December 2015. As 39 healthcare personnel (HCP) were infected, HCP can be at risk of contracting infectious diseases.

**Objectives:** For HCP safety through the correct use of personal protective equipment (PPE), this study aims to evaluate practical barriers to PPE use during- and post-MERS outbreak.

**Methods:** With Institutional Review Board approval, a focus group interview with infection control nurse leaders was conducted with consent and a brief survey on demographics and hospital characteristics. Participants' narrative responses and interactive discussions were recorded and categorized by theme. A fishbone diagram on summarized outcomes was reviewed by all participants and 2 board members of the Korean Association of Infection Control Nurses.

**Results:** From Seoul and Chungcheongnam-do, 7 leaders participated. Their hospitals (1,122 average beds [706-1980]) had at least 3 probable MERS cases (range: 3-5,300; 0-82 confirmed) during the 2015 outbreak. Leaders reported PPE training for 50-4,095 HCP during- and post-MERS using PPE sets and fluorescent markers (6 of the 7). All reported difficulties in developing hospital-customized PPE protocols and trainings despite variations in PPE and inconsistent donning/doffing ordering in guidelines. Difficulties observed in PPE use were anxiety, unfamiliarity, confusion from unstandardized protocols, doubts about PPE quality and effectiveness, complexity in PPE combinations, and a lack of personnel and budget.

**Conclusion:** Evidence-based gold-standard PPE guidelines should be formulated to reduce the burdens at each hospital. Thereafter, repeated training with standardized guidelines would increase HCP competencies in PPE use.

**Disclosure of Interest**

J. Kang Grant/Research support from: The Seoul National University Invitation Program for Distinguished Scholars, E. J. Kim: None Declared, J. H. Choi: None Declared, H. K. Hong: None Declared, S.-H. Han: None Declared, I. S. Choi: None Declared, J. G. Ryu: None Declared, J. Kim: None Declared, J. Y. Kim: None Declared, E. S. Park: None Declared

### P118

#### Mitigating the scourge of hospital acquired infection: can nursing education help?

Sunil Kakkar[1,2], Vipin Koushal[2], Manisha Biswal[3], Pankaj Arora[2], Sandhya Ghai[4], Navin Pandey[2], Anil Gupta[2]

[1]YS Parmar Government Medical College, Nahan; [2]HOSPITAL ADMINISTRATION; [3]Microbiology; [4]Nursing, Post Graduate Institute of Medical Education & Research, Chandigarh, India

**Correspondence:** Sunil Kakkar

*Antimicrobial Resistance and Infection Control* 2017, **6(Suppl 3):**P118

**Introduction:** Failure to comply with infection control practices by hospital staff has been observed as a major cause for increased incidence of HAI. Huge emphasis is currently being laid on educating & training health care workers, especially nurses, to reduce HAIs. The moot question that arises is how effective are these programs in meeting their intended objectives?

**Objectives:** This study was conducted on nurses at PGIMER to evaluate efficacy of a training module on infection control practices in immediate post intervention phase as also over a sustained period.

**Methods:** This was a questionnaire based interventional study conducted at PGIMER. An educational module was used to train nurses vis-à-vis infection control practices. The nurses were evaluated before training and subsequently 1 week and 1 month after training.

**Results:** The knowledge score of nurses on evaluation 1 week after training was significantly higher than their pre training scores ($p = 0.00$). Similarly the knowledge scores of nurses 1 month after training were also significantly higher than their pre training scores ($p = 0.00$). There was a significant decrease in knowledge scores on evaluation of nurses after a month as compared to their scores on evaluation after a week of training ($p = .001$). Unlike knowledge scores, the attitude of nurses towards HAI did not show a significant decrease on evaluation 1 month after training vis-a-vis their evaluation 1 week after training ($p = 0.026$). Evaluation of practices of nurses vis-à-vis hospital acquired infection did not show any significant difference in pre and post training phases.

**Conclusion:** Results showed a significant decline in knowledge level of nurses 1 month after training. This clearly demonstrates a need of continous training programs for knowledge improvement of this vital staff in a hospital.

**Disclosure of Interest**

None Declared

### P119

#### Are we using suitable behavioural interventions for healthcare workers' recidivist hand hygiene behaviour? A critique of management theories

Yew Fong Lee[1,2], Didier Pittet[1], Walter Zingg[1], Suraya A. Husin [2], Hock Hin Chua [3], Mary W. Ago [3], Loke Meng Ong [2], See Yin Wong[3], MaryLouise McLaws[4]
[1]Institute of Global Health and Infection Control Program, University of Geneva Hospitals and Faculty of Medicine, Geneva, Switzerland; [2]Ministry of Health, Kuala Lumpur; [3]Sarawak General Hospital, Kuching, Malaysia; [4]University of New South Wales, Sydney, Australia
**Correspondence:** Yew Fong Lee
*Antimicrobial Resistance and Infection Control* 2017, **6(Suppl 3):**P119

**Introduction:** Enhanced hand hygiene (HH) compliance bundled with other infection prevention practices reduce healthcare associated infection (HAI). Compliance can be as low as 30% but improvement can be achieved with multimodal strategies including role models, feedback, peer reminders and traditional poster messages. Sustaining improvements is difficult where staff interact with more than one patient and have limited direct contact with the supervising manager. The choice of a behaviour change theory suitable for this environment requires an understanding of the behaviour, benefits and disadvantages of applying each at patient level.
**Objectives:** To examined current theories used to improve HH compliance.
**Results:** An early behavioural model identified that HH is practiced as a mix of self protection and ultruism have achieved mix responses. The Geneva HH multimodal promotion model applied in Australia without a leadership component failed to improve compliance ($P = .204$) but when leadership was included compliance improved dramatically by 56% ($P < .001$). The peer-centred approach of the Washington protocol applied in Australia resulted in 48% (RR 1.48; CI = 1.2 to 1.81) improvement. But neither of these protocols sustained compliance. Even daily feedback of compliance from continuous automated HH compliance surveillance did not budge the 30% compliance on the ward with poor social cohesion compared with the socially cohesive ward where HH increased from 49% to 66% and reached 80% on occasions.
**Conclusion:** Sustaining improvements in HH on the ward requires a new approach because (i)it is an activity that has been learnt in childhood as a self-protective action resulting in lower compliance for Moment 1 compared to Moments 3-5 (ii)poor HH do not immediately result in infection unlike medication errors. We will discuss the benefits and disadvantages of applying of the Theory of Innovation where HH of a well-liked leader is mirrored, Social Cognitive Theory where peer-identified role models are identified to lead or Social Network analysis staff with highly connected information links could assist behaviour change.
**Disclosure of Interest**
None Declared

### P120

#### What do they know and what do they do? A cross-sectional mixed-methods study of nursing perspectives on individual and organizational influences on infection prevention in nursing homes

Judith Hammerschmidt, Tanja Manser
University Hospital Bonn, Institute for Patient Safety, Bonn, Germany
**Correspondence:** Judith Hammerschmidt
*Antimicrobial Resistance and Infection Control* 2017, **6(Suppl 3):**P120

**Introduction:** Infections are a major cause of morbidity and mortality in nursing homes (nh). Effective infection prevention (IP) requires expertise, continuous training, resources and local efforts as the challenges to patient safety are often organizational rather than clinical.
**Objectives:** We aim to contribute an understanding of individual and organizational factors of IP by exploring hindering and facilitating factors from the perspectives of staff and nursing managers.
**Methods:** We employed a mixed methods concurrent triangulation strategy. We surveyed nh staff about their knowledge, behavior and compliance concerning IP and interviewed nursing managers to explore their perspectives on organizational influences on IP.
**Results: Quantitative strand:** The majority of all survey participants (N =165) was female (80%). Most were licensed nurses (52%) working day shifts (63%). 60% responded that IP standards were complete and clear to them. 41% of respondents reported to always apply the contents of their last IP training. 21% report wearing hand and arm jewelry always or often during patient care. Nurses reported that gloves were always available (79%) in the residential area and protective clothing was always (67%) accessible. However, 15% indicated that they cannot disinfect their hands during active care, because no hand rub is available in resident rooms. Most nurses perceive their direct supervisor as a role model for IP compliance (always = 38%). **Qualitative strand:** Most interview participants (55% unit managers) were female (89%), and 37% had worked in their institution for 11-15 years. While they reported that nurses always have access to IP standards and highlighted the importance of staff empowerment concerning IP they indicated challenges educating foreign-language staff and felt that staff was not proactively seeking IP information. We found inconsistencies in role modeling behavior of nursing managers and tensions between local and institution-wide IP leadership.
**Conclusion:** Integrating perspectives of nursing staff and managers our study allows for a deeper understanding of the perceived barriers and facilitators of IP in nh. It also highlights the challenges of consistent role-modeling and that nursing managers do not fully reflect on being a role model for IP.
**Disclosure of Interest**
None Declared

### P121

#### Guinness World Record Attempt – analysing our experiences

Lucyna Gozdzielewska, Lesley Price, Paul Flowers, Jacqui Reilly, on behalf of Safeguarding Health through Infection Prevention (SHIP) Research Group
School of Health and Life Sciences, Glasgow Caledonian University, Glasgow, United Kingdom
**Correspondence:** Jacqui Reilly
*Antimicrobial Resistance and Infection Control* 2017, **6(Suppl 3):**P121

**Introduction:** Our recent study showed that although the World Health Organization's (WHO) 6-step technique was significantly more effective than the 3-step technique in reducing hand bacterial load, only 65% of nurses and doctors performed the 6-steps correctly. Thus, we began a campaign with our nursing students to implement our findings, promote the 6-steps and enhance optimal uptake. Following the campaign we involved 417 students in an attempt to break the Guinness World Record (GWR) for a hand-sanitising relay. We successfully broke the GWR with all but 2 students (99.5%) performing this technique correctly.
**Objectives:** To analyse our GWR experience through a lens of selected theories and identified factors that could have contributed to our success.
**Methods:** Greyson's Personality-profiling and Hofstede's Cultural Dimensions theories were chosen, to acknowledge the importance of the complexity of individual, social and organisational factors which shape hand hygiene behaviours as emphasised in WHO Guidelines on Hand Hygiene in Health Care.
**Results:** Student societies are generally characterised by a desire for entertainment and social life. Such societies reflects Hofstede's constructs of indulgence, small power dissonance and cultural dimensions and are likely to achieve better learning outcomes from activities involving amusement and learner-cantered approaches. The campaign used such learner-cantered approaches. In preparation for the GWR attempt, following classroom based activities; students were provided with a series of prompts that encouraged them to practice the 6-step technique daily in a self-directed manner. The GWR attempt involved amusement and social interactions which were likely to contribute to our success. In addition, Greyson argued that nurses achieve better outcomes as a group and are more likely to comply with the interventions that involve emotions and relationships. Thus, having a collective goal of breaking the GWR and the excitement and anticipation of being a GWR holder was likely to further influence participants' performance and learning outcomes.

**Conclusion:** We learned that factors influencing the uptake of hand hygiene education are complex, but analysing experiences through a lens of relevant theories can aid deeper understanding of success determinants.

**Disclosure of Interest**

None Declared

### P122

#### The impact of surveillance cameras in improving infection control practices and outcomes in an adult medical-surgical ICU in a Lebanese tertiary care center

Nada K. Zahreddine[1], Joseph Tannous [2], Tala Kardas [1], Rihab Ahmadieh [1], Zeina Kanafani[3], Souha Kanj Sharara[3]

[1]Infection Control and Prevention Program; [2]American University of Beirut Medical Center, Beirut, Lebanon; [3]Department of Internal Medicine, American University of Beirut Medical Center, Beirut, Lebanon

**Correspondence:** Nada K. Zahreddine

*Antimicrobial Resistance and Infection Control* 2017, **6(Suppl 3):**P122

**Introduction:** Lack of compliance with infection control (IC) measures in intensive care units (ICU) is significantly associated with serious infections and colonization with multidrug resistant Acinetobacter baumannii (MDR-ACB) in critically ill patients.

**Objectives:** To describe changes in the behavior of ICU healthcare workers through surveillance cameras using the Hawthorne effect. Improved compliance with IC practices is noted when healthcare workers (HCW) know that they are being observed or supervised.

**Methods:** Four Surveillance cameras were installed in the adult ICU of the American University of Beirut Medical Center (AUBMC) covering its 10 beds in October 2015. Practices are monitored by the IC team on daily basis and breaches were promptly corrected by calling-back ICU staff. Hand hygiene compliance was monitored through anonymous auditors before and after the installation of the cameras. MDR-ACB Colonization pressure (CP) defined as the proportion of patients colonized/infected with MDR-ACB was used to measure the burden of MDR-ACB and can estimate the probability of its transmission in ICU. CP is calculated by dividing the number of MDR-ACB patient-days by the total number of patient-days and multiplied by 1000.

**Results:** Hand Hygiene compliance rates increased from 56% and 67% before and after patient contact to 86% and 90% respectively. This was also associated with a decrease in the CP from 340.0 to 221.0 per 1000 patient-days in 2015 and 2016 respectively. Device associated infection (DAI) rates in ICU varied depending on the severity of illness, CP and staff compliance with IC practices.

**Conclusion:** Proper hand hygiene practices and decreased CP, both will ultimately lead to a decline in rates of infections and colonizations in ICU. Enhanced IC measures can be sustained using surveillance cameras combined with a comprehensive plan to protect the safety of critically ill patients. Further analysis of device associated infections is needed over prolonged period to measure the effect of surveillance cameras in ICU.

**Disclosure of Interest**

None Declared

### P123

#### Implementing a national infection control policy: clinician perspectives on contextual factors that influence implementation

Sally Havers[1], Philip L. Russo[2], Katie Page[1], Andrew Wilson[3], Lisa Hall[1]

[1]QUT, Brisbane; [2]Deakin Univeristy, Melbourne; [3]Menzies Centre for Health Policy, University of Sydney, Sydney, Australia

**Correspondence:** Sally Havers

*Antimicrobial Resistance and Infection Control* 2017, **6(Suppl 3):**P123

**Introduction:** Infection control practitioners (ICPs) need to be responsive to health policy changes and regulatory requirements, however how this is achieved from both a practice and resource perspective is rarely investigated. In 2010 new infection control policy requirements were introduced to all Australian hospitals regarding the practice and monitoring of aseptic technique (AT).

**Objectives:** This study aimed to investigate ICP perspectives on implementation activities associated with this change to identify the contextual factors that influenced this process.

**Methods:** ICPs from three Australian states were invited to participate in four focus groups in February of 2015. Purposeful sampling provided representation from public and private, regional and metropolitan services. All participants had been involved in implementation of AT policy in their hospital. Questions were based on the Consolidated Framework for Implementation Research focussing on identifying contextual factors that had influenced policy implementation. Following transcription, a thematic analysis using interpretive description was undertaken to determine key themes.

**Results:** Analysis identified seven common contextual factors that influenced organisation implementation. These included organisational factors such as role and responsibility allocation, leadership and culture. External contextual factors of influence included regulatory functions (such as accreditation) as well as policy content and the trigger for policy introduction. Resourcing, preparedness and sustainability were also important themes.

**Conclusion:** Common contextual factors affect ICPs implementing national infection control policy in a variety of hospital settings and locations. To improve the effectiveness and process of policy implementation a better understanding of these issues is important for policy makers, managers and clinicians.

**Disclosure of Interest**

None Declared

### P124

#### Review of video-reflexive ethnography in healthcare

Simone Passerini[1], Tanja Manser[2], Hugo Sax[1], Lauren Clack[1]

[1]Infectious Diseases and Hospital Epidemiology, University Hospital Zurich, Zurich, Switzerland; [2]Institute for Patient Safety, University Hospital Bonn, Bonn, Germany

**Correspondence:** Simone Passerini

*Antimicrobial Resistance and Infection Control* 2017, **6(Suppl 3):**P124

**Introduction:** Video-reflexive ethnography (VRE) is an innovative methodology that can be used to engage hospital staff and patients in quality improvement efforts. It begins with video-ethnography, filming live care sequences. Selected footage is then shown back to participants in a 'reflexive session', during which they are prompted to reflect on the care shown and to consider opportunities for improvement.

**Objectives:** We undertake a literature review to examine the ways in which video-reflexive ethnography has been employed to improve healthcare quality.

**Methods:** This systematic review was conducted according to the PRISMA guidelines. Medline, PyschINFO and PsychARTICLES databases were searched using keywords related to "video-reflexive ethnography" and "healthcare" for articles published in English language up until 15.03.2017. Inclusion criteria were: study population of healthcare workers, patients, or family members; in-situ filming in healthcare settings; followed by a reflexive session using video footage as a prompt.

**Results:** Our search strategy identified 311 studies, of which 10 met inclusion criteria and were included this review. Studies took place in acute care hospitals (surgical, medical, maternity, emergency, and intensive care wards) an outpatient clinic, and a palliative day hospital. Nine studies employed VRE together with additional observations or field interviews. Reflexive sessions were made with single interviewees (3), pairs (3) or focus groups (6). Topics addressed included team communication, medication management, childbirth, end-of-life care, handovers, infection prevention, and patient safety. Authors frequently reported that the successful execution of VRE required careful preparation, including ethics approval and participant recruitment and informing.
**Conclusion:** This review shows that VRE has been used in a variety of healthcare settings to address a broad range of quality improvement and patient safety issues. We conclude that video-reflexive ethnography appears to be a promising method for engaging healthcare workers and patients in the improvement of healthcare quality.
**Disclosure of Interest**
None Declared

#### P125
### Coaching approach to promote behaviour change in infection prevention and control in the health district of Kindia, Republic of Guinea

Mamadou Malal Diallo[1], Abdoulaye Diallo[2], Yolande Hyjazi[3], Aly Doumbia[4], Jacqueline Aribot[5]
[1]Pre-service education; [2]Suivi Evaluation; [3]Directrice Pays; [4]Prevention et Controle de l'Infection; [5]Suivi -Evaluation, Jhpiego, Conakry, Guinea
**Correspondence:** Mamadou Malal Diallo
*Antimicrobial Resistance and Infection Control* 2017, **6(Suppl 3):**P125

**Introduction:** Onsite coaching approach of health care providers in Infection Prevention and Control (IPC) has been implemented in 14 health facilities in the district of Kindia. The process aimed at improving health care providers' behaviour in IPC practices.
**Objectives:** Assist providers to improve their skills in IPC during health care service provision.
**Methods:** Observation of health workers while implementing IPC skills according to standards. Provide immediate feedback to providers and managers to address gaps and improve their skills.
**Results:** Among trained providers (n = 448), 6 local supervisors and 2 focal points were identified according to their performance in the application of PCI measures. The coaching visits reached 274 health care providers including support staff. 194/274 (71%) practiced competencies according to performance standards, of which 121/156 (78%) were reached in health centers, 46/85 (54%) in the regional hospital, and 27/33 (82%) support staff. The poor performance observed in the hospital is related to the lack of regular use of performance standards for self-assessments and internal evaluations.
**Conclusion:** Onsite coaching visits contribute to address poor performance; however behaviour change in the practice of IPC measures and knowledge to prevent the risks of infection for the health care providers and health facility users are still a major challenge of the health system.

**References**
Jhpiego/Johns Hopkins Medicine, Prevention et Controle de la maladie a virus ebola dans les etablissements de sante aux ressources limitees (Manuel de reference, octobre 2014) - Jhpiego/Ministere de la Sante, Standards de Performance en Prevention et Controle de l'Infection (Octobre 2014)
Disclosure of Interest
None Declared

### Healthcare workers' education

#### P126
### Train the trainer concept – a standardised approach to guide hand hygiene training

Ermira Tartari[1], Daniela Pires[1], Carolina Fankhauser-Rodriguez[1], Marcella Hernández-de Mezerville [2], Américo Agostinho[1], Fernando Bellissimo Rodrigues[3], Didier Pittet[1]
[1]WHO collaborating centre for Patient Safety, Infection Prevention &Control, University Hospitals of Geneva, Genève, Switzerland; [2]Hospital Nacional de Niños de Costa Rica Dr. Carlos Sáenz Herrera, San José, Costa Rica; [3]Universidade de São Paulo, Faculdade de Medicina de Ribeirão Preto, Ribeirão Preto, San Paolo, Brazil
**Correspondence:** Ermira Tartari
*Antimicrobial Resistance and Infection Control* 2017, **6(Suppl 3):**P126

**Introduction:** Harmonisation in hand hygiene (HH) training for infection control professionals (ICPs) is lacking.
**Objectives:** To describe a standardized approach to HH training, using a "Train-the-Trainers" (TTT) concept for both national and facility-based ICPs and its impact on HH knowledge.
**Methods:** This training course based on the World Health Organization (WHO) HH multimodal strategy, consists of lectures, hands-on practice and sharing of experiences. The theoretical part addresses HH related concepts such as burden of healthcare-associated infections, multimodal improvement strategy, approaches dealing with campaign fatigue and updates on HH research. The hands-on training focus on direct monitoring of HH compliance, using visual video reviewing of clinical scenarios and role-plays encouraging participation and feedback. A pre- and post-course questionnaire assessed baseline knowledge and that acquired during the course. The Wilcoxon signed-rank and the McNemar's statistical tests were used to compare the pre and post results from the questionnaires.
**Results:** The HH "TTT" course was organized in Brazil (2015), Spain (2016), and scheduled in 2017 for South Africa, Mexico, Malaysia, and Mashhad. 33 and 21 ICPs attended the course in Brazil and Spain, whilst between 30 and 40 ICPs are expected to the following courses. HH knowledge of participants showed a significant increase in the rate of correct answers from 77.0% to 89.7% in Brazil ($P < 0.001$) and from 65.3% to 75% in Spain ($P < 0.001$). Positive aspects reported were: learning environment, sharing experiences, hands-on practices and networking among ICPs.
**Conclusion:** The "TTT" training course based on the WHO HH multimodal improvement strategy and expert experience at HUG proved to be effective in enhancing participant's knowledge, sharing experiences and networking. Ultimately, this reference training method can be used by ICPs worldwide to further disseminate knowledge to other health care workers.
**Disclosure of Interest**
None Declared

#### P127
### Competency based infection prevention and control training of healthcare workers in Ghana: a systems approach

Kofi H. Asare[1], Chandrakant Ruparelia[2], Eva Ahiataku[1], Jacob Ayetey[1]
[1]MCSP, JHPIEGO, ACCRA, Ghana, [2]Technical Leadership Office, JHPIEGO, Baltimore, United States
**Correspondence:** Kofi H. Asare
*Antimicrobial Resistance and Infection Control* 2017, **6(Suppl 3):**P127

**Introduction:** Since 2015, Jhpiego, an affiliate of Johns Hopkins University is providing technical assistance to the Ghana Health Services (GHS) under the Maternal and Child Survival Program Funded by USAID.
**Objectives:** The project aims to build the capacity of healthcare workers (HCWs) to improve the quality of Infection Prevention and Control (IPC) practices at health facilities in 5 regions and thereby contribute to the reduction in healthcare associated infections and protect the HCWs from blood borne and other infections.
**Methods:** The project uses a Competency Based Training Model (CBTM) to train HCWs in IPC using onsite training approach. Jhpiego worked with GHS and other partners to conduct a competency analysis for IPC for all cadre of staff at health facilities. This was followed by updating the National IPC policy and guidelines, developing IPC facilitator's guide and training of trainers. The approach to prepare trainers included technical updates, standardizing IPC skills and strengthening of the competency based training skills. The national and the regional trainers co-trained initial IPC courses with Jhpiego trainers and received coaching and mentoring during co-training. A trainers WhatsApp group was established to share experiences. For rolling out the training in the regions we followed decentralized strategy and supported the regional directorate of health services to implement training. This strategy comprised of the regions signing MOUs to manage grants with clear deliverables, implementation plans and submitting weekly reports. The regional trainings at hospitals were supported by monthly supervisory monitoring and evaluation visits. The project targeted 13,975 HCWs in 56 hospitals.

**Results:** In all, 24 national and 151 regional trainers were developed to provide supporting supervision and roll out of trainings in their regions. As of March 2017, 3,603 healthcare workers in 13 health facilities have been trained. Average knowledge test scores increased from a baseline of 45% to 70% post training.

**Conclusion:** This approach to training healthcare workers is an effective way of improving their competency in IPC. It is impactful in strengthening healthcare systems in poorly resourced countries by helping to train large number of HCW in a short time.

**Disclosure of Interest**

None Declared

### P128

#### Tools for evaluating graduate infection control programs

Donna J. Haiduven
Global Health, UNIVERSITY OF SOUTH FLORIDA COLLEGE OF PUBLIC HEALTH, Tampa, United States
*Antimicrobial Resistance and Infection Control* 2017, **6(Suppl 3):**P128

**Introduction:** Both a graduate certificate and Master's in Public Health (MPH) Program with Infection Control concentration have been offered together since August 2013. Evaluation of these courses and programs is essential for those interested in the education of current and future Infection Preventionists.

**Objectives:** The objectives of this project were to develop, implement and evaluate tools for assessing the infection control courses and programs, and use the results to modify the courses and programs as necessary and indicated.

**Methods:** The following tools were used to evaluate the graduate infection control programs: 1) initial course survey, 2) mid-semester survey, and 3) final course evaluation, for the four certificate/MPH courses, and 4) exit interview. The surveys used a combination of objective Likert-scale questions as well as open-ended questions regarding student expectations. Exit interviews consisted of open-ended questions about the program and experience of the student. Descriptive statistics were used to evaluate the objective questions and all subjective comments were reviewed, with common themes identified.

**Results:** Two-hundred fifteen (92.3%) of students completed mid-semester surveys of the courses, with 48.4% extremely satisfied and 38% very satisfied with overall knowledge gained. A total of 330 students completed the final course evaluations, with a mean overall rating of 4.67/5.0 for course instruction. Individual course ratings of each survey analyzed as well. Common themes for the courses' subjective ratings included positive comments about the variety of options for viewing the lecture materials and the ability to work ahead in the courses. Negative comments included the length of the exams and the number of required readings. Exit surveys indicated that there were several different student categories with differing infection control educational needs. Extensive additional ratings categories for the four survey tools and a complete review of subjective comments resulted from the analyses.

**Conclusion:** Together, the evaluation tools provided invaluable information at both individual course and entire program levels. Courses were modified in existing and subsequent semesters to accommodate identified issues. Those interested in educating Infection Preventionists may find the structure and results of the individual evaluation tools useful and applicable in their educational programs.

**Disclosure of Interest**

None Declared

### P129

#### Infection prevention and control personnel in Bavarian Hospitals: development since 2013

Ulla Kandler[1], Silke Nickel[1], Verena Lehner-Reindl[1], Christiane Höller[2]
[1]Hygiene, Bavarian Health and Food Safety Administration, Erlangen; [2]Hygiene, Bavarian Health and Food Safety Administration, Oberschleißheim, Germany
**Correspondence:** Ulla Kandler
*Antimicrobial Resistance and Infection Control* 2017, **6(Suppl 3):**P129

**Introduction:** Infection prevention and control is a major concern in hospitals and specialized professionals are needed to implement appropriate measures to meet these challenges.

The Commission for Hospital Hygiene and Infection Prevention (KRINKO) has the official mandate to develop national recommendations for the prevention of healthcare-associated infections in Germany and published two recommendations on the requirement of specialized personnel in hospitals in the year 2009 and 2016, which had a major impact on legislation and the hospitals themselves.

**Objectives:** To emphasize the importance of the recommendations by the KRINKO and to detect potential possibilities for improvement, the Bavarian Health and Food Safety Authority (LGL) decided to examine the actual situation concerning infection prevention and control staff in Bavarian hospitals on a regular basis. The first survey on this behalf was conducted in the year 2011, further studies followed in 2013, 2015 and 2016.

**Methods:** The department of hospital hygiene and infection prevention at the LGL created a questionnaire which was sent to all local health administrations in Bavaria. The health administrations were asked to distribute the questionnaire to the hospitals in their district, to collect the completed forms after a period of two months and to return them to the LGL. The collected data were read into a data base, were validated and are currently analyzed.

**Results:** The present publication will show the results from the past years and demonstrate how the situation changed over the years. A comparison between the actual numbers of infection prevention and control staff and the recommended numbers from the KRINKO will be drawn to present the possibilities for further improvement.

**Conclusion:** The recommendations made by the KRINKO in 2009 and 2011 had an impact on legislation and the engagement of specialized staff became obligatory for hospitals and institutions, which provide care comparable to hospitals. The demand of qualified staff is still higher than the supply, but initiatives to qualify more persons in infection prevention and control have been started since the year 2010 and show positive effects.

**Disclosure of Interest**

None Declared

### P130

#### Evaluation of basic infection prevention practices in health care set-ups in Benin

Theodora Ahoyo[1], Dorothée Kinde Gazard[2], Marielle Gounongbe[1]
[1]BIOLOGIE HUMAINE, EPAC/UAC, ABOMEY CALAVI; [2]parasitologie, faculté de medecine, cotonou, Benin
**Correspondence:** Theodora Ahoyo
*Antimicrobial Resistance and Infection Control* 2017, **6(Suppl 3):**P130

**Introduction:** In the context of Viral Hemorrhagic Fever diseases (Ebola and Lassa) outbreak in West Africa in 2014, WHO and other international partners, have initiated and supported the implementation of a voluntary program to prevent infection in all affected countries. As part of this initiative an invitation to participate to the national promotion of contact precautions was sent to all acute hospitals by the Ministry of health of Benin republic.

**Objectives:** We assessed the efficacy of Contact Precaution practices, including, feasibility and adaptability to the local context.
**Methods:** during 35 weeks from March 2016 all hospital registered (32) received hygiene equipment and materials (running water and soap; alcohol based rubs) followed by an extensive campaign among personnel for the respect of infection transmission based precautions complemented with a semi-structured interview and direct observations. Hand hygiene audits were conducted using the WHO 'five moments for hand hygiene' observational tool to measure hand hygiene observance and hand washing technique.
**Results:** only 81% of registered hospital took part in exercice. On average, through the study period, hand washing rates increased from 22% to 47% $P = 0.0002$). 76% of personnel preferred hand washing with soap and water. The proportion knowledgeable about hand hygiene practices was 71%. The availability of individual protective equipment and running water varied from hospital to hospital (21% to 51%). Standard precautions and practices related to avoiding cross infection were not always complied height months later
**Conclusion:** the levelof contact precautions application differed importantly between health workers and between hospitals. Adherence to WHO guidelines was low despite good knowledge. The lack of qualified personnel and financial resources allocated for this program is one of the causes of weak compliance.
**Keywords: Compliance, hand hygiene, Healthcare workers, standard precaution, Benin**

References
Pittet D, Allegranzi B, Storr J, Bagheri Nejad S, Dziekan G, Leotsakos A. Infection control as a major World Health Organization priority for developing countries. J Hosp Infect. 2008;68:285–92
Disclosure of Interest
None Declared

### P131

#### Compliance of personal protective equipment in a hospital

Ju Ae Lee[1,2], Jeong Hwa Choi[2], Hyun Kyun Ki[2,3]
[1]Department of Clinical Nursing, University of Ulsan Graduate School of Industrial Technology; [2]Department of Infection control; [3]Division of Infectious Diseases, Konkuk University Medical Center, Seoul, Korea, Republic Of
**Correspondence:** Ju Ae Lee
*Antimicrobial Resistance and Infection Control* 2017, **6(Suppl 3):**P131

**Introduction:** Due to the epidemic of new high-risk pathogens such as MERS-CoV, training of health-care workers to wear personal protective equipment (PPE) has become a necessity.
**Objectives:** We evaluated the compliance of wearing and removal of level D PPE and N95 respirator to promote appropriate PPE wearing.
**Methods:** We selected 120 doctors and nurses of respiratory diseases department, and evaluated 16 sessions by 7-8 persons in June of 2016. We provided videos and protocols for correct wearing before the evaluation of compliance to allow individual practice. Evaluation of the compliance was made using the checklist of protocols PPE. Contamination during removal of PPE evaluate was done by spraying a liquid fluorescent material onto level D PPE (N95 respirator, goggles, chest, hands, and legs) and contaminated region was checked by using ultraviolet lighting. N95 respirator fit testing was selected among 3 products in the hospital and leakage rate was checked through the mask fit tester.
**Results:** The compliance rate of level D PPE wearing was 80.0% (96/120) and the compliance rate of removal was 30.8% (37/120). The compliance rate of N95 respirator donning 79.2% (95/120). During level D PPE removal, more than one site of fluorescent material contamination was 82.5% (99/120). The most predilection sites of the contamination were face (58.3%) and occipital of head (30.8%). The two N95 respirators showed a fitting rate of less than 50%. There were statistical differences in the fitting rate among the three types of the N95 respirators ($P < 0.01$).
**Conclusion:** Appropriate level D PPE removal was done by a very small number of the participants.To prevent the observed exposures, additional use of face shield, hat, apron, and long-sleeved gloves would be necessary. Depending on the shape of the face, the fitting rate of each product is different, so various kinds of N95 respirator should be provided. Repeated training and expansion of the training chances for hospital staffs should be improved of appropriate PPE wearing and removal.
**Disclosure of Interest**
None Declared

### P132

#### Medical students' knowledge about Ebola infection control measures

Ljiljana Markovic-Denic[1], Jovana Todorovic[2], Olivera Djuric[1], Janja Bojanic[3], Biljana Mijovic[4], Vuk Marusic[1], Dusan Djuric[5]
[1]Institute of Epidemiology; [2]Institute of Social Medicine, FACULTY OF MEDICINE, UNIVERSITY OF BELGRADE, Belgrade, Serbia; [3]University of Banja Luka, Faculty of Medicine, Banja Luka; [4]University of East Sarajevo, Faculty of Medicine, Foca, Bosnia and Herzegovina; [5]University of Kragujevac, Faculty of Medical Sciences, Kragujevac, Serbia
**Correspondence:** Ljiljana Markovic-Denic
*Antimicrobial Resistance and Infection Control* 2017, **6(Suppl 3):**P132

**Introduction:** Last Ebola virus disease (EVD) outbreak was the largest and most geographically dispersed Ebola outbreak known to date. During such large outbreaks, with pandemic potential, it is expected that medical students would be included in patient treatment. Their knowledge and awareness may influence the prompt and adequate implementation of infection control measures.
**Objectives:** To investigate preparedness, and the knowledge level about infection control measures of Ebola among medical faculty students.
**Methods:** A cross-sectional study was conducted among fourth-year and sixth-year students at four state medical faculties in two countries: Serbia (in Belgrade and Kragujevac) and Bosnia & Herzegovina (in Banja Luka and Foca). A self-administered, standardized, anonymous questionnaire containing data about social and demographic characteristics of students, data about epidemiological characteristics of EVD, and knowledge of infection control measures, was used.
**Results:** A total of 1089 students (53.6% of the fourth year and 46.4% of the sixth year) were included in the study. Male to female ratio was 0.51. Most of the respondents (91.6%) correctly identified contact with blood and body fluids as the most important nosocomial mode of Ebola virus transmission, and 86.6% indicated that wearing of personal protective equipment (PPE) is the most important preventive measures in hospitals. However, out of 8 parts of PPE recommended for health care workers caring for patients with EVB, students reported 3,5 (±1.82) parts on average, the fourth year students having better knowledge ($4.0 \pm 1.89$) ($p = 0.001$). Further, only 39.3% of students, statistically higher percent of the fourth year students ($p < 0.001$), recognized that a trained observer must supervise each step of PPE donning/doffing. Nevertheless, higher proportion of fourth year students (56.5%, $p < 0.001$) expressed the needs for additional education about EVB infection control measures.
**Conclusion:** This study indicated a gap in knowledge on infection control of EVD can be filled by education on emerging and re-emerging infectious diseases in the curricula.
**Disclosure of Interest**
None Declared

### P133

#### Prevention and control of infections risks in microbiology laboratories in Benin

Theodora Ahoyo, Eric C. koutchica, Perince F. Fonton
BIOLOGIE HUMAINE, EPAC/UAC, ABOMEY CALAVI, Benin
**Correspondence:** Theodora Ahoyo
*Antimicrobial Resistance and Infection Control* 2017, **6(Suppl 3):**P133

**Introduction:** Microbiology laboratories are unique environments in relation to the safety of those who work within them. Clinical specimens received from patients pose a hazard to personnel and sometimes their families because of infections agents they contains. In context of Lassa disease in Benin during 2015, an intensive campaign to promote good safety practices was performed throughout all specialty areas of the laboratory by motivational communication.
**Objectives:** to determine whether using motivational communication would improve biosecurity compliance in laboratory
**Methods:** this was a three months of multicenter prospective observational of variation in laboratories practices related to prevention of laboratory acquired infection. Process information about biosecurity (standards precaution, barriers used during sample collection and biosafety level) was collected from 1rst Jun to 30 August.
**Results:** ten hospital clinical microbiology laboratory participate, 83 biotechnologists and 21 others workers were enrolled. Only 1 laboratory can be classified in biosafety level II. The first result show an improvement of practices in particular regarding standards precaution practices from baseline globally 45% to 65% ($p < 0.0006$), most of personnel 85% wore masks and gowns 64%, hand hygiene procedures was performed, either by washing hands with soap and water 80% or with alcohol-based formulation 12%.
**Conclusion:** the use of motivational communication to provide education increased compliance to standards precaution can encourage staff participation in learning. Those taking part become personally involved and take an active role in reviewing or acquiring skills.
**Disclosure of Interest**
None Declared

### P134

#### A step towards health care worker safety by reducing the risk of sharp injuries

Zohra R. Rafique Iqbal, Rozina Roshan, Faisal Mahmood
Infection Control, Aga Khan University and Hospital, Karachi, Pakistan
**Correspondence:** Zohra R. Rafique Iqbal
*Antimicrobial Resistance and Infection Control* 2017, **6(Suppl 3):**P134

**Introduction:** Sharp injuries are major occupational health and safety issues globally. Health care workers (HCW) are on highest risk of getting blood borne pathogen including hepatitis B (HBV), hepatitis C (HCV), and human immunodeficiency virus (HIV) due to contaminated needles and sharp objects.
**Objectives:** It has been observed that there was a sudden rise of sharp injuries from unknown source**.** Thus Infection Control Department (IC) aimed to:
Reduce number of exposure from sharp injuries by educational training and safeguard interventions.
Decrease exposure of sharp injuries from unknown source which will automatically decreases cost of hospital and stress of HCW.
**Methods:** The PDSA Plan-Do-Study-Act model, a continuous quality improvement (CQI) tool, was used to decrease the preventable exposure to sharp injuries. P**lan**: it was observed that number and rate of sharp injuries has drastically increased in Qtr. 1 2016 i.e. 53 to 83 from 16.7 to 12.0 respectively. Moreover it has been observed that exposure from unknown source are also increasing i.e.11 to 18 which increase risk of acquiring blood borne diseases and hospital's cost. The data was analyzed by interviewing all exposed staff and identified the loop hole to prevent sharp injuries. **Do**: educational sessions were conducted, reinforcement was done to avoid recapping, flyers circulated to all patient care areas, disseminate pocket guide to HCW, fixed danger box in brackets to avoid spillage, introduce safe lancet to check blood glucose, **Check**: Observed staff practices and gave one to one teaching to improve practices, malpractices were notified to supervisors. **Act:** Increase frequency of audits to ensure safe disposal of sharps. Monthly sharing of exposure data with supervisors.
**Results:** A significant decline was observed in Q3 2016 the number of sharp injuries exposure decreased from 83 to 59 simultaneously rate decrease from 16.7 to 12.0. The numbers of exposure from unknown source decreased from 18 to 06.The target we set for exposure to sharp injury from **unknown source** below 10 and we were able to achieve 06 and the cost burden was lessened from three hundred sixty thousand (PKR 360,000) to one hundred twenty thousand (PKR 120,000) which means two times cost reduction.
**Conclusion:** Educational session are key component to improve staff practices as its a matter of their own occupational safety.
**Disclosure of Interest**
None Declared

## Surveillance of healthcare-associated infections (HAI)

### P135

#### Healthcare-associated bloodstream infections in Finland – adjusted ranking of hospitals by staphylococcus aureus rates and effect of infection control resources and practices on rankings

Emmi Sarvikivi, Jukka Ollgren, Tommi Kärki, Outi Lyytikäinen
NATIONAL INSTITUTE FOR HEALTH AND WELFARE, Helsinki, Finland
**Correspondence:** Emmi Sarvikivi
*Antimicrobial Resistance and Infection Control* 2017, **6(Suppl 3):**P135

**Introduction:** Healthcare-associated bloodstream infections (BSI), especially those caused by *Staphylococcus aureus* (SA-BSI) are often preventable. Rate of SA-BSI has been used as an indicator for hospital performance in infection control (IC).
**Objectives:** Our aim was to analyze the Finnish surveillance data in order to assess hospital rankings according to crude and adjusted rates of SA-BSI, and to investigate if IC resources and practices in the hospitals were reflected in the rankings.
**Methods:** 20 Finnish hospitals conducted prospective incidence surveillance for healthcare-associated BSIs in 1999-2015. A common protocol for laboratory-based case finding was used, and only BSIs with onset >48 h after admission were included. Patient-days with specialties were obtained from hospitals' databases to calculate incidence densities (ID) with 95% confidence intervals (CI). The hospitals' crude IDs and IDs adjusted by specialties in mixed effect's negative binomial regression model were calculated; rankings of hospitals were assessed by standardized morbidity ratio (SMR) rank method. The effects in the model were considered to be constant over selected time period. Results of a national survey on IC resources and practices in each hospital were also evaluated in the model. Analyses were performed by Stata 14.2 and WinBUGS 1.4.3.
**Results:** We identified 13942 BSIs, including 1966 SA-BSIs. For SA-BSIs, IDs varied between hospitals from 0 to 0.12 per 1000 patient-days. There were clear differences in crude and adjusted ranking positions of hospitals, but CIs were wide and mostly overlapped. Of the specialties, surgery seemed to increase risk for SA-BSIs ($p = 0.02$). The IC survey was completed by 17 hospitals. In the model, number of blood cultures per 1000 patient-days, and catheter-related procedures (e.g., guidelines, check-lists) remained significant ($p = 0.013$ and $p = 0.015$, respectively). If the hospital had implemented several of catheter-related procedures, the protective effect seemed to strengthen.
**Conclusion:** Adjusting by specialties may be needed when ranking SA-BSI rates. However, the rankings must be interpreted with caution.

Standardized procedures regarding catheters appeared to be beneficial in prevention of SA-BSIs.

**Disclosure of Interest**

None Declared

### P136

#### Bacteriemia diagnosed in a infectious diseases clinic in Dakar

Ndeye Aïssatou Lakhe[1,2], Khardiata Diallo Mbaye[3], Viviane M P Cisse Diallo[4], Daye Ka[4], Louise Fortes Deguenonvo[4], Cheikh Tidiane Ndour[4], Moussa Seydi[4]

[1]Service des Maladies Infectieuses, Service des Maladies Infectieuses/ Centre Hospitalier de Fann/; [2]Faculté de Médecine, Université Cheikh Anta Diop; [3]Service des Maladies Infectieuses, Centre Hospitalier de Fann/; [4]Service des Maladies Infectieuses, Centre Hospitalier de Fann, DAKAR, Senegal

**Correspondence:** Ndeye Aïssatou Lakhe

*Antimicrobial Resistance and Infection Control* 2017, **6(Suppl 3):**P136

**Introduction:** Bacteremia have important consequences in terms of mortality, morbidity and cost. Their prognosis depends on several factors, among them, the rapidity and above all the efficacy of first line antibiotics.

**Objectives:** The objective of our study was to describe the epidemiological, clinical, therapeutic and evolution aspects of bacteraemia.

**Methods:** We realized a descriptive retrospective study based on records of patients hospitalized in the department of infectious and tropical diseases, for which bacteraemia was diagnosed from 1 January 2013 to 31 December 2014.

**Results:** Seventy nine (4.11%) cases of bacteraemia were collected among the 1922 hospitalized patients and 85 strains had been isolated. The female predominance was clear with 44 cases (55.9%) The mean age of the study population was 26.97 ± 16.49. The most frequent reasons for hospitalization were sepsis in more than half of cases (55.69%). Tuberculosis was the most frequent diagnosis associated with 15 cases (18.98%). The portal was mostly urinary (21 cases). The most common agents isolated were Staphylococci (31.76%), Pseudomonas (15.29%), streptococci (9.41%), Escherichia (8.23%) and Acinetobacter (8.23%). The strains isolated were sensitive to imipenem, vancomycin, fusidic acid, piperacillin, colistin (except Pseudomonas) in more than 90%. The majority of patients (69.6%) had received two or more antibiotics. Thirty-three patients (41.77%) died.

**Conclusion:** Bacteremia in our context has a high mortality. The isolated bacterial strains are increasingly resistant to the antibiotics available. Rational use of antibiotics is more necessary than ever.

**Disclosure of Interest**

None Declared

### P137

#### A comprehensive inventory of infectious risk moments during acute care

Lauren Clack[1], Simone Passerini[1], Tanja Manser[2], Hugo Sax[1]

[1]Infectious Diseases and Hospital Epidemiology, University Hospital Zurich, Zurich, Switzerland; [2]Institute for Patient Safety, University Hospital Bonn, Bonn, Germany

**Correspondence:** Lauren Clack

*Antimicrobial Resistance and Infection Control* 2017, **6(Suppl 3):**P137

**Introduction:** The practice of hand hygiene (HH) has been well studied over the last decade for its role in infection prevention, yet less knowledge exists regarding other important infection-related behaviours. We hypothesize that an important portion of infectious risks lies at "infectious risk moments" (IRM) – seemingly innocuous, yet frequently occurring care manipulations resulting in transfer of pathogens – which include and go beyond indications for hand hygiene.

**Objectives:** We conducted 1) *exploratory observations* to establish a comprehensive inventory of IRM, and 2) *structured observations* to quantify the frequency and nature of IRM.

**Methods:** *Exploratory observations* were carried out in three settings (intensive care, medical, emergency) by single observers, who noted the care process being observed and any potential IRM. An inventory of all observed IRM was established and systematically coded according to the source, pathway, and endpoint from, through, and to which pathogens were transferred, respectively. This formed the taxonomy for the *structured observations,* which were carried out using a mobile observation tool programmed with Filemaker 14 in the same three settings by two observers to ensure systematic documentation of all IRM.

**Results:** 129.17 hours of *exploratory observations* resulted in the identification of 292 unique potential IRM. IRM were categorized according to the source (e.g. medical device, mobile object, physical environment), pathway (e.g. hands, gloves, mobile objects) and endpoint (e.g. patient critical site, non-critical site) of pathogens. 53.77 hours of *structured observations* (31.25 hours of active care) resulted in the identification of 1'338 potential IRM, for an average density of 42.8 IRM/active care hour. The average densities of IRM/ active care hour were 34.9, 36.8, and 56.3 in the intensive care, medical, and emergency wards, respectively. 566 unique moments were identified, which fell into 71 main categories.

**Conclusion:** Exploratory and structured observations resulted in the successful identification of IRM including, yet going beyond indications for hand hygiene to include moments involving glove use, mobile objects, medical devices, healthcare worker clothing, and invasive devices. This inventory may serve as a tool for identifcation and prioritization of future infection prevention efforts.

**Disclosure of Interest**

None Declared

### P138

#### Assessing the burden and trends of healthcare associated infection in a tertiary care hospital through point prevalence survey

Rushika Saksena[1], Shilpee Kumar[1], Rajni Gaind[1], Sunita Nagpal[2], Premrose Suri[2]

[1]Microbiology; [2]Infection control, Vardhman Mahavir medical college and Safdarjung hospital, Delhi, India

**Correspondence:** Rushika Saksena

*Antimicrobial Resistance and Infection Control* 2017, **6(Suppl 3):**P138

**Introduction:** To obtain basic Healthcare-Associated Infections (HAIs) information, such as prevalence, isolated pathogens and type of HAI, cross-sectional survey is a relatively cost-effective method. Repeated cross sectional surveys can also provide meaningful data to investigate potential trends of HAIs.

**Objectives:** To understand HAI epidemiology and to improve infection control program, four annual point prevalence surveys were conducted from 2012 to 2016.

**Methods:** Settting - 1531 bedded, tertiary care, government hospital with 8 ICUs and 41 wards (21 clinical departments)

We conducted 4 annual point prevalence surveys on HAI from 2012 to 2016. Before the survey, the questionnaires and definitions of the investigated variables in the questionnaire were circulated to all linked-nurses. On the day of survey, all in- patients were surveyed and questionnaires were completed. The data was extracted from all relevant sources including clinical records, temperature charts, laboratory reports and radiographs. Trained Infection control nurses reviewed medical records retrospectively to identify HAIs active at the time of the survey.

**Results:** A total of 5194 patients were included (1229 patients in 2012, 1447 in 2014, 1311 in 2015 and 1207 in 2016). Overall prevalence of HAI showed a decreasing trend over 4 years (7.4, 5.3, 8.8, & 5.9% respectively). Most departments have HAI rate varying between 0 and 10% except ICUs (44-62%) and burn & plastic wards (22-27%). Wound infections (41-47%) are the most common HAI in all the years. The rate of UTI has an upward trend (23, 12, 24 & 38% respectively) while rate of BSI has downward trend (15, 18, 11& 5% respectively). Gram-negative bacteria (*E.coli, Klebsiella & Acinetobacter*) are most isolated pathogens. There is increased rate of isolation of *E.coli* (15, 11, 21 & 25% respectively) and *Acinetobacter* (18, 10,13 &19% respectively) while decreased rate of isolation of *S.aureus* (15,16,13 & 4% respectively) over the years.

**Conclusion:** Despite the limitations of Point prevalence survey, our study yielded valuable data on the epidemiology of HAI. The study underscore the surveillance of HAI by repeated prevalence surveys for a better understanding of trends in the epidemiology of HAIs and prevention success.

**Disclosure of Interest**

None Declared

### P139

#### Healthcare associated infections: prevalence and risk factors

Héla Ghali, Sihem Ben Fredj, Salwa Khefacha, Mohamed Ben Rejeb, Houyem Said Latiri

Departement of Prevention and Security of Care, Sahloul-Sousse, Tunisia

**Correspondence:** Héla Ghali

*Antimicrobial Resistance and Infection Control* 2017, **6(Suppl 3):**P139

**Introduction:** Healthcare associated infections (HAI) are the most frequent adverse event in health-care delivery worldwide. The endemic burden of HAI is also significantly higher in low- and middle-income than in high-income countries, in particular in patients admitted to intensive care units and in neonates. Tunisia is not spared from this scourge. The national surveys of prevalence HAI were held regularly in the health institutions in the framework of the strategic axis "Surveillance of HAI" of the National Strategy of the hygiene and safety of care.

**Objectives:** Our study aimed to evaluate the prevalence of HAI in the university hospital of center-east of Tunisia and to assess its risk factors.

**Methods:** A cross-sectional study of prevalence was conducted from April 11$^{th}$ to April 28$^{th}$ 2016. All services have been included in the investigation, except those of emergencies and of haemodialysis due to their very short duration of hospitalization. The collection of data was performed by the plug of the survey of national prevalence of nosocomial infections.

**Results:** Among 287 eligible patients, 28 had a HAI. The prevalence of patients infected was 9.8%. 33 HAI have been identified in these patients. The prevalence of HAI was 11.5%. This prevalence was more important in intensive care units (29.2%). The most frequent infections were respiratory infections (39.4%) and urinary tract infections (18.18%). The multivariate analysis of intrinsic and extrinsic factors has highlighted a significant association between the HAI and the presence of central venous catheter (OR = 4.11; IC 95% = [1,04 – 16,28] ; p = 0,044), setting of prosthesis (OR = 28,80; IC 95% = [4,44 – 186,42] ; p = <10-3), the artificial nutrition(OR = 4,34; IC 95% = [1,03 – 18,23] ; p = 0,045), and the antibiotic therapy in the past 6 months (OR = 2,78; IC 95% = [1,09 – 7,09] ; p = 0,032). 16 infections were microbiologically documented (57.14%). In total, 21 pathogens have been identified, which the most frequent was Klebsiella pneumoniae.

**Conclusion:** Actions of prevention of HAI have already been conducted at the hospital. The results of this survey suggest a strengthening of these actions, including: the improvement of the system of monitoring, the promotion of the hygiene of hands, the training of the health staff and the quality approach relative to the safety of care.

**Disclosure of Interest**

None Declared

### P140

#### Outbreak of lassa fever in a healthcare setting : epidemiologic surveillance and risk stratification for contact persons - Saki Oyo State, Nigeria 2014

Olukemi T. Olugbade[1], Oluwaseyi Israel[1], Rhoda Fadahunsi[1], Abiodun Ogunniyi[1], Abisola Oladimeji[1], Adebola Olayinka[1,2]

[1]Field Epidemiology Practice, Nigeria Field Epidemiology and Laboratory Training Programme, Asokoro; [2]Medical Microbiology, Ahmadu Bello University, Zaria, Nigeria

**Correspondence:** Olukemi T. Olugbade

*Antimicrobial Resistance and Infection Control* 2017, **6(Suppl 3):**P140

**Introduction:** Lassa Fever (LF) is an infectious viral hemorrhagic disease transmitted through contact with effluents (fecal matter and urine) of *Mastomys natalensis* - a multi-mammate rat species, and infected persons.LF is endemic in West Africa. Outbreaks of LF occurred in 23 of the 36 states in Nigeria with 1,656 cases and 112 deaths in 2013. Secondary transmission from person to person has been established, presenting a healthcare risk for health workers.In February 2014, a laboratory confirmed case of LF was reported in a health worker in Saki,Oyo state.

**Objectives:** We investigated the outbreak, identified and traced contacts with follow up and contained transmission

**Methods:** We conducted active case search/record review of hospitals records, contact risk stratification assessment, environmental assessment and laboratory analysis.

**Results:** Twenty contacts were classified as 13 probable and 7 suspected cases. For risk stratification we collected socio-demographic information, extent of contact, observed signs or symptoms suggestive of LF, and use or non - use of PPE; this revealed 77 contacts with 73 (95%) being health workers who were in contact with confirmed case during the acute phase of illness. Categorization of risk showed 18 (23.3%) were category 1(no risk), 54(70.1%) category 2 (low risk) and 5 (6.5%) category3 (high risk). The 25 samples collected were all negative for LF. None of the 97 identified contacts developed LF after a 21 day follow up period. Environmental assessment showed bush burning, poor food handling, poor refuse disposal and rodent consumption practices. Poor infection control practices were observed in 2 of the 4(50%)health facilities where the confirmed case received care with just one facility (25%) having an isolation ward.

**Conclusion:** Epidemiologic surveillance, contact tracing and risk assessment are important elements in an outbreak response. We trained health workers on good Infection control practices, prompt isolation and optimal LF case managemen. We conducted community based advocacy and sensitization.We recommend that active surveillance and public enlightenment should be continuous to effectively sustain LF control and prevent further outbreaks in this community and Oyo State

**Disclosure of Interest**

None Declared

### P141

#### Survival anlysis of the incidence of health-care associated respiratory syncytial virus in selected referral hospitals in Kenya, 2009-2011

Lilian Mayieka[1], Samuel Mwalili[2], Geofrey Arunga[3], Leonard Cosmas[4], Linus Ndegwa[2]

[1]Kenya Medical Research Institute; [2]Centers for Disease Control; [3]Ipas Africa Alliance; [4]World Health Organization, Nairobi, Kenya

**Correspondence:** Lilian Mayieka

*Antimicrobial Resistance and Infection Control* 2017, **6(Suppl 3):**P141

**Introduction:** In Sub Saharan Africa, there is severe overcrowding and frequent breakdown of essential services like water and hygiene in hospitals hence facilitating spread of infections.

**Objectives:** We sought to document the incidence of health-care associated RSV in the three referral hospitals and evaluate the factors that affect its infection using adjusted hazard ratio.

**Methods:** We retrospectively studied acquisition of RSV 72 hours post admission among patients admitted in three hospitals in Kenya: Kenyatta National-KNH, New Nyanza and Mbgathi District hospitals. Screening was done using RT-PCR on naso/oro pharyngeal samples collected from patients who developed influenza-like illness. Cox proportional hazard model was used for analyses.Hazard ratio associated with risk factors were given as the exponent of the cox model coefficient.

**Results:** Two-hundred and fifty-five samples were analyzed from patients admitted in the hospital. Forty (16%) of the samples were RSV positive, 25 (62.5%) of them being from male. Twenty-six (65.0%) of the patients were aged below <2 years, 8 between 2-4 years and 6 patients were ≥ 5 years. Majority (33, 82.5%) of the patients was from pediatric wards.KNH contributed the most number of patients (24,

60.0%). Cox regression analysis showed that males had 82% chance of contracting RSV faster (aHR = 4.5; CI = 1.7-11.7; P = 0.003). Admitted patients aged >5 years had 99% chance of contracting RSV faster than those <2 years (aHR = 81.7; CI = 4.8-139.5; P = 0.001). Patients admitted at Mbagathi and New Nyanza had 92%(aHR = 11.5; CI = 3.7-35.5;P < 0.001) and 94% (aHR = 17.02;CI = 5.9-49;P < 0.001) chance of contracting RSV faster than those in KNH respectively. Patients in the ICU had a 91% chance of contracting RSV faster than those admitted in the pediatric wards (aHR = 10.1; CI = 4.0-24.9; P = 0.028)

**Conclusion:** Being male, aged 5 years or more, admitted in the ICU increased the chance of contracting RSV faster. Therefore, target interventions should be aimed at patients in these wards.

**Disclosure of Interest**

None Declared

### P142

#### Prevalence of health care acquired infections among new mothers and newborns in the Kyrgyz Republic

Gulmira A. Djumalieva, Aleksey Kravcov, Nadira Soronbaeva
National Scientific and Practical Center for Infection Control, "Preventive Medicine" Scientific Association under the Ministry of Health of the Kyrgyz Republic, Bishkek, Kyrgyztan
**Correspondence:** Gulmira A. Djumalieva
*Antimicrobial Resistance and Infection Control* 2017, **6(Suppl 3):**P142

**Introduction:** This paper serves as the research report on prevalence of Health Care Acquired Infections (HCAI) among new mothers and newborns in the Kyrgyz Republic as an indicator of the effectiveness of infection control measures.

**Objectives:** Objective of the study is to investigate the prevalence of Health Care Acquired Infections (HCAI) among new mothers and newborns in the Kyrgyz Republic as an indicator of the effectiveness of infection control measures.

**Methods:** Two point prevalence surveys of the Health Care Acquired Infections among new mothers and newborns were conducted between 2013 and 2016. The point prevalence surveys were conducted in 10 public health hospitals. In total, 657 new mothers and 720 newborns were examined in the first study, and 552 new mothers and 623 newborns - in the second study. We used the method of active detection of infections using the CDC case standard definitions, and with the use of forms specifically designed for epidemiological surveillance. The studies were conducted by appropriately trained infection control specialists and epidemiologists.

In quantitative terms, point prevalence was defined as the ratio of the number of people who have an infection, at a particular point in time, to the total amount of people in the population, at risk, at the same point in time.

**Results:** Prevalence of infections among the new mothers was 6.8% in the first study, and 3.1% - in the 2nd study. Amongst the new mothers with the C-section, prevalence was 19.8% in the first study, and 8.5% - in the second study. Amongst the new mothers with natural delivery, prevalence was 3.6% at the first study, and 1.4% - in the second. Incidence of the HCAI among newborns was 8.9% in the first study, and 5.1% - in the second study. In the period between the two studies, infectious control measures were actively introduced through trainings and equipping of the clinical departments of the hospitals.

**Conclusion:** Introduction of the infection control measures, such as the hand hygiene, the safety standards for the medical procedures, and the appropriate use of antibiotics for the preventive purpose, enable the reduction of prevalence by a factor of 1.5-2. The point prevalence survey allows to determine the effectiveness of the measures undertaken, as well as to determine priority areas for interventions.

**Disclosure of Interest**

None Declared

### P143

#### Real time analysis of local 2016 point prevalence study data and investigation of risk factors

Padma Polubothu[1], Ann Kerr[2], Teresa Inkster[1]
[1]Microbiology, Queen Elizabeth University Hospital, Glasgow; [2]Infection control, NHS GGC, Glasgow, United Kingdom
**Correspondence:** Teresa Inkster
*Antimicrobial Resistance and Infection Control* 2017, **6(Suppl 3):**P143

**Introduction:** Our hospitals participated in the European HAI point prevalence survey (PPS) 2016. Real time analysis of data was undertaken by hospital and then collated for the health board.

**Objectives:** To describe findings from the 2016 PPS and investigate risk factors for the most common hospital acquired infections(HAIs).It was hoped that risk factor analysis would enable us to identify areas for quality improvement.

**Methods:** Ten hospitals were visited and 3834 patients were included in the survey. Data was collected between 1/9/16-18/11/16 by members of the infection control team trained in PPS methodology.

**Results:** Local analysis of the PPS data showed a decrease in the HAI rate within our hospitals from 4.7% in 2011 to 3.1% in 2016.The most common HAIs identified were pneumonia and urinary tract infections.Further analysis of patients with pneumonia revealed that only 17% had been intubated in the previous 48 hours. The majority of patients with pneumonia were > 6 5 years (74%). Almost half (48%) were considered end of life on McCabe score. Only 35% of urinary tract infections were associated with urinary catheters. 75% of female patients with UTI were postmenopausal and 58% of UTI patients had renal impairment.

**Conclusion:** Overall HAI rates in our hospitals have reduced since the 2011 PPS. Pneumonia and urinary tract infection represent the two most common HAIs. The low percentages of ventilator acquired pneumonias (VAP) and CAUTI within these categories are likely a result of quality improvement programmes and implementation of care bundles.For a significant proportion of patients pneumonia was a near end of life event.Patients with UTI were more likely to be postmenopausal if female and over half had coexisting renal impairment. Further analysis of risk factors is required to ascertain whether any quality improvement measures could be implemented to reduce HAI rates further.

**Disclosure of Interest**

None Declared

### P144

#### Infection control software: placebo or panacea?

Jie Li[1], QiuSha Meng[1], Melvin Liu[2], Evelyn Chua[2], Thean Yen Tan[1]
[1]Infection Control, Changi General Hospital; [2]IHIS, Singapore, Singapore
**Correspondence:** Thean Yen Tan
*Antimicrobial Resistance and Infection Control* 2017, **6(Suppl 3):**P144

**Introduction:** Manual monitoring of healthcare-associated infections and multi-drug-resistant organisms (MDROs) is inefficient and often patchy. Our hospital evaluated the impact of introducing an infection control management system (ICMS).

**Objectives:** The objectives were to assess the impact of ICMS on 4 parameters: time to detection, detection capability, outbreak detection and workflow impact.

**Methods:** Following a review, an ICMS system was selected based on a range of inbuilt system capabilities for alerting, recording and monitoring infections and target organisms, together with a data analytics system. Post- implementation, we assessed the impact of the ICMS on the operational and detection capabilities of infection control nurses (ICNs).

**Results:** Time to detection: With integration of patient movement and laboratory data, ICMS-enabled real-time alerting allowed immediate intervention compared to delays under the previous manual method (0.5-4 hour delay). ICNs were now able to identify up to 20 additional patients readmitted with conditions or MDROs of interest, previously missed under the old system.
Detection capability: Average daily notifications rose from 12 (pre-ICMS) to 20 alerts (post-ICMS). Epidemiologically important organisms (e.g. Influenza A virus, RSV) were tracked for the first time. The flexible rule-based ICMS system allowed application of "tags" to patients with specific conditions or with significant contact history, allowing immediate review and action if patients with these tags were re-admitted or transferred.
Outbreak detection: Contact tracing lists were efficiently generated by the ICMS by defining the patients at risk based on user-defined parameters.
Workflow improvement: Substantial time savings for ICN's were achieved for the following tasks: MRSA ward exit screening (43 hours saved/annum); contact tracing: (230-476 hours); reports generation: (130 hours), manual laboratory notification (104 hours).
Some specific data analytics and workflow processes were not available in ICMS, necessitating workflow changes/workarounds. ICMS implementation took significant time and effort from users. Finally, some gaps in ICMS capabilities were also identified.
**Conclusion:** ICMS improved efficiency and operational oversight of IC, which paradoxically increased the daily ICNs workload.
**Disclosure of Interest**
None Declared

### P145

#### Burden of healthcare-associated infections in China: a systematic review

Jiancong Wang[1], Fangfei Liu[2], Ermira Tartari[1], Walter Zingg[1]
[1]Infection Control Program and WHO Collaborating Centre on Patient Safety, University of Geneva Hospitals and Faculty of Medicine, Geneva, Switzerland; [2]Department of Nosocomial Infection Management, The Second Affiliated Hospital, Xi'an Jiaotong University School of Medicine, Xi'an, Shaanxi Province, China
**Correspondence:** Jiancong Wang
*Antimicrobial Resistance and Infection Control* 2017, **6(Suppl 3):**P145

**Introduction:** Despite regular regional and national point prevalence surveys (PPSs) in China, only a few have been reported in English peer-reviewed journals. Thus, there is no good estimation about the burden of healthcare-associated infections (HAIs) in Mainland China.
**Objectives:** This study aimed to assess the burden of HAIs in Mainland China, using data published both in English and Chinese.
**Methods:** We searched PubMed, the China National Knowledge Infrastructure, and Wan Fang for multicentre PPSs in acute care hospitals in Mainland China in the last ten years. The weighted prevalence was estimated by applying a random effects model.
**Results:** A total of 1626 publications were retrieved, of which 115 were eligible for final analysis. HAI prevalence [95% confidence interval (CI)] in general hospitals, children hospitals, hospitals for maternal and child health, and oncology hospitals were 3.02% (2.79-3.26), 4.43% (3.39-5.47), 1.88% (1.47-2.29), and 3.96% (3.12-4.79), respectively. HAI prevalence in general hospitals in the Eastern, Southern, Central, Northern and Western China regions were 3.21% (2.73-3.68), 3.37% (2.12-4.63), 3.51% (3.18-3.84), 2.61% (2.20-3.01), 2.83% (2.50-3.15), respectively. HAI point prevalence was highest in adult intensive care units (26.07%, 95%CI: 23.03-29.12), followed by surgery (3.26%, 95%CI: 2.96-3.57), internal medicine (3.06%, 95%CI: 2.67-3.46), and pediatrics (2.09%, 95%CI: 1.76-2.43). Lower respiratory tract infection was the most frequent HAI (48.18%), followed by urinary tract infection (11.64%), and surgical site infection (9.88%). *Pseudomonas aeruginosa* was the most frequently isolated microorganism (15.08%), followed by *Escherichia coli* (12.80%).
**Conclusion:** This is the first systematic review about the burden of HAI in Mainland China. The identified HAI prevalence was higher than in the Chinese national PPS report, but lower compared to Europe or the USA. However, without the low prevalence of the hospitals for maternal and child health, the overall HAI prevalence is close to the recent national US PPS. This systematic review serves as a benchmark for future PPS in Mainland China.
**Disclosure of Interest**
None Declared

### P146

#### The annual incidence of healthcare-associated infection in a tertiary hospital in Beijing, China

Yuzheng ZHANG[1], Janice M. Johnston[2], Qiang FU[3]
[1]School of Public Health, UNIVERSITY OF HONG KONG, Hong Kong, China; [2]School of Public Health, UNIVERSITY OF HONG KONG, Hong Kong, Hong Kong; [3]National Institute of Hospital Administration, Beijing, China
**Correspondence:** Yuzheng ZHANG
*Antimicrobial Resistance and Infection Control* 2017, **6(Suppl 3):**P146

**Introduction:** Hospital-wide and targeted surveillance is fundamental for healthcare-associated infection (HAI) prevention, in recent years, some automated computer-based systems were developed to improve the efficiency of the surveillance.
**Objectives:** Based on a real-time HAI surveillance system, we monitored all types of HAI to determine the annual incidence of HAI in a tertiary hospital in Beijing, China.
**Methods:** We defined HAI with the *Healthcare-associated Infection Diagnosis Criteria (2001)* of National Health and Family Planning Commission of China (NHFPC), infections occurred after admission 48 hours were included. The data from January 1st 2015 to December 31st 2015 was exported from the real-time surveillance system, which included demographic information, microbiological reports, and antibiotic usage, etc.
**Results:** Of 162,335 unique inpatient admissions, 3,934 had 1 or more HAI, the number of HAI cases was 4,922, the incidence rate was 3.03%. The most common type HAI were respiratory tract infections (2122, 43.11%), bloodstream infections (787, 15.99%), urinary tract infections (650, 13.21%), surgery site infections (604, 12.27%), gastrointestinal tract infections (333, 6.77%). 4916 bacteria were reported, of which gram-negative bacteria (2653, 53.97%), gram-positive bacteria (1230, 25.02%), fungi (1026, 20.87%) accounted the major proportion, and the top five pathogens were *Escherichia coli* (463, 9.42%), *Pseudomonas aeruginosa* (410, 8.34%), *Klebsiella pneumonia* (384, 7.81%), *Acinetobacter baumannii* (333, 6.77%), *Candida albicans* (291, 5.92%). 36.10% of the inpatients received one or more antibiotics.
**Conclusion:** Compared to the international studies, the HAI incidence of the hospital is similar to developed countries', however, the respiratory tract infection should be the priority for the HAI prevention in China.
**Disclosure of Interest**
None Declared

### P147

#### Situation of health care associated infections in Vietnam

Ha Thi Kim Phuong* [1], HV Thanh[1], VQ Hieu[2], T Ninh[1], S Otsu[2]
[1]Vietnam Authority of Medical Services; [2]World Health Organization, Hanoi, Viet Nam
**Correspondence:** Ha Thi Kim Phuong
*Antimicrobial Resistance and Infection Control* 2017, **6(Suppl 3):**P147

**Introduction:** Health care associated infections (HAIs) have a large burden on the quality of medical examination and treatment in health facilites. Recognizing the current incidence of HAIs and related factors will help managers develop effective measures to reduce the risk of HAIs. Selected surveillance of HAIs in hospitals has been conducted in Vietnam since the 1990s, although a standardized surveillance approach has not yet been implemented in all health care facilities nationally and evidence on the results of this surveillance remains limited. To support the development of a representative and systematic national HAI surveillance system, training was recently conducted for facilities and supported by international parteners.

**Objectives:** We assessed the current situation of HAIs based on the data in selected central and provincial hospitals in Vietnam.
**Methods:** We analysed HAI surveillance data from selected implementing hospitals in Vietnam from 2013-2016. We calculated the risk of HAIs overall and according to type of infection, setting in the hospital and risk factor.
**Results:** The risk of HAIs was 3.4% from 2013-2016, including respiratory infections which accounted for the highest estimates of 48.8%, blood infections at 13.9%, wound infections at 8.8%, urinary tract infections at 6.4%, and gastrointestinal infections at 4.5%. Settings with the highest risk of HAIs included intensive care units (ICUs) at 16.8%, surgical departments (including orthopedics, obstetrics and gynecology) at 6.1%, infectious disease department at 5.4% and pediatrics department at 3.4%. The following patient factors had a higher risk of HAIs: Patients >55 years, with long-term hospitalization, undergoing surgery, and with invasive procedures.
**Conclusion:** We demonstrated the risk of HAI present in selected hospitals in Vietnam implementing HAI surveillance although it may not be representative of the national situation. It is the first step in demonstrating the problem and highlighting the need for improved surveillance efforts moving forward.
**Disclosure of Interest**
None Declared

### P148
### Burden of nosocomial infections, antimicrobial use and antimicrobial drug resistance in bacteria isolates: an Indian study
S. Parimalakrishnan[1], Sundar VD[2], Anton Smith A[1], Sudhir Singh Gangwar[3], Yatindra kumar[3]
[1]Pharmacy, Annamalai University, Annamalai Nagar; [2]Pharmacy, GIET College of Pharmacy, Rajahmundry, [3]Pharmacy; GSVM Medical College, Kanpur, India
**Correspondence:** S. Parimalakrishnan
*Antimicrobial Resistance and Infection Control* 2017, **6(Suppl 3):**P148

**Introduction:** Hospital acquired infection (HAI) is a major public health problem globally. Increasing antibiotic resistance of microorganism with HAI leads potential problem during the treatment.
**Objectives:** The objectives of the present study are to assess HAI, antibiotic use pattern and determine antibacterial drug resistance in bacterial pathogens causing HAI.
**Methods:** Point prevalence survey method was followed, monthly repeated, systematically to assess HAI prevalence and antimicrobial use in patients. A cross sectional study was conducted among the postoperative patients between January and December 2016 and followed for progression of clinical signs and symptoms at surgical site and bacteremia upto discharge of the patient. Socio demographic characters were collected using structured questionnaire. Wound swab and venous blood samples were collected and analysed for bacteria isolates and antimicrobial susceptibility testing following standard bacteriological techniques.
**Results:** Among 1495 patients enrolled, the HAI prevalence was 32% (150 missing). 386 bacterial isolates were cultured from 500 HAIs, most frequently *Candida* species (33.5%), *Pseudomonas aeruginosa* (23.4%), *Escherichia coli* (20.7%), *Staphylococcus aureus* (12.1%) and *Klebsiella pneumoniae* (10.3%). Carbapenems resistant *P. aeruginosa* (46.1%), cefotaxime resistant *E. coli* (29.5%) and cefotaxime-resistant *K. pneumoniae* (24.4%) were observed among patients. Antimicrobials were prescribed for 100% patients, with 75% of patients receiving more than 2 antimirobials. The most common antimicrobial groups were carbapenems, third generation cephalosporins and fluoroquinolones (31.8%, 21.5% and 16.7% of total antimicrobials, respectively).
**Conclusion:** A high prevalence of HAIs was observed, mainly caused by fungal and both Gram-positive and negative bacteria with high carbapenem resistance rates. The study does not accounted the other causes involved in HAI like socio demographic characters. Even then the use of high rate of antimicrobial shows that need to improve appropriate use of antimicrobials and effective infection control programmes.
**Disclosure of Interest**
None Declared

### P149
### Point prevalence survey of health care associated infection and anti microbial therapy in the context of post ebola outbreak at ignace deen teaching hospital Conakry Guinea
Koulibaly Moussa, Houndjo Bienvenu, Stanislas Djomo Djiro, Fatoumata Koulibamy, Lansana Mady Camara, Mariame Beavogui, Alphonse Kpoghomou, Sophie Gardes
[1]Minister of Health, Conakry, Guinea
**Correspondence:** Koulibaly Moussa
*Antimicrobial Resistance and Infection Control* 2017, **6(Suppl 3):**P149

**Introduction:** Health care associated infections (HAI) are the most frequent adverse events in the health care delivery worldwide. Limited data are available from African countries. In addition there is no antimicrobial resistance program
**Objectives:** We aimed to estimate the prevalence of HAI and antimicrobial drug use at Ignace Deen Teaching hospital(IDTH) after the end of Ebola outbreak in the country
**Methods:** A point prevalence survey was conducted 14-15 July 2016 in the IDTH(473 bed) according to French National Survey Protocol (2012) using clinical or radiological criteria. Infection prevalence rate and proportion of infected patients and exposure to antimicrobial drug were assessed
**Results:** Among 299 hospitalised patients, HAI occurred in 57 (19,1%). The average time of acquisition of HAI was 7,8 days.The highest prevalence was observed in Intensive care unit (75%). The most common type of HAI was surgical site infection :SSI(16 cases :28% of all HAI) These SSI affected 19,2% patients from the department of obstetric/gynecology and 8,6% of patients from other surgical services.The remaining sites were :lower respiratory tract (12 cases :21% of HAI), urinary tract (8 cases 14%),skin (8cases :14%),blood stream (7 cases: 12,5%)
Urinary catheterization, peripheral intravenous catheterization and intubation were associated with the occurence of HAI (p <0,05)
79% of patients surveyed were treated with antibiotics and *Ceftriazone* was the most frequent drug used (27%)
**Conclusion:** The prevalence rate of HAI is very high at IDTH. These baseline results are the foundation for the SSI surveillance program underway. Measures for improvement in antibiotic prescription are implementing
**Disclosure of Interest**
None Declared

### P150
### A study to assess the trends and pattern of health care associated infection (HAI) in the Al –Qassim region of Kingdom of Saudi Arabia
Sanjay K. Gupta, Ibrahim S. Al Harby, Omar H. Romaih, Sinimol Jabar, Sunitha L. Mathias, on behalf of Infection Control Department
Infection control, AR RASS HOSPITAL, Ar Rass, Saudi Arabia
**Correspondence:** Sanjay K. Gupta
*Antimicrobial Resistance and Infection Control* 2017, **6(Suppl 3):**P150

**Abstract video clip: Introduction: Healthcare-Associated Infections (HAIs):** In USA up to 2 million healthcare-associated infections per year are reported, of which 80,000 are lethal. In Europe 5 million HAI cases per year is reported, of which 50,000 (1%) are lethal. **Objectives:** To find out the trends and pattern of health care associated infection (HAI). **Methodology:**In the present study data was analyzed from 2012 to 2016 about their trends of occurrence, most common organism, site of infection and major contributing area in the hospital. The collected data was analyzed using SPSS 20. **Observations:** Hospital wide rate of HAI range from 0.35-1.96 per thousand patients day.The trend of health care associated infection cases from 2012 to 2015 in increasing trend 60,68,72,77 respectively in 2016 till November only 51 cases were reported showing slight decreasing trend first time in last five years. Common organism observed in last five years Acenatobacter Baumannii 88 (27%) followed by Pseudomonas aeruginosa 73 (22%), E coli 37(11%), Klebsiella P 34(10%) and Candida 28(9%), the combined infection due to these entire five organism was 80%. Significant contributor of HAI cases in these five years in the present study was intensive care unit (ICU) 157 (48%) followed by medical wards (Male and female) 106 (32%), if we combined these two areas our hospital they are the most common contributor of HAI cases in the Hospital 263(80%). Common site was reported in the present study is catheter associated urinary tract infection was significantly higher (CA-UTI) 152(46%) followed by ventilator associated pneumonia and blood stream infection respectively (VAP) 66(20%) & 62 (19%). The common organisms reported in UTI is P.Aeruginosa (22%) followed by E-coli (19%) and Candida (14%), the most common microbial agent associated with VAP is Acinetobacter B 48 (72%) and found to be significantly higher, common microbial agents associated with BSI is Staphylococcus species(26%)followed by P.Aeruginosa (22.5%) and Acinetobacter B(21%).**Conclusion:** HAI in increasing trend, most common health care associated infection is CA-UTI, most common organism is A. Baumannii and major contributing area is ICU observed in the present study.

**Disclosure of Interest**

None Declared

### P152

#### Increasing incidence of candidemia and emerging role of candida parapsilosis in a large teaching hospital in Northern Italy: a 9-year retrospective study

Andrea Orsi[1,2], Federico Tassinari[1,2], Alessio Mesini[2,3], Antonella Talamini[1], Dorotea Bellina[1], Valerio Daturi[1], Chiara Paganino[2], Malgorzata Mikulska[2,3], Cristiano Alicino[1,2], Claudio Viscoli[2,3], Giancarlo A. Icardi[1,2]

[1]Hygiene Unit, I.R.C.C.S. University Hospital San Martino - IST National Institute for Cancer Research; [2]Department of Health Sciences, University of Genoa; [3]Infectious Diseases Unit, I.R.C.C.S. University Hospital San Martino - IST National Institute for Cancer Research, Genoa, Italy

**Correspondence:** Andrea Orsi

*Antimicrobial Resistance and Infection Control* 2017, **6(Suppl 3):**P152

**Introduction:** Candida species are currently one of the most common cause of bloodstream infections in hospitals, with increasing numbers of these healthcare associated infections occurring in high risk settings.

**Objectives:** The aims of the study were to investigate the epidemiology of candidaemia in a large teaching hospital in Northern Italy and to describe the etiologic fraction sustained by different candida species.

**Methods:** We performed a retrospective analysis in IRCCS AOU San Martino – IST, a 1,300-beds tertiary adult acute-care teaching hospital in Genoa, Italy. Numbers of the first episode of candidaemia and numbers of hospital patient-days were obtained for the period 1 January 2008 - 31 December 2016. Annual incidences of candidaemia by candida species were calculated as the number of events per 10,000 patient-days and per 1000 admissions.

**Results:** Between January 2008 and December 2016, we identified 1240 candidaemia, 600 of which were caused by Candida albicans (48.4%), 352 (28.4%) by Candida parapsilosis, 122 (9.8%) by Candida glabrata and 166 (13.4%) by other species of Candida. During the study period, incidence of Candida albicans bloodstream infection significantly increases from 1.09 to 2.28 episodes per 10,000 patient-days (p-value < 0.05). Interestingly a marked increase in the incidence of Candida parapsilosis bloodstream infection was registered between 2014 and 2016 when this specie became the most common cause of candidaemia. Incidences of candidemia sustained by Candida glabrata and other species of candida remained constant during the study period. The highest incidence of candidaemia was registered in intensive care units (17.06 episodes per 10,000 patient-days) followed by rehabilitation wards (3.47 episodes per 10,000 patient-days).

**Conclusion:** During the study period, we observed a marked increase in the incidence of candidaemia sustained by Candida albicans and parapsilosis in our hospital. More infection-control efforts are needed to contain this alarming diffusion.

**Disclosure of Interest**

None Declared

### P153

#### Evaluation of measles case-based surveillance system in Edo State, Nigeria, January to December, 2016

Ibrahim A. Seriki

Epidemiology, Nigeria Field Epidemiology & Laboratory Training Program, Abuja, Nigeria

*Antimicrobial Resistance and Infection Control* 2017, **6(Suppl 3):**P153

**Introduction:** Measles is one of the leading causes of death in children though it is vaccine-preventable. In 2015, over 134,200 deaths were recorded globally. Measles infection is endemic in Nigeria with outbreaks recorded all year round. In Africa, measles surveillance is active and it involves case reporting and feedback.

**Objectives:** The objectives of measles surveillance include predicting outbreaks by identifying geographic locations and age groups at risk and evaluating vaccination strategies. We described the surveillance system, assessed the system attributes and performance indicators.

**Methods:** We engaged and interviewed 20 stakeholders using CDC 2001 guidelines for evaluating public health surveillance system and Guidelines for Measles Surveillance and Outbreak response in Nigeria to assess the system's attributes and performance indicators. We obtained measles surveillance data from Edo State Ministry of Health. Data was analyzed using Epi Info 7.0

**Results:** Out of 142 suspected cases, seven (4.9%) were laboratory confirmed (Measles IgM+). Only one (5.5%) Local Government Areas (LGA) reported at least 2 cases a year (Target ≥ 80%). Annual rate of investigation of suspected measles case was 4.4/100,000(Target ≥2/100,000). Proportion of samples that arrived at laboratory within 48 hours was 8% (Target ≥ 80%). Proportion of test results received within a week was 62% (Target ≥ 80%). Proportion of samples arriving at laboratory in good condition was 72% (Target ≥ 90%).

**Conclusion:** The system was flexible; useful and representative. The surveillance system is not Conference its objectives of detecting and predicting outbreaks. Timeliness of sample arrival at the laboratory and feedback need to be improved

**Disclosure of Interest**

None Declared

### P154

#### A predictive model for healthcare-associated infections

David Fhager, Maria Marklund, Andreas Winroth, Anders Johansson

Infection control and prevention, Västerbotten County Council Sweden, Umeå, Sweden

**Correspondence:** Maria Marklund

*Antimicrobial Resistance and Infection Control* 2017, **6(Suppl 3):**P154

**Introduction:** Healthcare-Associated Infection (HCAI) is associated with increased mortality, longer hospital stay and increased costs.

**Objectives:** We analyzed mortality of patients with/without HCAI and created predictive models based on risk factors to identify patients with HCAI.

**Methods:** Data from 22 cross-sectional surveys of HCAI conducted 2008-2015 in Västerbotten County, Sweden were used. The survey protocol included HCAI (yes/no), several previously described risk factors for HCAI, and demographic data. Comorbidity and mortality data were collected. Predictive models using multiple logistic regression (MLR) or linear discriminant analysis (LDA) were created. Effect sizes were estimated by odds ratios (ORs). Kaplan-Meier curves were used for survival analyses.
**Results:** Data of 16 309 patients were analyzed. The prevalence of patients with at least one HCAI was 10.0%. By MLR, surgery during hospital stay (OR 2.82), urinary catheter (OR 2.78), central venous catheter (OR 2.74), mechanical ventilation (OR 2.07), immunosuppression (OR 1.90), male sex (OR 1.19) and increasing age (OR 1.01 per year) were verified as HCAI risk factors. The predictive models with predestined sensitivity set at 90% had a mean specificity of 31.1% (MLR) or 32.2% (LDA). The inclusion of comorbidity for a subset of patients increased the specificity with 8.5%. The 90-day survival rate was 80.8% for patients with and 87.8% for patients without HCAI (P = 0.0113).
**Conclusion:** The predictive models presented here can identify patients with HCAI having an increased risk of mortality, but at the cost of low specificity (predicting many false positive cases). Adding comorbidity as a model-variable may improve the prediction.
**Disclosure of Interest**
None Declared

### P155
### Use of EpiData®Analysis for point-prevalence surveillance-data for healthcare-assosiated infections
Ellen Brustad
Infection Control Department, Vestre Viken HT, Baerum, Drammen, Norway
*Antimicrobial Resistance and Infection Control* 2017, **6(Suppl 3):**P155

**Introduction:** Surveillance-data for healthcare-associated infections (HAI) must be communicated in a way that is easy to interpret to give leaders important decision support.
**Objectives:** We present a method for showing point prevalence surveillance (PPS) results over time by using the statistic process control (SPC) module in EpiData®Analysis. This tool shows whether a process is stable (normal variation) or unstable (special variation). An unstable process is defined as trends, shifts in level, or observations too far from mean.
**Methods:** We collected PPS-data for HAI quarterly from 2011 to 2016 in Vestre Viken, a Norwegian four hospital trust with 240.000 annual bed days. The registrations were national and the Norwegian Institute of Public Health picked four days annually for these registrations which were based upon criteria from ECDC. The following HAIs were included: urinary tract infection, pneumonia, surgical site infection (three categories) and bloodstream infection. Data collection was done in cooperation between infection control personnel and clinical staff. The results were plotted as a percent (denominator: number of in-patients 8 o'clock a.m., numerator: numbers of infections) versus time in Excel and exported to EpiData®Analysis where we selected a suitable chart. We chose p-chart which shows i) percent ii) mean, iii) upper and lower control limits (3 sigma) for observations. We used the same method for registration and quality assurance throughout the period.
**Results:** EpiData®Analysis elegantly demonstrated that the PPS results in Vestre Viken hospital trust shifted towards a lower mean in 2014. This shift in process was seen as eight successive data points below the mean, i.e. one of the rules for special variation. The new mean of 3.7% replaced the old one of 4.9%. Especially one of our hospitals (our biggest hospital, 350 beds) contributed to this improvement (data not shown) which was the result of long term systematic improvement work in Vestre Viken hospital trust.
**Conclusion:** The shift of the process towards a lower mean showed that PPS-results had improved. We found that EpiData®Analysis was a suitable tool for presenting PPS results. EpiData®Analysis is a free web-based statistic tool and it is easy to use.
**Disclosure of Interest**
None Declared

### P156
### Measuring dynamic social contacts in a rehabilitation hospital: effect of wards, patient and staff characteristics
Audrey Duval[1], Laura Temime[2], Lulla Opatowski[1], Didier Guillemot[3,4,5,6], Eric Fleury[7], Lucie Martinet[8], Pierre-Yves Boëlle[9], Thomas Obadia[10] on behalf of I-BIRD study group
[1]Biostatistics, Biomathematics, Pharmacoepidemiology and Infectious Diseases (B2PHI), Inserm, UVSQ, Institut Pasteur, Université Paris-Saclay; [2] Laboratoire MESuRS, Conservatoire National des Arts et Métiers, Paris; [3] AP-HP, Raymond-Poincaré Hospital, Garche; [4]Université de Versailles Saint-Quentin, UMR 1181, B2PHI, Montigny-Le-Bretonneux; [5] Institut Pasteur, B2PHI; [6] INSERM 1181 Biostatistics, Biomathematics, Pharmacoepidemiology and Infectious Diseases (B2PHI), Paris; [7]ENS de Lyon, Université de Lyon, Laboratoire de l'Informatique du Parallélisme (UMR CNRS 5668-ENS de Lyon-UCB Lyon 1), IXXI Rhône Alpes Complex Systems Institute; [8]ENS de Lyon, DANTE/INRIA, LIP UMR CNRS 5668 Université de Lyon, Lyon; [9]Sorbonne Universités, UPMC Univ Paris 06, UMR_S 1136, Institut Pierre Louis d'Epidémiologie et de Santé Publique; [10]Malaria: Parasites & Hosts Unit, Department of Parasites & Insect Vectors, Institut Pasteur; Bioinformatics and Biostatistics Hub, Institut Pasteur, Paris, France
**Correspondence:** Audrey Duval
*Antimicrobial Resistance and Infection Control* 2017, **6(Suppl 3):**P156

**Introduction:** Understanding transmission routes of hospital-acquired infections (HAI) is key to improve their control. In this context, understanding inter-individual contact patterns in hospitals is essential.
**Objectives:** To describe and analyze the frequency and duration of dynamic contact patterns in a long-term care facility.
**Methods:** In this study, we used wearable sensors to detect Close Proximity Interactions (CPIs) among patients and hospital staff in a 200-bed long-term care facility over 4 months. First, the dynamic CPI data was described in terms of contact frequency and duration per individual status or activity and per ward. Second, we investigated the individual factors associated with high contact frequency or duration using generalized linear mixed-effect models to analyse individual characteristics regardless of ward-effect.
**Results:** Hospital porters and physicians had the highest daily number of distinct contacts, making them more likely to transmit HAI among individuals. Conversely, contact duration was highest between patients, with potential implications in terms of HAI acquisition risk. Contact patterns differed among hospital wards, reflecting varying care patterns depending on reason for hospitalization, with more frequent contacts in neurologic wards and fewer, longer contacts in geriatric wards.
**Conclusion:** This study is the first to report proximity-sensing data informing on inter-individual contacts in long-term care settings. Our results should help better understand HAI spread, parameterize future mathematical models, and propose efficient control strategies in these settings.
**Disclosure of Interest**
None Declared

## Intensive care and dialysis

### P157
### Next-generation sequencing applied to healthcare-associated infection diagnosis
Nathalia D. C. Rodrigues[1], Nadine V. S. Stefanutto[1], Maria Thereza Lazzarini[2], José Carlos Bonjorno Junior[2], Euclides Matheucci Júnior[3]
[1]Research and Development, QGene - Healthcare Solutions and Logistics; [2]Irmandade of Santa Casa de Misericordia de São Carlos; [3] Biotechnology, Federal University of São Carlos, São Carlos, Brazil
**Correspondence:** Nathalia D. C. Rodrigues
*Antimicrobial Resistance and Infection Control* 2017, **6(Suppl 3):**P157

**Introduction:** Healthcare-associated infections in the intensive care unit (ICU) is a matter of serious concern. Identification and antimicrobial resistance pattern of infectious agents in the ICU are crucial for successful treatment, recovery and safety of patients. Next-generation sequencing (NGS) technology has recently emerged as a cost-effective and convenient approach for clinical microbiology and infection prevention.
**Objectives:** This work aims to evaluate the applicability of NGS for rapid identification and antimicrobial resistance pattern of pathogens directly from clinical samples.
**Methods:** Clinical samples (n = 12) from patients with suspected infections were examined using culture-based conventional microbiology tests and NGS sequencing directly from samples. Clinical samples (urine, blood and sputum) from patients in the ICU at Irmandade of Santa Casa de Misericordia de São Carlos are collected in sterile tubes and DNA was then isolated by using the ZymoBIOMICS DNA Miniprep Kit. DNA libraries were prepared using the Nextera XT DNA Sample Preparation kit and sequencing was performed on an Illumina MiSeq using pared-end runs with 2x150 bp read length. The data-analyses was performed with KmerFinder 2.0 method, MetaPhlAn and Kraken Metagenomics computational tools for species identification and ResFinder 2.1 for acquired antibiotic resistance genes identification.
**Results:** The NGS and conventional microbiology tests resulted in the same prevalent micro-organism species and antimicrobial resistance profile. The NGS sequencing identified additional bacterial species related to co-infection and bacteria originating from the natural human microbiota. Compared to conventional microbiology, where the time needed for identification and susceptibility testing would be 72 hours to weeks before this information is available to the clinician, NGS sequencing directly from clinical samples could yield results in less than 48 h.
**Conclusion:** With the use of NGS is possible to obtain a fast and reliable infection diagnosis, allowing rational and effective treatment decisions.
**Disclosure of Interest**
None Declared

### P158

#### Six year surveillance study of device –associated infections from a pediatric hospital in South India

Sureshkumar Dorairajan[1], K Velupandi[2], Nithin Bansel[1], Ram Gopalakrishnan[1]
[1]Infectious Disease; [2]Apollo Hospitals, Chennai, India
**Correspondence:** Sureshkumar Dorairajan
*Antimicrobial Resistance and Infection Control* 2017, **6(Suppl 3):**P158

**Introduction:** Device -associated hospital -acquired infections (DA-HAI) are a major problem in pediatric hospitals with principal threat to patient safety. DA-HAIs are the leading cause of morbidity & mortality among hospitalized children. However, limited data exist on the burden of DA- HAI in the pediatric hospitals from the developing world.
**Objectives:** The objective of the study was to determine the DA-HAI rates in a pediatric hospital in South India.
**Methods:** A prospective DA-HAI surveillance study was conducted in an 80 bed tertiary care referral pediatric hospital in Chennai over 6 year period between January 2011 and December 2016. We applied Centers for Disease Control and Prevention (CDC) and National Healthcare Safety Network (NHSN) definitions to determine the rates of Ventilator associated pneumonia (VAP), catheter-associated urinary tract infections (CA-UTI) and central line associated bloodstream infections (CLABSI).
**Results:** We documented 38757 device days (mechanical ventilator, urinary catheter & central line) during the study period. The VAP rate was 4.88/1000 mechanical ventilator days, the CA-UTI rate was 1.01 /1000 urinary catheter days and the CLABSI rate was 2.23/1000 central line days. The individual device days and DA-HAI numbers are shown in table 1 below
**Tablle-1 Device days and DA-HAIs numbers**

| Device | Device days | No of DA-HAIs |
|---|---|---|
| Mechanical Ventilator | 7982 | 39 |
| Urinary Catheter | 13,291 | 14 |
| Central line | 17,484 | 39 |

**Conclusion:** In this study VAP and CLABSI were common DA-HAIs when compared to CA-UTI. Nationwide surveillance studies are urgently required to confirm this finding and to create benchmark for pediatric DA-HAIs in India.

**References**
Horan TC, Andrus M, Dudeck MA. CDC/NHSN surveillancedefinition of health care-associated infection and criteriafor specific types of infections in the acute care setting.Am J Infect Control 2008;36(5):309—32.
Disclosure of Interest
None Declared

### P159

#### Base line structure assessment and estimation of device related infections IN INTENSIVE CARE UNIT AT A TEACHING HOSPITAL, SUDAN

Hiba M. Azrag
Directorate General of Quality, Federal Ministry of Health, khartoum, Sudan
*Antimicrobial Resistance and Infection Control* 2017, **6(Suppl 3):**P159

**Introduction:** Healthcare associated infection (HCAI) is the most frequent adverse event in health-care delivery worldwide leading to significant mortality and financial losses for health systems. The endemic burden of health care-associated infection is significantly higher in low- and middle-income than in high-income countries, in particular in patients admitted to intensive care units (ICU).
**Objectives:** This study was conducted to assess the structure and to estimate device related infections in an ICU of a Teaching Hospital in Sudan.
**Methods:** A cross sectional descriptive hospital based study conducted in the ICU for a period of one month.All patients admitted in the ICU with an inserted device were included in the the study. First a checklist was used to assess the strucrure of the ICU. Then an ICU surveillance form was used to collect the data about the patients.
**Results:** 1. **Assessment of the stucture revealed the following weaknesses**
**In term of governing document:** no policies and procedures
**In term of infrastructure :**
-No isolation room
-There was one sink
-The room was ventilated with split units and fan
**In term of staff :**
-Lack of IPC organization structure
-Doctors were not trained on IPC
**In term of supplies :**
-Plain bar soap was used for hand washing
-Hand drying materials were not available
- Alcohol based hand rub was not available
-Environmental cleaning / disinfectant agents were not available.
**The survey of device related infections revealed the following:**
- Overall Infection Rate in ICU/1000 bed days was **6.1** for every 1000 bed days.
- Urinary Tract Infection Rate/1000 Catheter days was **zero**
- Blood Stream Infection /1000 Central Catheter days was **zero**
- Ventilator Associated Pneumonia /1000 ventilator days was **83.3** for every 1000 ventilator days
**Conclusion:** Despite the lack of an effective infection prevention and control program, the detected rates of the device related infection were lower than expected. This result may be due to two reasons. The firt being health care provider practices that was not observed in this study and the second being the short duration of the study. Therefore we recommended to conduct an observation of healthcare providers practices and to establish a Surveillance system that would enable an ongoing data collection for more accurate results

**Disclosure of Interest**

None Declared

### P160

#### Epidemiology of healthcare-associated infections with multidrug-resistant bacteria in the intensive care unit

Hyo-Jin Lee[1], Yang Ree Kim[1], Hae Kyung Lee[2]
[1]Department of Internal Medicine; [2]Department of Laboratory Medicine, College of Medicine, The Catholic University of Korea, Seoul, Korea, Republic Of
**Correspondence:** Hyo-Jin Lee
*Antimicrobial Resistance and Infection Control* 2017, **6(Suppl 3):**P160

**Introduction:** Healthcare-associated infection remains a major cause of mortality and morbidity in critically ill patients.

**Objectives:** The objective of this study was to identify the epidemiology of healthcare-associated multidrug-resistant organisms (MDRO) infections in intensive care unit (ICU) patients.

**Methods:** We conducted a retrospective study of isolated microorganisms from the patients admitted to the ICU of Uijeongbu St. Mary's Hospital from 2014 January to 2016 June. Microorganisms isolated within 48 hours of admission were excluded.

**Results:** Among 46,019 patient-days, total 5669 positive cultures from 1376 ICU patients were obtained. In the ICU patient group, *Acinetobacter baumannii* (22.9%), *Staphylococcus aureus* (19.0%), *Klebsiella pneumoniae* (5.2%), *Pseudomonas aeruginosa* (5.2%), and *Enterococcus faecium* (5.1%) were the most commonly isolated pathogens. However, in the non-ICU patient group, *Staphylococcus aureus* (13.0%), *Acinetobacter baumannii* (8.8%), *Enterococcus faecium* (7.9%), *Pseudomonas aeruginosa* (7.3%), and *Escherichia coli* (7.1%) were isolated in order. The proportions of carbapenem-resistant *Acinetobacter baumannii* (CRAB; 91.5% vs. 72.0%, $p < 0.001$), methicillin-resistant *Staphylococcus aureus* (MRSA; 83.5% vs. 76.0%, $p < 0.001$) and carbapenem-resistant *Pseudomonas aeruginosa* (CRPA; 34.0% vs. 27.5%, $p = 0.039$), were higher in the ICU patient group than those of non-ICU patients. The proportions of extended-spectrum beta-lactamase producing *Enterobacteriaceae* (ESBL-E), and vancomycin-resistant *Enterococcus faecium* (VRE) were not significantly different. CRAB rate per 1,000 patients-days in ICU showed significant trends toward decreasing (OR = 0.95; 95% CI = 0.91-0.99; $p = 0.011$), However, there was no significant trend in MRSA, CRPA, ESBL-E and VRE rates.

**Conclusion:** ICU patients had high rates of CRAB, CRPA, and MRSA. No trend toward decreasing rates were observed for MDRO except CRAB. This study suggests the need for constant attentions to healthcare-associated MDRO infection control in ICU patients.

**Disclosure of Interest**

None Declared

### P161

#### Incidence of intensive care-acquired infections and resistant pathogens in long-term stayed patients

Emine Alp[1], Aysegul Ulu-Kilic[2], Dilek Altun[1], Kursat Gundogan[3], Aynur Akin[4], Mary-Louise McLaws[5]
[1]Infectious Diseases and Clinical Microbiology; [2]Erciyes University, Kayseri, Turkey; [3]Medical Intensive Care Unit; [4]Anesthesiology and Reanimation Intensive Care Unit, Erciyes University, Kayseri, Turkey; [5]Epidemiology Healthcare Infection and Infectious Diseases Control, UNSW Medicine, Epidemiology Healthcare Infection and Infectious Diseases Control, Sydney, Australia
**Correspondence:** Emine Alp
*Antimicrobial Resistance and Infection Control* 2017, **6(Suppl 3):**P161

**Introduction:** Long term stay increase ICU acquired infection with multidrug resistant pathogens, and also risk of cross transmission.In low-middle income countries, because of limited resources, all patients are cared in a same acute-care ICU that posses multiple risks.

**Objectives:** The aim of this study was to show the incidence of ICU-acquired infections and resistant pathogens in long-term stayed patients in an acute care ICU in a middle income country.

**Methods:** Prospective study enrolling all ICU patients, stayed more than 48 h, from January 2015 to December 2015.Patient demographics, comorbidities,Charlson comorbidity index, APACHE II score,expected mortality, previous stay in hospital in one year, stay in another hospital before ICU, infection and colonisation at admission, invasive devices, ICU-acquired infection and mortality were recorded.The study end point was ICU discharge.Patients were grouped as short-term (<8 days) and long term (≥8 days)patients.

**Results:** During the study period, 534 patients (median age 63 yr) were included;262 (49%) stayed <8 days and 272 (51%) stayed ≥8 days.One hundred seventy four (32,6%) patients had ICU-acquired infection.The incidence was 54% in long-term stayed patients, whereas it was 11% in short-term stayed patients (p = 0.001) and the median time for infection was 8.5 days. Infection and colonisation with multidrug resistant (MDR) pathogens were more common in long-term stayed patients.In multivariate analysis, patients on mechanical ventilation, with tracheostomy, central venous line, chest tube and steroid use are long-term stayed patients, whereas patients after operation and transferred from the hospital clinic are short-term patients.

**Conclusion:** In low-middle income countries, ICUs have infrastructure and workforce limitations that are closely related with ICU-acquired infections.Mechanical ventilated patients with multiple invasive devices should be cared separately from postoperative patients and patients transferred from the hospital clinic for intensive care support to prevent ICU-acquired infections and cross-transmission of MDR pathogens.

**Disclosure of Interest**

None Declared

### P162

#### Management of infections associated with neurocritical care: a six-year surveillance report

Gleb V. Danilov[1], Nataliya V. Kurdyumova[2], Olga N. Ershova[3], Michael A. Shifrin[4], Ivan A. Savin[2]
[1]Neurotrauma; [2]ICU; [3]Epidemiology; [4]IT, BURDENKO NEUROSURGERY INSTITUTE, Moscow, Russian Federation
**Correspondence:** Gleb V. Danilov
*Antimicrobial Resistance and Infection Control* 2017, **6(Suppl 3):**P162

**Introduction:** Hospital-acquired infections (HAI) control following neurosurgery is a dramatic challenge in neurointensive care unit (NICU).

**Objectives:** We report the incidence trends and sound risk factors for common HAI associated with neurointensive care following the introduction of prospective surveillance at our clinic.

**Methods:** A data-driven infection control approach was implemented at Burdenko Neurosurgery Center in 2010. The prospective HAI surveillance data on 2106 patients stayed at NICU for 48+ hours collected in 2011-2016 via electronic data capture were thoroughly analyzed to test the effectiveness of our infection control strategies in terms of HAI incidence decrease and antimicrobial resistance control.

**Results:** The overall incidence of nosocomial meningitis in the last 6 months of surveillance (2016) decreased significantly compared to the first 6 months (2011) from 17.0 to 8.0 per 100 surveyed cases [SC] (p = 0.01) with external ventricular drainage and cerebral spinal fluid leakage being the main risk factors (p < 0.05). Incidence of nosocomial pneumonia was also reduced from 38.2 to 22.2 per 100 SC in the same time frame (p = 0.001), while ventilator-associated pneumonia rate descended non-significantly (from 19.2 to 12.4 per 1,000 ventilator days, p = 0.2). Bloodstream infections decreased 2.4 times without reaching statistical significance (from 7.9 to 3.3 per 100 SC, p = 0.06). The average 6-month incidence density of catheter-associated bloodstream infections didn't alter significantly by the end of study period (from 4.1 to 2.7 per 1000 line days, p = 0.4). Urinary tract infections incidence changed non-significantly (from 27.3 to 23.6 per 100 SC, p = 0.5). Understanding epidemiological data snapshots and trends led us to design and deploy the site-specific bundles of care into clinical practice and dynamically optimize the antimicrobial stewardship at our Center.

**Conclusion:** Prospective surveillance and data-driven approach to infection control at NICU proved to be effective, leading to significant reduction of most common HAI in neurosurgery and evidence-based understanding of their risk factors.

**Disclosure of Interest**

None Declared

### P163

#### Results of a multiresistant organism (MRO) reduction initiative in Critical Care Medicine (CCM)

Robert P. Stevens[1,2,3], Joshua Philp[4], SImone Kelly[5], Bety Krstevska[5]
[1]Microbiology, South Eastern Area Laboratory Services; [2]Faculty of Medicine, The University of New South Wales; [3]Infectious Diseases; [4]Infection Prevention and Control, [5]Critical Care Medicine, The Sutherland Hospital, Sydney, Australia
**Correspondence:** Robert P. Stevens
*Antimicrobial Resistance and Infection Control* 2017, **6(Suppl 3):**P163

**Introduction:** In 2014 an outbreak of multiresistant *Pseudomonas aeruginosa* occurred in CCM at the Sutherland Hospital. This arose in the context of high Vancomycin Resistant Enterococci (VRE) acquisition rates. In response a multimodal strategy named "Say no to MRO" was initiated and outcomes measured.

**Objectives:** To reduce the transmission of MROs within CCM.

**Methods:** The "say no to MRO" strategy comprised a series of interventions over a study period of 28 months. The interventions were: Universal adoption of contact precautions including gloves and impervious gowns regardless of MRO status, increased hand hygiene education, enhanced environmental cleaning including Deprox®, mapping of MRO acquisition with targeted cleaning, limited patient bed movement, and increased antimicrobial stewardship activities. MRO acquisition rate per 1000 occupied bed days (OBD), length of stay (LOS) in CCM and hand hygiene compliance rates were the measured outcomes of the study.

**Results:** Individual interventions had incremental effect on outcome measures. An overall reduction in MRO acquisitions from 15.8/1000 OBD to 1.3/1000 OBD was observed over the study period. This resulted in a reduction in the average LOS by 25 hours correlating to a projected cost saving of approximately two million Australian Dollars per annum. Audited hand hygiene compliance rates increased by over ten percent during the study period.

**Conclusion:** This multimodal intervention was effective in reducing the transmission of MROs within CCM and had the additional benefit of reducing length of stay. This intervention resulted in a substantial projected cost saving.

**Disclosure of Interest**

None Declared

### P164

#### Implementation and validation of hemodialysis bundle at outpatient dialysis setting

Ahmad Ibrahim, El-Saed Aiman, Dagunton Nimfa dagunton, Bambilla Noelle Francesca, Kaleal Mohammed, Alahmari Ebtihaj, Hejaili Fayez, Balkhy Hanan
INFECTION PREVENTION & CONTROL, MNGHA, RIyadh, Saudi Arabia
**Correspondence:** Ahmad Ibrahim
*Antimicrobial Resistance and Infection Control* 2017, **6(Suppl 3):**P164

**Introduction:**

End stage Renal Disease (ESRD) patients are at a high risk for bacteremia as the process of hemodialysis requires frequent use of catheters and needles to access the bloodstream.CDC has recently introduced dialysis auditing checklist as part of preventive initiatives.

**Objectives:**

Hemodialysis bundle aims to prevent bloodstream infections (BSIs) in hemodialysis patients, and increase the use and visibility evidence based practices. The bundle can be used by facility staff themselves to help guide their practices.

**Methods:**

The following items of dialysis bundle have been monitored; Arteriovenous (AV) fistula/catheter connection and disconnection, exit site care, and dialysis station routine disinfection.The monitoring was done in out-patient dialysis in KAMC between April and June 2017 and was preceded by intensive education and training of the local staff regarding the bundle implementation. To assure the validation of the collected data, an Infection Control Practitioner (ICP) did observe the bundle components at the same patient. Non-compliance of one or more of the CDC checklist items was considered non-compliance of that component.

**Results:**

A total of 348 observations have been monitored by ICPs while using the HD bundle audit, the compliance with different components were as follows; 17% for catheter connection, 16% for catheter disconnection, 2% AV cannulation, 56% for AV de-cannulation, 91% for exit site care and 83% compliance for dialysis station routine disinfection. For the purpose of validation a total of 29 observations have been monitored by both ICPs and HD staff. The findings showed excellent bundle validation with 94% agreement between the validator and the dialysis staff.

**Conclusion:**

There was acceptable compliance for exit site care and dialysis station routine disinfection but limited or less than expected compliance for other components. Ongoing program is in place to better enforce preventive practices. Monitoring of HD bundle is possible at out-patient dialysis setting, as local staff are capable of collecting valid data. Further studies are required to assess the impact of such bundle on HD related infections and events.

- APIC - guide of elimination of infection at hemodialysis
- CDC - dialysis guidelines
- National Kidney Foundation's Kidney Disease Outcomes Quality Initiative (KDOQI) Vascular Access Guidelines

Disclosure of Interest

None Declared

### P165

#### An original approach of risk management to improve quality and safety of care in assisted self-care dialysis centres in a French Region

Agnès Gaudichon[1], Elise Seringe[1], Léa Hoisnard[1], Caroline Daniel[1], Estelle Yamani[2], Pascal Astagneau[1]
[1]CPias Ile-de-France; [2]ARS Ile-de-France, Paris, France
**Correspondence:** Agnès Gaudichon
*Antimicrobial Resistance and Infection Control* 2017, **6(Suppl 3):**P165

**Introduction:** Assisted self-care dialysis centres (ADC) are outpatient settings with potentially high-risk for healthcare associated infections. To improve the quality and safety of care in ADC of Paris area, we used an approach of risk management focused on infectious diseases risk.

**Objectives:** Highlight critical points which would need a common corrective action

**Methods:** All ADC in Paris area were invited to perform an infectious safety walkround, using a standardized method developed by the regional infection control reference center. The visit included interviews with patients and all staff present, observations of arteriovenous fistula cannulation, facility visit and documentary census. All data were collected on a standardized form and a qualitative rating was assigned for each setting.

**Results:** Overall, 37 of the 62 ADC performed the safety walkround (59.7%), of which 32 with data available for analysis. For 5/32 ADC, staff resources were considered as moderately critical because nurse legal ratio was respected but there was an overworked due to patient with many comorbidities. Patient education for hygiene was semi-critical in 22/32 ADC and for vascular access management in 6/34 ADC. The communication of the information was difficult due to a lack of written support or because of language barrier. For 11/32 ADC, the wearing of jewellery was moderately critical with rings often present. Hand hygiene was critical for 1/32 and semi-critical for 12/32 centers. Plain soap was still preferred to alcohol-based handrub. Flu vaccination was critical for 3/32 and semi-critical for 21/32 ADC. In spite of information campaign, health care workers were still refractory to the vaccination. Environmental cleaning was semi-critical by 14/32 ADC as not always performed between two sessions. Fistula antisepsis was critical for 4/32 and semi-critical for 8/32 ADC related with a lack of skin cleansing before applying antiseptic. Personal protective equipment was critical for 6/32 and moderately critical for 8/32 ADC especially at cannulation time.

**Conclusion:** Cross-transmission risk remains not fully controlled in these high-risk settings. Risk analysis methods should be promoted to improve empowerment of patients and awareness of healthcare staff.

**Disclosure of Interest**

None Declared

### National and sub-national infection prevention and control (IPC) programmes

#### P166

**Improving evidence for infection prevention policy and practice – the role of capacity building in health services research**

Lisa Hall, Emily Bailey, Nick Graves
QUEENSLAND UNIVERSITY OF TECHNOLOGY, Kelvin Grove, Brisbane, Australia
**Correspondence:** Lisa Hall
*Antimicrobial Resistance and Infection Control* 2017, **6(Suppl 3):**P166

**Introduction:** To continually improve infection prevention policy and practice we need to provide good quality, relevant evidence for decision makers. One strategy to improve the quality, utility and implementation of evidence is to build capacity for targeted, policy-driven health services research.

**Objectives:** To improve evidence for infection prevention policy and practice through establishment of a capacity building health services research centre.

**Methods:** Through a large, competitive, national capacity building grant we established the Centre of Research Excellence in Reducing Healthcare Associated Infection (CRE-RHAI) in Brisbane, Australia. Over 6 years we trained 10 PhD students, and 5 post docs in health services research, with projects relevant to infection prevention and control. Research topics were driven by gaps in the infection prevention literature and stakeholder priorities on topics such as antimicrobial stewardship, surveillance, environmental cleaning, decision-making, and cost-effectiveness of infection prevention strategies. A key focus was on the integrated use of diverse range of methods including epidemiology, health economics, qualitative research and mathematical modelling. A cohort model of student development was established, enabling peer-to-peer feedback and collaboration along with a structured set of activities to develop generic, transferable, research skills.

**Results:** The CRE-RHAI successfully generated a large amount of contemporary, useful evidence for infection prevention and control alongside training the next generation of health services researchers. A number of the projects are already influencing policy, and several have provided the groundwork for larger research studies. We propose that the combined output and impact of the CRE-RHAI far exceeds what would have resulted from these researchers working in isolation.

**Conclusion:** Focused investment of research funds by major funding agencies into targeted, capacity building schemes is a valuable use of resources. Aligning research capacity building centres with stakeholder priorities can facilitate the development of useful and relevant research, and aid translation of evidence into improved policy and practice.

**Disclosure of Interest**

None Declared

#### P167

**Ease of implementation assessment of infection prevention and control core components in low-resource settings**

Sara Tomczyk[1], Walter Zingg[2], Julie Storr[1], Claire Kilpatrick[1], Benedetta Allegranzi[1]
[1]World Health Organization; [2]University of Geneva Hospitals, Geneva, Switzerland, Geneva, Switzerland
**Correspondence:** Sara Tomczyk
*Antimicrobial Resistance and Infection Control* 2017, **6(Suppl 3):**P167

**Introduction:** Guidelines on the core components (CCs) for infection prevention and control (IPC) programmes were recently issued by the World Health Organization. Uptake of the guidelines in low-resource settings is critical but effective implementation may require adaptation and prioritization.

**Objectives:** We assessed predetermined ease of implementation criteria in low-resource settings for each CC at the national and facility level.

**Methods:** Based on existing models and expert consensus, criteria included financial and human resources; equipment, materials and infrastructure; capacity for communication and local adaptation; and interconnectedness with other priorities. Focus group discussions were held with international experts using a semi-structured questionnaire. The results were validated by a follow-up survey with individual participants. We summarized common implementation gaps (defined as average availability of criteria rating *less* than perceived need) and opportunities (defined as average availability of criteria rating *greater* than perceived need).

**Results:** Four focus groups were held and 26 (84%) experts responded to the survey. The first CC recommends that an IPC programme should be established at the national and facility level. Experts commonly reported that interconnectedness with other priorities, human resources, and communication capacity present large gaps in the implementation of this component, whereas capacity for local adaption and equipment availability present opportunities. Similar findings were reported for CCs on evidence-based guidelines, education and training, multimodal strategies, and monitoring, audit and feedback. Financial resources were reported as a large gap for the implementation of CCs on healthcare-associated infection surveillance and adequate workload, staffing and bed occupancy. Equipment, materials and infrastructure were reported as a large gap for the CC on built environment.

**Conclusion:** These findings suggest common patterns concerning the perceived ease of implementation of each IPC CC in low-resource settings. The results could be useful for implementers at the national and facility level when considering the necessary adaptation and prioritization of WHO IPC CCs.

**Disclosure of Interest**

None Declared

#### P168

**Implementation of infection prevention and control (IPC) in low-resource settings: a qualitative analysis**

Sara Tomczyk, Julie Storr, Claire Kilpatrick, Benedetta Allegranzi
World Health Organization, Geneva, Switzerland
**Correspondence:** Sara Tomczyk
*Antimicrobial Resistance and Infection Control* 2017, **6(Suppl 3):**P168

**Introduction:** Robust evidence on the implementation of infection prevention and control (IPC) interventions in low-resource settings is limited.

**Objectives:** We aimed to qualitatively identify examples of IPC implementation in these settings and summarize key learned lessons.

**Methods:** From December 2016–March 2017, we conducted semi-structured interviews with IPC professionals from low-resource settings and analysed the results using a qualitative inductive thematic approach. IPC implementation examples within the data items were coded, collated into broader themes, and reviewed to ensure validity. Themes were defined as patterned elements across data capturing key IPC implementation lessons; these were summarized descriptively.
**Results:** Twenty-nine interviews were analyzed. Common IPC implementation themes (appearing ≥4 times) were as follows: To develop an IPC programme, key elements include advocacy with managers, the driving role of national IPC associations, identification of champions, integration with antimicrobial resistance (AMR) and quality programmes, and legislation. To develop guidelines, key elements include securing initial technical assistance which can be followed by local adaptation, identification of common guideline areas with other programmes, and an early focus on guideline implementation (e.g. link to training, monitoring, other tools). For training, the development of an IPC career path and multidisciplinary approaches are important elements. To implement surveillance, stakeholders should carefully address case definitions, consider step-wise pilots, integrate activities with AMR efforts, and emphasize the use of data for improvement. To implement multimodal strategies, key elements include monitoring as well as raising awareness of the approach. For monitoring, it is critical to identify the feedback process early including presentation of data to leaders and linkage of results to incentives and improvement plans. Long-term advocacy is needed for staffing and bed occupancy standards and IPC professionals should be included in the facility construction and renovation process to promote improvement of the built environment.
**Conclusion:** Key themes of IPC implementation examples were identified. They offer important qualitative evidence for IPC professionals to consider.
**Disclosure of Interest**
None Declared

### P169
### Implementation of infection prevention and control program at national level in Brazil: a systematic approach
Magda M. Costa[1], Fabiana C. Sousa[1], Maria Dolores S. Nogueira[1], Cleide F. Ribeiro[1], Diogo P. Soares[1], Maria Clara Padoveze[2] on behalf of The National Committee for Prevention and Control of Healthcare-Associated Infection
[1]Anvisa- Agência Nacional de Vigilância Sanitária, Brasilia; [2]SCHOOL OF NURSING OF UNIVERSITY OF SÃO PAULO, São Paulo, Brazil
**Correspondence:** Maria Clara Padoveze
*Antimicrobial Resistance and Infection Control* 2017, **6(Suppl 3):**P169

**Introduction:** The implementation of an Infection Prevention and Control Programme (IPCP) at national level is challenging for developing countries. In Brazil, despite many progress, no written national program was developed up to 2012.
**Objectives:** To describe the implementation process of the national IPCP in Brazil.
**Methods:** A first evaluation was performed in 2012 by using the infection prevention and control core components assessment tools (IPCAT) according to the World Health Organization (WHO). Members of the National Committee for Prevention and Control of HAI (NCPC-HAI) performed an individual preliminary assessment of the conformity of IPCP at national level. Individual data were compiled to identify the level of agreement among NCPC-HAI members. A face-to-face meeting was carried out to achieve a consensus and to identify the main gaps to be addressed in the first IPCP (2013 to 2015). On January 2016 another cycle of IPCAT application and further development of national IPCP (2016 to 2020) was performed.
**Results:** The first national IPCP included detailed goals and strategic actions to achieve the following objectives: a) reduction of primary blood stream and surgical site infections; b) establish mechanisms of control of antimicrobial resistance in healthcare settings; d) improve the conformity of national IPCP. From 2012 to 2015 the conformity to the core components at national level raised from, respectively: organization of IPCP- 19% to 67%; technical guidelines- 23% to 50%; surveillance of HAI- 59% to 77%; microbiology laboratory support-7% to 43%; environment- 13% to 50%; monitoring and evaluation- 0 to 67%; and links with public health and other services- 0 to 5%; but no change in the human resources conformity (16%) in the studied period. The next IPCP was supported by these results and included periodic assessment to monitor the progress of the national IPCP.
**Conclusion:** The use of IPCAT favored a systematic assessment and implementation of national IPCP and allowed to address relevant gaps to promote improvements.
**Disclosure of Interest**
None Declared

### P170
### Performance of programs for prevention of healthcare-associated infection in small hospitals: a regional study in São Paulo, Brazil
Rubia A. L. Baroni, Pryscilla L. C. Santos, MARIA CLARA PADOVEZE
NURSING SCHOOL, UNIVERSITY OF SÃO PAULO, SÃO PAULO, Brazil
**Correspondence:** Rubia A. L. Baroni
*Antimicrobial Resistance and Infection Control* 2017, **6(Suppl 3):**P170

**Introduction:** The occurrences of Healthcare-Associated Infections(HAI) can be reduced by an effective Program of Prevention and Control(PPCHAI). However, small hospitals may lack resources to establish a full-compliant PPCHAI.
**Objectives:** To describe the structure for HAI prevention and to evaluate PPCHAIs of small hospitals in a coastal region of the state of São Paulo, Brazil.
**Methods:** Prospective cross-sectional study, performed between 2015 and 2016 in 14 small hospitals (52.0% of the eligible institutions). An evaluation tool previously validated was applied through direct observation, documental analysis and interviews. The Conformity Index(CI) of the PPCHAI included elements of both structure and process regarding four indicators: 1) Technical-Operational-TO; 2) Guidelines-GD; 3) Surveillance System-SS; 4) Prevention Activities-PA.
**Results:** The overall CI of PPCHAIs was 69.5% (DP = 12.3). This result was lower than other studies that included larger hospitals in others regions of Brazil. The mean values of each indicator were: TO = 61.0%(DP = 20.3); GD = 84.5%(DP = 18.7); SS = 57.9%(DP = 39.5); PA = 74.5%(DP = 24.1). Only three hospitals evaluated had an Intensive Care Unit. Availability of specific isolation rooms were found in four hospitals; only one with Negative Pressure and High Efficiency Particulate Air System. Hand hygiene resources and sharps disposal were available in all hospitals; however, in three hospitals the sharps disposal were inappropriate.
**Conclusion:** These small hospitals showed low CI regarding PPCHAI and insufficient resources to isolation precautions, which may affect the patient safety. These findings point out to the need of public policies addressed to this type of healthcare settings in the Brazilian healthcare system.
**Disclosure of Interest**
None Declared

### P171
### Performance of infections relating the medical care prevention and control program, in Manaus City, Amazonas State, Brazil
Rubia A. L. Baroni[1], HADELANDIA M. OLIVEIRA [2]
[1]NURSING SCHOOL, UNIVERSITY OF SÃO PAULO, SANTOS; [2]NURSING SCHOOL, UNIVERSIDADE FEDERAL DO AMAZONAS, SÃO PAULO, Brazil
**Correspondence:** Rubia A. L. Baroni
*Antimicrobial Resistance and Infection Control* 2017, **6(Suppl 3):**P171

**Abstract video clip: Performance of programs for prevention of healthcare-associated infection, in Manaus City, Amazonas State, Brazil.**
**Introduction:**Performance of programs for preven**tion of healthcare-associated infection**(PPCHAI) are not well recognized in North Region of Brazil. **Objective:** The scope was to evaluate these programs in hospitals of Manaus City, Brazilian North Region. **Method:**Assessment transversal study in 2015 with 25 hospitals, 89.3% of the totality, above 50 beds. Four indicators were applied for the data collection: Technical-Operational-TO; Guidelines-GD; Surveillance System-SS; Prevention Activities-PA. **Results:** The overall performance was 69%. This result was similar than other with small hospitals in a Brazilian coastal region. The mean values of each indicator were: TO = 81,9%; SS = 73,2%; GD = 72,3%; PA = 48,6%. There were associations between hospital characteristics and PPCHAI performance. The better evaluation was related to private, accredited/certified hospitals; PPCHAI comprised of nurses and physicians; nurse and physician labor entailment duly institutionalized; exclusive hour charge for the nurses(over 6 hours) and physicians (over 4 hous); the skill period of the nurses acting is as of five years and that of the physicians lies between five and ten years; control and prevention capacitation in the admission(excepting the SS). **Conclusion:**The study allowed recognize the weaknesses and opportunities of these programs at North Region, aimed to increasing the possibility of improvements in the quality of health services provided to that population. The application of these indicators is an important tool for the knowledge of IRMCPC throughout the Brazilian territory.
**Descriptors:** Health Assessment; Hospital Infection; Health Care Indicators

**Disclosure of Interest**
None Declared

### P172

#### Implementation of an infection prevention and control (ipc) programme in a rural province in Zimbabwe: the successes and challenges

Admire S. Murongazvombo, Wilfred Moyo, Epiphania Chaitezvi, Pepetua Munjoma
MoHCC, Mashonaland Central Province, Zimbabwe
**Correspondence:** Admire S. Murongazvombo
*Antimicrobial Resistance and Infection Control* 2017, **6(Suppl 3):**P172

**Introduction:** The Ministry of Health and Child Care (MoHCC) Zimbabwe has strengthened the National IPC programme through the five-year Zimbabwe Infection Prevention and Control Project(ZIPCOP).The programme operates in an environment with a high burden of infectious diseases including Human Immunodeficiency Virus(HIV) and Tuberculosis(TB).
**Objectives:** This study sought to assess the successes and challenges in the implementation of a MoHCC run IPC programme in a rural province in Zimbabwe.
**Methods:** A provincial team of National Certified IPC trainers conducted Site Support Visits(SSVs) to 14 health facilities in the province. The team used a colour-coded checklist to assess IPC from 2015 to 2016, followed by feedback at each site. IPC practices were scored as: 2.appropriate intervention in place; 1.intervention in progress; 0.intervention not in place; or N/A if the domain is not applicable. The final score was categorized into four classes, S1: ≥75%; S2:65-74%; S3:50-64%; S4: <50%.
**Results:** Seventy-nine percent of sites (11/14) showed progress from baseline with 54.5%(6/11) improving by two classes, 45.5%(5/11) improving to the next class, while 21%(3/14)maintained their S1 and S2 baseline classes resulting in six S1, six S2, two S3 sites. Strengths were consistently noted in the following domains: collection and submission of blood and body fluids exposure statistics(14/14,100%), having a trained IPC focal person(13/14, 92.9%), conducting IPC surveillance(12/14, 85.7%) and administrative TB controls in place(12/14,85.6%). Weaknesses were noted in TB healthcare-worker (HW) screening(6/14, 42.9%), having personal protective equipment policy(6/14, 42.9%) and functional incinerators(7/14, 50%).
**Conclusion:** Site support and mentoring by the MoHCC Provincial IPC team, building on gains made by ZIPCOP, has resulted in a marked improvement in IPC practices in the sites in Mashonaland Central Province, Zimbabwe. This approach has yielded success in low-cost interventions such as administrative TB infection controls and IPC surveillance. The domains that posed a challenge include TB HW screening and incinerators. The study demonstrated the value of SSVs and the colour-coded checklist in improving IPC programmes.

**Disclosure of Interest**
None Declared

### P173

#### Past, present and future: the evolution of the infection prevention and control programme in Georgia

David Tsereteli[1], Giorgi Chakhunashvili[1], Ekaterine Jabidze[1], Ana Paula Coutinho Rehse[2]
[1]Communicable Disease, National Center for Disease Control and Public Health, Tbilisi, Georgia; [2]Health Emergencies and Communicable Diseases, WHO Regional Office for Europe, Copenhagen, Denmark
**Correspondence:** David Tsereteli
*Antimicrobial Resistance and Infection Control* 2017, **6(Suppl 3):**P173

**Introduction:** For many years the infection prevention and control (IPC) programme in Georgia was based on punitive administrative measures.
**Objectives:** Assessment of evaluation IPC programme in Georgia.
**Methods:** Descriptive study about the improvements of IPC programme in Georgia overtime.
**Results:** From the Soviet period and up to the 20th century, spite of case definitions of healthcare associated infections (HCAIs) were available and reporting of HCAIs was considered mandatory, only passive surveillance of HCAIs was conducted in hospitals. For decades the national prevalence of HCAIs was never higher than 1%, and data describing antimicrobial resistance (AMR) rates in hospitals was unavailable. Later in 2005, the Georgian National Medical Center in collaboration with Minnesota Department of Health and University of Minnesota, USA, implemented the first successful IPC programme. The National IPC guideline was then developed and published for the first time in 2009. During the past few years, a number of projects aiming to further improve and strength Georgia national IPC and AMR capacities were implemented in collaboration with WHO and other international partners. Among those projects, a national strategy to combat AMR was approved, the national AMR committee was created and empowered, legislation on IPC renewed, an IPC post-graduation training curricula developed, awareness on HCAIs and AMR increased and knowledge on IPC among healthcare workers improved. Georgia joined the Central Asian and Eastern European Surveillance of AMR network, established the National Microbiology laboratory network, EUCAST standards were implemented in laboratories, and more recently the pilot proof-of-principle study was carried out. The following activities are planned for next years: strengthening IPC programmes in hospitals, surveillance of the antibiotic use, promotion of antibiotic stewardship, update National IPC guideline and development of IPC standard operation procedures.
**Conclusion:** Mandatory notification of HCAIs is not an indicator of effective IPC programme. Political commitment, international support and availability of technical expertise at national level were crucial to instigate a fundamental change in the Georgian IPC programme.

**Disclosure of Interest**
None Declared

### P174

#### Accelerating implementation of the ipc policy in Kenya

RACHEL KAMAU
Health Standards Quality Assurance and Regulations, Ministry of Health, Nairobi, Kenya
**Correspondence:** RACHEL KAMAU
*Antimicrobial Resistance and Infection Control* 2017, **6(Suppl 3):**P174

**Abstract video clip: ACCELERATING IMPLEMENTATION OF THE IPC POLICY IN KENYA**
**Introduction**

The importance of maintaining high standards of infection prevention and control (IPC) practices in health care facilities is a matter of national concern for the Ministry of Health (MoH) in Kenya. In 2010, the MoH developed its first IPC Policy that recommended the establishment of a cohesive, comprehensive IPC program in the Department of Health Standards **Quality Assurance and Regulations. In 2013 an IPC unit was established within the Ministry.**

**Method**

This paper describes the achievements of the IPC program since 2013

**Achievements**

Following the establishment of the IPC unit, the National IPC Advisory Committee was appointed and its first task was to conduct a rapid situation assessment of IPC practices in Kenya to support the development of the national IPC strategic plan (2014-2018). The recommendations from the situation assessment to revise the national IPC policy and to update the national IPC guidelines was done in 2015. IPC training modules were also developed and are being used for training healthcare workers. An antimicrobial resistance (AMR) focal point was appointed to support the implementation of AMR surveillance activities in the IPC strategic plan. Also, the National Antimicrobial Stewardship Committee was appointed. The committee has supported the revision of the AMR situation analysis, the development of the National Policy, National Action Plan and the Surveillance Strategy for AMR. Counties have been supported to develop IPC structures and work-plans. However, training for most hospital IPC committees and healthcare workers have not been done due to inadequate resources. However, resources for IPC/AMR, have been increasing over the last 4 years. Currently 15 counties and 10 hospitals have trained TOTs on infection prevention and control.

**Conclusion**

Although a considerable number of IPC program achievements have been made mainly in the development of policies, guidelines, strategic plans as well as the establishment of IPC structures, there is need to strengthen them through capacity building at all levels. The IPC program should further take advantage of the programs dealing with quality of health care services, to facilitate change of IPC practices at health facilities.

**Disclosure of Interest**

None Declared

### P175

#### Using data to enhance implementation in a low resource setting - Liberia expereince

Catherine T. Cooper
Department of Health Services, Ministry of Health, Liberia, monrovia, Liberia
*Antimicrobial Resistance and Infection Control* 2017, **6(Suppl 3):**P175

**Introduction**

The Ebola virus outbreak that affected the West African sub-region, highlighted weaknesses in the health systems of all three affected countries including Liberia. It particularly revealed a gap in the area of infection prevention and control (IPC) and in quality health service delivery in the health sector. By the end of the outbreak in 2015, there were 10, 885 suspected, probably, and confirmed EVD cases. There were 4,841 cumulative deaths. There were 378 confirmed cases among health workers and 192 deaths. The Ministry of Health in an effort to address the gaps highlighted during the outbreak, introduced several IPC strategies and approaches at all levels of the health system. A Quality Management Unit was established with oversight for IPC at the end of the outbreak. The strategies addressed IPC training, supervision, supply chain, risk assessment of exposed health worker, mentoring and infrastructural improvement.

**Objectives**

The objective of the abstract is:

- To show the use of data to track performance of IPC practices in health facilities.

**Methods**

Through a regional approach, the ministry worked with WHO to develop a set of indicators for IPC and WASH that focuses on IPC practices. The target population included 770 health facilities in the public and private sector. The monitoring tool used had eleven (11) indicators. The process of monitoring the indicators begun in July 2016 and analysis of the indicators were done from September to December 2016.

**Results**

The results over the period showed a national average IPC compliance of 41%. The best performing indicators were that of a dedicated IPC/WASH person (71%) and water supply availability (70%). The indicators performing poorly were that of water storage – safe use of water tanks (2%), occupational health – health worker exposure (4%) and in-service training (12%).

**Conclusion:** The ministry however believes that keeping a visible focus on the IPC practices through a monitoring mechanism will ensure that IPC continues to be a priority in the health system. In a low resource setting a system of tracking progress can assist the health sector in setting its priorities and aligning its resources to those defined priorities. This monitoring system has contributed significantly to ensuring that patient safety and quality improvement continues to be a priority in the Liberia health system.

**Disclosure of Interest**

None Declared

### P176

#### Problem of prevention of healthcare-associated infections (HAI) in Russia. System of state support for its study and solution

Anna Popova[1], Elena Yezhlova[1], Elena Igonina[1], Vasily Akimkin [2], Nikolay Shestopalov[2]
[1]Federal Service on Surveillance for Consumer Rights Protection and Human Well-being; [2]Federal Budget Scientific Institution "Scientific Research Disinfectology Institute" of Federal Service for Surveillance on Consumer Rights Protection and Human Well-being, Moscow, Russian Federation
**Correspondence:** Vasily Akimkin
*Antimicrobial Resistance and Infection Control* 2017, **6(Suppl 3):**P176

**Introduction:** In modern conditions, prevention of healthcare-associated infections (HAI) is one of the global problems. In the Russian Federation, according to official statistics, about 25-30 thousand cases of HAIs (less than 0.1% of hospitalized patients number) are recorded each year, which does not reflect a real epidemiological situation with HAI, their social and economic consequences. According to research results, HAIs affect on average about 10% of patients amounting to at least 2.5-3 million cases annually. The total annual economic damage caused by HAI in Russia could reach 300 billion rubles.

**Objectives:** The problem of HAI is closely associated with the formation and spread of hospital strains of HAI pathogens with total resistance to antibiotics and disinfectants, which negatively affect a quality of patient care and effectiveness of preventive measures. A main objective of the presented study is to evaluate the problem on a government level and to propose its solution.

**Methods:** Taking into account the extremely high social and economic importance of the problem, the Government of the Russian Federation decided to conduct a large-scale 5-year project to study a real state of issues of registration and prevention of HAI in Russian hospitals. It is planned to involve 7 large regions of the Russian Federation, about 30 large medical institutions, 10 specialized scientific research institutes, and more than 50 well-known medical experts.

**Results:** The results of the project implementation include ensuring a full detection and registration of HAI cases in medical organizations; creation of standard protocols for the diagnosis and treatment of HAI based on monitoring data on the species composition of pathogens, their resistance to antibiotics and disinfectants; introduction of modern innovative epidemiologically effective methods and technologies for HAI prevention in public health care; ensuring compliance with the rules of hand hygiene by medical personnel; regulation of the market for the consumption of antibiotics, disinfectants and antiseptics.

**Conclusion:** The project implementation leads to better understanding of current HAI situation and to reduction of socio-economic damage level from HAI in Russian Federation.

**Disclosure of Interest**
None Declared

### P177

#### Setting up of a national action plan for prevention and control of healthcare-associated infections in Benin

Ahoyo A. Théodora[1], Alain K. Aissi[1], Louis Affovehounde[1], Denis Sossa[1], Eléonore A. Gandjeto[1], Clément Glèlè Kakaï[1], Chabi Bagou Orou Yorou[1], André Lukusa[2], Boniface Hakizimana[3], Dorothée GAZARD [4]
[1]Direction Nationale la Santé Publique, Ministère de la Santé; [2]OMS - Bénin; [3]OMS; [4]Ministère de la Santé, Cotonou, Benin
**Correspondence:** Alain K. Aissi
*Antimicrobial Resistance and Infection Control* 2017, **6(Suppl 3):**P177

**Introduction:** The socio-economic impact of the international health crisis linked to Ebola virus disease (EVD) outbreak between 2014 and 2016 justified the urgent need for the establishment of national programs for Infection Prevention and Control (IPC) in African countries.
**Objectives:** Analyze the relevance and the conditions of success of the implementation of the IPC national action plan adopted for Benin in July 2016.
**Methods:** Semi-guided interviews with the key contributors to the IPC national plan development, technical and financial partners and other relevant authorities from health system. Observation in 22 hospitals and review of activities reports of the Unit for Patient Safety and risk management at Ministry of Health between March 2016 and February 2017.
**Results:** Three strategic axis including 08 components adopted by consensus at a national workshop with 24 priority actions split into 95 activities. Selection, planning and budgeting of activities based on evaluation data of strengths, weaknesses, opportunities and threats of the health system. Taken into account of recommendations resulting from the missions of different IPC expert groups sent by WHO and CDC in the context of preparing for the response to possible EVD outbreak. Involvement of public and private sector healthcare professionals, patient associations and consumer advocacy groups. Revitalization of the Committees of fighting against Nosocomial infections and installation in 13 pilot hospitals, of an IPC operational team with clearly goals. Renewal of the government's commitment to reforming the health system by taking as high priority the improving of quality of care and patient safety.
**Conclusion:** Implementation of Benin IPC national action plan needs to be supported in its three axis : Improvement of standard precautions observance in all care situations; organization of HAI surveillance and fighting against antimicrobial resistance ; health worker Protection and community involvement.
**Disclosure of Interest**
None Declared

### P178

#### Impact of the international conferences on prevention and infection control in the promotion of patient safety in Benin Republic

Alain K. Aissi, Ahoyo A. Théodora, Louis Affovehounde, Denis Sossa, Eléonore A. Gandjeto, Chabi Bagou OROU YOROU
Direction Nationale la Santé Publique, MINISTÈRE DE LA SANTÉ, Cotonou, Benin
**Correspondence:** Alain K. Aissi
*Antimicrobial Resistance and Infection Control* 2017, **6(Suppl 3):**P178

**Introduction:** The three last editions of the International Conference on Prevention and Infection Control (ICPIC) have each been attended by some representatives of Benin.
**Objectives:** Analyze the effect of ICPIC on improving the health policy in Benin specifically in the field of patient safety.
**Methods:** Retrospective study based on documentary review and semi-directed interviews of the actors who participated in at least one edition of ICPIC or who have knowing the existence of ICPIC at the Ministry of Health. Data were collected from January to February 2017 and analyzed manually.
**Results:** At ICPIC 2011, three officials of Health Ministry and one university teacher presented papers. Their participation favored the choice of Benin as organization country of the 1st International Conference of Health Ministers of Africa on Patient Safety (CIMSEF 2012) thanks to the support from RIPAQS and HUG. This Conference led to increase awareness of many Health system decision-maker. In 2013, ICPIC was attended by ten representatives from Benin. Those latter, upon their return, called for the adoption of a national patient safety policy whose conception was entrusted to an unit created by ministerial decree. The actions of this unit focused on promotion of standard precautions, especially the observance of hand hygiene in health care establishments. Following ICPIC 2015, in a context where Ebola and Lassa haemorrhagic fever outbreaks continued to threaten the country, participants representing Benin strengthened their advocacy until obtaining the support from WHO to develop a National Actions Plan for Prevention and Control of healthcare-associated infections. This plan contains a special component for the health workers protection following the healthwise approach and the patient's involvement in the fight against infections.
**Conclusion:** The Ministry of Health needs to be supported to continue the implementation of IPC actions in hospitals. ICPIC is a source of opportunities for the improvement of Patient Safety for Benin and other African countries.
**Disclosure of Interest**
None Declared

### P179

#### Strengthening national IPC programs: an urgent need for Central African Countries

Pierre C. Kariyo
HSS, WHO, LIBREVILLE, Gabon
*Antimicrobial Resistance and Infection Control* 2017, **6(Suppl 3):**P179

**Introduction:** Infections that originate within health care facilities have always presented a major problem in delivering health care. Ebola outbreak in West Africa has shown that weaknesses in IPC practices and programs have led to the spread of the Ebola virus in healthcare workers and the community. In order to understand how IPC measures are implemented to prevent Healthcare Associated Infections a survey was conducted in 10 countries
**Objectives:** The aim of the survey was to try to understand how IPC programs are designed and implemented in the national health systems.
**Methods:** A survey was conducted using an ad hoc questionnaire that was administered to officials in the Ministry of health and collected by WHO country offices colleagues. The Data collected was qualitatively analyzed and calculations manually done
**Results:** Nine of 10 countries (90%) contacted completed the survey form. 8 of the 9countries (88.8%) have neither national IPC Policy and guidelines nor IPC program and therefore provide no supervision and monitoring of professional practices in the country's health services. 2 countries out of 9 (22.2%) that have established a national IPC program and validated their national guidelines don't track their implementation in health facilities. These countries were Chad and Congo. The reasons given for this were the lack of human and material resources allocated to the programs. Surveillance of Healthcare associated Infections (HAI) is nonexistent in the 10 (100%) countries and is not perceived as a national public health priority despite the pledge to tackle HAI's signed by most of these countries in 2008. However, like many of African countries, in the context of Ebola outbreak, all the 10 countries (100%) undertook, with the WHO support, activities related to preparedness and response to the detection and management of Ebola cases at least in the national referral hospital. Most countries have curricula that provide training professional on IPC components but there is generally no mechanism to monitor the professional practices regarding IPC at facility level.
**Conclusion:** National IPC programs need to be implemented in most African countries in order to ensure better quality of healthcare and improve patient safety. High level advocacy is still needed to create a national mechanism or program to oversee IPC activities in the country.
**Disclosure of Interest**
None Declared

### Water sanitation and quality

#### P180
**Water, sanitation & hygiene training for clinicians & cleaners in Cambodian Hospitals**
Lindsay Denny[1], Joanne McGriff[1], Khankryka Lim[2]
[1]Center for Global Safe WASH, Emory University, Atlanta, United States; [2]Center for Global Safe WASH, Emory University, Phnom Penh, Cambodia
**Correspondence:** Lindsay Denny
*Antimicrobial Resistance and Infection Control* 2017, **6(Suppl 3):**P180

**Introduction:** Safe water, sanitation, and hygiene (WASH) are fundemental to infection prevention and control (IPC) and thus to the provision of quality healthcare. However, staff at healthcare facilities (HCF) in low-income settings often are inadequately trained on WASH.
**Objectives:** To address gaps in WASH knowledge and practices, the Center for Global Safe WASH at Emory University developed, conducted, and evaluated a training for clinicians and cleaners in Cambodia.
**Methods:** The training occurred in 10 hospitals and focused on four topics where WASH and IPC intersect: hand hygiene, equipment processing, environmental cleaning and waste management. A needs assessment, including observation and survey, was conducted prior to curriculum development. During the training, participants completed a pre- and post-training assessment. An evaluation was administered three months after the training, including survey, observation, and demonstrations. The evaluation is currently being repeated six months post-training.
**Results:** The needs assessment included 250 clinicians and 40 cleaners. Clinicians' knowledge of waste management was highest (90%), while equipment processing was lowest (68%). Certain attitudes drew particular concern; for example, 25% of cleaners believed it was unnecessary for delivery rooms to be cleaned between patients. Hand hygiene compliance was poor (36%). Sixty-six percent of clinicians and 86% of cleaners reported never receiving WASH or IPC trained. An expert panel concluded that staff had insufficient knowledge and practice on all topics and recommended a comprehensive WASH training. Separate trainings were held for doctors, nurses and midwives, and cleaners. From pre- to post-training assessments, correct responses increased by an average of 25% for doctors, 22% for nurses and midwives, and 17% for cleaners. At the three-month evaluation, hospitals scored an average of 71% and hygiene compliance increased to 51%.
**Conclusion:** In Cambodia, an alarming number of clinicians have received suboptimal IPC training, while cleaners are often overlooked altogether. By addressing gaps, WASH training has made strides in raising awareness of IPC which has translated into improved hygiene practices.
**Disclosure of Interest**
None Declared

#### P181
**Water, sanitation, hygiene and infection prevention and control conditions in 52 healthcare facilities in Hoima District of Uganda**
Habib Yakubu[1], Richard K. Mugambe[2], Joanne McGriff[1], Christine L. Moe[1], Constance Bwire[3], Emmanuel Opoki[4], Fred Owera-Odom[3], John Bosco Isunju[2], James Michiel[1], Lindsay Denny[1]
[1]Hubert Department of Global Health, Center for Global Safe WASH, Emory University, Atlanta, United States; [2]Disease Control and Enviromental Health, Makerere University School of Public Health; [3]CARE International Uganda, [4]World Vision Uganda, Kampala, Uganda
**Correspondence:** Habib Yakubu
*Antimicrobial Resistance and Infection Control* 2017, **6(Suppl 3):**P181

**Introduction:** There is a dearth of knowledge of water, sanitation and hygiene (WASH) and infection prevention and control (IPC) conditions in developing countries. Poor (WASH) and IPC conditions are associated with healthcare associated infections that disproportionately burden neonates and their mothers. A 2015 WHO/UNICEF report states that about 38% of healthcare facilities (HCF) in lower and middle income countries do not have safe supply of water and 19% lack adequate toilets.
**Objectives:** The aim of the study was to conduct an assessment of (WASH) and (IPC) conditions in 52 healthcare facilities in Hoima District of Uganda to identify priority areas for improvement, guide plans for interventions and advocate for action
**Methods:** In November 2016, the WASH in HCF tool was deployed in 52 healthcare facilities in 10 sub counties of Hoima District. The WASH in HCF conditions tool consist of a) Interviews with HCF directors and administrative staff b) Observation of key wards c) water quality sampling and analysis of maternity, outpatient or inpatient wards. The data was collected on a mobile device and analyzed in SAS. A score card for each facility was produced based on whether the facility provides a basic, improved or unimproved or no service.
**Results:** Overall, only about 15.4% of the HCF met basic standards for managing waste, about 7.7% met the basic standards for sanitation, 9.6% had basic water supply systems and 11.5% had handwashing with soap and water facilities at the time of visit. In addition, 28.8% carried out cleaning routines.
Approximately 69% and 4% of the sampled water met the Ugandan national standards for drinking water for *Escherichia coli* and free chlorine respectively.
**Conclusion:** This assessment is a first of such survey carried out in Hoima District. The study shows that most of the (HCF) in Hoima District do not provide basic WASH services that has implications for infection prevention and control and healthcare associated infections. The results from this research is being used to inform interventions and advocate for action.
**Disclosure of Interest**
None Declared

#### P182
**Are hydrotherapy pools a risk to acquire antibiotic-resistant bacteria?**
Christiane Höller* [1], Daniela Köck[1], Manfred Köster[2], Giuseppe Valenza[2], Stefanie Huber[1]
[1]Hygiene, Bavarian Health and Food Safety Authority, Oberschleißheim; [2]Hygiene, Bavarian Health and Food Safety Authority, Erlangen, Germany
**Correspondence:** Christiane Höller
*Antimicrobial Resistance and Infection Control* 2017, **6(Suppl 3):**P182

**Introduction:** Patients colonized or infected with antibiotic-resistant bacteria are increasing in health care facilities. There is great uncertainty how to manage these patients and frequently they are excluded from important therapies, because transmission of these bacteria is feared.

**Objectives:** During some therapies like hydrotherapy a large release of bacteria has to be assumed. There are only limited data available on the possibility of bacterial contamination of such pools and their environment. Therefore a study was carried out in hospital pools in order to be able to better estimate the distribution and the associated risk of transmission.
**Methods:** Eleven hydrotherapy pools in Bavaria were sampled repeatedly for antibiotic-resistant bacteria. Samples were taken from different points of the swimming pool system a well as from the pool environment. Water samples were examined additionally for chemical and microbiological routine parameter. Technical details were recorded.
**Results:** Antibiotic-resistant bacteria, mainly *Pseudomonas spp.*, were found in the pool system as well as in the pool environment. Most of the positive pool samples were detected in the balance water and the filtrate, the pool water itself was contaminated seldom. In the environment of the pool most positive samples were found on the cleaning equipment and in the barefoot area.
**Conclusion:** Patients with antibiotic-resistant bacteria may spread the bacteria in the pool environment, but adequate pool water treatment and management of cleaning as well as cleaning equipment can prevent transmission. Special care has to be taken, however, if patients harbour biofilm-producing bacteria like *Pseudomonas* or *Acinetobacter spp*. If the pool water treatment system or the pool hydraulics is faulty or unknown, patients with these bacteria should refrain from participating in hydrotherap

**Disclosure of Interest**
None Declared

### P183

#### Decreasing the risk of health-care associated legionellosis requires joint efforts and close collaboration between all relevant functions in a hospital

Birgitta Lytsy
Department of Clinical Microbiology and Infection Control, Uppsala University Hospital, Uppsala, Sweden
*Antimicrobial Resistance and Infection Control* 2017, **6(Suppl 3):**P183

**Introduction:** Between 2005 and 2009 15 patients acquired legionellosis from the water system at Uppsala University Hospital (UUH), some fatal. Several control measures has been descibed with more or less effectiveness (1).
**Objectives:** To highlight the importance of a water saftey plan with defined roles, responsibilities and ways of communication between all functions can reduce the risk of health-care associated legionellosis.
**Methods:** During 2010 the hospital adopted the WHO water safety plan which included defining roles, responibilities and ways of communications between all relevant functions (2). A steering group chaired by the chief physicia, engineers, janitors, the clinical microbiology laboratory and infection prevention and control staff met regularly. The water safety plan included monitoring the water system twice yearly and defining action limits. Control measures included weekly flushing of taps, plugging dead ends, point-of-use filters in some wards, adjusting cold and warm water temperatures and replacing plastic waterlines with copper. Clear action plans were set up, documented and followed up in regular meetings.
**Results:** Between the years 2010-2017 three patients acquired legionellosis at UUH and all cases could be clearly explained by all functions of the steering group. The first case in 2012 acquired legionellosis from a dental unit which has been described in a recently published case-report (3). All three cases have been reported to the Swedish Social Care Inspectorate and carefully followed up to prevent new cases.
**Conclusion:** When a clear water safety plan was established at UUH the risk of acquiring legionellosis from the hospital water system dramatically decreased. The decisions to take on preventable actions were shared between all functions with different knowledge in order to control the risks that continuously developed over time.

**References**
1. Almeida D et al. Are there effective interventions to prevent hospital-acquired Legionnaires' disease or to reduce environmental reservoirs of Legionella in hospitals? A systematic review. Am J Inf Control 2016; 44:e183-e188.
2. LEGIONELLA and the prevention of legionellosis (www.who.int)
3. Schönning C et al. J Hosp Infect. 2017 Jan 20. doi: 10.1016/j.jhin.2017.01.009. PMID: 28228245

**Disclosure of Interest**
None Declared

### P184

#### Water supply in a new building contaminated with legionella pneumophilae

Leif P. Andersen* [1], Marlene Hoeg[1], Jakob Joensen[2]
[1]Clinical Microbiology 9301; [2]Technical, Copenhagen University Hospital, Rigshospitalet, Copenhagen, Denmark
**Correspondence:** Leif P. Andersen
*Antimicrobial Resistance and Infection Control* 2017, **6(Suppl 3):**P184

**Introduction:** Contamination of water supplies to new buildings with *Legionella pneumophilae* has been described previously.
**Objectives:** To describe surveillance and intervention of a kontaminated water supply in a new building.
**Methods:** Building: A new temporary building in five stores.
Water samples: Were taken from 20 different taps and showers in patient rooms, kitchens and waiting rooms. During the interventions samples were taken from 10 representative taps. Two water samples from each tap were taken and filtrated for total germ count and Legionella germ count.
Interventions: First continuous chlorination with 4 ppm $Cl^-$ of the system was done for three month. Second intervention was high dose chlorine with 400 ppm $Cl^-$ and ensuring that all taps were open until the smell of chloride was clear. The third intervention was to set up a central sterile filter at the water entrance to the building.
**Results:** The building was taken in use before water samples were collected. The first 20 samples showed high total germ count and Legionella germ counts up to $10^6$ *Legionella*/L. Our usual action level is $10^3$ *Legionella*/L. Therefore interventions were initiated with low dose chloride after three month there were no effect of low dose chloride. The high dose chloride was done during one day and dramatically decrease in *Legionella* germ count was seen to less than $10^3$ *Legionella*/L. Shortly after a central sterile filter was mounted and in two years follow up the *Legionella* germ count has been below $10^3$ *Legionella*/L. In addition all taps were automatically opened for at least 1 min each day.
**Conclusion:** First of all the water should have been controlled before the building was taken in use. The time from testing the water supply until the building was taken in use was several month without any precautions was taken. All pipes were made of PEC which increases the risk biofilm formation compared to stainless steel. All taps were electronic which reduces the water flow in the system. Many taps were used very little especially on the hot water site and thus acted as "dead-ends". Microbiological surveillance of water systems in new buildings is essential to prevent infections with *Legionella* spp. and *Pseudomonas aeruginosa*.

**Disclosure of Interest**
None Declared

### P185

#### The impact of chemotherapy and community water supply on schistosomiasis control in a Nigerian Village

Oyetunde Oyeyemi[1], Damilola Olowookere[2], Chibundi Ezekiel[3], Gbenga Oso[2], Alexander Odaibo[2]
[1]Basic Sciences, Babcock University, Ilishan-Remo; [2]Zoology, University of Ibadan, Ibadan, [3]Microbiology, Babcock University, Ilishan-Remo, Nigeria
**Correspondence:** Oyetunde Oyeyemi
*Antimicrobial Resistance and Infection Control* 2017, **6(Suppl 3):**P185

**Introduction:** Nigeria is known to suffer from high burden of schistosomiasis. Most intervention studies are chemotherapy based with little success being achieved due to reinfection and problem of drug resistance.
**Objectives:** The aim of this study was to assess the influence of health education, treatment and water supply on schistosomiasis transmission.
**Methods:** A longitudinal study was conducted on a cohort of 77 individuals living in a typical Nigerian village endemic for schistosomiasis. Mid-stream urine samples collected from the subjects were screened for urogenital schistosomiasis. The village dwellers were educated on the risk factors of schistosomiasis transmission and individuals infected with *Schistosoma haematobium* were treated with a single dose of 40 mg/kg praziquantel. Shortly after the baseline survey, a bore-hole was provided for the people as source of water supply. Then a post-intervention assessment of schistosomiasis transmission was conducted after one year.
**Results:** The baseline prevalence of schistosomiasis in the village was 20.8% while the intensity of infection was 19.3 eggs/10 mL urine. The post-intervention assessment of prevalence and intensity of *S. haematobium* was 2.6% and 1.4 eggs/10 mL urine respectively. The reduction rate in prevalence and intensity of infection was 87.5 and 92.2% respectively.
**Conclusion:** The study affirmed the role of integrated control measures as potent approach to sustaining any viable schistosomiasis mitigation programme in affected areas of Nigeria.
**Disclosure of Interest**
None Declaredpt?>

### Disinfection of environment and devices

#### P186
**Influence of formulation and wipe materials on efficacy of quaternary ammonium compounds (QAC) - containing wet wipe products**
Reiko Matsumura[1], Junko Ochi[1], Masashi Yamamoto[1], Yoshihiko Hirata[1]
[1]Biochemical Laboratory, SARAYA CO., LTD., Osaka, Japan
**Correspondence:** Reiko Matsumura
*Antimicrobial Resistance and Infection Control* 2017, **6(Suppl 3):**P186

**Introduction:** Quaternary ammonium compounds (QAC) are common disinfecting agents used for environmental disinfection of healthcare facilities in Japan. Recently, some commercially wet wipe available products contain both QAC and cleaning agent, possessing disinfecting and detergent properties. However, it has been reported that the efficacy of QAC was interfered by incompatibility of formulation and wipe materials, failing to perform the required efficacy.
**Objectives:** This study evaluated formulations and their compatibility with wet wipe materials to obtain the optimum combination that will not interfere with the efficacy of QAC.
**Methods:** QAC - impregnated sample wipes for environmental disinfection were wrung and evaluated for the efficacy of QAC. Furthermore, EN 16615 was also performed to evaluate the wet wipe products.
**Results:** It was confirmed that the efficacy of QAC was influenced by formulation and wipe materials. The wet wipe product which QAC efficacy was not affected and was compatible with the wipe materials, satisfied the requirements of EN 16615.
**Conclusion:** It is desirable to use wet wipe products which QAC efficacy will not be affected by formulation or wipe materials, for environmental disinfection in health-care facilities.
**Disclosure of Interest**
None Declared

#### P187
**Disinfectant efficacy testing: how updates to current methodology and new method developments impact use concentrations**
James Clarke, Aaron Nightingale, Lauren Oakes
Microbiology - Disinfection and Preservation, Lonza, Manchester, United Kingdom
**Correspondence:** James Clarke
*Antimicrobial Resistance and Infection Control* 2017, **6(Suppl 3):**P187

**Introduction:** It is the responsibility of disinfectant suppliers to substantiate the efficacy claims of their products through adequate efficacy tests in order for healthcare institutions to choose the appropriate disinfectants for any prescribed hygiene regime
**Objectives:** This investigation assesses how recent updates in testing methodology will affect the use levels required for disinfectants to meet the required efficacy criteria.
**Methods:** A selection of widely-used healthcare hard surface disinfectant chemistries were tested according to recently introduced and newly updated European efficacy standard to assess the impact of new methodologies on pass levels and hence use recommendations for users' hygiene plans.
1. Non-mechanical surface test (EN13697:2015) with increased bacterial load and harmonised *Aspergillus brasiliensis* spore preparation (High and low ratios of mature: immature spores)
2. Mechanical surface test (EN16615:2015), a new test method to substantiate wiping use patterns, using both viscose and synthetic wipes (PP/PE).
**Results:** 1) The increase in bacterial load in the EN13697:2015 test norm showed a positive correlation with small increases in use concentration required to meet the pass criteria for all disinfectant chemistries. 2) All disinfectant chemistries showed varying magnitudes of increased concentrations required to meet EN13697 pass criteria against mature *A. brasiliensis* spores when compared to immature spores. 3) The difference in pass concentrations identified for the same product in EN13697 and EN16616 varied between 0 and 50%. 4) The most significant impact on pass concentrations was demonstrated by the substrate composition in the EN 16615 method. QAC-based disinfectants synthetic (PP/PE) wipe substrate gave much lower pass concentrations compared to viscose substrates.
**Conclusion:** It is clear that updates to efficacy methodology and new method developments can impact the concentrations required for products to achieve certain pass criteria. Healthcare institutions should make certain their suppliers routinely assess their products opposite the most recent methods and method versions to verify that use levels are still effective. This will assist hospitals in using efficacious levels of disinfectants in their hygiene regimes for the benefit of their patients and staff.
**Disclosure of Interest**
None Declared

#### P188
**Biofilms in healthcare settings and the mro transmission risk**
Greg S. Whiteley[1], Iain Gosbell[2], Karen Vickery[3], Slade Jensen[2], Jessica Knight[2] on behalf of Surgical Infection Research Group, Australian School of Advanced Medicine, Macquarie University
[1]School of Science and Health, Western Sydney University, Richmond; [2]School of Medicine, Western Sydney University, Liverpool; [3]ASAM, Macquarie University, Sydney, Australia
**Correspondence:** Greg S. Whiteley
*Antimicrobial Resistance and Infection Control* 2017, **6(Suppl 3):**P188

**Introduction:** The presence of multi resistant organisms (MRO) located within biofilms on dry surfaces within hospital settings has been demonstrated. MRO and other microbial species demonstrate enhanced survival characteristics consistent with hydrated biofilms. Traditional methods of treatment including cleaning, disinfecting and sterilisation have been shown to have limited capacity against the defensive features of these biofilms and new treatments and approaches will be required.
**Objectives:** 1. To understand the role of dry surface and other biofilms in survival of pathogenic bacteria within hospital settings;
2. To investigate the frequency of presence and distribution of these biofilms within hospital settings.
**Methods:** Destructive sampling from a range of hospital surfaces and objects, medical devices and in addition a revised sampling method was used to identify biofilm and presence of MRO. Samples were collected in situ from a number of different hospitals and various locations within each hospital. Bacterial recovery methods included selective media, extended recovery times and stored samples were subjected to extended recovery times. Bacteria were regrown using a dry surface biofilm method (Almatroudi 2015) and subjected to disinfectants and sterilisation processes including autoclaving and dry heat. Bacterial survivors were identified using traditional enumeration and live/dead stains.

**Results:** Samples obtained from hospital surfaces and devices frequently showed biofilm visible under SEM. Sustained storage of samples revealed that MRO located within biofilms from dry surfaces have unexpectedly long survival and recovery characteristics. Exposure to a variety of disinfectants and Sterilant processes including chlorine and autoclaving were unable to guarantee inactivation of sessile bacteria located within the dry surface biofilms.

**Conclusion:** MRO located with biofilms on dry surfaces exhibit enhanced survival and transmission characteristics. Standard approaches to disinfection and sterilisation may be compromised where biofilms are present on hospital surfaces and reusable medical devices. A more aggressive approach to cleaning and hygienic treatments such as terminal room cleaning may be required.

Almatroudi et al., J Microbiol Methods:2015:117:171-176

Disclosure of Interest

None Declared

### P189

#### Analysis of coagulase-negative staphylococcus (CONS) strains isolated from the touch surfaces in polish hospital wards for drug resistance and potential biofilm formation

Agnieszka Chmielarczyk, Anna Różańska, Dorota Romaniszyn, Małgorzata Bulanda
Chair of Microbiology, Jagiellonian University Medical College, Krakow, Poland
**Correspondence:** Agnieszka Chmielarczyk
*Antimicrobial Resistance and Infection Control* 2017, **6(Suppl 3):**P189

**Introduction:** Transfer of etiological factors of nosocomial infections through contact route is still very frequent. The problem becomes particularly important in the case of multiresistant bacteria in a hospital environment such as methicillin resistant CoNS (MRCoNS).

**Objectives:** The aim of the study was to characterize the CoNS strains isolated from the touch surfaces in hospital wards for their drug resistance and the ability of biofilm formation.

**Methods:** The research material consisted of 92 strains from 13 wards in 3 hospitals. Identification of the species was carried out using an API-Staph and MALDI-TOF MS. Antibiotics used in this study included erythromycin, clindamycin, gentamycin, ciprofloxacin, trimethoprim/sulphametoxazole, mupirocin. MRSA phenotype was confirmed by detection of the *mec*A gene in PCR. Erythromycin resistance genes (*erm*A, *erm*B, *erm*C, and *msr*A/B) and the *mup* gene was performed by PCR. Biofilm formation was screened using the method with Congo red agar. For an accurate assessment an six-colour scale was used.

**Results:** There were 8 different species of CoNS found, most frequently *S.epidermidis* (34.8%) *S.haemolyticus* (30.4%) and *S.hominis* (15.2%) were isolated. Above 68% of the strains were MRCoNS. The *mec*A gene was confirmed in 69.5%. Among other antibiotics highest resistance was observed to erythromycin (71.7%) and to clindamycin (53.2%). The mechanism MLSB was detected in 51.6% isolates (including cMLSB 35.9% and iMLSB 16.3%) and MSB was confirmed in 17 isolates (18.5%). The gene *erm*B was detected in only one strain and the *msr*A gene in 27 isolates. Resistance to mupirocin was demonstrated in 14 strains and gene *mup* was found in 18 strains. Biofilm-producing strains (very-black, black and almost-black) were 31 (33.7%), and the strains that do not produce biofilm (bordeaux, very red and red) were 61 (66.3%).

**Conclusion:** MRCoNS are not dangerous for people with properly functioning immune systems but they are a risk for patients seriously ill, the elderly, immunocompromised, hospitalized in the ICU. Cleaning and disinfecting of touch surfaces is an obvious action relevant for the MRCoNS control. The project was financially supported by The Polish National Center for Research and Development - decision PBS3/A9/32/2015

**Disclosure of Interest**

None Declared

### P190

#### Chemical free cleaning is safer for health

Elizabeth E. Gillespie
Infection control and epidemiology, Monash Health, Clayton, Australia
*Antimicrobial Resistance and Infection Control* 2017, **6(Suppl 3):**P190

**Introduction:** Monash Health began cleaning without chemicals in 2011. All patient environments are now cleaned using this method, including intensive care units, operating rooms and neonatal and special care nurseries.

**Objectives:** To demonstrate the benefits of this cleaning method

To provide the cleaning assessment demonstrating cleaning compliance

**Methods:** Across 2150 inpatient beds at Monash Health, microfibre cloths of < 0.3 decitex and microfibre mops are dampened with water only. Steam is used intermittently to assist with the cleaning of stubborn stains, for difficult to reach areas such as bed rails, joints of equipment, cavities in equipment and areas where visible soil can accumulate.

Six monthly point prevalence screening for carbapenemase-producing Enterobacteriaceae (CPE) occurs in intensive care units (adult and neonatal), haematology unit and renal transplant unit. Ultraviolet markers (UVM) are used to demonstrate cleaning compliance.

**Results:** This cleaning method is used consistently regardless of a patients perceived or known carriage of disease or multidrug resistant organism, including CPE.

When UVM demonstrates cleaning compliance, sporadic gastroenteritis outbreaks are eliminated and clostridium difficile transmission does not occur. Where CPE carriage has been identified, transmission has been limited to 1 person, prior to the introduction of transmission based precautions. This cleaning system saves time and water, reduces risk of falls and back injury, eliminates chemical exposure, limits cost and protects equipment from chemical damage.

**Conclusion:** Cleaning without chemicals using the cleaning system described, is as effective as using a 2-step cleaning method of detergent followed by disinfectant. However, it is safer, as significant risks are eliminated or reduced. Used for all patient environmental cleaning, this system provides a level of protection not able to be demonstrated using chemical methods.

**Disclosure of Interest**

None Declared

### P191

#### How effective is standard cleaning to remove dirt and microorganisms?

Marc Dangel[1], Hansruedi Brunner[2], Mladen Matulin[3], Andreas Widmer[1]
[1]Division of Infectious Diseases & Hospital Epidemiology, UNIVERSITY HOSPITAL BASEL; [2]Bardusch AG; [3]UNIVERSITY HOSPITAL BASEL, Basel, Switzerland
**Correspondence:** Marc Dangel
*Antimicrobial Resistance and Infection Control* 2017, **6(Suppl 3):**P191

**Introduction:** Environmental cleaning is a fundamental principle of infection prevention in hospitals, but its role in reducing transmission of healthcare-acquired pathogens has been difficult to prove experimentally.

**Objectives:** The aim of the present short study was to evaluate whether adenosine triphosphate (ATP) presence, measured by bioluminescence methods (surrogate marker for dirt), can predict microbiological contamination of hospital surfaces, measured by colony forming units (CFU)/100 $cm^2$.

**Methods:** In four patient rooms we took Rodac plates from different places before and after cleaning. Simultaneously, we determined ATP testing (expressed as relative light units or RLU) from the same surfaces. The different places which we analysed were: floor under the patient's table, patient's table, armrest chair, bedside table, paper roll dispenser, flush tank button, toilet seat, floor at the entrance, door handle.

**Results:** A total 142 samples were taken, 72 Rodac plates and 70 ATP (2 missing data). All samples were positive for coagulase-negative *Staphylococcus, Micococcus ssp, Bacillus* and *Corynebacterium* species. Before cleaning, *Staphylococcus aureus* (MSSA) was found in 27.8% of the samples (10/36). After cleaning, MSSA was found in 22.2% (8/36) and in 2.7% (1/36) methicillin-resistant *Staphylococcus aureus* (MRSA) was found. Analysis of the MRSA strain by PFGE provides strong evidence that the strain originated from a patient of a room at the same ward, but 36.5 meters apart, who had been put on contact isolation.

Before and after cleaning, both dirt measured by ATP ($p < 0.04$) as well as microbiological contamination measured by (CFU)/100 $cm^2$ ($p < 0.02$), resulted in a significant reduction. To our surprise, dirt removal and microbiological reduction were highly correlated ($p < 0.001$).

**Conclusion:** ATP reliably detects residual dirt and can be used for a surveillance of the quality of cleaning. However, detection of *S. aureus* and even MRSA requires microbiological analyses, as these microorganisms are not detectable by ATP. In conclusion, ATP and microbiological sampling provide supplementary information, although ATP predicts to some degree the level of microbiological contamination.

**Disclosure of Interest**

None Declared

### P192

#### Monitoring the effectiveness of terminal cleaning in six Taiwan hospitals

I-Chen Hung[1], Hao-Yuan Chang[2], An-Chi Chen[1], Yeur-Hur Lai[3], Wang-Huei Sheng[4]

[1]Center for Infection Control, National Taiwan University Hospital; [2]School of Nursing, National Taiwan University, [3]Department of Nursing; [4]Department of Internal Medicine, National Taiwan University Hospital, Taipei, Taiwan, Province of China

**Correspondence:** I-Chen Hung

*Antimicrobial Resistance and Infection Control* 2017, **6(Suppl 3):**P192

**Introduction:** Improvement of environmental cleaning in hospitals has been shown to decrease cross-transmission of pathogens. Several objective methods, including aerobic colony counts (ACCs), adenosine triphosphate (ATP) bioluminescence assay and fluorescent marker were developed for cleanliness evaluation. However, few information regarding the correlation of each test and the interpretation of fluorescent marker are locking.

**Objectives:** Our study aim was to monitor the cleaning efficacy in hospitals, and compare the results of targeting surfaces with fluorescent markers with those obtained by quantitative ATP method and ACC method.

**Methods:** This study was conducted a prospective survey of a stratified sampling of 47 wards in 6 hospitals. The same 10 to 12 high-touch surfaces at each room were tested after terminal cleaning (TC), using fluorescent markers, ATP method and ACC method. The aggregate outcome of tested surfaces at each room was calculated TC score. In fluorescence marker, 10 scores meant less than 1/4 residual gel, 5 meant 1/4 to 3/4 residual gel, 0 meant more than 3/4 residual gel. The ACC benchmark was <2.5 colony forming units (CFU)/$cm^2$. An ATP benchmark was <500 relative light units (RLU) in general wards, and 250 RLU in ICU.

**Results:** In total, 830 ACC tests, 1390 ATP tests, and 1668 fluorescence markers from 139 beds were performed. *The mean* ± SD *TC score of* fluorescence marker, ATP method and ACC method were 46.0 ± 25.1, 64.2 ± 20.8 and 96.3 ± 6.2. The TC score of 3 methods had significant correlation each other (fluorescence marker v. ATP method, $r = 0.33$, $p < 0.001$; fluorescence marker v. ACC method, $r = 0.42$, $p < 0.001$; ATP method v. ACC method, $r = 0.25$, $p = 0.025$).

**Conclusion:** Fluorescent marker, as well as ATP bioluminescence assay, could offer objective standards for hospital cleanliness. Simplify the cleaning procedures and strengthen the cleaning of high-touch surfaces can improve the cleaning quality. Further studies are necessary to establish the association of cleanliness and microbial transmission and healthcare-associated infections.

**Disclosure of Interest**

None Declared

### P193

#### A new approach for validation of cleanliness monitoring

Greg S. Whiteley[1,2], Trevor O. Glasbey[1], Paul Fahey[3], Jessica Knight[4], Iain Gosbell[4], Slade Jensen[5]

[1]Whiteley Corporation, North Sydney; [2]School of Science and Health, Western Sydney University, Richmond; [3]School of Science and Health, Western Sydney University, Campbelltown; [4]School of Medicine, Western Sydney University, Liverpool; [5]School of Medicine, Western Sydney University, Lliverpool, Australia

**Correspondence:** Greg S. Whiteley

*Antimicrobial Resistance and Infection Control* 2017, **6(Suppl 3):**P193

**Introduction:** The monitoring of cleanliness levels on reusable medical devices (RMD) and environmental surfaces (ES) within healthcare settings is a potential cornerstone of infection prevention strategies. Recontamination of RMD and ES is a constant and heightens transmission risks of Multi Resistance Organisms (MRO). This study examined a combination of monitoring methods including ATP testing and environmental swabbing to establish the cleanliness of RMD and ES within healthcare settings.

**Objectives:** 1. To improve the validation of surface cleanliness monitoring

2. To improve the reliability of ATP testing data

3. To improve the efficiency of environmental surface microbiology

**Methods:** RMD and ES were subjected to ATP testing using commercially available devices and consumables. A new sampling algorithm was used to reduce the risks of inherent variability arising from the ATP testing. The algorithm uses a duplicate sampling approach combined with an intervention step. Microbiological methods used an aggressive swabbing technique intended to reveal both transient and resident microbes of interest. Bacterial elucidation applied extended recovery periods in selective media and identification using MALDITOF.

**Results:** Use of the ATP testing algorithm improved the reliability of ATP testing data ($P = 0.001$)and suggests new lower ATP levels for cleanliness acceptance. Aggressive swabbing technique demonstrated a surface contamination rate for MRO/biofilms as high as 17.65% of high touch objects located within the Intensive Care Unit.

**Conclusion:** The use of ATP testing has been contentious due to issues including variability and lack of correlation with relevant microbial contamination. The new sampling algorithm improves reliability to better than 99.9% through the mitigation of inherent variability, and suggests more severe standards for cleanliness standard setting (<25RLU – brand specific). The use of aggressive swabbing more reliably indicates MRO presence, even when located within dry surface biofilms. These new methods offer improved cleanliness monitoring for use within healthcare settings.

**Disclosure of Interest**

None Declared

### P194
### Withdrawn

### P195
### Using fluorescent marker to evaluate the effectiveness of surface cleaning work in patient room, procedure room and icu room at HCMC University Medical Center
Tuan M. Huynh, Nguyen Thi Hong Van, Nguyen Vu Hoang Yen, Trinh Thi Thoa, Pham Thi Lan, Ha Thi Nha Ca, Tran Tri Kien
Infection Control, Ho Chi Minh City University Medical Center, Ho Chi Minh City, Viet Nam
**Correspondence:** Tuan M. Huynh
*Antimicrobial Resistance and Infection Control* 2017, **6(Suppl 3):**P195

**Introduction:** Hospitals must implement control for environmental surface cleaning and disinfection, especially "high touch" area, to ensure that surfaces are appropriately clean and disinfected, so that there is no cross transmission to patient as well as healthcare worker. However, the usual control method, by using visual checklist, is not precise enough. Therefore, we carried out this study, using fluorescent marker, to evaluate the effectiveness of routine cleaning processes.
**Objectives:** Determine the rate of unsatisfactory cleaning by using fluorescent marker, at "high touch" areas in patient/procedure/ICU room at HCMC UMC.
**Methods:** "High touch" areas related to injection vehicle, ventilator, toilet, patient room/procedure room/ICU rooms were marked for surveillance.
We evaluated cleaning result using "visual checklist" as usual; using "fluorescent marker" and calculating TDC Score following CDC guideline in a manner of single-blind design.
**Results:** From 1/2016 to 6/2016, we collected a total of 883 "high touch" areas, evaluated 100% well using visual checklist; followed were their TDC Scores:
1. Medical equipment
Patient room: TV power button ~ 66.67%
ICU room: ventilator surface ~ 89.47%
2. Toilet
Patient room: toilet paper container surface ~ 85.71%, hand sink faucet ~ 86.67%, handrails ~ 92.86%, door-knobs ~ 93.33%
Procedure room: hand sink faucet, toilet faucet, handrails ~ 90,91%
ICU room: handrails ~ 68.42%, toilet tap ~ 85%, hand sink faucet ~ 94,74%
3. Other high touch points
Patient room: hanging table records ~ 60%, crank/bed height adjustment button ~ 78.57%, mattress side ~ 86.67%, nurse call button ~ 50%, wall light switch ~ 73.33%
Procedure room: crank/bed height adjustment button ~ 42.86%, mattress side ~ 81.82%, bed side rails ~ 90.91%, pressure regulator knob suction phlegm ~ 81.82%, wall light switch ~ 54.55%
ICU room: trunk of IV pole ~ 66.67%), crank/bed height adjustment button ~ 68.42%, panel hanging profile ~ 68.75%
**Conclusion:** By using fluorescent marker, we found significant rates of high-touch points in patient/procedure/ICU rooms which were not clean adequately although they were evaluated well cleaned using visual checklist.

**References**
CDC, 2010, "Option for Evaluating Environment Cleaning Level II, Appendix B_Objective Methods for Evaluating Environment Hygiene"
Disclosure of Interest
None Declared

### P196
### Monitoring the effect of an educational intervention on cleaning and disinfecting with ATP bioluminescence
KEC Jansen-te Braak[1], AFJ de Haan[2], GK Terlaak-Harbers[1], A Voss[1], J Hopman[1]
Medical Microbiology / Hygiene Infection Prevention; [2]Department for Health Evidence, Radboud University Medical Centre, Nijmegen, Netherlands
**Correspondence:** KEC Jansen-te Braak
*Antimicrobial Resistance and Infection Control* 2017, **6(Suppl 3):**P196

**Introduction:** Microbiological contamination of the hospital environment is a source for hospital acquired infections. We aimed to improve the quality of environmental cleaning and disinfecting through the introduction of an educational intervention (EI).
**Objectives:** Aim of the present study was to evaluate the impact of an educational intervention on the improvement of knowledge and the outcome of cleaning and disinfecting.
**Methods:** A before-after EI study was conducted on the cardiac ward of a 600-bed academic hospital from May to October 2016. Before intervention, cardiac health care workers responsible for cleaning after discharge were interviewed and observed during cleaning. An application was developed to register these scores. The target was an overall increase of correct measured elements (ME) and a decrease of (semi) wrong ME up to 0%. Cleanliness of high-touch surfaces was measured by adenosine triphosphate (ATP). The cut-off for cleanliness after disinfection was set at 100 relative light units (RLU). A total of 60 ATP measurements of three predefined high-touch room surfaces were conducted before and after cleaning. After EI, the "before" procedure was repeated. Per discharge environment ($n = 10$), the three high-touch surfaces were pooled, and the geometric mean was log transformed.
**Results:** The knowledge of cleaning and disinfecting improved. Before EI the correct ME were 46% and after EI 63%. ATP data expressed in relative light units (RLU) after cleaning before EI (311.1 RLU) were significant reduced after EI (158.8 RLU; $P \leq 0.028$).
**Conclusion:** EI improved the level of knowledge, although the objective was not fully achieved. After EI a significant decrease in environmental contamination was measured, however it still exceeded the pre-set cut-off of 100 RLU.
**Disclosure of Interest**
None Declared

## Sterilization

### P197
### Establishment of penetration time on medical device product families based on iso 17665-3 during performance qualification of steam sterilization cycles
Roel Beltran Castillo
Central Sterilising Services Department, Macquarie University Hospital, Macquarie University, Australia
*Antimicrobial Resistance and Infection Control* 2017, **6(Suppl 3):**P197

**Introduction:** Reusable medical devices (RMDs) vary significantly in material construction and geometric configuration; ISO 17665-3 provides information to group RMDs into families fit for a steam sterilization process. This underpins the need to establish penetration time at equilibrium throughout the RMD surfaces based on delegated product families, a data necessary to ensure sufficient exposure to steam sterilization process is delivered throughout the RMD.
**Objectives:** This study aims to provide an investigation on the credibility of ISO 17665-3 requirements. Is it really necessary to comply with standards to meet lethality of steam sterilization process?

**Methods:** All RMD item/s is grouped according to designation of product family based on ISO 17665-3. Specific attributes are tabled presenting products that can be grouped together as product family identified for a particular steam sterilization process. The most difficult to sterilize item/s in each product is chosen as a master product. This master product is then used as part of the reference load during performance requalification (PQ) of the steam sterilizer. The most challenging item with identified resistance (based on ISO 17665-3) in the master product is chosen for the study of penetration time. A thermometric measuring device attached to a software reader will be strategically located on the chosen RMD. This method provides live information of penetration time until equilibrium with the time lapse to attain plateau of identified lethal temperature.
**Results:** Based on data gathered, the material construction and complexity of RMD surface is directly related to resistance of heat transfer. This means RMDs with complex configuration and/or mixed material construction provided a longer time to reach the innermost parts and render the RMD surface exposed to lethal temperature. This confirms the ISO 17665-3 approach grouping RMDs into family of similar penetration resistance in order to attain lethality of process.
**Conclusion:** In conclusion it is beneficial establish penetration time of RMDs during annual PQ. This will address the reprocessing requirements to ensure sterilization lethality is achieved.
**Disclosure of Interest**
None Declared

### P198

#### Evaluation of a mist ultraviolet disinfection device for impact on bacterial contamination levels of laryngoscope handles

Elhadi Elghouati[1], ludivine Lorant[2]
[1]Infection control unit, Meulan les Mureaux hospital, Meulan-en-Yvelines; [2]clinical laboratory, Tonnerre hospital, tonnerre, France
**Correspondence:** Elhadi Elghouati
*Antimicrobial Resistance and Infection Control* 2017, **6(Suppl 3):**P198

**Introduction:** Laryngoscope is the medical device most commonly used for tracheal intubations. The Center for Disease Control and Pervention classifies Laryngoscope Handle (LH) as semi critical device. Failure to disinfect appropriately LH may caused infection and even death [1]. Decontamination technologies that utilize mist ultraviolet light C (M.UV-C) may be effective in reducing bacterial burden of LH
**Objectives:** Compare the automated M.UV-C technology and standard manual protocol with respect to their ability to reduce bacterial contamination of reusable LH
**Methods:** This prospective study was conducted between 15 decembre 2016 and 15 march 2017 in a 500-bed tertiary care hospital. A before-and-after intervention study was designed to compare the bacterial load of LH according the disinfection procedure. LH from the adult operating rooms were cleaned after each use according two protocols: manual protocol based on disinfectant wipes for 2 months and M.UV-C technology the subsequent 2 months. For each period, 45 sawbs were collected in order to determine bacterial contamination. Samples were collected from LH considered clean and ready for use. LH were swabbed with sterile cotton swabs moisturized with sterile normal saline and placed into a nonnutritive transport medium.Sawbs were discharge in 2 ml of sterile normal saline, samples (500 µl) were spread on count agar plates with BCP and incubated at 37 °C for a total of 48 hours. Colony forming units (CFU) were counted on each plate.
**Results:** In total, 90 samples were collected from 30 LH. Colony counts varied by cleaning protocol: manual protocol (mean = 46.5 CFU /ml) vs M.UV-C (mean = 0.75 CFU /ml). The mean bacterial load of LH decreased significantly from 46.5 to 0.75 UFC /ml ($P < 0.001$, Wilcoxon test) after the cleanning portocol was switched from standard to M.UV-C technology.
**Conclusion:** The M.UV-C technology appears to be superior vs manual cleaning for bacterial burden. The M.UV-C disinfection device significantly reduces bacterial contamination and mimimze risk of cross contamination from LH.

**References**
[1] UK Alert.Ref: MDA/2011/0096.27 September 2011
**Disclosure of Interest**
None Declared

### P199

#### Identification of the impact of education and mentoring on medical device reprocessing practices in three hospitals in Cotonou, Benin

Olive M. Fast
School of Nursing, Mount Royal University, Calgary, Canada
*Antimicrobial Resistance and Infection Control* 2017, **6(Suppl 3):**P199

**Introduction:** Preventing post-operative infections through the proper sterilization of medical devices is a challenge in low to middle income countries (LMICs) due to inadequate equipment and unsterile technique, compounded by a lack of training in instrument reprocessing practices.
**Objectives:** A mixed methods study utilizing surveys, interviews, and sterilization equipment testing was conducted from September 2016 to May 2017, to identify the impact mentorship and education had on medical device reprocessing (MDR) staff knowledge and the processing of medical devices in three hospitals in Cotonou, Benin, West Africa.
**Methods:** Participants received 40 hours of classroom training in MDR as well as mentoring in their work setting. A hospital assessment tool was used prior to education sessions and six weeks following the mentoring timeframe. Participants also participated in a 20-30 minute semi-structured interview and completed a survey prior to and following education sessions. Quantitative and qualitative data was analysed using the Dedoose data analysis system.
**Results:** Initial findings identify the value added component education and training have on MDR personnel. Participants self identified how their practice had changed due to a greater understanding of infectious processes as related to instrument sterilization. They also noted that education had changed how they approached their work, identifying spending more time ensuring instruments were thoroughly cleaned. While comparisons of survey results indicate retention of knowledge and improved understanding of MDR practices comparisons of hospitals assessments did not reflect the same level of process change that participants identified.
**Conclusion:** Findings from this study highlight the value education and mentoring have on both participants' knowledge and understanding of how to do their work and the value they placed on MDR practices. Implications for clinical practice are that formal education on MDR needs to be mandatory for all MDR staff if risks of surgical site infections are to be reduced. However, analysis of MDR processes before and after training suggest that while training and mentorship produce positive changes in individuals, the organization and culture they work in produce barriers to improvements that need to be addressed by others with authority.
**Disclosure of Interest**
O. Fast Grant/Research support from: Research partially funded by Mercy Ships Canada and Mount Royal University, Other conflict with: I am the chair of Sterile Processing Education Charitable Trust (SPECT), the organization that developed and implemented the education and training, which are the focus of this research project

### P200

#### Effect of surfactant in an alkaline detergent for steady cleaning performance during medical device reprocessing in washer disinfectors

Glen Lelyn Quan, Yuka Oda, Emiko Kawamukai, Yoshihiko Hirata
[1]Biochemical Laboratory, SARAYA CO., LTD., Osaka, Japan
**Correspondence:** Glen Lelyn Quan
*Antimicrobial Resistance and Infection Control* 2017, **6(Suppl 3):**P200

**Introduction:** Thorough cleaning of medical devices is essential in infection control and prevention during medical device (MD) reprocessing. The use of washer disinfectors plays a vital role in effective reprocessing as they ensure validated reprocessing cycles and, as a closed system, reduce human exposure to process chemistries.(1) Excessive foaming is often a problem for WDs as it can affect machine performance, decelerate jet pressure and spray arms, impede detergent performance, and therefore, compromise the quality of the cleaning process. Among the commonly used detergents, alkaline detergents especially, react with organic soil and cause excessive foaming.

**Objectives:** This study explored the impact of surfactant in an alkaline detergent to control excessive foam formation in WDs for steady cleaning efficiency towards medical devices in the presence of organic soil.

**Methods:** The foam formation of an alkaline detergent, with and without surfactant, was compared with a representative washer disinfector WD-32 (Saraya Co., Ltd.), in the presence of heparinized sheep blood. The detegency was then compared using soiled SUS test piece model that mimic simple and complex MDs.

**Results:** In the presence of heparinized sheep blood, the alkaline detergent sans surfactant showed excessive formation of foam, halting the machine in mid process, whereas with the surfactant-containing detergent, foam formation was suppressed, enabling complete and successful reprocessing. In terms of detergency, the surfactant-containing alkaline detergent not only exhibited higher detergency, but was also able to minimize the difference in detergency between simple and complex test device model in the presence of heparinized sheep blood, as well. With this, steady detergency performance was shown achievable regardless the type of MDs used even in the presence of organic soil.

**Conclusion:** The synergistic action of surfactant with alkaline detergent not only prevented machine failure and ineffective cleaning from excessive foaming, but was also able to deliver steady detergent action in reprocessing simple to complex MDs, increasing the quality of cleaning performance during medical device reprocessing.

(1) ESGE-ESGENA Guideline: Cleaning and disinfection in gastrointestinal endoscopy (Update 2008)

Disclosure of Interest

None Declared

### P201

#### Surgical equipment sterilization practice among health care workers in private hospitals in Zaria, North Western Nigeria

Lawal Amadu[1], Abdulhakeem A. Olorukooba[1], Aisha A. Abubakar[1], Muhammad S. Balogun[2], Shamsudeen Aliyu[3], Shamsudeen S. Yahaya[4], Muawiyyah B. Sufiyan[1], Bola B. Lawal[5], Itodo David[1]
[1]Community Medicine Department, Ahmadu Bello University Zaria, Kaduna state, Nigeria, Zaria; [2]AFENET, African Field Epidemiology Network, Abuja; [3]Medical Microbiology, Ahmadu Bello University Zaria, Kaduna state, Nigeria, Zaria; [4]State Primary Health Care agency, State Ministry of Health, Katsina; [5]Nigerian Field Epidemiology and Laboratory training program, NFELP, Zaria, Nigeria
**Correspondence:** Lawal Amadu
*Antimicrobial Resistance and Infection Control* 2017, **6(Suppl 3):**P201

**Introduction:** Sterilization and disinfection are indispensable for ensuring that surgical instruments do not transmit infections to patients. Ineffective sterilization and disinfection of surgical equipment would invariably result in Hospital acquired infections(HAI). The practice of sterilization among health care workers is generally poor. While most studies dwell on the practice among public health institutions, there is little or no information on the situation among Private health workers.

**Objectives:** This study aims to examine the practice of disinfection and sterilization of surgical equipment among health workers in Private hospitals in Zaria, Nigeria

**Methods:** A descriptive cross sectional study was carried out among 144 respondents selected using a multi-stage sampling technique. Pretested, pre-coded, self-administered questionnaire was used to elicit information from the respondents. Data was entered and analysed using IBM SPSS v 21.0. Test for association was carried out at a significant level of $P \leq 0.05$.

**Results:** Majority (45.8%) of the respondents were doctors, and more than half of them had less than 5 years working experience. Only 21.5% had good knowledge of sterilization while 96.5% had positive attitude. However, the practice of sterilization was found to be low as only 38.2% had appropriate practice. There was no statistical significant association between years of working experience and practice of sterilization ($P = 0.719$)

**Conclusion:** The practice of sterilization among private health workers was found to be poor. There is need for the Ministry of Health in collaboration with the Guild of Medical directors to institute and maintain training and education of Private health workers on sterilization and its importance in prevention and control of HAIs.

**Disclosure of Interest**

None Declared

### P202

#### Improved technique of rapid and accurate peracetic acid test strips with sharp color by comprising advanced indicator solution

Madan A. Mali, Satomi Sakai, Emiko Kawamukai, Yoshihiko Hirata
Biochemical Laboratory, SARAYA CO., LTD., Osaka, Japan
**Correspondence:** Madan A. Mali
*Antimicrobial Resistance and Infection Control* 2017, **6(Suppl 3):**P202

**Introduction:**

Test strips are normally used in disinfection process of medical devices to monitor the effective concentration of high level disinfectants such as peracetic acid. Currently, the peracetic acid test strips available in the market exhibit different color shades that require 20 seconds to 2 minutes reaction time for evaluation. At times when the color judgment falls between two color shades, it will be difficult for users to judge the results. If strips interpretation will be wrong, there is a risk for ineffective disinfection process of medical devices. With the development of sharp colored strips, such incidents can be avoided, and will make users monitor the concentration of peracetic acid more easily and effectively.

**Objectives:**

To obtain sharp color of rapid and accurate peracetic acid test strips by comprising advanced indicator solution

**Methods:**

1. *Preparation of Indicator solutions*

The base indicator solution A was prepared with combination of iodide-starch solution. The advanced indicator solution B was prepared with the combination of iodide-starch and water soluble additives.

2. *Test methods*

The test strips were comprised with solutions A and B respectively. Each strip was soaked into peracetic acid solutions for few seconds and strip color was monitored after suitable reaction time. The obtained strip colors of solution A and B were compared.

**Results:**

1. The strip comprised with base indicator solution obtained the different color shades.
2. The strip comprised with improved indicator solution obtained sharp color for PASS and FAIL criteria within 15 seconds.

PASS evaluation - complete black color at effective concentration of peracetic acid solution

FAIL evaluation - incomplete color with white spots at ineffective concentration of peracetic acid solution

**Conclusion:**

The rapid and accurate test strips obtained distinguishable sharp color with improved indicator solution. Due to shorter reaction time and easy judgment with PASS and FAIL criteria, these strips are very convenient, user friendly and time- saving for monitoring the concentration of peracetic acid effectively in disinfection process of medical devices.

**Disclosure of Interest**

None Declared

### ICPIC Clip award

#### P203
**Be a hero and defend health! join the preventive measures force: hand hygiene, respiratory hygiene, and cough etiquette**

Shu-Hui Tseng, on behalf of Taiwan Centers for Disease Control
Division of Infection Control and Biosafety, Taiwan Centers for Disease Control, Taipei City, Taiwan
**Correspondence:** Shu-Hui Tseng
*Antimicrobial Resistance and Infection Control* 2017, **6(Suppl 3):**P203

**Abstract video clip:** An educational short animation made to advocate hand hygiene, respiratory hygiene and cough etiquette to the general public, especially those who visit healthcare facilities for any reason (seeking treatment, accompanying a family member, visiting an inpatient, etc.).
The first part of the video briefly touches related topics such as pathogens, the discovery of penicillin and the subsequent development of more antibiotics, and antimicrobial resistance.
The educational aspect of the video emphasizes on the everyday preventive measures that can be taken by anyone: hand hygiene and wearing surgical masks. This part details the proper ways to:
1. Wash your hands using soap and water.
2. Sanitize your hands using alcohol hand sanitizer.
3. Put on a surgical mask.
For fun factor, pathogens are portrayed as cartoon monsters, and the preventive measures advocates as a Power Rangers-styled superhero team.
None Declared

#### P204
**Hands to yourself. just imagine what you do to your patients**

Ana S. Bispo[1], Carlos Palos[1], Pedro Pereira[2]
[1]Infection Control and Antibiotics Committee, HOSPITAL BEATRIZ ÂNGELO, Loures; [2]IADE.Universidade Europeia, Lisboa, Portugal
**Correspondence:** Ana S. Bispo
*Antimicrobial Resistance and Infection Control* 2017, **6(Suppl 3):**P204

**Abstract video clip:** HANDS TO YOURSELF is a short clip recorded at Hospital Beatriz Ângelo, a 425-bed, JCI-accredited, general hospital, during the anual presentation of anual data on infection control and antibiotics.
Attendants, mainly physicians and nurses, received pens previously powdered with special UV visible light powder.
When the lights switched for UV light, infection control nurse asks them to look at each other's faces.
Messages about bacteria (namely multidrug-resistant bacteria) in hands of professionals, and about the 5 moments of hand hygiene are displayed.
A free instrumental version of "Hands to my self" (Selena Gomez) was chosed and is synchronized with images.
The clip is now a part of every session about hand hygiene in the hospital.
Clip is directed by Pedro Pereira, from IADE-Laureate International Universities, an international reference school of design.
**Disclosure of Interest**
None Declared

#### P205
**Antibiotics awareness week: reserve antibiotics for a real bacterial infection**

Sung-Ching Pan[1], Guan-Jhou Chen[1], Kuei-Lien Tien[2], Wang-Huei Sheng[1], Yee-Chun Chen[1], Shan-Chwen Chang[1] on behalf of Center for Infection Control, National Taiwan University Hospital
[1]Department of Internal Medicine; [2]Center for Infection Control, National Taiwan University Hospital, Taipei
**Correspondence:** Sung-Ching Pan
*Antimicrobial Resistance and Infection Control* 2017, **6(Suppl 3):**P205

**Abstract video clip:**
**Background**
Since 2015, World Health Organization had launched Antibiotics Awareness Week annually under the menace of emerging antibiotics resistant pathogen globally. Echoing with the global campaign, National Taiwan University Hospital (NTUH) had organized a video campaign in conjunction with the Antibiotics Awareness Week in 2017.
**Method**
Center for Infection Control of NTUH and Taiyuan Arts and Culture Foundation had collaborated on 3-minute clip to address the importance of appropriate use of antibiotics. The video was created with traditional Taiwanese puppets to facilitate the connection with the audience and was available in Chinese (both of Mandarin and Min dialect) and English version.
**Result**
The theme of Antibiotics Awareness Week, 2017 is "Reserve antibiotics for a true bacterial infection". The video demonstrated a scenario of common cold and started with how we tell the difference between common cold and influenza infection, then followed by the importance of diagnosis to decide whether antibiotics is needed. Finally, we addressed the importance of hand hygiene, cough etiquette and isolation as key infection control measure to prevent disease transmission. The video had premiered at 17$^{th}$ of February, 2017 during Asia-Pacific Medical Students' symposium.
**Conclusion**
Successful antibiotics stewardship program warrants the cooperation of the patients and health care workers (HCWs). However, traditional health education programs tend to be rigid and inefficient when communicating to the general population. We aim to promote the antibiotics awareness with an eye-catching video staging Taiwanese puppet to draw the attention and to connect with our audience.
**Disclosure of Interest**
None Declared

#### P206
**Hand hygiene flash mob in nursing home: when the residents, the actors of their own health become actors as well**

Olivia Weinachter[1], Emeline Monnot[2], Christelle Savouret[3], Christine Duvernay[4], Stéphanie Morizet[5], Nathalie Floret[6]
[1]centre hospitalier, Vesoul; [2]centre hospitalier, Dole; [3]centre hospitalier, Trévenans; [4]centre hospitalier, Quingey; [5]centre hospitalier, Avanne; [6]Antenne régionale de lutte contre les infections nosocomiales, centre hospitalier universitaire, Besançon, France
**Correspondence:** Nathalie Floret
*Antimicrobial Resistance and Infection Control* 2017, **6(Suppl 3):**P206

**Abstract video clip:** Hands are likely to play a significant role in microorganism cross-transmission.
Hand hygiene is one of the primary modes of reducing health care-associated infection (HCAI) and of enhancing patient safety.
In 2009, the WHO Guidelines on Hand Hygiene in Health Care has encouraged partnership between patients, their families, and HCW to promote hand hygiene in health-care settings.
The French national infection control program released in 2015, aims to include HCAI prevention in a lonely program both around patient and shared on the 3 sectors of care delivery. Patients are expected to play a major role of actor in their own health.
In Franche-Comté (a region located in eastern France), since 2012, a regional organization based on a team of 10 hygienist nurses coordinated by the regional unit for nosocomial infection control, has been implemented in order to enhance HCAI prevention in nursing home.
As patient empowerment is an evolving concept in health care that has now been expanded to the domain of patient safety, to create tools to engage them in improving their hand hygiene was our priority.
In 2015, a fun and educational tool to teach residents the several indications for hand hygiene was developped.

The aim of this new project was to provide another tool to support residents' awareness focusing on the technique for hand hygiene.
A choreography taking into account the different steps of the hand hygiene technique was built and a music chosen. Duties to the Sacem organization have been paid.
A few number of nursing homes were asked to take part. Nursing home participation was on a voluntary basis.
Six of them agreed to participate. In each of them, the hand hygiene technique was learned to a dozen of residents after they have expressed their consent concerning the right to one's image.
Choreography was taught to the residents in partnership with the animation team of each nursing home.
In each place, several rushes featuring residents rubbing their hands have been set up and analyzed by the working group to provide the movie entitled "love tes mains".
This movie will be used to support residents hand hygiene awareness sessions in nursing home.
**Disclosure of Interest**
None Declared

### P207
**Hand hygiene promotion with celebrities**
Borbála Szél
Infection Control Unit, Albert Szent-Györgyi Health Center at University of Szeged, Szeged, Hungary
*Antimicrobial Resistance and Infection Control* 2017, **6(Suppl 3):**P207

**Abstract video clip:** Since 2009, when the well-known 5 dancers in the black dress (*Ô les mains!* – Geneva Unviversity Hospitals) taught us the basic hand hygiene rules according to the WHO, thousands of similar videos and clips have flooded the Internet. There is no doubt that hand hygiene is the leading measure to prevent healthcare associated infections, therefore performing best practice hand hygiene is fundamental in health-care facilities. These educational videos transfer essential information in minutes while entertain the recipient. Lecturers have preferences for using these audiovisual tools in their presentations to make them more attractive, thus the need for new awareness rising videos have increased. The aim of this clip was to demonstrate hand hygiene by a novel way. Hand hygiene scenes were cut out from movies and cartoons, then were edited into a 3 minutes long educational video. It shows how to wash and rub hands and when it should be performed. Also the clip represents the five moments for hand hygiene. With the help of this clip health-care workers who have insufficient hand hygiene compliance could improve their technique and attitude and become a pro in hand hygiene. For those health-care workers who are already hand hygiene masters, this clip will provide them the opportunity to see the gorgeous Anne Hathaway or the naked Brad Pitt.
**Disclosure of Interest**
None Declared

### P208
**Hand hygiene painting festival, Mashhad-Iran**
Mohammad Hassan Aelami[1], abbas Mohajeran[2], Maryam Khalesi[1]
[1]Pediatrics; [2]Mashhad University of Medical Sciences, Mashhad, Iran, Islamic Republic Of
**Correspondence:** Mohammad Hassan Aelami
*Antimicrobial Resistance and Infection Control* 2017, **6(Suppl 3):**P208

**Abstract video clip:** Hand Hygiene Painting Festival Mashhad-Iran
Introduction:Health-care associated infections are a major public health problem especially in child care centers and schools. The best way for prevention of these infections is hand hygiene.
Methods:We encouraged performing hand hygiene in child care centers and schools of Mashhad-Iran by participating in a painting festival.The subject for competition was "clean hands; save lives".Many organs such as Education and Training Office,welfare Office,Municipality,Institute for the Intellectual Development of Children and Young Adults and Mashhad University of Medical Sciences cooperated in this festival.
Results:More than 14000 paintings paricipated in this festival in 2014-2015. More than 800 awards including 30 bicycles were delivered to winners.
Conclusion:Hand hygiene can be promoted by different tools including painting festival.
**Disclosure of Interest**
None Declared

### P209
**CCLIN Sud-Ouest's Vlog - Exdr**
Caroline Bervas, Vincent Deroissart, Pierre Parneix, Elisabeth Sousa, Anne-Gaëlle Venier, Catherine Dumartin
CCLIN Sud-Ouest, Bordeaux, France
**Correspondence:** Caroline Bervas
*Antimicrobial Resistance and Infection Control* 2017, **6(Suppl 3):**P209

**Abstract video clip:** *eXDR… What does this term mean? To find out more with fun, watch our little video! It talks about eXDR, emerging extensively drug-resistant bacteria: carbapenemase-producing enterobacteriaceae (CPE) and glycopeptide-resistant enterococci (GRE). eXDR is a priority theme in French Program for prevention of healthcare-associated infection. So, with this vlog, French southwestern Centre for Healthcare Associated Infections prevention & Control wants to explain in a fun way what eXDR are. It describes the French epidemiology of eXDR and the problems encountered with these germs: their wide diffusion and the therapeutic dead-end. It presents two solutions to fight eXDR. The first is to avoid their emergence by the appropriate use of antibiotics. The second solution is to prevent their dissemination, first by being able to identify it, then to avoid the transmission of eXDR by hands, excreta, or equipment by implementing the specific measures.*
**Disclosure of Interest**
None Declared

## Catheter-associated bloodstream infections (BSI)

### P210
**Hand hygiene and catheter related blood stream infection control in intensive care unit, University Hospital in Latvia**
Agita Melbarde-Kelmere
Riga Eastern University Hospital, Riga, Latvia
*Antimicrobial Resistance and Infection Control* 2017, **6(Suppl 3):**P210

**Introduction:** Pauls Stradins University Intensive care unit (ICU) staff has a hand hygiene intervention study and CVC bundle intervention study. The organization of infection control at the hospital ICU was changed by updating and implementing patient treatment procedures.
**Methods:** A time-series prospective study about bacteraemias was carried out in ICU. One of the major aims of the study was to define and to summarise different indicators, perform interventions, and reduce the NI incidence rates.The duration of the study was 4.5 years (from 20 August 2008 until the end of June 2013). Throughout the study, bacteraemia results for ICU patients were collected and the result of the performed interventions was constantly evaluated, in addition also hand hygiene monitoring was performed. A special procedure for CVC usage (insertion, treatment and removal) was implemented in PSCUH in 2009. For hand hygiene (HH) monitoring, the WHO Hand Hygiene Monitoring Tool was used.
**Results:** From 20 August 2008 to 28 June 2013, 5739 hand hygiene observations were performed. Higher ratio of the hand hygiene compliance was seen when the feedback with the medical staff concerning the acquired results and their changes was established. The average hand hygiene compliance within the study was 36.7%. From January 2011 to June 2013, 1066 adult patients with CVC enrolled (12 485 CVC days). The incidence of CRBSI during pre-intervention period was high (10.1 per 1000 CVC days, CI = 7.9-12.8) but after …

bundle" implementation CRBSI rate decreased (3.8 per 1000 CVC days, CI = 2.5-5.8).

**Conclusion:** Implementation of infection control has proved its efficiency, by carrying out intensive training programme related to hand hygiene, CVC usage and treatment for nurses and doctors; it was possible to reduce CRB more than seven times. Although the results obtained during the hand hygiene intervention are not long-lasting, it is possible to improve those by repeating the intervention.

**Disclosure of Interest**

None Declared

### P211

#### 5 Year surveillance of clabsi in a tertiary care private sector nicu in Pakistan

Ali S. Hussain, Shabina Ariff
The Aga khan university hospital, Karachi, Pakistan
**Correspondence:** Ali S. Hussain
*Antimicrobial Resistance and Infection Control* 2017, **6(Suppl 3):**P211

**Introduction:** Amongst HAI, CLABSI are the most important cause of morbidity and mortality especially in critically ill patients. Due to many factors in low resource countries, CLABSI are either underreported, meaning that the impact of CLABSI may be much higher than what is reported.

**Objectives:** To determine the frequency,Characteristics and microorganism profile of CLABSI from our NICU.

**Methods:** We retrospectively analyzed our prospectively collected infection control surveillance data base from 2011-2015. All cases who were classified by our hospital's infection control team as CLABSI using the CDC NSHN definition were enrolled. Microorganisms that caused CLABSI were identified. Basic demographic and anthropometric data along with outcomes were recorded. Catheter type and its dwell time was recorded. Amongst risk factors, TPN days, Line days and line day at which CLABSI confirmed were recorded. CLABSI rates/1000 central line days and Device utilization ratio were calculated.

**Results:** 2046 babies were admitted in our NICU during the study period with a total of 17881 patient days and 6467 device days.Average yearly central line utilization ratio was 0.36/ year. A total of 183 patients had 245 blood stream infection out of which 57(31%) were labelled CLABSI. Our cumulative CLABSI rates were 8.9/1000 CL days.Amongst CLABSI,37(65%)babies were male.Median weight was 1.68 kg (IQR 1.25, 2.64) whereas median gestational age was 33 weeks(IQR 29, 37).Median time from line insertion to CLABSI was 10 days(IQR 5 to 18 days).Combination of UA and UV constituted 17(30%) CLABSI episodes followed by PICC line 15(26%).Gram-ve organisms were most common followed by CONS and candida. Klebsiella and Acinetobacter constituted 45% of the total infections.Distribution of microorganisms amongst different central lines were similar except for acinetobacter in which 9 out of 10 CLABSI were associated with UA/UV lines. 28 (49%) of our CLABSI patients died.In adjusted multivariate analysis only duration of central line >30 days was found as independent predictor for CLABSI(IRR 2.5, 95%CI 1.24-5.06)

**Conclusion:** We report a high CLABSI rate with substantial CLABSI related mortality. Higher CLABSI rates translating into eventual morbidity and mortality in developing countries projects the poor state of affairs and highlights the importance and need of investment in health care infrastructure.

**Disclosure of Interest**

None Declared

### P212

#### Epidemiology of central line associated blood stream infections (CLABSI) over ten years in a medical-surgical ICU in a tertiary care center in Lebanon

Nada K. Zahreddine[1], Joseph Tannous[2], Tala Kardas[1], Rihab Ahmadieh[1], Zeina Kanafani[3], Souha Kanj Sharara[3]
[1]Infection Control and Prevention Program; [2]American University of Beirut Medical Center, Beirut, Lebanon; [3]Department of Internal Medicine, American University of Beirut Medical Center, Beirut, Lebanon
**Correspondence:** Nada K. Zahreddine
*Antimicrobial Resistance and Infection Control* 2017, **6(Suppl 3):**P212

**Introduction:** CLABSI have been associated with significant morbidity and attributable mortality in patients admitted to Intensive Care Units (ICU).

**Objectives:** The study was conducted to determine CLABSI rates in ICU at the American University of Beirut Medical Center; and to describe the most frequently found causative microorganisms in bloodstream isolates and their resistance profiles.

**Methods:** Infection Control (IC) Preventionists conducted an active and prospective surveillance between January 2007 and February 2017 in a 10-beds ICU using the National Health Safety Network (NHSN) methodology. The CLABSI standardized rates were compared to the international reports of NHSN and the International Nosocomial Infection Control Consortium (INICC). The distribution of microorganisms associated with CLABSI was also analyzed.

**Results:** A total of 96 CLABSI were identified during a 10 years period covering 15765 central line (CL) days. The overall CLABSI rate was 6.1 per 1000 CL days. CLABSI rates were higher than the pooled NHSN rates reflecting hospitals from developed countries and higher than the benchmark from developing countries participating in INICC. Multidrug Resistant Acinetobacter baumannii was most commonly recovered (23.0%) followed by coagulase negative staphylococci (21.0%).

**Conclusion:** The risk of acquiring CLABSI associated with MDR-ACB in ICU patients is high and is strongly correlated with MDR-ACB colonization pressure. Multidisciplinary efforts were adopted throughout the study period to minimize this risk by applying an evidence based CL bundle in addition to introducing active surveillance for MDR-ACB on each patient admitted to ICU. The CLABSI rates were decreasing overtime until a sustained Zero CLABSI rate was reached over the last 8 months of the study period proving the success of the IC interventions.

**Disclosure of Interest**

None Declared

### P213

#### Withdrawn

### P215

#### Just do it! incidence registration of clabsi and cauti may be easier than you think

Mette Walberg
Infection Control Department, Vestre Viken HT, Baerum hospital, Drammen, Norway
*Antimicrobial Resistance and Infection Control* 2017, **6(Suppl 3):**P215

**Introduction:** Hospital leadership need knowledge of nosocomial infection rates for implementing optimum measures. We established incidence registration for central line associated blood stream infection (CLABSI) and catheter-associated urinary tract infection (CAUTI) in chosen units of a Norwegian hospital trust with ca. 250.000 annual bed days. The registration was based on data collected on paper.

**Objectives:** We wanted to know whether prospective incidence registration of CLABSI and CAUTI was possible despite lack of tailored electronic registration tools.
**Methods:** We used CDC criteria and Windows software (Excel). Clinical staff (CS) collected daily data on number of patients with central lines and urinary catheters and emailed scanned copies of results monthly to infection control personnel (ICP). The lists also included necessity for urinary catheter, number of admitted patients (catheter utilization rate) and CAUTI incidents. ICP updated incidence results for CLABSI and CAUTI, forwarded the results to CS monthly, and copied leaders into the email loops. ICP also collected CLABSI incident data. CS and ICP met regularly for data quality control.
**Results:** During one calendar year (2015-2016) we collected data for 1755 central lines in one infection disease unit. Only one CLABSI was found, i.e. an incidence of 0.57 per 1000 central line days. Corresponding numbers registered in an intensive care unit were 488 line days (21 weeks), no CLABSIs observed. In one urology unit we collected data for 652 urinary catheter days (47 weeks) and found 5 CAUTIs, an incidence of 7.7 per 1000 catheter days. Clinical staff in each unit spent 15 – 30 min daily for collection of data, while ICP used about 60 min monthly for collection of CLABSI incidents. ICP spent some hours monthly to update results for CLABSI and CAUTI. 30 min monthly was used for quality control of data from each unit. Before kickoff we spent several hours for project planning and design.
**Conclusion:** We experienced that incidence registration of CLABSI and CAUTI was easier than assumed. Careful planning and sharing of responsibilities between involved personnel was essential. The registrations ran smoothly when CS handled denominator data and numerator data for CLABSI were collected by ICP and CS took care of numerator data for CAUTI. We experienced that our work was vital for patient safety in our hospital trust.
**Disclosure of Interest**
None Declared

### P216

#### Impact of automated surveillance, hand hygiene and preventive interventions on device associated infections at a tertiary care Indian Hospital

Neha Rastogi, Purva Mathur, Alphina Karoung, Jacinta Gunjiyal
Microbiology and Infection Control, JPNATC, AIIMS,New Delhi, New Delhi, India
**Correspondence:** Neha Rastogi
*Antimicrobial Resistance and Infection Control* 2017, **6(Suppl 3):**P216

**Introduction:** Device associated infections (DAI) poses a grave concern to patient outcomes. Systematic surveillance is quintessential to curtail DAIs. However, its implementation especially in developing countries is challenging.
**Objectives:** This study reports the impact of automated surveillance and preventive interventions on DAIs using indigenously developed cost effective software at an Indian Trauma Care Center.
**Methods:** This was a prospective observational study for a period of 3 years. We developed our own software for algorithmic and coherent detection of DAIs based on CDC's NHSN definitions. The rates of DAIs, compliance to hand hygiene and preventive bundles for DAIs were meticulously entered into software and reported as feedback. All cadres of healthcare workers were imparted training on a regular basis. The effect of an intensive surveillance and awareness on the DAI rates, compliance to preventive bundles and hand hygiene were assessed.
**Results:** During the study**,** a total of 7,890 patients were assessed amounting to 39,980 patient days. The rates of VAP, CLABSI and CA-UTI at pre-implementation stage were 8.2, 2.4 and 4.8 /1,000 device days respectively. There was significant correlation between device days and the propensity to develop DAIs (p value = 0.04). In-hospital mortality attributing to infectious complications was 2.6% (n = 208). Rates of VAP, CLABSI and CA-UTI were significantly reduced to 7.07, 2.1 and 3.6/1,000 device days respectively (p value = 0.03). The compliance to hand hygiene increased from 58.9% to 61.3% (p value = 0.6). Increased adherence to preventive bundles for DAI was observed with statistically significant reduction of DAI rates(p value = 0.03).*Acinetobacter baumannii* was the most common pathogen implicated in VAP and CLABSI (37.3%). *Klebsiella pneumoniae* was the most common in CA-UTI (14.7%). Increasing multi-drug resistance was observed among bacterial isolates(63%).
**Conclusion:** Active and systematic surveillance with regular feedback are essential prerequisites to combat complications of DAIs. Stringent surveillance should be considered as a sustainable mechanism to facilitate performance improvement and infection control in hospitals.
**Disclosure of Interest**
None Declared

### P217

#### Investigation of an outbreak of klebsiella oxytoca bloodstream infections on a neonatal population

Seila I. Do Prado, Mayra G. Menegueti, Ana E. R. Lopes, Fabiana M. R. Molina, Gilberto G. Gaspar, Lécio R. Ferreira, Marisa M. Mussi, Lucimara B. Hisamitsu, Fernando Bellissimo-Rodrigues
Ribeirão Preto Medical School, Ribeirão Preto, Brazil
**Correspondence:** Seila I. Do Prado
*Antimicrobial Resistance and Infection Control* 2017, **6(Suppl 3):**P217

**Introduction:** Bloodstream infection (BSI) nosocomial outbreaks are uncommon but potentially life-threating. Most nosocomial outbreaks of BSI have been associated with microbial contamination of infused fluids during manufacturing.
**Objectives:** To describe and investigate an outbreak of BSI caused by *Klebsiella oxytoca* on a neonatal population.
**Methods:** This was a retrospective cohort study performed in a tertiary-care university hospital in October 2016. All neonates admitted during the outbreak period were included and BSI diagnosis was performed based on the Centers for Disease Control and Prevention (CDC) criteria. Selected clinical and demographic characteristics were evaluated on the patient's medical records as potential risk factors for BSI. We used Two-tailed Fisher's exact and Mann-Whitney tests for statistical analysis.
**Results:** Among 35 patients admitted in the study period, 6 (17%) developed a BSI due to *K. oxytoca*. The following variables yielded similar values among those who experienced BSI versus those who did not, respectively: median age (19.5 vs. 24 days, p = 0.895), gestational age at birth (31.5 vs. 34 weeks, p = 0.468), birth weight (1185 vs. 1840 g, p = 0.470), 1-minute Apgar score (6.5 vs. 6, p = 0.580); 5-minutes Apgar score (9 vs. 8, p = 0.497). On the other hand, exposure to central venous catheter was far more common among those who had a BSI versus those who had not (100% vs. 17.2%, p < 0.001), as well as exposure to total parenteral nutrition (100% vs. 34.5%, p = 0.005). After replacement of the parenteral nutrition provider, the outbreak was ended. All affected patients were adequately treated and no death attributable to the infection was observed during the study.
**Conclusion:** From the present investigation, we can state that exposure to central venous catheter and parenteral nutrition were implicated as the main drivers of this *K. oxytoca* BSI outbreak.
**Disclosure of Interest**
None Declared

### P218

#### Infections after patent ductus arteriosus surgical correction procedures in infants with very low birth weight

Katarzyna Kopeć-Godlewska[1], Anna Różańska[2], Jadwiga Wójkowska-Mach[2]
[1]JAGIELLONIAN UNIVERSITY MEDICAL COLLEGE, Kraków, Poland; [2]Chair of Microbiology, JAGIELLONIAN UNIVERSITY MEDICAL COLLEGE, Kraków, Poland
**Correspondence:** Anna Różańska
*Antimicrobial Resistance and Infection Control* 2017, **6(Suppl 3):**P218

**Introduction:** Very low birth weight (VLBW) neonates <1500 grams, comprise approx. 1% of liveborn infants in Poland. Owing to premature rupture of membranes, these children are born immature, with such structures as patent ductus arteriosus (PDA). PDA results in many complications that could compromise the health and survival of the neonates. In some of them it is necessary to use surgical methods to surgically close the PDA.

**Objectives:** The aim of the study was to see how many VLBW neonates treated in the participating wards needed surgical correction of PDA and also to analyse the incidence risk of various types of post-surgical infections together with their etiological factors.

**Methods:** Continuous prospective monitoring of infections was done in the years 2009-2013 in 6 neonatology departments. The studied group was made up of VLBW neonates that needed surgical correction of PDA. Infections were detected based on criteria according to Gastmeier.

**Results:** 2039 VLBW neonates were included in this study. PDA that needed surgical intervention was confirmed in 103 newborns (5.1%), significantly more frequently in children from multiple pregnancy and labour complicated by amnionitis. The surgical PDA correction was performed on average in the 19th day of life of the newborns. Infection was registered in 48.5% (n = 50) of neonates: 27 developed sepsisis (incidence 26.2%) and 23 pneumonia (incidence 22.3%). A correlation was observed between the day the procedure was performed and the time of infection: the earlier the newborn was operated on, the earlier was the infection manifested (p = 0.032). High CRIB score and amnionitis in the mother were significant risk factors for the infection.

**Conclusion:** Infections incidence after surgical correction of PDA in VLBW neonates is reported at a similar level to infections in all hospitalized VLBW neonates. The later the PDA surgery was done, the later the infections manifested.

None Declared

### P219

#### Identification and control of klebsiella oxytoca outbreak in the neonatal intensive care unit

Sheroline P. Primus, on behalf of Infection Control Nurse, Simone Keizer-Beache,Chief Medical Officer (Ministry of Health wellness and the Environment)

[1]Infection Prevention and Control Department, Milton Cato Memorial Hospital, Kingstown ST.Vincent, Kingstown, Saint Vincent and the Grenadines

**Correspondence:** Sheroline P. Primus

*Antimicrobial Resistance and Infection Control* 2017, **6(Suppl 3):**P219

**Introduction:** An outbreak of klebsiella oxytoca occurred in the Neonatal Intensive Care Unit (NICU) in January, 2015.

**Objectives:** This study sought to identify the source of this outbreak and to contain the nosocomial spread.

**Methods:** An investigation was launched in response to the isolation of klebsiella oxytoca in the 8 bed (NICU), with one death. An Infection Control Nurse Team conducted a document review and collected demographics, birth weight, clinical manifestations, diagnoses, antibiotic use, date of admission and reports of initial blood cultures. The NICU staff was interviewed using an unstructured questionnaire. Infection prevention and control practices were monitored with emphasis on hand hygiene, cleaning, disinfection and maintenance of the liquid soap dispenser. An environmental assessment was later conducted. Swabs were taken from the hands of five nurses and three doctors, reconstituted intravenous antibiotics, water for injection, liquid antimicrobial soap, stethescopes, cotton swabs, linen and incubators. The Clinical Microbiology Laboratory database was searched for additional cases of klebsiella oxytoca blood stream infections. Staff on the Paediatric ward/(NICU) participated in educational sessions on the prevention of infections. Adherence to previously issued guidelines was reinforced and a cleaning schedule was outlined.

**Results:** Admission blood cultures showed no bacterial growth. Klebsiella oxytoca was isolated from blood cultures of four neonates taken between January $6^{th}$ and $26^{th}$, 2015 with two resultant deaths. The Microbiology Laboratory database revealed two additional cases of Klebsiella oxytoca. klebsiella oxytoca was isolated from a liquid soap dispenser. This contaminated dispenser was discarded and replaced with single-use Chlorhexidine Gluconate 4% Antiseptic Solution. Follow-up observation indicated compliance with practices. Repeat blood cultures showed no bacterial growth.

**Conclusion:** The outbreak of klebsiella oxytoca bloodstream infections was associated with insufficient cleaning and disinfection practices of the liquid soap dispenser. Health care workers must understand how practices and procedures can contribute to infections. Knowledge and adherence to guidelines can avert similar outbreaks.

**Disclosure of Interest**

None Declared

### P220

#### Influence of coagulase negative staphylococci in blood culture on catheter-related infections diagnosis

Jérôme Ory, Magali Vidal, Natacha Mrozek, Olivier Lesens, Claire Aumeran

Service Maladies infectieuses et tropicales, CHU Clermont-Ferrand, Clermont-Ferrand, France

**Correspondence:** Jérôme Ory

*Antimicrobial Resistance and Infection Control* 2017, **6(Suppl 3):**P220

**Introduction:** Coagulase negative *staphylococci (CNS)* are the main pathogens isolated from central lines blood cultures and are responsible for most catheter-related infections.

**Objectives:** The objective of this study was to determine the clinical significance of *CNS* isolated from central line blood cultures.

**Methods:** CNS positive central lines blood cultures were prospectively collected over a 5-month period in a university hospital. Peripheral blood cultures, clinical and biological data were gathered. Using these data, 4 groups were defined: bacteraemia, probable bacteraemia, catheter colonization (CC) and blood culture contamination (BCC). Each group was described by Charlson score, antibiotic therapy and complications.

**Results:** From 680 positive central line blood cultures, 129 were positive with CNS. CNS was responsible for 28 bacteraemia, 9 probable bacteraemia and 92 CC or BCC. Our criteria did not allow us to differentiate CC or BCC.

Most CNS positive central lines were sampled in haematology ward (n = 58) and ICU (n = 30). CC or BCC were more common in haematology (n = 43) than in ICU (n = 24).

Mean length of stay for patients with bacteraemia, probable bacteraemia and CC/BCC was 24, 30 and 21 days respectively. Mean Charlson score was 4.3, 1.75 and 3.1 respectively. 95% of central lines were removed for bacteraemia or probable bacteraemia and 62% for CC or BCC. The rate of complication was low (n = 4) with local abscess (n = 1), severe sepsis (n = 1), admission in ICU (n = 1) and death (n = 1). Empiric antibiotic therapy was prescribed in 17 bacteraemia, 9 probable bacteraemia and 11 CC or BCC.

**Conclusion:** Most of the positive CNS central line blood cultures were CC or BCC, and have been mainly found in the haematology ward. These results could be affected by the blood culture sampling technique, in particular asepsis. Audit of practices will be necessary to confirm this hypothesis. Improving the blood culture technique may decreased the contamination rate and unnecessary antibiotic therapy.

**Disclosure of Interest**

None Declared

### P221

#### Implementation of aseptic non touch technique for procedure iv preparation and administration, iv cannulation and blood sampling to reduce hais rate at premier Jatinegara Hospital

Rosdelima Simarmata[1], Siti indriani[2]
[1]Infection Control Committee, Premier Jatinegara Hospital; [2]Infection Control, Hospital, East Jakarta, Indonesia
**Correspondence:** Rosdelima Simarmata
*Antimicrobial Resistance and Infection Control* 2017, **6(Suppl 3):**P221

**Introduction:** As an International organization that accredited by Joint Commission International (JCI) Accreditation, Premier Jatinegara Hospital always perform a risk management approach to improve quality of care that focused in reducing and preventing the Health Care Associated Infections (HAIs). The approach in order to reduce HAIs is by implementing Aseptic Non Touch Technique (ANTT) that focus on specific clinical procedure such as IV cannulation, IV preparation and administration and blood sampling as a pilot project.
**Objectives:** The purpose of this study is to standardize and Implementing practice base on the steps of aseptic non touch techniques for specific procedure and prevent the occurrence of phlebitis and Bloodstream infections (BSI) due to the inconsistency of the implementation proper aseptic technique.
**Methods:** Observation and audit cycle at selected clinical care as a pilot project. Process implementation divided into 3 they are Pre ANTT, Audit Cycle and Post ANTT.
**Results:** There was increase compliance practice of ANTT for IV cannulation 96%, IV preparation and administration 96% and blood sampling 94%, also zero incidence of phlebitis and decrease incidence of BSI 0.002%.
There was increase compliance practice of ANTT for IV cannulation 96%, IV preparation and administration 96% and blood sampling 94%, also zero incidence of phlebitis and decrease incidence of BSI 0.002%.
**Conclusion:** Increasing compliance of ANTT practice for specific clinical procedures, also decreasingincidence of BSI and zero phlebitis during period study.

Disclosure of Interest
None Declared

### P222

#### Withdrawn

### P223

#### Predisposing factors in the formation of biofilms in indwelling biliary stents

Chetana Vaishnavi, Jayanta Samanta, Prashant Kapoor, Rakesh Kochhar
Gastroenterology, Post Graduate Institute of Medical Education and Research, Chandigarh, India
**Correspondence:** Chetana Vaishnavi
*Antimicrobial Resistance and Infection Control* 2017, **6(Suppl 3):**P223

**Introduction:** Biliary plastic stents are a landmark achievement in the field of endoscopic retrograde cholangiopancreatography (ERCP) for the relief of obstructed biliary system by a non-surgical approach. The limiting factors for these plastic stents are their diameter and the tendency to get occluded. Factors involved in the formation of biofilms are not well studied.
**Objectives:** We quantified the biofilm mass and its principal constituents in biliary stents, investigated the bacteria in biofilm formation and analyzed its relation to the predisposing factors.
**Methods:** Demographic and clinical details of 81 patients with biliary stent retrieval were noted. Stents were processed for biofilm mass quantification, protein and polysaccharide estimation and molecular identification of bacteria involved. Correlation of biofilm formation to predisposing factors was also analyzed.
**Results:** The underlying causes for stent insertion were benign stricture (35.8%), bile duct stones (56.8%) and malignancy (7.4%) with cholangitis in 61.7% patients. The retrieved stent size was 7 Fr (76.5%) and 10 Fr (23.5%) with 65 days median insertion duration. Polybacterial consortia were isolated in 82.5% of the stents. The mean biofilm mass was 0.187 ± 0.18 OD, protein 0.50 ± 0.25 mg/mL and polysaccharide 0.051 ± 0.018 mg/mL and were higher in stents of patients with cholangitis. Protein was significantly lower in the 10 Fr stents compared to 7 Fr ones. Biofilm mass was higher ($p = 0.04$) in stents with indwelling time of ≥3 months and >6 months. Factors such as etiology of stenting and presence of single versus multiple microorganisms were insignificant.
**Conclusion:** Our study quantified biofilm mass, protein and polysaccharide in occluded biliary stents and demonstrated polybacterial profile. Cholangitis and smaller diameter stents depicted higher protein concentration in the biofilms predisposing to early biofilm formation. Longer stent indwelling time was associated with higher biofilm mass and protein concentration elucidating the time-dependent biofilm formation process.
**Disclosure of Interest**
None Declared

### P224

#### Increasing awareness of hospital acquired blood stream infections through self-investigation by hospital wards

Pnina Shitrit, Hanni lederman, Beatriz Gerber, Mira Maram
Meir medical center, Kfar Saba, Israel
**Correspondence:** Hanni Lederman
*Antimicrobial Resistance and Infection Control* 2017, **6(Suppl 3):**P224

**Introduction:** Blood stream infection (BSI) leads to morbidity and mortality. Most data regarding hospital-acquired BSI are from device-associated modules in intensive care units (ICU). Data on hospital wide acquisition is lacking.
**Objectives:** Our intervention aimed to assess the magnitude of the problem hospital- wide and to increase awareness of the healthcare teams in the wards through guided self-investigation.
**Methods:** Meir Medical Center is a 740-bed secondary hospital with about 66,000 admissions annually. Beginning January 2016, acquired BSI events were sent to the relevant departments daily with requests to investigate the event using a structured questionnaire. BSI was defined as hospital-acquired if blood culture was drawn on or after the third day of admission, if the patient was discharged within 7 days of the current BSI, or if BSI was related to a procedure in the last 30 days. Results were returned to the infection control team for analysis. Immediate feedback regarding quality of data and investigation results was given as needed. BSI clusters or events related to specific procedures initiated immediate investigation and intervention by the infection control team. A summary of the results was sent to the wards and to hospital management quarterly.
**Results:** A total of 970 BSI events occurred in 2016; 247 (24.5%) were hospital-acquired (3.72/1000 admissions). of these, 210 (85%) originated out of ICU. The wards completed 60% of the investigations. The most common sources were urinary tract infection (33%), central and peripheral IV lines (29%), and pneumonia (14%). Twenty-nine (19.5%) patients were transferred to ICU and 53 died in hospital (35.6%). Following investigation results, procedures such as peripheral IV insertion and indications for urinary catheter were reviewed by the wards. Two clusters related to specific procedures (prostate biopsy and cardiac device implants) were identified and led to immediate intervention.

**Conclusion:** Hospital Acquired BSI is common. Self-investigation by the wards led to increased awareness and efforts to reduce high-risk processes by wards and by hospital management. Ongoing investigations led to timely identification of clusters. Longer follow-up is required to assess decrease in BSI acquisition.

**Disclosure of Interest**

None Declared

### P225

#### Medical staff implication and use of sulfadiazine coated central catheter in cardiac surgery: a five years prospective, observational study

Arnaud de la Chapelle[1], Alain Mihoubi[2], Pierre Dellamonica[3]
[1]Anesthesiology; [2]Cardiology; [3]Infectious Disease, institut Arnault Tzanck, Saint Laurent du Var, France
**Correspondence:** Arnaud de la Chapelle
*Antimicrobial Resistance and Infection Control* 2017, **6(Suppl 3):**P225

**Introduction:** In cardiovascular surgery, bacterial colonization of the central venous catheter (CVC) line has been incriminated as a cause of endocarditis.

**Objectives:** In order to reduce this colonization, after a 5 years observational prospective study(2006-2010), we implemented in a prospective study two new measures consisting in the implication of all the praticians of the department (surgeons, anesthesiologists and cardiologists) to reduce CVC exposure time and the implantation of silver chlorexidine sulfadiazine coated catheter.

**Methods:** From January 2011 to December 2016, all patients hospitalized in our department with CVC were included and compared to the first period (2006-2010). The following data were collected: sign of systemic infection, microbiology, demography and adverse effects of therapy. The statistical data were processed using Systat 11

**Results:** 5019 catheters were implanted between 2006 and 2016; 128 catheters were colonized. The implantation rate was similar between the 2 periods: 557 by year+/-5 vs. 550 by year +/- 7 ($p = 0.1$). The patients were older during the second period 71+/-10 vs 69+/-10 $p = 0.03$. The others demographics data were similar between the two periods. The average rate of CVC colonization was reduced from 3.9+/-0.5% per year to 0.99+/-0.2% ($p < 0.01$). The level of catheter-related bacteremia (CLABSI) was similar 4.38+/-0.3‰ Vs 4.08+/-0.2‰ or 0.87 / 1000 catheter days Vs 0.89/ 1000 catheter days ($p = 0.4$). Catheter's dwell time was reduced: 5+/-7 days VS 4+/-4 days ($p = 0.032$). The extra cost of coated catheter was 2864 euros/year but we reduced additional days of hospitalization from 88 days/ year to 4 /year.

**Conclusion:** This study demonstrates that the implication of all medical staff reduced the catheter dwell time. Both measures divided by 4.5 the rate of catheter colonization. Reducing catheter colonization rate did not reduce CLABSI probably due to a low CLABSI rate during observational period.

**Disclosure of Interest**

None Declared

### P226

#### Reduction of methicillin resistant staphylococcus aureus bacteraemias in haemodialysis patients following the the kubler-ross change cycle

Claire Farrugia, Michael Borg
Infection prevention and control unit, MATER DEI HOSPITAL, MSIDA, Malta
**Correspondence:** Claire Farrugia
*Antimicrobial Resistance and Infection Control* 2017, **6(Suppl 3):**P226

**Introduction:** A high prevalence of MRSA bacteraemia was identified in dialysis patients attending the Renal Unit of Mater Dei Hospital, Malta. In 2010, this was estimated at 6 cases/100 patients/ year. Root cause analyses (RCA) showed they occurred primarily in haemodialysis (HD) patients with non-tunnelled haemodialysis catheters (NTHC).

**Objectives:** To reduce the MRSA bacteraemia in HD patients.

**Methods:** The need for improvement was evident but most healthcare professionals (HCPs) were passive and their self efficacy perception was low. An incremental approach was adopted using Kubler-Ross stages of grief. Initially some HCWs were shocked and claimed various reasons why their HD population is more prone to infection. The first recommendation implemented was the monthly MRSA screening for all HD patients, 11.7% of patients were found to be colonized and decolonization attempted. Change was facilitated by appointing a link nurse while the infection control unit (ICU) was crucial in facilitating communication with vascular surgery and medical imaging to change work processes. Anger was expressed through outpouring of bottled up frustrations. Throughout the bargaining stage, standard operating procedures (SOPs) were drafted, new products procured and educational material prepared. Gradually more HCPs accepted and supported the changes as the MRSA bacteraemias reduced. Kubler-Ross refers to this passive stage as the depression phase during which change is intergrated. Care bundles for insertion and maintenance of HD lines were introduced. Educational events and integration of these practices marked the acceptance phase and ownership of infection prevention in the HD population that these HCPs care for.

**Results:** Despite an increase in the haemodialysis population, MRSA incidence reduced significantly and most HCPs at renal unit are now promoting infection prevention measurues.

**Conclusion:** Understanding the Kubler-Ross change cycle and the journey these HCPs were going through enabled the ICU to accompany them and keep them focused. A new CEO and nursing director have also impacted this change process. The key factor in this success was providing a strategy, supporting different HCPs and empowering key frontline staff who together with motivated vascular surgeons and radiologists drove the improvement.

**Disclosure of Interest**

None Declared

### P227

#### CR-SBI targeting zero regional program

Giuseppe Murolo
Dipartimento Attività Sanitarie e Osservatorio Epidemiologico, Assessorato Salute - Regione Sicilia, catania, Italy
*Antimicrobial Resistance and Infection Control* 2017, **6(Suppl 3):**P227

**Introduction:** Illustrate the implementation of a Sicily's Risk Management program called 'CATETHER RELATED BLOOD STREAM INFECIONS -TARGETING ZERO'. In many Hospitals both culture and compliance to international guidelines were missing. The main goal of the Program was to lead the health care Professional teams to a new promotional approach: the plan was to reach a higher target with the intent of developing a new culture and prevention protocols: not only nurses and clinicians but Regional Institutions, Risk Managers, Hospital General Managers, and also Caregivers.

**Objectives:** Some clinicians in a specific centers where adopting Bundle to prevent infections as Catheter Related Blood Stream Infections (CR-BSI) but most were not, and there was no mandatory reason for them to. The final goal was to facilitate networking within the Region decision makers in order to define a common protocol for prevention and make it applicable everywhere in compliance with the International Giudelines.

**Methods:** The Regional Institution took action and decided for the first time to make prevention protocol mandatory. A Specific Regional Decree was formalized (June 2016) where the Hospital General Managers are told to be responsible for their hospital's alignment to international guidelines through the adoption of the Insertion and Exit-site management Vascular Access Bundles. After 6 month from the Regional Decree (Spring 2017) we expect a Regional HAI (Healthcare Associated Infections) prevalence survey data on prevention impact.

**Results:** all the Hospitals (public and private) decided to have a VASCULAR ACCESS TEAM, dedicated Educational Programs to increase knowledge, delineation of "privileges" for all personnel involved in the insertion and maintenance of intravascular catheters, adopting a

Regional Vascular data Sheet, Periodic Audit to assess adherence to guidelines. They are aligned at John Hopkins's Quality and Safety Research model to implement a Good Practices Called '4E' ENGAGE, EDUCATE, EXECUTE, EVALUATE.
**Conclusion:** the results suggests that "Targeting Zero" is an ambitious goal but it can be achieved through a specific and dedicated policy providing for the implementation of a multifaceted program and the involvement of all actors at different institutional levels of the system (regional administrators, CEOs of healthcare organizations and frontline professionals).
**Disclosure of Interest**
None Declared

#### P227b
**Microbiology analysis of catheter related bloodstream infections in critical care patients of a Costa Rican pediatric third level hospital**
Tatiana Barrantes Solís, Milena Arias Jiménez, Marlen Villegas Sánchez, Jorge Delgado Arguedas, Marcela Hernández de Mezerville
Hospital Nacional de Niños, Dr. Carlos Sáenz Herrera (HNN), San Jose, Costa Rica
**Correspondence:** Tatiana Barrantes Solís
*Antimicrobial Resistance and Infection Control* 2017, **6(Suppl 3):**P227b

**Introduction:** Catheter-related bloodstream infection (CRBSI) case definition requires at least one positive blood culture not related to infection in another body site. There are 3 main situations when central line contamination can occur: during insertion, at daily manipulation or by contamination of administered fluids.
**Objectives:** To describe the microbiology of the central line infections in the intensive care Units of the "Hospital Nacional de Niños, Dr. Carlos Sáenz Herrera" (HNN), the only pediatric referral center of Costa Rica.
**Methods:** Descriptive study of blood cultures of patients under 13 years old, hospitalized at the Neonatal Intensive Care Unit and the Pediatric Intensive Care Unit at HNN, who had a central line and developed a bloodstream infection between January 1st, 2013 and December 31st, 2015.
**Results:** We analyzed 113 positive blood cultures, 104 with only one germ and seven with 2 or more, for a total of 123 identifications. 46% were Gram-negative bacteria, 43% Gram-positive and 11% fungi. Main pathogens were *E.coli*, *K. pneumoniae* and *S. epidermidis*, as well as *Candida spp*. 20% were extensively drug-resistant bacteria and 2% were pandrug-resistant. In children who had more than one CRBSI, 33% were with the same pathogen. Median time from central line insertion to positive blood culture was 20.3 days.
**Conclusion:** Unlike this study, most report Gram-positive bacteria as the main pathogen in CRBSI, secondary to skin colonization during central line insertion. The predominance of Gram-negative bacteria and the time elapsed between insertion and sepsis manifestation suggest catheter manipulation as the possible source. The high proportion of extensive-resistant bacteria and yeast infections alerts about the increase of multidrug-resistant organisms and the need to improve the rational use antibiotics.
**Disclosure of Interest**
None Declared

### Surgical site infection: Surveillance

#### P228
**Public reporting used as a driver for quality improvement: the New Zealand surgical site infection improvement programme**
Nikki Grae, Sally Roberts, Deborah Jowitt, Arthur Morris, on behalf of Infection Prevention & Control Programme
New Zealand Health Quality & Safety Commission, Wellington, New Zealand
**Correspondence:** Nikki Grae
*Antimicrobial Resistance and Infection Control* 2017, **6(Suppl 3):**P228

**Introduction:** Public reporting has been a feature of the New Zealand Surgical Site Infection Improvement (SSII) Programme since 2013. The SSII Programme focuses on reducing SSI following hip and knee arthroplasty and cardiac procedures through a bundle of interventions (clipping not shaving the surgical site, alcohol-based skin prep, and timely administration of the appropriate antibiotics in the right dose). Quality and Safety Markers (QSM) were developed to monitor improvement in the process.
**Objectives:** The aim of the SSII Programme is to improve the standard of care by measuring compliance with these interventions. Public reporting of the QSM performance was seen as a driver for practice change and supported continuous quality improvement initiatives at the local level.
**Methods:** Standardised process and outcome data is recorded on all hip and knee arthroplasty and cardiac procedures funded by District Health Boards (DHB) which equate to approximately 12,500 procedures annually. The QSM process measures focus on the correct use of surgical antimicrobial prophylaxis (SAP) and use of alcohol-based skin prep and the outcome measure is the SSI rate. QSM targets were established to set the expected levels of performance. Reporting of each DHB performance against the QSM targets occurs quarterly and is available on a public-facing website.
**Results:** There has been a significant aggregated improvement in QSM performance between July 2013 and September 2016.
**Conclusion:** Transparent public reporting of each DHB performance against the QSM targets supported alignment and coordination of quality improvement activities nationally. Multidisciplinary teams with strong clinical engagement from individual DHBs implemented quality improvement initiatives based on their specific opportunities and systems. The initiatives and key learnings were then shared at regional and national meetings so other DHB SSII Programme teams could also benefit. The SSII Programme has resulted in significant improvement in QSM performance and most procedures now comply with recommended best practice.
**Disclosure of Interest**
None Declared

#### P229
**Surveillance of surgical site infections: an emergency in Saint John of God regional hospital of Northern in Republic of Benin**
Semevo Romaric Tobome[1,2], Thierry K. Hessou[1], Adrien M. Hodonou[3], Robert Akpata[4], Roberto Caronna[2], Alexandre S. Allodé[3], Alassan S. Boukari[1], Ulrich Parfait Otchoun[1], Roméo Haoudou[1], Giambattista Priuli[1]
[1]Saint John of God Hospital, TANGUIETA, Benin; [2]Sapienza University, Roma, Italy; [3]University of Parakou, Parakou, Benin; [4]French National Institute of Health and Medical Research (Inserm), Conakry, Guinea
**Correspondence:** Semevo Romaric Tobome
*Antimicrobial Resistance and Infection Control* 2017, **6(Suppl 3):**P229

**Introduction:** Surgical site infections (SSI), are seen as a reflection of the healthcare quality in hospitals
**Objectives:** To study the SSI at Saint John of God Hospital Tanguiéta (SJGHT) as a prelude to the implementation of a permanent monitoring system
**Methods:** It was a descriptive and prospective study in the Department of General Surgery of SJGHT from 1 July 2016 to 31 January 2017. It admits patients 15 years of age or older. The SJGHT haven't a microbiology unit. The study population consisted of all patients admitted in it, after a surgical operation from July to December 2016. For each patient surgical wound was monitored for one month. The diagnostic criteria used are those for the monitoring of the SSIs (WHO/2002.12. Prévention des infections nosocomiales 2e édition). Data was recorded and analyzed using Epi info 7. Statistical tests were used accordingly, p less than 0.05 considered as statistically significant
**Results:** For 343 patients opered, 105 (30.6%) had SSI. The mean age of the patients with SSI was 40.3 years. The sex ratio was 4. Emergency surgery resulted in a 50.0% rate of SSI ($p = 0.00$). The most common surgical intervention was laparotomy (26.7%). American Society of Anesthesiologists (ASA) score was good (1 or 2) in 77 patients out of 301 and poor (3 or more) in 30 out of 42; $p = 0.00$. 112 were operated on clean or clean-contaminated surgery and 6.1% presented SSI, 231 patients had been operated on infected contaminated or contaminated surgery, of whom 81.3% presented SSI ($p = 0.00$). For 35 patients the

NNIS was 2 and all of them presented SSI; NNIS was 1 for 105 patients, of whom 66.7% presented SSI; none of the remaining 203 patients with NNIS0 developed SSI (p = 0.00). It was: superficial 13.3%; deep 46.7%; organ/space 40.0%. The mean time prior to occurrence of SSI was 4.2 days. The hospital stay duration of patients with SSI was three times longer than the duration of patients without SSI (p = 0.00)

**Conclusion:** Implementation of a simplified SSI monitoring system is therefore an emergency in SJGHT

**Disclosure of Interest**

S. R. Tobome Employee of: none, Grant/Research support from: none, Speaker's bureau of: none, Shareholder of: none, Consultant for: none, Paid instructor for: none, Other conflict with: none, T. Hessou Employee of: none, Grant/Research support from: none, Speaker's bureau of: none, Shareholder of: none, Consultant for: none, Paid instructor for: none, Other conflict with: none, A. Hodonou Employee of: none, Grant/Research support from: none, Speaker's bureau of: none, Shareholder of: none, Consultant for: none, Paid instructor for: none, Other conflict with: none, R. Akpata Employee of: none, Grant/Research support from: none, Speaker's bureau of: none, Shareholder of: none, Consultant for: none, Paid instructor for: none, Other conflict with: none, R. Caronna Employee of: none, Grant/Research support from: none, Speaker's bureau of: none, Shareholder of: none, Consultant for: none, Paid instructor for: none, Other conflict with: none, A. Allodé Employee of: none, Grant/Research support from: none, Speaker's bureau of: none, Shareholder of: none, Consultant for: none, Paid instructor for: none, Other conflict with: none, A. Boukari Employee of: none, Grant/Research support from: none, Speaker's bureau of: none, Shareholder of: none, Consultant for: none, Paid instructor for: none, Other conflict with: none, U. P. Otchoun Employee of: none, Grant/Research support from: none, Speaker's bureau of: none, Shareholder of: none, Consultant for: none, Paid instructor for: none, Other conflict with: none, R. Haoudou Employee of: none, Grant/Research support from: none, Speaker's bureau of: none, Shareholder of: none, Consultant for: none, Paid instructor for: none, Other conflict with: none, G. Priuli Employee of: none, Grant/Research support from: none, Speaker's bureau of: none, Shareholder of: none, Consultant for: none, Paid instructor for: none, Other conflict with: none

### P230

#### Point prevalence and surveillance of healthcare associated infections in surgical wards of a University-Affiliated Hospital in Mashhad, Iran

Mohammad Hassan Aelami[1], Ali Jangjoo[2], Hamid Reza Naderi[2], Nasrin Khosravi Zenyani[2], Irandokht Mostafavi[2], Haleh Amirian[2]
[1]Pediatrics; [2]Mashhad University of Medical Sciences, Mashhad, Iran, Islamic Republic Of
**Correspondence:** Mohammad Hassan Aelami
*Antimicrobial Resistance and Infection Control* 2017, **6(Suppl 3):**P230

**Introduction:** Healthcare- associated infections (HAIs) are a major problem for patient safety.

**Objectives:** Its surveillance and prevention must be the first priority in each hospital.

**Methods:** The study was a point prevalence survey measured at two points (16Dec2012 and 8 Jan 2014) in Imam Reza hospital; Mashhad,Iran. All patients were followed by call till one month for surgical site infections using CDC criteria. All patients admitted more than 24 hours were included. Questionnaires with demographic and clinical characteristics were filled by the physicians.

**Results:** During the 2 point prevalence surveys, a total of 23 patients with HAIs were identified among 329 patients (6.9%). A total of 29 HAIs were reported which the most frequent HAIs were surgical site infections (SSIs; 3.6%), symptomatic urinary tract infections (SUTI; 1.8%), ventilator-associated pneumonia (VAP; 1.5%) and blood stream infections (BSI; 0.9%). Prevalence of HAIs was high in cardiothoracic surgery (40% in 2012) and ICU (38.4% in 2014). After 30-day surveillance for surgical site infections, we could follow 191 patients (58.2%) which, 6 of them (3.1%) developed surgical site infections and 7 of them (3.6%) expired. In 2012, having a urinary catheter was associated with symptomatic urinary tract infections and in 2014, having a central venous catheter was associated with blood stream infections.

**Conclusion:** Prevalence of HAIs in surgical wards in our hospital was relatively low and maybe underreported.

**Disclosure of Interest**

None Declared

### P231

#### Identification of isolated germs in surgical site infections in caesarean parturients at the Chu of Béni Messous, Alger- Algerie

Ghania Brahimi, A. L. Rebouh, S. Ait Seddik, S. Amrit, H. Ould Baba Ali, D. Toauti, Z. Bouchene, R. Belkaid
[1]CHU de Béni Messous, ALGER, Algeria
**Correspondence:** Ghania Brahimi
*Antimicrobial Resistance and Infection Control* 2017, **6(Suppl 3):**P231

**Introduction:** The patient's endogenous source is mostly involved in the occurrence of surgical site infections[1] (SSI). Exogenous contamination is less frequent.

**Objectives:** - Determine the incidence of SSI in Caesarean parturient
- Identify the causative germs.

**Methods:** This is a prospective cohort study with real-time data collection, including Caesarean parturients from 01 February to 30 May 2014 and 2015. The follow-up with phone calls was provided up to +30 days. In case of suspicion of an SSI, samples for cytobacteriological examination of the liquid coming from the superficial or deep part of the incision have been realized. The data was entered and evaluated using Epi info6 software.

**Results:** The incidence rate in 2014 is 16.72% (52/311) and 4.7% (14/300) in 2015. Third generation cephalosporins were used in 87.2% (472/541) for antibiotic prophylaxis. The SSI occurred after the exit of the parturients in 72.7% of the cases, 42 (63.6%) cases benefited cytobacteriological analysis from purulent secretions. The examination returned positive in 43% (18/42) of the cases, 57% (24/42) were decapitated (sampling after an average delay of antibiotic therapy of 6.9 ± 2.8 days). 20 germs were isolated, predominantly monomicrobial (88.9%), 65% were Gram-positive cocci: staphylococcus aureus accounted for 45% of the isolated germs including MRSA followed by Streptococcus agalactiae in 20%, no strain of staphylococcal coagulase has been found. Gram-negative bacilli represent 35%: 71.4% are enterobacteriaceae, 40% of which are extended-spectrum beta-lactamase (ESBL), Escherichia coli was isolated in 10%, the same for pseudomonas aeruginosa (without any resistance).

**Conclusion:** The endogenous flora is responsible for the majority of documented SSI, whether through poor skin preparation or aseptic error, but the emergence of ESBL is linked to the use of broad-spectrum antibiotics. This requires clinical, microbiological and therapeutic vigilance in view of their specific resistance profile to antibiotics.

[1]Astagneau
P, L'Hériteau F, Daniel F, et al. Reducing surgical site infection incidence through a network: results from the French ISO-RAISINsurveillance system. J Hosp Infect. 2009 Jun;72(2):127–34.

Disclosure of Interest

None Declared

### P232

#### Incidence of surgical site infection in postoperative patients at a tertiary care hospital in Tunisia

Sihem Ben Fredj, Hela Ghali, Mohamed Ben Rejeb, Salwa Khefacha, Houyem Said latiri
Prevention and Security of Care, University Hospital Of Sahloul, Faculty of Medecine of Sousse, Sousse, Tunisia
**Correspondence:** Sihem Ben Fredj
*Antimicrobial Resistance and Infection Control* 2017, **6(Suppl 3):**P232

**Introduction:** Surgical Site Infections (SSIs) are among the most harmful complications that occur after surgery. It has been generally found that developed nations tend to have lower incidence of SSIs when compared with developing nations. Therefore, reducing SSI occurrence is a public health priority and it requires the implementation of infection control programs which need a thorough knowledge of the SSI rate and risk factors of this complication. In fact, effective surveillance systems have been described as a preventive measure for reducing SSI.

**Objectives:** to determine the SSI incidence and epidemiology among surgical patients in a tertiary care setting in Sousse, Tunisia.

**Methods:** A prospective observational study was carried out from January 2015 to May 2015 in the General Surgery Department at the University Hospital of Sahloul in Sousse, Tunisia. During that period, all patients who underwent general surgical procedures and matching the inclusion criteria were included. Patient data were collected using a form built on the French national protocol for the SSI surveillance of ISO-RAISAN and on data from the literature. Regular follow-up was maintained until at least 30 days postoperatively.

**Results:** A total number of **365** patients were included. The mean age was **44.4 ± 20.7** years. The majority of patients (**96.1%**) had a score *ASA* of one or two. Interventions duration was greater than the 75$^{th}$percentile in 39 cases (**10.7%**). Among the 349 cases (96%) followed to 30 days after operation, 30 SSI have been diagnosed, with an incidence rate of **8.6%.** The incidence density was 12.9 cases for 1000 days of hospitalization. Nearly **40%** of patients developed a SSI during the first week and **90%** before the end of the 3$^{rd}$ week after the surgery. Furthermore, the global cost caused by SSI was estimated to 42709.96 Tunisian dinars.

**Conclusion:** The incidence of SSIs was found to be within the upper international limits. Therefore, more efficient programs are needed to decrease the SSIs rate since this complication increases hospitalization costs and length of stay, and impairs patient's quality of life. Hence, among means of prevention, aside from identification and control of known risk factors, ongoing surveillance has proven to be an independent factor for long term reduction of SSI rates.

**Disclosure of Interest**

None Declared

### P233

#### Results of a two-year surveillance of surgical site infections in thoracic surgery unit

Grzegorz Dubiel[1], Paweł Rogoziński[1], Krzysztof Buliński[1], Elżbieta Żaloudik[1], Anna Różańska[2], Jadwiga Wójkowska-Mach[2]

[1]Department of Thoracic Surgery, Pulmonology and Thoracic Surgery in Bystra, Bystra; [2]Chair of Microbiology, JAGIELLONIAN UNIVERSITY MEDICAL COLLEGE, Kraków, Poland

**Correspondence:** Anna Różańska

*Antimicrobial Resistance and Infection Control* 2017, **6(Suppl 3):**P233

**Introduction:** SSI is considered to be a priority in infection control.

**Objectives:** The objective of this study is the analysis of results of active targeted surveillance conducted over the period of 2 years in the Department of Thoracic Surgery at the Pulmonology and Thoracic Surgery Center in Bystra, in south of Poland.

**Methods:** The retrospective analysis was carried out on the basis of results of active monitoring of SSI in the 45-bed thoracic surgery unit, in the period from 1 April 2014 to 30 April 2016. Surgical site infections were identified based on the definitions of the European Centre for Disease Prevention and Control (ECDC) taking into account the time of symptom onset: if the symptoms occurred in the time of 30 days following the surgical procedure. Detection of SSI relied on daily inspection of wounds by a trained nurse, analysis of medical and nursing entries in the computer system and analysis of all results of microbiological tests taken in the unit and in the operating room.

**Results:** In the analyzed period, data were collected as regards 1387 treatment procedures Conference the registration criteria. 40 SSI cases were detected, incidence rate - 3%. procedures. Most cases -55% were found in the course of hospitalization and 45% were detected after the patient's discharge. 37.5% cases of SSI were classified as superficial infections, 7.5% as deep infection and 55% as organ space infection. Among patients who were diagnosed with SSI, most were men 77.5%. For patients with ASA score I-II incidence rate was 2%, ASA score III or more – 3.7%. The incidence rate varied from 0.3% in clean surgical site to 6.5% in clean-contaminated site.

**Conclusion:** The study validated the usefulness of targeted surveillance in monitoring surgical site infections in patients hospitalized in thoracic surgery department. SSI surveillance allowed to identify areas of care demanding modifications, which are organization of post-discharge and microbiological diagnostics of infection cases.

**Disclosure of Interest**

None Declared

### P234

#### The lucina project: an observational study of post-cesarean section surgical site infections in Kenya

Angie Sway[1], Joseph Solomkin[2]

[1]Oasis Global; [2]University of Cincinnati College of Medicine, Cincinnati, United States

**Correspondence:** Joseph Solomkin

*Antimicrobial Resistance and Infection Control* 2017, **6(Suppl 3):**P234

**Introduction:** Sepsis is the leading cause of maternal death in Sub-Saharan Africa (SSA), a region that sees some of the highest rates of maternal mortality and morbidity in the world. As one of the most commonly performed surgical procedures in SSA and a proven risk factor for surgical site infection (SSI), cesarean section (CS) is an important operation to target due to its massive impact on maternal and neonatal health. There is currently insufficient published data available on the patient and facility based context around SSI following CS to establish a true and clear understanding of this infectious category.

**Objectives:** The objective of this study was to collect accurate and valid data on the incidence of SSI following CS and the circumstances around SSI in two Kenyan hospitals. Our primary focus was on the consequences of timing of perioperative antimicrobial prophylaxis.

**Methods:** This was an IRB-approved observational study of six hundred and nine women who had CS operations at two Kenyan hospitals from September to December 2015. Hospital A provided antimicrobial prophylaxis prior to incision for all patients and Hospital B provided only post-operative prophylaxis to all patients.

**Results:** The median age was 26 ± 6 (18, 43) at Hospital A and 26 ± 5 (18, 44) at Hospital B. Median parity was 1 ± 1 (0, 7) at Hospital A and 1 ± 1 (0, 10). Patients also went through a comparable number of antenatal care visits (median = 4 ± 1 at both hospitals). The number of patients with prolonged rupture of membranes was 103 (34.4%) at Hospital A and 99 (32.9%) at Hospital B. There were a slightly higher number of patients with meconium stained liquor at Hospital B Hospital (115) than Hospital A (74). The SSI rate was 4.0% (12/299; 11 superficial SSI, 1 deep SSI) at Hospital A and 9.3% (28/301; 18 superficial SSI, 7 deep SSI, 3 organ/space SSI) at Hospital B.

**Conclusion:** The data shows a striking difference between SSI rates in patients who were given properly timed pre-operative antibiotics and patients who were only given post-operative antibiotics. Administration of post-operative antibiotics is currently the norm in much of SSA and there is strong evidence that many of the infectious problems encountered in this population would be reduced by the provision of antibiotic prophylaxis prior to the incision.

**Disclosure of Interest**

None Declared

### P235

#### Eight-year decline in incidence of surgical site infection following craniotomy through prospective surveillance in a tertiary hospital in Korea

Eun Jin Kim[1], Nam Joong Kim[1, 2], Pyoeng Gyun Choe[1, 2], Ji Seon Kim[1], Bo Ram Oh[1], Seung Ki Kim[3], Won Sang Cho[3], Yun Suhk Suh[4]

[1]Infection control center; [2]Department of internal Medicine; [3]Department of Neurosurgery; [4]Department of Surgery, Seoul National University Hospital, Seoul, Korea, Republic Of

**Correspondence:** Eun Jin Kim

*Antimicrobial Resistance and Infection Control* 2017, **6(Suppl 3):**P235

**Introduction:** Surgical site infections (SSI) after neurosurgery have serious clinical consequences and increase costs. Surveillance of SSI with feedback of appropriate data to surgeons has been known to be an important component of strategies to reduce SSI risk.
**Objectives:** This study conducted to examine the effect for 8 year surveillance and feedback program of neuro surgery in a tertiary hospital in Korea.
**Methods:** Patients who underwent craniotomy (included "CRAN" category from CDC/NHSN) from 2009 to 2016 were prospectively monitored for SSI up to 30 days after surgery or 90 days after implant insertion. SSIs were defined according to CDC/NHSN surveillance definition.
A confirmed case of SSI was notified to the surgeon and periodically feedback was given to the neuro surgery department. SSI rates over the 8 year were analyzed using the basic linear regression.
**Results:** A total of 6,572 CRAN category procedures performed on 6,137 patients were monitored. SSIs occurred in 138 cases, giving overall rate of SSI of 2.10%. The majority (67.4% [93/138]) of SSIs was organ or space SSI; only 11.6% and 21.0% were superficial-incisional and deep-incisional SSIs, respectively.
The incidence of SSI(crude rate) were significantly reduced over the 8 years (from 3.65% in 2009 to 0.70% in 2016, P = 0.047). The duration cut point (75 percentile of the duration of surgery in minutes) also significantly decreased over the 8 years (from 353 min in 2009 to 273 min in 2016, P = 0.002).
**Conclusion:** The prospective surveillance and subsequent feedback program were effective for reducing the rates of SSIs after Neuro surgery.
**Disclosure of Interest**
None Declared

### P236
### Prevention of surgical site infections (SSI) in cesarean sections: a clinical audit in the Gaza-Strip
Mohamedraed S. Elshami[1], Enas F. Alaloul[1], Sireen M. Alattar[2], AbdelKarim F. Alrekeb[3], Rania H. Sarsour[3], Iman H. Sarsour[3], Bettina Bottcher[1]
[1]Faculty of Medicine, ISLAMIC UNIVERSITY; [2]Obstetrics Department, Altahreer Hospital; [3]Obstetrics Department, Al-Helal El-Emirati Hospital, Gaza, Palestinian Territory, Occupied
**Correspondence:** Mohamedraed S. Elshami
*Antimicrobial Resistance and Infection Control* 2017, **6(Suppl 3):**P236

**Introduction:** Feedback of surveillance results regarding intraoperative measures to prevent surgical site infections (SSI) to obstetricians has been shown to be an important component of strategies to reduce SSI risk. This is the first clinical audit in Gaza-Strip to observe the application of those measures in cesarean sections (CS).
**Objectives:** To evaluate adherence of obstetricians to preventive intra-operative measures of SSI.
**Methods:** A prospective clinical audit was conducted in the operating rooms (ORs) of two obstetric Gaza-Strip hospitals (50 CS were taken from each one) from January to March 2017. Practice was compared to the standards of WHO guidelines (2016). The data was analyzed using SPSS program.
**Results:** In total, 100 CS were observed. The mean age was 30 years ± 6 and the mean duration of operations was 40 minutes ± 15. About 18% were emergency CS. Around 17% did not have antibiotic prophylaxis. Shaving was done in 34% of CS. Surprisingly, surgical scrubbing and cleaning of skin before incision were done with povidone-iodine solution in all cases, which is not recommended for this use. However, double gloving and change of gloves were practiced in 18% of CS, for which there is no specific recommendation. The use of warming devices and 80% fraction of inspired oxygen ($FiO_2$) are recommended to reduce SSI, but were only found in 27.5% and 46% respectively. Although recommended, irrigation of incisional wounds before closure with an aqueous povidone-iodine solution was only done in 7.5% of cases.
**Conclusion:** This audit shows a poor adherence to the WHO guidelines for the preventive intra-operative measures of SSI. Some of this might be due to a lack of local guidelines for SSI prevention, others to a lack of regular training and awareness among obstetricians. Thus, creating and employing strict infection control policies by a functional infection control committee should be considered urgently as well as promoting awareness and knowledge of evidence-based practice among obstetricians.
**Disclosure of Interest**
None Declared

### P237
**Withdrawn**

### P238
### Audit on the cutaneus preparation of the operated patient in surgical services of Beni Messous University Hospital Center Algiers 2017
A. L. Rebouh, G. Brahimi, S. Ait Seddik, A. Larinouna, H. Khellaf, N. Cheboub, W. Djouimaa, A. EL Kechai, A. Chetitah, D. Chenini, F. Boudebouz, R. Belkaid
[1]CHU de Béni Messous, ALGER, Algeria
**Correspondence:** G. Brahimi
*Antimicrobial Resistance and Infection Control* 2017, **6(Suppl 3):**P238

**Introduction:** Several studies have shown that a good preoperative cutaneous preparation (PCP) significantly reduced the cutaneous microbial flora.
**Objectives:** To describe the PCP practices, measure the gap with recognized recommendations, and propose a tool to put in place the necessary corrective measures.
**Methods:** Internal audit by observation of the nursing staff and interview of patients, related to the practices of the PCP in the services of general surgery (GS), ophthalmology (OPHT) and infantile surgery (IS) from 22 January to 2 February 2017 (3 working days per service). Data entry and analysis were carried out on Epi-Data.
**Results:** The audit involved 39 patients who underwent cold surgery. PCP information was given to 43.6% (17/39) patients; 79.5% (31) performed preoperative shower at home within 24 hours (77.4% the day before, 25.8% on surgery day and 3.2% both days). Among them 66.7% (16) received the prescription for antiseptic soap. The PCP checks weren't performed in the operating room (OR) in 94.8% of cases. The cleaning phase was not carried out (100%) by non-availability of the product, mucosal disinfection was done in the OR in all cases. Polyvidone iodine (PVI) was used for disinfection in 92.1%. In the OPHT service, the 10% PVI is diluted with saline solution before being applied to the ophthalmic mucosa in 87.5%, the unidose presentation is non-existent. There are no protocols displayed for the surgical field and no traceability of the PCO in the patient record.
**Conclusion:** Oral and written information about the PCP must be provided preoperatively by the surgeon or anesthesiologist. The availability of appropriate products for detersion and single-dose presentation for the ophtalmology department, will reduce the risk of infection associated with care. The surgical fields must be the subject of a written protocol and validated by the CLIN.
**Disclosure of Interest**
None Declared

### P239
### Surveillance of surgical site infections at the ENT service of Chu Beni Messous of Algiers in 2014
G. Brahimi[1], A. L. REBOUH [1], I. ARAREM[1], R. BELKAID[1], GHANIA S. HARAOUBIA[2]
[1]CHU de Béni Messous; [2]CHU ISSAD HASSANI, commune de Béni Messous, Alger, CHU, ALGER, Algeria
**Correspondence:** G. Brahimi
*Antimicrobial Resistance and Infection Control* 2017, **6(Suppl 3):**P239

**Introduction:** Surveillance of surgical site infections (SSI) is a priority in our facility. In the ENT (ORL) service, the SSI incidence has significantly decreased since it joined the active surveillance network in 2006.

**Objectives:** - To calculate the incidence rate of SSI- To identify the risk factors associated with the occurrence of SSI
**Methods:** Descriptive longitudinal study for analytical purposes. Data collection was carried out from 1st February to the 30th May 2014 with a follow-up up to 30 days. The SSI diagnosis was based on the CDC (Atlanta) criteria. The analysis of the data was carried out on the EPI-INFO6.04 software.
**Results:** A total of 326 interventions were included. The sex ratio was 0.93, the mean age was 34.9 +/- 18 years. 85.1% had an ASA score = 1, the average length of stay was 4.4 +/- 0.2 days. The percentage of patients reviewed on D30 was 93.5%. Two thirds of the interventions were clean-contaminated interventions, and half had a NNIS = 0 score ; no emergency intervention was performed, 14 patients developed SSI an average of 4 +/- 0.4 days following surgery. The overall SSI incidence was 4.3% (95% CI = [2.1 - 6.5] ; mean age of infected patients was 44+/-17.2 years.The incidence rate was 41.7% for the ASA score 2 versus 2.9% for the ASA 1 score, and the incidence of infections was significantly greater when the Altemeier class was contaminated (8.5% versus 2.6%, p <0.01). Stratified according to NNIS, the incidence rate varies from 3% for NNIS interventions 0 to 8% for NNIS interventions >1 (p < 0.001).
**Conclusion:** The monitoring of SSI in ENT has highlighted risk factors that should be taken into account in order to improve the management and prevention of risks related to surgical care. However, the improvement of preventive measures must be pursued by influencing extrinsic risk factors.
**Disclosure of Interest**
None Declared

### P240
### Surgical site infections among patients operated at Katutura State Hospital, Khomas Region, Namibia
Augustine Kastherody, Amagulu Nelao, Emmy Munenguni, Martina Kanandura
Ministry of Health and Social Services, Windhoek, Namibia
**Correspondence:** Augustine Kastherody
*Antimicrobial Resistance and Infection Control* 2017, **6(Suppl 3):**P240

**Introduction:** The study was conducted at Intermediate Hospital Katutura which has a bed capacity of 896 and is located in Windhoek, Namibia. The hospital has a total of 9 major operating rooms which covers general surgery, orthopedics, obstetrics and gynecology.
**Methods:** Files of patients who underwent general surgical operations between January 2006 and December 2014 were retrospectively reviewed to determine the incidence of surgical site infections (SSI). Random sampling was used to identify patient files for review. The criteria used to determine SSI was; temperature >390C, pus on the surgical site, positive culture results.
**Results:** A total of 79,385 patients underwent surgical operations in the defined period. Of these 52.7% were males (n = 41,815). A random sample of 800 files (1%) was retrieved to assess whether the patients had SSI or not. A total of 100 files were found to have had SSI which gives the incidence of 12.5%.The commonest procedure that was associated with SSI was laparotomy Organisms isolated according to the laboratory results from pus swabs and blood culture were; enterococcus, MRSA, pseudomonas and E. Coli
**Conclusion:** The incidence of SSI is high at Katutura hospital. There is therefore a need to promote infection prevention and control practices to reduce SSI.
**Disclosure of Interest**
None Declared

## Urinary tract infections

### P241
### Investigation of urovirulence agents in escherichia coli causing uriner tract infections
Cansu Önlen, Toğrul Nağiyev, Suna Kızılyıldırım, Fatih Köksal
Medical Microbiology, Medicine Faculty, Cukurova University, Adana, Turkey
**Correspondence:** Toğrul Nağiyev
*Antimicrobial Resistance and Infection Control* 2017, **6(Suppl 3):**P241

**Introduction:** Extraintestinal pathogenic *E.coli* (ExPEC) causes prominent of uriner tract infection (UTI). *Escherichia coli* (*E.coli*) may progress further via the ureters to the kidneys and cause pyelonephrit. Also, the bacteria damage kidney mucosa epitel to enter lymphatic system and cause bacteremia. Virulence factors making ExPEC different from other *E.coli* strains are the host cell adhesion, invasion, and two important factors/toxin, Cytotoxic Necrotizing Factor (CNF) and Cytolethal Distending Toxin (CDT) that are responsible for cell death.
**Objectives:** Detection of factors/toxin genotypes are taught to have predictive value for surveillance and the prognosis of infections. The aim of the present study was to detect the prevalences of urovirulence agents in *E.coli* strains that cause UTI in women and men and to compare in women-men urovirulence agents.
**Methods:** Samples were provided from the regional hospital in Adana province and identified by phenotypically IMVIC, genotypically for presence of the gene encoding the enzyme β-glucurinidase (*uidA*) and investigated by PCR to identify CNF1-CNF3 and CDT1-CDT4 and hemolysis (*hlyA*) genes by using specific primers.
**Results:** In 2 years period, 522 *E.coli* strains were recovered form urine from men (n = 80) patients and women (n = 442) patients with urinary tract complaints in different units. At least one virulence gene identified for *E.coli* was found in 108 (16.7%) of 522 *E.coli* strains. Respectivly; 70, 14, 13 of ExPEC strains contained *cnf1*, *cnf2*, *cnf3*. Genes *cdt1*, *cdt2*, *cdt3, cdt4* were detected 4, 2, 3, 2. These 108 strains were found hemolysis expression. Strains from women patients were more often displayed expression with urovirulence agents %72 than from men (%28).
**Conclusion:** Urovirulence agents and UTI are seen more frequently in women patients because of shorter urethra than men and that CNF gene family is believed to enhance colonization of *E.coli* in the urinary system and these genes of the *E.coli* gene pool are gained in the ability to survive in new environment, such as the human urinary tract. The widespread diffusion of the *cnf1* gene in *E.coli* help to distinguish ExPEC from commensal strains and these genes may be use spesific marker for noncommensal *E.coli*. It is thought that CNF antitoxin can be used in vaccination studies to prevent *E.coli* which causing UTI.
**Disclosure of Interest**
None Declared

### P242
### Effect of implementation of catheter associated urinary tract infection(CAUTI) bundle checklist in acutely ill patients(ICUS)
Asmaa Hagag
Infection prevention and control, Security forces hospital makkah, Makkah, Saudi Arabia
*Antimicrobial Resistance and Infection Control* 2017, **6(Suppl 3):**P242

**Introduction:** CAUTI account for 40% of all healthcare associated infections. A multidisciplinary team formulate and implement urinary catheter bundle checklist, as Data were obtained then compliance is analyzed and compared to the reduction in CAUTI incidence rate\1000 catheter days
**Objectives:** decrease the CAUTI incidence rate among ICU patient
**Methods:** Applied in26beds of ICU patients as14AICU,5PICU,and7-NICU beds,CAUTI surveillance was performed every month according to CDC-NHSN criteria with interpretation,it was noticed that there is increase in the CAUTI incidence rate compared to the international NHSN benchmark which is ranging from (5-5,5)per 1000 catheter days**Pilot phase**:CAUTI bundle checklist initiated as **8 items1**-documented indication**2**-hand wash and wear gloves when handling urinary catheter**3**-routine perineal care**4**-maintain catheter securment**5**-urinary bag less than2\3full and emptied prior to transport**6**-closed system is maintained**7**-drainage bag attached to the side of bed and below bladder level and**8**-not touch the floor **then started to educate** ICU staff for one month on how to implement.The list is completed for every cathetered patient allover the week for data validation then compliance calculation,**Implementation**:started by june-2016 as the CAUTI incidence rate was 5.9,then 6.2\1000

catheter days in July, but it was noticed increase in the incidence rate to 12.6 in August so,IPC team start to initiate the bundle checklist and did training,orientation during September, then measuring the compliance compared to the drop ofCAUTI incidence rate to 4.8 when the compliance was 95% at the end of September then starts to increase again to7.7 with drop in the compliance to89% in October,then the better compliance was in November 97% with decrease in CAUTI incidence rate to 4.5 (accepted range)

**Results:** There was a reduction in the rate of CAUTI in ICU, from 12.6 per1,000 catheter-days before the intervention to 4.5 per 1,000 catheter-days after the intervention

**Conclusion:** CAUTI bundle implementation is effective in reduction of CAUTI incidence/1000 catheter days of Acutely ill patients

1-Preventing
catheter-associated urinary tract infection in the zero-tolerance eraArticle in American journal of infection control · June 2011
2-GUIDELINE
FOR PREVENTION OF CATHETER-ASSOCIATED URINARY TRACT INFECTIONS 2009(HICPAC)

Disclosure of Interest

None Declared

### P243

#### Study on the decononisation of urinary tract catheters applaying a practice-like in vitro test method

Florian H. H. Brill[1], Steffen Pahl[1], Jan-Hendrik Klock[1], Henrik Gabriel[1], Holger Brill[1], Julia Hambach[1], Carmela Jänicke[1], Andreas Arndt[2]
[1]Dr. Brill + Partner Gmbh Institute for Hygiene and Microbiology, Hamburg, Germany; [2]B. Braun Medical AG, Sempach, Switzerland
**Correspondence:** Steffen Pahl
*Antimicrobial Resistance and Infection Control* 2017, **6(Suppl 3):**P243

**Introduction:** Urinary infections are among the most common nosocomial infections, accounting for 23.2% of them. In 80% of cases, these infections are catheterassociated urinary tract infections (CAUTI). The pathogens attach themselves to the catheter walls within a biofilm. Long-term use of urinary catheters is therefore a risk factor for developing a CAUTI. Changing the catheters more frequently would remove the biofilm, but is unpleasant for the patient and timeconsuming for personnel. Regular rinsing of the catheter can be a useful way to remove the emerging biofilm, and to prevent the creation of biofilm in the first place and potential subsequent blockages of the catheter. However, this is not yet standard clinical practice.

**Objectives:** The influence of rinsing the catheters should be tested *in vitro* in order to examine its operating principle.

**Methods:** In a practice-like *in vitro* test method the catheters were contaminated with *Escherichia coli*, *Proteus mirabilis* or methicillin-resistant *Staphylococcus aureus* for 72 hours. Afterwards, the catheters were rinsed with 0.02% Polihexanide (PHMB), 0.9% NaCl or were not treated (positive control) and the viable cell count was determined. The biofilm mass was quantified in a staining assay. In addition, fluorescence microscopy assays were performed in a flow cell to visualize the vitality of the cells after rinsing with PHMB or NaCl.

**Results:** Rinsing the catheters with Polihexanid singnificantly reduced the cell count ($p = 0.012$). The reduction factor compared to "non-treatment" was $2.56 \pm 0.67$ log. In comparison to NaCl, rinsing with Polihexanide results in significantly lower cell counts ($p = 0.034$), higher reduction rates in biofilm mass and is able to kill off the cells at the surface of the biofilm.

**Conclusion:** Rinsing the catheters reduces colonization of catheters by microorganisms. Polihexanide-rinsing is more effective compared to rinsing with saline solution. A reduction in biomass of the biofilm was demonstrated both by fluorescence microscopy and via the *in vitro*-model. Further studies need to investigate wheter the presented results can be transferred into practice and actually lead to a reduction in urinary tract infections.

**Disclosure of Interest**

F. Brill Grant/Research support from: B. Braun Medical AG, S. Pahl: None Declared, J.-H. Klock Grant/Research support from: B. Braun Medical AG, H. Gabriel Grant/Research support from: B. Braun Medical AG, H. Brill Grant/Research support from: B. Braun Medical AG, J. Hambach Grant/Research support from: B. Braun Medical AG, C. Jänicke Grant/Research support from: B. Braun Medical AG, A. Arndt Employee of: B. Braun Medical AG

### P244

#### Care bundles implementation: from theory to practice

David Peres[1,2], Fernanda Vieira[1], Ilda Devesa[1], Alexandra Fernandes[1], Isabel Neves[1,3,4] on behalf of Local Team of "STOP Hospital Infection" Project
[1]Infection and Antibiotic Resistance Control Unit, Matosinhos Local Health Unit, Matosinhos; [2]Medical Internship Programme, São João Hospital Center, Porto; [3]Infectious Diseases Unit, Matosinhos Local Health Unit, Matosinhos; [4]National Coordination of Prevention and Control of Infection and Antimicrobial Resistance Program, Directorate General of Health, Lisbon, Portugal
**Correspondence:** David Peres
*Antimicrobial Resistance and Infection Control* 2017, **6(Suppl 3):**P244

**Introduction:** In last decade implementation of care bundles became gradually generalized in several health units around the world. As many reports conclude, these set of four or five processes, when performed together for every patient every time, have the potential to improve patient outcome and in prevention of healthcare-associated infections (HAI's).

**Objectives:** To describe the implementation and monitorization of a bundle in an acute care hospital.

**Methods:** In 2015, a collaboration between Calouste Gulbenkian Foundation and Institute for Healthcare Improvement, established a challenge to Portuguese hospitals: to reduce in 50% the HAI over a period of 3 years. A methodology based on quality collaborative was implemented, emphasizing mutual learning, motivating and empowering teams using measurable and achievable targets, as well as planning sustainable improvements, with the involvement of management. Pedro Hispano Hospital, a 370 bed acute care unit, was one of the 12 portuguese hospitals to be selected for this project.

**Results:** For this project the following steps were put in place: (1) problem definition (e.g. many urinary infections in catheterized patients); (2) baseline measurement (e.g. incidence study to determine the proportion of catheter urinary tract infection - CAUTI) ; (3) determination of objectives to achieve (e.g. process measure: bundle of urinary catheter insertion/maintenance fulfilled > 95%; outcome measure: reduction of 50% of number of CAUTI per 1000 patients catheterized); (4) design/ adaption of the bundles using the Deming cicle methodology (e.g. poor registry of urinary catheter indication of use, following changes in software to remind professionals to do this) and (5) monitoring through run charts (e.g. improvement of fulfillment of bundle and reduction in CAUTI rate). Difficulties detected: work overload of health professionals with little time for training or meetings; non-adequacy of some clinical material (e.g. amount of gel of urinary catheter kits, quality of urinary collectors).

**Conclusion:** Implementation of care bundles can be an important driver for the health professionals to deliver evidence-based care. They should be part of an organizational cultural change, with focus on sustainable improvements.

**Disclosure of Interest**

None Declared

### Methicillin-resistant *Staphylococcus aureus* (MRSA) and vancomycin-resistant *enterococci* (VRE)

#### P245

**High level aminoglycoside resistant enterococci in hospital-acquired urinary tract infections**

Rasha H. El-Mahdy, Ahmed M. Mostafa
Medical Microbiology and Immunology department, Faculty of Medicine, Mansoura University, Mansoura, Egypt
**Correspondence:** Ahmed M. Mostafa
*Antimicrobial Resistance and Infection Control* 2017, **6(Suppl 3):**P245

**Introduction:** Enterococci are increasing in resistance to various antimicrobial agents and effective treatment represents a major health concern. The aim of this study was to determine the antimicrobial resistance pattern of enterococci and the frequency of vancomycin and high-level aminoglycoside resistance among them.
**Objectives:** to determine the antimicrobial resistance pattern of enterococci and to determine the frequency of vancomycin and high-level aminoglycoside resistance.
**Methods:** Methods: A total of 80 isolates of enterococci, (73 *Enterococcus faecalis,* 7 *Enterococcus faecium*) were collected from patients with hospital-acquired urinary tract infections in Mansoura University hospitals. Antimicrobial susceptibility testing was done by disc diffusion method. Identification of species and detection of vancomycin and aminoglycoside-modifying enzymes genes AME were done by PCR.
**Results:** All isolates were sensitive to vancomycin and linezolid. Fifty-three isolates were high-level aminoglycoside resistance (HLAR). aac(6′)-Ie-aph(2″)-Ia gene was associated with *aph(3*′)-IIIa and ant(6)-Ia gene in 69% of HLAR isolates.
**Conclusion:** Our results show increasing frequency of HLAR among hospital-acquired Enterococci and revealed that aph(3′)-IIIa and ant(6)-Ia were the most common aminoglycoside modifying enzymes genes detected.
**Disclosure of Interest**
None Declared

#### P246

**Control of hospital-acquired vancomycin resistant enterococci (VRE) colonisation: a descriptive study**

Li Jie, Qiu Sha Meng, Maria Theresa Cabahug, Ying Wang, Thean Yen Tan
Infection Prevention and Control, Changi General Hospital, Singapore, Singapore
**Correspondence:** Li Jie
*Antimicrobial Resistance and Infection Control* 2017, **6(Suppl 3):**P246

**Introduction:** Vancomycin-resistant enterococci are nosocomial pathogens, and transmission within health-care institutions is well-documented. Increasing acquisition of VRE was seen in our institution since Jan 2014.
**Objectives:** This observational study monitored VRE acquisition and clinical infections following sequential implementation of control measures, and describes the impact on laboratory costs and clinical infections.
**Methods:** Increased hospital-acquired VRE (HO-VRE) colonisation was detected in early 2014. Increased VRE surveillance was implemented which included VRE contacts, patients with length of stay > 7 days, patients with a recent history of hospitalisation in other hospitals, and renal dialysis patients. The following interventions were implemented sequentially: (1) cohorting of VRE patients on individual wards (2) creation of a VRE-specific cohort ward (3) emphasis on hand hygiene and contact precautions (4) enhanced environmental and equipment cleaning (5) weekly feedback on ward-level VRE acquisition. The numbers of VRE screening tests was derived from the laboratory, and the number of clinical infections was tracked by infection control nurses.
**Results:** HO-VRE colonisation rate was 13.7/10,000 patient days in 2014, reached a nadir of 2.8 in 2015, and plateaued at 7.6 for 2016. During the 3 year period, there were at least 4 clusters of VRE cross-transmission. The number of VRE screening tests performed was 37,368, and 9 clinical infections were reported for the three years, respectively. The cost of VRE screening was 1.8 million Singapore dollars for three years. The annual aggregated hospital-wide hand hygiene compliance for the three years was 54%, 60% and 65% respectively.
**Conclusion:** Control of VRE remains extremely challenging in hospital setting with multi-bedded open ward environment. The implementation of a multiple intervention program showed reduction in VRE acquisition, but continuing clusters of transmission. There was a significant increase in laboratory costs. Despite ongoing HO-VRE transmission, clinical infections remain low.
**Disclosure of Interest**
None Declared

#### P247

**Controlling a vancomycin-resistant enterococci outbreak IN A TUNISIAN TEACHING HOSPITAL**

Héla Ghali, Sihem Ben Fredj, Mohamed Ben Rejeb, Salwa Khefacha, Houyem Said Latiri
Department of Prevention and Security of Care, Sahloul-Sousse, Tunisia
**Correspondence:** Héla Ghali
*Antimicrobial Resistance and Infection Control* 2017, **6(Suppl 3):**P247

**Introduction:** Antimicrobial resistance is a growing threat to public health and motivates to improve prevention and control programs. Despite their low pathogenicity, *vancomycin-resistant enterococci* (VRE) are nosocomial pathogens in several countries.
The high potential for transmission of VRE between patients justify strict control measures. Indeed, VRE represents the second cause of urinary tract and wound infections and the third cause of nosocomial bacteremia in the United States.
**Objectives:** An epidemic of VRE has affected our hospital and the objective of this work is to describe the measures put in place.
**Methods:** Following the alert given by the service of plastic surgery concerning a patient carrier of VRE, a team of the prevention and healthcare security service made an investigation. A review of files was conducted to draw the synoptic table and the table of cases.
**Results:** By contacting the microbiology laboratory, we have identified four other cases of VRE and who were hospitalized in Medical resuscitation department (2 cases, one of them was transferred to the Physical rehabilitation department), and Nephrology department (2 cases). The visit has allowed to detect several malfunctions in professional practice. A crisis cell has allowed to validate, coordinate and implement control measures following the recommendations of the Technical Center of nosocomial infections. In fact, the process was to technically isolate cases in their sector of hospitalization, to restrict the use of antibiotics, to strength measures of basic hygiene, and to make a screening by rectal swab for both cases and contacts (other patients and health staff).
These measures have helped to control the situation and no other case has been reported.
However, these are short-term strategies, and other measures in the medium and long term should be taken into account in order to face similar outbreaks.
**Conclusion:** The efforts to control the outbreak were efficient since no new cases have been reported. However, a continuous monitoring in order to detect new cases earlier is crucial to minimize the dissemination of VRE.
**Disclosure of Interest**
None Declared

#### P248

**Whole genome sequencing highlighted two small st117 vancomycin-resistant enterocccus outbreaks within a large st80 outbreak**

Estelle Moulin[1], Laurence Senn[2], Dominique Blanc[2], Mohamed Abdelbary[2], Gregory Chaillou[2], Nicolas Demartines[2], Gilbert Greub[2]
[1]Infection Control; [2]CHUV, LAUSANNE UNIVERSITY HOSPITAL, Lausanne, Switzerland
**Correspondence:** Estelle Moulin
*Antimicrobial Resistance and Infection Control* 2017, **6(Suppl 3):**P248

**Introduction:** Despite low incidence of vancomycin-resistant *Enterococcus* (VRE) in Switzerland (0.3-2%), the Lausanne University Hospital experienced several VRE epidemic events in medical and surgical units since 2011.
**Objectives:** We used prospective whole genome sequencing (WGS) to investigate the last VRE outbreak (*E. faecium vanA*) that occurred in a 60-beds surgical ward.
**Methods:** Systematic screening for VRE (rectal swabs) was performed in all patients hospitalized in the epidemic ward on a weekly basis. Selective culture plates were used to isolate VRE. One isolate per patient was generally further analysed by whole genome sequencing (WGS) using MiSeq (Illumina). Isolates' sequence type (ST) was assigned from short reads data. Genome alignment of all isolates was acquired with Snippy by mapping against a reference. A maximum likelihood tree was constructed based on the number of "core genome single nucleotide polymorphisms" (SNPs). Contact precautions in isolation zones were applied in VRE cases and contacts, and environment disinfection was reinforced.
**Results:** Following the identification of the first case, the weekly screening revealed 41 additional VRE positive patients between October 2016 and January 2017. Isolates from 35/42 (83%) patients harboured *vanA* gene cluster. Among the latter, 29 belonged to ST80 (26 highly related), five to ST117 (four with a low genetic diversity suggesting transmission), and one strain was not sequenced. All the seven *van B* isolates belonged to ST117 and WGS revealed that four were highly related. Thus, one major and two minor VRE lineages circulated in the same ward over the same period of time.
**Conclusion:** This study highlights the co-circulation of different VRE lineages inside the same ward. Combining epidemiological investigations with WGS analysis of the isolates enables to differentiate clusters of cases and allows better understanding and management of this kind of nosocomial outbreaks.
**Disclosure of Interest**
None Declared

### P249

#### Emergence of linezolid resistance in enterococcus species in a tertiary acute care centre

Smita Sarma[1], Shalini Upadhyay[1], Yatin Mehta[2]
[1]Microbiology and Infection Control; [2]Medanta- The Medicity, GURGAON, India
**Correspondence:** Smita Sarma
*Antimicrobial Resistance and Infection Control* 2017, **6(Suppl 3):**P249

**Introduction:** Isolation of linezolid resistant enterococcus (LRE) in urine samples has increased in our hospital recently. This is alarming as treatment options are limited.
**Objectives:** We tried to identify the risk factors associated with linezolid resistance, correlate linezolid consumption with increased isolation and evaluate fosfomycin as a therapeutic option
**Methods:** This was a prospective observational study for 6 months. Cases were defined as inpatients growing LRE in urine culture. Controls were inpatients whose urine culture grew linezolid sensitive enterococcus (LSE). The specimen were plated onto culture media and incubated at 37 °C for 24-48 hrs. Identification of the isolates was done by both phenotypic and automated method. Antimicrobial susceptibility testing was determined by using the Vitek-2 automated system. Linezolid resistant strains were tested for fosfomycin sensitivity. Clinical details of the cases and controls were recorded including history of catheterisation, prior exposure to linezolid and colonisation with multidrug resistant organisms
**Results:** A total of 25 cases and controls were enrolled in our study. The risk factors identified in the case group were diabetes, urinary catheterisation, increased duration of hospitalisation, past exposure to linezolid and immunocompromised state. Demographically the cases and control patients did not differ significantly, but cases were hospitalized significantly longer than controls. Urinary catheterization was significantly higher in cases then in controls. Prior isolation of vancomycin resistant enterococcus (VRE) was more common in cases than controls and 60% of the cases were colonised with LRE at the time of isolation of the urinary isolates. All the enterococcus isolates were sensitive to fosfomycin
**Conclusion:** Fosfomycin may be considered for treating LRE urinary tract infections. Although the overall linezolid consumption in the hospital showed an increasing trend, there was no prior exposure to linezolid in 92% of the cases. We suggest identifying the mechanism for linezolid resistance as resistance may not only emerge during treatment, but a possibility for horizontal transmission may also be looked into. Sequencing of our isolates would throw some light in identifying the mechanism of resistance
**Disclosure of Interest**
None Declared

### P250

#### Knowledge of transmission based precautions among south Indian health care workers- a report

Dorairajan Sureshkumar[1], Jothimani Hemalatha[2], Soujanya Saravanakumar[3]
[1]infectious Disease, Madras Medical Mission; [2]100 Degree F, Chennai; [3]KMC, Manipal, Mangalore, India
**Correspondence:** Jothimani Hemalatha
*Antimicrobial Resistance and Infection Control* 2017, **6(Suppl 3):**P250

**Introduction:** Standard and transmission-based precautions are essential to control and to prevent the nosocomial spread of multidrug resistant organisms (MDROs in the hospitals. A lack of knowledge regarding transmission based precautions among health care workers (HCW) reduces compliance with these practices.
**Objectives:** The objective of the study was to assess the knowledge of transmission based precautions among health care workers (HCW) in South India.
**Methods:** This cross sectional study was conducted among HCW working in different hospitals across South India selected through purposive sampling using a structured questionnaire regarding their knowledge about transmission based precautions. Content validity of the questionnaire was established via the feedback of 10 infection control personnel working in the accredited hospitals in South India. The questionnaire contains details regarding transmission based precautions for common MDROs like methicillin-resistant *Staphylococcus aureus* (MRSA),vancomycin resistant *Enterococci* (VRE), carbapenem resistant *Enterobacteriaceae* and *Clostridium difficle* (CDI). Survey questionnaire completed by consenting respondents through face-to-face interaction were taken for analysis. Data were compiled using descriptive statistics
**Results:** Totally 125 HCWs participated in the survey. Only 28% (35/125) HCWs know correctly the all three (contact, droplet and airborne) transmission based precautions. The other parameters surveyed were shown below in table -1

**Table 1 Transmission based precautions Knowledge among HCWs in South India**

| Organism | No of HCW with correct answer/ Total HCW | Percentage |
|---|---|---|
| MRSA | 73/125 | 58.4% |
| VRE | 48/125 | 38.4% |
| CRE | 37/125 | 29.6% |
| CDI | 34/125 | 27.2% |

**Conclusion:** The majority of South Indian HCWs lack knowledge on transmission based precautions. Among the MDROs, the HCWs had better knowledge for MRSA transmissionprevention. More structured educational programs are needed for HCWs to improve their knowledge on transmission based precautions in South India.

**Disclosure of Interest**
None Declared

### P251
### Prevention of staphylococcus aureus bacteraemia in haemodialysis patients
Madelaine Holland[1], Itisha Gupta[2], Tamasin Stevenson[3], Jyoti Baharani[3]
[1]Infection Prevention and Control; [2]Department of Microbiolgy, Birmingham Public Health Lab Public Health England; [3]Renal Medicine, Heart of England NHS Founation Trust, East Birmingham, United Kingdom
**Correspondence:** Madelaine Holland
*Antimicrobial Resistance and Infection Control* 2017, **6(Suppl 3):**P251

**Introduction:** *Staphylococcus aureus* (SA) is a common cause of bacteraemia (SAB) in patients with chronic haemodialysis.These patients are at risk of intermittent or persistent colonisation which increases the risk of vascular access infection and bacteraemia.
**Objectives:** Reducing colonisation and vascular access infection is the key to prevent SAB and we present our strategies to minimise these.
**Methods:** The Heart of England Foundation Trust has 4 haemodialysis units and a home dialysis programme serving a total of 406 patients dialysing via; graft 2.7%, intravascular catheter (IVC) 12% and an arterio-venous fistula (AVF) 85.2%.
Since 2007 there has been a SA screening programme for all chronic haemodialysis patients and following mandatory surveillance of meticillin- sensitive SA (MSSA) in 2011 a Root Cause Analysis (RCA) has been performed for every SAB.
**Results:** The focus of infection was identified, common themes monitored and actions prioritised with the aim to reduce future SAB.
Since monitoring in 2011 there has been a sustained reduction of SAB until this year.
Defined focus of infection and common themes were
- Patients with grafts or pacemakers' in-situ
- IVC related infections
- Patients who had recurring SAB
- Patients who were SA positive on either line insertion or routine surveillance
- Patients allergic to the routine skin preparation
- Management of patients following SAB result.

**Conclusion:** Specific actions were identified and prioritised.
An IVC insertion checklist was developed to ensure patients who were SA positive received the correct decolonisation treatment prior to line insertion.
A Patient Group Directive (PGD) sticker was developed which allows nursing staff to promptly dispense decolonisation treatment for SA positive patients.
'High risk' patients were identified and antibiotic prophylaxis was agreed.
Persistent colonisers with SA were offered an antimicrobial wash for use at home.
A SA blood culture (STaB) sticker highlighting specific treatment details was developed to standardise the management of patients and prevent recurrence.
**Disclosure of Interest**
None Declared

### P252
### Burden of multidrug-resistant bacteria in hospital environment in slovakia: results from one-year multicenter prevalence study Hospital-Enviro-Rez
Lenka Michalikova[1], Jaroslava Brnova[1], Sona Hnilicova[1], Anna Liskova[2], Zuzana Sirotna[3], Vladimir Krcmery[4]
[1]Department of Laboratory Medicine, School of Health Sciences and Social Work, Trnava University, Trnava; [2]Laboratory of Molecular Microbiology, St. Elisabeth University; [3]National reference laboratory for environmental microbiology, Public Health Authority of Slovak Republic; [4]Laboratory of Molecular Microbiology, St. Elisabeth University, Bratislava, Slovakia
**Correspondence:** Lenka Michalikova
*Antimicrobial Resistance and Infection Control* 2017, **6(Suppl 3):**P252

**Introduction:** The role of hospital environment in harboring and transmitting multidrug-resistant (MDR) organisms has become clearer due to a series of publications linking environmental contamination with increased risk of hospital-associated infections.
**Objectives:** The aim of this study was to assess burden of antibiotic resistance in bacteria isolated from hospital environment in Slovakia.
**Methods:** One-year multicenter prevalence study was performed in January to December 2015 and positive strains isolated from hospital environment of all Slovak hospitals were included in the study. Antimicrobial susceptibility was determined using disc diffusion and colorimetric test according to EUCAST. The presence of genes encoding multiresitance were performed by polymerase chain reaction (PCR). Statistical analyses were performed using R-project and $P < 0{,}05$ was considered significant.
**Results:** From overall 22 922 hospital environment swabs were together 800 (4,5%) positive. Staphylococcus spp. (27,8%), Enterobacter spp. (18,2%), Enterococcus spp. (17,6%) and Pseudomonas spp. (13,9%) were the most frequently isolated species. Phenotype of MDR was confirmed in 28,5% isolates, and was significant higher in gram-negative bacteria (36,8% vs. 18,1%; $P < 0{,}0001$). MRSA and VRE from all Staphylococcus aureus (204) and Enterococuss spp. (134) positive swabs were found in 21,1% and 3,6% respectively. None VRSA was isolated from hospital environment in Slovakia. Production of ESBL was identified in 29,7% gram-negative bacteria and production of carbapenemases was confirmed in 8,3% of isolates with meropenem resistance ($n = 66$). Vancomycin coding gene vanA were found only in 9 isolates of Enterococcus spp.
**Conclusion:** This is the first study concerning on burden of MDR bacteria in hospital environment in Slovakia. These results highlight the significance of better infection control practise and performing effective measures to reduce spreading of MDR bacteria within Slovakia health care facilities.
**Disclosure of Interest**
None Declared

### P253
### Infection control strategies to prevent methicillin-resistant staphylococcus aureus infections in a tertiary hospital
Sharifah Azura Salleh, Nordiah Jalil, Siti Rokiah Yusuf, Nor Rina Mahawar, Razila Yacob, Farahzilla Anuwar, Norhayatie Mohamad Kosnen
Infection Control Unit, Universiti Kebangsaan Malaysia Medical Centre, Kuala Lumpur, Malaysia
**Correspondence:** Sharifah Azura Salleh
*Antimicrobial Resistance and Infection Control* 2017, **6(Suppl 3):**P253

**Introduction:** MRSA is one of the predominant pathogens in healthcare-associated infections (HAIs) with significant morbidity and mortality.
**Objectives:** Our institution implemented infection prevention and control strategies for the identification, management and control of MRSA to reduce the risk of transmission and acquiring MRSA HAI.
**Methods:** MRSA surveillance program was part of the Infection Control Unit activity at our 880-bed tertiary teaching hospital. All positive MRSA culture results upon validation were placed in a designated box in the Microbiology Laboratory. Daily laboratory data were collected and entered into the MRSA line listing by infection control nurse (ICN). All affected MRSA patients were visited by ICN to ensure adherence to recommended precautions including isolating or cohorting patients; emphasizing on the 5-moments of hand hygiene; contact precautions; cleaning and disinfection of equipment and the environment; and visitor policies. The ICN ward round also determined whether patients had active MRSA infection or colonization. Decolonization of MRSA colonizers was done with daily chlorhexidine baths and intranasal mupirocin. Infected MRSA cases were treated with vancomycin. Staff education regarding MRSA was emphasized. Active surveillance test for MRSA screening in ICU and NICU upon admission and weekly was also conducted. Outcome of MRSA HAI rates and total MRSA (infection plus colonization) rates were monitored and reported to relevant stakeholders. Data analysis was based on excluding duplicate MRSA isolates from the same patient.

**Results:** The total MRSA rates were 0.98, 1.19, 1.53 and 1.48 per 1000-patient days in year 2013, 2014, 2015 and 2016 respectively. However, a declining trend of MRSA HAI rates were observed with 0.17, 0.14, 0.10, and 0.08 per 1000-patient days for those respective years. Nonetheless our MRSA HAI rates were also far below the national MRSA HAI rates. These encouraging results might be attributed to the proactive implementation of recommended practices.

**Conclusion:** Proactive implementation of recommended practices will help in preventing and controlling the spread of MRSA as decreasing MRSA HAI rates were observed throughout the years.

**Disclosure of Interest**

None Declared

### P254

**Withdrawn**

### P257

#### New peptides with antibacterial and potential immunomodulatory activity against multiresistant staphylococcus aureus

Laura A. Barrero Guevara, Helena Groot de Restrepo, Carolina Muñoz-Camargo

Department of Biological Sciences, Universidad de los Andes, Bogotá, Colombia

**Correspondence:** Laura A. Barrero Guevara

*Antimicrobial Resistance and Infection Control* 2017, **6(Suppl 3):**P257

**Introduction:** *Staphylococcus aureus* infections are a common concern worldwide due to the increasing number of bacterial strains with multiresistant properties to existing antibiotics, incrementing the need for novel molecules and therapy approaches for their treatment.

**Objectives:** In this study we evaluated the antimicrobial activity against reference and clinical isolated multiresistant strains of *S. aureus* and the immunomodulatory potential of new antimicrobial peptides.

**Methods:** The half maximal inhibitory concentration (IC50) of 8 peptides individually and in combination was determined with a microdilution technique against an ATCC strain and 4 clinically isolated *Methicillin-Resistant S. aureus* (MRSA). Additionally, cytotoxicity was measured with MTT assays *in vitro* in a Vero cell line. Finally, immunomodulatory potential of the peptides was evaluated predicting potential binding affinity to receptors of the immune system with SYFPEITHI database and the Immune Epitope Database and Analysis Resource (IEDB).

**Results:** Individually, 5 of the peptides tested inhibited significantly the bacterial growth of the reference strain of *S. aureus*, but only 3 of them demonstrated antimicrobial activity against clinical isolated bacteria. In combination, the peptides had significantly inhibitory effect in the bacterial growth of reference and multiresistant *S. aureus* strains. In addition, peptides individually and in combination did not present cytotoxic activity, not even in the highest concentrations tested. Regarding the immunomodulatory potential, 5 peptides were predicted to have affinity with immune receptors.

**Conclusion:** Taken together, these results demonstrate the potential of using these peptides in combination to be used as novel antimicrobial molecules against *S. aureus,* with the possibility of having immunomodulatory capacities. Furthermore, this study remarks the importance of testing novel antimicrobial molecules not only against reference strains, but also against multiresistant bacteria, as well as a possible approach using combinations of different molecules for the treatment for multiresistant bacteria in clinical practice.

**Disclosure of Interest**

None Declared

### P258

#### Epidemiology of biofilm formation and virulence in clinical methicillin- resistant staphylococcus aureus (MRSA) isolates

Susanne Altaner, Frank Günther, Nico T. Mutters, Nicole Henny

Department of Infectious Diseases, Heidelberg University Hospital, Heidelberg, Germany

**Correspondence:** Susanne Altaner

*Antimicrobial Resistance and Infection Control* 2017, **6(Suppl 3):**P258

**Introduction:** MRSA is an important cause of nosocomial infections with a high rate of morbidity and mortality worldwide. The ability of certain isolates to form biofilms is an important aspect of the pathogenesis in Staphylococcus aureus infections.

**Objectives:** The goal of our study was to evaluate the extent to which the formation of biofilms and the specific strength of virulence patterns influenced the epidemiological spread and the persistence of distinct clones and lineages in the hospital setting.

**Methods:** We analyzed 347 MRSA isolates, collected between 2015 and 2017 at the Heidelberg University Hospital, a large German 2,200 bed university teaching hospital. The epidemiological background of each collected isolate was analyzed by using Spa-typing and BURP-clustering. Native and induced biofilm formation properties of the MRSA isolates were analyzed by crystal violet technique. The presence of most relevant virulence factors (Panton-Valentine Leukocidin: PVL, Toxic shock syndrom toxin: TSST, and Enterotoxine A and B) were analyzed by PCR.

**Results:** The strongest native, as well as induced biofilm forming properties were associated to Spa-type t535 (mean $0.93 \pm 0.093$) and clonal complex cc008 ($0.40 \pm 0.19$). PVL was detected in 41(11.8%), TSST in 31(8.9%), SEA in 53(15.2%) and SEB 27(7.8%) in 347 MRSA isolates. While presence of SEA was associated to elevated native biofilm formation as well as induced biofilm formation, the presence of lucPV and TSST was associated to elevated inducibility of biofilm formation ($p < 0.05$). The tested clinical MRSA isolates showed a broad range of biofilm formation potential. The mean native biofilm formation was $0.32 \pm 0.15$, while the mean glucose induced biofilm formation was $0.46 \pm 0.17$. Addition of 2% glucose resulted in an average increase of 1.74 compared to the native formation. However, 54(15.6%) MRSA isolates showed no inducibility or even decreased biofilm formation.

**Conclusion:** Clinical MRSA isolates show a broad range of biofilm formation. Distinct MRSA types and clonal lineages with outstanding ability to form biofilms under native as well as induced conditions might therefore cause serious problems in treatment and infection control measures especially if linked to additional virulence patterns.

**Disclosure of Interest**

None Declared

### P259

#### Bacteriological screening of breast milk samples destined to direct milk donation: prospective evaluation between 2007 and 2015

Claire Masson[1], Constance Minebois[1], Catherine Braux[1], Isabelle Pelloux[2], Leila Marcus[3], Valérie Belin[3], Fabrice Cneude[3], Thierry Debillon[3], Caroline Landelle[1, 4], Marie-Reine Mallaret[1, 4]

[1]Infection Control Unit; [2]Bacteriology laboratory; [3]Neonatology and Neonatal Intensive Care Department, CHU Grenoble Alpes; [4]ThEMAS TIM-C UMR 5525, University Grenoble Alpes/CNRS, Grenoble, France

**Correspondence:** Claire Masson

*Antimicrobial Resistance and Infection Control* 2017, **6(Suppl 3):**P259

**Introduction:** In France, direct milk donation from the mother to her preterm babies <34 weeks of amenorrhea is possible; bacteriological quality of breast milk samples was assessed according to national recommendations.

**Objectives:** The objective of our study was to analyze the bacteriological quality of breast milk samples destined to direct milk donation in a French university hospital.
**Methods:** All the samples from the milk destined to direct milk donation in the neonatalogy and neonatal intensive care units at the Grenoble teaching hospital from March 2007 to April 2015 were included. A sample was considered to be correct if its total flora was less than $10^6$ CFU/mL and in the absence of *Staphylococcus aureus* and other pathogens (Enterobacteriaceae…). In case of incorrect result, another sample was analysed. The non-compliance rate was analysed between 2008 and 2014.
**Results:** Six hundred and two breast milk samples were taken from the milk of 500 mothers. The overall non-compliance rate for the 1st sample was 20.2%; 55 (54.5%) had a total flora $\geq 10^6$ CFU/mL, 46 (45.5%) were contaminated with *S. aureus* and 7 (6.9%) by other pathogens. A statistically significant increase of the non-compliance rate was observed between 2008 and 2014 (10.2% to 29.8%) explained by a statistically significant increase in the rate of samples with a total flora $\geq 10^6$ CFU/mL. No statistically significant increase in the rate of *S. aureus* contamination and other pathogens was observed. The overall non-compliance rate was 45.6% for the 1st controlled samples and 28.6% for the 2nd controlled samples.
**Conclusion:** The absence of any increase in contamination by *S. aureus* or other pathogens is reassuring. The increase of the non-compliance rate, due to an increase of samples with a total flora $\geq 10^6$ CFU/mL, could be explained by a decrease of good practices in milk collection. The education of mothers about milk collection should be strengthened.
**Disclosure of Interest**
None Declared

### P260
### Exploring the relative importance of different surfaces in nosocomial pathogens transmission via contact route
Hao Lei[1], Rachael Jones[2], Yuguo Li[1]
[1]The University of Hong Kong, Hong Kong, China; [2]University of Illinois at Chicago, Chicago, United States
**Correspondence:** Hao Lei
*Antimicrobial Resistance and Infection Control* 2017, **6(Suppl 3):**P260

**Abstract video clip:** In this study, we used data from literature on methicillin-resistant *Staphylococcus aureus* (MRSA) and developed a stochastic mathematical model to study how contact heterogeneity on different types of hospital environmental surfaces, when noncompliance of daily surface hygiene happened, may lead to different exposure to the susceptible patients via contact route in a hypothetical 6-bed intensive-care unit. The susceptible patients are divided into two types, these who are cared by the same nurse as the index patient (Type 1) and these who are not (Type 2). The results showed that for both types susceptible patients, the patient nearby high-touch surfaces (HTSs) had the most impact. If the HTSs were not cleaned, the pathogen exposure to Type 1 and 2 susceptible patients would increase 56% (95% confidence interval (CI): 54% > 58%) and 106% (95% CI: 100% > 112%) respectively. The communal surfaces (CSs) had the least impact, which could only lead a less than 10% increase of the pathogen exposure to two types susceptible respectively if they were not cleaned. The impact of clinical equipment (CE) differed largely for two types of susceptible patients. If the CE was not cleaned, the exposure to Type 1 patients would only increase 5% (95% CI: 5% > 6%), while for Type 2 patients, it can increase 74% (95% CI: 68% > 79%). This study quantitatively showed the importance of cleaning patient nearby surfaces, especially the HTSs, and the CE, on controlling the nosocomial infection transmission via contact route.
**Disclosure of Interest**
None Declared

### P261
### Panton–valentine leucocidin expression and antibiotic resistance in staphylococcus aureus isolated from skin and soft tissue infections and wounds in Nigeria
Olayemi O. Ayepola[1], Nurudeen A. Olasupo[2], Louis O. Egwari[1], Frieder Schaumburg[3]
[1]Biological Sciences, Covenant University, Ota; [2]Microbiology, Lagos State University, Ojo, Nigeria, [3]Medical Microbiology, University Hospital, Munster, Germany
**Correspondence:** Olayemi O. Ayepola
*Antimicrobial Resistance and Infection Control* 2017, **6(Suppl 3):**P261

**Introduction:** The tenacity of *Staphylococcus aureus* as a pathogen can be attributed to its arsenal of virulence factors among which is the panton–valentineleukocidin (PVL) a leukocytolytic toxin that has been epidemiologically associated with severe cutaneous and necrotizing infections as well as bone and joint infections. Traditionally the toxin has been associated with community-acquired MRSA infections but Sub-Saharan Africa is considered to be a PVL endemic region showing PVL prevalence among *S. aureus*, particularly methicillin susceptible isolates.
**Objectives:** The study was carried out to detect the presence of the PVL gene as well as the expression of the toxin and antibiotic resistance in methicillin susceptible *S. aureus* strains isolated from soft tissue infections (SSTIs) and wounds.
**Methods:** A total of 97 *S. aureus* isolates were obtained from SSTIs (49) and wounds (48).The presence of the PVL gene (*lukS-PV*) was detected by polymerase chain reaction. Antimicrobial susceptibility testing of *S. aureus* was carried out using the Vitek 2 automated systems. Extracellular expression of PVL by *lukS-PV*–positive strains was evaluated by a Western blot.
**Results:** The PVL gene was detected in 76% of SSTI isolates and 77% of wound isolates. Rabbit anti-*LukS-PV* detected a band of MW 32 kD corresponding to *LukS-PV*. The level of PVL produced by *lukS-PV*–positive *S. aureus* isolates varied from strain to strain. The expression of PVL was observed in 75% of SSTI isolates and 62% of wound isolates. Resistance was highest to penicillin (100% in SSTI isolates and 94% in wound isolates); followed by trimethoprim/sulfamethoxazole (84% in SSTI isolates and 83% in wound isolates); and tetracycline (8% in SSTI isolates and 10% in wound isolates).
**Conclusion:** A qualitative expression of the protein has been found to correlate with severity for specific clinical manifestation, most especially skin and soft tissue infections. Prevalence of antibiotic resistant PVL-positive methicillin susceptible *S. aureus* strains is of great concern as this could promote the emergence of more virulent strains with severe implications on PVL mediated infections.
**Disclosure of Interest**
None Declared

### P262
### Ouabain as a potential biofilm inhibitor against staphylococcus aureus
Antresh Kumar[1], Ajay K. Singh[2]
[1]Biotechnology; [2]Bioinformatics, CENTRAL UNIVERSITY OF SOUTH BIHAR PATNA, Patna, India
**Correspondence:** Antresh Kumar
*Antimicrobial Resistance and Infection Control* 2017, **6(Suppl 3):**P262

**Introduction:** *Staphylococcus aureus* is a dangerous human pathogen which causes a diverse range of superficial and invasive diseases in hospital and community-acquired infections with a higher morbidity and mortality. Biofilm formed by *S. aureus* anchored on medical devices; host tissue that restricts penetration of antibiotics and significantly contribute in drug resistance. It is a serious concern to find out a new approach or antimicrobials to eradicate such infections. Repurposing already-approved drugs with well-characterized toxicology and pharmacology is a novel way to reduce the time, cost, and risk associated with antibiotic innovation.

**Objectives:** Evaluation the therapeutic activity of ouabain against *S. aureus*.
**Methods:** Drug susceptibility, biofilm inhibition, haemolytic assay and microscopic analysis were employed in this study.
**Results:** Ouabain is well known and clinically safe cardiac drug that used to treatment of cardiac shock by inhibiting $Na^+$ /$K^+$ ATPase pump. Ouabain acts as biofilm inhibitor as it significantly reduces established staphylococcal biofilm at 3uM without affecting of *S. aureus* growth even tested up to 10 mM concentration. Ouabain inhibited biofilm formation at micromolar concentration which confirmed by microscopic analysis. *S. aureus* cells treated with 5 mM ouabain altered over all cell wall hydrophobicity with 85% reduction in hydrophobic as compared to control. Additionally, ouabain remarkably inhibits toxins production in *S. aureus*. Intriguingly, ouabain synergized the antimicrobial activity of the aminoglycoside antibiotics, elicited hypersensitive cell susceptibility. Such therapeutic efficacy of ouabain is through depolarization of cell membrane and reduction of cell hydrophobicity with no haemolytic activity.
**Conclusion:** This study provides an insight that ouabain acts as a potent biofilm inhibitor by reducing cell wall hydrophobicity, adherence and may be used as an anti-biofilm leading molecule for clearing up *S. aureus infections*.
**Disclosure of Interest**
A. Kumar Employee of: no conflict, Grant/Research support from: no conflict, Speaker's bureau of: no conflict, A. Singh Employee of: no conflict, Grant/Research support from: no conflict, Speaker's bureau of: no conflict

### P263

#### Methicillin-susceptible staphylococcus aureus in the neonatal intensive care unit: do not underestimate the spread

Caroline Fondrinier[1], Pascal Thibon[2], Claire Lesteven[1], Marguerite Fines-Guyon[3], Vincent Cattoir[3], Xavier Le Coutour[1], Bernard Guillois[4]
[1]Hygiène Hospitalière; [2]ARLIN, Basse Normandie, [3]Microbiologie, [4]Neonatalogie, CHU CAEN, Caen, France
**Correspondence:** Caroline Fondrinier
*Antimicrobial Resistance and Infection Control* 2017, **6(Suppl 3):**P263

**Introduction:** We observed two methicillin-susceptible *Staphylococcus aureus* (MSSA) outbreaks in a neonatal intensive care unit (NICU) and the spread of an identified clone during two years
**Objectives:** We conducted an epidemiological study to describe and identify risk factors of MSSA acquisition in order to prevent neonatal colonization.
**Methods:** A retrospective cohort study was performed from may the 1st to September the 30, 2014, including all the children with at least one nasal screening. For each infant, 35 risk factors were collected: clinical characteristics, exposition to healthcare workers and invasive devices, colonization pressure etc. A univariate analysis and a multivariate logistic regression analysis were used to identify the factors responsible for MSSA acquisition.
**Results:** 113 neonates were included, including 31 cases. The significant risk factors in both univariate and multivariate logistic regression analysis were for MSSA colonization: gestational age (<33 weeks; RR = 7.41) and birth weight (<1500 g; RR = 4.13). In multivariate analysis surgery was also a significant risk factor (RR = 5.13, IC95 [1.60-16.5]; p = 0.006). The mean of percent colonization pressure was significantly higher for colonized infants. It was a strong independent predictor of MSSA acquisition.
**Conclusion:** The risk factors for acquiring a MSSA among infants in NICU were a low gestational age and a low birth weight especially when the colonization pressure was high. We confirm the importance of infection control program to prevent MSSA acquisition and the need of integrating MSSA with methicillin-resistant *Staphylococcus aureus* in an epidemiological surveillance program in NICU. When MSSA is the most prevalent *Staphylococcus aureus* type, and the colonization pressure was high, decolonization should be considered for MSSA colonized neonates to prevent an outbreak.
**Disclosure of Interest**
None Declared

## Extended spectrum beta-lactamase (ESBL)

### P264

#### Ugly bugs in the healthy guts! - carriage of multidrug resistant and esbl producing commensal enterobacteriaceae in the intestine of healthy Nepalese adults

Narayan P. Parajuli, Anjila Maharjan, Govardhan Joshi, Puspa R. Khanal
Laboratory Medicine, Manmohan Memorial Institute of Health Sciences, Kathmandu, Nepal
**Correspondence:** Narayan P. Parajuli
*Antimicrobial Resistance and Infection Control* 2017, **6(Suppl 3):**P264

**Introduction:** Carriage of multidrug resistant and ESBL producing bacteria in the human gastrointestinal tract is a major factor for infection with antibiotic resistant bacteria.
**Objectives:** The objective of the study was to determine the incidence of ESBL producing enterobacterial strains colonizing the intestinal tract of apparently healthy adults in Kathmandu, Nepal.
**Methods:** A total of 510 stool specimens were obtained from apparently healthy adult students of a health science college in Kathmandu, Nepal. Predominant fecal commensal isolates were isolated, identified, and the antimicrobial susceptibility test was performed according to the standard microbiological methods. Multidrug resistant isolates were selected for ESBL confirmation by combined disk test and E-test methods. Molecular characterization of plasmid borne ESBL genes was performed by using specific primers of CTX-M, SHV and TEM by polymerase chain reaction.
**Results:** Among five hundred and ten fecal commensal bacteria recovered from participants; *Escherichia coli* (432, 84.71%) was the predominant organism followed by *Klebsiella oxytoca* (48, 9.41%) and *Klebsiella pneumoniae* (30, 5.88%). ESBLs were isolated in 9.8% of the total isolates including *Klebsiella oxytoca* (29.17%), *Escherichia coli* (7.87%) and *Klebsiella pneumoniae* (6.67%). Among ESBLs, bla-TEM was the predominant type (92%) followed by bla-CTX-M (60%) and bla-SHV (4%).
**Conclusion:** Multidrug resistant and ESBL producing enterobacterial commensal strains among healthy individuals is of serious concern. Carriage of ESBL organisms in healthy individuals suggest the possibility of sustained ESBL carriage among the diseased and hospitalized patients. Similar type of epidemiological survey in larger community and hospital setting is recommended.
**Disclosure of Interest**
None Declared

### P265

#### MDRO surveillance for patients who receive treatment abroad and return to the Turks and Caicos Islands Hospital

Surbrena J. Forbes-Pedican
Infection Prevention and Control, The Turks and Caicos Islands Hospital, Blue Hills, Turks and Caicos Islands
*Antimicrobial Resistance and Infection Control* 2017, **6(Suppl 3):**P265

**Introduction:**
From November 2015 to February 2016 ESBL Klebsiella pneumoniae and other MDROs were isolated from urine and wound cultures of 14 patients. An outbreak investigation was launched and containment measures were implemented, the investigation revealed that the common causative factor among the patients was treatment abroad.
**Objectives:**
To prevent and control the spread of MDROs within the healthcare setting and regulate the use of antibiotics.
**Methods:**
The investigation was conducted from November 2015 until February 2016. Data was collected to characterize the suspected infections using a categorization tool with a range of categories including organism, diagnosis, surgical procedure, treatment abroad country and date. The IPC department initiated measures in collaboration with other departments within the hospital as follows to prevent spread of the organism in the facility:
1. Development of a specific panel to identify specific MDROs

2. Microbial culture swabs routinely taken for patients pre and post treatment abroad
3. Periodic swabbing of staff and hospital environment for colonization of MDROs

**Results:**

A total of 14 patients were identified with infections with MDROs after treatment abroad including 1 fatality. There were 23 swabs from attending staff and 46 hospital environmental swabs across both sites analyzed, none was tested positive for MDROs. Lab microbial surveillance from January to December 2016 identified 108 MRSA isolates, 55 ESBL E. coli, 26 ESBL Klebsiella pneumonia, 3 Multi drug resistant Acinetobacter species and 6 cases of Clostridium difficile. These isolates were identified among both in and out patients who were treated in the hospital as well as some of whom had no treatment abroad.

**Conclusion:**

The active surveillance of all positive culture results in the Turks and Caicos Islands hospital led to the identification of new MDROs in the country. This information was vital for the implementation of infection control measures to prevent the spread and colonization of these organisms within the hospital. It has also been instrumental in the development of the Antimicrobial stewardship program which is currently being implemented in the hospital.

**Disclosure of Interest**

None Declared

### P266

#### Carriage rate of multi-drug resistant bacteria among Palestinian authority residents and tourists seeking advanced medical care in Israel

Matan J. Cohen, Ran Nir Paz, Carmela Schwartz, Sharon Amit, Allon E. Moses, Shmuel Benenson
Clinical Microbiology and Infectious Diseases, Hadassah-Hebrew University Medical Center, Jerusalem, Israel
**Correspondence:** Shmuel Benenson
*Antimicrobial Resistance and Infection Control* 2017, **6(Suppl 3):**P266

**Introduction:** Medical tourism is an emerging approach for supplying medical care for patients lacking sufficient medical services in their home countries. Such transfer of patients may also impose the transfer of resistant pathogens across the globe. Hadassah Medical Center serves as a referral center for global medical tourists as well as residents of the Palestinian authority.

**Objectives:** We sought to assess whether patients of both these groups are more likely to harbor multi-drug resistant bacteria (MDR) and thus necessitate pre-emptive isolation.

**Methods:** We reviewed all patient admissions to our institution between 2009 and 2014, from whom at least one microbiological sample was collected. We examined patient sex, age, nationality, previous hospitalization and time from arrival to MDR detection.

**Results:** During the 5 years of the study there were 942,702 referrals of 471,106 patients; 111,577 patients had at least one microbiological specimen taken during hospitalization. Overall, there were 3,985 (3.5%) patients with at least one MDR-positive culture. MDR positivity rates were highest among patients from the Palestinian authority (12.2%) followed by other foreign tourists (5.4%). Compared to Israeli patients, controlling for sex, age and previous hospitalization, patients from the Palestinian authority and tourists were had increased rates of MDR positivity (OR, 95%CI): 8.0 (6.3 to 10.1) and 2.3 (1.6 to 2.3), respectively.

**Conclusion:** Foreign patients seeking advanced medical care are more likely to carry MDR bacteria than the local patients. Strategies to minimize the spread of such bacteria may include pre-admission screening or pre-emptive isolation until MDR carriage status is verified.

**Disclosure of Interest**

None Declared

### P267

#### Improving admission procedures of patients at risk of multidrug-resistant organisms: an educational intervention

Dominique JOUBERT[1], Stéphane CULLATI[2], Anne-Claire RAE[1], Bernard MUGNIER[1], Didier PITTET[1] on behalf of Geneva University Hospitals, Switzerland
[1]HUG; [2]University of Geneva, Geneva, Switzerland
**Correspondence:** Dominique JOUBERT
*Antimicrobial Resistance and Infection Control* 2017, **6(Suppl 3):**P267

**Introduction:** Emergency Units (EU) can contribute to prevent healthcare-acquired infections and outbreaks. In a pilot survey at our EU, healthcare workers' (HCWs) compliance with admission screening (AS) of patients colonized by multidrug-resistant organisms (MDROs) was 35% and compliance with additional precautions (AP) was 48%. We conducted a cross-sectional survey to better understand HCWs' perceptions of MDRO control procedures and evaluated to impact of an educational intervention to improve HCWs' compliance with infection control (IC) procedures.

**Objectives:** To explore HCWs' perceptions and knowledge of MDRO procedures and to improve compliance with AS procedures.

**Methods:** This study was conducted at the EU of the University Hospitals of Geneva, Switzerland. Before the intervention, two data collections were performed in 2016. Compliance audits and a cross-sectional survey exploring knowledge and perceptions toward AS of MDRO carriers were conducted. Audit targeted AS and compliance with AP including bed/environment signage, protective equipment, and compliance during care. An educational intervention was conducted; it included two components: a) training sessions based on the results of the survey carried out among HCWs; b) a new computerized support tool ("Order set") was implemented to simplify AS and prescription of AP. After the intervention, compliance audits were conducted among a total of 100 HCWs.

**Results:** 136 (57%) EU HCWs answered the survey. 11% knew the probability of being a MDRO carrier and 7% correctly identified MDROs. 31% reported that AS was a high priority. During the educational intervention, almost all EU HCWs (n = 180) were trained. Compliance to AS for MDROs improved from 35% [95% CI 25.2-44.8] before the intervention to 76% [95% CI 67.6- 84.4] after (p = 0.034). Compliance with the adequate AP improved from 48% [95% CI 38.1-57.9] before the intervention to 65% [95% CI 55.7-74.3] after (p = 0.058)

**Conclusion:** Results indicated that knowledge about emerging MDRO and related risk factors of carriage was poor among EU staff. After the educational intervention, EU staff compliance with AS of high-risk patients improved. This quality improvement project is an excellent example of collaboration between EU and infection control staff.

**Disclosure of Interest**

None Declared

### P268

#### An outbreak of extended-spectrum beta-lactamase (ESBL)-producing klebsiella pneumoniae in a rehabilitation center: an unusual source and supposed route of transmission

Marrit Boonstra[1], Dorine Spijkerman[2], Anne Voor in 't holt[1], Rob Van der Laan[2], Lonneke Bode[1], Wim Van Vianen[1], Corné Klaassen[1], Margreet Vos[1], Juliëtte Severin[1]
[1]Medical Microbiology and Infectious Diseases, Erasmus MC; [2]Rijndam Rehabilitation Center, Rotterdam, Netherlands
**Correspondence:** Juliëtte Severin
*Antimicrobial Resistance and Infection Control* 2017, **6(Suppl 3):**P268

**Introduction:** Outbreaks due to multidrug-resistant microorganisms in rehabilitation centers have rarely been reported.

**Objectives:** To describe an outbreak of ESBL-producing *Klebsiella pneumoniae* (ESBL-*K. pneumoniae*) at a single ward in a rehabilitation center in Rotterdam, the Netherlands.

**Methods:** In October 2016, two patients were found to be colonized by the same ESBL-*K. pneumoniae* strain. Contact tracing suggested transmission to seven other patients. Additional infection control measures were taken, which included weekly screening of patients for ESBL colonization. Also, environmental cultures were taken (n = 163). Swabs were directly plated on Brilliance ESBL agar (Oxoid). ESBL was confirmed phenotypically. Isolates were genotyped using multilocus variable number tandem repeat analysis (MLVA).
**Results:** During the outbreak period (October 2016 until December 2016), 14 patients were colonized with an ESBL-*K. pneumoniae*, of which 11 patients had an isolate with an identical genotype. Contact precautions were applied for all 14 patients, but adapted in such a way that the rehabilitation process could be continued. A household washing machine used for washing mats, linen, but also patient clothes contaminated with faeces was identified as a likely source. Several sites of the machine were repeatedly found to be contaminated with the outbreak strain. In an experimental setting, clothes that were contaminated with ESBL-*K. pneumoniae* prior to washing remained positive after washing at a temperature of 30 °C but became negative after washing at a temperature of 90 °C. The outbreak was eventually controlled by several measures including the implementation of new protocols on the use of the washing machine.
**Conclusion:** In this outbreak, a household washing machine was identified as an unusual link in the chain of transmission. We hypothesize that the route of transmission of the outbreak strain was via the washing machine that led to contamination of clothes. We stress the need for specific infection prevention guidelines for rehabilitation centers.
**Disclosure of Interest**
None Declared

### P269
### Prevalence on admission and acquisition of extended-spectrum betalactamase producing –enterobacteriaceae (ESBL-E) in geriatrics

Carolina Fankhauser-Rodriguez[1], Jacques Schrenzel[2], Virginie Prendki[3], Marc Desilets[3], Valérie Sauvan[1], Marie-Noelle Chraiti[1], Friederike Maechler[4], Petra Gastmeier[4], Stephan Harbarth[1]
[1]Infection Control Program; [2]Bacteriology Laboratory; [3]Internal Medicine and Rehabilitation Service, HUG, Geneva, Switzerland; [4]RGNOSIS WP5, Charité Hospital, Berlin, Germany
**Correspondence:** Carolina Fankhauser-Rodriguez
*Antimicrobial Resistance and Infection Control* 2017, **6(Suppl 3):**P269

**Introduction:** The increasing prevalence of ESBL-E in the community is a cause of concern for hospitals. Early detection of ESBL-E carriers could allow timely implementation of control measures, appropriate selection of antimicrobials and eventually decrease hospital-acquired infections.
**Objectives:** To describe the prevalence upon admission, and the acquisition rate of ESBL-E on 2 geriatric wards, during 2 periods, applying only Standard Precautions (SP) versus implementation of Contact Precautions (CP) for ESBL carriers, in the context of a multicenter trial (R-Gnosis WP5).
**Methods:** From Jan 2014 to Feb 2016, anal swabs were collected (on admission, discharge and weekly) from patients (pts) admitted to 2 wards. All ESBL-positive pts were put under CP from Jan 2014 to Jan 2015; and under SP only, from Feb 2015 to Feb 2016. Compliance with hand hygiene (HH) was measured during both periods.
**Results:** During **SP** and **CP** periods, respectively, median age (y) was 84.8 (±8.5) and 84.5 (±7.8); 31.5% and 33.8% were male. Median length of stay (d) was 26.3 (±2.9) and 24.01(±1.6). A total of 2022 and 1652 anal swabs were performed, with a mean of 4.8 (±3.5) and 4.2 (±2.3) swabs per patient; and with 95.6% (392/410) and 95.4% (375/393) pts screened on admission. 13.5% (53/392) and 10.9% (41/375) of pts were found to be ESBL-E carriers on admission, mostly ESBL-*E. coli* (35/53 and 27/41), and *K. pneumoniae* (7/53 & 3/41); and a previously known ESBL carriage of 7.9% (31/392) and 5.6% (19/375). During hospitalization, 25 and 27 new ESBL-E cases were detected: *E. coli* (12/25 and 13/27); *K. pneumoniae* (4/25 & 4/27) and other ESBL-E (9/25 & 10/27); with an attack rate (AR) of 2.29 and 2.8 new cases/1000 pt-days (p = 0.06). 7 and 2 patients acquired ESBL-E infections with an AR of 0.64 and 0.2 cases/1000 pt-days (p = 0.85). HH compliance was 78% and 80%, with 497 and 409 opportunities observed.
**Conclusion:** On admission carriage of ESBL-E increased during the study.
There were no significant differences in ESBL-E acquisition or infection rates between SP and CP periods.
**Disclosure of Interest**
None Declared

### P270
### High prevalence of esbl-producing bacteria in private and shared latrines in an informal urban settlement in Dar Es Salaam, Tanzania

Stefan Herb[1], Lauren D'Mello[2, 3], Hamisi M. Malebo[4], Robert M. Njee[5, 6], Fatuma Matwewe[3], Jeroen Ensink[2], Vladimira Hinic[7], Andreas Widmer[8], Reno Frei[7, 8]
[1]Division of Infectious Diseases and Hospital Epidemiology, University Hospital Basel, Basel, Switzerland; [2]Environmental Health Group, Faculty of Infectious and Tropical Diseases, London School of Hygiene and Tropical Medicine, London, United Kingdom; [3]Maji Safi kwa Afya Bora Ifakara (MSABI), Morogoro; [4]Department of Traditional Medicine Research; [5]National Institute for Medical Research; [6]Ministry of Health, Community Development, Gender, Elderly and Children, Dar es Salaam, Tanzania, United Republic of; [7]Division of Clinical Microbiology; [8]Division of Infectious Diseases and Hospital Epidemiology, University of Basel, Basel, Switzerland
**Correspondence:** Hamisi M. Malebo
*Antimicrobial Resistance and Infection Control* 2017, **6(Suppl 3):**P270

**Introduction:** Data about the burden of extended-spectrum beta-lactamase (ESBL)-producing microorganisms in Africa are limited. The prevalence of human faecal ESBL carriage in the community of an informal urban settlement in Dar es Salaam, Tanzania is estimated by using environmental contamination of household latrines with ESBL as a surrogate marker.
**Methods:** Within the context of a large survey in February 2014 assessing 636 randomly selected household latrines for faecal contamination by the detection of growth of E. coli and total faecal coliform bacteria, a subset of the samples were screened for ESBL.
**Results:** Seventy latrines were randomly selected for ESBL screening. An average of 11.4 persons (SD ±6.5) were sharing one latrine. Three (4.3%) latrines had hand-washing facilities and 50 showed faeces on the floor. ESBL-producing Enterobacteriaceae were confirmed in 17 (24.3%) of the 70 latrine samples: 16 E. coli and 1 Klebsiella pneumonia. Five ESBL E. coli strains were detected on door handles. The most prevalent ESBL type was CTX-M-1 group (76.5%). Pulsed-field gel electrophoresis typing of a subset of ESBL-producing E. coli isolates revealed both diverse singular types and a cluster of 3 identical isolates.
**Conclusion:** Almost a quarter of private and shared latrines in an informal urban settlement in Tanzania are contaminated with extended-spectrum beta-lactamase (ESBL)-producing microorganisms, suggesting a high prevalence of human ESBL faecal carriage in the community. Shared latrines may serve as a reservoir for transmission in urban community settings in Tanzania.
**Disclosure of Interest**
None Declared

### P271
### Resistance of enterobacter cloacae to third generation cephalosporin due to hyperproduction of ampc beta-lactamases induced by exposure to piperacillin/tazobactam

André Silva-Pinto[1,2,3], Nuno Rocha-Pereira[1,2,3], Elisabete Ricardo[4], Cidália Pina-Vaz[4,5], Carlos Lima-Alves[1,2,3]
[1]Unit of Infection and Antimicrobial Resistance Control and Prevention; [2]Infectious Diseases, Centro Hospitalar São João; [3]Faculty of Medicine, Porto, Portugal; [4]Microbiology, Faculty of Medicine; [5]Clinical Pathology, Centro Hospitalar São João, Porto, Portugal
**Correspondence:** André Silva-Pinto
*Antimicrobial Resistance and Infection Control* 2017, **6(Suppl 3):**P271

**Introduction:** *E. cloacae* is a natural chromosomal producer of AmpC. Resistance to 3rd generation cephalosporin (3GC) may occur when AmpC is hyperproduced. This phenomenon is well established after selective pressure by 3GC. Nevertheless, only one case reports the association of piperacillin/tazobactam (PT) exposure.
**Objectives:** Description of a case report of induction of AmpC hyperproduction after selective pressure by PT.
**Methods:** The AmpC hiperproduction was investigated on the second isolate of *E. cloacae* using: test disk assay with MAST disks AmpC Detection Set D69C and AmpC multiplex PCR according to FJ Pérez-Pérez *et al (1)*. PCR amplification products were analysed in an agarose gel 1% stained with ethidium bromide and UV visualized. Positive control strains containing the plasmid-mediated genes DHA, EBCM and FOX were used.
**Results:** A 24 years-old man was admitted to hospital due to severe trauma with degloving of the lower limb. On day 7, PT was started due to fever, leucocytosis, C reactive protein elevation and purulent exudate from the wounds. In a surgical biopsy of the wounds, *E. cloacae* was identified with resistance to ampicillin, amoxicillin/clavulanate and 2nd generation cephalosporin. In the next 25 days, he was on PT and several surgical debridements were performed. On day 32, *E.cloacae* was again identified from a surgical biopsy with new resistance to 3GC and ertapenem (sensitive to cefepime, imipenem and meropenem). The antibiotic was changed to cefepime and the patient had good evolution. The phenotypic assay showed positive results for the production of AmpC β-lactamases. However, in the multiplex PCR no target gene amplification occurred, conversely to the positive controls, so no plasmidic AmpC is present.
**Conclusion:** This case report illustrates the possible induction of AMPc hyperproduction not plasmid-mediated but, chromosomal *AmpC* group due to selective pressure with PT, raising concerns about the use of PT to treat *E. cloacae* infections.

**References**
1-J Clin Microbiol 40:2153
Disclosure of Interest
None Declared

### P272
### Molecular detection of beta-lactamase-mediated resistance in salmonella species isolated from slaughtered swine
Alyzza Marie B. Calayag[1,2], Phyllis Anne P. Paclibare[1], Pauline Dianne M. Santos[1], Kenneth W. Widmer[3], Windell L. Rivera[1,2]
[1]Institute of Biology; [2]Natural Sciences Research Institute, University of the Philippines, Quezon City, Philippines; [3]International Environmental Research Center, Gwangju Institute of Science and Technology, Gwangju, Korea, Republic Of
**Correspondence:** Alyzza Marie B. Calayag
*Antimicrobial Resistance and Infection Control* 2017, **6(Suppl 3):**P272

**Introduction:** Extensive use of antimicrobials in livestock production has often been investigated and linked to the spread of antimicrobial resistance. This has led to the regulation and ban of antimicrobial growth promoters (AGPs) in many countries. However in most Southeast Asian countries, AGPs are still being used. In the Philippines, the swine industry contributes to the largest volume of livestock production. Penicillins, in particular, are one of the most commonly used AGP in swine production.
**Objectives:** In the present study, we aimed to screen *Salmonella* spp. isolates for transmissible ß-lactamase-encoding genes.
**Methods:** A total of 191 *Salmonella* spp. cultures isolated from slaughtered swine were revived. The isolates were collected from freshly slaughtered swine from six different abattoirs in Metro Manila, Philippines during 2013-2014. Multiplex PCR was used to screen for the three most common *bla* genes, $bla_{CTX-M}$, $bla_{SHV}$, and $bla_{TEM}$. Isolates harboring the $bla_{CTX-M}$ gene were further subtyped using multiplex PCR.
**Results:** Out of 191 *Salmonella* spp. isolates, 120 and 9 carried $bla_{TEM}$ (62.8%) and $bla_{CTX-M}$ (4.7%), respectively. Of the latter, six were classified under subtype CTX-M-1 and three were under subtype CTX-M-2. Co-carriage of the $bla_{CTX-M}$ and $bla_{TEM}$ genes was observed in 14 (7.3%) isolates. The gene $bla_{SHV}$ was not found in any of the isolates.
**Conclusion:** We report here the incidence of the most common *bla* genes in *Salmonella* spp. isolated from slaughtered Philippine swine. To our knowledge, this is the first report of $bla_{CTX-M}$-carrying *Salmonella* spp. from non-clinical samples in the Philippines.
**Disclosure of Interest**
None Declared

### P273
### Antibiotic resistance trends of salmonella enterica serotype typhi in Zimbabwe (2012 – 2017)
Tapfumanei Mashe[1,2,3], Muchaneta Mugabe[3], Babill Stray-Pedersen[1,4], Andrew Tarupiwa[3], Karen H. Keddy[5,6], Anthony M. Smith[5,6], Sekesai Zinyowera[3], Shannon L. Smouse[6], Arvinda Sooka[6], Joshua Mbanga[2]
[1]Letten Foundation Research Centre, Harare; [2]Department of Applied Biology and Biochemistry, National University of Science and Technology, Bulawayo; [3]Bacteriology, National Microbiology Reference Laboratory, Harare, Zimbabwe; [4]Division of Women, Rikshospitalet, Oslo University Hospital and Institute of Clinical Medicine, University of Oslo, Ohio, Norway; [5]Faculty of Health Sciences, University of the Witwatersrand; [6]Centre for Enteric Diseases, National Institute of Communicable Diseases, Johannesburg, South Africa
**Correspondence:** Tapfumanei Mashe
*Antimicrobial Resistance and Infection Control* 2017, **6(Suppl 3):**P273

**Introduction:** The actual burden of typhoid fever is underestimated especially in developing countries where most of the cases go undiagnosed. Resurgence of typhoid fever in Zimbabwe was reported in January 2010, mainly affecting two densely populated suburbs of Harare: Mabvuku and Tafara. The official cumulative figures between January 2012 and 22 March 2017, were 13769 suspected cases, 396 confirmed cases and 40 deaths.
**Objectives:** The objectives of the study were to determine the susceptibility of *Salmonella* Typhi isolates to antimicrobial agents presently used in the treatment of typhoid fever and to perform molecular sub-typing of ciprofloxacin-resistant *Salmonella* Typhi strains.
**Methods:**
A total of 285 *Salmonella* Typhi strains isolated from blood and stool cultures of typhoid fever patients between 2012 and 2017 were confirmed as *Salmonella* Typhi serologically and by Polymerase Chain Reaction of the STY 0201 gene. Antimicrobial susceptibility testing of these 285 isolates was performed and molecular epidemiological analysis using Pulsed-field gel electrophoresis (PFGE) analysis was performed.
**Results:** A total of 252 isolates (88. 4%) were resistant to two or more drugs including ciprofloxacin, chloramphenicol and ampicillin. Eleven multi-drug resistance patterns were observed and the most common pattern, resistotype A (resistance to ampicillin-chloramphenicol) was exhibited by 181 (63.5%) isolates. An increase in ciprofloxacin resistance from 4.2% (2014) to 21.4% (2016) was observed. *Salmonella* Typhi isolates were 100% susceptible to ceftriaxone and azithromycin. It was also observed that the ciprofloxacin-resistant isolates circulating in Harare showed a common PFGE pulsotype.
**Conclusion:** There is emerging antimicrobial resistance to ciprofloxacin in Zimbabwe. A better understanding of how the molecular epidemiology relates to the geographic distribution of typhoid fever may greatly contribute to the prevention and control of outbreaks through comprehensive and integrated strategies based on scientific evidence.
**Disclosure of Interest**
None Declared

### P274

#### Risk factors associated with faecal carriage of extended-spectrum b-lactamase (esbl) producing enterobacteriaceae in hospitalised children in Harare, Zimbabwe

Céline Gardiol[1,2], Alexander Aiken[1], Marcelyn Magwenzi[3]
[1]London School of Hygiene and Tropical Medicine, London, United Kingdom; [2]Office fédéral de la santé publique, Berne, Switzerland; [3]Department of Medical Microbiology, University of Zimbabwe, Harare, Zimbabwe
**Correspondence:** Céline Gardiol
*Antimicrobial Resistance and Infection Control* 2017, **6(Suppl 3):**P274

**Introduction:** The prevalence of *Enterobacteriaceae* with Extended-spectrum β-lactamases (ESBL) and other forms of resistance is increasing worldwide but little information is available from African countries. Previous work in Zimbabwe identified a very high ESBL resistance carriage rate amongst paediatric hospital admissions in Harare[1].
**Objectives:** We aimed to identify risk factors associated with faecal carriage of ESBL-production in enteric bacteria in this population.
**Methods:** A case-control study was conducted in children being admitted into three paediatric wards at a tertiary-referral hospital in Harare, Zimbabwe, between June and August 2016. Rectal swabs or faecal samples were collected within 24 hours of admission. CLSI disc-based methods were used to detect and confirm carriage of ESBL-producing *Enterobacteriaceae*. Risk factors were assessed using a standardized questionnaire.
**Results:** One hundred and twenty-nine children were recruited, of whom 73% were less than 5 years old. Faecal carriage of ESBL-producing *Enterobacteriaceae* was confirmed in 52 (40%) of the children. Sixty-seven (52%) children had been treated with antibiotics during the past 12 weeks and this was found to be associated with faecal carriage of ESBL (Odds Ratio 2.65, 95%CI 1.21 - 5.79). There was an increased risk of ESBL carriage in the 1-4-year-old age group (OR 2.59, 95% CI 1.08 – 6.23), in comparison with the baseline group of those less than 1 year. No significant association was found between ESBL resistance colonization and recent hospitalization.
**Conclusion:** Use of antibiotics prior to hospitalization was common and this was a risk factor for colonization with this clinically-relevant form of antibiotic resistance. The frequency of colonization with ESBL-producing *Enterobacteriaceae* in this paediatric population in Zimbabwe is of concern. Underlying reasons for this remain poorly understood and further investigation is urgently needed in order to inform preventive measures against further development or spread of ESBL resistance.

[1]Magwenzi
et al. Carriage of antibiotic-resistant Enterobacteriaceae in hospitalised children in tertiary hospitals in Harare, Zimbabwe. Antimicrobial resistance and Infection Control (2017) 6:10

Disclosure of Interest
None Declared

### P275

#### Diversity index is strikingly efficient in prediction of healthcare-associated infections outbreaks

Elena B. Brusina
Department of Epidemiology, Kemerovo State Medical University, Kemerovo, Russian Federation
*Antimicrobial Resistance and Infection Control* 2017, **6(Suppl 3):**P275

**Introduction:** Evolution and spreading of multidrug-resistant strains lead to the outbreaks of healthcare-associated infections (HAIs) with a high case fatality rate; hence, the search for efficient predictors of these outbreaks is rapidly ongoing.
**Objectives:** To determine predictors of outbreaks caused by hospital strains of bacteria.
**Methods:** Having performed a cohort study of 152 HAI outbreaks in 38 Russian hospitals from 1983 to 2016, we isolated 7,352 pathogens following evaluation of antimicrobial resistance and pathogenicity and sequence typing.
**Results:** Using cluster analysis, we stratified all pathogens to four clusters. Among all pathogens, *Salmonella typhimurium, Salmonella infantis, Enterococcus spp.* and *Pseudomonas aeruginosa* demonstrated the highest epidemic potential, evolving to hospital strain as early as 7-28 days before the beginning of the outbreak. *Klebsiella pneumoniae, Staphylococcus aureus, Acinetobacter spp.* and *Serratia marcescens* evolved to hospital strain 67, 93, 112 and 129 days before the beginning of the outbreak, respectively. Pathogen species, length of hospital stay, antimicrobial resistence at baseline, proportion of patients with purulent infections, heterogeneity of diseases within the hospital unit, type of the hospital unit, and contact between the patients were the main factors affecting evolution of hospital strains. Colonization of the gut preceded HAIs in all cases. Strikingly, diversity index ≤ 0.4 was the ultimate predictor of the outbreaks.
**Conclusion:** Monitoring of bacterial diversity demonstrated a high efficiency in control HAIs.
**Disclosure of Interest**
None Declared

### P276

#### The first romanian study concerning the prevalence of mrsa and ESBL gram-negative bacteria among hospitalized patients

Andreea Moldovan[1], Laura Dobrin[2], Mihai Capilna[3], Ioan Cotoara[1], Bogdan Moldovan[3]
[1]Prevention and control of infections; [2]Microbiology, [3]General Surgery and Gynecology, Sf Constantin Hospital, Brasov, Romania
**Correspondence:** Andreea Moldovan
*Antimicrobial Resistance and Infection Control* 2017, **6(Suppl 3):**P276

**Introduction:** This six-year study represents a premiere in the Romanian medical system - the results of the first study concerning the prevalence of methicilin-*resistant Staphylococcus aureu*s (MRSA) and extended spectrum betalctamases (ESBL) gram-negative rods colonization among patients, using an active surveillance culturing. We studied, in the same time, the influencing factors of MRSA and ESBL colonization and the efficiency of the strategies for controlling the MRSA, ESBL spread.
**Objectives:** The final aim of the study was to correctly identify and manage the nosocomial infections, in particular those related to the colonizers.
**Methods:** The study was conducted in St.Constantin Hospital, a private medical establishment with surgical and oncological ward (91 beds), between April 2011- March 2017. All the patients were screened for this colonization prior to admission or, in case of emergency hospitalization, at the admission. We applied the epidemiological strategies for controlling the multidrug resistant bacteria spread, focusing first the patients' isolation and barrier precautions, in parallel with complementary hand hygiene methods and rules, environmental cleaning and disinfection, education of the patients and continuous medical training of the medical staff and observational studies.
**Results:** Among 14 519 patients screened both for MRSA and ESBL, the rate of prevalence for the colonistaion was 8,29% for MRSA, 7,14% for ESBL and 1,06% had a double colonisation. The main risk factors vary for surgical and oncological ward. Applying the infection control measures, starting from the colonisation, we noticed a statistically semnificative improvement on the prevalence of nosocomial infections.
**Conclusion:** Taking into account every link of the nosocomial infections chain of transmission (active screening, hand hygiene, hospital hygiene, infection control precautions, a strong antibiotic stewardship policy, training of the medical staff, patients, and visitors) will provide a better medical act, decreasing the rate of NI and other complications involving the medical care, including the improvement of morbidity and mortality index.
**Disclosure of Interest**
None Declared

### P277

#### Multidrug resistant organisms (MDRO): information outside hospital

Ilda Devesa[1], Alexandra Fernandes[1], David Peres[1], Fernanda Vieira[1], Isabel Neves[1,2]
[1]Infection Control and Antimicrobial Resistance Unit; [2]Infectious Diseases Unit, Matosinhos Local Health Unit, Matosinhos, Portugal
**Correspondence:** Ilda Devesa
*Antimicrobial Resistance and Infection Control* 2017, **6(Suppl 3):**P277

**Introduction:** New technologies and information systems facilitates MDRO reporting. Sharing MRDO information increases the effectiveness of epidemiological surveillance and promotes the control of its dissemination. Our facility is constituted by an acute hospital with 370 beds and serves a population of 174931 inhabitants (primary (PC) and other ambulatory care (OAC). Using multimedia information network improves notification during hospitalization and post-discharge (PC, OAC, home care facility (HCF) or hospital, in our geographic area or neighbors).
**Objectives:** To highlight the importance of new information technologies in the communication of MDRO at various healthcare levels, enabling the beginning of infection control measures timely.
**Methods:** Design an information circuit for MDRO positive patient:1. Issuance of alert from Microbiology laboratory (centralized) to Infection Control Team and result availability with alert in the clinic process; 2. If PC or OAC, we inform the Health Unit that assists the patient (family doctor and nurse); 3. If patient has been transferred, we inform this unit (hospital, HCF). Some MDRO are notified to the Regional Coordination Group.
**Results:** The data on notifications were collected from an internal database that enables the network (started in 2012). The number of registrations had increase in 2012 to 2016; were 125 notifications (76 PC, 12 HCF, 4 H, 33 OAC) and 180 (59 PC, 11 HCF, 12 hospital, 98 OAC) in 2012 and 2016 respectively. In 2012 the most frequently reported MRDO were *Enterobacteriaceae* ESBL+ (66%) and MRSA (15%); in 2016 *Enterobacteriaceae* ESBL+ (68%), carbapenemase-producing *Enterobacteriaceae* (23%) and MRSA (5,5%).
**Conclusion:** The implementation of MDRO alert system is essential in Infection Control and contributes to more effective measures, which can reduce healthcare(HCI) associated infection and the spread of antimicrobial resistance (AR). Access to data in real time, sending the alert to health professionals, articulation with the various levels of care are essential factors for the success of this type of surveillance system. The influence of this circuit in HCI and AR is under evaluation.
**Disclosure of Interest**
None Declared

## Multidrug-resistant *Acinetobacter* and *Pseudomonas*

### P278

#### Prevalence and antimicrobial resistance of pseudomonas aeruginosa isolates in pediatric cardiac intensive care unit

Nelya Bissenova, Aigerim Yergaliyeva
Laboratory of Microbiology, National Scientific Medical Research Center, Astana, Kazakhstan
**Correspondence:** Aigerim Yergaliyeva
*Antimicrobial Resistance and Infection Control* 2017, **6(Suppl 3):**P278

**Introduction:** Pseudomonas aeruginosa is important cause of serious infections in newborns and young children that are associated with higher mortality.
**Objectives:** We aimed to determine dynamic of resistance rate of P.aeruginosa strains isolated from patients in pediatric intensive care unit.
**Methods:** A total of 264 strains of P.aeruginosa isolated from clinical samples (blood, respiratory tract, wound samples and others) of 877 patients of pediantric cardiac ICU (infants and children the first three years) after cardiac surgery (surgery on the heart and major blood vessels) from January 2014 to December 2016 were included to the prospective microbiologic study. Identification and susceptibility testing were performed using the Vitek 2 automated system.
**Results:** During the study period the frequency of detection of P.aeruginosa strains from clinical samples increased from 5.7% in 2014 to 9.2% ($p < 0.05$) in 2016 (from total number of isolates). The most isolates were allocated from respiratory tract (96.5%). Resistance rate increased to meropenem from 37.7% in 2014 to 76.7% in 2016 ($p < 0.001$), to ceptazidime from 40.8% to 61.4% ($p < 0.02$), to cefepime from 29.3% to 61.4% ($p < 0.001$), to ticarcillin/clavulanic acid from 22.2% to 51.2% ($p < 0.01$), to gentamicin from 25% to 60.5% ($p < 0.001$), to ciprofloxacin from 10.4% to 50% ($p < 0.001$) respectively. The lowest resistance rate were to cefoperazone/sulbactam – 42.9%; 95% CI 25.1-62.6
**Conclusion:** The results presented in this study have shown increasing resistance rates of P. aeruginosa to all antibiotics, especially to carbapenems. These suggest appropriate a strong antibiotic policy. Rapid and accurate reporting of antibiotic resistance is essential in therapeutic decisions.
**Disclosure of Interest**
None Declared

### P279

#### The hospital sanitary as a source of a vim-producing multidrug resistant pseudomonas aeruginosa outbreak at the pediatric hemato-oncology ward

E. De Brabandere[1], R Ablorh[1], I Leroux-Roels[1,2]
[1]Infection control, [2]Medical microbiology, GHENT UNIVERSITY HOSPITAL, Ghent, Belgium
**Correspondence:** E. De Brabandere
*Antimicrobial Resistance and Infection Control* 2017, **6(Suppl 3):**P279

**Introduction:** This study describes an outbreak investigation and the infection control measures in an attempt to identify the potential source and stop transmission.
**Objectives:**
**Methods:** A combination of descriptive epidemiology, microbiological sampling of the environment and molecular strain typing
**Results:** In August 2016 a two year-old immunocompromised boy developed a bacteremia with a multidrug resistant (MDR) *Pseudomonas aeruginosa* (PA) at the pediatric hemato-oncology ward. Investigation of patient data revealed that 2 other patients, hospitalized before in the same room, had positive cultures (1 bacteremia, 1 rectal colonization) with a phenotypically identical PA. Since then two other patients were colonized with this PA strain. All patients had the same room as an epidemiological link. Therefore a microbiological sampling of this room was executed. The same PA strain was found in the aerator of the tap and in the bathtub drain. Molecular typing of the MDR PA strains demonstrated the presence of a Verona integron metallobetalactamase (VIM), the strains were also shown to be genetically identical. Additional environmental investigation of the entire department revealed the contamination with (sensitive) PA of 23% of taps and 82% of drains. Multiple remedial actions were taken, such as replacing the plumbing and taps, installing point-of-use filters, disinfecting the drains with acetic acid and washing immunocompromised patients without water. Cultures of rectal samples are performed on a weekly basis and no additional patient cases have occurred. Control cultures of the environment revealed however that the drain remains a persistent reservoir of (MDR) PA. A 'PA management plan' has been initiated to investigate the source of PA in the tap water and to evaluate the most efficient strategy to decontaminate the drains in patient rooms.
**Conclusion:** A recent outbreak with MDR VIM PA in our pediatric hemato-oncology unit revealed that taps and drains can be the source of infection with waterborne pathogens, such as PA. Despite remedial actions, the drains remain an important reservoir of PA. Additional investigations are ongoing and in the meanwhile measures are taken to limit the exposure of this highly vulnerable population to PA.
**Disclosure of Interest**
None Declared

### P280

#### Analysis of suceptibility of pseudomonas aeruginosa based on ars-data within in- and outpatient care in Germany from year 2010 to 2015

Beneditta Suwono, Tim Eckmanns, Muna Abu Sin, Ines Noll, Marcel Feig, Benedikt Zacher
Robert Koch Institute, Berlin, Germany
**Correspondence:** Beneditta Suwono
*Antimicrobial Resistance and Infection Control* 2017, **6(Suppl 3):**P280

**Introduction:** ARS (*Antibiotika-Resistenz-Surveillance*) is a German laboratory-based voluntary surveillance system for AMR, which contains susceptibility data on all clinically relevant bacterial pathogens from any patient material (1)
**Objectives:** To analyze the proportion of non-susceptibility (I + R) in *P. aeruginosa* regarding both carbapenem- (CARPEN) and multi-drug-resistance (MDR) from ARS-data from 2010-2015 within in- and outpatient care in Germany.
**Methods:** All first-isolated-data, which were regularly collected from 2010-2015, were included. An MDR-isolate was defined as non-susceptible to ≥1 agent of ≤ 3 antimicrobial groups out of the following 5: penicillin(piperacillin/tazobactam),cephalosphorins(cefepime, ceftazidime),fluoroquinolones(ciprofloxacin, levofloxacin),aminoglycosides(amikacin, gentamicin, tobramycin) and carbapenems(imipenem, meropenem). Logistic regression was used to estimate the proportion, which was adjusted for age, care type, gender, materials, regions and time.
**Results:** 63,701 isolates were registered (16% non-susceptible to CARPEN and 12% MDR). The proportion increased yearly 8% for CARPEN (95% CI;adjusted(adj)$OR_{CARPEN}$ = 1.08[1.06;1.09]), and decreased 12% (adj$OR_{MDR}$ = 0.88[0.87;0.90]) for MDR isolates. Females (adj$OR_{CARPEN}$ = 0.80[0.76;0.83],adj$OR_{MDR}$ = 0.74[0.70;0.78]) and outpatients (adj$OR_{CARPEN}$ = 0.61[0.58;0.64], adj$OR_{MDR}$ = 0.54[0.51;0.58]) had lower proportions compared to males and inpatients respectively. The CARPEN and MDR isolates were most prevalent in western regions (adj$OR_{CARPEN}$ = 1.53[1.32;1.77], adj$OR_{MDR}$ = 1.17[0.98;1.41];baseline north-eastern region) and in respiratory tract specimens given baseline to swab specimens (adj$OR_{CARPEN}$ = 1.94[1.82;2.08],adj$OR_{MDR}$ = 2.04[1.88;2.22]).Patients 30-75 y.o. had twice higher proportion in CARPEN and three times higher in MDR given baseline to under 15 y.o. (adj$OR_{CARPEN}$ = 2.26[1.95;2.62]–2.06 [1.82;2.33],adj$OR_{MDR}$ = 3.19[2.63;3.88]–3.02 [2.56;3.58]).
**Conclusion:** The non-susceptibility proportion to carbapenems has increased whereas the MDR reduced over a six year period. Lower proportions were found in female and outpatient care patients. Highest proportions were found in western region of Germany and respiratory tract specimens.

**References**
(1) https://ars.rki.de/
Disclosure of Interest
None Declared

### P281

#### Lessons from an outbreak of ges-2 producing pseudomonas aeruginosa in critical care medicine (CCM)

Robert P. Stevens[1,2,3], Bety Krstevska[4], Simone Kelly[4], Justine Harris[5]
[1]Infectious Diseases, The Sutherland Hospital; [2]Faculty of Medicine, University of New South Wales; [3]Microbiology, South Eastern Laboratory Services; [4]Critical Care Medicine; [5]Clinical Services, The Sutherland Hospital, Sydney, Australia
**Correspondence:** Robert P. Stevens
*Antimicrobial Resistance and Infection Control* 2017, **6(Suppl 3):**P281

**Introduction:** In January 2014 a previously healthy 61yo female was transferred from CCM at The Sutherland Hospital (TSH) to a referral centre for treatment of Acute Myeloid Leukaemia. Three weeks later, and despite multiple anti-infectives, she developed overwhelming sepsis and succumbed. Two sets of ante-mortem blood cultures were positive for a multiresistant *P. aeruginosa* susceptible to amikacin, aztreonam and polymixin B, but resistant to gentamicin and all beta-lactams including carbapenems - a resistance phenotype not previously seen at that centre.
**Objectives:** To assess whether an outbreak of multiresistant P. aeruginosa had occurred in CCM and terminate it.
**Methods:** Interrogation of the laboratory database for isolates with similar antibiogram. Utilization of molecular methods to characterize resistance mechanisms.
Active screening of patients and the environment in CCM. Multiple interventions to ensure termination of any outbreak.
**Results:** Testing of the initial isolate by polymerase chain reaction (PCR) revealed the presence of a GES-2 gene. 7 additional patients from TSH over an 8 month period had clinical samples positive for a *P. aeruginosa* with identical resistance phenotype - found to harbour the GES-2 gene - strongly suggestive of an outbreak. All patients had been admitted to CCM at TSH - the only epidemiological link.
Active screening of patients and the environment in CCM revealed a *P. aeruginosa* of identical phenotype from a hand basin.
**Conclusion:** Earlier outbreak recognition could have led to earlier intervention and may have prevented the associated fatality. Recognition was delayed by ad hoc laboratory reporting and limited epidemiological oversight. Use of molecular methods to characterise resistance mechanisms was beneficial. Clear lines of reporting and improved data collation are essential in response to an outbreak.
**Disclosure of Interest**
None Declared

### P282

#### First report of new delhi metallo-beta-lactamase-1 producingacinetobacter baumannii isolated from clinical samples in Iran

Shahnaz Armin, Fatemeh Fallah, Leila Azimi, Abdollah Karimi, Saeed Maham, Hossein SamadiKafil
Pediatric Infections Research Center, Mofid Children Hospital, ShahidBeheshti University of Medical Sciences, Tehran, Iran, Tehran, Iran, Islamic Republic Of
**Correspondence:** Shahnaz Armin
*Antimicrobial Resistance and Infection Control* 2017, **6(Suppl 3):**P282

**Introduction:** Appearance antimicrobial resistance is one of the global challengeable problems in health care systems. Carbapenem is one of the last lines of antibiotic therapy in multi-drug resistance bacteria. So, emergence of resistance to carbapenem can lead to considerable problem.
**Objectives:** The aim of this study was detection of the metallo-beta-lactamase in the isolated *Acinetobacter baumannia*s cause of serious bacterial infection.
**Methods:** In this cross sectional study 28 *Acinetobacter baumannii* isolated from clinical specimens in a teaching hospital in Tabriz from July to September 2016 were surveyed and after identification tests their Antibiotic susceptibility testing was prepared according to CLSI guide line. As most common metallo-bata- lactamase genes in *A. maumannii* ($bla_{VIM}$, $bla_{IMP}$, $bla_{NDM-1}$ and $bla_{SPM-1}$) detected by PCR assay and confirmed by sequencing.
**Results:** All of 28 isolated *A.baumannii* confirmed as a carbapenem resistant *A. baumannii* strains were detected according to conventional biochemical and microbiological tests in this study. The most of resistance has been observed to imipenem in collected strains and the most effective antibiotic was colistin. $bla_{NDM-1}$was observed in 3 (11%) isolated *A. baumannii*. Other tested metallo-bata- lactamase genes have been not observed.
**Conclusion:** Regard of our knowledge this manuscript is the first report of NDM-1 producer *A. baumannii* in Iran.We found NDM-1 as a responsible gene for producing carbapenem resistance in these strains.Unfortunately, NDM-1 producing gram-negative bacteria can be resistance to all beta-lactam antibiotics and make important complications in health care systems. So, detection of NDM-1 producing strains is the alarming sign for health care system in Iran.

**Disclosure of Interest**

None Declared

### P283

#### Performance of acinetobacter baumannii screening among patients admitted in icu at a swiss tertiary care hospital

Alain Kenfak Foguena, Dominique Blanc, Antony Croxatto, Jean-Luc Pagani, Laurence Senn
CHUV, Lausanne, Switzerland
**Correspondence:** Alain Kenfak Foguena
*Antimicrobial Resistance and Infection Control* 2017, **6(Suppl 3):**P283

**Introduction:** *Acinetobacter baumannii* represents a major concern in intensive care units (ICU). Since the occurrence of a multidrug resistant *A. baumannii* outbreak in our burn unit in 2002-2004 (Zanetti et al. *Infect Control Hosp Epidemiol.* 2007), we implemented a systematic screening of high risk patients: sanitary repatriation from abroad, hospitalization abroad during the previous year or visit of the Indian subcontinent the previous year. However, we sometimes face patients presenting with *A. baumannii* in clinical samples, despite negative screening on admission.
**Objectives:** The aim of this study was to evaluate the performance of our screening program.
**Methods:** All high risk patients screened for *A. baumannii* on admission into the ICU of the Lausanne University Hospital were included. Screening consisted in rectal swab, throat swab, urine, bronchial sampling and wound swab if any. Cultures were performed using a selective broth containing sodium acetate as the only carbon source. To determine the performance of our screening strategy, we further classified patients according to the presence or absence of *A. baumannii* in clinical sample during their stay in ICU.
**Results:** From 2010 to 2016, 76 patients were screened on admission, 13 (17.1%) of whom were colonized with *A. Baumannii*. Clinical samples during hospitalisation revealed *A. Baumannii* in three (4.8%) other patients 1, 6 and 20 days after admission. This interval may suggest the need of repeating screening in high risk patients.
Despite local guidelines, the number of screened sites was inconstant. Overall, in the 16 infected or colonised patients the performance of the screening was different among sites: respiratory samples (60%, 3/5), throat (53%, 8/15), rectum (62%, 8/13) and urines (33%, 2/6).
**Conclusion:** Our results showed that no clinical site is preferentially colonized with *A. Baumanii*, emphasizing the need to screen multiple sites (including respiratory tract). One may also consider repeating sampling in high risk patients.

**Disclosure of Interest**

None Declared

### P284

#### Risk factors for death among patients with bloodstream infections caused by carbapenem-resistant Acinetobacter baumannii

Gilberto G. Gaspar, Mayra G. Menegueti, Ana E. R. Lopes, Magda F. I. Silva, Roberto Martinez, Fernando Bellissimo-Rodrigues
Medical School, University of São Paulo, Ribeirão Preto, Brazil
**Correspondence:** Gilberto G. Gaspar
*Antimicrobial Resistance and Infection Control* 2017, **6(Suppl 3):**P284

**Introduction:** Multidrug-resistant (MDR) bacteria has become a major public health concern, and carbapenem-resistant *Acinetobacter baumannii* is an emergent MDR microorganism in the healthcare setting, associated with a high risk of fatal outcome. To date, it is not completely understood which are the main risk factors for death among patients infected by this pathogen.
**Objectives:** To evaluate potential risk factors for death among hospitalized patients with bloodstream infections caused by carbapenem-resistant *A. baumannii*.
**Methods:** Prospective cohort study conducted in a tertiary-care university hospital, from January 2015 to July 2016. We included all patients who presented a blood culture positive to carbapenem-resistant *A. baumannii* along with clinical symptoms of systemic infection, during the study period. Selected clinical and demographic characteristics were evaluated as possible risk factors on the patients' medical records. We used the Two-tailed Fisher's exact and the Mann Whitney tests to analyze the data.
**Results:** We included 31 patients during the study period, 21 (67.7%) of them suffering in-hospital death, and 10 of them being discharged alive from the hospital. The following variables yielded similar values among those who survived and those who died, respectively: median age (52 vs. 52 years, $p = 0.685$), length of stay prior to the infection (16.5 vs. 16.0 days, $p = 0.543$); time between the onset of symptoms and introduction of adequate treatment (1 vs. 3 days, $p = 0.246$), intensive care unit admission (25.0% vs. 57.9%, $p = 0.077$); submitted to surgical procedure (58.3% vs. 52.6%, $p = 0.525$), use of immunosuppressive drugs (41.7% vs. 52.6%, $p = 0.716$), and use of combined antimicrobial therapy (88.9% vs. 85.7%, $p = 1.000$). The only factor marginally associated with the outcome in-hospital death was the time to blood culture become positive, after sampling (633 vs. 450 min., $p = 0.057$), which may be related to the level of bacterial load in the bloodstream.
**Conclusion:** A lower time to blood culture become positive may be a potential marker of poor prognosis among patients with bloodstream infections caused by A. *baumannii.*

**Disclosure of Interest**

None Declared

### P285

#### Attributable mortality rates and sepsis scores of patients with extensive drug-resistant Acinetobacter baumannii bacteremia

Wison Laochareonsuk, Pawin Wanasitchaiwat, Woranan Boonsomsuk, Tharntip Sangsuwan[1], Silom Jamulitrat, on behalf of MedPSU
Community Medicine, Prince of Songkla University, Hat Yai, Thailand
**Correspondence:** Tharntip Sangsuwan
*Antimicrobial Resistance and Infection Control* 2017, **6(Suppl 3):**P285

**Introduction:** During the past decades, extensive drug-resistant *Acinetobacter baumannii* has emerged as an important opportunistic nosocomial pathogen but the virulence of this strain has remained controversial
**Objectives:** To determine mortality rates and sepsis scores of patients with extensive drug-resistant *A. baumannii* bacteremia
**Methods:** A retrospective study was conducted in adult patients (age > 15 years) admitted to Songklanagarind hospital during 2009 and 2015 with blood culture positive for *A. baumannii* after 3 days of admission. Antimicrobial resistance was categorized into four levels comprising: non-multidrug resistance (nonMDR), multidrug-resistant (MDR), extensively drug-resistant (XDR), and possible pandrug-resistant (possiblePDR). Severity of underlying disease of the patients immediately before onset of bacteremia was determined by sequential organ failure (SOFA) score and American Association of Anesthesia (ASA) score. Virulence of *A. baumannii* was assessed in term of sepsis score and in hospital mortality rate
**Results:** The study identified 38, 110, 168, and 14 cases of bacteremia caused by nonMDR, MDR, XDR, and possiblePDR respectively. After adjusting for confounding effect by using Cox proportional hazard model, mortality rates attributable to *A. baumannii* was significantly associated to levels of drug resistance. Using nonMDR as a reference, the incidence rate ratios and corresponding 95% confidence intervals (95%C.I) of MDR, XDR, and possiblePDR were 2.3 (95%C.I = 0.9-4.9), 3.1 (95%C.I = 1.4-7.0), and 1.9 (95%C.I = 0.6-5.5) respectively
**Conclusion:** Mortality rate severity of sepsis increase significantly in XDR *A. baumannii*

**Disclosure of Interest**

None Declared

### Community-acquired antimicrobial resistance and healthcare-associated infections (HCAI)

#### P286

**Bacteriological profile and antibiotic resistance pattern of urinary tract bacterial pathogens among isolates at ethiopian public health institute bacteriology national reference laboratory, (2014-2015)**

Abebe Aseffa Negeri, on behalf of Clincal Bacteriology
Addis Ababa, ETHIOPIAN PUBLIC HEALTH INSTITUTE, Addis Ababa, Ethiopia
**Correspondence:** Abebe Aseffa Negeri
*Antimicrobial Resistance and Infection Control* 2017, **6(Suppl 3):**P286

**Abstract video clip: Abstract**

**Background:** Urinary tract infection is one of the most commonly encountered diseases in human population next to respiratory and gastrointestinal infections. It affects different parts of urinary tract system in which females are more prone to the infection. Urinary tract infection is caused by various ranges of bacterial pathogens. Antibiotics resistances due to these pathogens are constantly evolving.

**Objective:** To determine the etiological Bacterial pathogens of the UTI and their antibiotic resistance pattern.

**Methods:** It was retrospective study undertaken over two years (from January 2014-December 2015) at Ethiopian public health institute, clinical Bacteriology and Mycology National referral laboratory, using the laboratory record book of test results and patient bio-data. Pathogens were identified using standard microbiological methods and disc diffusion techniques were used for antibiotics susceptibility testing. The laboratory data was compiled and analyzed using Microsoft Excel.

**Results:** Among a total of 882 samples analyzed, the overall prevalence of urinary tract infection was 30.5%(269). Of patients with culture positive, females were accounted for 68% (183) while male patients were 32%(86), *Escherichia coli* was the most predominant bacterial pathogens isolated 48.3% (130) (followed by *Klebseilla spp* 13% (35), Coagulase negative staphylococcus 33 (12.8%), *Staphylococcus aureus* 28 (10.4%). Gram negative bacteria pathogens showed extreme resistance to ampicillin (92.3%) and less resistance to Amikacin (6.4%) while the resistance rate of Gram positive bacteria isolated in this study was range between 0% for vancomycin and 85.7% for cotrimoxazole.

**Conclusion:** The study profiled high prevalence of urinary tract infection causing bacteria pathogens and showed increasing antibiotic resistance pattern among the isolates. This leaves the clinicians with very few alternative options of drugs for the treatment of UTI. Periodic evaluation of predominant pathogens and their resistance pattern as it changes on with time.

**Key words**: Urinary tract Infection, Bacterial profile, Antibiotic resistance

**Disclosure of Interest**
None Declared

#### P287

**Molecular characterization and integron mediated antimicrobial resistance from diarrheagenic E.Coli isolates in India**

Taru Singh, Shukla Das
MICROBIOLOGY, UCMS & GTBH, Delhi, India
**Correspondence:** Taru Singh
*Antimicrobial Resistance and Infection Control* 2017, **6(Suppl 3):**P287

**Introduction:** Integrons are mobile DNA elements that capture and express genes found in their environment as part of small mobile elements, termed gene cassettes. Class 1 integrons was found to be more prevalent as compared to other classes of integrons. Mutations causing quinolone resistance occur primarily in a highly conserved region of the quinolone resistance-determining region [QRDR] of DNA gyrase and topoisomerase IV.

**Objectives:** To gain insight on the distribution of genes encoding class 1, 2 and 3 integrons among diarrheagenic *E.coli* isolates and gene cassettes associated with them. To determine antibiotic resistant genes and to determine the single nucleotide polymorphism (SNP) in gyrA and parC in quinolone resistance determining regions from children upto five years of age from Delhi, India. An attempt was also made to create a 3D model and find a suitable inhibitor using an in silico study.

**Methods:** A total of 120 *E.coli* isolates including 80 diarrheagenic *E.coli* (cases) and 40 healthy isolates (controls) were recruited in this study. Fresh stool samples were collected and identified as *E.coli* using biochemical tests. Antibiotic susceptibility testing was performed according to CLSI guidelines. Extracted DNA was used for conventional PCR for identification of genes associated with virulence, class 1, 2 and 3 integron, gene cassettes, antibiotic resistance genes and SNP detection. DNA sequence analysis was performed and sequences were deposited in NCBI's BankIt submission database.

**Results:** Class 1 integron was identified in 43 and 9 isolates while 12 and 7 isolates harbored class 2 integron in diarrheagenic cases and healthy controls respectively and no class 3 integrons were detected in any of the isolate. 9 and 3 isolates showed co existence of class 1 and class 2 integrons in diarrheagenic cases and healthy controls.

**Conclusion:** PCR sequence analysis of the quinolone resistance-determining region (*gyrA/B, parC*) revealed mutations. Ciprofloxacin was found to be suitable inhibitor of DHFR. Comparative modeling is a useful tool in bioinformatics to predict the three dimensional (3D) structure of an unknown protein.

**Disclosure of Interest**
None Declared

#### P288

**Ciprofloxacin resistance among neisseria gonrhea isolates obtained from genital samples referred to Ethiopian Public Health Institute**

Surafel F. Dinku[1], Tamrat Tadesse[2], Teklil Biza[1], Rajiha Abubeker[1], Negga Asamene[1], Amete Mihret[1], Degefu Beyene[1]
[1]Clinical Bacteriology and Mycology; [2]ETHIOPIAN PUBLIC HEALTH INSTITUTE, Addis Ababa, Ethiopia
**Correspondence:** Surafel F. Dinku
*Antimicrobial Resistance and Infection Control* 2017, **6(Suppl 3):**P288

**Introduction:** Gonococcus is a major public health challenge due to the high frequency of infections accompanied by in adequate treatment options. This study focuses on determining of prevalence and antimicrobial resistance of gonococcal isolates referred to the Ethiopia Public Health Institute.

**Objectives:** To detemine the gonococcal ciprofloxacine resistance patterm

**Methods:** Seven hundred thirty five gonococcal isolates were obtained from patients referred to Ethiopian Public Health Institute for genital culture and sensitivity test between 2008 and 2014. All suspected gonococcal isolates identification and antimicrobial resistance tests were confirmed using standard microbiology techniques.

**Results:** Five hundred eight gonococcal isolates 508/735 (69%) were resistant for ciprofloxacin, which is the current choice of antibiotics used for syndromic management of urethral discharge syndrome. The resistance pattern of gonococcal isolates for other candidate antibiotics, were spectinomycin 3.2%, cefoxitin 0%, and ceftriaxone 4%.

**Conclusion:** Ciprofloxacin resistance above acceptable range and not recommended to use alternative candidate antibiotics for syndromic management

**Disclosure of Interest**
None Declared

### P289

#### Using edetection for finding missing tb patients in low –resource settings

Shelly Batra, Sonali Batra, Sandeep Ahuja
OPERATION ASHA, New Delhi, India
**Correspondence:** Sonali Batra
*Antimicrobial Resistance and Infection Control* 2017, **6(Suppl 3):**P289

**Introduction:** Finding the missing TB patients is of paramount importance to help the world achieve its target of ending the TB epidemic by 2030.
**Objectives:** The objective of the study was to find the impact on detection rates of TB patients with the use of technology
**Methods:** Hence Operation ASHA has developed and piloted an android based software application- eDetection, that improves detection by identifying patients early, enrolling them in treatment and reducing chances of infection to other individuals. This application is loaded on a standard handheld android tablet through google play and eliminates the need for expensive hardware. The health workers, during their routine work, ask community members a basic questionnaire, which is algorithm based. Depending on the results of the questionnaire, the application directs the next step, which could be: get sputum tested, visit a physician etc. Once the suspect's details are saved, the system keeps track of the suspect through each stage of diagnosis namely – Sputum Result Pending, Antibiotics Medication, Repeat Sputum Result Pending, X-ray Result Pending. The diagnosis of a suspect is termed as 'Completed', if the diagnostic results are indicative of TB. If diagnostic results are negative, the suspect is 'Archived' into the system.
**Results:** This was piloted for 4 months in Cambodia and results compared during the same months in the previous year. The detection has gone up by 20%, whereas detection in the contiguous areas had actually gone down by 48%. So there is a clear difference of 63% in detection rate with and without eDetection application.
**Conclusion:** It also helped in improving the staff productivity by 20% and reducing the treatment costs by 20%. Thus it helps in cost savings of $197 per detection resulting in a saving of $650 million in detection of all the missing 3.3 million TB patients.
**Disclosure of Interest**
None Declared

### P290

#### Evaluation of rapid assay for detection of multidrug-resistant and extensively drug-resistant mycobacterium tuberculosis

Nagwan G. El Menofy[1], Abeer K. Abdulall[1], Zeinab H. Ahmed[2], Zienab, A. Mostafa and Mohamed, S. E. Ashour
[1]Department of Microbiology and Immunology, Faculty of Pharmacy-Al-Azhar University (Girls, Cairo; [2]Department of Microbiology and Immunology, Faculty of Pharmacy-Al-Azhar University (Girls, Nasr city, Egypt
**Correspondence:** Nagwan G. El Menofy, Zienab A. Mostafa, Mohamed S. E. Ashour
*Antimicrobial Resistance and Infection Control* 2017, **6(Suppl 3):**P290

**Introduction:** Multidrug-Resistant Tuberculosis (MDR-TB) and Extensively Drug-Resistant Tuberculosis (XDR-TB) are major public health problems and present a major challenge for tuberculosis control programs.
**Objectives:** The aim of this study was to evaluate the colorimetric method; resazurin micro titer assay (REMA) for rapid detection of *Mycobacterium tuberculosis* (*M. tuberculosis*) resistance to anti TB drugs.
**Methods:** This study was cross-sectional in design**.** Drug susceptibility testing was carried out on 50 *M. tuberculosis* isolates isolated from sputum specimens by culturing on Löwenstein–Jensen (L-J). Detection of *M. tuberculosis* resistance to INH, RIF, OFX and KAN were performed by proportion method (PM) on L-J media and by REMA plate method which use the oxidation–reduction colorimetric indicator resazurin and had been proposed for the determination of drug resistance and minimal inhibitory concentration of anti TB drugs against *M. tuberculosis*. Data was analyzed using IBM SPSS advanced statistics version 21 (SPSS Inc., Chicago, IL).
**Results:** Drug susceptibility testing by PM revealed that 28 (56%) isolates were resistant to INH, 22 (44%) isolates were resistant to RIF, and 7 (14%) isolates were resistant to OFX and 2(4%) were resistant to KAN**.** It was found that 20(40%) isolates were MDR-TB while no XDR-TB isolates were detected. Results by REMA plate method were compared to the PM considered the PM as gold standard. The agreement between the two methods for detection of drug resistance to INH, RIF, OFX, and KAN was found to be 98%, 96%, 98%, and 100% respectively. The sensitivity and specificity of REMA plate method was 85% and 100% respectively.
**Conclusion:** REMA plate method showed high agreement, very good sensitivity and specificity compared with the PM, which took several weeks to confirm the results and might represent an inexpensive procedure for rapid detection of resistance to first and second line anti-TB drugs in low-resource countries.
**Disclosure of Interest**
None Declared

### P291

#### Withdrawn

### P292

#### Prevention strategies on healthcare-associated infections in outpatient care - a systematic review

Diana Neves[1], Qiao Fu[2], Walter Zingg[3]
[1]Infection Control Programme, Hospital Santa Maria Centro Hospitalar de Lisboa Norte, Lisbon, Portugal; [2]Infection Control Programme, West China Hospital, Sichuan University, Chengdu, China; [3]Infection Control Programme, University of Geneva Hospitals, Geneva, Switzerland
**Correspondence:** Diana Neves
*Antimicrobial Resistance and Infection Control* 2017, **6(Suppl 3):**P292

**Introduction:** There is little evidence about the prevention of healthcare-associated infections (HAIs) in outpatient care and no systematic review has been performed on this subject.
**Objectives:** To assess the scope and impact of reported HAI-prevention strategies in outpatient care.
**Methods:** A systematic review was performed based on the PRISMA guidelines. Studies referring directly or indirectly to the prevention of HAI or transmission of pathogens, reporting prevalence or incidence outcome data in any patient undergoing medical care in an outpatient setting or at home were eligible for analysis. Medline, Embase, the Cochrance database and the outbreak database were searched for reports published between January 1996 to July 2016 without age restriction. Study quality was assessed using the "Integrated quality Criteria for the Review of Multiple Study designs" (ICROMS) tool. The selected studies were grouped into the topics haemodialysis, endoscopy, oncology, and dental care.
**Results:** Of a total of 7830 identified titles and abstracts, 52 reports fulfilled the inclusion criteria. A total of 35 intervention studies were quality-assessed, the other 17 were outbreak reports or laboratory studies. Only two studies fulfilled the minimal ICROMS quality criteria. Ten studies were randomized controlled trials, all testing medical products, mostly in the prevention of BSI in haemodialysis and home parenteral nutrition. The majority were small trials with poor reporting on sequence generation and allocation to the study groups. Seven studies applied a multimodal quality improvement strategy, five measuring vascular access-related BSI or catheter-related BSI in haemodialysis, one measuring hand hygiene, and one measuring HCV transmission in haemodialysis. All other intervention studies looked mainly at technology.
**Conclusion:** The identified studies were heterogeneous and generally of low quality. Multimodal strategies aiming at behaviour change work similarly in outpatient care as well as in inpatient care. The conclusions of studies testing the effectiveness of technology are uncertain. HAI surveillance and prevention should be made available also for outpatient care both in clinics and homecare.

**Disclosure of Interest**

None Declared

### P293

#### Use of primary health centres as Directly Observed Treatment (DOTS) centres; a contributing factor to development of multidrug resistant tuberculosis in Nasarawa State, Nigeria

Chinonyelum O. Okolo[1], Shafa U. Mohammed[1], Momoh O. DANJILI[2]
[1]Coordination and Strategy, Nigeria Supply Chain Integration Project, LAFIA; [2]Pharmacy, GENERAL HOSPITAL TOTO, Toto, Nasarawa State, Nigeria
**Correspondence:** Chinonyelum O. Okolo
*Antimicrobial Resistance and Infection Control* 2017, **6(Suppl 3):**P293

**Introduction:** Primary Health Centres (PHC) found at various Local Government Areas (LGAs) cannot really be called care centres [1]. Multidrug Resistant Tuberculosis (MDR-TB) has been established to develop in cases where patients do not adhere due to lack of transport fare, fear of stigmatization and long duration of therapy.

**Objectives:** This study seeks to determine another salient contributor to development of MDR-TB in patients; use of PHCs run by non-Pharmacists as DOTS centres.

**Methods: Twelve (12) Pharmacists and one lab scientist comprising of three females and ten males from the thirteen (13) LGAs in the State were randomly selected. These participants were questioned during a focus group discussion.**

**Results:** The focus group discussion arrived at the following: Primary health centres had no Pharmacist at their DOTS centres which results at TB drugs being managed by non-Pharmacists. During stock-out, TB drugs are rationed by reducing the required dose given to patients.

**Conclusion:** Primary Health Care Centres without Pharmacists contribute to development of multidrug resistant tuberculosis in Nasarawa State because the healthcare workers currently handling TB therapy at such PHCs do not understand the effects of under-dosing, non-adherence, improper drug management and storage on development of MDR-TB

**Disclosure of Interest**

None Declared

### P294

#### Application of good hygiene practices and prevention of healthcare associated infections outside health establishments

Mohamed Mahjoub[1], Nabiha Bouafia[1], Rym Bouriga[2], Hela Ghali[1], Asma Ammar[1], Asma Ben Cheikh[1], Olfa Ezzi[1], Hassene-Safouane Achach[1], Waadia Bannour[1], Radhia Hellali[1], Mansour Njah[1]
[1]Hospital Hygiene; [2]medical oncology, university hospital Farhat Hached Sousse Tunisia, Jawhara, Tunisia
**Correspondence:** Mohamed Mahjoub
*Antimicrobial Resistance and Infection Control* 2017, **6(Suppl 3):**P294

**Introduction:** Healthcare associated infections (HAI) occurring outside of health establishments are characterized by absence of controlled and organized prevention strategy with lack of structured epidemiological surveillance system which contributed to their underestimate.

**Objectives:** The objective was to specify degree of good hygiene practices (GHP)'s application among practitioners at their medical offices in a coastal town of Tunisia (second large Tunisian town after the capital Tunis).

**Methods:** A descriptive cross-sectional study was conducted in 2016 using a self-administered and pre-tested questionnaire to all physicians in private practice of a coastal town in the Tunisian center.

**Results:** Response rate was 93.1%. Majority of respondents (75.92%) have an updated vaccination status. Hydro-alcoolique solution is adopted by 53.7% of respondents for hands hygiene. "Autoclaving" for equipment sterilization is reported in 12.12% of cases. 98,14% wear gloves while acts of invasive care. The waste arising from involving infectious risks' care activities are classified with the regular waste in 87,96% of cases.

Doctors of less than 50 years are significantly more sorter of waste arising from their care activities, moreover, they have also a more updated vaccination status than those older than 50 years (respectively 36.61% versus 16.21%; p = 0.027 and 83,09% versus 62.16%; p = 0.016).

**Conclusion:** HAI are likely to rise with the healthcare increasing in liberal sector and specificities of the medicine in the current city. Knowledge of the professional practices specificities and care organization in medical office with identification of obstacles to achieve better observance of GHP application allows choice's adaptation of the precautions to be taken in this sector. To be able to fill in the gaps of training, information and awareness, and to organize the fight and the prevention of structured HAI in liberal sector, willingness to improvement of care's quality and safety in this sector is required with concrete involvement of several stakeholders.

**Disclosure of Interest**

None Declared

## Inpatient antibiotic use and antibiotic stewardship

### P296

#### Antibioprophylaxis in cesarean section: what are the current practices in four hospitals in Benin?

Angèle M. DOHOU[1], Valentina BUDA[2], Thierry VAN HEES[3], Achille Loconon YEMOA[4], Sévérin ANAGONOU[5], Françoise VAN BAMBEKE[1], Francis Moïse DOSSOU[6], Olivia DALLEUR[1]
[1]Louvain Drug Research Institute, Université catholique de Louvain, Brussels, Belgium; [2]Pharmacology and Clinical pharmacy, "Victor Babes" University of Medicine and Pharmacy, Timisoara, Romania; [3]Center for Interdisciplinary Research on Medicines, Université de Liège, Liège, Belgium; [4]Unité de formation et de recherche en Pharmacie; [5]Bacterioly and virology; [6]Surgical pathology, Université d'Abomey Calavi, Cotonou, Benin
**Correspondence:** Angèle M. DOHOU
*Antimicrobial Resistance and Infection Control* 2017, **6(Suppl 3):**P296

**Introduction:** Cesarean delivery is free for the patients in Benin since 2009 and therefore, this practice is increasing. Good practice of antibioprophylaxis for cesarean delivery has been shown to reduce the incidence of postoperative infectious morbidity.

**Objectives:** To assess the quality of antibioprophylaxis in cesarean section according to the "five conventional criteria of antibioprophylaxis" (i.e.: right indication, molecule, dose, timing, duration).

**Methods:** We conducted a prospective observational study in four hospitals in the south of Benin over a period of 1 month. Data was collected by trained survey teams and was analyzed using SPSS v. 24 software.

**Results:** 141 cesareans (81.56% emergency cases, n = 115) were observed. 99.29% (n = 140) of women undergoing cesarean intervention received an antibiotic in the hour before the incision or after the cord clamping. The antibiotics used in the hospitals (metronidazole, ampicillin, gentamicin, ciprofloxacin, ceftriaxone, amoxicillin + clavulanic acid, ceftriaxone + sulbactam) were not those recommended by the guidelines (cefazolin). In the same hospital, three to sixteen different regimen were prescribed. Patients received up to five antibiotics. Only 50.71% (n = 71) of them received the recommended dose of antibiotic for the first administration. Moreover, the antibioprophylaxis period was continued for more than the recommended 48 hours in 31.42% patients.

**Conclusion:** In the four hospitals, the practices of antibioprophylaxis in cesarean intervention were not uniform and the use of the antibiotics was not as recommended. Broad-spectrum antibiotics were used in prophylaxis, increasing the risk of selection antibiotic resistance, which is an important threat to public health. Further investigations are needed to understand the reasons of this practice and to develop solutions to improve antibioprophylaxis in cesarean section and other interventions in Benin.

**Disclosure of Interest**

None Declared

### P297

#### Are antibiotic resistant pathogens more common in subsequent episodes of diabetic foot infection?

Ilker Uckay[1], Elodie von Dach[1], Benjamin Kressmann[2], Dan Lebowitz[2], Funda Timurkaynak[1], Benjamin A. Lipsky[2], Didier Pittet[1]
[1]Infection Control Program; [2]Infectious Diseases, Geneva University Hospitals, Geneva, Switzerland
**Correspondence:** Ilker Uckay
*Antimicrobial Resistance and Infection Control* 2017, **6(Suppl 3):**P297

**Introduction:** After antibiotic therapy of an initial diabetic foot infection (DFI), pathogens isolated from subsequent episodes might become more resistant to commonly prescribed antibiotics. If so, this might require modifying current recommendations for the selection of empiric antibiotic therapy.
**Objectives:** We investigated whether the Infectious Diseases Society of America (IDSA) DFI guideline recommendations should be modified based on the number of past DFI episodes.
**Methods:** Single-center retrospective cohort survey of patients hospitalized for DFI.
**Results:** We found 1018 DFI episodes in 482 adult patients, who were followed-up for a median of 3.3 years after the first DFI episode. The total number of episodes was 2257; the median interval between recurrent episodes was 7.6 months. Among the recurrent DFIs, the causative pathogens were the same as in the previous episode in only 43% of cases (158/365). *Staphylococcus aureus* was the predominant pathogen in all episodes (range, 1 to 13 episodes) and was not more prevalent with the increasing number of episodes.
DFI were treated with systemic antibiotics for a median duration of 20 days (interquartile range, 11-35 days). Overall, the pathogens of recurrent episodes were not more resistant compared to the prior ones. The resistance pattern to key antibiotics such as methicillin, rifampicin, clindamycin or ciprofloxacin lacked significant increase over the episodes (Pearson-$\chi^2$-tests 0.76, 1.00, 0.06 and 0.46; corresponding p-for-trend values were 0.21, 0.27, 0.38, and 0.08, respectively)
**Conclusion:** Recurrent episodes of DFI are frequent following the successful treatment of a first episode. A history of prior DFI episode did not predict for the greater likelihood of an antibiotic resistant isolate in subsequent episodes. Thus, broadening of the spectrum of empiric antibiotic therapy for recurrent episodes of DFI does not appear necessary.
**Disclosure of Interest**
None Declared

### P298

#### Less antibiotic use and remission in diabetic foot infections

Ilker Uckay[1], Elodie von Dach[1], Benjamin Kressmann[2], Funda Timurkaynak[1], Didier Pittet[1]
[1]Infection Control Program; [2]Geneva University Hospitals, Geneva, Switzerland
**Correspondence:** Ilker Uckay
*Antimicrobial Resistance and Infection Control* 2017, **6(Suppl 3):**P298

**Introduction:** Diabetic foot infections (DFIs) are a source of systemic and local antibiotic misuse worldwide. DFI recur frequently.
**Objectives:** Optimizing the duration of systemic antibiotic prescription for community-acquired and healthcare-associated DFI.
**Methods:** In the frame of a clinical pathway for DFI, we conducted a multivariate, cluster-controlled (at patient's level) Cox regression model among adult patients hospitalized for DFI; defining treatment failure as a clinical recurrence ≥14 days after treatment ended
**Results:** Among 1018 DFI episodes in 482 patients, 392 were osteomyelitis cases, 626 soft tissue infections, 246 large abscesses, 322 cellulitis, 335 necrosis, and 313 cases with revascularization. We performed surgical debridement in 824 episodes (81%), of which 596 episodes (59%) involved partial amputation. The median total duration of antibiotic treatment was 20 days, 5 days of which was intravenous. After a median follow-up of three years, 251 episodes (251/1018; 25%) witnessed a second DFI episode or more at the same anatomical localization. In multivariate analysis, remission was inversely associated to diabetes mellitus type I (hazard ratio (HR) 0.3, 95%CI 0.2-0.6) and the number of surgical interventions (HR 0.8, 0.6-0.9). The total duration of antibiotic prescription or of its parenteral use had no influence (both HRs 1.0, 95%CI 0.99-1.01). More than 3 weeks of antibiotics had the same outcome than less than 3 weeks. More than 1 week of intravenous therapy had the same effect as less. Plotting of antibiotic durations against recurrence failed to identify any thresholds.
**Conclusion:** We could not define the optimal duration of antibiotic therapy for DFI. In view of the known hazards of unnecessarily prolonged antibiotic therapy, these limited data might support shorter treatment duration; for which we started a prospective randomized trial.
**Disclosure of Interest**
None Declared

### P299

#### Effect of an intervention targeting inappropriate continued empirical vancomycin use: a quasi-experimental study in a region of high mrsa prevalence

Pyoeng Gyun Choe[1,2], Hei Lim Koo[2], Eunyoung Lee[1], Kyoung-Ho Song[1], Wan Beom Park[1], Ji Hwan Bang[1], Eu Suk Kim[1], Hong Bin Kim[1], Sang Won Park[1], Myoung-don Oh[1], Nam Joong Kim[1]
[1]Department of Internal medicine, Seoul National University College of Medicine; [2]Infection Control Office, Seoul National University Hospital, Seoul, Korea, Republic Of
**Correspondence:** Pyoeng Gyun Choe
*Antimicrobial Resistance and Infection Control* 2017, **6(Suppl 3):**P299

**Introduction:** In our institution, over the last 12 years, about 60% of *S. aureus* infections were methicillin-resistant (MRSA), and this proportion has not changed significantly, whereas vancomycin consumption has increased more than twofold, from 18 defined daily doses (DDDs) per 1,000 patient-days to 40.0 DDDs per 1,000 patient-days. Our previous analysis showed that a quarter of total vancomycin use represented inappropriately continued empirical use.
**Objectives:** To evaluated the effect of an antimicrobial stewardship intervention targeting for inappropriate continued empirical vancomycin use.
**Methods:** This was a quasi-experimental study comparing vancomycin use in a 6-month pre-intervention and 6-month intervention period in a single tertiary hospital. If empirical vancomycin was continued for more than 96 hours without documentation of beta-lactam-resistant gram-positive microorganisms it was considered inappropriate continued empirical vancomycin use. The intervention consisted of the monitoring of appropriateness by a pharmacist and direct discussion with the prescribing physicians by infectious diseases specialists when empirical vancomycin was continued inappropriately. An interrupted time series analysis was used to compare vancomycin use before and during the intervention.
**Results: :** During the intervention period, we identified 272 cases of inappropriate continued empirical vancomycin use. The infectious diseases specialist intervened in 223 (82.0%) of these cases, and 148 (66.4% of these 223 cases) were discontinued within 24 hours of the intervention.
Following implementation of the intervention, overall vancomycin consumption decreased by 14.6%, from 37.6 DDDs/1,000 patient-days in the pre-intervention period to 32.1 DDDs/1,000 patient-days in the intervention period ($P < 0.001$). The inappropriate consumption of vancomycin also declined from 8.0 DDDs/1,000 patient-days to 5.8 DDDs/1,000 patient-days ($P = 0.009$).
**Conclusion:** Intervention involving direct discussion between infectious diseases specialists with prescribing physicians significantly reduces the amount of inappropriate continued empirical vancomycin use.
**Disclosure of Interest**
None Declared

### P300

#### Implementing antimicrobial stewardship program in a cancer hospital in Pakistan

Aun Raza, Faisal Sultan
Internal medicine, Shaukat Khanum Memorial Cancer Hospital and research Centre, Lahore, Pakistan
**Correspondence:** Aun Raza
*Antimicrobial Resistance and Infection Control* 2017, **6(Suppl 3):**P300

**Introduction:** Antimicrobial stewardship programs in hospitals intend to rationalize the use of antimicrobials to improve patient care, reduce drug resistance and to curtail the hospital costs.
**Objectives:** To implement an electronic based antimicrobial stewardship program in a cancer hospital.
**Methods:** Shaukat Khanum Memorial Cancer Hospital and Research Centre is a 187-bed cancer hospital in Lahore, Pakistan. An antimicrobial stewardship program was in place from 1995 to 2010 using a paper approval system which became increasingly difficult to implement due to increasing work volume and increasing resistance of micro-organisms and limited ability to restrict. The in house development of a hospital information system provided the opportunity to create an electronically driven antibiotic stewardship program. This was implemented in 2011/12 and orders for a list of restricted antibiotics generate an approvals cascade. The system allows for automated approval for specific indications as well as initiating an approval queue for unlisted indications. The infectious diseases service receives the electronic approval request, along with indications which are able to review from complete patient data, directly from the approval screens. They have the option accept, reject or modify the request, which then allows the pharmacy to dispense accordingly. Restricted antimicrobials include Carbapenems, Glycopeptides, antifungals, Linezoild, Colistin etc. Some agents, especially those used in sepsis are dispensed in limited number of doses to allow timely administration while the approvals are processed. This is the first such system that we are aware of in the developing world. We reviewed the defined daily doses (DDD) of four antibiotics (Meropenem, Imipenem, Vancomycin and Teicoplanin) used in adults, 6 months before and after implementing electronically driven antimicrobial stewardship program.
**Results:** Implementing electronic based antimicrobial stewardship program in our hospital fulfills the requirements of an effective antimicrobial stewardship program. There was 291% reduction in DDD of Meropenem, followed by 91% reduction in Vancomycin, 37% and 22% reduction in Teicoplanin and Imipenem, respectively.
**Conclusion:** In specialized hospitals with limited numbers of ID trained people a strong electronic based ASP is a viable option for managing maximum numbers of antibiotic prescriptions.
**Disclosure of Interest**
None Declared

### P301

#### Drug utilization review; a predictor of an effective antibiotic stewardship program if tagged with standard guidelines

Amjad mahboob[1], Umar zia[2], Sidrah andleeb[2]
[1]Internal Medicine, Gajju Khan Medical College, Swabi; [2]Pharmacy, Shaukat Khanum Memorial Cancer Hospital and Research Centre, Lahore, Pakistan
**Correspondence:** amjad mahboob
*Antimicrobial Resistance and Infection Control* 2017, **6(Suppl 3):**P301

**Introduction:** Emergence of resistant-bacterial strains is a serious challenge in treatment of infections these days. One of the major causes is inappropriate use of antibiotics. Prompt and coordinated actions like antibiotic stewardship program (ASP) and promotion of standard guidelines are required to prevent our journey to pre-antibiotic era.
**Objectives:** The objective was to measure the effectiveness of ASP at SKMCH & RC.
**Methods:** Retrospective cross-sectional DUR for antibiotics were conducted before and after the implementation of ASP. Comparison of the two DURs was done in a retrospective cohort fashion. Adult and pediatric patients who completed their antibiotic course in chemo-bay of SKMCH & RC were selected. Laboratory results regarding fever, absolute neutrophil count (ANC) and culture susceptibility were reviewed for proper identification of infection. Culture results, ANC and temperature were evaluated throughout antibiotic use. Dosage, route and frequency were checked for appropriateness. Rationality of antibiotic use was decided using antimicrobial guidelines of IDSA. Hospital information system (HIS) was used to retrieve the data.
**Results:** Drug utilization review (DUR) for chemo-bay antibiotics was carried out before and after implementation of ASP. The first DUR was carried out in the mid of 2011 for two months. A second DUR, after ASP implementation was carried out in the mid of 2013. Results of pre and post ASP DUR were compared using antimicrobial guidelines of Infectious disease society of America (IDSA). It was observed that 32.3% of the cases received antimicrobials according to the guidelines at chemo-bay in 2011, whereas in 2013 it was 72.4%.
**Conclusion:** These results demonstrated that DUR if tagged with standard guidelines is an effective tool to measure the implementation of ASP and will encourage judicious use of antibiotics in the Hospital.
**Disclosure of Interest**
None Declared

### P302

#### A district maternity hospital antimicrobial consumption in the setting of a national antimicrobial appropriate use intervention, China

Ying FANG[1], Wen-juan ZHANG[2], Wen-hong ZHOU[3], Wei GUO[3], Yi-hua HUANG[1], Yan DOU[1], Yan HUANG[4], Bin GAO[5]
[1]Pharmacy; [2]Nosocomial Infection Control Unit; [3]Obstetrics unit; [4]Anesthesiology unit, Tianjin 2nd Hospital; [5]Infectious disease, Tianjin 4th centre hospital, Tianjin, China
**Correspondence:** Bin GAO
*Antimicrobial Resistance and Infection Control* 2017, **6(Suppl 3):**P302

**Introduction:** Clinical use of antimicrobials in maternity hospitals, serving major women healthcare in China, was conducted by the Chinese special clinical antimicrobial appropriate use (CAAU) program (2011 ~ 2013), without subsequent evaluation.
**Objectives:** To investigate the effectiveness of the CAAU program in a maternity hospital.
**Methods:** Multi-modal strategy following local and national guidelines for the appropriate use of antimicrobials, including antimicrobial prescribing target settings, surveillance of prophylactic use of antimicrobials for C-section surgery and prevention of surgical site infections, as well as performance feedback, were introduced in a district maternity hospital in 2010. WHO Anatomical Therapeutic Chemical classifications of antimicrobials was used to calculate defined daily doses (DDDs). Annual antimicrobial consumption data from Jan 2009 to Dec 2015 were obtained and converted to DDDs per 100 patient-days.
**Results:** The annual antimicrobial consumption by DDDs per 100 patient-days decreased nearly by a half among inpatients ($93.79 \pm 18.23$ in 2009, $44.78 \pm 13.43$ in 2010 ~ 2012); it remained however stable in the hospital ($147.49 \pm 48.71$ in 2009, $154.56 \pm 32.78$ in 2010 ~ 2012). Following a targeted workshop intervention (2013-2014), we observed a significant decrease in annual antimicrobial consumption among inpatients ($22.20 \pm 4.80$ in 2013, $16.61 \pm 7.55$ in 2014), and more than 30% reduction in the hospital ($101.32 \pm 9.54$ in 2013, $90.94 \pm 18.02$ in 2014). After the targeted workshop intervention (2013-2014), annual antimicrobial consumption in inpatients in 2015 was quite similar ($23.33 \pm 5.60$ in 2015) as in 2013; in the hospital it was similar ($180.16 \pm 62.68$ in 2015) as that observed before the intervention.
**Conclusion:** Systematic and comprehensive interventions are necessary to keep sustained the reasonable use of antimicrobials.
**Disclosure of Interest**
None Declared

### P303

#### Carbapenem usage in medical wards: an antibiotic stewardship feedback project

Choon Seong Ng[1], Petrick Periyasamy[2], Chee Lan Lau[3], Dato' Faridah Md Yusof[3], Datin Dr Norlinah Mohd Ibrahim[1], Roslina Abd Manap[4]
[1]Internal Medicine; [2]Infectious Disease; [3]Pharmacy; [4]Office of Deputy Dean (Undergraduate & Alumni), HOSPITAL CANSELOR TUANKU MUHRIZ, NATIONAL UNIVERSITY OF MALAYSIA MEDICAL CENTRE (PPUKM), Kuala Lumpur, Malaysia
**Correspondence:** Choon Seong Ng
*Antimicrobial Resistance and Infection Control* 2017, **6(Suppl 3):**P303

**Abstract video clip:** Carbapenem stewardship programmes (CSP) can reduce antibiotic use but clinical outcome of such measures needs further evaluation. We examined this in a prospective manner using feedback mechanism.

**Methods:** Our single-center prospective cohort study involved all carbapenem prescriptions across the medical wards in a tertiary university hospital setting. The impact of such stewardship was analysed according to the accepted and the rejected groups. The primary endpoint was safety. Safety measure applied in this study was the death at 1 month. Secondary endpoints included length of hospitalisation and readmission.

**Results:** Over the 19 months' period, input from 144 carbapenem prescriptions was analysed on the basis of acceptance of our CSP recommendations on the use of carbapenems. Recommendations made were as follows : de-escalation of carbapenem; stopping the carbapenem; use for a short duration of 5-7 days; required prolonged duration in the case of carbapenem-sensitive Extended Spectrum Beta-Lactamases bacteremia; dose adjustment; and surgical intervention for removal of septic foci. De-escalation, shorten duration of carbapenem and carbapenem cessation comprised 79% of the recommendations. Acceptance rate was 57%. Those who accepted CSP recommendations had no increase in mortality ($p = 0.92$), had a shorter length of hospital stay (LOS) and had cost-saving. Infection-related deaths were found to be higher among those in the rejected group. Lastly, Pitt's bacteremia score appeared to be a key element affecting the carbapenem prescription's behaviour in this trial.

**Conclusions:** Carbapenem stewardship program in the medical wards not only saves money, but most importantly it is safe and does not harm the patients with added benefits of reducing the length of hospital stay. However, more time is needed to engage the primary clinical teams by formal clinical presentation and immediate personal feedback by senior Infectious Disease (ID) personnel to increase its acceptance.

**Disclosure of Interest**
None Declared

### P304

#### Effects of the antimicrobial stewardship programs on the antimicrobial resistance and clinical outcomes in a tertiary university hospital

Jingwen Li[1], Leilei Li[1], Yi Xie[2], Weijia Yin[1], Fu Qiao[1], Shichao Zhu[1], Zhiyong Zong[1]
[1]Department of Infection Control; [2]Department of Laboratory Medicine, West China Hospital of Sichuan University, Chengdu, China
**Correspondence:** Jingwen Li
*Antimicrobial Resistance and Infection Control* 2017, **6(Suppl 3):**P304

**Introduction:** Antimicrobial resistance continues to increase and has become a serious public health issue in China, which has initiated a nationwide antimicrobial management program since June 2011.

**Objectives:** This study aimed to investigate the effectiveness of the antimicrobial management program on antimicrobial resistance and inpatient outcomes in West China Hospital, a tertiary hospital with over 5,000beds.

**Methods:** Numbers of hospitalized patients, their length of hospital stay (LOS), all-cause mortality rates, the antibiotics use density were counted and the results of antimicrobial resistance in bacteria were compared before and after nationwide antimicrobial management program in 2011 (i.e., 2009 to 2010 vs. 2012 to 2015). Poisson distribution,Pearson correlation analysis and Chi-Square test were used in data analysis.

**Results:** The number of discharged patients was 155,609 and 182,116 for year 2009 and 2015, respectively, representing a 17% increase. The antibiotics use density (AUD) decreased from 60.96 (2009-2010) to 41.19(2012-2015, $P = 0.0002$). Noticeably, AUD sharply dropped to 36.49 in 2015, evidencing the increasingly prudent prescription of antibiotics. At the same time, the all-cause in-hospital mortality rate decreased significantly ($P = 0.02$) with little change in the average LOS. In addition, unrestricted use, restrictions on the use and special use of antimicrobial drugs have declined. However, it is necessary to point out that the change of the bacteria resistance rate was heterogeneous. For Gram-positive bacteria, MRSA decreased from 37.1% to 29.1% ($P = 0.00$) while E. faecium increased from 4.9% to 10.9% ($P < 0.05$). For Gram-negative bacteria, the non-susceptible rates to carbapenems in A. baumannii, P. aureginosa and K. pneumoniae increased after the antimicrobial stewardship (51.5% vs. 58.5%, 23.9% vs. 26.6%, and 2.1% vs. 3.2%, $P < 0.05$).

**Conclusion:** The antimicrobial stewardship has been effective to promote proper use of antimicrobial drugs, resulting in significant decrease of antimicrobial drug overuse. Although AUD decreased, the finding of significant increase in multiple drug-resistant bacteria, particularly carbapenem-non-susceptible Gram-negative bacilli, needs to be closely examined.

**Disclosure of Interest**
None Declared

### P305

#### High use of third generation cephalosporins in a university hospital in Tirana, Albania: results of a point prevalence survey

Silva Tafaj[1], Donika Mema[2], Borana Galanxhi[2], Sofiela Telo[2], Ann Versporten[3], Herman Goossens[3], Perlat Kapisyzi[2]
[1]Microbiology Laboratory; [2]UNIVERSITY HOSPITAL "SHEFQET NDROQI", Tirana, Albania; [3]Laboratory of Medical Microbiology, Vaccine and Infectious Diseases Institute, University of Antwerp, Antwerp, Belgium
**Correspondence:** Silva Tafaj
*Antimicrobial Resistance and Infection Control* 2017, **6(Suppl 3):**P305

**Introduction:** A uniform and standardized method for surveillance of antimicrobial use in hospitals was used to assess the variation in antimicrobial prescribing in University Hospital Shefqet Ndroqi, Tirana, Albania. bioMérieux provided unrestricted funding support for the survey.

**Objectives:** To gain a first insight into the patterns of antibiotic consumption and detect targets for antibiotic prescribing improvement in our hospital.

**Methods:** PPS was conducted in four separate days over a one month period between March 17 and April 16, 2015. The survey included all inpatients receiving an antimicrobial on the day of PPS. Data collected included age, gender, weight, antimicrobial agents, doses, reasons and indications for treatment, microbiological data, compliance to guidelines, documentation of reasons and stop/review date of prescription. Denominators included the total number of inpatients. A web-based application is used for data-entry, validation and reporting as designed by the University of Antwerp (http://www.global-pps.com).

**Results:** Of 127 patients admitted in a total of five wards surveyed, 117 patients (92.9%) received one or more antimicrobials. AM prevalence ranged from 93.3% to 97.6% in Pneumology Adult Medical Wards, 76.2% in Surgery Ward and 100% in ICU. The indications were therapeutic in 74.4% of antimicrobials prescribed and prophylactic in 24.6%.

Out of 191 antimicrobial treatments, 110 (57.6%) were administered parenterally and 81(42.4%) orally.

Top 3 indications for AM use were pneumonia (47%), bronchitis (36.1%) and tuberculosis (13.3%).

Top 3 antibiotics used were ceftriaxone 64 treatments (33.5%), ciprofloxacin 25 treatments (13.1%) and azithromycin 20 treatments (10.5%).

Most therapeutic antimicrobials were for a community acquired infection (99.3%).Duration of surgical prophylaxis was > than 1 day for all cases.
**Conclusion:** There exists an urgent need for quality improvement of antimicrobials use in our hospital. This PPS evidenced a clearly excessive use of third generation cephalosporins for medical and prophylactic indication. It also evidenced higher multiple ATB diagnosis than other countries in Europe. The carbapenems use is still low.
**Disclosure of Interest**
None Declared

### P306
### Organisational governance for antimicrobial stewardship: a Thai case study
Nantanit Sutthiruk[1], Mari Botti[1], Julie Considine[1], Andrea Driscoll[1], Ana Hutchinson[1], Kumthorn Malathum[2]
[1]School of Nursing and Midwifery, Deakin University, Melbourne, Australia; [2]Faculty of Medicine, Ramathibodi Hospital, Mahidol University, Bangkok, Thailand
**Correspondence:** Nantanit Sutthiruk
*Antimicrobial Resistance and Infection Control* 2017, **6(Suppl 3):**P306

**Introduction:** Antimicrobial stewardship (AMS) is recognised as a program to optimize antimicrobial prescribing, improve patient outcomes and minimise adverse events including antimicrobial resistance (AMR). Effective clinical governance is fundamental to the implementation and sustainability of AMS programs.
**Objectives:** The aim of this study is to describe key stakeholders' perspectives of the appropriateness of AMS governance in a university hospital in Thailand. Recognised gaps and recommendations for more effective governance were identified.
**Methods:** A case study approach was used. Semi-structured interviews were conducted with 15 key multidisciplinary clinicians, heads of department and healthcare administrators involved in AMS programs in a 1,000-bed university hospital in Bangkok. The exploration was informed and framed by the core elements of hospital AMS programs, Center for Disease Control and Prevention (CDC) recommendations. Key themes were identified through qualitative descriptive analyses.
**Results:** The presence of an influential, clinical leader to champion AMS and the introduction of a number of quality projects targeting antimicrobial prescribing such as the Antibiotic Smart Use campaign in outpatients department, the Carbapenem control program, infectious diseases specialist consultations and the antibiogram application for smartphones, were perceived as strengths of the AMS initiatives in the study hospital. Identified gaps were lack of a multidisciplinary team approach, insufficient staff education and training, low doctor engagement, and inadequate IT resources to support monitoring of antimicrobial use.
**Conclusion:** Although there is leadership support for organisation-wide implementation of AMS, these initiatives are hampered by sub-optimal information management, and have not reached clinicians of differing disciplines to the extent necessary for their engagement and consistent enactment of the principles of AMS.
**Disclosure of Interest**
None Declared

### P307
### Prescription practices for invasive fungal infections in a quaternary care center in India from an anti-fungal stewardship perspective
Anup R. Warrier, Narendra Patil
Aster Medcity, Kochi, India
**Correspondence:** Anup R. Warrier
*Antimicrobial Resistance and Infection Control* 2017, **6(Suppl 3):**P307

**Introduction:** In India, with its increasing diabetes population and growing transplant programs, non-albicans Candida and invasive aspergillus have gained importance. Judicious use of anti-fungals is thus essential to ensure appropriate antifungal therapy. On this background, we studied the prevailing practice of anti-fungal prescriptions for invasive fungal infections - Invasive Candidiasis (IC) and Invasive Aspergillosis (IA) in our 350-bed center.
**Objectives:** To review the prescribing practices for antifungals for invasive fungal infections with respect to the appropriateness of prescription - whether indicated as per standard criteria/scores, and the choice with dose of the antifungal is appropriate to the clinical situation.
**Methods:** For a period of 2 months (January & February 2017), all in-patients prescribed anti-fungals were reviewed prospectively for whether indicated (Candida Score for IC and EORTC criteria for IA), the appropriateness of choice and dose of antifungal for the suspected pathogen, and the final outcome in terms of mortality. The risk factors were assessed for relation with mortality using 2X 2 table and Fischer's Exact Test (2 tailed).
**Results:** There were 45 in-patients in whom antifungal drugs were prescribed for suspected IC (24 patients) or IA (21 patients). Of these, 36 patients (80%) met the criteria for empirical antifungal therapy with respects to Candida Score for more than/equal to 3 for IC and EORTC criteria for probable/possible IA. The choice of antifungal was appropriate in all the patients as per suspected syndrome, but the dose was inadequate in 6 patients (13.3%). Mortality was 42.2% (19 patients).
**Conclusion:** There is a gap in the assessment of patients for IC and IA as per the standard risk scores and criteria – 20% in our study received antifungals without Conference definitive criteria/scores. There is also a tendency to avoid dosing anti-fungals as per body weight – around 13% of our patients were under dosed. Thus there is a definite role for antifungal stewardship. Invasive fungals infections are associated with high mortality – around 42% in our study. However, the inadequate dosing did not achieve statistical significance in association with mortality ($p = 0.054$).
**Disclosure of Interest**
None Declared

### P308
### How integrated information systems can expedite microbiology laboratory work in antibiotic stewardship programs
Alexandra S. Simões[1], Isabel Couto[1], Melanie R. Maia[1], João Gregório[1], Pedro Póvoa[2,3], Miguel Viveiros[1], Luís V. Lapão[1]
[1]Global Health and Tropical Medicine, Instituto de Higiene e Medicina Tropical, Universidade Nova de Lisboa; [2]Cedoc, Nova Medical School, UNL; [3]Hospital de São Francisco Xavier, Centro Hospitalar de Lisboa Ocidental, Lisboa, Portugal
**Correspondence:** Alexandra S. Simões
*Antimicrobial Resistance and Infection Control* 2017, **6(Suppl 3):**P308

**Introduction:** Integrated Information Systems have proven to be useful in Antibiotic Stewardship Programs (ASP) since it increases availability of information for healthcare professionals and have been described as effective in reducing antimicrobial consumption without compromising healthcare quality, reducing antibiotic resistance and costs. However, an important amount of ASP information are results from Microbiology Laboratory. In fact, a successful ASP largely depends on the active involvement of the Microbiology Laboratory and on its capacity to deliver information.
**Objectives:** To describe the importance of the Microbiology Laboratory in ASP, and how integrated information systems can leverage it.
**Methods:** A review of literature was done based on the following keywords: Microbiology Laboratory, Antibiotic Stewardship Program, Microbiology, Antibiotics, and Information systems.
**Results:** The role of the Microbiology Laboratory in an ASP covers microbial isolation and identification, determination of antimicrobial susceptibility patterns, epidemiological surveillance and outbreak detection, education, and report of results. Despite being resources consuming, it can be leveraged by integrated information systems. Report of results - Making microbiology results accessible through an integrated information system is an advantage by ensuring that all results are available in an organized, easily accessible, and timely manner. Adequately integrated information systems facilitate the exchange of clinical and microbiological data between clinicians and laboratory. Epidemiological surveillance and outbreak detection - Information systems that incorporate information about the patient, disease, infectious agent and antimicrobial susceptibility are optimal to build up antimicrobial resistance surveillance networks, turning outbreaks detection easier and facilitating the retrospective report of results.
**Conclusion:** The Microbiology Laboratory can enhance its work by using integrated information systems for surveillance, report of results and timely communication.
**Disclosure of Interest**
None Declared

### P309
### Innovative e-health based antimicrobial stewardship program: the first 2 years of the road ahead
Carlos Palos, Luís França, Paulo Rodrigues
Infection Control and Antibiotics Committee, HOSPITAL BEATRIZ ÂNGELO, Loures, Portugal
**Correspondence:** Carlos Palos
*Antimicrobial Resistance and Infection Control* 2017, **6(Suppl 3):**P309

**Introduction:** Antimicrobial stewardship programs (ASP) improve quality of prescriptions (inappropriated in 30 to 50% of cases) with high impact on selection, duration of therapy, emergence of multidrug-resistant bacteria, length of stay and costs. E-health, defined as the use of information and communication technologies in health, plays a major role in ASP. Hospital Beatriz Ângelo is a 425 bed, paper-free, JCI accredited, general hospital, opened in 2012. Infection Control and Antibiotics Committee (ICAC) developed an ASP starting at e-prescription. A new prescription template was created, generating real-time e-mail alerts to ICAC and pharmacy whenever an agent is conditioned (e.g. carbapenems, quinolones, anti-MRSA agents) or is selected outside the right context. This allows real-time interventions, in a back-to-front way. Case-sensitive stop orders are also associated with prescriptions. On the 1st trimester of 2015, the ASP was fully implemented for those antimicrobials, joined by ceftriaxone on January 2016.
**Objectives:** To evaluate the impact of a 2-year full implementation of an e-health based ASP in hospital antibiotic profile and associated costs.
**Methods:** Antibiotic consumptions, as Defined Daily Doses (DDD), were compared for the 4th trimesters of 2014 (pre-intervention), 2015 and 2016. Associated costs were also compared on an annual basis.
**Results:** After 2 years of ASP, DDD variations were as follows: ertapenem -60%; ciprofloxacin -55%; ceftriaxone -54%; meropenem -50%; levofloxacin -47%; linezolide -26%; vancomycin -22%; amoxiclavulanate -19% (the most prescribed antimicrobial); colistin +173%; doxycyclin +151%; fosfomycin +99%; amikacyn +32%; piperacilin-tazobactam +2,0%. Quick changes occurred on the 1st year, remaining afterwards. Results were in line with guidelines, objectives (including returning of “old” antibiotics) and do reflect Carbapenem Resistant *Klebsiella* emergence, as well a significant MRSA reduction. Costs dropped 62.000€ in the 1st year.
**Conclusion:** This innovative e-health based ASP is a major tool in order to improve antimicrobial use, through improvements on prescription and real-time antimicrobial stewardship. Antibiotic prescription should be a team decision, requiring dedicated and differentiated human resources. e-health ASP plays a major role: the road is ahead!
**Disclosure of Interest**
None Declared

### P310
### Implementing effective antibiotic stewardship programs throughout a collaborative process between healthcare workers and researchers
Alexandra S. Simões, Melanie R. Maia, João Gregório, Luís V. Lapão
Global Health and Tropical Medicine, Instituto de Higiene e Medicina Tropical, Universidade Nova de Lisboa, Lisboa, Portugal
**Correspondence:** Alexandra S. Simões
*Antimicrobial Resistance and Infection Control* 2017, **6(Suppl 3):**P310

**Introduction:** Antibiotic Stewardship Programs (ASP) are designed to prevent and control antibiotic resistance. However, ASP implementation could be challenging, since, to be effective, it must be done in collaboration with healthcare workers to benefit from their involvement and inputs.
**Objectives:** To co-design and implement, with healthcare workers, an effective ASP.
**Methods:** The study was conducted in three Portuguese Hospitals using Design Science Research Methodology (DSRM) as a collaborative approach joining researchers from several healthcare areas such as medicine, pharmacy, microbiology, management and information systems, and healthcare workers in the same team.
**Results:** To help healthcare workers dealing with antimicrobial resistance, DSRM stages were followed: (i) problem identification – identification of antibiotic management processes already in place; (ii) solution definition – an integrated information system (HAITooL) to support ASP implementation; (iii) design - in collaboration with healthcare workers, HAITooL was designed to assist physicians and infection control teams to monitor/control antibiotic use and antibiotic resistance; (iv) HAITooL implementation; and (v) evaluation of HAITooL in ASP implementation. HAITool aggregates all patient related data (vital signs, microbiology and pharmacy data) in a single information system and enables integrated views of all information. HAITooL, has two big modules: (i) Monitoring, which enables real-time monitoring of patient’s clinical situation, antibiotic consumption, and rates of infection by antibiotic resistant bacteria; and (ii) Antibiotic Prescription Decision Support System, which informs physicians about the accuracy of antibiotic prescription, providing timely and appropriate information on antibiotic use.
**Conclusion:** The collaborative process between healthcare workers and researchers enables the co-design and implementation in line with organizational processes, of an information system which facilitates local epidemiological surveillance, allows the implementation of interventions to improve antibiotics, improves a timely and adequate antibiotic prescription, and facilitates the assessment of situation, surveillance and reporting of results, establishing itself as an essential tool for ASP implementation.
**Disclosure of Interest**
None Declared

### P311
### Antibiotic prescription: barriers and suggested antibiotic stewardship interventions in two Portuguese Hospitals
Alexandra S. Simões[1], Daniela A. Alves[2], João Gregório[1], Sónia Dias[1], Pedro Póvoa[3,4], Isabel Couto[1], Miguel Viveiros[1], Luzia Gonçalves[5,6], Luís V. Lapão[1]
[1]Global Health and Tropical Medicine, Instituto de Higiene e Medicina Tropical, Universidade Nova de Lisboa; [2]Unidade de Saúde Pública Internacional e Bioestatística, IHMT, UNL; [3]Cedoc, Nova Medical School, UNL; [4]Hospital de São Francisco Xavier, Centro Hospitalar de Lisboa Ocidental; [5]Unidade de Saúde Pública Internacional e Bioestatística, Instituto de Higiene e Medicina Tropical, Universidade Nova de Lisboa; [6]Centro de Estatística e Aplicações da Universidade de Lisboa, Lisboa, Portugal
**Correspondence:** Alexandra S. Simões
*Antimicrobial Resistance and Infection Control* 2017, **6(Suppl 3):**P311

**Introduction:** Antimicrobial resistance is one major worldwide problem frequently associated with antibiotics overuse and incorrect prescription. In Portugal, antibiotic consumption is still higher than European average, (45.6% vs 35.8% of hospitalized patients received antibiotics). Since physicians play an important role on antibiotic use, understand their antibiotic prescribing habits is fundamental.
**Objectives:** To identify barriers in antibiotic prescription process, in two Portuguese hospitals, in order to design and implement effective antibiotic stewardship interventions.
**Methods:** The study was conducted under the scope of HAITool project - A Toolkit to Prevent, Manage and Control Healthcare-Associated Infections in Portugal. A self-administered questionnaire was distributed to 30 physicians in two Portuguese hospitals in 2016.
**Results:** Participant physicians described the "lack of (or delayed) microbiology results" (30.0%), "no access to antibiotic susceptibility patterns" (16.7%), "lack of antimicrobial prescribing guidelines adapted to hospital epidemiology" (13.3%), and "quality of clinical data" (13.3%) as the major barriers on antibiotic prescription process. Moreover, when asked to suggest effective strategies to improve antibiotic prescription, "Education and training" was mentioned by 43.3% of respondents, followed by "easy access to local epidemiological data" and "development of antimicrobial prescribing guidelines adapted to hospital epidemiology" (16.7% each).
**Conclusion:** In order to reduce the barriers, felt by participant physicians on antibiotic prescription process, we suggest antibiotic stewardship interventions based on: (i) education on the antibiotic resistance problem, (ii) an easier access to microbiology results and local epidemiological data (e.g. information system) (ii) development of easy and accessible antimicrobial prescribing guidelines adapted to hospital epidemiology.
**Disclosure of Interest**
None Declared

### P312

#### Antibiotic stewardship in an acute care hospital: cost analysis after 2 years

Isabel Neves[1,2,3], Ana Durães[3,4], Sofia Jordão[1,2], Ricardo C. Abreu[1,2], Mário Guimarães[1,2], Maria J. Soares[1,5], David Peres[3], Fernanda Vieira[3], Valquiria Alves[1,3,5]
[1]Antibiotic Stewardship Group; [2]Infectious Diseases Unit; [3]Infection Control and Antimicrobial Resistance Unit; [4]Pharmacy; [5]Microbiology Laboratory, Matosinhos Local Health Unit, Matosinhos, Portugal
**Correspondence:** Isabel Neves
*Antimicrobial Resistance and Infection Control* 2017, **6(Suppl 3):**P312

**Introduction:** Antibiotic stewardship promotes adequate use antibiotics use, and can reduce the costs in these drugs by 10-30%. We present a multimodal strategy applied in an acute care hospital, with impact on reduction and cost after 2 year.
**Objectives:** To infer the cost effectiveness of an antibiotic stewardship program (ASP) by 2 years.
**Methods:** Multimodal strategy: 1. ASP implementation, strategy *back end* with prospective audit in 96 h; validation of all carbapenems and quinolones prescriptions, promoting its correct use. 2. Protocols and computer parameterization for surgical prophylaxis. 3. Antibiotic therapy computer parameterization for 8 days, if there's no clinical schedule. 4. Availability of the ASP Group for proactive consultation/case talk. Consumption data were taken from the Integrated Management System Medicinal Product (Glintt HS) and the ATC and DDD system were used for data analysis. Antibiotic adequacy and audit data are collected by local ASP data base.
**Results:** After 2 years: 93% of total quinolones and carbapenems prescriptions were audited, 60% and 70% considered adequate in 1$^{st}$ and 2$^{th}$ year of ASP, respectively. Comparing the 2 years of ASP with the previous period (2014), there was a decrease in the consumption of quinolones of 45% (2015) and 11% (2016) DDD/1000 patient days and carbapenems 17% and 3,5%, respectively. The economic impact was a reduction of 110 390€ in costs of total antimicrobials and 1182€/1000 patients day.
**Conclusion:** This strategy allowed to reduce direct costs in antibiotic consumption. Although the audit is directed only to 2 antibiotics classes, the back end model allowed educate prescribers for judicious use, with impact on the global antibiotics prescribing. Other costs, such as reduction of morbimortality, hospital stay, reduction of resistance, should be considered for an overall evaluation of the ASP cost effectiveness.
**Disclosure of Interest**
None Declared

### P313

#### What about of antibiotic stewardship in Portugal?

Isabel Neves[1,2,3], Pedro Pacheco[1], Carlos Palos[1], Ana P. Cruz[1], Margarida Valente[1], Goreti Silva[1], Paulo Fernandes[1]
[1]National Coordination of the Prevention and Infection Control and Antimicrobial Resistance Program (PPCIRA), Directorate General of Health, Lisbon; [2]Infectious Diseases Unit; [3]Infection Control and Antimicrobial Resistance Unit, Matosinhos Local Health Unit, Matosinhos, Portugal
**Correspondence:** Isabel Neves
*Antimicrobial Resistance and Infection Control* 2017, **6(Suppl 3):**P313

**Introduction:** In 2013 Ministry of Health issued a law to infection control/antimicrobial resistance (AR) coordinating teams in healthcare facilities (hospital (H), primary care (PC), long term care facilities (LTCF)), namely, antibiotic stewardship program (ASP) and quinolones and carbapenems prescription monitoring. Three years later PPCIRA audited institutions compliance.
**Objectives:** To audit ASP with data analysis of antibiotic consumption (AC) and antimicrobial resistance (AR).
**Methods:** 1. Audit ASP with institutions voluntary response. 2. Data analysis with AC and AR. Audit data were obtained by online questionnaire, collected by PPCIRA, AC through the national drug authority (INFARMED) and AR through national surveillance laboratory (INSA).
**Results:** The sample was satisfactory for the H and PC and not valuable for LCTF, with 91%, 77% and 18% of institutions respectively responding. Results: H: 66% with ASP, 66% with physician in charge, 85% with *back end* strategic, 61% use guidelines, 60% registered in clinical process; In the PC: 14.3% with ASP, 28.6% with physician in charge, 59% use national guidelines, 25.7% registered in clinical process. Overall AC in Portugal decreased. In H: AC 1.6 DHD in 2015, decreased 1% in 1$^{st}$ half 2016; carbapenems reduced 0.146 in 2013 to 0.133 DHD in 2015 (less 4.3% in 2015 and 6.3% in 1$^{st}$ half 2016); quinolones reduced 0.18 DHD in 2013 to 0.15 in 2015. PC: AC 21.3 DHD in 2015, decrease 3.1% in 1$^{s}$ half 2016; quinolones reduced 2.18 DHD in 2013 to 2.05 in 2015. AR: comparing 2013 and 2015, there was a decrease in resistance to quinolones in *E. coli*, *P. aeruginosa*, *A. baumannii* and to carbapenems in *P. aeruginosa, A. baumannii;* stability in methicillin resistance in *S. aureus*; although low, resistance of *K. pneumoniae* to carbapenems increase.
**Conclusion:** A regulatory law on ASP, has forced its implementation, which is already reflected in AC reduction. The majority of H has already established ASP, in PC this activity is still small, but quinolones decrease in community is favourable. In LCTF we must use an adapted survey to infer ASP. The reduction in AC seems contributed to decrease AR.
**Disclosure of Interest**
None Declared

## Hand hygiene: understand, teach and promote

### P314

#### Hand hygiene campaign to improve compliance in nursing homes

Laetitia Qalla-Widmer, Delphine Héquet, Monica Attinger, Christiane Petignat
Médecine, CHUV Unité HPCI vaud, Lausanne, Switzerland
**Correspondence:** Laetitia Qalla-Widmer
*Antimicrobial Resistance and Infection Control* 2017, **6(Suppl 3):**P314

**Introduction:** The Unit for Infection Control and Prevention of Canton Vaud (Switzerland) gives a high priority to preventing healthcare associated infections. Several studies have demonstrated that hand hygiene can significantly reduce the cross-transmission of pathogens.

Unfortunately, caregiver's observance to guidelines remains limited. In 2012-2014, a first successful hand hygiene campaign was conducted in nursing homes (NH) of the Canton Vaud. In order to sustain achievements, a second campaign was planned in 2016.

**Objectives:** The purpose of this campaign was to assess hand hygiene compliance amongst the NH staff in 2016 and to determine the evolution over time, comparing the findings to previous campaign.

**Methods:** First, hand hygiene and gloves workshops were organized in NH. Then, direct observations were performed according to the WHO moments for hand hygiene. NH were very disparate in size and mission. In order to make the results comparable, we determined a percentage of observations by type of care (hygiene, technical, mobilization) according to the NH size. A percentage of observations needed was allocated to each type of care considering their frequency and the ability to observe nurses and nursing assistants. We used an online data collection tool developed by Swissnoso (Cleanhands).

**Results:** In total, 115/160 (71%) NH took part in this campaign. A total of 15'226 hand hygiene opportunities was recorded over 8 months (1'425 hours of observations). Globally, hand hygiene compliance significantly improved from 75% to 80% ($p < 0.001$) compared to the 2012-2014 campaign. Compliance is lower for the indication "before contact" with the patient than for the indication "after contact", although compliance for both indications increased uniformly (+2% and + 3% respectively). Compliance was identical for the two categories of observed caregivers. Nursing assistants showed the greatest improvement (+6%).

**Conclusion:** This campaign was successful in light of high participation rates and significant increase in global compliance rate reaching 80%. This accomplishment can be explained by the multimodal methodology of the awareness campaign, the repetition of the campaign over time. However, a global lower compliance rate "before patient" gives hints for developing future strategy to optimize hand hygiene compliance.

**Disclosure of Interest**
None Declared

### P315

#### Barriers that explain why nurses in dutch long-term care facilities (LTCFS) do not perform hand hygiene in daily practice

Anja Haenen[1], Dominique Lescure[1], Andreas Voss[2], Marlies Hulscher[1], Anita Huis[1]
[1]IQ Healthcare; [2]Medical Microbiology, Radboudumc, Nijmegen, Netherlands
**Correspondence:** Anja Haenen
*Antimicrobial Resistance and Infection Control* 2017, **6(Suppl 3):**P315

**Introduction:** Hand hygiene (HH) is important to prevent healthcare associated infections. Research, however, shows that HH compliance, both in hospitals and LTCFs, is low. To improve HH, the choice of interventions should be informed by the barriers and facilitators that influence current HH behaviour.

**Objectives:** This study aims to explore the barriers and facilitators by LTCF nurses.

**Methods:** We organized focus group discussions with 30 nurses from 5 LTCFs. We started each group with explaining the WHO five moments of HH. Our topic guide was based on a compiled list of 57 potential determinants grouped into 7 domains: guideline factors; individual health professional factors; patient factors; professional interactions; incentives and resources; capacity for organisational change; and social, political, and legal factors. New themes that emerged during the focus groups were added to the interview guide and verified in the next focus group. New focus groups were planned until no new concepts or themes emerged and saturation occurred. The focus group discussions were transcribed verbatim and thematic analysis was independently conducted by 2 investigators. Each focus group lasted about 1.5 hour.

**Results:** Our focus group discussions provided 33 potential determinants from 6 domains (guideline factors; individual health professional factors; patient factors; professional interactions; incentives and resources; capacity for organisational change). Within this variety of determinants, we identified barriers that were specifically related to the context of LTCFs and their vulnerable residents.

**Conclusion:** Nurses from LTCFs experienced many barriers to hand hygiene, partly similar to those experienced by their colleagues in the hospital settings. The presence of these barriers will be confirmed by questionnaire in all nurses of 27 LTCF teams, from 14 LTCFs who participate in our study. The next step is to develop a tailored intervention to overcome these barriers. To assess whether our intervention is effective we will observe HH compliance in a stepped wedge cluster randomized design.

**Disclosure of Interest**
None Declared

### P316

#### Simulation-based learning: impact on hand hygiene compliance among healthcare workers from a neonatal intensive care unit

Mayra G. Menegueti, Ana Elisa R. Lopes, Fabiana M. Molina, Seila I. do Prado, Iara C. Pedro, Cristina C. Dalri, Claudio P. Campos Jr, Fabio Carmona, Fernando Bellissimo-Rodrigues
Ribeirão Preto Medical School, University of São Paulo, Ribeirão Preto, Brazil
**Correspondence:** Mayra G. Menegueti
*Antimicrobial Resistance and Infection Control* 2017, **6(Suppl 3):**P316

**Introduction:** Although hand hygiene is a corner stone for preventing healthcare associated infections, compliance with this simple procedure by healthcare workers (HCW) remains suboptimal in most of settings. Among the 5 components of the World Health Organization (WHO) Multimodal Strategy for Hand Hygiene Improvement, Training and Education is one of the most challenging.

**Objectives:** To evaluate the impact of using simulation-based learning on hand hygiene compliance among HCW from a neonatal intensive care unit (NICU).

**Methods:** This was a matched clinical trial, carried out from June to November 2016 on a tertiary-care hospital. The study population consisted of 20 HCW located on the NICU. Hand hygiene compliance was estimated through direct observation in the periods just before and after the training, following WHO standards. Simulation-based learning consisted of exposing HCW in a single session to a practical scenario with different opportunities for hand hygiene in which the professional should provide care to a patient manikin. We also evaluated the quality of the hand hygiene practice as a binary outcome (adequate or not), comparing observations with the WHO "How to handrub" and "How to handwash" techniques. We compared the results before and after the training by applying the Wilcoxon test for paired samples.

**Results:** In the pre-intervention period, the pooled mean hand hygiene compliance was 19.1% (67/347), and most of the actions were hand washings with soap and water (62.7%). After the training, the pooled mean compliance raised to 25.6% (91/355; $p < 0.001$), and most of the actions were handrubbing with alcohol-based hand gel (59.3%). Regarding the quality of the technique, in the pre-intervention period, 83.6% (56/67) of the hand hygiene actions were considered inadequate, while in the post-training period this fell to 19.8% (18/91, $p < 0.001$).

**Conclusion:** In the studied conditions, simulation-based learning was demonstrated useful for improving the quality of the hand hygiene action and the use of alcohol-based hand gel, but was only marginally effective for improving hand hygiene compliance among HCW.

**Disclosure of Interest**
None Declared

### P317

#### Development of an emergency department hand hygiene self-assessment framework: supporting the improvement of hand hygiene in Australia's emergency departments

Sally Havers[1], Kate Ryan[1], Rhonda L. Stuart[2], Andrew Stewardson[1,3], Lindsay Grayson[1,3]
[1]Hand Hygiene Australia; [2]2. Infection Prevention & Epidemiology, Monash Health; [3]Infectious Diseases Department, Austin Health, Melbourne, Australia
**Correspondence:** Sally Havers
*Antimicrobial Resistance and Infection Control* 2017, **6(Suppl 3):**P317

**Introduction:** Hand hygiene compliance (HHC) among healthcare workers in Emergency Departments (EDs) in Australia is substantially lower than other patient areas. EDs represent a unique setting with distinct environment, staff and patient factors compared with in-patient wards.

**Objectives:** The aim of this study was to develop a draft ED-specific hand hygiene (HH) self-assessment framework (SAF) that will support the identification and improvement of key issues for HH behaviour in the ED setting.

**Methods:** A draft ED HH SAF based on the validated WHO Hand Hygiene SAF was developed in October, 2016. In January, 2017 a working party was formed to review the tool, with representation from key clinicians, stakeholders and expert groups, including representatives from Hand Hygiene Australia, The Australian Commission on Safety and Quality in Health Care, the Australasian College of Emergency Medicine and the Australasian College of Infection Prevention and Control. Modifications to the draft tool were finalised in February for testing in Australian hospitals in May, 2017.

**Results:** The draft SAF includes 43 questions organised according to the five components of the WHO Multimodal Hand Hygiene Improvement Strategy. Modifications were made in response to feedback from Working Party to ensure acceptability in the ED setting and completion by ED clinicians. Coordinated pilot testing of the ED Self-Assessment Framework will commence in May 2017 by a sample of hospitals of different size, jurisdictions, and locations across Australia. Testing will include completion of the tool and a feedback survey regarding usability and utility of the tool. Final results and modifications will be compiled before release of the ED SAF for use in all EDs across Australia.

**Conclusion:** The ED setting presents unique and challenging factors for HH improvement programs. The development and introduction of an ED specific SAF based on the WHO SAF provides EDs with a standardised method of identifying and improving key barriers to HH behaviour within this setting.

**Disclosure of Interest**

None Declared

### P318

#### Hand hygiene knowledge and perceptions among anesthesia providers in Palestine

Ahmed S. Abutayeh[1], Ruba Sajdeya[1], Fady Zaben[2], Colin Green[3], Malik Zaben[1]
[1]Medical Education; [2]Nursing Education; [3]IMET2000-Pal, IMET2000-Pal, Ramallah, Palestinian Territory, Occupied
**Correspondence:** Ahmed S. Abutayeh
*Antimicrobial Resistance and Infection Control* 2017, **6(Suppl 3):**P318

**Introduction:** Hand hygiene is considered the single most important procedure for prevention of hospital acquired infection (HAI) in patients and health care providers. Hand hygiene compliance in anesthesia providers has been poorly studied in Palestine. In the absence of standard protocols for hand hygiene in the Palestinian health care system, compliance may be poor.

**Objectives:** To evaluate compliance of anesthesiologists with hand hygiene practice inside the operating room(OR).

**Methods:** A multi-centre, cross-sectional, descriptive study, using a self-administered questionnaire, was conducted in January-March 2015. Participants' compliance regarding IC practices and availability of training material and programs policies were examined using 48 items questionnaire. SPSS was used for data analysis.

**Results:** Fifty-seven anesthesia doctors from nine governmental and private hospitals in West Bank responded to our survey. Most participants were male (93%) of them 66.7% were residents, and 29.8% were specialists. 61.4% had a postgraduate degree (master, diploma, PhD). Only one third of the respondents begin their day at the OR with hand washing while only half of them always wash their hands between cases. 36.4% reported that they rarely wash their hands before inducing general anesthesia, one third of them specialists. 20.4% rarely wash their hands in neuroaxial blocks, 35.1% in peripheral blocks, 41.7% in venous cannulation. Surprisingly, 24% or participants reported the absence of alcohol inside the operating room.

**Conclusion:** Anesthesia providers adherence to hand hygiene practice and guidelines is extremely low. This exposes patients and healthcare providers to serious nosocomial infections. Further research is required to know why they do not comply the infection control (IC) practices. Hand hygiene improvement programs should be prioritized and addressed to help anesthesiologists to employ safe hand hygiene.

**Disclosure of Interest**

None Declared

### P319

#### Hand hygiene in finnish acute care hospitals: results of a national survey, 2014

Emmi Sarvikivi, Saija Toura, Dinah Arifulla, Jere Veltheim, Jukka Ollgren, Outi Lyytikäinen
NATIONAL INSTITUTE FOR HEALTH AND WELFARE, Helsinki, Finland
**Correspondence:** Emmi Sarvikivi
*Antimicrobial Resistance and Infection Control* 2017, **6(Suppl 3):**P319

**Introduction:** Hand hygiene (HH) is the cornerstone of infection control (IC) in hospitals. Surveys on resources targeted at IC in Finnish hospitals were previously conducted in 2000 and 2008, and repeated in 2015.

**Objectives:** In addition to assessing current IC resources, we aimed at finding appropriate IC indicators for future public reporting.

**Methods:** HH procedures were investigated as a part of a web-based survey on IC activities in Finnish acute care hospitals. A link to the questionnaire was distributed by e-mail to all acute care hospitals' IC teams in May 2015. Questions on handrub consumption, observation policies regarding HH compliance, existing guidelines and training available on HH were asked, illustrating the hospitals' situation in 2014. Means, medians and ranges were used for comparisons. No individualised results of hospitals were reported. Hospitals were asked which components of the survey would be useful to repeat annually, and which could be used for public reporting.

**Results:** Of the hospitals invited, 43 (95%) answered: 5 tertiary care, 15 secondary care, and 23 other hospitals. Intensive care was provided by 33 hospitals. The median of handrub consumption hospital-wide was 38 l/ 1000 patient-days (range, 20-200), and for intensive care units 65 l/1000 patient-days (range, 20-280). Observation of HH compliance was reported in 36 (84%) of the hospitals, and 34 (79%) of the hospitals had investigated the availability of HH opportunities. Written HH guidelines for nursing staff were available in all participating hospitals, and all of them provided training for healthcare workers including nurses, medical doctors and other staff. Written HH instructions for patients were available in 36, and for visitors in 37 hospitals. Questions on annual surveys and public reporting were answered by 26 and 27 hospitals, respectively. Vast majority (24/26) supported the idea of annual surveys on handrub consumption, and 20/27 considered public reporting of handrub consumption appropriate.

**Conclusion:** Compared to earlier surveys, handrub consumption increased both hospital-wide and in intensive care units. Annual handrub consumption on hospital level might be one of the publicly reported IC indicators in the future.

**Disclosure of Interest**

None Declared

### P320

#### Hand hygiene in Dutch general practice offices

Nataliya Hilt[1], Mariëtte Lokate[2], Alfons OldeLoohuis[3], Andreas Voss[4]
[1]Medical Microbiology; [2]Epidemiology and Medical Microbiology, University Medical Center Groningen, Groningen; [3]Primary and Community Care, Radboud University Medical Center; [4]Microbiology and Infectious Diseases, Canisius-Wilhelmina Hospital, Nijmegen, Netherlands
**Correspondence:** Nataliya Hilt
*Antimicrobial Resistance and Infection Control* 2017, **6(Suppl 3):**P320

**Introduction:** Hand hygiene (HH) is considered one of the most important measures to prevent health care-associated infections (HAI) and to limit the spread of antimicrobial-resistant pathogens. So far, most studies focused on HH compliance within the hospital setting, whereas little is known for the outpatient setting.
**Objectives:** The aim of this study was to evaluate compliance with the recommendations on HH in general practices (GP), based on the professional international and national guidelines.
**Methods:** An observational study was conducted at five Dutch GP offices in September, 2016. We measured HH compliance through direct observation using WHO's 'five moments of hand hygiene' observation tool. All observations were done by one trained professional.
**Results:** Hereby we present the preliminary data of our study. 28% of observed HCWs were men (n = 5) and 72% were women (n = 13). The age of the healthcare providers ranged from 21 to 64 years (mean 41.4 ± 13.02). The preferred method of HH was soap and water (56%) versus 44% for alcohol-based hand rub (ABHR). We monitored a total of 155 hand hygiene opportunities in 18 HCWs. The overall compliance was 34%. HH compliance per profession was 30%, 48% and 17% for the general practitioners, practice assistants, and nurses, respectively. HH compliance varied depending on which of the five moments HCWs were undertaking. Compliance was 6% before patient care; 49% after patient care; 52% after body fluid exposure; 29% prior to executing aseptic tasks; and 0% after contact with the patient environment (after home visit of a patient).
**Conclusion:** The WHO recommended switch from hand wash to ABHR was not implemented by the majority of HCWs in three observed general practice offices. HH compliance among health care providers in Dutch GP offices was found to be low, especially with regard to moment 5, after contact with the direct patient environment during home visits.
**Disclosure of Interest**
None Declared

### P321

#### The bubble of life - hand hygiene saves lives

Claire Kilpatrick[1], Julie Storr[2]
[1]S3 Global; [2]Global S3, London, United Kingdom
**Correspondence:** Claire Kilpatrick
*Antimicrobial Resistance and Infection Control* 2017, **6(Suppl 3):**P321

**Introduction:** A multimodal approach to hand hygiene improvement, grounded in application of the 'My Five Moments for Hand Hygiene' at the point of care, is recommended by the World Health Organisation (WHO). To build on current achievements and stimulate a refreshed approach in the context of real-life clinical workflow, a description of the 'bubble of life' was created to present a novel approach to engagement, enhanced compliance rates and support for behaviour change in the long term.
**Objectives:** To stimulate infection prevention leaders to think differently about hand hygiene improvement within clinical workflow, aiming to overcome known barriers and challengesTo encourage infection prevention leaders to strengthening their messages and the words used when talking about the importance of hand hygiene at the right times in practice, using the 'bubble of life' concept
**Methods:** An analysis of existing language used to describe WHO's My Five Moments for Hand Hygiene was undertaken. A novel, progressive approach to simplify the language was then used to re-describe the patient zone and health care interactions within it, informed by expert input from the field of communication. The new description has undergone rudimentary testing in two countries and was part of a larger exploratory exercise to assess the feelings evoked by words commonly used in a hand hygiene context.
**Results:** Using the 'bubble of life' to describe the patient zone has enhanced engagement with the My Five Moments for Hand Hygiene in selected countries. It has provided the opportunity to simplify complex germ transmission theories and aid visualisation of germ transfer and methods to reduce risks to patients during routine clinical work-flow interactions.
**Conclusion:** Capacitating health workers in their messaging and communications, in addition to enhancing health worker understanding has the potential to support realistic and reliable application of WHO's My 5 Five Moments for Hand Hygiene in conjunction with a multimodal approach. As hand hygiene compliance rates are still not optimal, a refreshed approach in the context of real-life clinical workflow is important for safer patient care.
**Disclosure of Interest**
None Declared

### P322

#### Strategies to improve hand hygiene practice

Sook Cheng Wong, Phei Yin Tan, on behalf of Isolation Ward B46 Nursing, KHOO TECK PUAT HOSPITAL, Singapore, Singapore
**Correspondence:** Sook Cheng Wong
*Antimicrobial Resistance and Infection Control* 2017, **6(Suppl 3):**P322

**Introduction:** Healthcare Associated Infection (HCAI) is a major patient safety issue and prevention must be a high priority for Healthcare settings. The impact of HCAI are prolonged hospital stay, disability, increased antimicrobials resistance, financial burden and deaths. When HCWs fail to perform hand hygiene between patient contact and care delivery, microbial transfer occurs. Transient microorganisms are removed by routine hand washing or handrub, which is a simple and effective preventive measure.
**Objectives:** To improve Hand Hygiene among Healthcare team. To prevent microorganism transmission and improve patient safety
**Methods:** Assign ownership and responsibility to every staff who delivers care to the patient to ensure Hand Hygiene compliance. All staff are encouraged to be Hand Hygiene Champions practicing the '5 Moments of Hand Hygiene' consistently. The CCTV in the rooms were utilized to monitor staff practices in patients' rooms. Reminders to attending staff were sent to patient's room via phone calls, call-bells and intercom when needed.
**Results:** Hand Hygiene Compliance increased from 72% to 90% and results were shown to staff monthly during Roll Calls and reminders for compliance. Staff felt motivated and were challenged to maintain compliance which Improved staff satisfaction, pride of achievement and teamwork, as staff rise to the challenge for better compliance. This improvement was sustained from April to December 2015
**Conclusion:** Assigning ownership and responsibility to every staff puts onus on the staff to monitor, advise and remind colleagues to promote Hand Hygiene compliance, Staff takes the responsibility for colleagues and patient safety seriously as it is within their control. Hand Hygiene equipment and reminders are visual and accessible within the area. To sustain Hand Hygiene compliance, all staff are empowered to remind colleagues during handover, via intercom and phone to comply with practices. Encouragement, peer pressure and challenge to be the Hand Hygiene Role Model promote compliance. Support and Role Modeling from Consultants increase junior Drs' Hand Hygiene practice. Celebrate small successes, to motivate, praise and encourage compliant behaviour as monthly results are released by Infection Control.
**Disclosure of Interest**
None Declared

### P323

#### Does wearing fake tan effect hand hygiene practice? A pilot study

Lynn Melone, Lesley Price, Valerie Ness, Liz Simpson, Jennifer MacDonald, Jacqueline Reilly
School of Health and Life Sciences, Glasgow Caledonian University, Glasgow, United Kingdom
**Correspondence:** Lynn Melone
*Antimicrobial Resistance and Infection Control* 2017, **6(Suppl 3):**P323

**Introduction:** Anecdotal evidence from the observation of student nurses in a clinical simulation environment suggested that those wearing fake tan had difficulty removing hand hygiene training lotion from their hands.
**Objectives:** A pilot study was performed to assess the uptake of fake tan in student nurses, and test the feasibility and acceptability of a study to evaluate the impact of wearing fake tan on the removal of hand hygiene training lotion during hand washing.
**Methods:** A voluntary sample of student nurses was asked to apply hand hygiene training lotion and then wash their hands. Following application of the training lotion and hand washing, the percentage of the hands covered with training lotion was measured by direct observation of the hands in an ultraviolet light box. Differences in the percentage of training lotion removed, in those wearing fake tan and those not, were explored using Fisher's exact tests.
**Results:** Of the 217 participants recruited, 46 (21%) wore fake tan. Statistical analysis revealed no significant difference between fake tan and non-fake tan wearers (Fisher's exact test 4.152, n = 217, p = 0.243). Fake tan did not impede the removal of hand hygiene training lotion from student nurses' hands during hand washing. However, the pilot study was designed to test the feasibility and acceptability of such a study and a number of lessons learnt can inform further research. There was initial concern that, given the popularity of wearing fake tan, student nurses would not participate in the study. The study showed this not to be the case as recruitment was successful. We were aware that there were a number of potential confounding variables including, type of fake tan used, length of time since the tan was applied, technique used by participants to apply it and whether moisturiser as well as fake tan had been used. Our study collected data on these variables but the number of possible variations and combinations made it impractical to take account of these in the analysis and highlighted the need to use an experimental design to control for these variables.
**Conclusion:** Whilst the merits of fake tan, in terms of the prevention of sun damage through sun protection behaviours are promoted, its role in hand hygiene is worthy of further investigation.
**Disclosure of Interest**
None Declared

### P324

#### Influence of pocket size alcohol-based hand-rub solutions in the degree of compliance of hand hygiene recommendations

Ginger G. Cabrera Tejada, Juan G. Mora Muriel, Marina D. C. Lopez-Bajoz Mesa, Ana Sofia L. Azevedo, Marina Fuster Perez, Carmen M. Benito Miralles, Concepción García González, Jose Sanchez Payá
Unidad de Epidemiología, Servicio de Medicina Preventiva, Hospital General Universitario de Alicante, Instituto de Investigación Sanitaria y Biomédica de Alicante (ISABIAL - Fundación FISABIO), Alicante, Spain
**Correspondence:** Ginger G. Cabrera Tejada
*Antimicrobial Resistance and Infection Control* 2017, **6(Suppl 3):**P324

**Introduction:** Healthcare worker compliance with hand hygiene (HH) continues being a great challenge, which may improve with the availability of pocket size alcohol-based hand rub solutions (PAS), the only format that guarantees its availability in the place and time in which it's needed.
**Objectives:** The objective of this study was to evaluate the influence of the use of PAS in the Degree of Compliance of Hand Hygiene Recommendations (DCHHR).
**Methods:** Observational cross-sectional study of a surveillance program by direct observation of the DCHHR during the daily activity of the health workers of a third level hospital during the years 2005-2016. The outcome variable was DCHHR, and as explanatory variables: use of PAS, years, age, sex, estate, know HM Triptych, attendance sessions update, and attendance area. For the association study, the Chi-square test was used, and to quantify the magnitude of the association, the Odds Ratio (OR) was calculated with its 95% confidence intervals (95% CI). The adjusted OR was calculated using a logistic regression model. The level of statistical significance was $p < 0.05$.
**Results:** A total of 47488 observations in which hand hygiene was recommended were analyzed. The DCHHR was 66.3% (9803/14778) when PAS was available, and 46.2% (15114/32710) when PAS was not available. The magnitude of the association of the different explanatory variables with the DCHHR was: AS in PF (Yes), ORa = 2.1(2.0-2.2); Years (2009-2012), ORa = 1.5(1.4-1.5); Years (2013-2016), ORa = 1.2(1.2-1.3); Age(<35 years),ORa = 0.8(0.8-0.9); Sex(Male), ORa = 0.8(0.7-0.8); Nursing, ORa = 1.2(1.1-1.3); Station(Nursing Assistant), ORa = 1.0(1.0-1.1); (Other), ORa = 0.6(0.6-0.7); Assistance Area (Medical-Surgical), ORa = 0.7(0.6-0.7).
**Conclusion:** The use of pocket sized alcohol-based hand rub solution for the accomplishment of hand hygiene is the variable that, once adjusted by the rest of variables, greater magnitude of association has with the DCHH. The recommendation of the use of PAS for the accomplishment of HH should be an absolute priority in all health centers.
**Disclosure of Interest**
None Declared

### P325

#### Understanding consumer perceptions of healthcare-associated infections and hand hygiene through a global survey

Claire Kilpatrick[1], Benedetta ALLEGRANZI[1], Maryanne McGuckin[2]
[1]World Health Organisation, Geneva; [2]McGuckin Methods International, Philadelphia, Switzerland
**Correspondence:** Claire Kilpatrick
*Antimicrobial Resistance and Infection Control* 2017, **6(Suppl 3):**P325

**Introduction:** Strategies to improve compliance with hand hygiene practices should be multimodal and multidisciplinary. Empowering patients and consumers to take an active role in encouraging health care workers to practice hand hygiene has been seen as one of the component of such strategies. A global consumer survey was commissioned by the World Health Organisation (WHO) and undertaken to determine knowledge, perceptions, and information sources for hand hygiene compliance in healthcare settings and describe considerations for connecting with consumers as positive influencers in the promotion of hand hygiene.
**Objectives:** To undertand perceptions and knowledge of consumers in relation to hand hygiene and healthcare-associated infections in a range of countries.
**Methods:** An organisation experienced in measuring consumer awareness and engagement with infection prevention programs was commissioned by WHO to survey a sample of 1,001 respondents, 18 years of age and older living in Ireland (n = 250), India (n = 251), Mexico (n = 251) and Hong Kong (n = 249) in 2014.
**Results:** The majority of respondents surveyed believe that health workers clean their hands at the right times when treating or caring for patients. Responses to questions about health workers cleaning their hands at the right time were based on respondents' own personal experience for at least seven out of ten. Significantly fewer responses were based on information from friends and relatives, and few were based on media. Those in Mexico are most likely to not go into a hospital if they thought health worker hands were not clean. Those in India are more likely to not go into a hospital than would those in Hong Kong and Ireland.
**Conclusion:** Understanding consumers' perceptions is important in knowing how to use the consumer/patient influence to further improve hand hygiene compliance in health care as part of a multimodal strategy. The results of this survey are further endorsed by similar surveys conducted in the USA.
**Disclosure of Interest**
None Declared

### P326

#### Hand hygiene adherence in relation to influenza season over six consecutive years

Rosamaria Fulchini, Philipp Kohler, Christian R. Kahlert, Werner C. Albrich, Rolf Kuhn, Matthias Schlegel
Division of Infectious Diseases and Hospital Epidemiology, Kantonsspital St. Gallen, St. Gallen, Switzerland
**Correspondence:** Rosamaria Fulchini
*Antimicrobial Resistance and Infection Control* 2017, **6(Suppl 3):**P326

**Introduction:** Health care worker (HCW) adherence with hand hygiene (HH) is important to prevent healthcare-associated infections.
**Objectives:** We aimed to assess the impact of influenza season on HH adherence.
**Methods:** We performed a retrospective analysis comparing HH adherence at a Swiss tertiary care hospital during non-influenza periods with adherence during influenza seasons defined according to surveillance reports of the Federal Office of Public Health. HH adherence was monitored by direct observation according to "my five moments" (WHO) using the mobile application *CleanHands*. The data covered the period from December 20th 2010 until November 24th 2016. The $X^2$-test was used to compare the differences in adherence between non-influenza and influenza seasons stratified for the respective years, professional groups, settings, departments and HH indications. Multivariable logistic regression was used to analyse the relationship between influenza season and HH adherence while controlling for the above mentioned covariates.
**Results:** A total of 12'740 HH opportunities were observed. Overall adherence was 77%. During influenza seasons HH adherence was higher compared to non-influenza periods with 80% vs. 76% ($p < 0.001$). The effect was most pronounced in the 2010/2011 season (87% vs. 80%, $p = 0.001$), among doctors (82% vs. 75%, $p < 0.001$) and in inpatient settings (81% vs. 78%, $p = 0.004$). In multivariable analysis influenza season was an independent predictor of increased HH adherence (OR = 1.17, 95% CI 1.05-1.30). Independent factors associated with lower HH adherence were non-nurse/non-physician compared to nurses (OR = 0.48, 95% CI 0.36-0.62), surgical theatre compared to medical wards (OR = 0.53, 95% CI 0.44-0.64), and outpatient compared to inpatient setting (OR = 0.43, 95% CI 0.35-0.53).
**Conclusion:** We identified influenza season as potential determinant that positively affects HH adherence of HCW, especially in physicians. Further systematic study is needed to verify this association and evaluate the clinical impact on transmission or nosocomial infection and implications for HCW education in a prospective setting.
**Disclosure of Interest**
None Declared

### P327

#### Web-based monitoring for 5 moments for hand hygiene: promising results for nurses but not for doctors

Helena Ojanperä, Tuula Keränen, Hannu Syrjälä
Infection control unit, OULU UNIVERSITY HOSPITAL, Oulu, Finland
**Correspondence:** Helena Ojanperä
*Antimicrobial Resistance and Infection Control* 2017, **6(Suppl 3):**P327

**Introduction:** According to WHO the golden standard for hand hygiene is direct observation.
However, usage of that method is laborious in real life in healthcare settings.
**Objectives:** To describe an online web service (SaaS) method for improving the observation and reporting of hand hygiene moments in university hospital, which allows monitoring of hand hygiene practices both in staff and administrative levels.
**Methods:** In Oulu University hospital (OUH) health care workers have been observed by linknurses since 2013. OYSTestLab, Flowmedik and OUH Infection control unit have developed online system (eRub), which included following possibilities: ward, occupation, time and type of disinfection (5 moments).These findings are coded straight by smartphone. Real-time feedback is directly available in OUH's intranet. Database obtained from 2013 to 2016 has now transferred to this eRub – system. Since February 2017 hand hygiene observations have made by this eRub.
**Results:** During 2016 handrubtime ($\geq$20 s) was reached in 31% among doctors (n = 2247) and in 68% among nurses (n = 8023). Handrub was not use in 37% by doctors and by nurses in 10%. During the whole year 2016 the median handrubtimes of 5 moments of nurses (number of observations) vs. doctors (number of observations): before touching a patient; 24 s. (n = 1991) vs. 6 s. (n = 725), after touching a patient; 25 s. (n = 2173) vs. 12 s. (n = 699), before clean/aseptic prosedure; 27 s. (n = 1371) vs. 15 s. (n = 259), after body fluid exposure risk; 26 s. (n = 1008) vs. 10 s. (n = 284), after touching patient surroundings; 22 s. (n = 1480) vs. 10 s. (n = 280).
**Conclusion:** The median handrubtime of nurses exceed 20 s. in all five moments while corresponding figures of doctors were always below 20 s. Next months will show whether hand hygiene practices of doctors will improve in this novel online eRub –system.
**Disclosure of Interest**
None Declared

### P328

#### Can we interpret hand hygiene compliance data over time?

Nicole Bartlomé-Wyss[1], Eveline Bucheli Laffer[1], Tobias Erlanger[2], Andreas Limacher[2], Anna Conen[1], Christine Mohr-Edokpolo[1], Christoph Fux[1]
[1]Clinic for Infectious Diseases and Hospital Hygiene, KANTONSSPITAL AARAU, Aarau, [2]Department of Clinical Research and Institute of Social and Preventive Medicine, Trials Unit, Bern, Switzerland
**Correspondence:** Nicole Bartlomé-Wyss
*Antimicrobial Resistance and Infection Control* 2017, **6(Suppl 3):**P328

**Introduction:** Hand hygiene is the most important intervention to reduce nosocomial infections.
**Objectives:** Its promotion by direct observation and feedback as well as educational campaigns is recommended in the literature and widely performed. However, little is known about the long-term impact of these measures on HH compliance (HHC). We analyzed the effects of hospital-wide interventions in a tertiary care hospital from 2007-2015.
**Methods:** HHC according to the WHO 5 moments was monitored yearly. We evaluated HHC dynamics by profession, ward and indication as well as the effect of specific interventions.
**Results:** A total of 12'459 HH indications were observed on 37 individual wards. Between 2007 and 2015, HHC for nurses improved from 59.0% to 78.8% (OR 1.12 per year, 95% CI 1.07-1.17), HHC for physicians from 63.1% to 68.0% (OR 1.02, 0.97-1.07). Thereby, hand disinfections without indication, measured between 2013 and 2015, significantly increased in physicians, but not in nurses. The HH indication least respected was "before invasive". Hospital-wide campaigns significantly improved HHC of nurses (OR 1.33, 1.08–1.65) and physicians (OR 1.30, 1.04–1.63). Due to substantial year-to-year variations, the HHC performance of nurses on an individual ward was not predictive for the subsequent year. Only few wards with consistently superior and inferior HHC could be identified with a trend for lower HHC of nurses in larger wards (OR per 10 beds 0.95, 0.90–1,003). Additional team instructions of wards with HHC <65% significantly improved their rates in the following year, but were not more effective than simple feedback of HHC results. For a reliable comparison of HHC between wards in a given year and between years on a given ward, a high number of observations is needed: For a presumptive HHC of 60%, 70% and 80%, respectively, 369, 323 and 246 observations per ward and year are required to achieve a CI +/-5%.
**Conclusion:** Yearly monitoring and individual feedback for every ward together with educational campaigns significantly improved HHC. The increasing rate of hand disinfections without indication in physicians underlines growing awareness, but also lacking knowledge, calling for targeted interventions. The reliable comparison of wards is strongly limited by the high number of observations needed.
**Disclosure of Interest**
None Declared

### Hand hygiene: *in-vitro* to *in-vivo*

#### P329

**Closing the gap – the three new European virucidal activity claims**

Maren Eggers, on behalf of VAH/DVV
Labor Enders, Stuttgart, Germany
**Correspondence:** Maren Eggers
*Antimicrobial Resistance and Infection Control* 2017, **6(Suppl 3):**P329

**Introduction:** An increasingly interconnected world, urbanization and population growths are factors that contribute to an increase in pathogens that cross species barriers or appear in locations not previously seen. The question is how to prevent and respond quickly to emerging viruses?
**Objectives:** When facing an emerging infectious disease outbreak, the reaction time to control the spread is crucial. Antivirals and vaccines will take time to be developed. On the other hand, appropriate hand hygiene can be adopted almost immediately to limit the spread by reducing the transmission. However, it is it is imperative to use antiseptic products and disinfectants with proven efficacy against the virus identified or suspected to cause the disease/outbreak.
**Methods:** Therefore, disinfectants have to pass a virucidal activity test performed in compliance with good laboratory practice or similar quality assurance systems and European standards. By identifying a marker viral strain which is representative of a group of viruses this would facilitate rapid testing resulting in a rapid response to recommendations and guidelines during infectious disease outbreaks.
**Results:** Recent outbreaks of emerging and endemic viruses such as norovirus have resulted in the three European virus claims for hand hygiene:
· Virucidal active (against all viruses including enterovirus 71 and enterovirus D68) covered by the marker viruses poliovirus, adenovirus and murine norvirus
· Limited spectrum of virucidal activity (against endemic viruses) covered by the marker viruses adenovirus and murine norvirus
· Activity against enveloped viruses (including new Avian Influenza Ebola, MERS and others) covered by the marker virus vacciniavirus
**Conclusion:** By testing against these marker viruses means that in case of a new pandemic a product against enveloped viruses is sufficient; in case of norovirus pandemic, products with the claim-limited spectrum of virucidal activity can be chosen. And in case of an outbreak with a very stable virus such as the enterovirus D68 epidemic in USA, France and Norway in 2014 a virucidal active product must be applied.
**Disclosure of Interest**
None Declared

#### P330

**Virucidal activity of who-hand rub formulations against enveloped viruses including Zika, Ebola and emerging coronaviruses**

Florian H. H. Brill[1], Anindya Siddharta[2], Stephanie Pfaender[3,4], Nathalie J. Vielle[3,4,5], Ronald Dijkman[3], Martina Friesland[6], Britta Becker[1], Jaewon Yang[7], Michael Engelmann[6], Daniel Todt[6], Marc P. Windisch[7], Joerg Steinmann[8], Jochen Steinmann[1], Stephan Becker[9], Marco P. Alves[3, 4], Thomas Pietschmann[6], Markus Eickmann[9], Volker Thiel[3,4], Eike Steinmann[6]
[1]Institute for Hygiene and Microbiology, Dr. Brill + Partner GmbH, Bremen; [2]Twincore, Centre for Experimental and Clinical Research, Hannover, Germany; [3]Department of Infectious Diseases and Pathobiology, University of Bern, Vetsuisse Faculty, Bern; [4]Federal Department of Home Affairs, Institute of Virology and Immunology, Bern and Mittelhäusern; [5]Graduate School for Cellular and Biomedical Sciences, University of Bern, Bern, Switzerland; [6]Institute of Experimental Virology, Twincore, Centre for Experimental and Clinical Research, Hannover, Germany; [7]Applied Molecular Virology, Institut Pasteur Korea, Seongnam, Gyeonggi-do, Korea, Republic Of; [8]Institute of Medical Microbiology, University Hospital Essen, Essen; [9]Institute for Virology, Philipps University of Marburg, Marburg, Germany
**Correspondence:** Florian H. H. Brill
*Antimicrobial Resistance and Infection Control* 2017, **6(Suppl 3):**P330

**Introduction:** To prevent viral transmission, hand hygiene is the most important measurement to reduce the spread of infections. The World Health Organization (WHO) published two alcohol-based formulations to be used in healthcare settings and outbreak situations. Inactivation efficacies of these products have not been determined against (re-) emerging viruses.
**Objectives:** In this study, we evaluated the virucidal activity of the WHO products in a comparative analysis against enveloped viruses.
**Methods:** Virucidal activity studies were performed with a quantitative suspension test with 30 seconds exposure time. One part by volume of test virus suspension and one part by volume of the organic load were mixed with eight parts by volume of one of the two WHO formulations at different concentrations.
**Results:** Zika virus (ZIKV), Ebola virus (EBOV), Severe Acute Respiratory Syndrome coronavirus (SARS-CoV), Middle East Respiratory Syndrome coronavirus (MERS-CoV), bovine coronavirus (BCoV), hepatitis C virus (HCV), influenzavirus (H1N1) and Modified Vaccinia virus Ankara (MVA) served as test viruses. All viruses tested were highly susceptible to WHO formulations. Isopropanol-based WHO formulation II demonstrated a superior virucidal effect compared to ethanol-containing WHO formulation I. Coronaviruses and ZIKV showed the highest susceptibility to both WHO formulations. Higher concentrations were required for complete inactivation of EBOV and HCV, H1N1 and MVA displayed the highest stability.
**Conclusion:** WHO alcohol-based formulations show a strong virucidal effect against emerging pathogens including ZIKV, EBOV, SARS- and MERS-CoV could be demonstrated implicating the usability of these WHO formulations in healthcare and outbreak-associated viral infections.
**Disclosure of Interest**
None Declared

#### P331

**Is chlorhexidine essential in alcohol based handrubs for surgical hand antisepsis?**

Thomas J. Hennig[1], Andreas Arndt[1], Sebastian Werner[2], Kathrin Naujox[3]
[1]Centre of Excellence Infection Control, B. Braun Medical AG, Sempach, Switzerland; [2]Managing Director; [3]Study Director / Division Manager Bacteriological Test Methods, HygCen Germany GmbH, Schwerin, Germany
**Correspondence:** Thomas J. Hennig
*Antimicrobial Resistance and Infection Control* 2017, **6(Suppl 3):**P331

**Introduction:** The use of alcohol-based hand rubs (ABHRs) for surgical hand antisepsis is recommended by the World Health Organization (WHO) and is increasing globally.
The US ASTM-E1115 standard assesses the reduction of bacteria from hands immediately after antisepsis, after wearing surgical gloves for 6 hours (persistent activity), and after multiple applications over 5 days.
In the European standard EN 12791 (2016) all products must be at least as efficacious as a reference surgical rub based on 60% n-propanol. The immediate effect after the hand hygiene procedure and the level of microbial regrowth after 3 hours under gloved hands are measured.
**Objectives:** Are ABHRs from the USA superior to European ones, as the requirement for persistent activity in US test methods is twice as long as in the European standard?
**Methods:** The method of EN 12791 (2016) was modified for direct benchmarking to assess immediate and 6-hour effects. 3 M™ Avagard™ CHG was used as reference product instead of 60% n-propanol.
The mean log reduction factors (RF) as obtained with Softa-Man®, B. Braun, (100 mL contains 45 g ethanol and 18 g n propanol) and 3 M™ Avagard™ CHG (Chlorhexidine gluconate 1% solution and ethanol 61%, w/w) were compared.
**Results:** Despite the fact that Avagard™ CHG (A) was applied 3 minutes, for twice as long as the chlorhexidine-free Softa-Man® (B), it showed lower reduction factors both immediately after application(A: RF 1.42, B: RF 1.96) and after 6 hours under surgical gloves (A: RF 1.24, B: RF 1.67, statistically significant s ($P \leq 0.01$)).

**Conclusion:** 3 M Avagard has demonstrated superior persistent activity compared to two ethanol-only products (80% and 70% w/w Ethanol) after 6 hours of glove wear but was never compared to n-propanol containing ABHRs.
Chlorhexidine has reduced efficacy against Gram-negative bacteria, is slow-acting and a known allergen.
Highly efficacious n-propanol based chlorhexidine-free ABHRs can have superior efficacy compared to chlorhexidine-containing ABHRs, even after 6 hours.
Modern ABHRs for surgical hand antisepsis do not require chlorhexidine.

Werner HP. Test report SN 17848

**Disclosure of Interest**

T. Hennig Employee of: B. Braun Medical AG, A. Arndt Employee of: B. Braun Medical AG, S. Werner: None Declared, K. Naujox: None Declared

### P332

#### Hand hygiene and the use of information and communication technologies (ICT) for education, who selfassessment framework and direct observation of compliance

Annibale Raglio[1], Franca Averara[1], Rosa Aristolao[1], Giancarla Caglioni[1], Eleonora Cacciabue[2], Simonetta Cesa[3], Fabio Pezzoli[4] on behalf of Gruppo Igiene Mani HPG23
[1]USSD Controllo Infezioni Ospedaliere; [2]Direzione Medica; [3]Direzione Professioni Sanitarie; [4]Direzione Sanitaria, ASST PAPA GIOVANNI XXIII, BERGAMO, ITALY, Bergamo, Italy
**Correspondence:** Annibale Raglio
*Antimicrobial Resistance and Infection Control* 2017, **6(Suppl 3):**P332

**Introduction:** The use of information and communication technologies (ICT) in the hand hygiene process has the threefold purpose of facilitating the spread, to stimulate the understanding / learning and facilitate the collection of data.
**Objectives:** This study presents the computerized technologies that have been developed and used at ASST Papa Giovanni XXIII (HPG23) of Bergamo and their impact on hand hygiene compliance.
**Methods:** In 2014 it was prepared an e-learning course "Hand hygiene: milestone to reduce healthcare associated infections." In the second half of 2014, the WHO Framework has been incorporated as an on-line questionnaire. All the health care workers (HCWs) were asked to discuss their possible choice during Conference in their Unit to reach one shared answer for each Unit to put in the on-line questionnaire. From 2014 to June 2015 it was developed a database Hospital Infections Informatic System (IOIS) for the collection of direct observations. The link nurses and doctors have been trained to do the observers for hand hygiene, to perform hand hygiene observations and to include the results in the database. The compliance results were compared with a direct observation performed in 2011. The alcohol hand rub solution (AHRS) consumption was calculated for 2013, 2014 and 2015.
**Results:** The e-learning course was performed by 3893 HCWs employed at HPG23. All the Units answered to the online questionnaire for the WHO Framework. Fifty-three of 75 reached the intermediate or consolidated level and 17 achieved the advanced or implanted level. The 150 link nurses and doctors have recorded on the IOIS 13847 opportunities. The average compliance was of 68%, with 71% for nurses and 66% for physicians. In 2011, the compliance was 55%. The AHRS consumption in 2013 was 13 L / 1000 patient-days, 15 L in 2014 and 19 L in 2015.
**Conclusion:** With the introduction of ICT, hand hygiene compliance improved from 55% in 2011 to 68% in 2015. Also the AHRS consumption increased from 13 L / 1000 patient-days in 2013 to 15 L in 2014 and 19 L in 2015. Our ICT are repeatable

**Disclosure of Interest**

None Declared

### P333

#### Role of infection control nurse-directed patient empowerment program in hand hygiene in a Chinese population

Shuk Ching Wong[1], Winnie Li[1], Judy Fung[2], Joseph Yeung[3], Anders Yuen[4], WK Lo[2], KY Yuen[5], Vincent Cheng[6]
[1]Infection Control Team; [2]Department of Medicine; [3]Department of Surgery; [4]Central Nursing Department, Tung Wah Hospital; [5]Department of Microbiology, The University of Hong Kong; [6]Infection Control Team, Queen Mary Hospital, Hong Kong, China
**Correspondence:** Shuk Ching Wong
*Antimicrobial Resistance and Infection Control* 2017, **6(Suppl 3):**P333

**Introduction:** Patient participation is increasingly recognized as an important element in hand hygiene campaign. However, empowerment program is a great challenge in the Chinese culture, where respect and submission to those of higher status, including the healthcare professionals, is a core value to maintain harmony in the Chinese society.
**Objectives:** To assess the role of infection control nurse-directed patient empowerment program in hand hygiene in a Chinese population.
**Methods:** Patients' willingness in participation in a hand hygiene empowerment program was assessed in an extended-care hospital of 550-beds between May and June 2016 using a standard questionnaire. Patients' acceptance in the empowerment program before and after a 15-minutes face-to-face education by ICNs was reported.
**Results:** Of the 130 recruited hospitalized patients with a mean age of 65.4 years, 63 (48.5%) were of primary education level or below. Majority of patients (120, 92.3%) realized that healthcare workers (HCWs) should clean their hands before touching patients, despite that 124 (95.4%) patients had not asked healthcare workers to perform hand hygiene. Seventy (56.5%) of 124 patients trusted HCWs would have already cleaned their hands, 41 (33.1%) considered it should be the responsibility of HCWs to clean hands, whereas 21 (16.9%) did not want to challenge HCWs by questioning their professional ability. Patient willingness in participating in a hand hygiene empowerment program significantly increase from 6 (4.6%) to 71 (54.6%), ($p < 0.001$, McNemar's test) after provision of education by ICNs.
**Conclusion:** ICNs play an active role to facilitate the implementation of patient empowerment in hand hygiene program in a Chinese healthcare setting.

**Disclosure of Interest**

None Declared

### P334

#### Identifying optimal pass–fail criterion for hand hygiene technique

Kamilla Nagy[1], Akos Lehotsky[2], Szava Bansaghi[3], Tamas Haidegger[3]
[1]Faculty of Medicine, University of Szeged, Szeged; [2]Semmelweis University, Budapest; [3]HandInScan, Debrecen, Hungary
**Correspondence:** Tamas Haidegger
*Antimicrobial Resistance and Infection Control* 2017, **6(Suppl 3):**P334

**Introduction:** Hand hygiene is one of the most important components of infection prevention.
**Objectives:** The aim of the study was to observe the inherent performance of HCW and visitors, and to set a pass–fail criterion for the future. While it is well understood that a complete, 100% coverage of the hands should be achieved during hand hygiene, the practice shows that it is extremely hard to make people achieve that on a regular bases.
**Methods:** From August 2016 to January 2017, a complex hand hygiene monitoring program took place at the Albert Szent-Györgyi Health Center (Szeged, Hungary). As part of the program, volunteer health-care workers (HCW) and also visitors had a chance to monitor their own hand hygiene performance with a software-based hand hygiene control device (Semmelweis Scanner, produced by HandInScan). During the monitoring period, 1648 hand hygiene events were recorded, and analyzed retrospectively.

**Results:** In the study, three pass–fail criteria were analyzed: a hand hygiene event was considered sufficient if at least 90, 95 or 97% of the hands' surface was covered with the handrub (Visirub, produced by Paul Hartmann). The increasing standard resulted in dramatic drop in success rate. On the palmar side, which is considered to be the most critical, 7.83%, 13.54% and 20.81% of the participants failed to achieve sufficient coverage, according to the 90, 95 and 97% pass ration, respectively. The number were significantly higher on the dorsal side. In total, only 259 people achieved a complete 100.00% coverage.
**Conclusion:** Based on the findings, it becomes clear that setting a goal for total quality hand hygiene may be overambitious in a hospital environment, since numerous failure on the trials may make the HCW disappointed and undermotivated. Future hand hygiene campaigns should also focus on how to disinfect the back of the hands more effectively.
**Disclosure of Interest**
K. Nagy: None Declared, A. Lehotsky Other conflict with: Founder, HandInScan, S. Bansaghi Employee of: HandInScan, T. Haidegger Other conflict with: Founder, HandInScan

### P335

#### Evidence-based hand hygiene: microbiological validation of the fluorescein training

Akos Lehotsky[1], Szava Bansaghi[2], Peter Szeremy[3], Laszlo Szilagyi[4], George Weber[1], Tamas Haidegger[5]
[1]Semmelweis University, Budapest; [2]HandInScan, Debrecen; [3]Faculty of Medicine, University of Szeged, Szeged, Hungary; [4]Sapientia Univeristy, Cluj, Romania; [5]Obuda University, Budapest, Hungary
**Correspondence:** Akos Lehotsky
*Antimicrobial Resistance and Infection Control* 2017, **6(Suppl 3):**P335

**Introduction:** Hand hygiene is the most efficient tool in fighting nosocomial infections. Besides compliance, special attention should be paid to technique. Flourescent trial (when hand hygiene is performed with an UV-dye containing handrub) is the most common way to examine the hand hygiene technique.
**Objectives:** Our study investigated whether the qualifying flourescent regions on the hands are disinfected in fact.
**Methods:** Artificial hand phantoms were fabricated from plastic and covered with cow skin to fit into regular-size Petri dishes. The phantoms were soaked into 0. 5 McF Staphylococcus epidermidis suspension. Selected regions were treated with flourescent hand rub (Visirub from Paul Hartmann AG, Heindenheim, DE), then images of the samples were recorded under 360 nm UV-A light (where the dye shines the most). Right afterwards, the phantoms were stamped onto blood agar. Plates were incubated at 37 °C for 48 hours. After culturing, image of S. epidermidis colonies were also recorded. Corresponding pairs of pictures (n = 25) were fed to a robust software image registration algorithm, thus obtaining pixel-to-pixel mapping between the two images (under UV and after-cultivation). This mapping enabled us to statistically evaluate the extent to which pathogen-free regions matched the disinfected areas of the hand phantom.
**Results:** All samples were evaluated successfully. Coverage areas were counted and matched against each other. Average sensitivity and average specificity values were $0.95 \pm 0.03$ and $0.98 \pm 0.02$, respectively. This high sensitivity and specificity means that pictures recorded in UV-light are technically show same same coverage than of the results of the agar plates after culturing.
**Conclusion:** We were able to prove that regions on the hand treated sufficiently with the UV-dye containing hand rub are in fact disinfected; not developing colonies after a regular blood agar culturing. This finding confirms that the flourescent trial is indeed a reliable method for verifying individual hand hygiene technique.
**Disclosure of Interest**
None Declared

### P336

#### Hand hygiene: linking training to implementation and outcomes

Finda Mayah-Toto[1], Kayla Enriquez[2], Kanagasabai K. Udhayashankar[3], Michelle Niescierenko[4]
[1]Obstetrics Department, Redemption Hospital, Monrovia, Liberia; [2]Emergency Department, University of California, San Francisco, San Francisco, United States; [3]Pediatric Department, John F. Kennedy Medical Center, Monrovia, Liberia; [4]Global Health Program, Harvard Medical School, Boston, United States
**Correspondence:** Finda Mayah-Toto
*Antimicrobial Resistance and Infection Control* 2017, **6(Suppl 3):**P336

**Introduction:** Healthcare workers were the highest risk group for contracting Ebola Virus Disease (EVD) during the West African outbreak, due to the lack of Infection Prevention and Control (IPC) practices within healthcare facilities. The *Safe Quality Services Package* (SQS) was implemented in Liberia in 2015/2016 and sought to address these IPC gaps and promote better practices, specifically hand hygiene (HH) compliance. HH has proven to be the best way to prevent healthcare associated infections, protecting healthcare workers, patients and community members.
**Objectives:** To assess if HH compliance at public Liberian hospitals was 80% or higher, after implementation of SQS to all healthcare workers
**Methods:** Hand Hygiene audits were conducted at 24 public hospitals in Liberia from October 2016 - January 2017. Data collection was done through direct observation of healthcare worker hand hygiene practice before and after patient contact. Observation was done for 20 minutes duration, on different hospital wards at different times of the day to ensure representation of all cadre's of healthcare workers were observed. Average HH compliance was calculated for each hospital as well as across all facilities.
**Results:** A total of 507 healthcare worker hand hygiene practices were observed across 24 hospitals. Average HH compliance across all facilities was 46% with individual facility results ranging from 11% - 86%. One out of 24 hospitals achieved HH compliance above 80%. Analysis by cadre showed a range of compliance from 20% - 61%.
**Conclusion:** Healthcare worker HH compliance is paramount in preventing the spread of infections. Despite SQS focus on this key concept, overall HH compliance was found to be low, with some cadres performing better than others. This highlights that training and education, although essential, are not enough to encourage behavior change in Liberian public hospital healthcare workers. Further support via mentorship and hand hygiene tool kits is needed to achieve a compliance of 80% or better.
**Disclosure of Interest**
None Declared

### P337

#### Optimizing hand hygiene action: handrubbing for 15 seconds with hand size customized volume

Daniela Pires, Herve Soule, Fernando Bellissimo-Rodrigues, Marlieke EA de Kraker, Didier Pittet
Infection Control Programme, University of Geneva Hospitals, Geneva, Switzerland
**Correspondence:** Daniela Pires
*Antimicrobial Resistance and Infection Control* 2017, **6(Suppl 3):**P337

**Introduction:** Previous studies on hand hygiene (HH) performance after *E. coli* contamination showed that handrubbing for 15 seconds (s) was non-inferior to 30s and that the volume of alcohol-based handrub (ABHR) used should be customized to hand size.
**Objectives:** We tested the combination of these two parameters on bacterial reduction on healthcare workers' (HCWs) hands according to the type of bacteria and contamination levels.

**Methods:** Using the EN 1500 experimental study design, 15 HCWs with extensive experience in HH rubbed their hands with a volume of isopropanol 60% v/v customized to their hand size and calculated to obtain 2 $log_{10}$ bacterial reduction. The following sequence was repeated: hand contamination (*E. coli* or *S.aureus*, $10^8$ or $10^6$ cfu/mL), baseline fingertips sampling, handrubbing (15 s or 30s) and resampling. For each bacterium and concentration, two sequential fingertips samplings were also performed to check bacterial reduction due to sampling (control condition). A generalized linear mixed model was used, with random intercept for subject, to assess the $log_{10}$ bacterial reduction (from baseline to resampling, subtracting the control), adjusting for handrubbing duration, bacterium and baseline contamination. The interactions between duration/bacterium and duration/baseline contamination were assessed.
**Results:** A mean bacterial reduction of 2.27 $log_{10}$ (SD ± 1.2) and 2.02 $log_{10}$ (SD ± 1.05) were obtained after handrubbing for 30s or 15 s, respectively. After fitting the model, the difference in reduction achieved after 30s or 15 s was not significant (-0.23 $log_{10}$; 95% confidence interval (CI) -0.63 0.16; P = 0.246). No significant association was found between baseline contamination and bacterial reduction (P = 0.065). The reduction achieved with *E. coli* was significantly lower than with *S. aureus* (-1.04 $log_{10}$ 95%CI -1.47 -0.60; P < 0.001). The tested interactions were not significant.
**Conclusion:** Handrubbing for 15 s *vs.* 30s with a hand size-customized volume of ABHR yielded no significantly different bacterial reduction, irrespective of bacterium or contamination levels. Bacterial reduction was associated with bacterium type; *E. coli* seems to be more resilient. The clinical significance of these results require further research.
**Disclosure of Interest**
None Declared

### P338

#### Should in-vitro based efficacy testing influence clinical recommendations for hand antisepsis?

Ojan Assadian
Institute for Skin Integrity and Infection Prevention, University of Huddersfield, Vienna, Austria
*Antimicrobial Resistance and Infection Control* 2017, **6(Suppl 3):**P338

**Introduction:** For alcohol-based hand rubs, the European Norm EN 1500 requires testing at 2 x 30 seconds (total: 1 minute). However, based on the testing norm, the currently recommended clinical application time of 30 seconds is longer than the actual time spent in clinical practice.
**Objectives:** The objective of this study was to investigate if a shorter application time of 15 s is microbiologically not inferior to the requirements of the EN 1500 and if such shortening may positively influence compliance with the frequency of hand antisepsis.
**Methods:** In-vitro experiments were conducted to determine the antimicrobial efficacy of different hand rubs within 15 s, followed by clinical observations to assess the effect of a shortened hand antisepsis procedure (group 1 = 15 seconds; group 2 = 30 seconds) performed by registered nurses under clinical conditions in a neonatal intensive care unit. Independent observers monitored the frequency of hand antisepsis during shifts.
**Results:** All tested hand rubs fulfilled the requirement of being equal or even significantly higher effective within 15 s when compared to a reference alcohol propane-2-ol 60% (v/v) within 2 x 30 s. Microbiologically, reducing the application time to 15 s had a similar effect when compared to a 30 s hand rub, but resulted in significantly increased frequency of hand antisepsis (7·9 times/h ± 4·3 vs. 5·8 times/h ± 2·9; P = 0·05) in a neonatal intensive care unit.
**Conclusion:** When different alcohol-based hand rubs are compared, it is imperative to test under identical conditions. The EN 1500 requires testing of 3 ml for 30 seconds followed by a second application of 3 ml for 30 seconds. However, 6 ml of hand rub are rarely applied over an application time of 1 minute in clinical practice. Therefore, agreed standardised test procedures stipulated in norms and testing standards must not be confused with requirements and conditions commonly found in clinical practice, no matter how close such phase 2/ step 2 test conditions approach clinical conditions.
Time pressure and workload are recognized barriers to compliance. Therefore reducing the recommended time for hand antisepsis in clinical practice, using tested and well evaluated hand rub formulations, may be a further step to improve compliance towards hand hygiene in clinical practice.
**Disclosure of Interest**
O. Assadian Grant/Research support from: Research supported by Schulke, B.Braun, Antiseptika, Saraya, Bode/Hartmann

### P339

#### A comparison of the efficacy of liquid, gel and foam presentations of alcohol based hand rub

Martyn A. C. Wilkinson[1], Christina R. Bradley[1], Kevin Ormandy[2], John Hines[2]
[1]Hospital Infection Research Laboratory, Queen Elizabeth Hospital Birmingham, Birmingham; [2]Deb IP, Derby, United Kingdom
**Correspondence:** Martyn A. C. Wilkinson
*Antimicrobial Resistance and Infection Control* 2017, **6(Suppl 3):**P339

**Introduction:** Alcohol based hand rubs are widely used for hygienic hand disinfection and are presented in different formats i.e. liquid, gel or foam.
**Objectives:** This study was designed to determine if there is any difference in efficacy between these 3 formats. In addition, an assessment of the drying time for these formats was carried out.
**Methods: Efficacy**: An EN1500 test was carried out with 20 volunteers. The reference product was 2 x 3 ml of 60% isopropyl alcohol (IPA) for 60 seconds, as described in EN1500. The three test products were 3 ml of 60% IPA in liquid (distilled water), gel or foam form. Each volunteer tested all three products, their order of application decided by a Latin square design.
**Drying time**: Two different volumes of 60% IPA in liquid, gel or foam format were applied to the hands of 15 volunteers. They self-reported when the hands were dry and the time was recorded. The time taken was rated on a 3 point scale; too short, OK and too long.
**Results:** The $log_{10}$ reduction obtained by the reference was 5.39 and for the liquid IPA, gel IPA and foam IPA were 4.19, 4.26 and 4.22 respectively. All of the products failed to meet the criteria of EN1500 (2013), with margins of inferiority greater than 0.6 $log_{10}$ units. However, the difference in mean $log_{10}$ RF between the products was not significant (ANOVA, p = 0.961). Unsurprisingly, the drying time increased with volume (p < 0.001) for all formats. In addition, both foams and liquids are associated with shorter drying times than gels (p < 0.05). Volunteers perceived the liquid format to dry more quickly than the gel or foam (p < 0.05).
**Conclusion:** This study showed no difference when 3 formats of the same formulation of 60% IPA i.e. in liquid, gel or foam format, were tested in the same manner using the methodology described in EN 1500. Gels take notably longer to dry than either liquids or foams, which dry in very similar times. User perception however is that liquids dry faster than the other formats. Therefore while foams appear to offer a very useful balance between drying time and controllability, some user education may be required to overcome concerns. The decision on which product to use will be determined by user preference as there is no difference in efficacy.
**Disclosure of Interest**
M. Wilkinson: None Declared, C. Bradley: None Declared, K. Ormandy Employee of: Deb IP, J. Hines Employee of: Deb IP

### P340

#### Extent of microbial contamination of water used for handwashing in healthcare facilities in Tanzania

Hamisi M. Malebo[1], Robert M. Njee[2], Eva Sosovele[3], Emmanuel A. Makundi[1], Godfrey Mubyazi[1], Irene Mremi[1], Joseph Hokororo[2], Honest Anicetus[2], Hussein Mohamed[4], Yolanda J. Mbatia[1], Calister P. Imeda[1], Doris Mbatta[1], Susan F. Rumisha[1], Julius J. Massaga[1]
[1]National Institute for Medical Research; [2]Ministry of Health, Community Development, Gender, Elderly and Children (MoHCDGEC); [3]Department of Molecular Biology and Biotechnology, University of Dar es Salaam; [4]Muhimbili University of Health and Allied Sciences (MUHAS), School Of Public Health and Social Sciences, Dar es Salaam, Tanzania, United Republic of
**Correspondence:** Hamisi M. Malebo
*Antimicrobial Resistance and Infection Control* 2017, **6(Suppl 3):**P340

**Introduction:** Harmful microorganisms can be transferred to hands from contaminated water used for handwashing. Contaminated hands of healthcare with disease causing pathogens is a well-recognized cause of common source outbreaks of healthcare acquired infections (HCAIs).
**Methods:** Ninety six healthcare facilities (HCFs) in seven districts in Tanzania were involved in the study. Water samples from handwashing facilities and hand washes from healthcare workers and clients were collected, analysed and tested for bacteria.
**Results:** Four different bacterial species were detected. Bacterial isolates were found among samples from all the districts, Escherichia coli was prevalent in 46.5% whereas Proteus spp., Pseudomonas spp. and Klebsiella spp. were detected in 96% of the water samples from hand washing facilities in the visited HCFs. Total coliform (TC) in water samples were found to range from 0.036 - 110 MPN/mL whereas Fecal Coliform (FC) ranged from 0.036 to 4.6 MPN/mL ($P < 0.01$). A higher degree of microbial count ranging from 1.1 to 6.8 x 106 CFU/mL from HCFs Staff and Clients hand washes were observed ($P < 0.01$). Microbes were found in 94.4% of the hand washes from staff and clients.
**Conclusion:** Although handwashing has the ability to get rid of microbes, handwashing using contaminated water in hand washing facilities in healthcare settings as found in this study, could be considered a possible contributor among other transmission routes of HCAI and may be of public concern.
**Disclosure of Interest**
None Declared

### P341

#### Withdrawn

### P342

#### Characterization of bacteria flora on the hands of healthcare workers

Olayemi O. Ayepola, Louis Egwari, Grace Olasehinde, Olamide Adedeji, Omotola Badejo
Biological Sciences, Covenant University, Ota, Nigeria
**Correspondence:** Olayemi O. Ayepola
*Antimicrobial Resistance and Infection Control* 2017, **6(Suppl 3):**P342

**Introduction:** The most common cause of healthcare-associated infections is person-to-person transmission of nosocomial pathogens via the hands of healthcare personnel. Hand hygiene is one of the most important measures to prevent nosocomial infections in the healthcare setting but healthcare workers' compliance cannot be ascertained.
**Objectives:** This study was carried out to evaluate the efficacy of hand hygiene in reducing the burden of bacterial hand contamination in 200 healthcare workers in four selected hospitals in Ota, Ogun State, Nigeria.
**Methods:** A total of 400 swab samples were collected from the hands of healthcare workers before and after hand washing with soap or administering an alcohol based rub. Bacterial isolates were identified by established microbiological methods. Antimicrobial susceptibility profile of the bacterial isolates was tested by disc diffusion method. A significant reduction in bacterial load was observed with the alcohol based hand rub compared to hand washing with soap.
**Results:** A significant reduction in bacterial load was observed with the alcohol based hand rub compared to hand washing with soap. *Staphylococcus aureus* was the most prevalent isolate (53.20%), followed by *Escherichia coli* (29.80%) and *Pseudomonas aeruginosa* (17.10%). The results of the antimicrobial susceptibility testing showed varied resistance to the antibiotics tested. Among *S. aureus* isolates, 84% of the isolates were resistant to penicillin, 84% to trimethoprim/sulphamethoxazole and 8% were methicillin resistant. Most of *Escherichia coli* and *Pseudomonas aeruginosa* isolates were susceptible to the tested antibiotics.
**Conclusion:** This study has highlighted the importance of promoting hand hygiene practices within the hospital environment to reduce infections caused by multidrug resistant pathogens.
**Disclosure of Interest**
None Declared

### P343

#### Methodical approaches to evaluation of skin antiseptics adopted in european union countries and in Russia

Vasily Akimkin, Tatiana Shestopalova
Federal Budget Scientific Institution "Scientific Research Disinfectology Institute" of Federal Service for Surveillance on Consumer Rights Protection and Human Well-being, Moscow, Russian Federation
**Correspondence:** Vasily Akimkin
*Antimicrobial Resistance and Infection Control* 2017, **6(Suppl 3):**P343

**Introduction:** We analyzed application regimes of skin antiseptics that underwent pre-registration studies in various testing laboratories of European Union countries and in Russia over the past 15 years. It is established that in the European Union skin antiseptic efficacy evaluation studies have less criteria compared to efficacy evaluations adopted in Russia. Thus, in accordance with European standards, skin antiseptics efficacy is studied in relation to skin contaminated with *E. coli*. Russian recommendations include evaluation of antiseptic efficacy both with respect to skin contaminated with *E. coli* and with respect to skin with general microbial contamination.
**Objectives:** In this regard, Scientific Research Disinfectology Institute specialists objective was a comparative evaluation of skin antiseptics examination methods and application recommendations in a view of their mutual harmonization at the international level.
**Methods:** In the course of the scientific experiment, a parallel study of efficacy and safety of more than 30 different skin antiseptics was carried out according to methods adopted in the Russian Federation (Guideline R 4.2.2643-10 "Methods for laboratory testing and testing of disinfectants to assess their effectiveness and safety") and methods established in the EU (EN 1500: 1997 "Chemical disinfection and antiseptics - hand hygiene products - test method and requirements").
**Results:** It is established that, despite the difference in applied evaluation methods and criteria for assessing efficacy of skin antiseptics adopted in the countries of the European Union and in Russia, both approaches can be used to obtain required information on parameters of tested antiseptics.
**Conclusion:** The obtained results serve as a basis for the use of test data, which was obtained in accordance with European standards, in Russia for evaluation of skin antiseptics of European manufacturers. Further studies of Scientific Research Disinfectology Institute specialists are being carried out with respect to scientific justification of a possibility to harmonize and improve methods of skin antiseptics examination in order to develop a single document regulating efficacy and safety evaluation procedure.
**Disclosure of Interest**
None Declared

### P344

#### Effect of the duration of use on the properties of two hydroalcoholic solutions commonly used in Benin

Victorien T. DOUGNON, Anges Dossou, Laurencia Dokoundé, Honoré Bankolé, Lamine Baba-Moussa
UNIVERSITY OF ABOMEY-CALAVI, CALAVI, Benin
**Correspondence:** Victorien T. DOUGNON
*Antimicrobial Resistance and Infection Control* 2017, **6(Suppl 3):**P344

**Introduction:** The poor adherence to hand hygiene by healthcare personnel sparked mass production of alcoholic solutions usually stored in vials or providers. Depending on their frequency of use, these solutions may remain in these bottles for a long time. This is a factor that can influence their effectiveness.
**Objectives:** This therefore required to assess the impact of the duration of use of these solutions on the conservation of their antiseptic properties. This is the purpose of this study.
**Methods:** To do this, two alcoholic solutions, one commercial and the other prepared and used in a daily basis of the Benin health center, were tested on three reference strains: *Staphylococcus aureus* ATCC 43300, *Escherichia coli* ATCC 27853 and *Pseudomonas aeruginosa* ATCC 35218. The effectiveness of each gel was then monitored over a period of 30 days.
**Results:** At the end of the study, only the commercial solution was found active against the three reference strains tested with respective inhibition diameters of 53,5 mm for *Staphylococcus aureus*, 28 mm for *Escherichia coli* and 21 mm for *Pseudomonas aeruginosa*. The effective dose was 50 µl. For a dose of 100 µl, respective inhibition zones of 71; 49 and 41 mm were obtained for the three strains. The effectiveness over time for each gel reveals that after 30 days, regardless of the dose used, the effectiveness of the commercial solution remains constant. All these results show that not all hydroalcoholic solutions used in our health centers are systematically effective.
**Conclusion:** It is therefore urgent that actions evaluating the antibacterial performance of these products should be carried out by the authorities before they are placed on the market. Support from development partners is essential. This would guarantee the health protection of the populations.
**Disclosure of Interest**
None Declared

### P345

#### Approaches to detecting gram-negative bacteria in the hospital environment and on hands in an African Hospital setting

Muchaneta Mugabe[1], Marcelyn Magwenzi[2], Valerie Robertson[3], Alex Aiken[4]
[1]Microbiology, National Microbiology Reference Laboratory; [2]Microbiology; [3]Infection control & Microbiology, University of Zimbabwe, Harare, Zimbabwe; [4]Tropical medicine and Hygiene, London school of Hygiene and Tropical Medicine, London, United Kingdom
**Correspondence:** Muchaneta Mugabe
*Antimicrobial Resistance and Infection Control* 2017, **6(Suppl 3):**P345

**Introduction:** Emerging evidence indicates frequent transmission of antibiotic resistant Gram-negative bacteria in hospital settings in African countries. However, there is limited information on low-cost approaches to detection of such bacteria, either in the near-patient hospital environment or on the hands of healthcare workers (HCW). We explored methods for the detection and measurement of Gram-negative bacterial load relevant to an African hospital setting.
**Objectives:** To identify low-cost approach to detect gram negative bacteria
**Methods:** We performed environmental swabs quantifying Gram-negative contamination and ATPase bioluminescence measurements in paediatric wards in two tertiary hospitals in Harare, Zimbabwe. We used hand-print and hand-rinse methods to examine the hands of adults in the same wards. Samples were plated onto MacConkeys agar for enumeration of colony forming units (cfus). Antibiotic susceptibility testing was performed for a randomly-selected sub-set of isolates by standard methods.
**Results:** In May 2015, we performed 991 environmental swabs, from beds and room surfaces. Gram-negative bacteria were detected in 15% (149/991 of samples),over 100 cfus were enumerated in 6% (61/991) of samples. In 474 samples paired with ATPase bioluminescence readings, these correlated well with Gram-negative bacterial counts (coefficient of association 2.21, 95%CI 1.29-3.12, $p < 0.001$). one hundred and fifty-two samples were collected from hands of HCW and parents/guardians. Hand contamination with Gram-negative bacteria was found in 91% of adults (hand-rinse method mean = 14.6 cfu; IQR 3-65). Amongst 92 *Enterobacteriaceae* recovered from the hospital environment (n = 43) and hands (n = 49), the prevalence of resistance to gentamicin and cefpodoxime were very high (62% and 76% respectively).
**Conclusion:** Contamination of the near-patient hospital environment and the hands of adults with multi-drug resistant Gram-negative *Enterobacteriaceae* was common in these wards, representing a substantial risk to patients. Methods used in these evaluations could form objective markers of progress when attempting to reduce the risk of nosocomial infections in an African hospital setting.
**Disclosure of Interest**
None Declared

### P346

#### Modelling the effect of surface cleanliness and health care worker hand hygiene on hcai risk

Cath J. Noakes[1], P.Andrew Sleigh[1], Amir Khan[1], Umberto Armani[1], Sue Bale[2], Elizabeth Waters[2], Marco-Felip King[1]
[1]School of Civil Engineering, University of Leeds, Leeds; [2]Research & Development Department, NHS, Newport, United Kingdom
**Correspondence:** Marco-Felip King
*Antimicrobial Resistance and Infection Control* 2017, **6(Suppl 3):**P346

**Introduction:** Introduction: Improving hand hygiene compliance in hospitals is difficult[1] and surface cleanliness has been shown to relate directly to infection risk[2].
**Objectives:** This research presents a mathematical model that shows that a equilibrium point exists between investing more into hand disinfection and improving surface cleaning.
**Methods:** Methodology: This research presents a mathematical model for predicting hand colonisation of healthcare workers and investigates the likelihood of infection transmission from environmental sources. A multi-objective optimisation genetic algorithm is used to investigate the trade-off between improving surface cleaning and hand hygiene compliance with the view of minimising current infection risk.
**Results:** Results and Discussion: The study suggests that investing in surface cleanliness has a stronger effect on infection rates than improving current levels of hand hygiene. In addition, the current system [2] performs optimally only 11% of the time. 2.5 cfu/cm$^3$ is an optimal point for high touch surface cleanliness levels. The model predicts that a 10% reduction of surface contamination equates to an 8% decrease in infection risk. After 75% hand hygiene compliance, returns on infection risk reduction become increasingly smaller.
**Conclusion:** Conclusions: A framework to reduce infection risk in hospital rooms by optimising the effort put into surface cleaning and hand hygiene is introduced. The mathematical model provides a tool to quantitatively evaluate in terms of cost, the influence of hospital room design on infection risk.

**References**
[1] M. F. King, C. J. Noakes, P. A. Sleigh, S. Bale, and L. Waters, “Relationship between healthcare worker surface contacts, care type and hand hygiene: An observational study in a single-bed hospital ward,” *J. Hosp. Infect.*, vol. 94, no. 1, pp. 48–51, 2016.
[2] M.-F. King, C. J. Noakes, and P. a. Sleigh, “Modelling environmental contamination in hospital single and four-bed rooms,” *Indoor Air*, vol. 25, no. 6, pp. 694–707, 2015.

Disclosure of Interest
None Declared

### Infection prevention and control (IPC) indicators monitoring

#### P347

#### Automation – a boon to data driven infection control programs

Smita Sarma[1], Kanchan Raghuwanshi[1], Nikhil Kumar[2], Vaibhav Khurana[2], Sharmila Sengupta[1], Yatin Mehta[2], Pankaj Sahni[2]
[1]Microbiology and Infection Control; [2]Medanta- The Medicity, GURGAON, India
**Correspondence:** Smita Sarma
*Antimicrobial Resistance and Infection Control* 2017, **6(Suppl 3):**P347

**Introduction:** Collection and analysis of surveillance data is time consuming and laborious if done manually. Using checklists while doing audit helps in uniform collection of data but it is time consuming and inconvenient. Automation can help in easy capture, analysis, interpretation and dissemination of data. In our hospital we have designed an automated tool which helps in easy capture of data through predefined digital checklists ready to be used on tablets by the infection control nurses during their regular audits
**Objectives:** This study aims at exploring the impact of automation in effectiveness of infection control surveillance process
**Methods:** The process of automation was done in the following steps
1. The content of the checklist was finalised by infection control team
2. The digital checklists were created as tablet-ready web-based forms using easily available software
3. The checklist portal was hosted on a server on existing hospital IT infrastructure and made available on tablets via Wifi to infection preventionists
4. The data entry done in digital checklists was configured to be stored in the hospital's database
5. A business intelligence tool was used to design live dashboards to give the output in the form of auto populated charts and reports ready for review and interpretation
**Results:** Automation of 15 infection control surveillance checklists for a 1200 bedded hospital, there was a total saving of 215 man hours (26 man days) in a month. This helped in cutting down the non value added activities such as physical form filling, transcribing to a computer, data cleaning, compilation and chart making
**Conclusion:** Automation increased the efficiency and accuracy of the surveillance process and provided a powerful tool to standardize the data collection, eliminating non value added tasks and preventing errors. Surveillance data became readily available for both internal review of the team as well as for all the relevant stakeholders in the organisation in real time. Not only does this makes the overall process more efficient, but also gives the infection control preventionist more time and resources to analyze the outcomes and engage in training activities. In today's era of technology available in India, this process saves money, efforts and manpower utilisation.
**Disclosure of Interest**
None Declared

#### P348

#### User acceptance of an electronic tool to implement the ecdc point prevalence survey in German hospitals

Michael Behnke, Luis A. Peña Diaz, Alexander Gropmann, Seven J. Aghdassi, Petra Gastmeier
Institut for Hygiene, Charité – University Medicine Berlin, Berlin, Germany
**Correspondence:** Michael Behnke
*Antimicrobial Resistance and Infection Control* 2017, **6(Suppl 3):**P348

**Introduction:** The European Centre for Disease Prevention and Control (ECDC) has organized a European point prevalence survey (PPS) of healthcare-associated infections (HAI) and antibiotic usage (AU). German hospitals took part in the year 2016.
**Objectives:** To develop a web-based software tool (PPS Portal) to simplify the data entry and data analysis process.To measure the usability of the tool.
**Methods:** We implemented the PPS Portal based on the ECDC specifications for a light and full protocol. It represents the ECDC data forms in a hierarchical tree structure. The target users of the portal are mostly Infection Control Practitioners. Additional functionalities like automatic user registration, validation of the data, import and export of the data in ECDC-compatible formats are provided.
To measure the usability of the software, we implemented a system usability scale (SUS) questionnaire. The SUS includes 10 specific questions. Every question has an answer score range from 1 to 5. A master score (in the range from 0 to 100) was calculated out of all answer scores. This master value allows an interpretation of the usability of the software. All registered participants were invited to answer the SUS questionnaire.
**Results:** The PPS Portal was first online in May 2016. The Data entry period for the participating hospitals was from Mai to October 2016. The users entered data for 230 hospitals, 3255 wards and 20761 patients. Validation was processed from September to November 2016. More than 100 validation rules were applied.
177 of 291 users answered the user feedback survey. The mean SUS-Score is 68,5 (IQR: 57,5; 70; 80).
**Conclusion:** The SUS shows an acceptable to good user acceptance of the PPS Portal. To optimize specific functions in the user interface closer examinations are needed. Overall the experience shows that the PPS Portal is stable and easy to use. Its capability to manage multiple countries and networks at one time and its multilingual features allow the usage in different countries at the same time. It will be used in summer 2017 in Switzerland in two different languages.
**Disclosure of Interest**
None Declared

#### P349

#### The effect of automated notifications on isolation of infected patients with super bug (CRE)

Zohra R. Rafique Iqbal, Rozina Roshan, Faisal Mahmood
Infection Control, Aga Khan University and Hospital, Karachi, Pakistan
**Correspondence:** Zohra R. Rafique Iqbal
*Antimicrobial Resistance and Infection Control* 2017, **6(Suppl 3):**P349

**Introduction:** Cross transmission of Multi Drug Resistance Organism (MDRO) has become a challenge for hospitals among these MDRO a super bug i.e. CRE (Carbapenem Resistant Enterobacteriaceae) is increasing worldwide. These patients remained without isolation for couple of hours as there was no trigger or alert system to identify readmitted infected patient with CRE.
**Objectives:** 1. To develop a computerized system to generate isolation alerts when CRE patients are readmitted.
2. To reduce the time to isolation of patients readmitted with CRE from 38 hours per patient per month.
**Methods:** The Plan-Do-Study-Act (PDSA) model, a continuous quality improvement (CQI) tool, was used to improve the isolation rates. **Plan**: In 2016 it was observed that number of CRE patients were increasing from 33 (Jan) to 72 (Aug) and Number of readmitted CRE patients were also increasing 08 (Jan) to 24 (Aug). It took an average of 38 hours per patient to initiate isolation. Problem identification was done through brain storming activity and identified that no computerized system alerts, lack of collaboration of infection control department with bed management office and admission office, lack of knowledge of health care workers about its transmission. **Do**: involved Information Technology (IT) department to put Isolation Alerts in system and automatic emails were generated. Education sessions were conducted for health care workers **Study:** monitoring of the isolation indicator on daily basis. To calculate the hour's patients were not isolated with the reasons. **Act:** all the gaps were identified and rectified and flyers were developed and placed at admission office and bed management office, email receipt list was revised and alerts also displayed at time of bookings
**Results:** After the intervention drastic improvement was seen and we were able to achieve our targets and more than 75% patients were isolated at time of admission. The average hours per patient not isolated also improved from 38 hours to 9.8 hours for all CRE

readmitted patients and when we continued monitoring this data for subsequent month we were able to achieve Zero hours
**Conclusion:** The burden of MDRO is increasing day by day in hospitals thus a computerized alert system plays a significant role in timely identification of infected patients
**Disclosure of Interest**
None Declared

### P350

#### E-health based infection control and antimicrobial stewardship activities: one day sample

Carlos Palos[1], Ana Bispo[1], Vanessa Pinto[1], Paulo Rodrigues[1], Luis França[1], Patrícia Pereira[2], Tiago Lobo[3]
[1]Infection Control and Antibiotics Committee; [2]Clinical Pathology Lab; [3]Pharmaceutical Service, HOSPITAL BEATRIZ ÂNGELO, Loures, Portugal
**Correspondence:** Carlos Palos
*Antimicrobial Resistance and Infection Control* 2017, **6(Suppl 3):**P350

**Introduction:** Hospital Beatriz Ângelo (HBA) is a JCI-accredited, 425-bed, paper-free, general hospital, opened in 2012. E-health, defined as the use of communication and information technologies in the health field, is optimized in order to improve infection control and antibiotic stewardship, allowing real-time interventions at all levels.
**Objectives: OBJECTIVE:** Evaluation of one day e-health based infection control and antimicrobial stewardship activities in a secondary hospital.
**Methods:** A global e-health approach was implemented since opening. Antimicrobial stewardship (AS) is started mainly after real-time e-mail alerts generated by the prescription of conditioned antimicrobials or whenever there's an inadequate reason. AS can also be started whenever alerts are issued by positive microbiology results for blood cultures or epidemiologically important microorganisms (EIM). Bed management for colonized/infected patients is based on an infection control dashboard, integrating several isolation symbols and e-mail alerts automatically generated by Electronic Epidemiologic Questionaire on Admission ((EEQA), a risk assessment tool filled by physicians at patient's admission) or manually inserted whenever new isolation is implemented. Epidemiologic surveillance is based on automatically generated alerts for catheter-associated urinary tract infections, surgical site infections and microbiologic results (bloodstream infection).
**Results:** In one sample day, of 372 inhospital patients, 39 (10,5%) were isolated because of infection/colonization with EIM, while 29 (7,7%) were on contact precautions, awaiting for results of admission screening. Implementation of isolation was done on 2 patients and contact precautions were stopped in 8. Micro alerts where generated for 6 blood cultures, 3 urine cultures and 2 EIM. EEQA isolation procedure alerts were generated in 10 cases. AS interventions were done in 10 situations.
**Conclusion:** E-health is a major tool for infection prevention and control, as well for antibiotic stewardship. Full data integration allows real-time interventions which are especially important for colonization/infection risk assessment, bed management and antibiotic stewardship, improving quality and safety.
**Disclosure of Interest**
None Declared

### P351

#### Management of the control of hospital infection in a hospital of palliative care: indicators as markers of service quality - Brazil

Andreia C. B. P. D. Schunck[1,2], Amanda M. D. P. Jacque[1], Samir Salman[1]
[1]Serviço de Controle de Infecção Hospitalar, HOSPITAL PREMIER; [2]Serviço de Controle de Infecção Hospitalar, INSTITUTO DE INFECTOLOGIA EMILIO RIBAS, São Paulo, Brazil
**Correspondence:** Andreia C. B. P. D. Schunck
*Antimicrobial Resistance and Infection Control* 2017, **6(Suppl 3):**P351

**Introduction:** Hospital infections are a major cause of morbidity and mortality in hospitals, as well as being responsible for an increase in hospitalization time and an increase in additional costs with the patient, becoming a public health problem.
**Objectives:** To evaluate the infection rates of a Hospital for Palliative Care and to verify the incidence of death related to hospital infection.
**Methods:** Retrospective, descriptive study with quantitative approach. The research was conducted in a private hospital in São Paulo, Brazil, from January 2015 to December 2016. Data collection was performed by the Hospital Infection Control Service and data analysis was performed according to the diagnostic criteria for infection Related to the health care of the Long Stay or Psychiatric Hospital Manual of the State Health Department of Health of São Paulo 2016.
**Results:** In 2015 the incidence densities were 3.27 for urinary tract infection, 1.84 for pneumonia, 0.16 for gastrointestinal infection and 0.57 for integument per 1000 day-patients and in 2016 were 3.07 of urinary tract infection, 1.10 pneumonia, 0.20 gastrointestinal infection, and 0.63 tegumentary infection per 1000 patient-days. The overall infection incidence was 5.85 in 2015 and 4.99 in 2016 of infections per 1000 patient-days. The incidence of death in 2015 was 0.61 and in 2016, 0.23 deaths per 1000 patient-days. The results found in this hospital corroborated with the international data of Long Stay Institutions. In the United States, the incidence of global infection ranges from 1.8 to 13.5 infections per 1000 patient-days and the incidence of death 0.04 to 0.71 per 1000 patient-days.
**Conclusion:** This research shows the importance of a Hospital Infection Control Service within a Hospital of Care for infection control, including surveillance, isolation precautions, outbreak control, patient care, and employee.

Disclosure of Interest
None Declared

### P352

#### Withdrawn

### P353

#### Process evaluation of artificial patients' feeding in intensive care

Mohamed Mahjoub[1], Yahia Abouda[1], Nabiha Bouafia[1], Rym Bouriga[2], Hela Ghali[1], Asma Ammar[1], Olfa Ezzi[1], Asma Ben Cheikh[1], Waadia Bannour[1], Radhia Héllali[1], Azza Harrari[1], Mansour Njah[1]
[1]Hospital Hygiene; [2]medical oncology, university hospital Farhat Hached Sousse Tunisia, Jawhara, Tunisia
**Correspondence:** Mohamed Mahjoub
*Antimicrobial Resistance and Infection Control* 2017, **6(Suppl 3):**P353

**Introduction:** Unconscious patients' survival in resuscitation has increased thanks to the intensive care and also to the artificial feeding. Although this last one is generally coordinated jointly with the health institutions' pharmacies, we must take into consideration its qualification.
**Objectives:** Our objectives were to evaluate application conformity degree of good hygiene practices (GPH) throughout the process of artificial feeding, at a Tunisian university hospital, in order to better control its defects.
**Methods:** We conducted an audit of artificial feeding process during three months (June, July and August 2016), from the product's reception up to its administration to patients. We used an evaluation grid accessing the GPH standards' level of application and the respect of health rules by direct and repeated observations but also by interviewing the staff of both intensive care and pharmacy.
**Results:** A total of 86 observations and discussions with healthcare staff have been conducted. The overall hygiene standards' compliance rate applied to the whole process was 65%. The most non compliant step was the storage at hospital central pharmacy (52.5%). On the other hand, despite shortcomings, the highest average compliance rate was patients' administration of the products (75%). Failures' causes have been analyzed by Ichikawa diagram of causes-effect and the SWOT (Strength-Weakness /Opportunities-Threats) analysis.
**Conclusion:** Our study has revealed defects along artificial feeding process, in our hospital, which can be remedied by GPH's application. In order to strengthen food security's systems, all establishment stakeholders must federate their effort and assume their responsibilities.
**Disclosure of Interest**
None Declared

### P354
**Cord care practices and newborn health related implications among mothers in milgoma" Sabon Gari Zaria, North-West, Nigeria**
Onoja-Alexander O. Mary[1], Olorukooba A. AbdulHakeem[2], Igboanusi C. John-Camillus[3], Onoja D. Alexander[4], Sodangi J. Khadeeja[5], Dafile P. Grace[6]
[1]Department of Community Medicine, Ahmadu Bello University Teaching Hospital; [2]Department of Community Medicine; [3]Department of Community Medicine, Ahmadu Bello University Teaching Hospital Zaria, Zaria; [4]Wellcare Initiative Kano, Kano; [5]Department of Medicine, Gombe State University Teaching Hospital, Gombe; [6]Association of Orphan and Vulnerable Children of Nigeria, Abuja, Nigeria
**Correspondence:** Onoja-Alexander O. Mary
*Antimicrobial Resistance and Infection Control* 2017, **6(Suppl 3):**P354

**Introduction:** Umbilical cord care is a cause of concern for mothers' right from birth until its separation. Good cord care practices reduces incidence of neonatal morbidity and mortality while cord care practices vary from place to place, some can be harmful to a newborn
**Objectives:** 1. The aim was to establish various cord care practices and the health related implications among newborns in Milgoma
3.
**Methods:** A descriptive cross-sectional study was conducted among 110 mothers using systematic random sampling. Data was collected using semi-structured interviewer administered questionnaire,on socio-demographic characteristics,cord care practices and health related implications among newborns. Data was analyzed using SPSS version 20.0 and results were presented using Chi square tables and charts
**Results:** Majority of the respondents were within the age group 20-29 years, Hausa, Muslims. Thirty eight percent of respondents used hot compress, while 30% use of methylated spirit, herbs (15%), toothpaste(10%), use of garlic and ginger (4%) and only 1.5% knew about use of cow dung. 31.8% of respondents children have had complication of poor cord practices. Localized infection was the most common poor cord care complication (33.2%), bleeding (25.3%), sepsis (21.8%) burns(9.6%), neonatal tetanus 5.2% and neonatal jaundice has the least (4.8%).There was a significant statistical relationship between educational status of respondents and knowledge of scores(P =0.000)
**Conclusion:** Respondents demonstrated a fair knowledge towards cord care, however majority of them practiced harmful cord care practices and majority of the cord-related complications developed were cord infections. Thus the need for enlightenment of mothers by local heath authorities to stop harmful traditional practices and adopt beneficial ones.
**Disclosure of Interest**
None Declared

### P355
**Infection prevention and control (ipc) performance monitoring in 5 medico-communal centers (CMC) in Conakry, Republic of Guinea**
Abdoulaye Diallo[1], Jacqueline Aribot[1], Mamadou Malal Diallo[2], Aly Doumbia[3], Yolande Hyjazi[4]
[1]M&E; [2]Pre service education; [3]Infection prevention and Control; [4]Country Director, Jhpiego, Conakry, Guinea
**Correspondence:** Abdoulaye Diallo
*Antimicrobial Resistance and Infection Control* 2017, **6(Suppl 3):**P355

**Introduction:** In order to monitor IPC activities in the 5 CMC of Conakry following the training of the providers, periodic evaluations are carried out to measure the level of implementation of IPC performance standards.
**Objectives:** Assess IPC practices in health care facility using IPC performance standards
**Methods:** The evaluation is done through direct observation, an interview and a documentary review by a team of 2 evaluators in 2 days per facility, A summary sheet is used to summarize the results obtained by standards.
**Results:** From December 2014 to October 2016, 5 IPC performance evaluation visits were conducted in the 5 CMC of Conakry, with an average of 5 months interval. Analysis of findings from 8 out of 32 IPC standards showed median performances ranging from 38% to 80%. 100% of CMC reached the required performance threshold (75%) at the 5th assessment.
**Conclusion:** The use of IPC standards for self-assessments improves the performance of health workers. The best performance is linked to the regular use of performance standards for self-assessments to address identified and persistence gaps

**References**
Jhpiego/Johns Hopkins Médicine, Prévention et Contrôle de la maladie à virus Ebola dans les établissements de sante aux ressources limitées (Manuel de référence, octobre 2014)
Jhpiego/Ministère de la Sante, Standards de Performance en Prévention et Contrôle de l'Infection (Octobre 2014)
Disclosure of Interest
None Declared

### P356
**Infection control in acute care hospitals, Finland, 2014**
Dinah K. Arifulla, Jukka Ollgren, Jere Veltheim, Outi Lyytikäinen
NATIONAL INSTITUTE FOR HEALTH AND WELFARE, Helsinki, Finland
**Correspondence:** Dinah K. Arifulla
*Antimicrobial Resistance and Infection Control* 2017, **6(Suppl 3):**P356

**Introduction:** Infection control (IC) requires adequate human resources and activities.
**Objectives:** A nation survey was conducted to describe the coverage and characteristics of these issues in Finland and to evaluate progress during recent years.
**Methods:** Web-based survey was provided via E-mail and mail to all Finnish acute care hospitals. The questionnaire covered information on hospital characteristics, IC staff and activities in 2014. We also asked which information is feasible to update annually and which could be reported publicly.
**Results:** A total of 43 (94%) hospitals responded (5 tertiary, 15 secondary and 23 primary care hospitals); 33 provided intensive care and 38 performed surgery. Of the hospitals, 77% had infectious disease specialists and 30% clinical microbiologists participating in IC activities. They used a median of 26% (range by region, 21–33%) and 9% (range, 0–30%) of their work time on IC activities, respectively. All hospitals had trained infection control nurses (ICN), who used a median of 59% (range, 33–68%) of their work time on IC activities of their own hospitals. The median of hospital beds per full-time equivalent ICN was 204 (range, 175–274). All but one hospital had a link nurse system. The written prevention and control guidelines for urinary tract infection, surgical site infection, bloodstream infection, and pneumonia were available in 37-84% of the hospitals and were variously implemented in practice by training (range by infection type, 37-63%), check list (5-23%), auditing (0-2%), surveillance (56-79%) and feedback (42-60%). Of the hospitals, 15/38 surveyed timing of surgical antibiotic prophylaxis. Influenza vaccine coverage among staff varied regionally between 31-74%. The latter information was favored for public reporting beside the ICN to bed ratio and hand rub consumption.

**Conclusion:** In comparison with the results from our previous survey in 2008, the ICN to bed ratio has improved (2008: 257 vs. 2014: 204). However, the doctors' input in IC is still limited. The unavailability of infection prevention guidelines for the most common infections and lack of tools in implementation cause concern. National guidelines with implementation tools could improve this issue.

**Disclosure of Interest**

None Declared

### P357

#### Reporting of infections associated with care: the barriers to reporting between different actors
### REGIONAL FRENCH STUDY FROM 01/01/2012 TO 02/05/2016

Narbey David[1], Marie-Antoinette INGABIRE[1], Claude BERNET[2], ANNE SAVEY[2], PASCAL FASCIA[1]
[1]ARLIN RHONE-ALPES; [2]CCLIN SUD EST, SAINT-GENIS-LAVAL, France
**Correspondence:** Narbey David
*Antimicrobial Resistance and Infection Control* 2017, **6(Suppl 3):**P357

**Introduction:** The reporting of nosocomial infections (NI) has become mandatory since 26/07/2001. However, although the data show a trend towards increased reporting, many institutions still report too little.

**Objectives:** Make an inventory of the reports in a region and then to try to understand the obstacles to this report.

**Methods:** Longitudinal observational retrospective study of the reports of the identified region from 01/01/12 to 02/05/16 from the e-sin database. Qualitative variables will be described in terms of enrollment and percentage; The quantitative variables, as a mean and their standard deviation. To identify the factors favoring NI reporting, logistic regression analysis will be performed, with the dependent variable being the reporting of at least one report versus more than one report.

**Results:** 902 reports were issued over the study period (164 different institutions). We notice a gradual increase in the number of reports. Public institutions report the most. Infections related to the nature, characteristics or profile of resistance to anti-infectives of the pathogen involved as well as epidemic cases are the most common. After adjustment, 2 variables seem to influence the reporting: NI of a rare or particular character, in relation to local, regional or national epidemiological data, as a result of the pathogen in question and having an infection versus colonization. It should be noted that the large size of the institution which appeared significant in univariate no longer appears after adjustment.

**Conclusion:** In order to try to improve the reporting, it would be useful to have a permanent referral officer in each establishment since it has been shown that the absence of a hygienist practitioner was an obstacle to the validation of an external report. On the other hand, the notions of colonization and infection, even if they represent a difference from a clinical point of view, represent the same risk in terms of hygiene measures. One must not lose sight of the usefulness of reporting which is to rapidly detect unusual infections and take measures for the management of serious and / or elevated nosocomial infectious risk situations.

**Disclosure of Interest**

None Declared

### P358

#### Management of healthcare infectious risks outside health establishments: a survey among liberal practitioners

Mohamed Mahjoub[1], Rym Bouriga[2], Nabiha Bouafia[1], Hela Ghali[1], Asma Ammar[1], Asma Ben Cheikh[1], Olfa Ezzi[1], Waadia Bannour[1], Radhia Héllali[1], Mansour Njah[1]
[1]Hospital Hygiene; [2]medical oncology, university hospital Farhat Hached Sousse Tunisia, Jawhara, Tunisia
**Correspondence:** Mohamed Mahjoub
*Antimicrobial Resistance and Infection Control* 2017, **6(Suppl 3):**P358

**Introduction:** Liberal medical practice, outside health establishments, may supply healthcare associated infections. Good application degree of hygienic practice depends on doctors' perception and training (academic and continuous).

**Objectives:** Our objectives were to specify good hygienic practice (GHP)'s observance degree according to physicians in private practice and to compare two groups' answers relatively to the practitioners age (more or less than 50 years).

**Methods:** A descriptive cross-sectional study was realized in 2016 by means of a pre-tested and auto-administered questionnaire with all the general practitioners of private practice in a coastal city of the Tunisian central east region

**Results:** Participation's rate is 93,1% with a sex-ratio of 1,7. Sixty-two per cent of the general practitioners are less than 50-years-old. A significant difference in favors of people under 50 was reported for the GHP's observance relatively to healthcare activities' waste management (87,3% versus 45,6%; p $10^{-4}$). Respondents of less than 50 years formulate significantly more their wishes to do a training course in BPH (91,5% versus 54%; $p = 10^{-4}$). The victims of blood exposure accidents (BEA) are significantly more prevailing among people over 50 years (62,2% versus 21,1%; $p = 10^{-4}$)

**Conclusion:** Our study reveals importance of in-service training's strengthening for general physicians, especially the oldest at medical exercise with incentive reminders of the updated recommendations to improve the healthcare associated infections (HAI)' risks awareness and their daily optimal management.

Wills to organize HAI' fight and prevention in liberal medical practice are essential. These initiatives require from professionals to seize HAI problem and to launch evaluation process. It is justifiable to develop motivational and compensatory measures to favor work between peers.

**Disclosure of Interest**

None Declared

### P359

#### Particularities of healthcare practice and medical device vigilance: a survey among physicians in a university-hospital

Mohamed Mahjoub[1], Rym Bouriga[2], Nabiha Bouafia[1], Hela Ghali[1], Asma Ammar[1], Asma Ben Cheikh[1], Olfa Ezzi[1], Waadia Bannour[1], Radhia Héllali[1], Mansour Njah[1]
[1]Hospital Hygiene; [2]medical oncology, university hospital Farhat Hached Sousse Tunisia, Jawhara, Tunisia
**Correspondence:** Mohamed Mahjoub
*Antimicrobial Resistance and Infection Control* 2017, **6(Suppl 3):**P359

**Introduction:** Health alert including medical device vigilance (MDV) contribute to care safety in health institutions. Evaluation of MDV systems in different environments of medical practice represents an essential step to support improvement of healthcare's quality.

**Objectives:** The objective is to estimate the knowledge, attitudes and practices (KAP) of physicians relating to the MDV and to compare it between medical and surgical specialties, in the various hospital services of a Tunisian Central East University-Hospital.

**Methods:** A descriptive cross-sectional study was conducted in 2016 including 183 incumbent physicians performing in 24 hospital services, using a pre-established, pretested and self-administered questionnaire,. Seizure and Analysis of the data were made by SPSS software 20.0

**Results:** Response rate was 81.9%, Practicing physicians in medical services accounted for 81.1% of the participants. The doctors who recognize the existence of a standard form of reporting was significantly greater in the medical services than surgical ones (51.9% versus 27.8%; p = 0.04).In addition, physicians who are convinced by the importance of reporting under the MDV was significantly more frequent in surgical services (66.7% versus 32.5%; p = 0.007). The importance of efficient and organized MDV's management was more frequently reported by physicians of surgical services than medical ones (83.3% versus 14.3%; p < 0.001).

**Conclusion:** The institutional strategies for the development of safety culture of care relating to the MDV must take account of the specialty of medical practice in order to better manage healthcare risks and to improve its quality and safety. Adaptation of doctors' KAP about MDV with training-information programs is crucial since they are influenced by the environment of medical practice.

**Disclosure of Interest**

None Declared

### P360

#### Resource consumption due to healthcare-acquired infections

Shmuel Benenson[1], Phillip Levin[2], Allon E. Moses[1], Carmela Schwartz[1], Matan J. Cohen[1]

[1]Clinical Microbiology and Infectious Diseases; [2]Intensive Care, Hadassah-Hebrew University Medical Center, Jerusalem, Israel

**Correspondence:** Shmuel Benenson

*Antimicrobial Resistance and Infection Control* 2017, **6(Suppl 3):**P360

**Introduction:** Available reports of the financial burden incurred by healthcare-acquired infection (HAI) are dependent on local reimbursement contracts and costs limiting the applicability for generation of valid financial schemes.

**Objectives:** We sought to generate a detailed and valid report of the differences in resource consumption between patients who acquired infections during hospital care and severity-status-matched controls.

**Methods:** Matched cohort study; Patients with Central Line Associated Blood Stream Infection (CLABSI), Surgical Site infections (SSI) and *Clostridium difficile* infection (CDI) were identified between 2014 and 2015. We matched three control per patient according to accepted severity indices (SAPS-II, ASA and Charlson, respectively), age, sex, hospital unit, time since admission and surgery type (for SSIs). All resources used during patient care were identified, as were length of stay and mortality.

**Results:** We identified 62 CLABSI events, with 179 control; 76 SSIs, 213 controls; 92 CDI cases, 276 controls. Respective in-hospital mortality rates were 63% vs. 47% (p = 0.03), 4% vs. 1% (p = 0.11) and 27% vs. 12% (p < 0.001). There was an average increase of 28 (CLABSI), 11 (SSI), and 12 (CDI) antibiotic treatment days. CLABSI led to an average increase of 58 blood tests, 11 imaging tests, and 12 more cultures. The respective SSI estimates are 16, 3 and 3 and the CDI estimates are 17, 2 and 4 (all p < 0.001). There were similar average hospital stays between cases and controls: early mortality among cases evened other longer stays. Results were stable under sensitivity analysis examining the control group results' maximum and minimum consumption records. Various other additional resources (TPN, dialysis, repeat surgery etc.) were required for patient care and differed between cases and controls.

**Conclusion:** Our study provides a detailed report of the resource consumption associated with HAI. Unlike previous financial analyses, institutions, administrative bodies and healthcare policy leaders can adapt our findings to identify the local financial implications of HAI and devise incentive/disincentive imbursement schemes to augment infection control initiatives and reduce HAI.

**Disclosure of Interest**

None Declared

## Infection and prevention in immunocompromised hosts

### P361

#### Infection control in immunocompromised patients living in sub-urban slums

Chioma O. Nwuba[1], Ngozi F. Onyemelukwe[2]

[1]Program Implementation, i + Solutions, Abuja; [2]Medical Laboratory Sciences, University of Nigeria, Enugu, Enugu, Nigeria

**Correspondence:** Chioma O. Nwuba

*Antimicrobial Resistance and Infection Control* 2017, **6(Suppl 3):**P361

**Introduction:** In the sub-urban slums of Nigeria fraught with poor sewage disposal practices, sewage spills, lack of sanitation and low socio economic status, prevalence of infectious diseases is high especially amongst immune-compromised patients. Immune-compromised patients are often vulnerable to opportunistic infections including gastro-enteric infections. Such opportunistic infections present with severe clinical symptoms which are more difficult to treat in this group of patients compared to infections in otherwise healthy people.

**Objectives:** To examine the impact of poor sanitation practices on prevalence of gastro-enteric infections amongst HIV patients living in sub-urban slums.

**Methods:** Stool specimens from 202 HIV patients accessing care and treatment services at two sub-urban clinics, were collected and examined through direct observation in saline (0.85% NaCl solution). Lugol's iodine method was used for the detection of ova, larvae, trophozoites and cysts of intestinal parasites using light microscopy. Smears of direct and concentrated specimens were examined by modified acid fast staining for *Cryptosporidium parvum, Isospora belli* and *Cyclospora spp*. CD4 T-lymphocyte count of each patient was estimated using the Becton Dickenson FACS-count system.

**Results:** Enteric parasites were detected in 91(45%) stool samples, of which 54 (59.3%) were opportunistic and 37(40.7%) were non opportunistic. Opportunistic parasites isolated include *C. parvum*, *I. belli* and *Cyclospora spp*. C. parvum (30.8%) was the most frequently encountered pathogen followed by *I. belli* (24.2%) and *Entamoeba histolytica* (23.1%). Opportunistic parasites were isolated in all 42 (20.8%) patients with CD4 count <200 cells/ul. Overall, C.parvum and *E. histolytica* were the most frequently occurring parasites. Opportunistic parasites were undetectable at CD4 count >500 cells/ul while non-opportunistic parasites were detectable only in 3 patients with CD4 count >500 cells/ul.

**Conclusion:** Infection prevention and control strategies in immune-compromised patients living in sub-urban slums of Nigeria should not only focus on the administration of drugs for opportunistic infections but also on the provision of basic sanitary facilities, health education campaigns and resuscitation of the public sanitary inspector's act.

**Disclosure of Interest**

None Declared

### P362

#### Multidrug resistant bacteria in surveillance cultures from hematopoietic stem cell transplant (HSCT) patients

Prasanth C. Bhat[1], Vivek Bhat[2], Navin Khattry[3]

[1]Medical Administration, ADVANCED CENTRE FOR TREATMENT, RESEARCH AND EDUCATION IN CANCER (ACTREC), TATA MEMORIAL CENTRE; [2]Microbiology, TACTREC, TATA MEMORIAL CENTRE; [3]Medical Oncology, ACTREC, TATA MEMORIAL CENTRE, Navi Mumbai, India

**Correspondence:** Prasanth C. Bhat

*Antimicrobial Resistance and Infection Control* 2017, **6(Suppl 3):**P362

**Introduction:** Infections due to multi-drug resistant bacteria is a major contributor to morbidiy and mortality in oncology setting and is particularly true in hematopoietic stem cell transplant (HSCT) patients who have profound underlying immunosuppression. Patients' endogenous flora from the bowel, mouth and skin may be responsible for many of the serious bacterial infections. Surveillance cultures for drug resistant organisms may have a role in alerting the clinician to the possible etiology of subsequently developing infections.

**Objectives:** 1. To conduct bacterial surveillance culture and to understand multi drug resistance pattern of organsim from HSCT patient sample

2. To provide evidence based antibiotic choice for empirical treatment during HSCT

**Methods:** 68 patients who underwent hematopoietic stem transplant were included in the study. Swabs for surveillance culture were collected from the anterior nares and axilla to look for the presence of Methicillin Resistant *Staphylococcus aureus* (MRSA). Faeces cultures were performed to screen for multi-drug resistant Gram negative bacilli including resistance to third and fourth generation cephalosporins, beta- lactam –beta lactamase inhibitor combinations (BLBLIs), vancomycin resistent enterococci and carbapenems.

**Results:** *Escherichia coli* (66) was the commonest organisms isolated followed by *Klebsiella pneumonaie* (17) in the faecal surveillance cultures. Resistance to 3$^{rd}$ and 4$^{th}$ generation cephalosporins was > 50% in *E. coli,* resistance to meropenem was 13.67% and piperacillin-tazobactam was at 30%. Among *K. pneumoniae*, resistance to cefotxime and cefepime was at 35.29%, however all strains were susceptible to the carbapenems. There were 27 isolates of enterococci, of which 18.52% were vancomycin resistant. Five nasal swabs grew *Staphylococcus aureus*, of which one was methicillin resistant (MRSA).

**Conclusion:** There is a high level of antibiotic resistance to cephlosporins and BLBLIs in the endogenous Gram negative organisms of our HSCT patients as evident from surveillance faecal cultures. This information can be useful in empirical treatment of subsequently developing infections.

**Disclosure of Interest**

None Declared

### P363

#### Current situation of piccs in oncohematological patients of a II level hospital

Carmen Escassi, Desiré Macias, Encarnación Román, Samuel Gonzalez, Maria José Pastor, Isabel Fuentes, Maria José Perez, Cristobal León, Pedro Jimenez

Servicio Andaluz de Salud (SAS), Sevilla, Spain

**Correspondence:** Carmen Escassi

*Antimicrobial Resistance and Infection Control* 2017, **6(Suppl 3):**P363

**Introduction:** Central venous catheters (CVCs) are essential in the care of patients with severe processes treated in surgical units, intensive care units and hemato-oncological units. In our hospital since 2014, echocardiographic peripherally inserted CVCs have been introduced as an alternative to traditional CVCs

**Objectives:** Describe the characteristics of infectious complications (IC) that occurred during the use of venous catheters central peripheral insertion (PICC) guided eco technique

**Methods:** A prospective, observational, analytical, and unicentric study of all oncohaematological patients who had a PICC inserted. Study period from March 2014 to September 2016. All socio-demographic variables, mechanical and infectious complications were collected during PICC use. Statistical analysis with SPSS Statistic 23

**Results:** During the study period, 591 PICCs were inserted, of which 344 were oncohematologic patients, of whom 54.9% were women and 45.1% were men. The avarege number of days of implantation was 124.31. The inherent oncological pathology was mainly solid tumor (81.1%), followed by lymphoma (12.25) and leukemia (5.8%). The main reason for requesting the PICC was chemotherapy (84.6%). The complications during catheter use were: suspected infection 5 (3.5%), microbiologically confirmed bacteremia 2 (1.4%), accidental removal of catheter 8 (5.7), local infection and obstruction of PICC 6 (4.3%), rest By termination of treatment or by patient's exitus (not related to infectious complications). The incidence density of IPCC-related bacteremia was 0.6‰. The main reason for catheter removal was completion of treatment (56%) followed by exitus 32 (22.7%) (not related to infectious complication), suspicion of infection 5 (3.5%), bacteriemia confirmed microbiologically 2 (1.4%), Accidental withdrawal of catheter 8 (5.7), the rest were mechanical complications

**Conclusion:** The incidence density of IPCC-related bacteraemias was lower than those published in the national registry (Envin) and a study conducted in our Hematology Unit in 2012.

It seems reasonable to assume that the use of CCIPs is associated with a lower

Incidence of serious complications, related not only to their insertion, but during their use (fewer infectious or thrombotic complications)

**Disclosure of Interest**

None Declared

### P364

#### Pseudo-outbreak of methylobacterium species in a hematological department

Katrine H. Hansen, Marlene Høg, Leif P. Andersen

Department of Clinical Microbiology, RIGSHOSPITALET, Copenhagen, Denmark

**Correspondence:** Katrine H. Hansen

*Antimicrobial Resistance and Infection Control* 2017, **6(Suppl 3):**P364

**Introduction:** *Methylobacterium* species are low pathogenic water borne bacteria occasionally involved in outbreaks in hospital settings. It has been reported as an opportunistic pathogen that may infect immunocompromised hosts.

**Objectives:** Here we describe four patients with central venous catheter (CVC)-related *Methylobacterium* spp. infections in the same department within a period of three months.

**Methods:** Four patients with acute myelogenous leukemia were admitted to the hematological department. Blood samples were drawn routinely from the CVC from all patients and cultured in a commercial blood culture bottles system for seven days. Positive blood cultures were examined by microscopy and grown on blood agar plates. Water samples were collected from taps located at all patient rooms where the patients had been and filtrated on TSA media. Bacteria colonies were identified by the API 20 NE system. Ribotyping was performed on an automatized Riboprinter on isolates from the four patients and two water samples.

**Results:** From April through June 2005 four patients within the same hematological unit had positive blood cultures drawn from CVCs with *Methylobacterium* spp. This indicated that there could be an outbreak with this species since four cases of a rare bacterium occurred in the same unit at the same time. *Methylobacterium* spp. were identified in two water samples from two different taps located at the same patient room where all patients had been. At the time of blood culturing, one patient had symptoms of pneumonia and presented with fever, neutropenia and increased C-reactive protein. The remaining patients had no symptoms, showed no fever and were not neutropenic. Ribotyping of the six isolates of *Methylobacterium* spp. revealed that isolates from two patients admitted at the same time had 92% similarity while the remaining isolates had different ribotypes with less than 80% similarity. Isolates from water were *Methylobacterium radiotolerans*, whereas isolates from the patients all were *Methylobacterium mesophilicum*.

**Conclusion:** The results of the ribotyping indicate the presence of a pseudo-outbreak with potential cross-contamination between two patients in the hospital unit not related to the water distribution system.

**Disclosure of Interest**

None Declared

### P365

#### Prevalence of respiratory viruses in immunosupressed patients in an oncological hospital in São Paulo – Brazil

Adriana M. C. E. Silva[1], Joseanne L. Lima[1], Juliana Virolli[1], Paula Vidal[1], Beatriz Quental[1], Sayonara Scota[2], Andreia Schunck[2], Ivan L. A. França e Silva[1]
[1]Serviço de Controle de Infecção Hospitalar, Hospital A.C.Camargo Cancer Center; [2]Serviço de Controle de Infecção Hospitalar, Instituto de Infectologia Emilio Ribas, são paulo, Brazil
**Correspondence:** Joseanne L. Lima
*Antimicrobial Resistance and Infection Control* 2017, **6(Suppl 3):**P365

**Introduction:** It is estimated that half of all acute respiratory infections are caused by viruses, mainly affecting immunosuppressed patients and the elderly.

**Objectives:** To verify the prevalence of respiratory virus in immunosuppressed patients of a Cancer Hospital of the State of São Paulo.

**Methods:** Retrospective study performed from January to December, 2016, at a Cancer Hospital of São Paulo. In the patients presenting respiratory symptoms, the viral panel was collected. Detection is done by the RT-PCR technique (reverse transcriptase polymerase chain reaction).

**Results:** In this period, 312 viral panel samples were analyzed, of which 48.71% (152) were female patients and 51.28% (160) male patients; 52.88% (165) had hematological tumors and 46.47% (145) solid tumors; 40.38% (126) of the patients were older than 60 years and 59.60% (186) were under 60 years of age. Prevalence was found in this study of 44.55% (139) of respiratory virus. Among them 34.53% (48) of Rhinovirus, 7.19% (10) of Influenza, 5.03% (7) Metapneumonvirus, 25.89% (36) Influenza Virus H1N1, 7.19% (10) Parainfluenza, 15.82% (22) RSV, 2.15% (3) Bocavirus, 0.71% (1) Enterovirus, 1.43% (2) Adenovirus.

**Conclusion:** The importance of diagnosis is not only due to the large variety of pathogens involved but also to the high frequency of co-infections. In addition, the correct identification of the agents can avoid the excessive use of antibiotics and antivirals in the treatment of respiratory infections and pneumonia. The diagnosis of the viral etiology of respiratory infections, besides contributing to the prevention of the transmission of respiratory viruses, can lead to a reduction in hospitalization time, the use of antibiotics and antivirals, specific isolation precautions and laboratory tests, rationalizing costs.

Disclosure of Interest

None Declared

### P366

#### Infection control in the stem cell transplant unit: a quality improvement initiative

Laxmitej Wundavalli[1], Uma Shanker Agrawal[2, 3], Sidhartha Satpathy[1], Sanjay Arya[1], Anil Kumar[4], Th Agnes Asanai[5]
[1]Hospital Administration, All India Institute of Medical Sciences, New Delhi; [2]All India Institute of Medical Sciences, New Delhi, India; [3]Infection Control and In-service Education, IRCH, All India Institute of Medical Sciences, New Delhi, -; [4]Directorate General of Health Services, Ministry of Health and Family Welfare; [5]Infection Control and In-service Education, IRCH, All India Institute of Medical Sciences, New Delhi, India
**Correspondence:** Laxmitej Wundavalli
*Antimicrobial Resistance and Infection Control* 2017, **6(Suppl 3):**P366

**Introduction:** Prevalence of Healthcare Associated Infections(HAI) varies between 5.7 and 19.1% in developing countries. Multimodal interventions have been found to be more effective than single interventions in prevention and control of HAIs. The success of any initiative is further dependent upon the participation of various stakeholders with differing perspectives, priorities and behaviours. A Quality Improvement(QI) initiative was undertaken to improve infection control practices in the stem cell transplant unit of a cancer hospital in Delhi, India.

**Objectives:** To improve the infection control practices in the stem cell transplant unit using QI techniques and tools. To increase hand hygiene compliance to 70% or above by 8 weeks. To reduce the HAI rate to 6% in one year.

**Methods:** Magnitude of the problem was assessed via surveillance through open air sampling of Mannitol Salt Agar plates and direct observation to assess hand hygiene compliance, brain storming done for root cause analyses using QI tools, 10 priority interventions were identified which were ranked based on 5 parameters in a feasibility matrix. PDCA cycles were done.

**Results:** Pre-intervention, overall hand hygiene compliance was 47.5% and it was lowest in hospital orderlies and sanitation attendants (9.09%). Post-intervention, it significantly increased to 66, 70 and 70% at 3rd, 6th and 9th week respectively. Among hospital orderlies and sanitation attendants, it rose to 69, 65 and 64 per cent in 3rd, 6th and 9th week respectively. Intervention was continued till 9 weeks and compliance to hand hygiene followed till 24 weeks. There was no significant decline in compliance of HCWs related to hand hygiene till 24th week. Pre-intervention, the average bacterial colony count and Staph aureus colony count reduced from 12.9 and 5.45 to 1.65 and 1 respectively.

**Conclusion:** QI initiatives for infection prevention and control based on interventions identified and executed through involvement of all stakeholders is an effective strategy to bring a significant reduction in risks of acquiring HAIs.

**Disclosure of Interest**

None Declared

### P367

#### Elizabethkingia miricola; an emerging pathogen in the paediatric haematology setting

Teresa Inkster[1], Padma Polubothu[2], Kathleen Harvey-wood[2], Susie Dodd[3], Linda Stewart[2], Brenda Gibson[4], Jane Turton[5], Dervla Kenna[5]
[1]Infection control; [2]Microbiology, Queen Elizabeth University Hospital, Glasgow; [3]Infection control; [4]paediatric haematology, Royal Hospital for Children, Glasgow; [5]ARHAI, Public Health England, London, United Kingdom
**Correspondence:** Teresa Inkster
*Antimicrobial Resistance and Infection Control* 2017, **6(Suppl 3):**P367

**Introduction:** *Elizabethkingia sp* are Gram negative organisms, ubiquitous in the environment. *E. meningoseptica* can cause sepsis, endocarditis and respiratory infection. *E. miricola* is rare, first identified in condensation from the space station Mir (2003). The first clinical isolate of *E. miricola* was identified in 2008 from an adult with lymphoma. MALDI-TOF is unreliable in distinguishing Elizabethkingia species.This may be the reason for the paucity of reported infections with *E. miricola*. The only method currently available to correctly identify *Elizabethkingia* subspecies is rpoB sequence cluster analysis.

**Objectives:** We describe 3 cases of *E.Miricola* bacteraemia and the ensuing investigations.

**Methods:** 3 paediatric haematology patients were positive for *E. meningoseptica* from blood cultures in a 6 month period.Isolates were sent to the ARHAI Reference Unit and identified as *E. miricola*.

**Results:** Isolates were identified by the reference unit as *E.miricola* using rpoB sequence cluster analysis. Typing revealed three unique strains of the organism. The three isolates were ESBL producers and meropenem resistant due to the production of a metallo-β-lactamase. All three patients required treatment of *E. miricola* bacteraemia. Infection control investigation revealed no commonalities between patients other than having been an inpatient on the same ward at different times. Water samples from outlets and chilled beams on the ward tested negative for *Elizabethkingia sp*.

**Conclusion:** Following its identification in 2003 clinical isolates and case reports of *E. miricola* have been rare. It was unusual for us to encounter three bacteraemias over a six month period. Typing revealed three different strains making a point source unlikely. Water testing of outlets and chilled beams were all negative. Patients required treatment and the isolates were multi-resistant meaning treatment options were limited. Our isolates were misidentified initially as *E. meningoseptica*. Diagnostic labs should consider sending *E.meningoseptica* to reference units to identify rarer species such as *E. miricola*.

**Disclosure of Interest**

None Declared

### P368

#### Bacteraemia following faecal microbiota transplantation for recurrent clostridium difficile infection in an immunosuppressed patient

Mingjuan Tan[1], Nares Smitasin[2], David Ong[3,4], Alison J. Lee[5], Roland Jureen[6], Paul Tambyah[2,4], Nicholas Chew[2,4]
[1]University Medicine Cluster; [2]Division of Infectious Diseases, National University Hospital; [3]National University of Singapore (NUS) Yong Loo Lin School of Medicine; [4]Division of Gastroenterology & Hepatology; [5]Department of Paediatrics; [6]Department of Laboratory Medicine, National University Hospital, Singapore, Singapore
**Correspondence:** Mingjuan Tan
*Antimicrobial Resistance and Infection Control* 2017, **6(Suppl 3):**P368

**Introduction:** Faecal microbiota transplantation (FMT) is a widely used treatment option in refractory and recurrent *Clostridum difficile* infection (CDI). The experience in immunosuppressed patients has been largely safe and effective.

**Objectives:** To highlight a potential complication of FMT in immunosuppressed hosts.

**Methods:** We report a case of a young immunosuppressed woman who developed polymicrobial bacteraemia after FMT for recurrent CDI.

**Results:** The patient was a 25 year old woman with a primary immunodeficiency syndrome of unclear aetiology, multiple hypersensitivities and autoimmune polyglandular syndrome: type 1 diabetes mellitus, adrenal insufficiency and hypothyroidism. She was on immunoglobulin replacement as well as insulin, prednisolone and levothyroxine. Previous infections included *Mycobacterium fortuitum* osteomyelitis, multiple bacterial and fungal wound infections, acute cholecystitis, a chronic rectal ulcer requiring partial resection, and catheter-related bloodstream infections. Due to prior treatment with broad-spectrum antibiotics, she developed recurrent CDI despite multiple courses of oral metronidazole and vancomycin. She underwent FMT via colonoscopy. Six hours later, she developed septic shock with a 40 °C fever, tachycardia and hypotension. Blood cultures grew *Enterococcus faecalis, Escherichia coli* and *Klebsiella pneumoniae*. She was treated with intravenous antibiotics. She recovered well enough to be discharged, but soon returned with fever. Unfortunately, she continued to have CDI, and eventually underwent total colectomy with end ileostomy formation.

**Conclusion:** While FMT is an effective treatment for recurrent CDI, it should be carefully considered in immunosuppressed patients, in whom we do not understand the interaction between the faecal microbiome and host immune system.

**Disclosure of Interest**

None Declared

### P369

#### Multiresresent microrganisms isolated in surveillance swab in hiv / aids patients at a hospital de infectologia de São Paulo - Brazil

Andreia C. B. P. D. Schunck, Adriana M. Costa e Silva, Nilton J. F. Cavalcante, Sayonara Scota, Regia D. F. Feijo
Serviço de Controle de Infecção Hospitalar, INSTITUTO DE INFECTOLOGIA EMILIO RIBAS, São Paulo, Brazil
**Correspondence:** Andreia C. B. P. D. Schunck
*Antimicrobial Resistance and Infection Control* 2017, **6(Suppl 3):**P369

**Introduction:** Infections caused by multiresistant microorganisms are associated with prolonged hospital stays, high costs, and increased morbidity and mortality.

**Objectives:** To verify the incidence of multiresistant bacteria (Enterococcus spp resistant to vancomycin, Acinetobacter baumannii and Klebsiella pneumoniae producing carbapenemase) in surveillance swabs in HIV/AIDS patients of a Hospital of Infectology of the State of São Paulo.

**Methods:** Retrospective study conducted from October 2016 to February 2017, in a Hospital of infectious diseases in the State of São Paulo. The surveillance swab was collected weekly from all patients admitted to the Intensive Care Unit.

**Results:** In this period, 130 surveillance swab cultures collected from HIV/AIDS patients, with an incidence of 24.6% (32) were analyzed for multiresistant microorganisms. Sixty percent (2 cases) of Acinetobacter baumannii, 60.6% (20 cases) of Enterococcus spp resistant to vancomycin (VRE) and 33.3% (11 cases) of Klebsiella pneumoniae producing carbapenemase (KPC) were isolated. This study shows the importance of identifying these microorganisms for preventive measures to be implemented: colonized patients are placed in contact precautions, the hygienization of the environment is performed with 0.5% sodium hypochlorite, hygienization of the hands with chlorhexidine degermant and is Performed an audit by the Hospital Infection Control Service.

**Conclusion:** Several factors are related to the persistence of these microorganisms within hospital units, such as individual vulnerability of patients, prolonged use of antimicrobials generating selective pressure, cross-transmission by colonized and/or infected patients, and the impact of adherence to prevention and control measures of infections. The simple act of sanitizing hands before and after patient care can prevent these transmissions.

Disclosure of Interest

None Declared

### P370

#### Incidence of healthcare-associated infections in hiv / aids patients at the intensive therapy unit of an institute of infectology of São Paulo - Brazil

Andreia C. B. P. D. Schunck, Adriana M. Costa e Silva, Sayonara Scota, Regia D. F. Feijó, Rosana Richtmann, Nilton J. F. Cavalcante
Serviço de Controle de Infecção Hospitalar, INSTITUTO DE INFECTOLOGIA EMILIO RIBAS, São Paulo, Brazil
**Correspondence:** Andreia C. B. P. D. Schunck
*Antimicrobial Resistance and Infection Control* 2017, **6(Suppl 3):**P370

**Introduction:** Healthcare-associated Infections (HAI) represents one of the main problems in the quality of health care due to an increase in hospitalization time, morbidity and mortality, as well as disorders caused by pain, malaise, isolation, and finally by patient suffering. Patients with HIV/AIDS are potentially a population at higher risk for acquisition of HAI when compared to other groups of hospitalized patients.

**Objectives:** To verify the incidence density of the infections related to health care in a Hospital of Infectious Diseases of the State of São Paulo.

**Methods:** Descriptive, retrospective and quantitative study, whose objective was to analyze the occurrence of hospital infections in patients of the Intensive Care Unit from January to December, 2016, in a reference Hospital in Infectology of the State of São Paulo.

**Results:** 90 infections related to health care were verified between January and December 2016 in patients with HIV/AIDS. Of these infections, the incidence density ratio was 13.6 (41) of primary laboratory bloodstream infections; 19.7 (28) of pneumonia associated with mechanical ventilation; 2.0 (6) for pneumonia, 2.9 (7) for infection related to central vascular access and 1.7 (5) for urinary tract infection. The highest incidence density of HAI observed in the Intensive Care Unit was pneumonia associated with mechanical ventilation, corroborating with the study by Romcy YM (2009).

**Conclusion:** The importance of approach and application of the Bundle of Prevention of Pneumonia Associated with Mechanical Ventilation, besides the early withdrawal of invasive devices that act as important risk factors and are determinant in the occurrence of infections.

CDC. National Healthcare Safety Network (NHSN) Patient Safety Component Manual, 2017.

Disclosure of Interest

None Declared

### P371

#### Care-seeking behaviors among hiv-infected adults in Mozambique: barriers and facilitators to timely enrollment in HIV care and treatment

Amilcar J. Magaco[1], Reka Cane[2], Carlos Botao[1], Francisco Mbofana[1]
[1]NATIONAL INSTITUTE OF HEALTH, Maputo, Mozambique;
[2]Malhangalene B, 53, NATIONAL INSTITUTE OF HEALTH, Maputo, Mozambique
**Correspondence:** Amilcar J. Magaco
*Antimicrobial Resistance and Infection Control* 2017, **6(Suppl 3):**P371

**Introduction:** The Ministry of Health of Mozambique pledged to eliminate vertical transmission, reduce sexual transmission by 50% and increase antiretroviral therapy coverage up to 80%. To achieve these goals, it is essential that HIV-infected persons must access HIV care services in a timely manner. However, HIV-infected persons in Mozambique are continuously accessing care and treatment services at an advanced stage of the disease.

**Objectives:** To explore reasons why patients with positive diagnosis do not adhere to services and care of HIV, regardless of the disease stage.

**Methods:** In-depth interviews were conducted to 90 newly diagnosed HIV-positive patients in one health facility in Maputo City and two urban and rural health facilities in the province of Zambézia. Interviews were recorded, transcribed and translated prior to analysis by NVivo.

**Results:** According with patients, issues such as long distances to the health facility, long waiting time, lack of access to transport, lack of money, stigma and side-effects were the most frequently barriers to timely enrollment in HIV care and treatment after testing. Most of the participants living in Zambezia stated that they had difficulties of accessing to the clinic because of the distance. One of the facilitators for the timely enrollment of patients in the care and treatment of HIV is related to the fact that enrollment's process in HIV care happens the next day after the test completion. Family support associated with the basic knowledge related to HIV was mentioned as one of the main factors that led to an early onset of ART therapy. While some patients reported that the sensation of feeling good while doing the medication is one of the factors that mostalso contributes to ART therapy adherence, others showed to feel motivated for the treatment due to the encouragement given by their social networking.

**Conclusion:** Adherence to treatment is influenced by the desire of feeling healthy. Our results sugest that one of the major facilitators for a timely enrollment of patients in care services and treatment of HIV is the flexibilate process of enrollment in HIV care at health facilities. The stigma and side-effects were the most frequently barriers to timely enrollment in HIV care and treatment.

**Disclosure of Interest**

None Declared

## Challenges with endoscopes

### P372

#### Protein and atp concentrations found in clinical soil from flexible endoscopes are correlated

Henning Reuter[1], Maren David[2], Marco Bommarito[2]
[1]3M Germany, Neuss, Germany; [2]3M Healthcare, St Paul, United States
**Correspondence:** Henning Reuter
*Antimicrobial Resistance and Infection Control* 2017, **6(Suppl 3):**P372

**Introduction:** Deadly outbreaks of CRE and other MDROs, have been associated with flexible endoscopes, highlighting the importance of effective cleaning and disinfection.

**Objectives:** The objective of this study is to quantify how protein and adenosine tri-phosphate (ATP) concentrations vary in clinical soil removed from patient-used endoscopes as the device is cleaned and disinfected.

**Methods:** The study measured levels of protein and ATP in 89 endoscopes (48 colonoscopes, 38 gastroscopes, 3 duodenoscopes) at two US clinical sites for three points during reprocessing: bedside cleaning, post-manual cleaning, and after high level disinfection (HLD). ATP concentrations in Relative Light Units (RLUs) were determined using a luminometer. Protein concentrations in mg/mL were determined using a bicinchoninic acid (BCA) assay. A non-linear least squares regression was used to determine the relationship between the two markers.

**Results:** A Log Normal function was found to provide the best fit to the data (solid red line, $R^2 = 0.72$; 95% confidence intervals shown as dotted red lines). The analysis shows that ATP and protein levels are linearly correlated (dashed black line, $R^2 = 0.68$) for protein concentrations greater than 8 mg/ml and ATP values greater than 20 RLUs (corresponding to an ATP concentration of ~7 fmoles/ml). Also shown on Deviation from linearity of the ATP-protein correlation occurs well below the intersection point of cleanliness criterions found in ANSI/AAMI ST-91:2015. A BCA assay was chosen because it has a limit of detection < 1 mg/ml.

**Conclusion:** This study found that the concentration of ATP and protein found in clinical soil collected from patient used endoscopes, are linearly correlated to levels well-below those defined by currently accepted cleanliness criterions.

**Disclosure of Interest**

H. Reuter Employee of: 3 M Germany, M. David Employee of: 3 M Healthcare, St Paul, M. Bommarito Employee of: 3 M Healthcare, St Paul

### P373

#### Characterization of the effectiveness of clinical soil removal from endoscopes during cleaning and disinfection

Henning Reuter[1], Marco Bommarito[2], Sabine Heiligtag[3]
[1]3M Germany, Neuss, Germany; [2]3M Healtcare, St Paul, St Paul, United States; [3]3M Germany, Neuss, Germany
**Correspondence:** Henning Reuter
*Antimicrobial Resistance and Infection Control* 2017, **6(Suppl 3):**P373

**Introduction:** Several recent deadly outbreaks of CRE and other MDROs, have been associated with flexible endoscopes, highlighting the importance of effective cleaning and disinfection.

**Objectives:** The objective of this study is to quantify how clinical soil is removed from patient-used endoscopes as the device progresses through cleaning and disinfection.

**Methods:** The study measured levels of adenosine tri-phosphate (ATP) and protein in 118 endoscopes (53 colonoscopes, 59 gastroscopes, 6 duodenoscopes) at four clinical sites (2 in the US and 2 in Germany) for three points during reprocessing: bedside cleaning, post-manual cleaning, and after high level disinfection (HLD). ATP concentrations in Relative Light Units (RLUs) were determined using a luminometer. Protein concentrations in µg/mL were determined using a bicinchoninic acid (BCA) assay. A general linear model (GLM) employing three factors (clinical site, endoscope type and reprocessing step) was used for the statistical analysis. P-values < 0.01 indicated significance.

**Results:** GLM analysis the ATP data showed that the only significant factors were the endoscope type and the reprocessing step, whereas for the protein data, only reprocessing step was a significant factor. Importantly, clinical site was not a significant factor for either marker. Mean values of ATP and protein are summarized by workflow step and endoscope type respectively. Also shown are the differences in the means by each factor. A Tukey pairwise comparisons identified significant differences in the means.
**Conclusion:** This study found that levels of clinical soil, characterized by the concentrations of ATP and protein, decrease as the endoscope is decontaminated, independently of clinical site. The study also found that contamination levels in gastroscopes and duodenoscopes were significantly higher than in colonoscopes at each step of the process. These findings imply that the process used to clean and disinfect these devices can only remove a fixed amount of contamination at each step, and may not be capable of properly decontaminating endoscopes with excessive initial amounts of clinical soil.
**Disclosure of Interest**
H. Reuter Employee of: 3 M Germany, M. Bommarito Employee of: 3 M Healthcare, St Paul, USA, S. Heiligtag Employee of: 3 M Germany

### P374
### Columbu's egg for endoscopes reprocessing traceability?
Bispo S. Ana[1], Carlos Palos[1], Filipa Pires[2]
[1]Infection Control Comittee; [2]special exams and ambulatory surgery, Hospital Beatriz Ângelo, Loures, Portugal
**Correspondence:** Bispo S. Ana
*Antimicrobial Resistance and Infection Control* 2017, **6(Suppl 3):**P374

**Introduction:** Hospital Beatriz Ângelo(HBA) is a JCI-accredited, 425-bed general hospital. Proving adequacy and conformity to endoscope reprocessing guidelines has quality, safety and legal implications.In HBA, endoscopies are performed at a special unit, comprising 2 Automatic Washer-Disinfector(AWD). 23 scopes are in use, with a mean daily number of 32 exams(8793 last year).The will of starting a traceability system lead to design an easy manual system, allowing the knowledge of sequence of all patients submitted to exam with each scope.
**Objectives:** Implementing a traceability system to correlate scopes and patients.
**Methods:** Our traceability system is a manual registry that only uses AWD validation ticket and patient's identification tags.Tags and tickets are organized in a specific order, one folder per scope.We start by introducing the previous patient ID number(patient A) in the subsequent AWD cycle, so that this information ends up in AWD validation ticket of that cycle.In the first raw of scope's folder, this ticket is stamped on the left. Next patient submitted to exam with that scope identification's tag(patient B)is stamped on the right, and so on. In doing so, we are able to know:1)who underwent previous examination(patient A); 2) if the reprocessing process was valid;and 3)who was the patient that went to next examination(patient B).In the row beneath we repeat this scheme, starting with patient B post AWD validation ticket to the left and next patient submitted to exam identification tag(patient C)to the right.
**Results:** Currently, traceability is possible for all exams performed in each scope since implementation.If any problem occurs we are able to identify every patient that underwent previous and afterwards endoscopic procedure with a specific scope.We do this without access to any software or data processing module, but only by organizing data with specific scheme, using solely AWD validation cycle ticket.
**Conclusion:** Traceability of reprocessing processes is a gold standard for critical devices.Semi-critical devices however lack this information support, which is critical in the recent outbreaks associated to scope design.Our data organization allows any unit with standard AWD machines to have a traceability system and to proof adequate reprocessing procedure prior to any exam in any point of time, with no need of additional IT requirements.
**Disclosure of Interest**
None Declared

### P375
### Why audit endoscopic reprocessing processes?
Fernanda Vieira[1], Ilda Devesa[1], Alexandra Fernandes[1], David Peres[1], Isabel Neves[1,2]
[1]Infection Control and Antimicrobial Resistance Unit; [2]Infectious Diseases Unit, Matosinhos Local Health Unit, Matosinhos, Portugal
**Correspondence:** Fernanda Vieira
*Antimicrobial Resistance and Infection Control* 2017, **6(Suppl 3):**P375

**Introduction:** The endoscopic technology is increasingly complex, devices reprocessing requires detailed description of the procedure and a close monitoring. We consider that current guidelines are not objective enough, and it is necessary to effectiveness monitor the washing, the most basic procedure. All medical devices should be thoroughly rinsed prior to any disinfection or sterilization process. The presence of organic matter inhibits contact with the disinfection/sterilization agent and may interfere with the high disinfection effectiveness.Quality control of the all phases involved cleaning endoscopes has been associated with patient infections reduction.
**Objectives:** To enhance the importance of audit endoscopy cleaning.
**Methods:** 1. Cleaning process audit in two times, initial and after institution of corrective measures, using biochemical methods with ATP technology (RLU). Such detect dirt and infer quality of cleaning, but are not reliable for detect microbial contamination.All hospital endoscopes were audited: mechanical automatic lavage (gastroenterology and pneumology) and manual lavage (urology, gynecology and otorhinology).2. Sharing of initial audit results to the operational team and discussion of corrective measures which should be implemented. Subsequently, new audit was made to infer the improvements.
**Results:** The endoscopes subject to mechanical automatic lavage were clean; in manual lavage we detected two nonconformity: one related with an cystoscopy and one related with a endovaginal probe. In the second audit, the results were very positive, in the cystoscopy we've found a reduction from 6791 to 31 RLU; in the endovaginal probe reduction from 298 RLU to 128 RLU.
**Conclusion:** Periodic auditing for the cleaning procedure is essential as an indicator of quality control. Audits of reprocessing practices promote identification of failures and consequent opportunity for continuous quality improvement. Incorrect reprocessing steps, such as pre-cleaning and cleaning, may cause a wrong disinfection. A visual assessment is not enough. This audit using ATP technology was useful to monitor and control the process of cleaning medical devices, identifying problems, promoting improvements and patient safety.
**Disclosure of Interest**
None Declared

### P376
### A microbiologic surveillance study of reprocessed endoscopes at MNGHA
Ahmad Ibrahim, MAJED SHOMRANI, THAMER ENAZI, Mishal Johani, MOHAMAD NOR FAYZAH, Awaji Ahmed, Winger Dawn, Dagunton Nimfa, BACHU SHAKILLA, Gysman Laura, Balkhy Hanan
INFECTION PREVENTION & CONTROL, MNGHA, RIyadh, Saudi Arabia
**Correspondence:** Ahmad Ibrahim
*Antimicrobial Resistance and Infection Control* 2017, **6(Suppl 3):**P376

**Introduction:** Gastrointestinal (GI) endoscopy is an important tool for the diagnosis and management of GI tract disorders. GI endoscopy is performed in body cavities heavily colonized with microorganisms. The reprocessing of the endoscope to allow for its use in multiple patients involves multiple steps: pre-cleaning, cleaning, disinfection, rinsing, drying, and then storage. There are effective protocols for the reprocessing of endoscopes. However the accepted time interval (hang time) for the storage of reprocessed endoscope is not well defined. There is a strong paucity of research in this area.
At the MNGHA, the practice has been reprocessing every 24 hours regardless of whether the endoscope has been used or not.
We decided to conduct a microbiological surveillance on selected endoscopes to evaluate any potential growth of microorganisms.

**Objectives:**
To assess if there is an association between the duration of hanging reprocessed endoscopes and contamination, the results finding will yield in changing the hang time storage of reprocessed endoscopes.
**Methods**
Four reprocessed endoscopes were selected to undergo microbiological surveillance testing at 48 hours after reprocessing and 72 hours after. Swabs were obtained from the Tip, Knob and Lumen fluid from each endoscope. A total of 240 culture samples were obtained for aperiod of 66 days.
**Results**
Of the 240 samples 5 colonies were detected. Comparison of the two groups (48 hrs & 72 hrs) showed the following : Growth of coagulase negative *Staphylococcus aureus* in two colonies at the tip at 48 hours but none at 72 hours. Growth of *Bacillus* species from the lumen fluid at 48 hours but none at 72 hours and *Corynebacterium* from lumen fluid in two colonies at 72 hours. These 3 pathogens are classified under "low- concern bacteria" (normal commensals of skin and environment)
**Conclusion**
No growth of pathogenic bacteria was observed, concluding that any potential contamination identified during early storage could not be found.We therefore recommend that the hanging time of reprocessed endoscopes be 72 hours until the appropriate storage cabinet becomes available. In the absence of evidence-based guidelines for unresolved issues, every effort should be made in a timely and efficacious manner to ensure patient safety.
**Disclosure of Interest**
None Declared

### Occupational health

#### P377
**Withdrawn**

#### P378
**Seroconversion of occupational exposure to blood and body fluid among healthcare personnel over 6 years at a teaching hospital in Korea**

Eunjin Ha[1], Dongsuk Lee[1], Juhyeun Lee[1], Ohmee Kweon[1], Eun Suk Park[1], Jun Yong Choi[1,2]
[1]Department of infection control; [2]Department of Internal Medicine, Severance hospital, Seoul, Korea, Republic Of
**Correspondence:** Eunjin Ha
*Antimicrobial Resistance and Infection Control* 2017, **6(Suppl 3):**P378

**Introduction:** The risks associated with a blood and body fluid exposure are serious in healthcare personnel(HCP).
**Objectives:** The aim of this study is to verify the seroconversion rate of occupational exposures.
**Methods:** An observational prospective study was done at a 2,322-bed teaching hospital in Korea from January 2010 to December 2015. The prevention approaches including education, use of personal protective equipment and safety-engineered devices has been maintained through this period and the injury rate per 100 occupied beds per year did not changed significantly(average of 21.4). At the time of self-reporting of exposure, blood sample of HCP and the source was collected for baseline HBV, HCV, and HIV serum markers. The HCP exposed, susceptible to HBV or exposed to HIV was administered postexposure prophylaxis(PEP) according to CDC recommendations. Follow-up laboratory testing was obtained on HCP exposed to HBV, HCV and HIV up to maximum 6 months.
**Results:** A total of 2,975 injuries were reported. Percutaneous injury was the most common(88.9%), mucous membrane exposure was following(10.4%). Serologic test results of the source identified were obtained in 2,180 cases(388 HBV-positive, 188 HCV-positive, 38 HIV-positive and 11 multiple seropositive). Sixty eight among exposed HCP to HBV had Anti-HBs titers <10 mIU/mL (17.6%), 7 of them had already vaccinated. No HBV seroconversion case has been detected. No HIV seroconversion after exposure to HIV-infected source was found. Among exposed HCP to HCV, 172 were finished follow-up laboratory testing. Two cases had positive results for HCV-PCR conducted at 4 weeks after exposure and seroconversion rate was 1.2%. One was associated to disposal of a suture needle, the other was to remove a hollow-bore phlebotomy needle.
**Conclusion:** Since most HCP is immune to HBV as the result of pre-exposure vaccination and PEP is used for exposures to sources known to be HIV-infected, no seroconversion of HBV and HIV was seen. HCV seroconversion rate is similar to other studies. Vaccination, prevention of percutaneous injuries and other blood exposures and prompt and appropriate PEP intervention are important in preventing the transmission of bloodborne viruses to HCP.
**Disclosure of Interest**
None Declared

#### P379
**Surgeon's knowledge, attitudes and practices related to blood exposure in the operating rooms of a Tunisian Tertiary Care Hospital**

Nawel Zammit[1], Mohamed Mahjoub[1], Rym Bouriga[2], Nabiha Bouafia[1], Asma Ammar[1], Hela Ghali[1], Olfa Ezzi[1], Asma Ben Cheikh[1], Wadiaa Bannour[1], Radhia hellali[1], Mansour Njah[1]
[1]Hospital Hygiene; [2]medical oncology, university hospital Farhat Hached Sousse Tunisia, Jawhara, Tunisia
**Correspondence:** Mohamed Mahjoub
*Antimicrobial Resistance and Infection Control* 2017, **6(Suppl 3):**P379

**Introduction:** Accidental blood exposure is frequent among surgeons and may cause severe consequences such HIV and hepatitis B and C infections.
**Objectives:** To determine the Surgeon's knowledge, attitudes and practices related to accidental blood exposure in the operating rooms of a Tunisian tertiary care hospital.
**Methods:** Cross sectional study was conducted in the four operating rooms of a tertiary care Tunisian hospital between July and December 2016. All surgeons of the hospital were included. A self-administrated questionnaire was used to collect data about knowledge, attitudes and practices related to accidental blood exposure.
**Results:** The prevalence of blood exposure was 79.7%. The higher prevalence was reported in Gynaecology (100%) followed by General surgery (91.7) then Ophthalmology (75%) and finally Oto-rhino-laryngology (71.4%) (p = 0.047).
Gynaecologists were the most surgeons who underestimated the protective role of double gloving during interventions (29.6%) compared to the surgeons from the General surgery (16.6%), Ophthalmology (16.2%) and Oto-rhino-laryngology (0%). While this difference was not statistically significant (p = 0.27). Compared to the other surgeons, Gynaecologists were the surgeons who request serology for themselves after blood exposure the less frequently: 50% versus 75% in both: General surgery and Oto-rhino-laryngology and 92% in Ophthalmology (p = 0.05). Moreover, Gynaecologists were the surgeons who registered blood exposure accidents the less frequently (24%), compared to Ophthalmologists (73%), General surgeons (25%) and Oto-rhino-laryngologists (25%) (p = 0.01).
**Conclusion:** The prevalence of accidental blood exposure in the Tunisian operating rooms is high, especially in Gynaecology. Interventions to improve the surgeon's attitudes and practices related to blood exposure prevention are required.
**Disclosure of Interest**
None Declared

#### P380
**Blood exposure accidents in surgical departments: a survey among physicians in a university-hospital**

Mohamed Mahjoub[1], Nawel Zammit[1], Rym Bouriga[2], Nabiha Bouafia[1], Asma Ammar[1], Hela Ghali[1], Asma < H3 > Ben Cheikh[1], Wadiaa Bannour[1], Radhia hellali[1], Mansour Njah[1]
[1]Hospital Hygiene; [2]medical oncology, university hospital Farhat Hached Sousse Tunisia, Jawhara, Tunisia
**Correspondence:** Mohamed Mahjoub
*Antimicrobial Resistance and Infection Control* 2017, **6(Suppl 3):**P380

**Introduction:** Some specialties are deemed to be at increased risk of blood exposure accidents (BEA) at hospital. Surgeons are known as the most exposed caregivers to the risk of BEA.
**Objectives:** Our objective was to clarify the prevalence of BEA among surgeons and to describe their perceptions and practical attitudes following the occurrence of a BEA.
**Methods:** A descriptive cross-sectional study was conducted in 2016 including all surgeons, holders or in training (residents), in surgical services of our University-Hospital that were present during the period of the study. Collection of data was made by a self-administered pre-established and pre-tested questionnaire. Seizure and analysis of the data were made by the SPSS software v20.0
**Results:** Overall response rate was 83.1% (64/77). History of BEA was reported by 79.7% of the participants. BEA's reported average number was 3. The BEA had occurred by stitch in 75% of cases. The BEA most provider specialties were: gynecology (95%), general surgery (91.7%), ophthalmology (75%) and finally the otorhinolaryngology (71.4%) (p = 0.047).
Following a BEA, 25% of the victims report that they have not conducted the first care (especially no application of an antiseptic), 9.8% have consulted to emergencies, 29.4% have stated and have benefited from viral serologies and 15.7% have had the agreement of the source patients for their practice of serologies.
**Conclusion:** Better mastery of the infectious risk linked to the BEA among surgeons remains a health issue. It is essential to opt for the strengthening of the training and information about universal measures of BEA prevention; and to opt for dissemination of safety equipment.
Implementation of BEA structured prevention strategy, according to the updated and clearly defined modalities, is essential in order to better manage risks of infection and to improve quality and safety of surgical practices.
**Disclosure of Interest**
None Declared

### P381
**Withdrawn**

### P382
### The effect of multimodal intervention for the prevention of needlestick injury on the improvement of safety behaviors and incidence of needlestick injury among nurses

Eunkyung Lee[1,2], Hye-Ran Choi[3], Jae Sim Jeong[4]
[1]Department of Clinical Nursing, University of Ulsan Graduate School of Industrial Technology; [2]Department of Infection Control, Konkuk University Medical Center; [3]Department of Clinical Nursing, University of Ulsan College of Medicine; [4]Department of Nursing, University of Ulsan College of Medicine, Seoul, Korea, Republic Of
**Correspondence:** Eunkyung Lee
*Antimicrobial Resistance and Infection Control* 2017, **6(Suppl 3):**P382

**Introduction:** Needlestick injuries are most prevalent among nurses who frequently handle a syringes or needles.
**Objectives:** The study was carried out with the aim of improving the safety behaviors and reducing the incidence of needlestick injurie through various interventions.
**Methods:** Before and after experiment design was adopted. A total of 29 nurses in the internal medicine department of a university hospital in Seoul, Korea were conveniently sampled. The interventions included the use of needle transfer trays, lecture education, needle safety behavior video viewing, and blood sampling practice using safety devices. The needle safety behavior scores were collected by direct observation and self-assessment questionnaires. The incidence of needlestick injuries was collected through the questionnaire and reported to the infection control office.
**Results:** The total number of injuries before intervention was 26 out of 29 subjects and decreased to 13 in post intervention surveys. The proportion of nurses who experienced needlestick injuries were significantly reduced from 89.7% before the intervention to 44.8% after the intervention ($p = .033$). In the self-assessment score for safety behaviors, the total score before the intervention was 38.31 ± 3.02, and the total score after the intervention was 39.97 ± 4.18 points ($p = .026$). The observation score of the needle safety behaviors was 6.24 ± 0.82 points at 10 points before intervention and 7.75 ± 1.24 points after intervention ($p < .001$). In addition, it increased from 5.13 ± 0.94 to 7.14 ± 0.89 ($p < .001$) for venous sampling, and from 6.35 ± 0.99 to 7.61 ± 0.8 ($p < .001$) for intravenous injection, and the increases were all statistically significant.
**Conclusion:** Multimodal interventions were found to be very effective in promoting the incidence and safety behaviors of the needlestick injuries. However, there is no difference in some safety behaviors, so it is necessary to identify the cause.
**Disclosure of Interest**
None Declared

### P383
### Practices of injections safety: a clinical audit at al-shifaa hospital in the Gaza-Strip

Mohamedraed S. Elshami, Enas F. Alaloul, Jomana K. Hashem, Bettina Bottcher
Faculty of Medicine, Islamic University, Gaza, Palestinian Territory, Occupied
**Correspondence:** Mohamedraed S. Elshami
*Antimicrobial Resistance and Infection Control* 2017, **6(Suppl 3):**P383

**Introduction:** A safe injection is one that does not harm the recipient, does not expose the provider to any avoidable risks and does not result in waste that is dangerous for the community. This is the first clinical audit in the Gaza-Strip to observe the application of different protective practices while giving injections to patients.
**Objectives:** To evaluate adherence of healthcare professionals of injections safety measures.
**Methods:** This was a prospective clinical audit conducted in the emergency room at Al-Shifaa Hospital from January to March 2017 according to WHO guidelines. The data was analyzed using SPSS program.
**Results:** In total, 100 cases were observed, of which 40% were for venous access. Among the performers of injections, 50% were females. Sixty percent were nurses, 20% were medical students and 20% were nursing students. The overall compliance rate for all practices was 20%. It was noted that the highest compliance rate was among nursing students (p = 0.2) and a higher rate was obtained among males than females (p = 0.3). Interestingly, the highest rate was observed for those in the 20 to 30 year age group (p = 0.4). The hands of the performer were visibly clean in all observations. Only 20% washed their hands just after patient contact. Although wearing non-sterile gloves is recommended for venous access, this was only done in 5%. Disinfecting skin with 60–70% alcohol-based solution was performed in all cases. However, inappropriate technique, touching the puncture site and no waiting for at least 30 seconds after disinfection were observed in 60%, 20% and 90% respectively. The needle and syringe were immediately discarded into a robust sharp container in 80% but recapping the needle using one-hand scoop technique was practiced in none of them while injecting blood into a laboratory tube while holding it with the other hand was done in 80%, which is not recommended, as it poses a risk for needle stick injury.
**Conclusion:** Infection control measures concerning the administration of injections is shockingly poor among all healthcare professionals, including those still in training, who should show higher guideline adherence. As this is a serious shortfall in patient safety practices, it requires urgent address in form of implementation and training in infection control practices among healthcare staff.
**Disclosure of Interest**
None Declared

### P384

#### Factors influencing take-up rate of influenza vaccine amongst healthcare workers in a cancer centre in Singapore

Ming Zhen Priscilla Han[1], Whee Sze Ong[2], Sin Hui Wong[1]
[1]Infection Control Unit; [2]Division of Clinical Trials and Epidemiological Sciences, National Cancer Centre Singapore, Singapore, Singapore
**Correspondence:** Ming Zhen Priscilla Han
*Antimicrobial Resistance and Infection Control* 2017, **6(Suppl 3):**P384

**Introduction:** In Singapore, all healthcare workers who come in contact with patients and patient specimens are recommended for annual seasonal influenza vaccination. Although the seasonal influenza vaccination is free and offered at our Ambulatory Cancer Centre, the uptake among HCW has seen a gradual decline from 41% in 2007 to 28% in 2015.

**Objectives:** The objectives of this study were to examine motivators and barriers influencing healthcare worker's compliance with influenza vaccination; and identify modifiable factors that can improve vaccine take-up rate.

**Methods:** A cross-sectional anonymous survey was distributed to 906 healthcare workers (HCW) at Cancer Centre with a response rate of 56% in 2016. The survey consisted of 3 sections; demographic data, vaccination history and Health Belief Model Categories Scale.

**Results:** A total of 205 HCW (41%) were vaccinated in 2015. About half of HCW had ≥ 5 years working experience, and 74% had patient contact. HCW who received seasonal influenza vaccines in the past 5 years ($p < 0.001$) and those with patient contact ($p = 0.05$) were more likely to be vaccinated. Nineteen of the 21 HBM factors were also associated with influenza vaccine uptake ($p < 0.05$). On multivariate analysis, independent factors associated with vaccination uptake were (1) history of receiving influenza vaccine in the past 5 years (OR = 28.75; 95% CI, 12.01–68.83); (2) perceived protection after the influenza vaccination (OR = 2.77; 1.64-4.69); (3) concur the use of talks/ poster as reminder for influenza vaccination (OR = 2.01; 1.18-3.43); and (4) concerns about the serious adverse reactions after receiving influenza vaccine (OR = 0.41; 0.22-0.76).

**Conclusion:** This study allowed an in-depth insight into the fundamental reasons of healthcare workers' reluctance to receive influenza vaccination. With the identification of factors influencing vaccination, tailoring promotion programmes targeted at addressing HWC's concerns; giving positive motivations thereby leading to increase uptake of vaccinations is invaluable.

**Disclosure of Interest**

None Declared

### P385

#### Infection control: health care workers screening at selected facilities

Francina M. Tjtiuka
Quality Assurance, Ministry of Health, Windhoek, Namibia
*Antimicrobial Resistance and Infection Control* 2017, **6(Suppl 3):**P385

**Introduction:**

The national Infection Control guideline recommends a package for preventing TB transmission amongst Health care workers (HCWs) The burden of TB disease among HCWs is unknown despite the high prevalence in the region.

**Objectives:** To assess the TB burden among HCW in the region

**Methods:** Namibia notified 10,610 cases of all forms of TB in 2013. Ohangwena region accounted for 10% of these cases and 15% of the country's Drug Resistant TB cases.

Sensitization meetings were held with the Nurse Managers and all HCWs, and symptom screening was done for all HCWs (747) with a standardized questionnaire. Symptomatic staff submitted sputum samples, which were tested using Gene Xpert.

**Results:** Out of 747 HCW screened, 316 (42%) had at least one symptom of TB. Of these, 291 (92%) submitted sputum for further laboratory investigation with Gene Xpert. M.tb was detected in 20 (7%) of the samples; none of these had rifampicin resistance. Nurses constituted 8 (40%) of the confirmed cases

**Conclusion:** There is a high incidence of TB among HCWs in the region, possibly due to occupational exposure. Infection Prevention and Control (IPC) measures need to be strengthen including airborne infection precautions. Nurse Manger's need to ensure that facilities conduct risk assessment and develop facility Infection Control plans. There is a need to strengthen routine TB screening of HCWs and evaluate role of occupational exposure in TB transmission.

**Disclosure of Interest**

None Declared

### P386

#### Compliance with who tb laboratory biosafety measures in public health laboratories in Kisumu County, Kenya, 2015

Barbara K. Burmen, Tobias Oloo
HIV and TB Implementation Science and Services, KENYA MEDICAL RESEARCH INSTITUTE/CENTERS FOR DISEASE CONTROL RESEARCH AND PUBLIC HEALTH COLLABORATION, Kisumu, Kenya
**Correspondence:** Barbara K. Burmen
*Antimicrobial Resistance and Infection Control* 2017, **6(Suppl 3):**P386

**Introduction:** In 2014, Tuberculosis (TB) laboratories (labs) in Kenya processed approximately 600,000 sputum samples.The same year, Kisumu county of Kenya had a high TB Case Notification Rate (CNR) per 100,000 population; 302 vs 210 nationally.

**Objectives:** To assess laboratories biosafety measures in TB laboratories in Kisumu County.

**Methods:** A cross sectional survey was conducted within TB diagnostic labs in Kisumu County in 2015 using an adopted version of the World Health Organization's (WHO) biosafety recommendations for TB labs (18 administrative, 14 Post Exposure Prophylaxis, 22 codes of practice and 14 Lab Design measures). Overall compliance was defined adherence to at least 75% of each of the four measures. Chi square statistics were used to describe lab characteristics by compliance status.

**Results:** Thirty-seven (32/ 86) TB diagnostic centers in the county were assessed. Majority were based in government-owned health facilities (50%), of level 3 (41%), were not undergoing accreditation (84%), had ≥2 lab technologists (49%), had a lab cleaner (94%) did not have a sample receptionist (84%), and had an average monthly workload of <20 sputum smears. Only 7 (22%) of laboratories were compliant. A significantly higher proportion of compliant labs were observed in level 4 and 5 facilities compared to level 2 and 3 (44% vs. 13%), where there was a specimen receptionist compared to where there wasn't (80% vs. 11%), and among labs undergoing accreditation compared to those that weren't (100% vs. 7%), ($p < 0.05$). A higher proportion of compliant labs was also observed among mission-owned compared to privately-owned and government-owned health facilities (29% vs. 22% and 19% respectively); and where, there were ≥2 lab technologists compared to where there was one (26% vs. 15%), there was a biosafety officer compared to where there wasn't (29% vs. 135) and where the workload was ≥20 sputum smear per month compared to where it was less (33% vs. 10%) ($p \geq 0.05$). Conversely, a lower proportion of labs that had a lab cleaner were complaint compared to where there wasn't (20% vs 50%; $p = 0.3$)

**Conclusion:** Kisumu county Lab personnel operate under suboptimal biosafety conditions. The TB control program ought to enhance and routinely monitor Tb laboratory biosafety measures.

**Disclosure of Interest**

None Declared

### P387

#### Knowledge of occupational risk and practice of risk prevention among hairdressers in a North-Western Community, Nigeria

Olorukooba A. Abdulhakeem[1], Shamsudeen S. Yahaya[2], Lawal Amadu[1], Muhammed S. Ibrahim[1], Mary O. Onoja-Alexander[1], Biliaminu B. Lawal[3], Dahiru Muhammed[1]
[1]Community Medicine, Ahmadu Bello University Zaria, Zaria;
[2]Community Medicine, Ministry of Health Katsina State, Katsina;
[3]Epidemiology, Nigerian Field Epidemiology and Laboratory Training Network, Abuja, Nigeria
**Correspondence:** Olorukooba A. Abdulhakeem
*Antimicrobial Resistance and Infection Control* 2017, **6(Suppl 3):**P387

**Introduction:** It is a fact that hair and beauty salon workers as well as their customers are at risk of infection. The knowledge and practice of risk prevention among this group in developing countries has been shown to be poor thus increasing the risk of infection transmission.

**Objectives:** To determine the knowledge of occupational risk and the practice of risk prevention among hair dressers in Sabon Gari Local Government Area, Kaduna state.

**Methods:** A cross sectional descriptive study was carried out within the months of April and May of 2016. Cluster sampling technique was used to obtain a sample of 56 hairdressers. A pretested semi-structured interviewer administered questionnaire was used to elicit information from the respondents. Data was entered and analysed using IBM SPSS version 21. Chi square test or Fisher's exact test were used as test of association between categorical variables.

**Results:** Majority (76.8%) of the hairdressers were aware of the occupational risk associated with their work. Only 16.3% and 20% of the respondents agreed that HIV and Hepatitis (B and C) respectively can be transmitted in the salon. About three-fifths of the respondents had a good knowledge score. The practice of handwashing before each client was present in 57.1% of respondents while only 79.6% of the hairdressers used new razor blade for each client. Majority (96.4%) sterilized sharp equipment before use. The overall practice of risk prevention was found to be good in 68.5% of the respondents. There was a statistically significant relationship between level of education of the respondents and knowledge score($P = 0.003$). However, no statistically significant relationship was found between their knowledge and practice of risk prevention ($P = 0.713$).

**Conclusion:** Although the overall knowledge and practice of risk prevention was found to be high, their knowledge and practices on certain aspects of the risk was unacceptable. It is recommended that the local government health authorities should institute specific health trainings focused on deficient areas in order to prevent spread of infections.

**Disclosure of Interest**

None Declared

### P388

#### Development of an exposure decision pathway in a suburban fire department

Christine McGuire-Wolfe[1,2], Donna J. Haiduven[1]

[1]Global Health, University of South Florida College of Public Health, Tampa; [2]Infection Control Officer, Pasco County Fire Rescue, Land O'Lakes, United States

**Correspondence:** Donna J. Haiduven

*Antimicrobial Resistance and Infection Control* 2017, **6(Suppl 3):**P388

**Introduction:** Pasco County Fire Rescue (PCFR) provides fire suppression and emergency medical services (EMS) response in a suburban area of Florida (United States). The department employs approximately 475 emergency medical technicians (EMTs) and paramedics and operates 24 hours a day, 365 days a year. Exposure responses are necessary 24 hours per day, including when the Infection Control Officer (ICO) is not in the office.

**Objectives:** The objective of this project was to develop a system to assist field personnel without background in infection prevention in forming a decision regarding exposure response when the ICO was not in the office.

**Methods:** Current guidelines from the United States Centers for Disease Control and Prevention were used to develop a summary decision pathway. Once the questions in the pathway were answered, a "level" was determined which instructed the employee that immediate, moderate, or delayed response from the ICO was appropriate.

**Results:** A color-coded algorithm was developed to detrmine decision levels for each possible exposure situation. For example, questions that indicated a bloodborne pathogen exposure may have occurred were coded green and required an immediate notification of the ICO. Suspicions of exposure to tuberculosis were coded yellow, as baseline testing could be arranged during the next business day.

**Conclusion:** Implementation of a color-coded, binary decision pathway has empowered field personnel to seek professional guidance from the Infection Control Officer when appropriate and has reduced unnecessary after hours calls for issues that could be appropriately delayed until normal working hours.

**Disclosure of Interest**

None Declared

### P389

#### The role of an infection preventionist in a fire department providing emergency medical services (EMS) response

Donna J. Haiduven[1], Christine McGuire-Wolfe[1,2]

[1]Global Health, University of South Florida College of Public Health, Tampa; [2]Infection Control Officer, Pasco County Fire Rescue, Land O'Lakes, United States

**Correspondence:** Donna J. Haiduven

*Antimicrobial Resistance and Infection Control* 2017, **6(Suppl 3):**P389

**Introduction:** Emergency Medical Services (EMS) personnel are not typically included in discussion regarding traditional healthcare workers. However, EMS personnel operate in an environment that increases the risk for exposure to pathogens. Pasco County Fire Rescue utilizes approximately 475 emergency medical technicians (EMTs) and paramedics to provide emergency medical services in the community. Infection Prevention services are provided by the Infection Control Officer (IC0).

**Objectives:** The objectives of this project were to: 1) describe exposure scenarios to personnel within a Fire Department and 2) to demonstrate how such exposures justify the need for an Infection Preventionist in this type of setting.

**Methods:** The two week period from February 1st to February 14th, 2017 was reviewed to delineate the issues and concerns that required attention from the ICO.

**Results:** Within a 14 day period, 4 significant exposures were reported by field personnel (EMTs and paramedics): 1) scabies 2) norovirus 3) hepatitis B and 4) hepatitis C. In addition, the ICO continued to work to promote voluntary tuberculosis (TB) screening program that was implemented. During this time period, the ICO also participated in a newly formed coalition of community partners assembled by the local Health Department to address a significant increase in acute Hepatitis B cases locally.

**Conclusion:** There is a demonstrated need for the guidance and expertise of an Infection Preventionist within agencies providing EMS response.

**Disclosure of Interest**

None Declared

## Coated surfaces

### P390

#### Nanoshield surface coating protection combination with cleaning product cleaner for removal bacterial contamination

Lenka Michalikova, Sona Hnilicova, Jaroslava Brnova

Department of Laboratory Medicine, School of Health Sciences and Social Work, Trnava University, Trnava, Slovakia

**Correspondence:** Lenka Michalikova

*Antimicrobial Resistance and Infection Control* 2017, **6(Suppl 3):**P390

**Introduction:** NANOSHIELD (Nanosystem s.r.o) is a nanotechnology products for surface protection, especially for concrete surfaces, interior and exterior plaster, painted and unpainted metals, glass, natural and synthetic textiles, ceramics and plastics. Combination with cleaning product Cleaner (Nanosystem s.r.o.) inhibited bacteria and destroyed its molecular structure by disrupting the membrane of their cells. It has high cleaning and antibacterial properties. There is no need to use gloves or other protective equipment due to its natural composition.

**Objectives:** We performed monitoring of bacterial contamination in a public toilets using an ATP bioluminescence assay (3 M). We compared bacterial contamination on NANOSHIELD treated surface cleaned with CLEANER and surface without NANOSHIELD

cleaned with traditional cleaning products. Statistical analyses were performed using R-project and P < 0, 05 were considered significant.

**Methods:** We performed monitoring of bacterial contamination in a public toilets using an ATP bioluminescence assay (3 M). We compared bacterial contamination on NANOSHIELD treated surface cleaned with CLEANER and surface without NANOSHIELD cleaned with traditional cleaning products. Statistical analyses were performed using R-project and P < 0, 05 were considered significant.

**Results:** NANOSHIELD was applicate on different surface in public toilets (button flushing, toilet seat, interior door handle on the toilet, faucets, flush). Median RLU on surfaces in public toilet treated with NANOSHIELD cleaned with CLEANER was significant lower (P < 0,05) than surface without NANOSHIELD cleaned with common cleaning products [80 RLU (IQR = 101 – 1569) vs. 2325 RLU (IQR = 1481 – 8483); P < 0,05].

**Conclusion:** On the basis of the measurements ATP, surfaces treated with NANOSHIELD plus CLEANER have significant lower bacterial contamination than surface without NANOSHIELD cleaned with common products. Further studies are necessary to assess the effect this products on microbial contamination of environment, especially in health care setting.

**Disclosure of Interest**

None Declared

### P391

#### "A multi-level antimicrobial coating for patient privacy curtains: implication of a new system to combat hospital acquired infections (HAIS)"

Awais Farid[1], King L. Yeung[2]
[1]ENVR; [2]CBME, Hong Kong University of Science and Technology, Kowloon, Hong Kong
**Correspondence:** Awais Farid
*Antimicrobial Resistance and Infection Control* 2017, **6(Suppl 3):**P391

**Abstract video clip: Background:**

Microorganisms are ubiquitous in the environment. Many bacteria including multi-drug resistant organisms (MDRO), such as Methicillin-resistant *Staphylococcus aureus* (MRSA), *carbapenem-resistant Acinetobacter Baumannii* (CRAB), multi-drug resistant *Acinetobacter* (MDRA) and Vancomycin-resistant Enterococcus (VRE), can survive and persist in the hospital environment, and cause significant mortality and morbidity among patients. Although significant efforts have been made to disinfect hard surfaces in the hospital environment, soft and porous materials such as hospital textiles have been generally ignored. This study aims to investigate the efficacy and long-term performance of a new multi-level antimicrobial system for hospital textiles via laboratory investigation to implement in hospital environment for future.

**Methodology:**

A cationic antimicrobial coating in a compatible micellar structure was produced in the laboratory to generate contact-killing and anti-adhesion property from FDA-approved polymeric materials. An interventional study was designed to assess the antimicrobial activity of polymeric coating against most commonly found hospital acquired infections. The study was completed in 6 months by analyzing the log reduction in treatment arm (antimicrobial coating) against control arm (bleach) on patient privacy curtains.

**Results:** The results of 1 minute contact time of targeted pathogens including MDRO with coated surface on patient privacy curtains in lab showed more than 3 log reduction and 99.97% antimicrobial efficacy. This new micellar antimicrobial formulation has proved to be very efficient against common bacteria, pathogens and MDROs found in the hospital environment. Results also demonstrated the formulation to be durable and providing long-term surface disinfection against microorganisms.

**Conclusion:** As a consequence, this will have the beneficial impact of reducing the risk of transmission of pathogens from environment to patient host, and thus lower the number of HAIs. It will have an immediate impact of decreasing the viability of MDRO and other pathogens in the environment, reduce transmission of diseases and improve both individual wellness and public health.

**Disclosure of Interest**

None Declared

### P392

#### Impact of thermal and mechanical treatment simulating long-term use on antimicrobial effectiveness of selected copper alloys intended for use as touch surfaces in healthcare facilities

Anna Różańska[1], Agnieszka Chmielarczyk[1], Dorota Romaniszyn[1], Małgorzata Bulanda[1], Monika Walkowicz[2], Piotr Osuch[2], Tadeusz Knych[2]
[1]Chair of Microbiology, JAGIELLONIAN UNIVERSITY MEDICAL COLLEGE; [2]Faculty of Non-Ferrous Metals, AHG University of Science and Technology, Kraków, Poland
**Correspondence:** Anna Różańska
*Antimicrobial Resistance and Infection Control* 2017, **6(Suppl 3):**P392

**Introduction:** An enhancement for traditional disinfection methods used in healthcare facilities are touch surfaces made from materials with antimicrobial properties, including copper alloys. A certain downside of copper alloys is their susceptibility to oxidation, which in some extent decreases aesthetic qualities. Therefore, studies concerning the selection of the best antimicrobial effectiveness and utility values for alloys are conducted in different centers.

**Objectives:** The aim of this work was to assess the antimicrobial properties of selected copper alloys, after thermal and mechanical treatment simulating long-term use

**Methods:** The present study involved representatives of Gram-positive (*S. aureus*) and Gram-negative (*E. coli*) bacteria. The experiments employed two variants of the so-called wet exposure – bacterial suspension in NaCl vs. TSB (simulation of clean and dirty environmental conditions), in time periods from 30 to 300 min and quantitative culture.

**Results:** The highest efficiency of ETP copper was confirmed along with bacteriostatic and bactericidal properties for the studied alloys. As regards individual strains tested, a slightly different rate of complete reduction in bacterial suspension density was observed, compared to the results of other authors. For bacterial suspension in NaCl, complete reduction was observed in under 30 min for EC and 120 min for SA, depending on the alloy. For suspension in TSB (SA and EC, simulation of dirty conditions), for each of the alloys tested, reduction higher or equal to 3log was observed in less than 300 min. No significant differences were found as regards the efficiency of antimicrobial activity for non-oxidized alloys and the ones with smooth surfaces, in comparison with alloys subjected to thermal and mechanical treatment (simulation of oxidation and microdamage to the surface during normal use).

**Conclusion:** Prolonged use of touch surfaces made from copper alloys does not influence the effectiveness of their antimicrobial activity.

The project was financially supported by The Polish National Center for Research and Development awarded on the basis of decision PBS3/A9/32/2015

**Disclosure of Interest**

None Declared

### P393

#### Effects of surface stain and wiping using detergents on antibacterial activities of copper

Hiroshi KAWAKAMI, Pengyuan WANG, Yoshihiro SATO, Yasushi KIKUCHI
Department of Mechanical and Physical Engineering, Osaka City University, Osaka, Japan
**Correspondence:** Hiroshi KAWAKAMI
*Antimicrobial Resistance and Infection Control* 2017, **6(Suppl 3):**P393

**Introduction:** Frequently hand-tuched places in hospitals and health care facilities are periodically wiped using detergents. While the wiping will reactivate antibacterial activities of copper, which are inactivated by surface staining, the detergents may degrade the surfaces and inactivate antibacterial activities of copper.
**Objectives:** Effects of oily surface stain and wiping using detergents on antibacterial activities of a copper were investigated.
**Methods:** Sample used was oxygen free copper (>99.6%). Triolein was employed as a model surface stain. Detergents used for wiping were hypochlorites (NaOCl aq.) and ethanol (EtOH). The test surface of specimens was firstly stained by triolein and then subjected to wiping using a sterile wipe loaded with a detergent.
The strain used was *E. coli* (NBRC 3972). Antibacterial tests were carried out according to the ISO 22196.
All data reported were the averages of at least three replicate data sets. The Scheffé test was employed for multiple comparison test and a *p*-value of less than 0.05 was considered statistically significant. Prior to application of the Scheffé test, analysis of variance was carried out to confirm the significance of the data.
**Results:** The bacterial counts for specimens wiped using the detergents were not significantly different from the value for specimens with clean surface (<10 colony forming unit (CFU)). Antibacterial activities of specimens immersed in a detergent for 24 hours were also measured. While the bacterial counts for EtOH treated specimens were about 10 ~ 20 CFU, the bacterial counts for NaOCl aq. treated specimens were $10 \sim 1.0 \times 10^5$ CFU depending on the concentration of free available chlorine.
**Conclusion:** Wiping using detergents, as well as dry wiping, reactivates antibacterial activities of copper, which was inactivated by oily surface staining. Hypochlorites can be harmful to copper surfaces: the wiping using hypochlorites degrades copper surfaces resulting in inactivate antibacterial activities of copper. Further study on the effects of hypochlorites on the antibacterial activities of copper under practical conditions are required.
**Disclosure of Interest**
None Declared

### P394

#### Antibacterial activity of isolated copper particles in clinical samples

Naldy P. Febré[1], Viviana Silva[2], Andrea Báez[3], Humberto Palza[4], Katherine Delgado[4], Isabel Aburto[5], Victor Silva[2]
[1]Universidad Andres Bello, Facultad de Enfermería; [2]Universidad Mayor, Escuela de Tecnología Médica, Santiago; [3]Universidad Austral de Chile, Instituto de Estadística, Valdivia; [4]Universidad de Chile, Facultad de Ciencias Físicas y Matemáticas; [5]Instituto Nacional de Heridas, Santiago, Chile
**Correspondence:** Naldy P. Febré
*Antimicrobial Resistance and Infection Control* 2017, **6(Suppl 3):**P394

**Introduction:** The antimicrobial activity of copper is recognized and it is used as an antimicrobial agent.
**Objectives:** To evaluate the antimicrobial activity of copper against microorganisms obtained from chronic cutaneous wound infections.
**Methods:** We developed a prospective study on bacteria isolated from wound patients and evaluated the antimicrobial activity of five copper particles. These particles were characterized by UV spectrophotometry assays, X-ray diffraction, scanning electron microscopy and mass spectrometry. The activity was evaluated by minimum inhibitory concentration (MIC) and minimum bactericidal concentration (MBC) by macrodilution method following CLSI recommendations. Internationally required ethical standards were met.
**Results:** 104 baterial strains were isolated from 74 patients, 12 of these strains presented multiresistance and were used with five Cu + 2 ions. We selected two copper particles, the MIC for acetate monohydrate copper has an average of 826.6 µg / ml, with ranges of 400 to 1200 µg / ml and mode of 1000 µg / ml. For nitrate trihydrate copper the mean MIC was 1133.3 µg / ml, with ranges from 400 to 1800 µg / ml and 1000 µg /ml. The MBC ranges for acetate monohydrate copper was 600 µg / ml to 2000 µg / ml and for nitrate trihydrate copper was 400 µg / ml to 1600 µg / ml.
**Conclusion:** 1) The release capacity and antibacterial activity determining that both compounds, acetate monohydrate copper and nitrate trihydrate copper are capable of eliminating resistant bacteria; 2) The MIC and MBC values are very close showing high antimicribial activity in safe ranges for humans.
**Disclosure of Interest**
None Declared

### P395

#### Effective antimicrobial banknotes for preventing cross contamination

Henri ROSSET[1], Nathalie VAST[2], Jean GARNIER[3], François RENAUD[4]
[1]Research & Development, ARJOWIGGINS SECURITY, APPRIEU; [2]Marketing, ARJOWIGGINS SECURITY, Paris, France; [3]Silkscreen Laboratory, SICPA SA, Prilly, Switzerland; [4]Education, Lyon 1 University, Lyon, France
**Correspondence:** Henri ROSSET
*Antimicrobial Resistance and Infection Control* 2017, **6(Suppl 3):**P395

**Introduction:** Banknotes carry bacteria, fungi and virus. Thus, banknote handling could lead to the potential risk of cross contamination.
**Objectives:** To manage this risk, we have adopted a new approach by enhancing the antimicrobial performance of banknotes through the use of specific active components incorporated in the banknote paper (Bioguard®) and in post-printing varnish (SICPAPROTECT®).
**Methods:** Specimen banknotes for the study were treated by means of 2 processes: incorporation of agents in the paper (Bioguard®), and/or incorporation of agents in the post-printing varnish (SICPAPROTECT®). The antibacterial activities of the specimens have been measured by means of the standard ISO 20743 against *Staphylococcus aureus* (SA) and *Escherichia coli* (EC). Antifungal activity has been assessed using the AATCC test method 30:2004 part III with *Aspergillus niger* (AN). Moreover, treated Bioguard® paper has been studied in a field test on Philippine banknotes.
**Results:** The antimicrobial performances of untreated and unvarnished paper were 78% (SA) and 16% (EC). The unvarnished but treated paper (Bioguard®) showed antimicrobial activities of up to 99.6% (SA) and 99.01% (EC). Varnished and treated paper has even higher performance of 99.84% (SA) and 100% (EC). Untreated paper with post-printing varnish SICPAPROTECT® containing agents against pathogens showed antimicrobial performance of 98.7% (SA) and 99.73% (EC).
In the real-life circulation test, uncirculated treated banknotes showed antibacterial performance between 99.98% and 100% against SA and EC. The performance remained at 99.97% (SA) and 100% (EC) for banknotes after 8 and 12 months in circulation. Most of the circulated banknotes were protected against AN.
**Conclusion:** Effective antimicrobial performance of treated banknotes with Bioguard® can be observed. The banknotes with Bioguard® treatment and/or SICPAPROTECT® varnishes can achieve durable antimicrobial performance.
Using banknotes with Bioguard® paper and/or post-printing varnishes SICPAPROTECT® could be an efficient way for reducing transmission risk of germs during banknote handling.
**Disclosure of Interest**
None Declared

### Global health

#### P396

**Effectiveness of public health education by lecture on improving the knowledge, attitude and practices on leptospirosis and its management among adolescents in a public school in Manila**

Jenna Angela D. Rubio[1], carlos T. Nunez[2], jennie A. Wong[2]
[1]Pediatrics, Pamantasan ng Lungsod ng Maynila-Ospital ng Maynila Medical Center, Las Pinas; [2]Pamantasan ng Lungsod ng Maynila-Ospital ng Maynila Medical Center, Manila, Philippines
**Correspondence:** Jenna Angela D. Rubio
*Antimicrobial Resistance and Infection Control* 2017, **6(Suppl 3):**P396

**Introduction:** Leptospirosis is endemic worldwide. Based on the Philippine Pediatric Society Disease Registry, there were 800 cases of leptospirosis from May 2006 to August 2016. Making it a public health concern for health care facilities. Case fatality rate is about 8-9%, with increased prevalence of the disease among adults and adolescents.

**Objectives:** To evaluate the effectiveness of public health education by lecture on improving the knowledge, attitude and practices about leptospirosis among adolescents in grade 7-10 in a public school in Manila.

**Methods:** This is a prospective cross-sectional analytical study design. A pre-tested, self-administered questionnaire was administered among 357 students in a public school in Intramuros, Manila. Lecture regarding leptospirosis was done by the author. A post-test was done after the lecture. Statistical method used was Paired t-test.

**Results:** A total of 357 students were included in this study, with 88.37% response rate. Total mean pre-test knowledge score was 88.64% for the topic of leptospirosis. For the attitude associated with leptospirosis, total mean pre-test score was 80.97%. For the practices related to leptospirosis, respondents had a total mean pre-test score of 72.12%. Pre-test knowledge scores were compared to the post-test scores. After a lecture was conducted, there was a significant increase on their knowledge on leptospirosis ($p = <0.0001$). There was also an improvement on post-test scores on attitude, as well as the practices regarding leptospirosis ($p = <0.0001$).

**Conclusion:** The results showed that there is a significant improvement in knowledge, attitude and practices for leptospirosis after an informative lecture. Positive attitude should be complemented with knowledge, to enhance the ability of individuals to integrate preventive measures into daily practice. One of the more important findings in this study, with respect to public health impact, is increasing the awareness regarding leptospirosis may help in the prevention of the disease in the adolescent population.

**Disclosure of Interest**
None Declared

#### P397

**Development and effectiveness of clinical pathway for emerging infectious diseases**

Ju Hyun Lee[1], Eunsuk Park[1], Oh Mee Kweon[1], Dongsuk Lee[1], Eun Jin Ha[1], Nam Su Ku[1,2], Jun Yong Choi[1,2]
[1]Department of infection control, Severance hospital; [2]Department of Internal Medicine, Yonsei University College of Medicine, Seoul, Korea, Republic Of
**Correspondence:** Ju Hyun Lee
*Antimicrobial Resistance and Infection Control* 2017, **6(Suppl 3):**P397

**Introduction:** Delayed screening of emerging infectious diseases may lead to a massive epidemic such as that of the Middle East Respiratory Syndrome (MERS) in Korea in 2015.

**Objectives:** Severance is a 2,400-bed hospital treating more than 16 thousand patients from abroad annually, demands for a system that promptly discovers and isolates patients with an emerging infectious disease.

**Methods:** In January 2016, an emerging infectious disease CP was developed by the Infectious Disease Response Team. Information on countries of breakout and symptoms of emerging infectious diseases were posted on the electronic bulletin board and was updated weekly. When patients were admitted or visited the hospital, the physician in charge was instructed to record countries of recent visit, date of entry, and symptoms. Patients with suspected emerging infectious disease were transferred to the negative pressure isolation room located outside the hospital, and patient information was text messaged to the director of infectious diseases. Once the director of infectious diseases activated CP, the patient information was forwarded to all members of the infectious disease response team, and the medical staff provided care with proper personal protective equipments. Furthermore, patients were reported to the public healthcare center then transferred to the designated isolation hospital according to the instructions by the Korea Centers for Disease Control.

**Results:** Six patients were CP-activated as suspected MERS patients from February, 2016 to January, 2017. With the exception of two patients whose CP was deactivated after being treated, the remaining four patients underwent the standard procedure and were transferred to a nationally designated isolation hospital in an average of 4 hours and 30 minutes. We were able to minimize worker exposure to MERS suspect patients to an average of 17.5 people, and the management protocol for exposed individuals was concluded when all of the suspected case results were negative on the MERS test.

**Conclusion:** The CP for emerging infectious diseases was effective in minimizing exposure time and individuals that were exposed by standardizing the early response protocol for patients suspected to have an emerging infectious disease.

**Disclosure of Interest**
None Declared

#### P398

**Effects of local climate variables on the dengue morbidity in Davao Region, Philippines**

Jesavel Iguchi[1], Xerxes Seposo[1], Yasushi Honda[2]
[1]Graduate School of Comprehensive Human Sciences; [2]Faculty of Health and Sports Sciences, University of Tsukuba, Tsukuba City, Japan
**Correspondence:** Jesavel Iguchi
*Antimicrobial Resistance and Infection Control* 2017, **6(Suppl 3):**P398

**Introduction:** Dengue fever is a major public health concern. It causes 390 million infections annually (1). It is one of the significant causes of hospitalization and death among young children in the Philippines (2). According to CDC, dengue is highly sensitive to climate where temperature, precipitation, and humidity are critical to mosquitoes' survival, reproduction, and development (3).

**Objectives:** This study aims to evaluate the effects of climate variables on the dengue morbidity in Davao Region, Philippines.

**Methods:** The secondary data, i.e., the weekly dengue incidences (2011-2015) in Davao Region, Philippines were obtained from the Department of Health-Davao (DOH-Region IX). The case classifications are suspected cases and are not laboratory confirmed. For the weekly local climate variables, the data were obtained from the National Oceanic and Atmospheric Administration (NOAA-USA, NCDC) website. The report covers the weekly average temperature, rainfall, and dew point. Using a Poisson regression combined with distributed lag nonlinear model (DLNM), we evaluated the non-linear and delayed effects of climate variables on the dengue incidences. Wavelet coherence analysis was also used to detect dengue periodicity.

**Results:** From the time series plot of weekly dengue incidences by year, we found peaks of dengue incidences around the months of June, July, August and September which are the rainy months in the Philippines. The wavelet coherence analysis showed that there were strong coherence between dengue incidences and rainfall between the year 2012-2015. The DLNM showed, that the estimated effect of rainfall on dengue cases obviously differed for low and high cumulated rainfall. There was a high Relative Risk (RR) at high cumulated rainfall for the period of 8 weeks (lag 8). And there was a rapid increase of RR at above cumulative rainfall of 80-100 mm.

**Conclusion:** Our results showed that climate variables affects dengue incidences. In particular, we found that rainfall is positively correlated with dengue incidences.

Disclosure of Interest

None Declared

### P399

#### Saliva as an alternative sample for malaria detection and epidemiological surveillance: a cross sectional study in the centre and South West Regions in Cameroon

Palmer Masumbe Netongo[1], Séverin Donald Kamdem[* 2], Barbarra Atogho-Tiedeu [1], Jean Paul Chedjou[1], Ardin Marius Ouayoue Noutong[1], Theophile Patrick Djiokeng[2], Sophie Wankio Toju[2], Jessica Yonga[1], Kenji Obadia[3], Eric Berenger Tchoupe[4], Prisca Djivida[4], Irenee Domkam[5], Magatte Ndiaye[6], David Visanuvimol[7], Wilfred Mbacham[4], Rose Leke[4] on behalf of Molecular Diagnostic Research Group
[1]Biochemistry, Biotechnology Center, University of Yaoundé I; [2]Microbiology / Immunology, School of Health Sciences, Catholic University of Central Africa, Yaoundé, Cameroon; [3]Hawai University, Hawai, United States; [4]Biotechnology Center, University of Yaoundé I; [5]Centre International de Reference Chantal Biya, Yaoundé, Cameroon; [6]Cheikh-Anta-Diop University, Dakar, Senegal; [7]Genotek, Ontario, Canada
**Correspondence:** Séverin Donald Kamdem
*Antimicrobial Resistance and Infection Control* 2017, **6(Suppl 3):**P399

**Introduction:** Malaria is a serious public health burden in Cameroon. Accurate diagnosis of malaria is essential for the avoidance of unnecessary presumptive treatment. Notwithstanding the benefits of blood-based tests, their invasive nature requires trained personnel and raises the risk of accidental transmission of infectious diseases. To overcome these obstacles, alternatives were explored. In contrast to blood, saliva presents a reduced biohazard and can be painlessly collected in relatively large quantities by individuals with moderate training.

**Objectives:** Determine the sensitivity of saliva comparing to blood in the molecular detection of *Plasmodium falciparum* using PCR.

**Methods:** A cross-sectional study was carried out in the Obala (centre region) and Kumba district hospitals (South west region). Blood and saliva were collected from each participant and analyzed by nested PCR, targeting the species-specific nucleotide sequence of the small subunit ribosomal RNAgene (18S rRNA) of *P. falciparum*. R-software version 3.0.2 was used to carry out the statistical analysis.

**Results:** From the 100 participants included, 52% was male and 48% female with a sex ratio male / female of 1.08. Malaria prevalence using PCR was 57% and 35% respectively with blood and saliva. When compared to blood, saliva showed a sensitivity; specificity; positive predictive value and negative predictive value of 49.20%; 71.42%; 75.60% and 43.85%. The agreement between saliva and blood was weak but significant ($\kappa = 0.181$; *p-value* = 0.024). In addition, the observed agreement (57.14%) was higher than the expected (47.67%).

**Conclusion:** Saliva is a suitable alternative specimen for malaria diagnostic and epidemiological surveillance.

**Disclosure of Interest**

None Declared

### P400

#### Detection of plasmodium falciparum DNA in human saliva: loop-mediated isothermal amplification for malaria diagnosis

Palmer MASUMBE NETONGO[1], Ardin Marius OUAYOUE NOUTONG[1], Severin Donald KAMDEM[2], Barbara ATOGHO-TIEDEU[1], Jean Paul CHEDJOU[1], Theophile Patrick DJIOKENG[2], Sophie WANKIO[2], Obadia KENDJI[3], Jessica YONGA[1], Eric Berenger TCHOUPE KAMOUA[1], Prisca DJIVIDA[1], Irene DOMKAM[4], Magatte NDIAYE[5], David VISANUVIMOL[6], Wilfred MBACHAM[1], Rose LEKE[7] on behalf of The MOLECULAR DIAGNOSTICS RESEARCH GROUP
[1]Biochemistry, University of Yaounde I; [2]Microbiology/Immunology, School of Health Sciences, Catholic University of Central Africa, Yaounde, Cameroon; [3]University of Hawaii, Honolulu, United States; [4]Centre International de Reference Chantal Biya, Yaounde, Cameroon; [5]Université Cheikh-Anta-Diop, Dakar, Senegal; [6]DNA Genotek inc, Ottawa, Canada, [7]Biotechnology centre, University of Yaounde I, Yaounde, Cameroon
**Correspondence:** Ardin Marius OUAYOUE NOUTONG
*Antimicrobial Resistance and Infection Control* 2017, **6(Suppl 3):**P400

**Introduction:** Current malaria diagnostic methods use blood which increases the risk of blood-borne infectious diseases. Saliva is readily available and easy to collect but has not received much attention hitherto. Molecular methods such as PCR showed great benefit but the high cost and availability only in well-equipped laboratories render this technique inadequate for routine diagnosis. Recently, a new, rapid, simple, and sensitive molecular technique called Loop-mediated isothermal amplification (LAMP) was developed.

**Objectives:** Assess the sensitivity and specificity of LAMP for the detection of *Plasmodium falciparum* DNA in saliva of malaria patients.

**Methods:** From 1$^{st}$ to 23$^{rd}$ December 2015, 100 febrile patients referred to the District Hospitals in Kumba and Obala towns found in the South-West and Centre Regions of Cameroon participated in the study. Saliva and blood samples were collected and analyzed by LAMP, targeting the species-specific nucleotide sequence of the small subunit ribosomal RNA gene (18S rRNA) of *P. falciparum*. R-software version 3.0.2 was used to carry out the statistical analysis.

**Results:** When compared to LAMP Blood as standard, the Observed Agreement of Saliva-LAMP was 49% against an Expected Agreement of 43.88% (карра = 0.091, Standard Error = 0.064, Z = 1.42, P value = 0.078). The sensitivity was 44.04%, a Positive Predictive Value of 90.24% while specificity was 75% with a Negative Predictive Value of 20.33%.

**Conclusion:** LAMP technology matches the WHO recommendations for an ideal diagnostic test for developing countries. Considering the field adaptability of LAMP technology and the ease of saliva sampling, further research into the method is worthwhile.

**Disclosure of Interest**

None Declared

### P401

#### The roles of periplaneta americana cockroaches in dissemination and genetic alteration of Oral Polio Vaccine (OPV) virus in Nigeria

Okikiola M. Olajide[1], Olajide O. Agbede[2], Amase Nyamngee[2], Bamidele S. Oderinde[3]
[1]Microbiology, Ahmadu Bello University, Zaria; [2]Medical Microbiology and Parasitology, University of Ilorin, Ilorin; [3]Microbiology, University of Maiduguri, Maiduguri, Nigeria
**Correspondence:** Okikiola M. Olajide
*Antimicrobial Resistance and Infection Control* 2017, **6(Suppl 3):**P401

**Introduction:** Poliovirus is an enterovirus transmitted by the faecal-oral route. The Oral Polio Vaccine (OPV) given to children in Nigeria continues to be shed in faeces weeks after vaccination.

**Objectives:** As the polio eradication programme in Nigeria progresses, it is expedient to confirm the role of coprophagous insects such as cockroaches, known to feed on sewage, in the transmission and genetic modification of the virus.
**Methods:** In-bred *Periplaneta americana* cockroaches were fed with trivalent oral polio vaccine (tOPV) and their products were harvested continuously for 30 days for virus isolation by culture on L20B and Rhabdomyosarcoma cell lines. Samples collected from both test and control groups of cockroaches in 30 days (faeces, saliva, body, legs, shedded skin and oothecae) were processed and 200 μl of the resulting supernatant was inoculated on the respective monolayered cell lines. Positive samples identified with evidence of cytopathic effects were harvested and intratypic differentiation (ITD) screening was carried out using rRT-PCR.
**Results:** Faecal samples of days 1, 3, and 5 had poliovirus serotypes P1, P2, P3; P1; and P1 respectively. The saliva collected on day 1 also had P1, the body collected on day 1 had P3, and the oothecae had the three serotypes. The Sabin vaccine-derived poliovirus (VDPV) assay done using rRT-PCR showed that there was no genetic change in the virus isolated and none had sequences divergent to Sabin strains present in tOPV.
**Conclusion:** Results showed that it is possible for *Periplaneta americana* cockroaches to mechanically transmit poliovirus at the earliest stage of contact especially through the faeces, saliva and body. Genetic modification of the tOPV virus does not occur within *Periplaneta americana* cockroaches, however, some serotypes of the virus are lost in the cockroaches. The study suggests that the oothecae could be a key source of infection, and also shows the higher potency of poliovirus serotype 1 followed by type 3.

Disclosure of Interest
None Declared

### P402

#### Seroparasitology investigation of trichomoniasis in women referred to health centers of Rasht City, Iran

Zarrintaj Valadkhani[1], Roya Ramezanian[2], Mahdi Assmar[2]
[1]Pasteur Institute of Iran, Tehran; [2]Lahijan unit, Islamic Azad University, Lahijan, Iran, Islamic Republic Of
**Correspondence:** Zarrintaj Valadkhani
*Antimicrobial Resistance and Infection Control* 2017, **6(Suppl 3):**P402

**Introduction:** Trichomoniasis is one of the most common sexually transmitted disease that caused by trophozoite *Trichomonas vaginalis*. This parasite can infect urogenital tract in women. The most important point about this infection is diagnosis and treatment of the patients and their sexual partners, in order to prevent from vaginal discharge with an unpleasant smell, vaginal spotting, genital burning or itching, frequent urge to urinate, pain during urination or sexual intercourse. This infection also has impact on premature rupture of membranes and low birth rate,as well as it is considered to be a significant cause of neonatal morbidity.
**Objectives:** In the present study the referring women's samples were investigated with direct smear, staining, culture and indirect immunoflourscent antibody methods for *Trichomonas vaginalis* infection.
**Methods:** This study was conducted on 120 women who were referred to health centers in Rasht city, Iran. The history of the patients was collected by questionnaires. The vaginal-cervical secretions were collected for direct and staining methods as well as for TYI-S-33 culture media. Meanwhile the serum samples also were collected for doing immune-fluorescent antibody (IFA) test.
**Results:** Out of 120 women that were checked by gynecologist, some suspected patients based on their clinical observation/complain had faced. But only one of them was confirmed by culture media and direct smear (0.83%). By using IFA test, the numbers of infected patients to *Trichomonas vaginalis* were increased to seven (5.83%). All these infected patients were symptomatic for trichomoniasis.
**Conclusion:** The sexually transmitted diseases have similar symptoms and signs in the patients, thus it is recomended the gynecologist use the laboratory diagnostic methods in order to prescribe the suitable drugs. Using culture media as diagnostic method has special criteria for its growth, however contamination with bacteria is a major problem, other methods in this study also have low sensitivity and specificity, however due to high sensitivity and specificity of IFA test, using this method is recommended. Although the infected men are mostly asymptomatic, but it should be considered that the couple's treatment is simultaneously.
**Disclosure of Interest**
None Declared

### P403

#### Confirmatory assays for detection of neisseria gonorrhoeae using real-time pcr base methods

Edobor P. Imarenezor
Microbiology, Federal University Wukari, Taraba State, Wukari, Nigeria
*Antimicrobial Resistance and Infection Control* 2017, **6(Suppl 3):**P403

**Introduction:** Ever since the advent of molecular techniques, diagnosis of *Neisseria gonorrhoeae* has been ruin by false positive results when compared with culture, which is currently the gold standard. False positives results are often due to the cross-reaction of nucleic acid amplification test (NAAT) with closely related non-pathogenic Neisseria species.
**Objectives:** This study aimed to institute and compare the sensitivity and specificity of previously available N. *gonorrhoeae* real-time assays which target the following genes: porA pseudogene and cppB gene.
**Methods:** Optimization of the PorA pseudogene real-time PCR was carried out by varying the concentration of magnesium chloride as follow: 5 mM ranges between 19.08 (4.31) and 23.27 (17.57), 4 mM ranges from 17.18 (1.15) and 22.01 (16.43) and for 3 mM the ranges is from 21.71 (2.20) and 27.33 (15.27) with the standard deviation in bracket and as well as the forward and reverse primers which has varying concentration as 50 mM, 300 mM and 900 mM for both the forward and reverse primers.
**Results:** The 30 non gonococci specimens gave negative results. This shows that PorA pseudogene real time PCR is a suitable assay for the confirmation of putative N. *gonorrhoeae* cultures and can assist in identification. The potential of the PorA pseudogene real-time PCR to detect the presence of N.*gonorrhoeae* specific DNA directly from clinical samples was then evaluated. An initial experiment was performed which involved the addition of a primer and probe set which acted as an internal control, it was determined that the internal control did not compromised the sensitivity of the PorA pseudogene real-time PCR assay and could be used reliably to screen for assay inhibition. The PorA pseudogene real-time PCR was then used to examine some clinical specimens which had been examined previously at three laboratories, each of which different commercial N. *gonorrhoeae* NAAT platforms was used. The results from this investigation show a high specificity evidence of PorA pseudogene real-time PCR when compared to previous results obtained from the other laboratories.
**Conclusion:** The study has succeeded in establishing to very large extent that the PorA pseudogene real-time PCR is a very valuable assay for the detection and confirmation of N. *gonorrhoeae* specific DNA from both putative cultures and directly from clinical samples.
**Disclosure of Interest**
E. Imarenezor Employee of: federal university wukari, taraba state

## Environmental control and cleansing

### P404

#### Effectiveness of isopropyl alcohol and ultraviolet based sanitizer on decontamination of mobile phones used by dental personnel

Srikanthan Sriram, Parangimalai Diwakar Madan kumar
PUBLIC HEALTH DENTISTRY, RAGAS DENTAL COLLEGE & HOSPITAL, CHENNAI, India
**Correspondence:** Srikanthan Sriram
*Antimicrobial Resistance and Infection Control* 2017, **6(Suppl 3):**P404

**Introduction:** Mobile phones have become an inevitable mode of communication. Dental office and the dental operators along with their personal equipment (mobile phones) are exposed to numerous pathogens as a part of their profession, which serve as an exogenous source of nosocomial infection.
**Objectives:** This study aimed at assessing the effectiveness of isopropyl alcohol and a customized Ultaviolet chamber,in decontamination of mobile phones.
**Methods:** A cross sectional study was carried out on 30 touch screen mobile phones belonging to dental professionals in a college setting. Swabs were collected along the screen, camera lens and on/off buttons of mobile phones which are frequently contacted. Swabs were streaked onto nutrient agar (NA) and incubated at 37 °C for twenty four hours for assessment of microbial load before and after the disinfection procedures. The disinfection process was performed using 70% isopropyl alcohol and an Ultra Violet chamber (TUV/15 W/G15 T8). Mann Whitney- U test was used to compare the values between the two groups. Wilcoxon Signed Ranks Test was used to compare the values within each group
**Results:** There was a statistically significant reduction in the mean number of colonies ($p = 0.001$) after decontamination by the two groups (Isopropyl alcohol and UV chamber) indicating that both agents were effective in disinfection. The reduction in microbial load in the mobile phones post intervention was 79.89% in the isopropyl alcohol group and 71% in the UV chamber group.
**Conclusion:** The study concluded that the percentage reduction in microbial load of the mobile phones was better with isopropyl alcohol compared to UV chamber. It is recommended that mobile phones in the dental setup be regularly decontaminated and dentists must adhere to strict infection control protocols specifically in relation to hand hygiene.
**Disclosure of Interest**
None Declared

### P405
### Mobile phones: a boon or bane in the hospital setting?
James M. Maragia
Laboratory, Lodwar County and Referral Hospital, Lodwar, Kenya
*Antimicrobial Resistance and Infection Control* 2017, **6(Suppl 3):**P405

This abstract is not included here as it has already been published.

### P406
### Short-term efficacy of introducing a wipe for stethoscopes cleaning: a before and after study in clinical setting
Nuno Rocha-Pereira[1,2], Cristóvão Figueiredo[2], André Silva-Pinto[2], Abel Gomes[1], Arnaldo Dias[1], Cláudia Pereira[1], Gisélia Braga[1], Carlos Lima-Alves[1]
[1]Unit of Prevention and Control of Infections and Antimicrobial Resistance; [2]Infectious Diseases Department, Centro Hospitalar São João, Porto, Portugal
**Correspondence:** André Silva-Pinto
*Antimicrobial Resistance and Infection Control* 2017, **6(Suppl 3):**P406

**Introduction:** Stethoscopes may serve as vectors in the transmission of infectious agents.
**Objectives:** Evaluate efficacy of introducing an alcohol-free wipe (AFW) in reducing biological burden in stethoscopes (BBS). Evaluate stethoscopes cleaning practices.
**Methods:** A quasi-experimental before and after study was performed in a general medical ward during 11/2015. All healthcare workers or students using a stethoscope were included after consent. Introduction of AFW Mikrobac®), 5-minute talk and response to a questionnaire evaluating hand hygiene and stethoscopes cleaning practices composed the intervention. BBS was measured with a bioluminescence ATP monitoring device. Continuous variables were summarized by median and interquartile range, while categorical variables were presented as proportions. Statistical significance was admitted when $p < 0,05$.
**Results:** 144 individuals were included in the study, 72 pre-intervention and 72 post-intervention. A significant reduction in BBS was noticed [Median ATP count:73(IQR 27–379,5) VS 31,5(IQR 5-104,25), $p = 0,001$]. In pre-intervention evaluation, 11(15,3%) individuals reported cleaning before and after each patient and 27(37,5%) reported weekly cleaning. After intervention, 27(37,5%) professionals reported daily cleaning and 23 (31,9%) cleaned every other day. Before intervention 36 (50,0%) individuals used dressings soaked in alcohol for cleaning and 28 (38,9%) dressings soaked in alcoholic hand hygiene solution; after intervention 41 (56,9%) professionals reported use of AFW and 11 (15,3%) dressings soaked in alcohol.
**Conclusion:** Introduction of AFW alongside with educational intervention showed short-term efficacy in reducing BBS in a general medical ward. Intervention also changed professionals' cleaning practices. Further investigation is needed about potential impact of this kind of strategy in reducing healthcare associated infections and about the durability of intervention's effect.
**Disclosure of Interest**
None Declared

### P407
### Microbial contamination on transvaginal ultrasound probes in a gineco-obstetrics department of a tertiary hospital in Spain
Paula Peremiquel Trillas[1], José Ángel Rodrigo-Pendás[1], Lluís Armadans-Gil[1], Jessica Gonzalez-Montes[1], Maria Goya-Canino[2], Elisa Llurba-Olivé[2], Juliana Esperalba-Esquerra[3], Virginia Rodríguez-Garrido[3], Glòria Roig-Carbajosa[3], Elena Carreras-Moratonas[2], Magda Campins-Martí[4]
[1]Preventive Medicine and Epidemiology Department; [2]Obstetrics and Gynaecology Department; [3]Microbiology Department; [4]reventive Medicine and Epidemiology Department, Hospital Universitari Vall d'Hebron, Barcelona, Spain
**Correspondence:** Paula Peremiquel Trillas
*Antimicrobial Resistance and Infection Control* 2017, **6(Suppl 3):**P407

**Introduction:** Transvaginal ultrasound (TVUS) probes are semicritical devices routinely used with disposable protective covers. These covers can suffer ruptures (between 1-9% as described in literature) that facilitate probe contamination with pathogenic microorganisms present in the vagina. Transmission to other patients can occur if disinfection of probes is not done properly after each use.
**Objectives:** The objective of this study is to determine the prevalence of microbial contamination of TVUS probes in our hospital.
**Methods:** Cross-sectional study. Swap samples were taken from TVUS probes available for use in the exploration units, theoretically after standard chemical disinfection at point of use. Investigation of any bacteria and fungi growing was done by culture and detection of Human papillomavirus (HPV), *Chlamydia trachomatis* and *Neisseria gonorrhoeae* by molecular techniques.
**Results:** A total of 51 samples were taken from 17 TVUS probes. The prevalence of contamination was 70.59% (36/51). 64.71% (33/51) of the samples contained skin and environmental flora. In 19.61% (10/51) of the samples nonfermenting Gram-negative bacteria grew and in 5.88% (3/51) of them methicillin-sensitive *S. aureus* grew. Two samples were HPV positive for 3 different genotypes. None of the samples were positive for fungi, *Chlamydia trachomatis* or *Neisseria gonorrhoeae*.
**Conclusion:** Skin and environmental flora are frequently isolated in TVUS probes. HPV contamination is significant on TVUS probes, as described by other authors. As there is a risk of transmitting infections via TVUS procedures, TVUS probes must be properly disinfected, using high-level disinfectants. Healthcare workers must be educated about the risks associated with this procedure and the importance of the proper disinfection technique.
**Disclosure of Interest**
None Declared

### P408

#### Global survey on the handling of human waste and bedpan management

Gertie V. Knippenberg-Gordebeke[1], Tim T. Lieske[2], Pola Brenner [3], Jim Gauthier[4]
[1]KNIP KNowhow Infection Prevention, Consultancy Infection prevention, Venlo, Netherlands; [2] Hospital Hygiene, Uni. Hospital, Essen, Germany; [3]Department of Microbiology, Universidad de Valparaiso, Valparaiso, Chile; [4]Infection Prevention, Sealed Air Diversey Care, Mississauga, Ontario, Canada
**Correspondence:** Gertie V. Knippenberg-Gordebeke
*Antimicrobial Resistance and Infection Control* 2017, **6(Suppl 3):**P408

**Introduction:** It is estimated that 10% of the global population are carriers of multi drug resistant organisms (MDRO). Unsafe disposal of faeces and urine and improper decontamination of bedpans and urine bottles (medical devices) pose risks for transmission of MDRO. Many healthcare settings do not have clear steps to outline bedpan management. Improper handling can lead to contamination of the environment and the hands. Safer disposal of faeces can help to minimize the risk for Healthcare Associated Infections (HAIs).
**Objectives:** To determine and assess the awareness for procedures related to bedpan management within the participant's setting.
**Methods:** 60 observations, 69 standardized interviews and 399 experience exchanges about bedpan management with infection prevention professionals in 30 countries were performed (2013-2017)
A global digital survey in English and Spanish (2017) was sent to professionals in healthcare settings
**Results:** Insufficient procedures to address the transport of full bedpans and urine bottles and insufficient decontamination methods. Dirty utility rooms are poorly equipped and potential reservoirs of MDRO.
Preliminary questionnaire results 24/03 (Final results to be presented at ICPIC)
177 responders from 32 countries
24% local guidelines for bedpan management in case of MDRO
47% no equipment for emptying and decontamination for bedpan/ urine bottle
10% use a Macerator, 45% use a Washer/Disinfector(WD),14% use a Liner (Coverbag)
88% bedpan emptied in toilets or slophoppers
32% no separation of clean and dirty items in the sluice room
Role bedpan management in outbreaks:
35% never searched, 5% Macerator, 9% WD,17% bedpans
Infections: urinary tract, gastrointestinal, wound, other
Publishing of these outbreaks: No: 89%, Yes: 5%
**Conclusion:** The handling of human waste, and proper bedpan management needs to be addressed in all healthcare facilities as a risk reduction initiative for (HAIs).
Reusable bedpans are medical devices and require more rigorous decontamination with improvements in implementation of new strategies.

Disclosure of Interest
G. Knippenberg-Gordebeke Consultant for: MEIKO, CLEANIS, T. Lieske: None Declared, P. Brenner : None Declared, J. Gauthier: None Declared

### P409

#### Microbial dissemination from a patient toilet

Jesse Cooper[1], Elizabeth Bryce[2], Sarah Henderson[3], George Astrakianakis[1], Karen Bartlett[1]
[1]School of Population and Public Health, University of British Columbia; [2]Vancouver Coastal Health; [3]British Columbia Center for Disease Control, Vancouver, Canada
**Correspondence:** Jesse Cooper
*Antimicrobial Resistance and Infection Control* 2017, **6(Suppl 3):**P409

**Introduction:** The role of the hospital environment in transmission of healthcare-associated infections (HAIs) in recent years has gained increased attention. Although flushing toilets are known to generate droplets and aerosols, their potential contribution to pathogen transmission and environmental contamination in hospitals has not received due attention.
**Objectives:** This study aimed to evaluate airborne dissemination of Gram negative and Gram positive organisms, and a norovirus-surrogate (MS2 bacteriophage) from a flushing toilet in a patient bathroom.
**Methods:** We used an *Escherichia coli* and *Enterococcus faecalis* inoculum, as well as a norovirus-surrogate, MS2 bateriophage, inoculum to evaluate bioaerosol generation from a patient washroom in an acute care facility, by sampling at multiple time points post-flush and at four locations in close proximity to the toilet. Our analysis included calculating measures of central tendency (geometric mean), variance (geometric standard deviation), and upper 95% confidence limits for airborne concentrations.
**Results:** Airborne *E. coli* were more abundant than *E. faecalis* immediately post-flush and at the closest sampling location (866 CFU/$m^3$ vs. 533 CFU$m^3$). At all other time points and locations, *E. faecalis* concentrations were significantly higher than *E. coli*. Based on the upper confidence limits, airborne *E. faecalis* concentrations may be greater than 1000 CFU/$m^3$ 8 minutes post-flush and 140 CFU/$m^3$ up to 30 minutes post-flush. MS2 bacteriophage may be present at over 1000 PFU/$m^3$ up to 8 minutes post-flush and 400 PFU/$m^3$ 45 minutes post-flush.
**Conclusion:** The Gram positive organism used in this study persisted longer in the toilet plume, with significantly higher airborne concentrations compared to Gram negative *E. coli*. This finding is consistent with studies showing Gram positive bacteria displaying greater resistance to desiccation both in air and on surfaces. Thus, the toilet plume may pose a greater risk for transmission of Gram positive organisms compared with Gram negative. Based on MS2 bacteriophage persistence, airborne norovirus from toilet flushing may significantly exceed its infectious dose of 18 virions up to forty-five minutes post-flush.
**Disclosure of Interest**
None Declared

### P410

#### Mattresses and covers: sleeping with the enemy!

Carlos A. Palos, Patricia Pereira, Ana S. Bispo
Infection Control Comittee, Hospital Beatriz Ângelo, Loures, Portugal
**Correspondence:** Carlos A. Palos
*Antimicrobial Resistance and Infection Control* 2017, **6(Suppl 3):**P410

**Introduction:** Hospital Beatriz Ângelo (HBA) is a JCI-accredited, 425-bed, paper-free, general hospital, opened in 2012. In 2014, HBA started to notice problems with mattresses, in parallel with a huge increase in the incidence of Carbapenem-Resistant Bacteriae (CRE), mainly *Klebsiella* (CRK). Mattresses and covers are the environment component closer to patients. Several safety alerts have been issued since 2010. In particular, covers might be OK on visual inspection after patient discharge. However, this may not be true, and microperfurations, mainly where patient's pelvic zone is applied, can be revealed only by opening the covers.
**Objectives:** Uncover infection risk on hospital covers and mattresses
**Methods:** In order to improve detection of damaged covers, in addition to recommended procedures, opening of covers was implemented after patient's discharge. If soiled covers were detected, mattresses where then removed from use. This was a part of a sequential intervention, comprising implementation/reinforcement of several aspects: real-time antibiotic stewardship, hand hygiene awareness and auditing, environmental cleaning (including moving on to hydrogen peroxide and 1-step cleaning approach), inclusion of CRE on the Electronic Epidemiologic Questionary on Admission tool, creation of dedicated beds for cohorting patients and staff and, finally, replacement of all mattresses.
**Results:** After opening apparently well covers, heavy contamination was found in the internal side and mattresses were also contaminated and microbiologic studies revealed the presence of bacteria, mainly Gram negative, such as CRK and Carbapenem-resistant *Pseudomonas* on mattresses.

**Conclusion:** Covers for hospital bed mattresses can be damaged, even if in normal visual inspection they are apparently in good condition. Addition of an opening test after patient's discharge can reveal spoil and be a sign to discard both covers and mattresses. In addition, there's an urgent need to improve technical specifications for manufacturers in order to increase safety. Mattresses can be the main environmental reservoirs for multi-drug resistant bacteria, contributing to outbreaks and endemic situations.
**Disclosure of Interest**
None Declared

### P411

#### Using a multidisciplinary approach in infection prevention and control to provide safe patient's care

Yveta Leharova, BScN, ICC, Ramona Rodrigues, BScN., M.Sc(A), CIC, CNS, IPC, FAPIC, Valerie Cass, BScN., MBA, Charles Frenette, MD, FRCP(C)
MONTREAL UNIVERSITY HEALTH CARE CENTER, Montreal, Canada
**Correspondence:** Yveta Leharova
*Antimicrobial Resistance and Infection Control* 2017, **6(Suppl 3):**P411

**Introduction: BACKGROUND:**
In April 2015 the Royal Victoria moved to a new 500 single bed hospital. Three months following the move, the 36 single bed transplant unit, declared a Vancomycin resistant enterococcus (VRE) outbreak and subsequently confirmed additional C.difficile (CDI) outbreaks.
**Objectives:** This retrospective comparison study focused on the impact of implementing a multidisciplinary prevention program over two fiscal years.
**Methods: METHODS:**
The Head nurse and Infection Control consultant were regularly implementing Environmental and hand hygiene audits. In October 2016 weekly multidisciplinary and bi-weekly housekeeping supervisors' huddles were held to discuss and implement corrective measures. Environmental and Hand Hygiene audit were collected pre/post implementation of the multidisciplinary prevention program. VRE and C.difficile cases isolates were molecular subtyped for some of the outbreaks to confirm transmission among patients.
**Results: RESULTS:**
Environmental/instrument cleaning and hand hygiene audits were poor in 2015/16 but improved during the course of the 2016/17. In 2016-17 there were no outbreaks on this single unit following close collaboration between Nursing, Infection Control and the multidisciplinary prevention initiative compare to previous year. Overall, the number of VRE nosocomial cases has decreased by 58% and CDI nosocomial cases decreased 38%.
**Conclusion:**
The multidisciplinary prevention program benefits patient's care in this hospital setting by providing a safe clean environment.
**Disclosure of Interest**
Y. Leharova, BScN, ICC Employee of: No conflict of interest, R. Rodrigues, BScN., M.Sc(A), CIC, CNS, IPC, FAPIC: None Declared, V. Cass, BScN., MBA: None Declared, C. Frenette, MD, FRCP(C): None Declared

### P412

#### The role of clean and safe health facilities(cash) initiative in infection prevention and control practices in selected health facilities, Ethiopia

Molla G. Fisehatsion
Health Service Quality Directorate, Federal Ministry of Health, Addis Ababa, Ethiopia
*Antimicrobial Resistance and Infection Control* 2017, **6(Suppl 3):**P412

**Introduction:** Making health facility clean and safe is an important determinant of quality of care and patient satisfaction. Health care provided in health facilities should be safe, effective, patient-centered, timely, efficient and equitable. Health facilities should ensure that patients/Clients are the corner-stone in the whole health care delivery process
**Objectives:** The ultimate goal of a clean and safe health facility (CASH) include clean, comfortable and safe environment for patients, attendants, visitors, staff and members of the general public; increased patient satisfaction and confidence in local health care settings in relation to environmental hygiene and the organizations commitment to reduce the incidence of hospital acquired infections
**Methods:** Out of the public hospitals which implemented CASH initiative, five of them were selected randomly.Regarding to CASH progress, national CASH audit tool which is very comprehensive was used and based on the audit tool a progressive assessment was conducted and compared with the baseline data.Regarding to infection prevention and patient safety practices, Ethiopian Hospitals Reform Implementation Guideline (EHRIG) report which consists IPPS as one chapter and collected at quarter basis was used
**Results:** When the CASH initiative was launched on October 2014, the hospitals infection prevention and control practices was very low. The score of Alamata, D/brhan, St.Peter, Minilik and Yekatit Hospitals was (62%, 70%, 40%, 35% &25%) respectively.
After the implementation of CASH initiative Alamata, D/Birhan, St.Peter, Minilik and yekatit (80%,84%,82%,75%, & 65%) respectively in 2016.
The mean score of the selected five hospitals in regard to infection prevention and control practices was 46% initially and it is improved to 77% in 2016.
**Conclusion:** The Ethiopian Federal Ministry of Health has been implementing this initiative in complement to the infection prevention and control program with high government commitment at all levels and creating a competitive environment among health facilities and bringing a successful result
**Disclosure of Interest**
M. Fisehatsion Employee of: Federal Ministry of health,Ethiopia: As part of a regulatory, legislative or judicial process, I haven't provided an expert opinion or testimony, related to the subject of the meeting or work, or a commercial entity or other organization

### P413

#### Eco-friendly hospitals – are they a risk for hospital hygiene?

Christiane Höller[1], Bertram Knörr[2]
[1]Hygiene, Bavarian Health and Food Safety Authority, Oberschleißheim; [2]Hygiene, Bavarian Health and Food Safety Authority, Erlangen, Germany
**Correspondence:** Christiane Höller
*Antimicrobial Resistance and Infection Control* 2017, **6(Suppl 3):**P413

**Introduction:** High energy costs as well as the trend to build environmentally sustainable has reached hospital architecture. In order to receive funds, hospitals in Bavaria have submitted during the last years several corresponding ideas to the ministry which finances hospital buildings. Some of these ideas give cause for concern.
**Objectives:** A hospital environment is energy consuming. A big part of the energy is allocated to ventilation, heating/cooling, and supply with hot water. Although some ways to save energy are conceivable, they can not be easily implemented in a hospital and some should not be considered at all.
**Methods:** The application documents, which had been submitted to the Bavarian Ministry for eco-friendly funding were examined for proposals with a hygienic risk.
**Results:** Ventilation systems in operation rooms can save energy by using overflowing air for adjoining rooms or by overnight shutdown, however some principles must be adhered to in order to guarantee a functioning ventilation. Passive ventilation, which is increasingly common in residential construction, must be viewed very critically due to the high levels of heat, moisture and odor in a hospital. As supply with hot water is very costly, there are attempts to lower these by lowering the water temperature. In order to prevent microbiological growth at these temperatures either a prophylactic disinfection or the insertion of ultrafiltration devices right after the water meter are modern countermeasures that create more problems than solutions.
In order to offer the patients nature-near materials and moreover to save chemicals and disinfectants there are attempts to switch to pure soap as a sole cleaning agent, preferably on parquet floors. While in the best case this can lead to high costs due to material damage, hygienic problems can also arise. Another way to save disinfectants is promoted by some companies that develop self-disinfecting surfaces. This might be a promising future technique, but there are still a lot of open questions that have to be answered before it can be recommended.
**Conclusion:** If an eco-friendly construction or operation of a hospital is planned, the infection control staff should be involved in advance in order to avoid risks for the patients and the staff.
**Disclosure of Interest**
None Declared

### P414

#### Cleaning and disinfection procedures in Bavarian Hospitals: survey of the current status

Ulla Kandler[1], Cora Ertl[2], Silke Nickel[1], Verena Lehner-Reindl[1], Christiane Höller[2]
[1]Hygiene, Bavarian Health and Food Safety Administration, Erlangen; [2]Hygiene, Bavarian Health and Food Safety Administration, Oberschleißheim, Germany
**Correspondence:** Ulla Kandler
*Antimicrobial Resistance and Infection Control* 2017, **6(Suppl 3):**P414

**Introduction:** Appropriate cleaning and disinfection of environmental surfaces plays a crucial role in infection prevention in hospitals. From the choice of detergents and disinfectants to the education of staff there are numerous critical points with the opportunity for mistakes.
**Objectives:** The purpose of this study was to assess current practice in cleaning and disinfection in Bavarian hospitals and to detect critical points, which need further attention and show room for improvement.
**Methods:** The department of hospital hygiene and infection prevention at the LGL created a standardized checklist which was sent to all local health administrations in Bavaria. They were asked to choose one area of interest in each hospital in their district, to inspect these areas including the observation of a cleaning and disinfection procedure. Results from the inspection were documented in the prepared checklist. The completed checklists were sent back to the LGL. The collected data were read into a data base and were analyzed with IBM SPSS Statistics 23.
**Results:** All 357 Bavarian hospitals were inspected with respect to their cleaning and disinfection procedures. Obligatory guidelines for these procedures were found in 99% of the visited wards. However, asking for details revealed discrepancies between the guideline and the situation seen during the inspection, because 15% of the procedures were not correctly performed. Other topics of concern were for example cleaning frequency on weekends and bank holidays, the adequate processing of used cleaning materials, the training of cleaning staff, the correct dosage of disinfectant and clarification of responsibilities especially with housekeepers from external enterprises.
**Conclusion:** All hospitals cover cleaning and disinfection in their standard operation procedures, but the discrepancies and inadequacies seen during the inspection show clearly that more emphasis is necessary in regard to structural and process quality. Out-sourcing may have an impact on the outcome of the overall quality. This and further questions like training of staff are looked into more thoroughly at the time being.
**Disclosure of Interest**
None Declared

### P415

#### Using education to improve the knowledge and beliefs of environmental service workers

Mei Wen Chen, I-Chen Hung
National Taiwan University Hospital, Taipei, Taiwan, Province of China
**Correspondence:** Mei Wen Chen
*Antimicrobial Resistance and Infection Control* 2017, **6(Suppl 3):**P415

**Introduction:** Improvements in hospital cleaning have been associated with reductions the risk of multidrug-resistant organisms transmission in hospital rooms. Understanding the knowledge and beliefs of environmental service workers(ESWs) may help to improve cleaning quality and to ensure effective practices.
**Objectives:** The aim of survey is using education to improve the knowledge and beliefs of ESWs.
**Methods:** We used pre-test and post-test of educational program to examine ESWs' beliefs and knowledge about environmental cleaning. Questions were developed based on observations of ESWs performing their duties and from previous studies. The education topics were included knowledge related to cleaning practices and workflow challenges.
**Results:** 120 ESWs participated in the survey, including 51 outsourcing staffs and 69 self-employed staffs. There were 72 ESWs (60%) had over three years experience in environmental services. After educational program, ESWs agreed that they had been trained to properly perform cleaning from 83.3% to 94.1%, ESWs were confident in their abilities to do so from 75.0% to 88.1%. In pre-test, only five of fourteen high-touch equipment were been considered to clean carefully by more than 90% ESWs, another three equipment, including suction controllers, vital signs monitor panels, respiratory ventilator controllers were ignored. After educational program, all of the high-touch equipment were be notice.
**Conclusion:** Through the educational program, we provide ESWs with knowledge and strategies to do environmental cleaning better and effective.

**References**
Bernstein DA, Salsgiver E, Simon MS, Greendyke W, Eiras DP, Ito M, Caruso DA, Woodward TM, Perriel OT, Saiman L, Furuya EY, Calfee DP. Understanding Barriers to Optimal Cleaning and Disinfection in Hospitals: A Knowledge, Attitudes,and Practices Survey of Environmental Services Workers. *Infect Control Hosp Epidemiol.* 2016 Dec;37(12):1492-1495.
Jennings A, Sitzlar B, Jury L. A survey of environmental service workers' knowledge and opinions regarding environmental cleaning. *Am J Infect Control.* 2013, 41(2):177-9.
Matlow AG, Wray R, Richardson SE. Attitudes and beliefs, not just knowledge, influence the effectiveness of environmental cleaning by environmental service workers. *Am J Infect Control.* 2012, 40(3):260-2.
Disclosure of Interest
None Declared

### P416

#### Investigations on the influence of architecture and design on the occurrence of nosocomial infections and multiresistant pathogens

Wolfgang Sunder, Jan Holzhausen
Technical University (TU) Braunschweig, IIKE, Braunschweig, Germany
**Correspondence:** Wolfgang Sunder
*Antimicrobial Resistance and Infection Control* 2017, **6(Suppl 3):**P416

**Introduction:** The study aims to provide important data for the decision as to whether, in the future, much more single bed rooms should be installed in hospitals in response to the increased incidence of multiresistant pathogens in Germany. Or, alternatively, to install upgraded twin rooms in the sense of infection prevention.
**Objectives:** National and international guidelines require the isolation of patients with multidirectional pathogens into single-bed rooms. However, the implementation of the recommendations has become ever more difficult due to the increasing incidence of these pathogens (1). As a result, some countries have begun to implement new hospitals with single-bed rooms only. This entails various disadvantages and higher costs (2), (3). A recent survey at 621 KISS hospitals showed that currently in Germany only 2 out of 36 beds per station can be found in single rooms (4).
**Methods:** Identification and evaluation of the infection-critical constructional interfaces and procedural sequences of the patient's room and sanitary cell (a). Creation of a requirements catalog and a infection-proof innovative concept (b). Development of drawing sets, detail solutions and cost estimation (c). Basic assessment of the demonstrator's suitability; Optimization (d).

**Results:** The aim of the study is to create an infection-proof twin room with a sanitary cell as an alternative to single rooms and to realize it as a demonstrator.
**Conclusion:** The consequences of the described architecture and design investigations have not yet been scientifically evaluated as a basis for decision-making. With the results an architect/hospitals is able to consider actions timely to support prevention in new building or alterative planning.

(1) Gastmeier P, Schröder C. Dramatic increase in vancomycin-resistant enterococci in Germany. J Antimicrob Chemother. 2014;Epub March 9.
(2) Pennington H, Isles C. Should hospitals provide all patients with single rooms? BMJ. 2013;347:f5695.
(3) Florey L, Flynn R, Isles C. Patient preferences for single rooms or shared accommodation in a district hospital. Scott Med. 2009;54:5-8.
(4) Stiller A, Sunder W. Untersuchung zur aktuellen Krankenhausbaustruktur in Deutschland im Hinblick auf die Infektionsprävention. in Vorbereitung zur Publikation. 2015.

Disclosure of Interest
None Declared

### P417

#### Prevention of healthcare-associated infections in intensive care units – potential for improvement due to construction design and operational actions

Inka Dreßler[1], Jan Holzhausen[2], Wolfgang Sunder[2], Harald Budelmann[1]
[1]iBMB; [2]IIKE, TU Braunschweig, Braunschweig, Germany
**Correspondence:** Inka Dreßler

*Antimicrobial Resistance and Infection Control* 2017, **6(Suppl 3):**P417

**Introduction:** The prevalence of healthcare associated infections (HAI) can be reduced with suitable actions by approximately 30% [1]. Considering exogenous infections, the relevance of infections induced by contact transmission(80-90%) outweigh infections induced by media(10-20%) [4]. Hence, the prevention of contact infections with construction design and operational actions in ICUs is focused.
**Objectives:** According to the multi-barrier concept to reduce the HAI-risk, there are several approaches conceivable: a) Physical barriers, b) hand hygiene infrastructure, c) cleaning and disinfectant strategies as well as d) construction design. Approaches a) und b) are described in literature [5] and are judged so far as inadequately implemented in Germany. It is the goal to provide a better basis for approach c) and d).
**Methods:** Therefore, cleaning und cleanability experiments are conducted and experts are interviewed.
**Results:** Proper hygienic implementation of cleaning routines in hospitals is indispensable since in literature (e.g. [2, 3]) onsets from surfaces in the environment are described. The conducted experiments show that there are different optimal cleaning strategies depending on the cleaning procedure. Moreover, structural ideal layouts for ICUs as well as recommendations for hygienic materials under consideration of the experiments are given.
**Conclusion:** Nowadays horizontal prevention measures in German ICUs are poorly integrated. Especially the risk for contact infections can be reduced by taking suitable measures into account. Analyzing prevalent deficits as well as the development of construction design and operational approaches such as an optimized cleaning strategy an improvement of the Status Quo is possible. With this an architect/ building engineer is able to consider actions timely to support prevention of HAIs in new building or alteration planning.

Disclosure of Interest
None Declared

### P418

#### Spatial remodeling of a central sterile supply department of a Brazilian University Hospital: a case report

Christian E. S. Pelaes, Rodrigues, CM; Napoleao, AA; Locilento, AFB; Lucenti, FL; Avo, LRS; Gabassa, VC; Neves, FF; Suzuki, MN; Martins, BR; Figueiredo, RM
Health Surveillance and Patient Safety Department, UNIVERSITY HOSPITAL OF UNIVERSITY OF SAO CARLOS / EBSERH, Sao Carlos, Brazil
**Correspondence:** Christian E. S. Pelaes

*Antimicrobial Resistance and Infection Control* 2017, **6(Suppl 3):**P418

**Introduction:** The central sterile supply department (CSSD) comprises that service within the hospital in which medical/surgical supplies and equipments are prepared for reuse. The steps for processing each material include:pre-cleaning, reception,cleaning,-drying,evaluation,preparation,disinfection/sterilization,storage,distribution. The CSSD physical layout should favor a unidirectional flow, avoiding the crossing of cleaned and contaminated materials [1-2].
**Objectives:** To report the adequacy of a CSSD remodeling regarding to American and Brazilian architectural policies
**Methods:** To describe the experience of remodeling a CSSD, from September 2015 to May 2016 in a university hospital founded 10 years ago in Sao Paulo state. After a service inspection realized by the Infection Prevention and Control Unit, gaps were identified in the infrastructure area that compromised the materials cleaning and disinfection process and the professional's safety. Emergency measures were approved with a new model of physical layout and remodeling of the CSSD.
**Results:** Specific areas were built for each stage of material processing system: A dirty area (purge), exclusive changing room/toilet, exclusive chemical disinfection room and cleaning supply section into the dirty and clean areas. Employees no longer move freely from one controlled area to another. The preparation and sterilization areas were preserved. The storage area was separated into two enclosures (disinfection and sterilization) with humidity and temperature control. It was possible to build an area for monitoring technical actions in conjunction with administrative activities, besides an exclusive pantry to promote a coexistence environment for the employees.
**Conclusion:** It was able to comply with the regulations established by Centers for Disease Control (CDC) and policies from the National Health Surveillance Agency from Brazil (ANVISA) that contribute to the patients and healthcare workers' safety, as well as prevent infection in the hospital environment.

Disclosure of Interest
None Declared

Download to read this full article text.

